# Rate-Tunable, Metal-Mediated Amide Bond Cleavage for the Controlled Release of Pharmaceuticals

**DOI:** 10.1021/jacs.5c13166

**Published:** 2025-11-07

**Authors:** Zhuoran Zhong, Dariusz Śmiłowicz, Mallory J. Gork, Leah C. Garman, Ilia A. Guzei, Eszter Boros

**Affiliations:** Department of Chemistry, 5228 University of Wisconsin-Madison, Madison, Wisconsin 53706, United States

## Abstract

That the incorporation of *N*-methyl amino acids adjacent to a hydrolytic, azamacrocyclic metal complex results in rate-tunable, metal-mediated amide bond cleavage (TMAC) under physiological conditions. Spectroscopic and crystallographic data provide unprecedented mechanistic insight: the Ga^3+^ complex of (7-amido-1,4,7-triazonane-1,4-diyl)­diacetic acid polarizes the amide bond proximal to canonical and noncanonical amino acids, forming two coordination isomers with different cleavage rates, N_3_O_3_ (fast) and N_4_O_2_ (slow) in aqueous solution. Both were characterized by NMR spectroscopy and identified by single-crystal X-ray diffraction. Subsequent hydrolysis of the amide bond occurs by exogenous nucleophilic attack, as demonstrated by ^18^O-isotope labeling experiments and proceeds with a variable rate, depending on the nature of the amino acid side chain and amide-methylation status. The in vivo applicability of TMAC was subsequently demonstrated by pharmacokinetic modulation of a cancer targeted, ^68^Ga-labeled radiopharmaceutical. Specifically, 6 serum-albumin binding chelates, linked to a peptide targeting the prostate specific membrane antigen (PSMA) were constructed. Variable amino-acid-chelate linkers allow tuning of the rate of release and clearance of the radioactive isotope. Indeed, diagnostic positron emission tomography (PET) imaging, metabolite and biodistribution analysis indicate that rate tunable cleavage and release of the ^68^Ga-chelate minimize tracer accumulation in blood and liver compartments while maximizing tumor uptake. In contrast, a [^68^Ga]­Ga-chelate incorporating a noncleavable glycine linker, exhibited elevated blood and liver uptake with moderate tumor localization. Taken together, TMAC provides remarkable control over the in vivo behavior of targeted pharmaceuticals.

## Graphical abstract

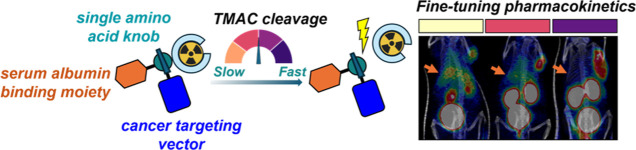

## Introduction

Prodrug activation enables spatiotemporally controlled release and activation of therapeutic agents within target tissues.^1–3^ To this end, stimuli-activated prodrugs incorporating transition metals have attracted considerable interest owing to their distinctive features, such as tunable redox states, varied coordination geometries, and catalytic activity.^4–6^

For instance, numerous Pt­(IV)-based prodrugs, such as satraplatin and miriplatin, have been developed to mitigate the off–target toxicity of first-generation platinum chemotherapeutic agents cisplatin and oxaliplatin, leveraging their six-coordinate octahedral geometry to prevent unfavorable interactions with DNA.^7–10^ Drug activation can be achieved by exogenous X-ray irradiation or photons to trigger conversion of Pt­(IV) prodrugs to the biologically active Pt­(II) species.^11,12^ Beyond platinum-based systems, exogenous transition metal-mediated prodrug activation strategies have been developed successfully and validated in vitro. For instance, Bernardes and co-workers pioneered a [AuCl_4_]^−^-induced amide bond cleavage strategy to achieve the release of dual-functional therapeutic agents. This strategy was successfully validated in a zebrafish model, demonstrating its potential for in vivo applications (Figure 1).^13^

**Figure 1:**
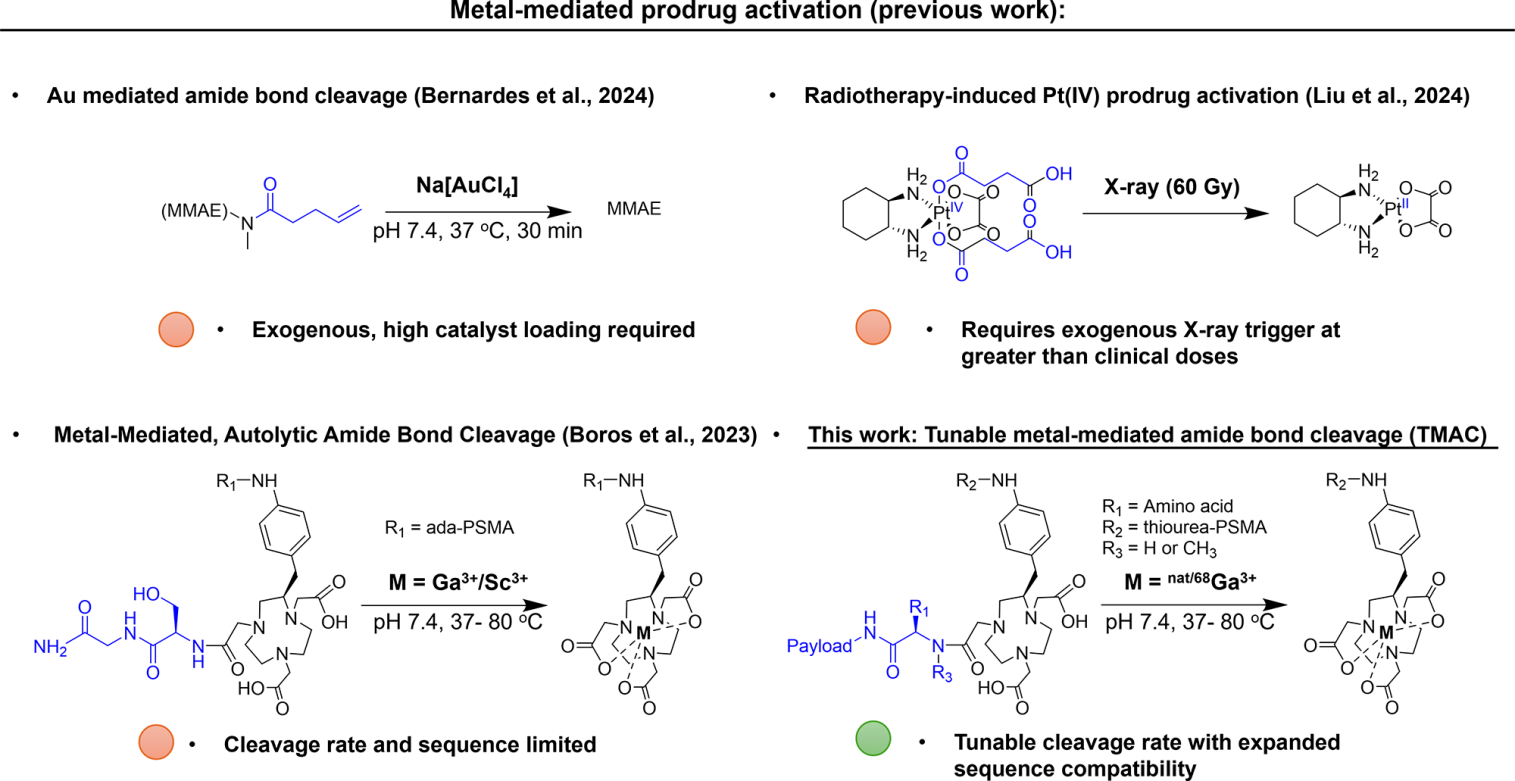
Summary of transition metal-mediated prodrug activation strategies. Conventional approaches require external stimuli such as transition metal treatment or X-ray irradiation, or are constrained by specific peptide sequence requirements. In contrast, TMAC utilizes endogenous metal ions to mediate amide bond cleavage, with kinetics readily tunable by either adjacent amino acids or the identity of the metal centers.

Despite the attractive features of transition metal platforms for prodrug activation, the need for external triggers or biocompatible, organometallic catalysts colocalizing with the prodrug at the site of interest, remain a critical barrier to preclinical and clinical translation.^14–18^

A potential strategy to address these challenges is the implementation of a single-molecule, modular metallo-prodrug activation strategy with an endogenous activation trigger. In nature, metallo-proteases can selectively recognize and cleave amide bonds, which otherwise are considered exceptionally inert, with a half-life of ∼350–600 years at room temperature under physiological conditions.^19,20^ A variety of metalloproteases utilize metallo-cofactors, including Zn^2+^, Mn^2+^, or Fe^2+^, along with specific active-site residues to achieve highly selective and efficient peptide-bond hydrolysis.^19–22^ Inspired by these enzymes, Groves and co-workers demonstrated that a small-molecule Co^3+^ chelate system could promote amide bond hydrolysis by coordination of a ternary hydroxide which acts as a nucleophile to hydrolyze the amide bond.^23^ In a related study, Burstyn showed that a nontethered small Cu^2+^ complex could hydrolyze both the inactivated dipeptide Gly–Gly and bovine serum albumin under physiological conditions.^24^ Bal and co-workers demonstrated that Ni­(II) aqua ions could selectively hydrolyze amide bonds adjacent to serine and threonine residues at pH > 8.5.^25,26^ However, these systems all had significant shortcomings, preventing their application to functional metallodrugs in vivo: the metal ions, even if chelated, were coordinatively undersaturated and therefore labile and incompatible with more complex biological environments.^27–29^

Previously, our group introduced metal-mediated autolytic amide bond cleavage (MMAAC) in which Lewis acidic metal ions, such as Ga^3+^ and Sc^3+^, chelated by (7-amido-1,4,7-triazonane-1,4-diyl)­diacetic acid, trigger the rearrangement and cleavage of a serine residue adjacent to the metal complex via an N,O acyl shift, followed by ester hydrolysis (Figure 1).^30^ This approach was successfully applied to solid-phase ion separation and in vivo release of a model serine-ibuprofen conjugate labeled with the radioactive isotope ^67^Ga (*t*_1/2_ = 78.3 h). However, this system presented with several shortcomings: (1) sequence dependence on serine as the chelate-adjacent amino-acid, and (2) the lack of reaction rate tunability. This posed significant limitations on applying MMAAC to endogenously triggered, in vivo compatible metalloprodrug systems.

Herein, we report the evolution of this concept, termed tunable metal-mediated amide bond cleavage (TMAC). We demonstrate that *N*-methylated, noncanonical amino acids unlock a secondary reaction pathway by exogenous nucleophile attack, circumventing the need for a nucleophilic amino acid side chain to induce N, O acyl shift. Spectroscopic and crystallographic tools provide unprecedented insight into the underlying reaction mechanism. In contrast with MMAAC, TMAC rate is readily tunable by modulation of complex adjacent amino acids and accelerated to biologically relevant release rates by *N*-methylation. Exploration of proof-of-concept, targeted conjugates incorporating TMAC in a mouse model reveals that the cleavage mechanism is cargo agnostic and in vivo compatible, indicating that TMAC is compatible system.

## Results and Discussion

### Complex Adjacent Amino Acid Screening and Scope

Following our previous work focused on N, O acyl shift compatible chelate conjugates, we originally sought to accelerate amide bond cleavage by *N*-methylation, to enhance the peptide’s leaving group character. We initiated the study by synthesis of a model tripeptide sequence which is capped at the N-terminus by the aza-macrocyclic 1,4,7-triazacyclononane-1,4,7-triacetic acid (NOTA) chelator previously identified as compatible with MMAAC. To this end, tripeptides with the sequence NO2A-X-G-W-NH_2_ (where X represents the variable complex adjacent amino acid) were constructed. Tryptophan (W) was incorporated as a spectroscopic handle for HPLC monitoring due to its characteristic absorbance at 280 nm, while glycine (G) was included as a short spacer amino acid to minimize the steric influence of W on the metal chelate.^30^ Subsequently, chelation with Ga^3+^ yielded the corresponding model tripeptide metal complex, [Ga­(NO2A)]^+^-X-G-W-NH_2_. The Ga^3+^ ion was selected as the gold-standard metal ion since it effectively triggered bond cleavage in the Ser model tripeptide in prior studies, but also formed a thermodynamically and kinetically inert, in vivo compatible metal complex.^30^ Following successful complexation and purification, the pH and reaction temperature of the metal complex solution were adjusted to pH 7.4 and 80 °C to initiate cleavage at a readily observable rate (Figure 2A). Reaction progress was monitored and quantified using high-performance liquid chromatography and mass spectrometry (HPLC-MS, Figure 2B,C). In the absence of a metal ion, all model tripeptides remained intact at all conditions and temperatures tested (Figures S113–S121). As anticipated based on our previous work, amide bond hydrolysis was observed for serine (Ser) and threonine (Thr), owing to the presence of nucleophilic hydroxy groups in their side chains, in accordance with the mechanistic hypothesis that the amide bond hydrolysis proceeds through N, O acyl shift (Figures S158, S159). In the liquid chromatogram, a second peak with a longer retention time, with the same mass as the reactant, was observed. Previously, we hypothesized that this peak correlated to an intramolecular rearrangement prior to the subsequent amide bond cleavage as part of the N, O-acyl shift reaction. However, control peptide sequences, incorporating glycine and β-alanine as the linking amino acid also displayed the formation of a persistent isomer peak (Figures S149, S153) that did not progress to release the tripeptide. As glycine and β-alanine side chains contain no nucleophilic substituents and cannot trigger the N, O-acyl shift reaction, this indicated that a second, possible mechanism of complex isomerization was operational.

**Figure 2:**
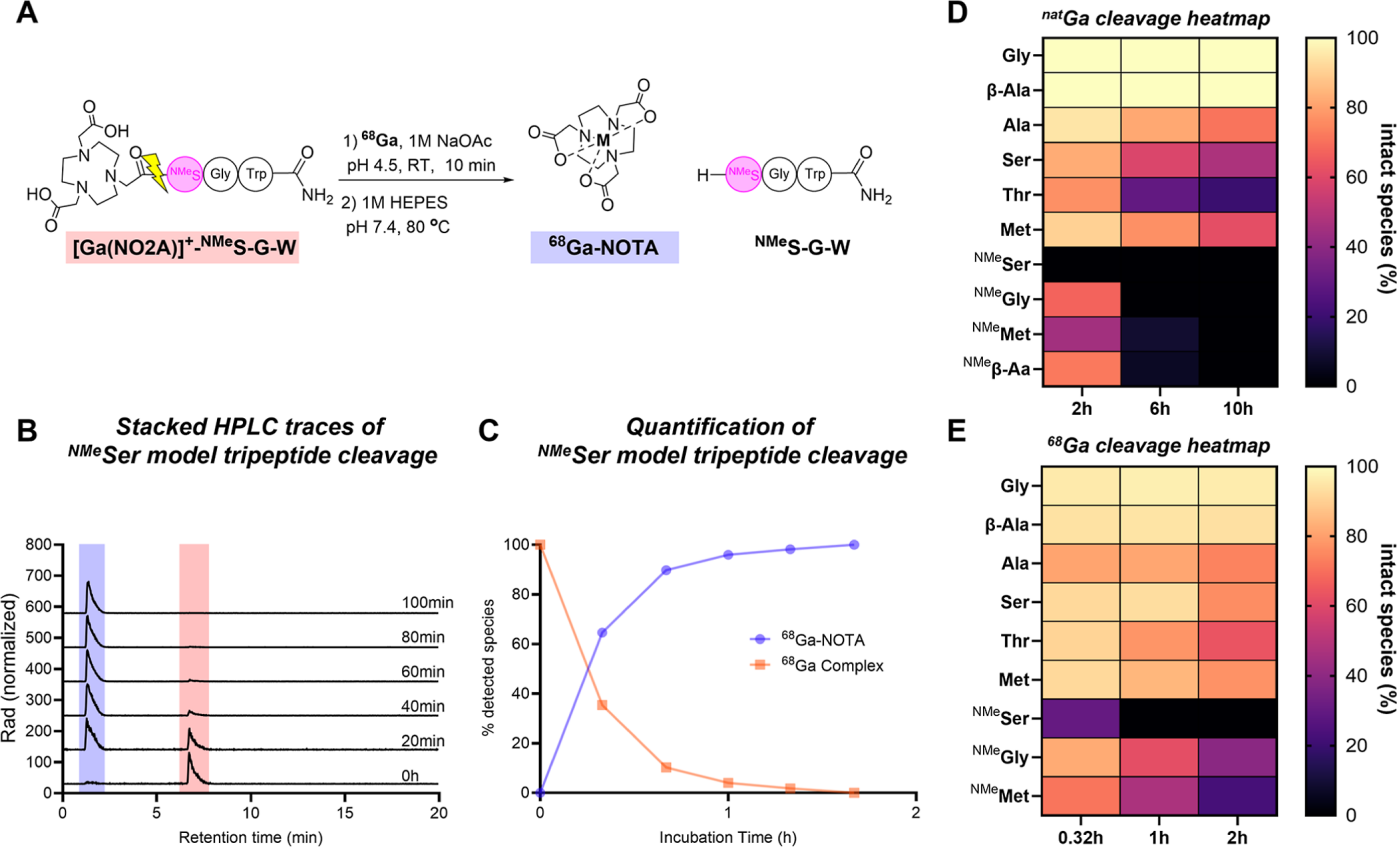
(**A**) Stepwise complexation of natural gallium (^nat^Ga) and radiochemical labeling of ^68^Ga with a *N*-methylated Serine-based model tripeptide, alongside the reaction scheme for monitoring autolytic amide bond cleavage. (**B**) Stacked HPLC chromatograms tracking the progression of amide bond cleavage at 80 °C and pH 7.4. (**C**) Quantification of the cleavage at 80 °C and pH 7.4. (**D**) Comparative heatmap illustrating time-dependent ^nat^Ga-driven autolytic amide bond cleavage kinetics across tested model tripeptide systems. (**E**) Comparative heatmap illustrating time-dependent ^68^Ga-driven autolytic amide bond cleavage kinetics across tested model tripeptide systems.

We probed the reactivity profiles of noncanonical, methylamide derived amino acid containing sequences next. We hypothesized that the rate of amide bond hydrolysis could be accelerated significantly by designing peptide sequences that act as a bulkier leaving group. To test this hypothesis, we synthesized a tertiary amide bond containing model sequence incorporating *N*-methyl serine (^NMe^Ser), which efficiently promoted amide bond cleavage (Figure S175). However, additional peptide sequences containing complex-adjacent alanine (Ala) and methionine (Met) also resulted in amide bond cleavage (Figures S156, S164). Furthermore, the tertiary amide conjugate ^NMe^Gly also exhibited efficient amide bond cleavage under the same conditions, with cleavage rates exceeding those of the nonmethylated canonical amino acids (Figure 2D). These observations were at odds with the metal-complex-induced amide bond hydrolysis proceeding exclusively by N, O acyl shift for efficient amide bond cleavage. Reaction rates were revealed to be variable, ranging from comparatively slow (Ala, 20% cleavage after 6 h) to moderate (Thr, 50% cleavage after 6 h) to fast (^NMe^Ser, 100% cleaved after 1 h). Cysteine and selenocysteine linker systems presented with significant side product formation because of their oxidative reactivity and therefore were not further investigated.

To investigate if the observed reaction rates and reactivity trends were concentration dependent, we conducted cleavage experiments with the positron emitting radioisotope ^68^Ga (*t*_1/2_ = 68 min). Cleavage rates were reproduced under radiochemical, tracer conditions employing ^68^Ga-radiolabled complexes, indicating that cleavage kinetics were governed by the complex’s reactivity (Figure 2E) and were not influenced by the relative hydroxide concentration. In addition to probing concentration dependence, these experiments also allow for the direct detection and quantification of the released [^68^Ga]­Ga­(NOTA) chelate complex (Figure 2B, E), providing complementary information to the macroscopic experiments where the tripeptide is detected and quantified (Figures S211–S219). In all instances, no side reactions were observed, and the reaction led to the formation of inert products without further proteolytic degradation.

To assess the ability of different metal centers to induce TMAC, we conducted cleavage experiments using Gly (10) and ^NMe^Gly (17) conjugates. In good correlation with our previous findings for Cu^2+^, Zn^2+^ complexes were inert toward direct amide bond hydrolysis at pH 7.4 and 80 °C (Figure S144, S145). In contrast, the trivalent metal centers Fe^3+^ and In^3+^ exhibited faster cleavage than Ga^3+^ for both conjugates (Figure S144, S145). These experiments indicate that TMAC is compatible with other Lewis acidic, trivalent metal centers, whereas divalent ions show no reactivity.

These results, while encouraging with respect to accessing tunable payload release, indicated that mechanistic studies were required to provide insight into the putative, secondary cleavage mechanism.

### Mechanistic Studies

Given that amide bond cleavage was also observed for amino acids without a nucleophilic side chain (required for N, O acyl shift reactions), we postulated that external nucleophiles, specifically H_2_O and OH^–^, play a critical role in facilitating amide bond hydrolysis.

To this end, we first probed the influence of increasing pH on cleavage kinetics. Specifically, we conducted pH-dependent cleavage experiments at pH 4.5, 6.5, and 8.5 (80 °C). Under these conditions, divergent trends in reaction rate were observed for canonical amino acids when compared with the *N*-methylated counterparts (Figure 3A). Specifically, canonical amino acids reacted fastest at low pH conditions, with a drop-off in rate at and above pH 6.5. This behavior was consistent for all model systems, Gly, Ala and Met, studied for pH dependence. Notably, the Gly linked peptide was also hydrolyzed at pH 4.5, whereas it remained inert at pH > 5. In contrast, *N*-methylated amino acid conjugates exhibited the inverse trend: both ^NMe^Gly and ^NMe^Met conjugates showed acceleration of cleavage rate with increasing pH. This correlated well with our original hypothesis that an increase in OH^–^ ion concentration accelerates the amide bond hydrolysis. We concluded that other analytical methods, specifically pH-dependent ^1^H NMR spectroscopy, were required to gain additional insight into the divergent reaction rate trends. Indeed, spectral data of both classes of compounds showed distinct behavior. Figure 3B,C shows stacked ^1^H NMR spectra of [Ga­(NO2A)]^+^-Ala-Gly-Trp (left) and [Ga­(NO2A)]^+^-Gly-Gly-Trp (right), respectively. Diagnostic chemical shift regions in immediate vicinity of the metal center, such as the amide-methylene, as well as those characteristic for the amino acid side chain offer valuable insight into the pH-dependent behavior of the corresponding complexes. Indeed, the ^1^H NMR spectra of the [Ga­(NO2A)]^+^-Ala-Gly-Trp (Figure 4C, left), reveal the presence of two distinct coordinative species dominating at low and high pH, respectively. Quantitative integration of the gradual interconverting signals at 4.30 and 4.22 ppm to 3.76 and 3.71 ppm determines a p*K*_a_ value of 6.33 for the interconversion. In absence of Brønsted acids with correlating p*K*_a_ on the molecular scaffold, we posit that the immediate vicinity of the amide proton to the Ga^3+^ metal center lowers the p*K*_a_ by several orders of magnitude. The corresponding deprotonation event results in transformation of the coordinative donor environment from N_3_O_3_ to N_4_O_2_ (Figure 3B). The ^1^H NMR spectra of the [Ga­(NO2A)]^+^-Gly-Gly-Trp and [Ga­(NO2A)]^+^-Met-Gly-Trp complexes exhibit comparable p*K*_a_ values (5.72 and 6.40, respectively), further supporting consistent formation of the proposed coordination isomers (Figures S251, S255).

**Figure 3:**
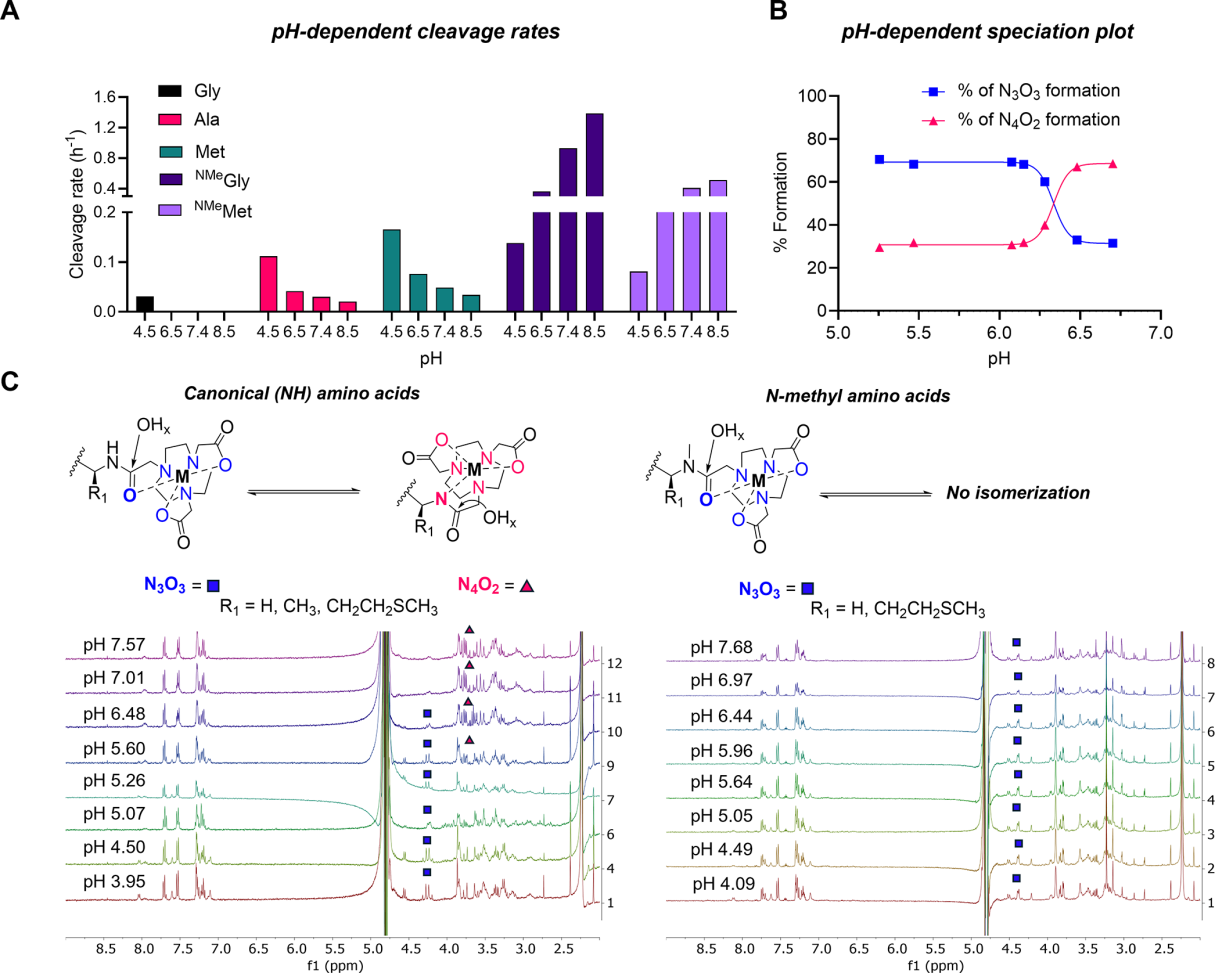
(**A**) pH-dependent autolytic cleavage profiles (pH 4.5, pH 6.5, pH 7.4, pH 8.5) for model tripeptides containing Gly, Ala, Met, ^NMe^Gly, and ^NMe^Met. (**B**) pH-dependent speciation plot (based on ^1^H spectra) showing equilibrium transformation between N_3_O_3_ and N_4_O_2_ coordination geometries of the [Ga­(NO2A)]^+^-Ala-Gly-Trp complex. (**C**) Left: schematic description of equilibrium isomerization between N_3_O_3_ and N_4_O_2_ for canonical amino acids with pH-dependent ^1^H NMR spectra (400 MHz in H_2_O with 0.1 M KCl and 0.01 M HCl, pH 3.95–7.57) of [Ga­(NO2A)]^+^-Ala-Gly-Trp. Right: schematic description of fixed isomer N_3_O_3_ for *N*-methylated amino acids with pH-dependent ^1^H NMR spectra (400 MHz in H_2_O with 0.1 M KCl and 0.01 M HCl, pH 3.95–7.57) of [Ga­(NO2A)]^+^-^NMe^Gly-Gly-Trp. Both spectra are referenced to trimethylsilyl propanoic acid (TSP).

**Figure 4:**
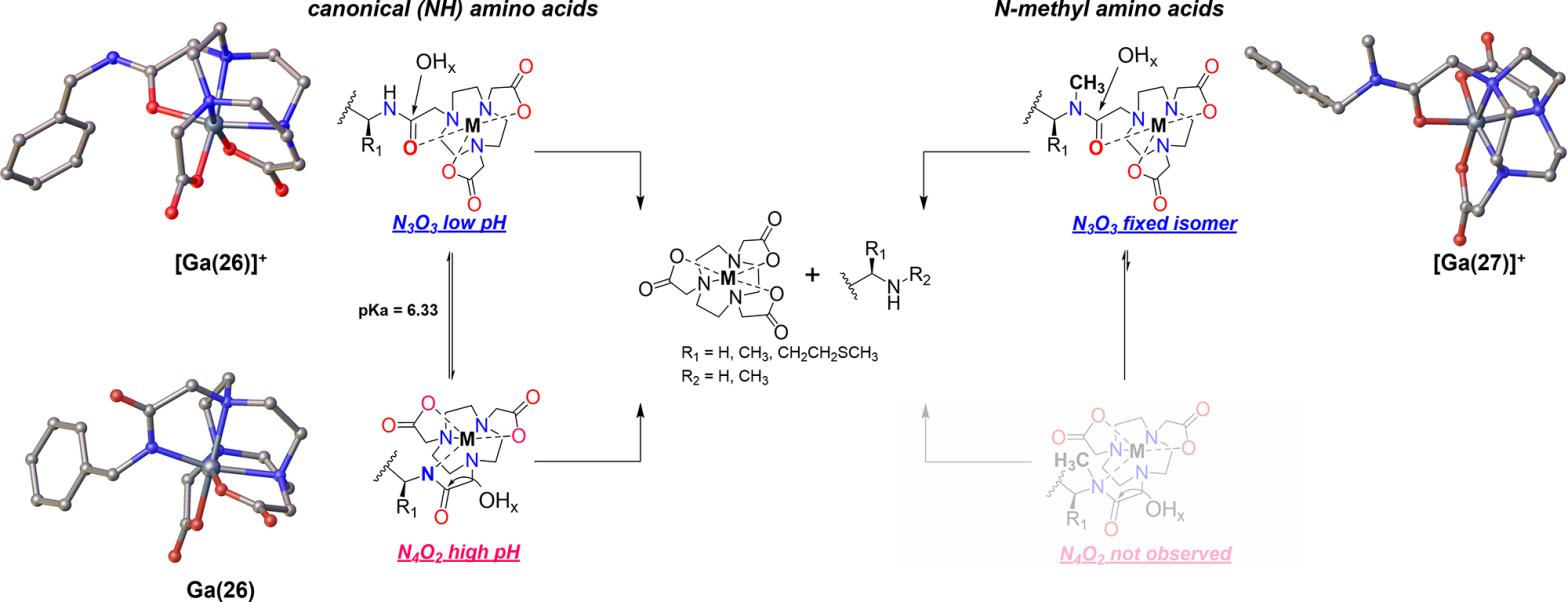
Schematic description of cleavage mechanism by exogenous hydroxide/hydrido attack for canonical amino acids (left), with corresponding representations of X-ray crystal structures of [Ga­(**26**)]­ClO_4_, Ga­(**26**)·H_2_O and mechanism proposed for *N*-methylated amino acids proceeding through a single, “isomer-locked” coordination complex, further evidenced by the model complex structure of [Ga­(**27**)]­ClO_4_·4H_2_O. Crystal structures are shown as ball and stick diagrams , and omission of hydrogens, water molecules and counterions.

In contrast, the ^1^H spectra of the [Ga­(NO2A)]^+^-^NMe^Gly-Gly-Trp showed no spectral changes, indicating that methylation of the amide nitrogen “locks” the N_3_O_3_ complex species configuration (Figure 3C). Combined with the rate trends observed for the pH-dependent cleavage, we propose that the N_3_O_3_ species mediates a rapid, base-catalyzed amide bond hydrolysis reaction, whereas the N_4_O_2_ species is also compatible with exogenous nucleophile attack, but with a slowed rate of release (Figure 3C).

To further affirm this mechanistic hypothesis and the formation of coordinative isomers, we synthesized benzyl-functionalized NO2A model ligands 26 and 27 (Supporting Information Scheme S5, S6) as analogues of canonical and *N*-methylated amino acids, respectively. The corresponding Ga^3+^ complexes were formed, and the pH of the complex solution was either retained at pH < 3 or adjusted to 7. Crystals suitable for single-crystal X-ray diffraction were obtained and analyzed. Figure 4 shows the corresponding crystal structures of [Ga(26)]^+^ (low pH, N_3_O_3_ isomer), Ga(26) (high pH, N_4_O_2_ isomer), as well as the *N*-methylated, “isomer-locked” structure of [Ga(27)]^+^ (N_3_O_3_ isomer is prevalent at low and high pH conditions). Similar observations made by Chung and Que on homologous systems further support the proposed pH dependent isomerization.^31,32^

An alternative, intramolecular attack by the neighboring carbonyl amide via Robinson annulation remains a possibility for both isomers.^33^ To address this possibility, we measured the rate of reaction of the ^NMe^β-Ala-linked model peptide conjugate, which would form a 7-membered species with a rate of reaction orders of magnitude slower if proceeding through Robinson annulation. However, this model conjugate produced similar cleavage rates to the corresponding ^NMe^β-alanine (0.88 h^–1^ at pH 7.4, Figure S183), making this intramolecular nucleophilic attack less probable.

Cleavage experiments conducted in H_2_^18^O with [Ga­(NO2A)]^+^-^NMe^Gly-Gly-Trp and [Ga­(NO2A)]^+^-Ser-Gly-Trp produced isotopically labeled Ga­(NOTA), incorporating a single ^18^O atom, further supporting exogenous nucleophilic attack by hydroxo/hydrido species (Figures S259–S261). It is noteworthy that the cleavage of constructs containing amino acids with nucleophilic side chains, such as Ser or Thr, may proceed simultaneously through N, O-acyl shift mediated intramolecular nucleophilic attack, and by exogenous hydroxide attack. As such, isotope labeling experiments in H_2_^18^O cannot readily distinguish between these due to the involvement of an exogenous nucleophile during the ester hydrolysis step following N, O-acyl shift rearrangement.^30^

Therefore, we conducted additional Kinetic isotopic effect (KIE) measurements for Met (15) and ^NMe^Met (18) conjugates, which were chosen to avoid alternative reaction mechanisms. Our experiments determined KIE values (1.46 for Met, 2.81 for ^NMe^Met) that imply a secondary KIE, where the proton transfer is not rate-limiting (Figures S262–S265). Furthermore, cleavage kinetics under various buffer conditions and concentrations indicated that rates remained consistent in hydroxyethylpiperazine ethanesulfonic acid (HEPES), piperazine-*N*,*N*′-bis­(2-ethanesulfonic acid) (PIPES) (Table S1), further supporting the robustness of the reactivity paradigm. Reaction rates in phosphate buffered saline (PBS) were accelerated, motivating investigation of biologically relevant systems.

### Constructing a Radiopharmaceutical Compatible with TMAC

Given the compatibility of TMAC with physiological conditions, we hypothesized that the hydrolysis of the metal complex could be employed to release a payload in vivo at different rates. Specifically, TMAC provides an ideal strategy to fine-tune the pharmacokinetics of radiopharmaceuticals. Specifically, we sought to balance increased tumor uptake, with limiting exposure to radiosensitive organs. We proposed that induction of hydrolysis to promote clearance of the radioactive payload would readily prevent prolonged circulation and exposure to healthy, radiosensitive tissues such as the heart, liver and marrow.

Indeed, most currently utilized peptide-based radiopharmaceuticals such as ^177^Lu-PSMA-617 (Figure 5, left), a clinically approved radiopharmaceutical for the treatment of prostate specific membrane antigen (PSMA)-expressing prostate cancer, consist of chelate-peptide conjugates with short in vivo half-lives, delivering most of the radioactive dose to the kidneys and bladder by rapid excretion, while limiting delivery to the tumor. Efforts to slow blood retention by incorporation of blood serum albumin binding moieties have demonstrated success in preclinical and clinical studies.^34,35^ However, while these constructs provide enhanced tumor uptake of the radioactive payload, they also significantly increase radiation burden to radiosensitive organs. For instance, our previous work on an iodophenyl-modified, PSMA-targeting chelate significantly enhanced blood half-life and dose to the tumor in mice, but also delivered doses to the marrow above the FDA-recommended dosimetry threshold.^36^ Similarly, a construct incorporating iodo-tyrosine to enhance blood circulation in clinical trials, termed PSMA I&T (Figure 5, middle), resulted in myelosuppression and renal radiotoxicity in patients treated with the ^177^Lu therapeutic. This underscores the need for improved pharmacokinetics of such agents.^37^

**Figure 5:**
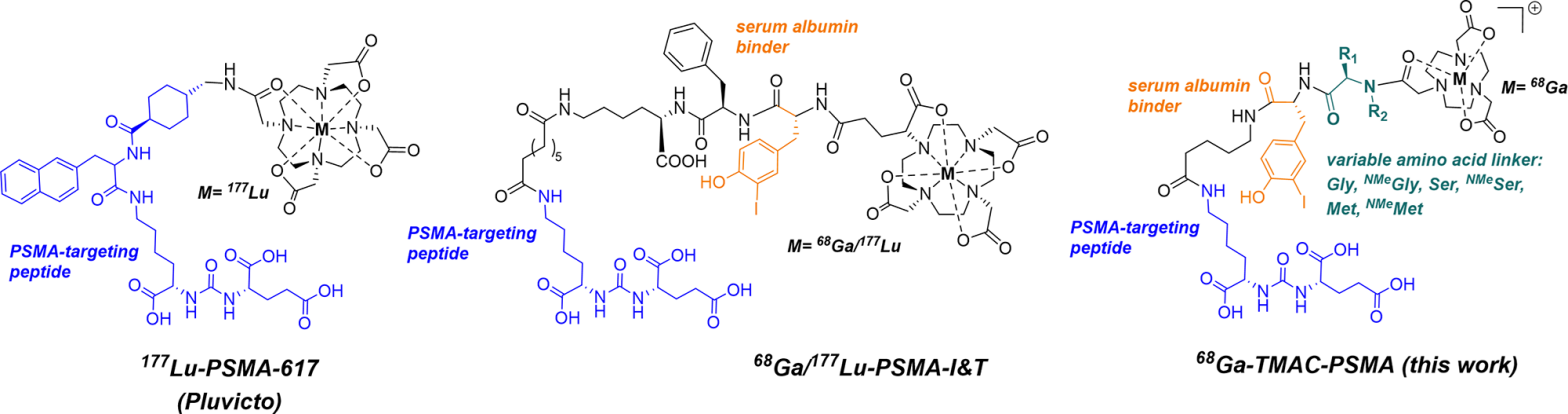
Chemical structure of the FDA approved radiotherapeutic ^177^Lu-PSMA-617 and the serum-albumin binding ^177^Lu/^68^Ga-PSMA-I&T in current clinical trials, with prolonged blood residency achieved by serum albumin binding and structure of the ^68^Ga-TMAC-PSMA system with variable amino acid linkages to produce modulation of blood residency time by rate-selective release of the radiochelate.

To this end, we designed a class of molecules compatible with TMAC. The corresponding six, targeted derivatives of [Ga­(NO2A)]^+^-R (where R represents a variable amino acid linker) conjugated to a serum albumin binder, 3-iodo-tyrosine and a prostate-specific membrane antigen (PSMA) targeting vector, hexKuE, Figure 5, right).^38^ Based on screening data (Figure 2) with the model tripeptides, we selected derivatives with a range of cleavage rates from noncleavable (Gly), to slow cleaving (Met < Ser < ^NMe^Gly) to rapidly cleaving (^NMe^Met < ^NMe^Ser). The synthesis of targeted derivatives was achieved by solid-phase peptide synthesis (Scheme S4).

We first evaluated whether the amide bond hydrolysis rate of the functionalized derivatives is agnostic to the cargo. We found that the targeted constructs displayed half-lives of amide bond hydrolysis consistent with those of the corresponding model tripeptides (Table 1). Furthermore, experiments conducted at 37 °C and in the presence of mouse plasma confirmed previously determined trends in reactivity, including a vastly accelerated cleavage rate for *N*-methylated amino acid derivatives. HSA binding affinity measurements confirmed comparable binding affinity for all conjugates, indicating that the change of the amino acid linker did not have a significant impact on the conjugate’s ability to bind serum albumin (Table S3).

**Table 1:** Characterization Parameters for Select TMAC Conjugates Including Cleavage Half-Life, Cleaved Metabolite, and Tumor Uptake

amino acid linker R	half-life of model tripeptide (h)	half-life of PSMA-conjugate (h)	% cleaved complex in 2 h p.i. urine metabolite	tumor uptake at 2 h p.i. (% ID/g)
Gly (20)	not observed	not observed	4.0 ± 0.8	8.3 ± 0.1
Ser (21)	365^a^/182^b^	533^a^/408^b^	10.8 ± 1.9	10.2 ± 2.7
Met (22)	408^a^/239^b^	462^a^/462^b^	6.0	7.5
^NMe^Gly (23)	35.9^a^/49.5^b^	38.3^a^/32.3^b^	7.4 ± 1.3	15.2 ± 6.9
^NMe^Ser (24)	3.65^a^/6.12^b^	2.52^a^/2.49^b^	32.3 ± 2.1	19.6 ± 3.8
^NMe^Met (25)	28.1^a^/151^b^	27.0^a^/22.4^b^	5.9	3.5

a Comparative amide bond cleavage half-lives of ^67^Ga-radiolabeled model tripeptide complexes and corresponding functionalized conjugates performed in 0.25 M pH 7.4 HEPES buffer at 37 °C.

b Performed in a 1:1 mixture of HEPES buffer and mouse plasma at 37 °C. The half-lives of Ser (21) and Met (22) conjugates are estimated based on pseudo first-order kinetics.

With the constructs validated in vitro, we next sought to probe the impact of different rates of cleavage on the pharmacokinetic behavior of the corresponding, ^68^Ga-radiolabeled conjugates. To this end, we first radiolabeled the conjugates with ^68^Ga isotope at a consistent molar activity of 10 nmol/mCi. We employed a bilateral tumor model with a PSMA-expressing tumor on the right flank and a PSMA-negative tumor on the left flank. Each cohort was imaged by positron emission tomography-computed tomography (PET-CT) at 30, 60, and 90 min post injection, followed by terminal biodistribution and urine metabolite analysis at 120 min.

Taking into consideration the characterized cleavage rates, we postulated that the conjugates with cleavable linkers, Ser (**21**), ^NMe^Gly (**23**), and ^NMe^Ser (**24**), would exhibit shorter biological half-lives and reduced off–target activity, whereas the construct with a noncleavable linker, Gly (**20**), would show longer biological half-life and prolonged retention in blood pool and liver (Figure 6A). Indeed, PET-CT images revealed that both [^68^Ga]­Ga-**20** (Gly), and the slow-cleaving conjugate [^68^Ga]­Ga-**21** (Ser), exhibited prolonged blood circulation, evidenced by the enhanced heart and liver uptake visible in PET images at all three time points (Figures 6B, S267–S269). This is in line with expectations, with previous work on serum albumin binding radiopharmaceuticals demonstrating elevated liver and blood/heart uptake at early time points.^34,37^ In contrast, the more rapidly cleaving [^68^Ga]­Ga-**23** (^NMe^Gly) and [^68^Ga]­Ga-**24** (^NMe^Ser) demonstrated decrease in liver uptake and lowered blood pool retention, paired with efficient renal clearance (Figure 5B). Further affirmation for in vivo TMAC activity is provided by analysis of the urine metabolites (Figure 6C): the cleavage product [^68^Ga]­Ga­(NOTA) is readily observed for cleavable constructs [^68^Ga]­Ga-**21** (Ser), [^68^Ga]­Ga-**23** (^NMe^Gly) and [^68^Ga]­Ga-**24** (^NMe^Ser).

**Figure 6:**
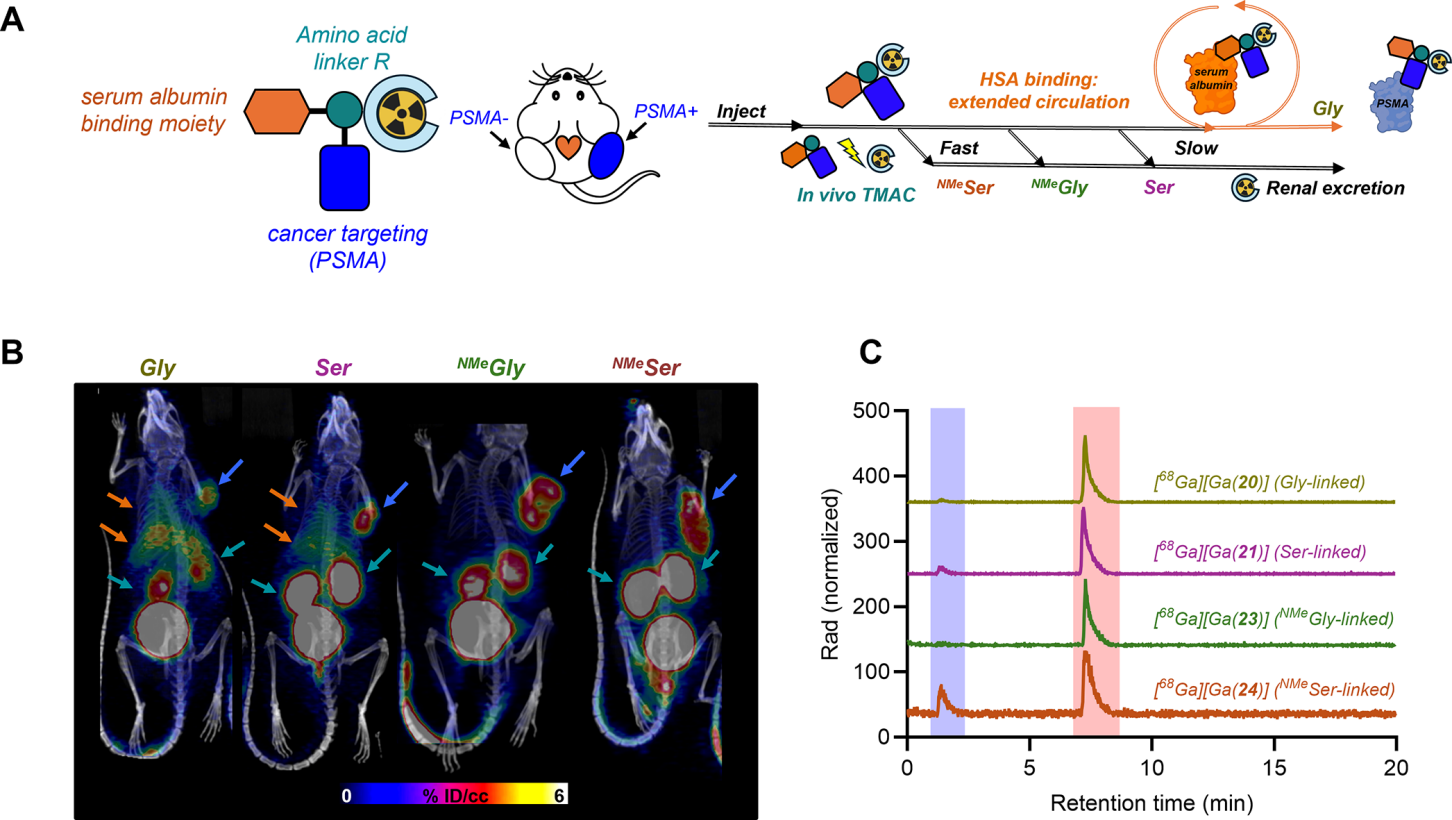
(A) proposed mechanism of action for self-cleaving PSMA-targeted conjugates labeled with ^68^Ga, with rapidly cleaving conjugates clearing faster renally, whereas slow/noncleaving conjugates are retained in circulation before the target binding event. (B) 30 min post injection PET imaging of Gly, Ser, ^NMe^Gly and ^NMe^Ser conjugates showing accelerated renal clearance for the latter conjugates, which also possess faster cleavage in vivo. Blue arrows indicate tumor, orange arrows indicate liver and heart uptake due to enahnced blood retention and green arrows indicate kidneys. (C) Urine metabolite analysis with intact conjugate (red box) and cleaved [^68^Ga]­Ga­(NOTA) (blue box).

However, we note that the in vitro cleavage rate is not fully predictive of the observed fraction of cleaved product in the urine metabolite and cannot reliably predict relative tumor uptake (Table 1). A confounding factor is the recognition of the peptide sequence by endogenous proteases during the metabolic processing of the tracer in vivo and subject of future investigation in our laboratory.

## Conclusions

In this work, we have uncovered a tunable, metal-mediated amide bond cleavage strategy (TMAC). Reactivity scope and mechanistic studies using spectroscopic tools and crystallography provide insight into the role of coordinative isomerism in controlling reaction rate at physiologically relevant pH. Subsequently, we demonstrated the applicability of TMAC to tune the pharmacokinetics of a diagnostic radiopharmaceutical for prostate cancer. Our findings indicate that TMAC provides unprecedented tunability for the self-immolative degradation of pharmaceuticals.

Prospective work pertains to the expansion of TMAC to other transition metal ions, lanthanides and metalloids. While other metals such as Fe^3+^ and In^3+^ display comparable cleavage to the investigated Ga^3+^ complexes, additional mechanisms may be implicated and provide logical opportunities to further expand the tunable nature of the TMAC strategy. From a practical, clinically relevant standpoint, implementation of a therapeutic radioisotope-compatible TMAC platform could mitigate prolonged radiation exposure risks, eventually enabling improved patient care and outcomes.

## Supplementary Material



## Notes

The Supporting Information including detailed synthesis procedures, characterization data, cleavage experiments, mechanism studies, images, and related biodistribution analysis are provided within the Supporting Information (PDF)
