# Supplementary material for: Rate-Tunable, Metal-Mediated Amide Bond Cleavage for the Controlled Release of Pharmaceuticals
Source: J Am Chem Soc. 2025 Nov 7;147(46):42491–9. doi: 10.1021/jacs.5c13166 (PMC12636028; doi:10.1021/jacs.5c13166)
Supplement: Supplementary file 1 [file ja5c13166_si_001.pdf]

Supporting Information for: Rate-Tunable, Metal-Mediated Amide Bond Cleavage for the Controlled Release of Pharmaceuticals

Zhuoran Zhong, Dariusz Śmiłowicz, Mallory J. Gork, Leah C. Garman, Ilia A. Guzei and Eszter Boros*.

1 Table of Contents

Abbreviations 3
1 Experimental procedures 4
1.1 Materials 4
1.2 General Methods for Characterization 4
2 Synthesis and Characterizations of Peptides 6
2.1 Synthesis of Model Tripeptides 6
2.2 Synthesis of Ahx-KuE Peptides 7
2.3 Synthesis of Model Ligands for Crystallographic Studies 8
2.4 Characterization Data of Peptide Intermediates and Final products 9
2.5 NMR Spectra of Peptides 36
2.6 Cleavage Assay for Ligands 79
3 Complex synthesis and characterization 84
3.1 General Complexation Protocol and Cleavage Assay Procedure 84
3.2 Complexation Protocol for Model Ligands 84
3.3 HRMS, HR-ESI-MS and HPLC Data for Coordination Complexes 85
3.4 HPLC-Monitored Complex Cleavage Experiments 108
4 Radiochemical Synthesis and Characterization 148
4.1 Radiolabeling with ^{68}Ga and Cleavage Assay in HEPES buffer 148
4.2 Radiolabeling with ^{67}Ga and Cleavage Assay in HEPES buffer 148
4.3 Radiolabeling with ^{67}Ga and Cleavage Assay in Mouse Plasma 148
4.4 Characterization of ^{68}Ga-radiolabeled complexes 149
4.5 Cleavage Assay of ^{68}Ga-Radiolabeled Complexes. 163
4.6 Cleavage Assay of ^{67}Ga-Radiolabeled Complexes. 179
5 Mechanistic Studies 203
5.1 Metal Complex Speciation 203
5.2 H_2O and $H_2{}^{18}O$ Cleavage Experiments 207
5.3 Cleavage assays in H_2O and D_2O for kinetic isotopic effect measurement 214
6 In vitro and In Vivo Experiments 218
6.1 HSA Binding Assay 218

6.2 In Vivo Biodistribution and Pharmacokinetics in NU/J Mice 219
6.3 Tumor Xenograft Model 219
6.4 ^{68}Ga Tracer Preparation Procedure 219
6.5 In Vivo PET-CT Imaging and Ex Vivo Biodistribution Results 220
6.6 Metabolite Analysis 222
6.7 Tabulated Biodistribution Data 225
7 X-ray Diffraction Analysis 227
7.1 X-ray Diffraction Analysis of [Ga(27)]ClO_4 227
7.1.1 Data Collection 227
7.1.2 Structure Solution and Refinement 227
7.1.3 Summary 228
7.1.4 Acknowledgement 228
7.2 X-ray Diffraction Analysis of [Ga(26)]ClO_4·H2O 237
7.2.1 Data Collection 237
7.2.2 Structure Solution and Refinement 237
7.2.3 Summary 238
7.2.4 Acknowledgement 238
7.3 X-ray Diffraction Analysis of Ga(26)·4H_2O 251
7.3.1 Data Collection 251
7.3.2 Structure Solution and Refinement 251
7.3.3 Summary 252
7.3.4 Acknowledgement 252
8 References 262

Abbreviations

AA Amino acid
Ac Acetate
Ahx Aminocaproic acid
Boc Tert-butyloxycarbonyl
Ci Curie
D_2O Deuterium oxide
DCM Dichloromethane
DIEA N,N-diisopropylethylamine
DMF Dimethylformamide
DMSO Dimethyl sulfoxide
DPBS Dulbecco's phosphate buffered saline
ESI-MS Electrospray ionization mass spectrometry
Et_2O Diethyl ether
EPPS 4-(2-Hydroxyethyl)-1-piperazinepropanesulfonic acid
FDA Food and Drug Administration
Fmoc fluorenylmethoxycarbonyl
HEPES 4-(2-hydroxyethyl)-1-piperazineethanesulfonic acid
HPLC High performance liquid chromatography
ICP-OES Inductively coupled plasma - optical emission spectrometry
MALDI Matrix assisted laser desorption/ionization
MBq Megabecquerels
MeCN Acetonitrile
MeOH Methanol
Mtt 4-Methyltrityl
MOPS 3-(N-morpholino)propanesulfonic acid
NaOAc Sodium acetate
NOTA 1,4,7-Triazacyclononane-1,4,7-triacetic acid
PBS Phosphate buffered saline
PIPES 2,2'-piperazine-1,4-diylbisethanesulfonic acid
PyBOP (Benzotriazol-1-yloxy)tripyrrolidinophosphonium
R_t Retention time
RA Rink amide resin
SPPS Solid-phase peptide synthesis
tBu Tert-butyl
TFA Trifluoroacetic acid
TIS Triisopropylsilane
WA Wang resin
Amino acids are abbreviated using their conventional 3-letter codes.

1 Experimental procedures

1.1 Materials

All starting materials were purchased from Acros Organics, Alfa Aesar, Millipore Sigma or TCI America and used without further purification. Fmoc-protected amino acids were purchased from Bachem. NOTA-bis (t-Bu ester) was obtained from Macrocyclics. Rink Amide resin and Wang resin resins were purchased from Millipore Sigma. $^{68}GaCl_3$ was obtained from an Eckert & Ziegler $^{68}Ge/^{68}Ga$ generator (Radiopharmaceutical Production Facility in University of Wisconsin Madison School of Medicine and Public Health, 20 mCi).

1.2 General Methods for Characterization

NMR spectra (^1H, $^{13}C\{^1H\}$, COSY, and HSQC) were collected at the University of Wisconsin-Madison Department of Chemistry Paul Bender Chemical Instrumentation Center (CIC) using a Bruker Avance III 500 with a DCH liquid He cryoprobe (Bender Fund), a Bruker Avance Neo 500 with a 5mm Prodigy-BBO liquid N_2 cryoprobe (NSF CHE-2017891), or a Bruker Avance III 600 with a TCI-F liquid He cryoprobe (NIH S10 OD012245). Data was processed using MestReNova 14.3.3-33362. Chemical shifts (δ) are reported in parts per million (ppm) relative to tetramethylsilane. ^1H, $^{13}C\{^1H\}$, COSY, and HSQC NMR spectra are referenced to residual solvent signals.

High resolution ESI mass spectrometry was carried out at the University of Wisconsin-Madison Department of Chemistry Paul Bender Chemical Instrumentation Center (CIC) using a Thermo Scientific Q Exactive Focus Orbitrap MS system, and at the University of Wisconsin-Madison School of Pharmacy Analytical Instrumentation Cluster using a Bruker MaXis Ultra-High Resolution Quadrupole Time-of-Flight MS system.

Ultraviolet-visible spectra were collected with the NanoDrop 1C instrument (AZY1706045). Spectra were recorded from 190 to 850 nm in a quartz cuvette with 1 cm path length. The absorbance of model ligands was measured in ammonium acetate buffer (10 mM pH 5.5).

ICP-OES and MP-AES analyses were carried out using an Agilent 5110 inductively coupled plasma optical emission spectrometer and an Agilent 4210 microwave plasma atomic emission spectrometer, respectively. For both techniques, a 6-point standard curve (1-100 ppm) with respect to gallium was used and fits were found to be $R^2 > 0.99$.

Analytical- and radio-HPLC methods were carried out using an Agilent 1260 Infinity II system. The instrument was set to detect UV absorption was recorded at 220 nm and 254 nm and were coupled to an in-line LabLogic Dual Scan-RAM detector and were controlled using the LabLogic Laura software package. RadioHPLC analyses utilized a LabLogic 1" NaI photomultiplier tube detector with 2" lead shielding and radioTLC analyses utilized a LabLogic plastic photomultiplier tube detector.

Semipreparative HPLC was carried out using a Shimadzu HPLC-20AR equipped with a binary gradient pump, UV-vis detector, and manual injector. UV absorption was recorded at 220 and 254 nm.

Flash chromatography was carried out using a Combi Flash Rf+ system with UV detection at 220 and 254 nm.

Liquid chromatography mass spectrometry (LCMS) was carried out on a Phenomenex Luna C18 column (5 µm, 150 mm × 3 mm, 100 Å, AXIA packed) at a flow rate of 0.8 mL/min using a single quadrupole Agilent 1200 Infinity II LC/MSD system equipped with a binary gradient pump, UV- vis detector, automatic injector, and an atmospheric pressure electrospray ionization (AP-ESI) source. Ultraviolet absorption was recorded at 220 nm and 254 nm, and positive and negative mass spectra were collected from m/z = 100-1000.

Chromatography Solvent Systems:

Analytical HPLC - Method A: binary solvent system (A: water + 0.1% FA; B: MeCN + 0.1% FA); gradient (0–3 min: 5% B; 3–8 min: 5-95% B; 8–11 min: 95% B; 11–13 min: 95–5% B; 13–16 min 5% B); flow rate: 0.8 mL/min; column: Phenomenex Luna C18 column (5 µm, 150 mm × 3 mm, 100 Å, AXIA packed).

Analytical HPLC – Method B: binary solvent system (A: water + 0.1% TFA; B: MeCN + 0.1% TFA); gradient (0–2 min: 5% B; 2–14 min: 5-95% B; 14–16 min: 95% B; 16–16.5 min: 95–5% B; 16.5–20 min 5% B); flow rate: 0.8 mL/min; column: Phenomenex Luna C18 column (5 µm, 150 mm × 3 mm, 100 Å, AXIA packed).

LCMS - Method C: binary solvent system (A: water + 0.1% FA; B: MeCN + 0.1% FA); gradient (0-3 min: 5% B; 3-10 min: 5-95% B; 10-13 min: 95% B; 13-13.5 min: 95-5% B; 13.5-16 min: 5% B); flow rate: 0.8 mL/min; column: Phenomenex Luna C18 column (5 µm, 150 mm × 3 mm, 100 Å, AXIA packed).

Semipreparative HPLC – Method D: binary solvent system (A: water + 0.1% FA; B: MeCN + 0.1% FA); gradient: (0–3 min: 5% B; 3–17 min: 5–50% B; 17–21 min: 50–95% B; 21–25 min: 95% B; 25–27 min: 95–5% B; 27–30 min: 5% B); flow rate: 10 mL/min; column: Phenomenex Luna C18 column (250 mm × 21.2 mm, 100 Å, AXIA packed).

Flash chromatography – Method E: Binary solvent system (A: water + 0.1% TFA; B: MeCN + 0.1% TFA); flow rate: 40 mL/min; column: RediSep C18 column (50 g HP C18 gold).

2 Synthesis and Characterizations of Peptides

2.1 Synthesis of Model Tripeptides

The synthesis of model tripeptides was carried out following a general procedure, typically on 0.13 mmol scale using a Rink amide (RA) resin with Boc-protected tryptophan (200 mg, 0.64 mmol/g). The RA resin was swollen in DCM (2 mL) and DMF (2 mL) for 1 min three times each. Fmoc-Gly-OH (152 mg, 0.51 mmol) was loaded onto the resin using Benzotriazole-l-yl-oxy-tris-pyrrolidinophosphonium hexafluorophosphate (PyBOP) (133 mg, 0.26 mmol) as coupling reagent in the presence of N, N-Diisopropylethylamine (DIEA) (89 μL, 0.51 mmol) within 12h. The Fmoc group was subsequently removed by treatment of the resin with 20% piperidine in DMF (2 mL) for 30 min. Subsequent amino acids were coupled in the same manner until the full sequences were assembled. Finally, the Fmoc group on N-terminus was deprotected and NOTA-bis (t-Bu ester) (106 mg, 0.26 mmol) was coupled using PyBOP (100 mg, 0.19 mmol) as coupling reagent in the presence of DIEA (89 μL, 0.51 mmol) within 12 h. Eventually, the products were washed and dried. The final model tripeptides from the resins were released by treating with a mixture of TFA/TIS/H_2O (95%/2.5%/2.5%).

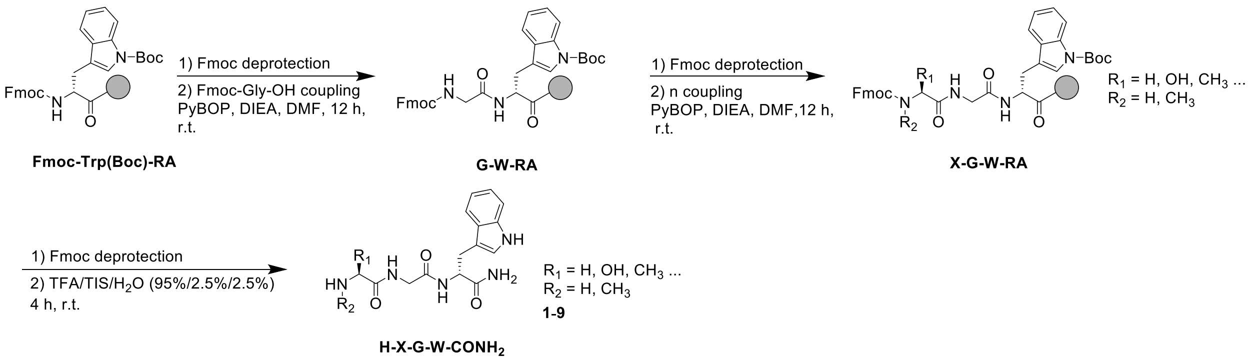

Scheme S1. Synthesis pathways for tripeptides.

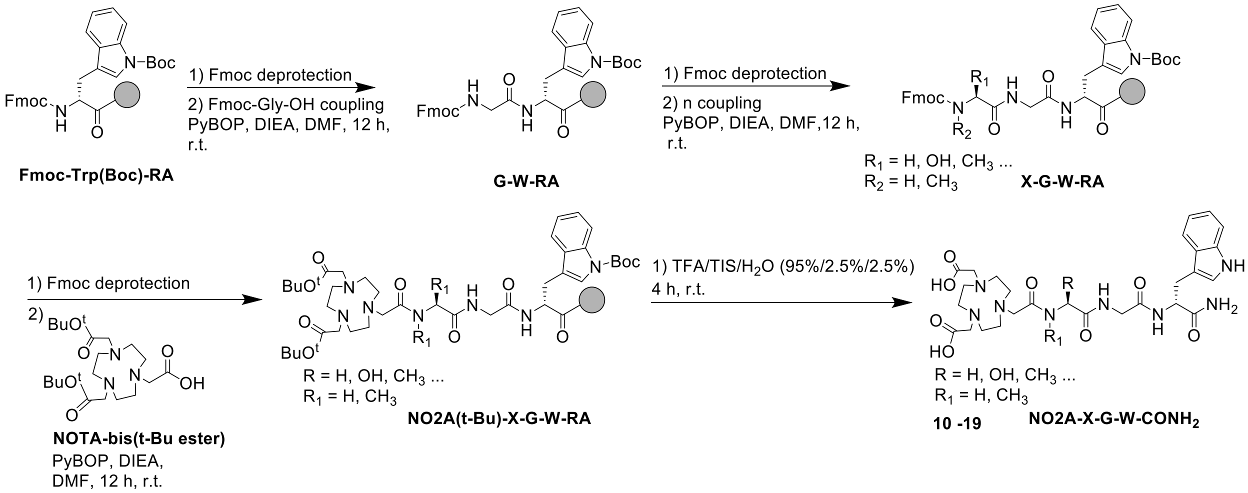

Scheme S2. Synthesis pathways for model tripeptides.

2.2 Synthesis of Ahx-KuE Peptides

The synthesis of the Ahx-KuE PSMA-targeting peptide (Glu-urea-Lys-NOTA) was carried out on a 0.16 mmol scale using a Wang resin (200 mg, 0.8 mmol/g). The starting material, Glu-urea-Lys, was synthesized according to our previously established protocol.[1] The Fmoc group from N-terminus was removed by shaking resin with 20% piperidine in DMF for 30 min. The Fmoc-Tyr(3-I)-OH was coupled to the peptide using PyBOP (167 mg, 0.32 mmol) as the coupling reagent in the presence of DIEA (111 µL, 0.64 mmol) within 12 h. After that, the peptide was elongated by coupling NOTA-bis (t-Bu) (133 mg, 0.32 mmol) using PyBOP (125 mg, 0.24 mmol) in the presence of DIEA (111 µL, 0.64 mmol) within 12 h. After coupling, resin was washed and dried. The final product was cleaved from the resin by treating it with a solution of TFA/TIS/H_2O (95%:2.5%:2.5%) for 6 h.

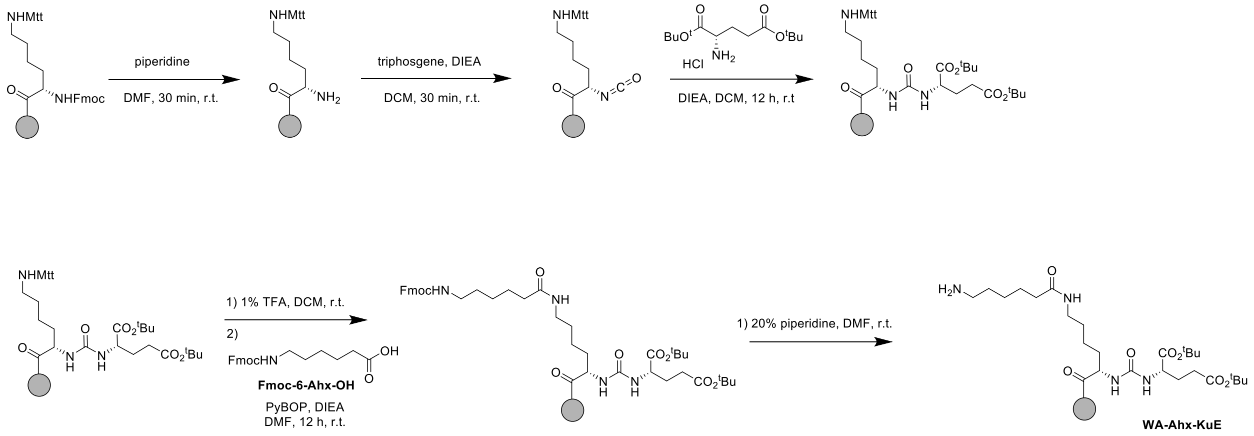

Scheme S3. Synthesis pathways for **WA-Ahx-KuE**.

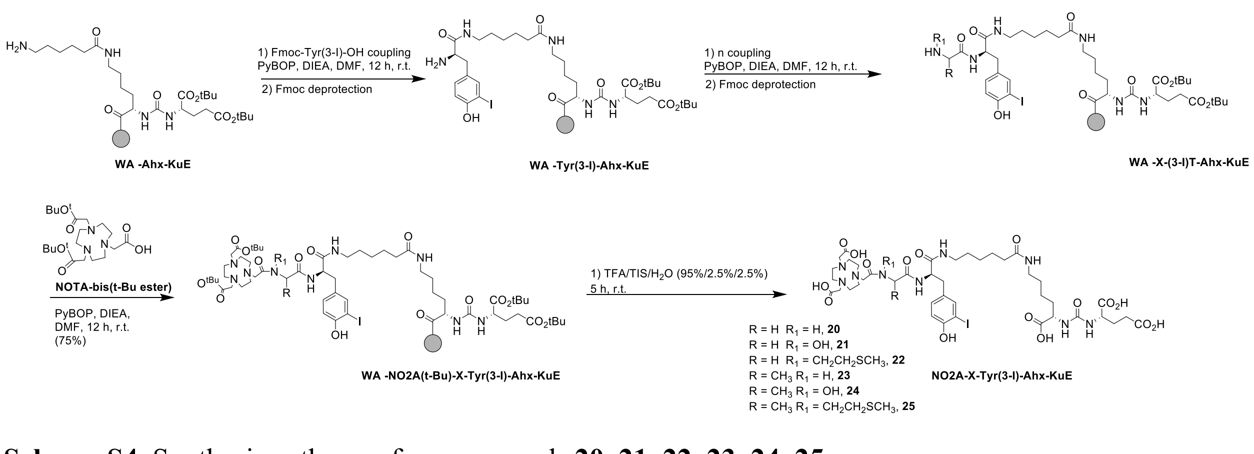

Scheme S4. Synthesis pathways for compounds **20**, **21**, **22**, **23**, **24**, **25**.

2.3 Synthesis of Model Ligands for Crystallographic Studies

The alkylation procedure for model ligand followed our previous established protocol.[2] To a solution of the secondary amine and potassium carbonate in dry acetonitrile at room temperature was added slowly the corresponding alkyl chloride or alkyl bromide as a solution in an equivalent volume of dry acetonitrile under N_2. The reaction mixture was stirred at room temperature for 1 hour and filtered. The solvent was removed under reduced pressure. The resulting crude material was redissolved in acetonitrile/water (3:7). The product was isolated using flash chromatography (Method E).

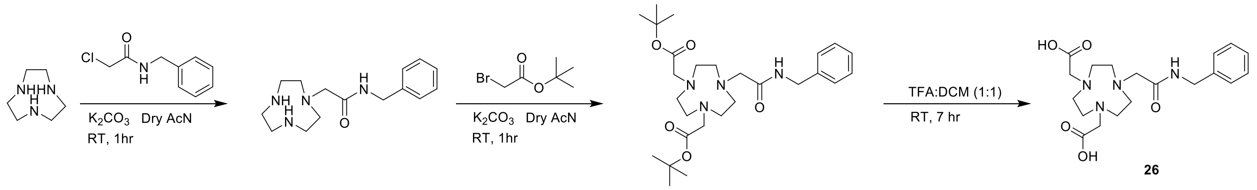

Scheme S5. Alkylation procedure for nonmethylated model ligand **26**.

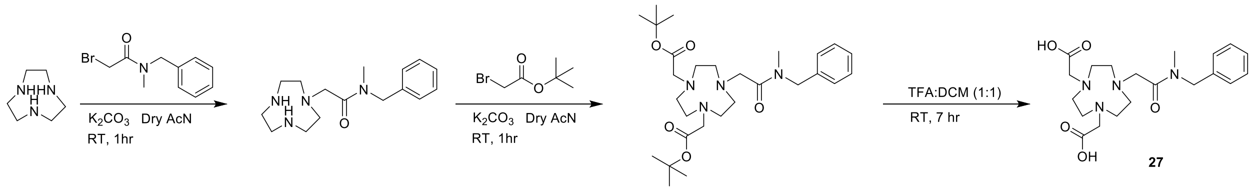

Scheme S6. Alkylation procedure for methylated model ligand **27**.

2.4 Characterization Data of Peptide Intermediates and Final products

(R)-2-(2-(2-aminoacetamido)acetamido)-3-(1H-indol-3-yl)propanamide, **H-G-G-W-CONH$_2$**. Compound **1** (200 mg,0.128 mmol) was synthesized using the general coupling strategy outlined in **Section 2.1** and **Scheme S1** from compound **Fmoc-Trp(Boc)-RA**. The product was isolated as as white solid following deprotection and purification using semi-preparative HPLC (Method D). Yield: (0.036 mg, 0.11 mmol, 87%). Rt (Method A): 5.97 min. HR-ESI-MS $[M+H]^+$ calc. for C15H19N5O3 317.15, found 318.1560. 1H NMR (500 MHz, D_2O): δ 7.71 (d, 1H), 7.54 (d, 1H), 7.29 (t, 2H), 7.21 (t, 1H), 4.70 (t, 1H), 3.94 (d, 2H), 3.79 (s, 2H), 3.36-3.24 (m, 2H). 13C NMR (126 MHz, D_2O): δ 176.14 (C10), 170.83 (C14), 167.61 (C12), 136.10 (C1), 126.90 (C6), 124.54 (C7), 121.94 (C3), 119.35 (C4), 118.34 (C5), 111.89 (C2), 108.93 (C8), 54.17 (C11), 42.17 (C13), 40.29 (C15), 27.00 (C9). HR-ESI-MS $[M+H]^+$ calc. for $C_{15}H_{19}N_5O_3$ 318.1561, found 318.1560.

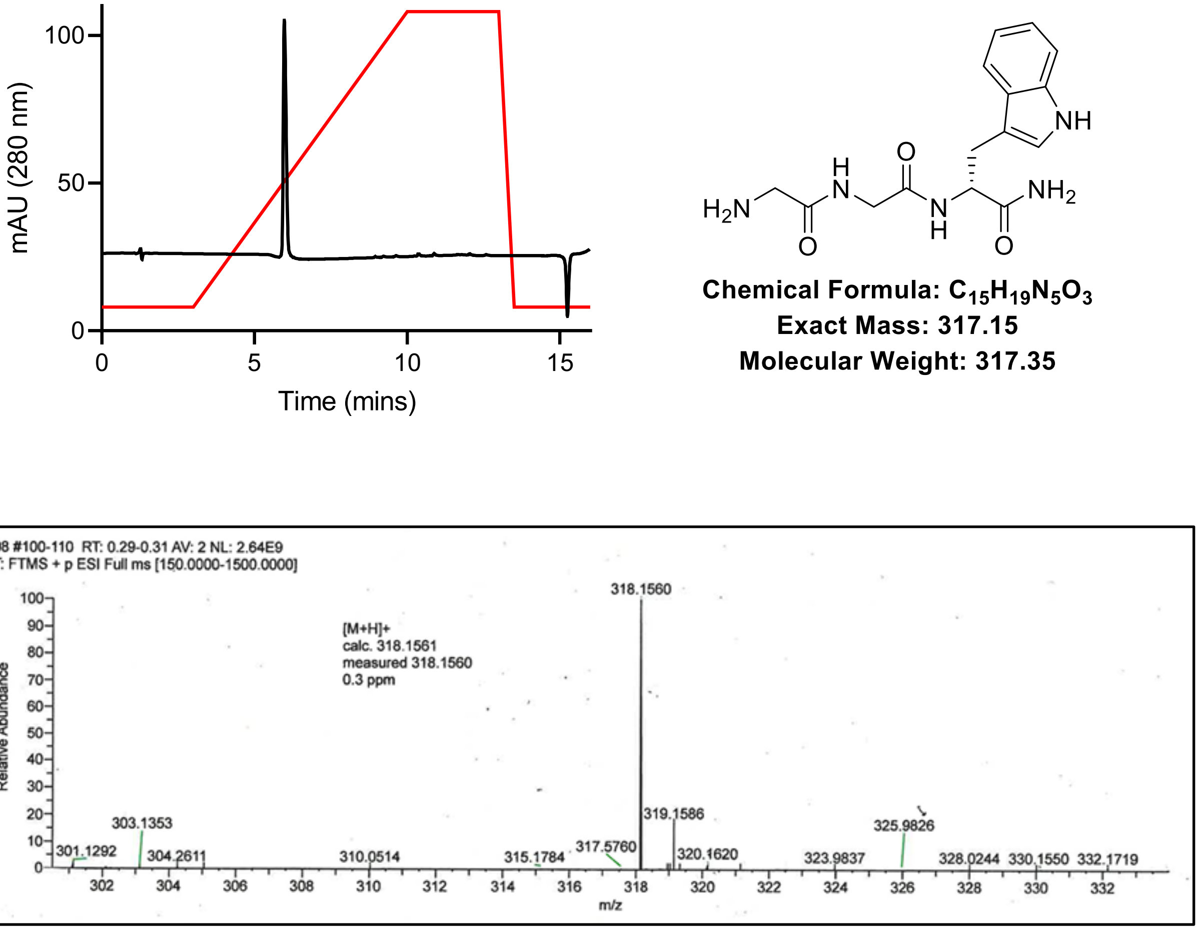

Figure S1. Characterization data for product **1**.

(R)-3-amino-N-(2-((1-amino-3-(1H-indol-3-yl)-1-oxopropan-2-yl)amino)-2-oxoethyl)propanamide, **H-β-Ala-G-W-CONH₂**. Compound **2** (200 mg,0.128 mmol) was synthesized using the general coupling strategy outlined in **Section 2.1 and Scheme S1** from compound **Fmoc-Trp(Boc)-RA**. The product was isolated as a white solid following deprotection and purification using Semi-Preparative HPLC (Method D). Yield: (0.036 mg, 0.11 mmol, 87%). Rt (Method A): 5.97 min. ^{1}H NMR (500 MHz, D_2O): δ 7.71 (d, 1H), 7.54 (d, 1H), 7.29 (t, 2H), 7.21 (t, 1H), 4.70 (t, 1H), 3.94 (d, 2H), 3.79 (s, 2H), 3.36-3.24 (m,2H). 13C NMR (126 MHz, D2O): δ 176.14 (C10), 170.83 (C14), 167.61 (C12), 136.10 (C1), 126.90 (C6), 124.54 (C7), 121.94 (C3), 119.35 (C4), 118.34 (C5), 111.89 (C2), 108.93 (C8), 54.17 (C11), 42.17 (C13), 40.29 (C15), 27.00 (C9). HR-ESI-MS $[M+H]^+$ calc. for $C_{15}H_{19}N_5O_3$ 332.1717, found 332.1714.

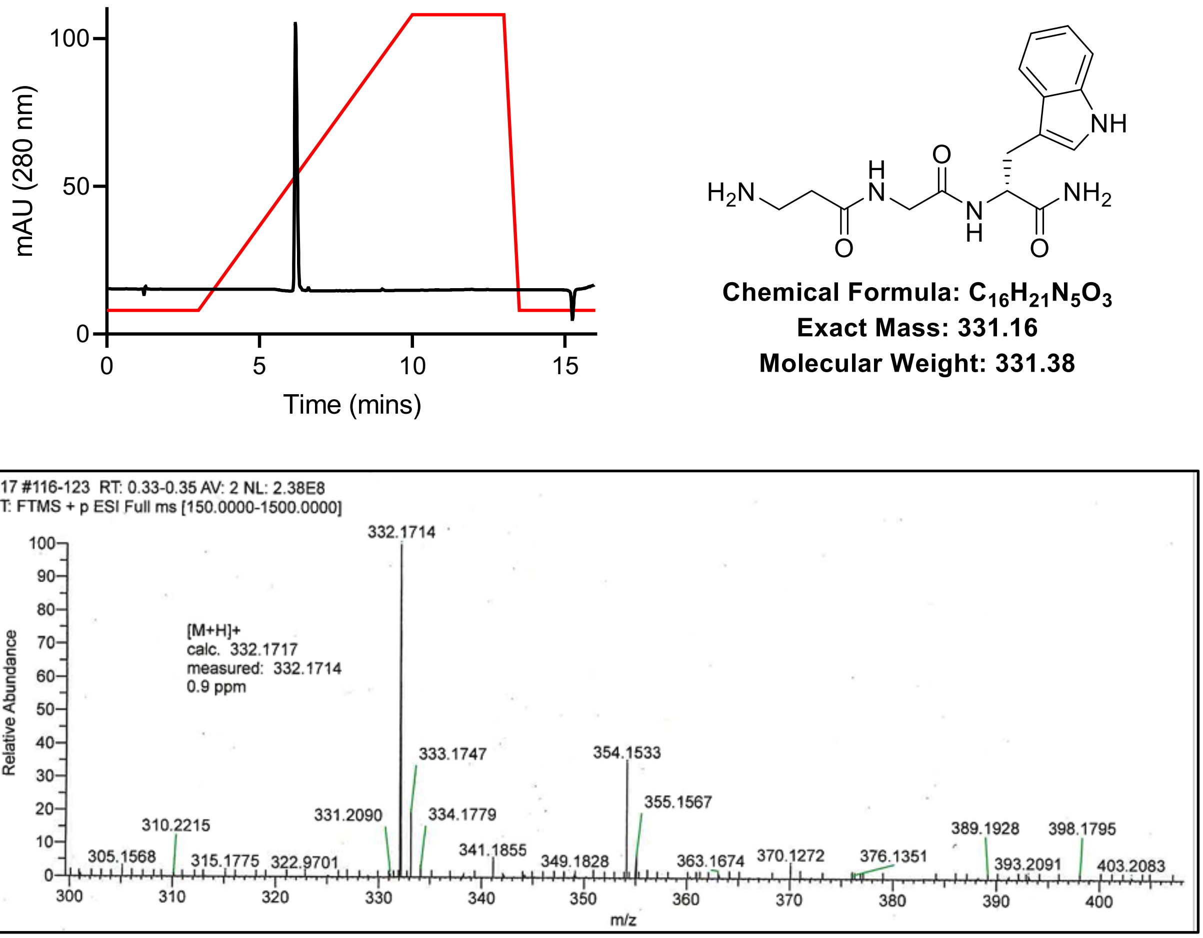

Figure S2 Characterization data for product **2**.

2-amino-N-(2-(((R)-1-amino-3-(1H-indol-3-yl)-1-oxopropan-2-yl)amino)-2-oxoethyl)propenamide, **H-A-G-W-CONH₂**. Compound **3** (200 mg,0.128 mmol) was synthesized using the general coupling strategy outlined in **Section 2.1** and **Scheme S1** from compound **Fmoc-Trp(Boc)-RA.** The product was isolated as a white solid following deprotection and purification using Semi-Preparative HPLC (Method D). Yield: (0.036 mg, 0.11 mmol, 80%). R_t (Method A): 6.12 min ^{1}H NMR (500 MHz, D_2O) δ 7.71 (d, 1H), 7.54 (d, 1H), 7.31 – 7.24 (m, 2H), 7.21 (t, 1H), 4.75 – 4.62 (m, 1H), 4.05 – 3.77 (m, 3H), 3.46 – 3.16 (m, 2H), 1.45 (d, 3H). ^{13}C NMR (126 MHz, D_2O) δ 176.12 (C^{10}), 172.23 (C^{14}), 170.74 (C^{12}), 136.11 (C^1), 126.89 (C^6), 124.53 (C^7), 121.95 (C^3), 119.34 (C^4), 118.35 (C^5), 111.89 (C^2), 108.88 (C^8), 54.10 (C^{11}), 49.06 (C^{13}), 42.20 (C^{15}), 27.04 (C^9), 16.63 (C^{16}).HR-ESI-MS $[M+H]^+$ calc. for $C_{16}H_{21}N_5O_3$ 332.1717, found 332.1711.

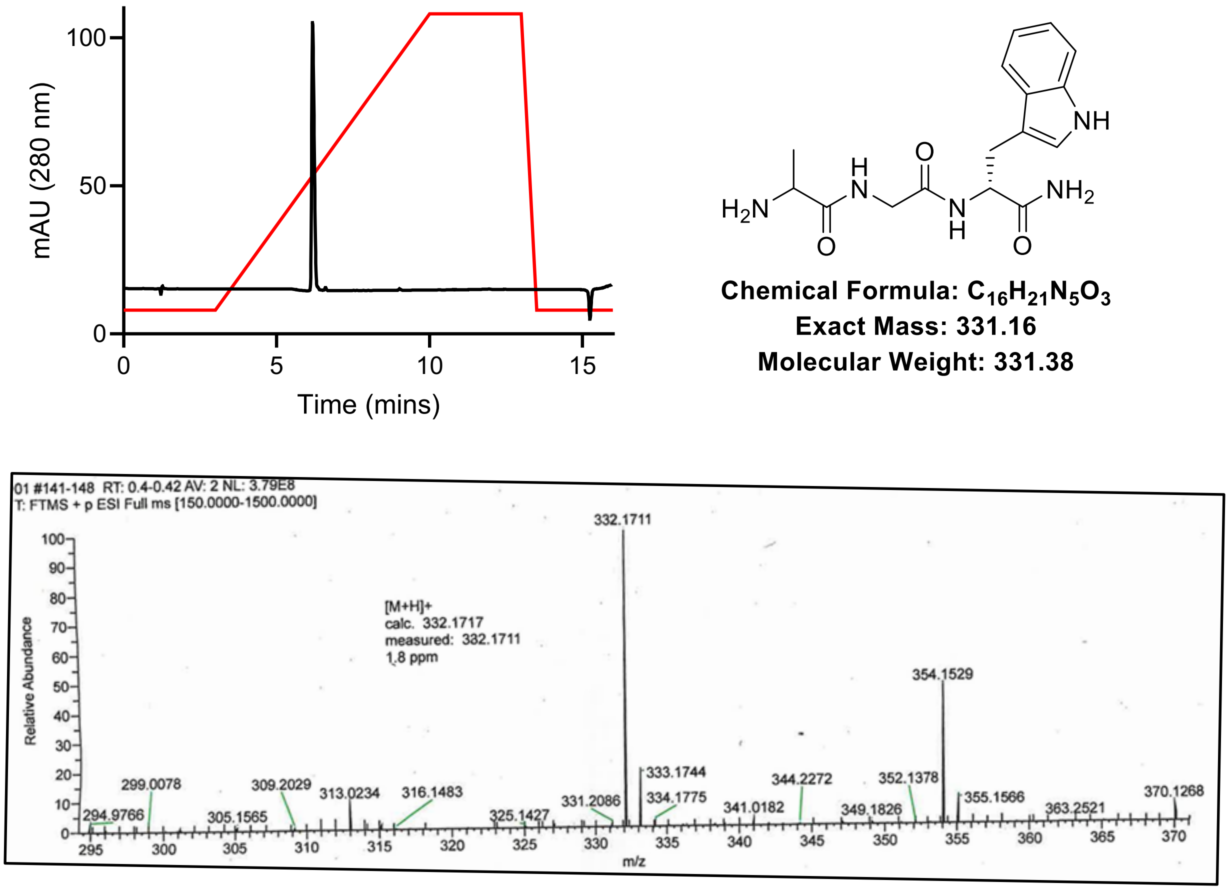

Figure S3. Characterization data for product **3**.

(S)-2-amino-N-(2-(((R)-1-amino-3-(1H-indol-3-yl)-1-oxopropan-2-yl)amino)-2-oxoethyl)-3-hydroxypropanamide, **H-S-G-W-CONH$_2$**. Compound **4** (200 mg,0.128 mmol) was synthesized using the general coupling strategy outlined in **Section 2.1 and Scheme S1** from compound **Fmoc-Trp(Boc)-RA**. The product was isolated as a white solid following the deprotection and purification using semi-preparative HPLC (Method D). Yield: (17 mg, 0.048 mmol, 38%). R_t (Method A): 5.89 min. ^{1}H NMR (500 MHz, D_2O): δ 7.72 (d, 1H), 7.55 (d, 1H), 7.33 – 7.26 (m, 2H), 7.21 (t,1H), 4.71 (t, 1H), 4.13 (t, 1H), 4.03 – 3.84 (m, 4H), 3.43 – 3.20 (m, 2H). ^{13}C NMR (126 MHz, D_2O): δ 176.13 (C^{10}), 170.69 (C^{14}), 168.64 (C^{12}), 136.12 (C^1), 126.88 (C^6), 124.54 (C^7), 121.95 (C^3), 119.35 (C^4), 118.34 (C^5), 111.89 (C^2), 108.93 (C^8), 60.16 (C^{16}), 54.57 (C^{11}), 54.16 (C^{15}), 42.28 (C^{13}), 27.05 (C^9). HR-ESI-MS $[M+H]^+$ calc. for $C_{16}H_{21}N_5O_4$ 348.1666, found 348.1662.

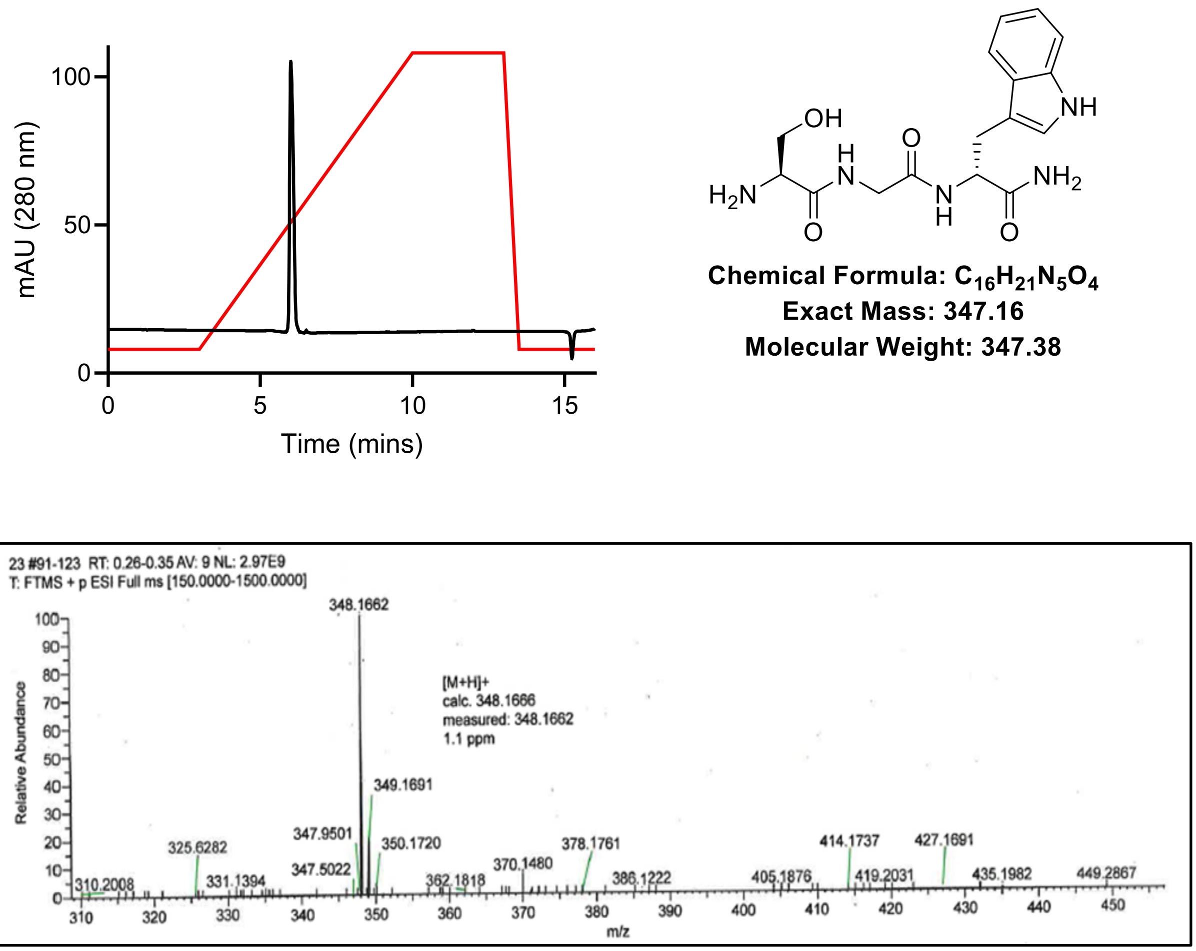

Figure S4. Characterization data for product **4**.

(2S)-2-amino-N-(2-(((S)-1-amino-3-(1H-indol-3-yl)-1-oxopropan-2-yl)amino)-2-oxoethyl)-3-hydroxybutanamide, **H-T-G-W-CONH$_2$**. Compound **5** (200 mg,0.128 mmol) was synthesized using the general coupling strategy outlined in **Section 2.1** and **Scheme S1** from compound **Fmoc-Trp(Boc)-RA**. The product was isolated as a white solid following the deprotection and purification using semi-preparative HPLC (Method D). Yield: (10 mg, 0.028 mmol, 22%). R$_t$ (Method A): 6.12 min. ^{1}H NMR (500 MHz, D$_2$O) δ 7.65 (d, 1H), 7.47 (d,1H), 7.26 – 7.20 (m, 2H), 7.14 (ddd, 1H), 4.64 (dd, 1H), 4.03 (p, 1H), 3.95 – 3.83 (m, 2H), 3.79 (d, 1H), 3.39 – 3.13 (m, 2H), 1.20 (d, 3H). ^{13}C NMR (126 MHz, D$_2$O) δ 176.12 (C^{10}), 170.54 (C^{14}), 168.77 (C^{12}), 136.14 (C^{1}), 126.89 (C^{6}), 124.55 (C^{7}), 121.96 (C^{3}), 119.36 (C^{4}), 118.36 (C^{5}), 111.90 (C^{2}), 108.96 (C^{8}), 66.16 (C^{16}), 58.66 (C^{15}), 54.17 (C^{11}), 42.25 (C^{13}), 27.11 (C^{9}), 18.53 (C^{17}). HR-ESI-MS [M+H]$^+$ calc. for $C_{18}H_{25}N_5O_3$ 362.1823, found 362.1822.

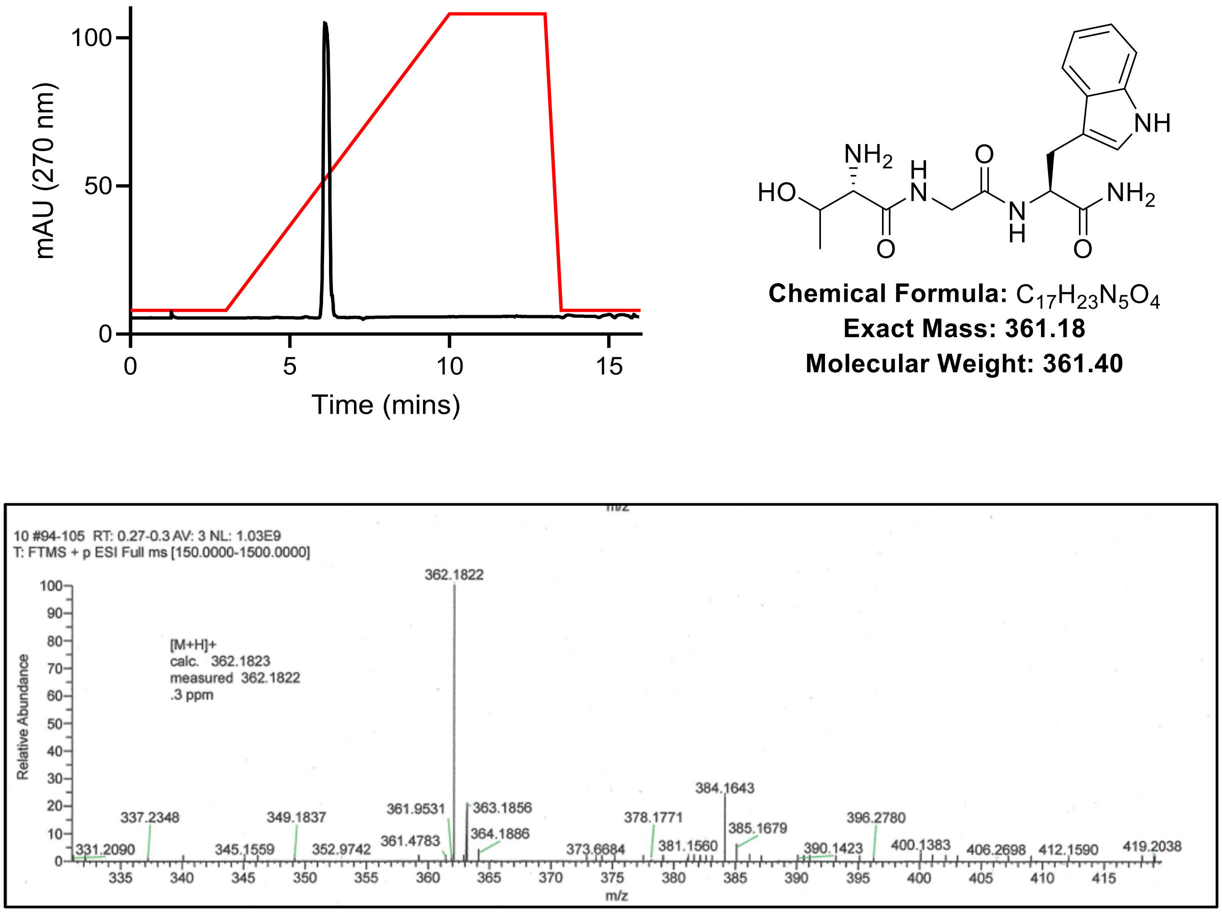

Figure S5. Characterization data for product **5**.

(S)-2-amino-N-(2-(((R)-1-amino-3-(1H-indol-3-yl)-1-oxopropan-2-yl)amino)-2-oxoethyl)-4-(methylthio)butanamide, **H-M-G-W-CONH₂**. Compound **6** (200 mg, 0.128 mmol) was synthesized using the general coupling strategy outlined in **Section 2.1** and **Scheme S1** from compound **Fmoc-Trp(Boc)-RA**. The product was isolated as a white solid following the deprotection and purification using semi-preparative HPLC (Method D). Yield (22 mg, 0.056 mmol, 44%). R_t (Method A): 6.67 min. ^{1}H NMR (500 MHz, D_2O): δ 7.63 (d, 1H), 7.46 (m, 2H), 7.24 – 7.17 (m, 1H), 7.12 (t, 1H), 4.62 (t, 1H), 4.02 (t, 1H), 3.87(s, 1H), 3.30-3.26 (m, 2H), 2.57 – 2.43 (m, 2H), 2.09 – 2.01 (m, 5H). ^{13}C NMR (126 MHz, D_2O): δ 176.08 (C^{10}), 170.40 (C^{14}), 170.13 (C^{12}), 136.11 (C^{1}), 126.88 (C^{6}), 124.53 (C^{7}), 121.94 (C^{3}), 119.34 (C^{4}), 118.35 (C^{5}), 111.90 (C^{2}), 108.95 (C^{8}), 54.13 (C^{11}), 52.27 (C^{18}), 42.22 (C^{13}), 29.87 (C^{17}), 28.15 (C^{16}), 27.14 (C^{9}), 13.93 (C^{15}). HR-ESI-MS $[M+H]^+$ calc. for $C_{18}H_{25}N_5O_3S$ 392.1751, found 392.1748.

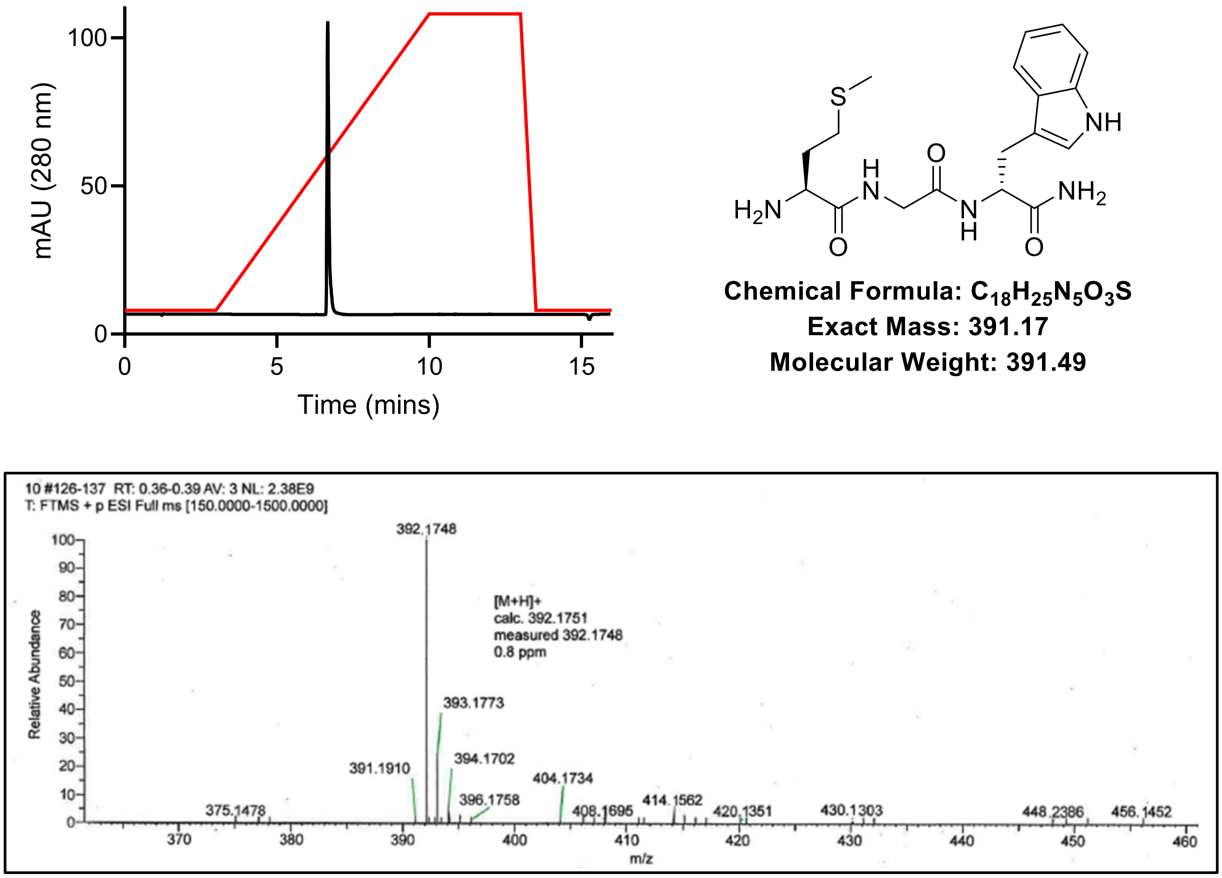

Figure S6. Characterization data for product **6.**

(S)-N-(2-(((R)-1-amino-3-(1H-indol-3-yl)-1-oxopropan-2-yl)amino)-2-oxoethyl)-3-hydroxy-2-(methylamino)propanamide, **H-$^{\mathbf{NMe}}$S-G-W-CONH$_2$**. Compound **7** (200 mg, 0.128 mmol) was synthesized using the general coupling strategy outlined in **Section 2.1** and **Scheme S1** from compound **Fmoc-Trp(Boc)-RA**. The product was isolated as a white solid following the deprotection and purification using semi-preparative HPLC (Method D). Yield (9 mg, 0.025 mmol, 20%). R_t (Method A): 5.96 min. ^{1}H NMR (500 MHz, D2O) δ 7.65 (d, 1H), 7.47 (d, 1H), 7.25 – 7.18 (m, 2H), 7.14 (ddd, 1H), 4.68 – 4.61 (m, 1H), 3.98 – 3.80 (m, 5H), 3.46 – 3.06 (m, 2H), 2.63 (s, 3H). ^{13}C NMR (126 MHz, D_2O) δ 176.13 (C^{10}), 170.56 (C^{14}), 167.75 (C^{12}), 136.14 (C^1), 126.89 (C^6), 124.49 (C^7), 121.97 (C^3), 119.36 (C^4), 118.36 (C^5), 111.89 (C^2), 108.96 (C^8), 62.37 (C^{16}), 59.12 (C^{15}), 54.13 (C^{11}), 42.35 (C^{13}), 31.43 (C^{17}), 27.09 (C^9). HR-ESI-MS $[M+H]^+$ calc. for $C_{17}H_{23}N_5O_4$ 362.1823, found 362.1817.

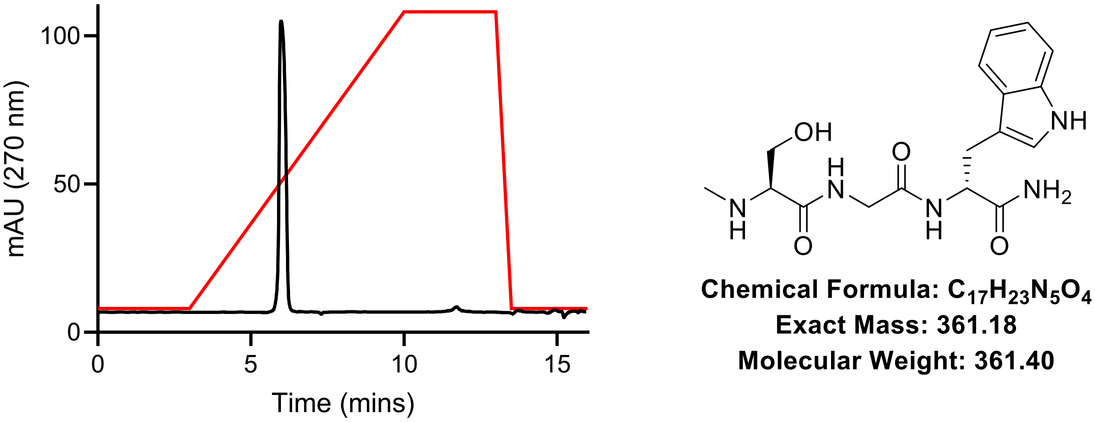

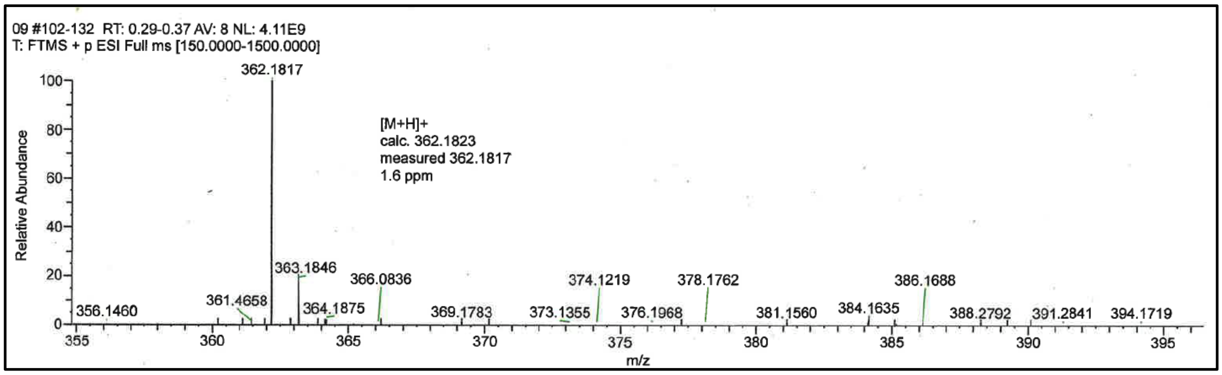

Figure S7. Characterization data for product **7.**

(R)-3-(1H-indol-3-yl)-2-(2-(2-(methylamino)acetamido)acetamido)propanamide, **H-^{NMe}G-G-W-CONH$_2$**. Compound **8** (200 mg, 0.128 mmol) was synthesized using the general coupling strategy outlined in **Section 2.1** and **Scheme S1** from compound **Fmoc-Trp(Boc)-RA**. The product was isolated as a white solid following the deprotection and purification using semi-preparative HPLC (Method D). Yield (22 mg, 0.056 mmol, 44%). R_t (Method A): 6.42 min. ^{1}H NMR (500 MHz, D_2O) δ 7.64 (d,1H), 7.46 (d,1H), 7.25 – 7.18 (m, 2H), 7.13 (t, 1H), 4.62 (t, 1H), 3.96 – 3.80 (m, 2H), 3.75 (s, 2H), 3.32 – 3.11 (m, 2H), 2.66 (s, 3H).^{13}C NMR (126 MHz, D_2O) δ 176.12 (C^{10}), 170.73 (C^{14}), 166.80 (C^{12}), 136.10 (C^1), 126.90 (C^6), 124.54 (C^7), 121.94 (C^3), 119.36 (C^4), 118.34 (C^5), 111.89 (C^2), 108.90 (C^8), 54.15 (C^{11}), 49.22 (C^{15}), 42.15 (C^{13}), 32.81 (C^{16}), 27.01 (C^9).HR-ESI-MS $[M+H]^+$ calc. for $C_{16}H_{21}N_5O_3$ 332.1717, found 332.1711.

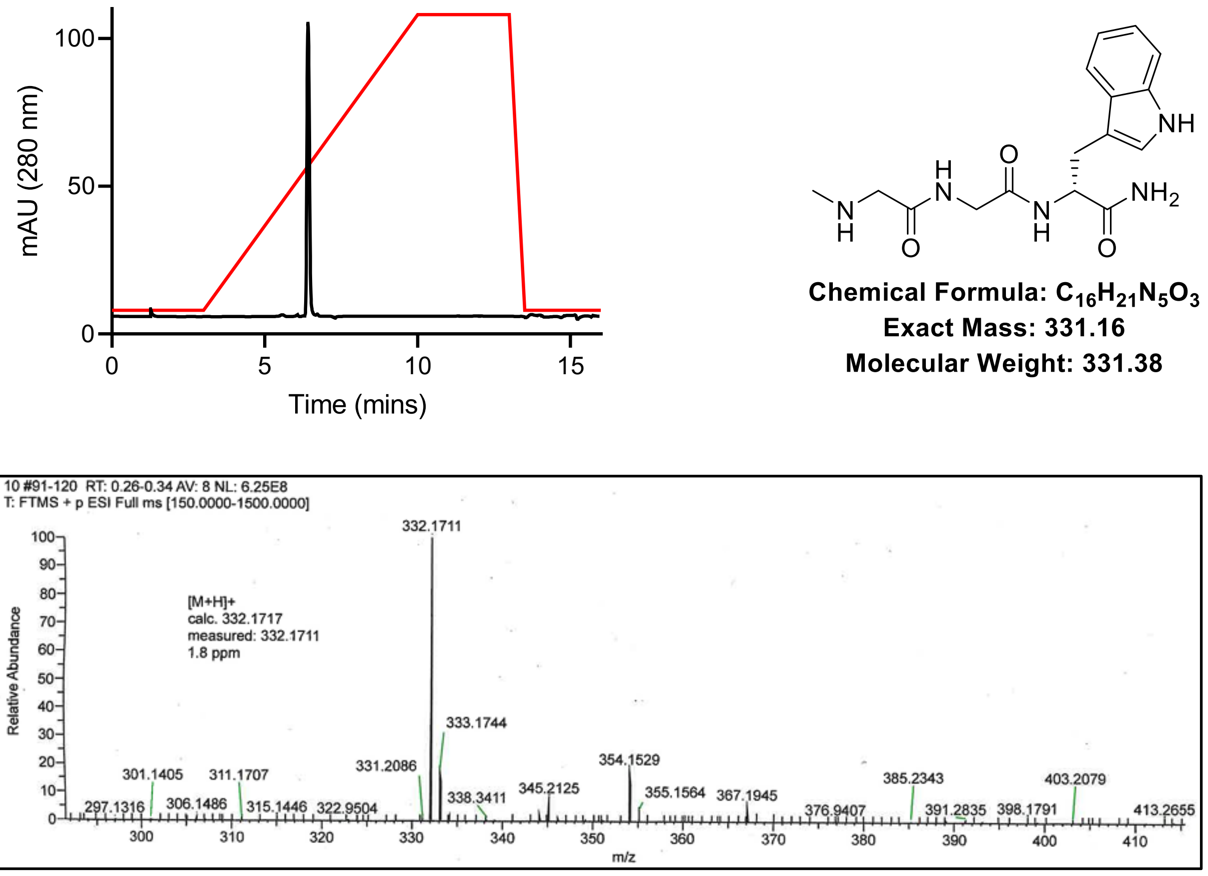

Figure S8. Characterization data for product **8.**

(S)-2-amino-N-(2-(((R)-1-amino-3-(1H-indol-3-yl)-1-oxopropan-2-yl) amino)-2-oxoethyl)-4-(methylthio)butanamide, **H-^{NMe}M-G-W-CONH$_2$**. Compound **9** (200 mg, 0.128 mmol) was synthesized using the general coupling strategy outlined in **Section 2.1** and **Scheme S1** from compound **Fmoc-Trp(Boc)-RA**. The product was isolated as a white solid following the deprotection and purification using semi-preparative HPLC (Method D). Yield (22 mg, 0.056 mmol, 44%). R_t (Method A): 6.22 min. ^{1}H NMR (500 MHz, D_2O): δ 7.73 (dt, 1H), 7.55 (dt, 1H), 7.33 – 7.29 (m, 2H), 7.22 (ddd, 1H), 4.73 (dd, 1H), 4.02 (dd, 1H), 3.99 (s, 2H), 3.52 – 3.18 (m, 2H), 2.67 (s, 3H), 2.63 – 2.46 (m, 2H), 2.20 - 2.14 (m, 2H), 2.09 (s, 3H). ^{13}C NMR (126 MHz, D_2O): δ 176.07 (C^{10}), 170.12 (C^{14}), 168.52 (C^{12}), 136.14 (C^1), 126.89 (C^6), 124.49 (C^7), 121.95 (C^3), 119.35 (C^4), 118.37 (C^5), 111.90 (C^2), 109.01 (C^8), 60.52 (C^{11}), 54.13 (C^{15}), 42.30 (C^{13}), 31.52 (C^{19}), 28.89 (C^{17}), 27.95 (C^{16}), 27.21 C^9), 13.96 (C^{18}). HR-ESI-MS $[M+H]^+$ calc. for $C_{19}H_{27}N_5O_3S$ 406.1907, found 406.1904.

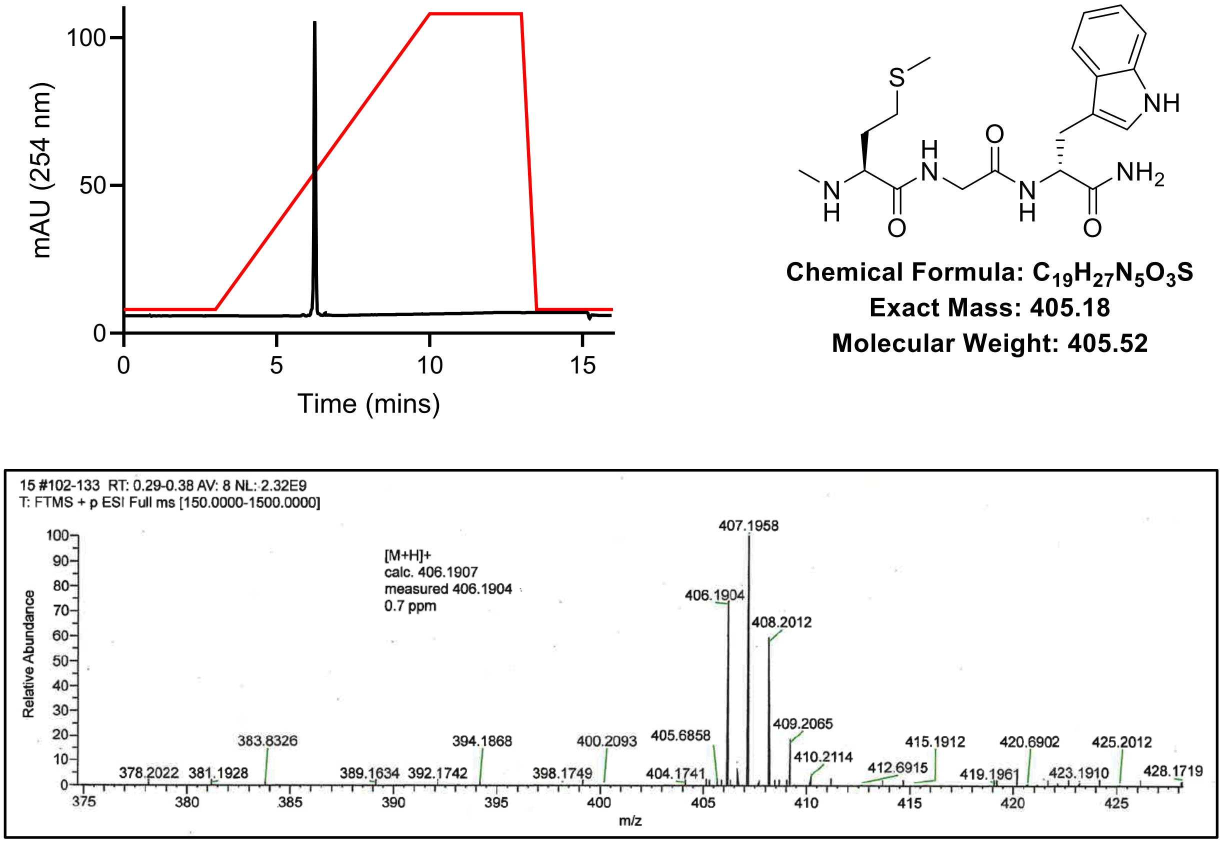

Figure S9. Characterization data for product **9.**

(R)-2,2'-(7-(2-((2-((2-((1-amino-3-(1H-indol-3-yl)-1-oxopropan-2-yl)amino)-2-oxoethyl)amino)-2-oxoethyl)amino)-2-oxoethyl)-1,4,7-triazonane-1,4-diyl)diacetic acid, **NOTA-G-G-W-CONH$_2$**. Compound **10** (157 mg, 0.100 mmol) was synthesized using the general coupling strategy outlined in **Section 2.1** and **Scheme S2** from compound **Fmoc-Trp(Boc)-RA**. The product was isolated as a white solid following the deprotection and purification using semi-preparative HPLC (Method D). Yield (24 mg, 0.039 mmol, 39%). R_t (Method A): 6.95 min. ^{1}H NMR (500 MHz, D_2O) δ 7.69 (d, 1H), 7.53 (d, 1H), 7.31 – 7.23 (m, 2H), 7.19 (t, 1H), 4.72 (t, 1H), 3.98 (d, 2H), 3.94 – 3.81 (m, 2H), 3.72 – 3.53 (m, 4H), 3.48 (d, 2H), 3.39 – 2.68 (m, 14H). ^{13}C NMR (126 MHz, D_2O): δ 176.43 ($C^{21,19}$), 174.40 (C^{10}), 171.86 (C^{12}), 171.49 ($C^{14,16}$), 136.12 (C^1), 126.90 (C^6), 123.99 (C^7), 122.03 (C^3), 119.39 (C^4), 118.39 (C^5), 111.88 (C^2), 109.25 (C^8), 59.02 (C^{17}), 56.83 (C^{18}), 53.85 (C^{20}), 50.63 ($C^{cycl.}$), 49.07 ($C^{cycl.}$), 48.26 ($C^{cycl.}$), 42.53 (C^{13}), 42.35 (C^{15}), 26.77 (C^{19}). HR-ESI-MS $[M+H]^+$ calc. for $C_{27}H_{38}N_8O_8$ 603.2885, found 603.2884.

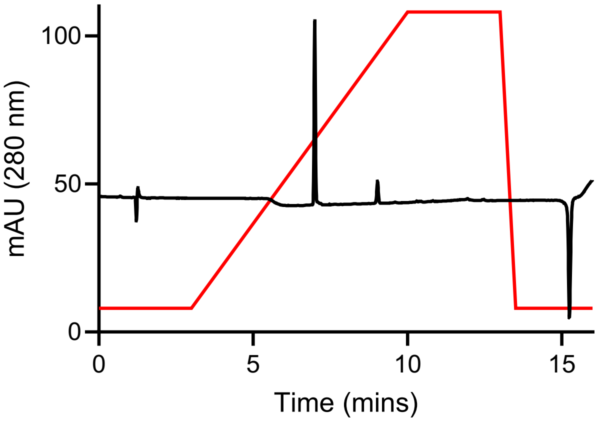

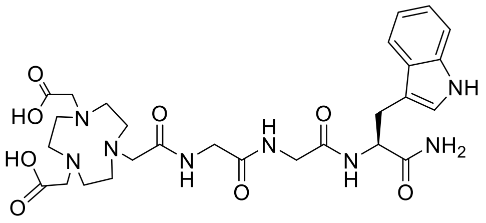

Chemical Formula: $C_{27}H_{38}N_8O_8$
Exact Mass: 602.28
Molecular Weight: 602.65

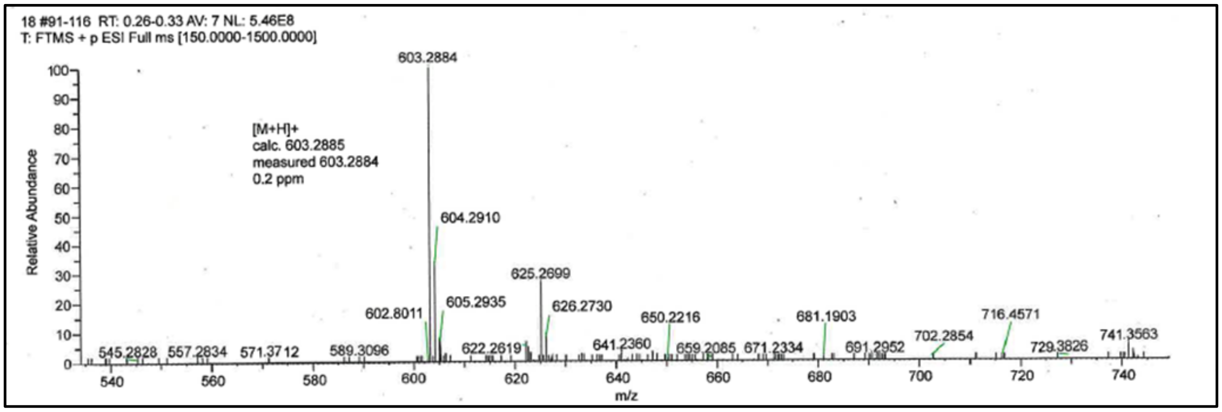

Figure S10. Characterization data of compound **10.**

(S)-2,2'-(7-(2-((3-((2-((1-amino-3-(1H-indol-3-yl)-1-oxopropan-2-yl)amino)-2-oxoethyl)amino)-3-oxopropyl)amino)-2-oxoethyl)-1,4,7-triazonane-1,4-diyl)diacetic acid, **NOTA-$^{\beta}$A-G-W-CONH$_2$**. Compound **11** (157 mg, 0.100 mmol) was synthesized using the general coupling strategy outlined in **Section 2.1** and **Scheme S2** from compound **Fmoc-Trp(Boc)-RA**. The product was isolated as a white solid following the deprotection and purification using Semi-preparative HPLC (Method D). Yield (24 mg, 0.039 mmol, 39%). R_t (Method A): 6.89 min. ^{1}H NMR (500 MHz, D_2O) δ 7.70 (d, 1H), 7.53 (d, 1H), 7.31 – 7.24 (m, 2H), 7.23 – 7.16 (m, 1H), 4.67 (dd, 2H), 3.90 – 3.78 (m, 2H), 3.72 (s, 4H), 3.37 (d, 5H), 3.26 (s, 5H), 3.09 (t, 4H), 2.81 (s, 4H), 2.42 (t, 2H). ^{13}C NMR (126 MHz, D_2O) δ 176.42 ($C^{20,22}$), 174.60 (C^{10}), 173.52 (C^{12}), 172.91 (C^{14}), 171.48 (C^{17}), 136.21 (C^1), 126.98 (C^6), 124.44 (C^7), 122.08 (C^3), 119.50 (C^4), 118.44 (C^5), 111.99 (C^2), 109.07 (C^8), 59.10 (C^{18}), 57.22 ($C^{19,21}$), 54.04 (C^{11}), 51.02 ($C^{cycl.}$), 49.37 ($C^{cycl.}$), 48.59 ($C^{cycl.}$), 42.68 (C^{13}), 35.87 (C^{16}), 35.00 (C^{15}), 26.91 (C^9). HR-ESI-MS $[M-H]^-$ calc. for $C_{28}H_{40}N_8O_8$ 615.2896, found 615.2903.

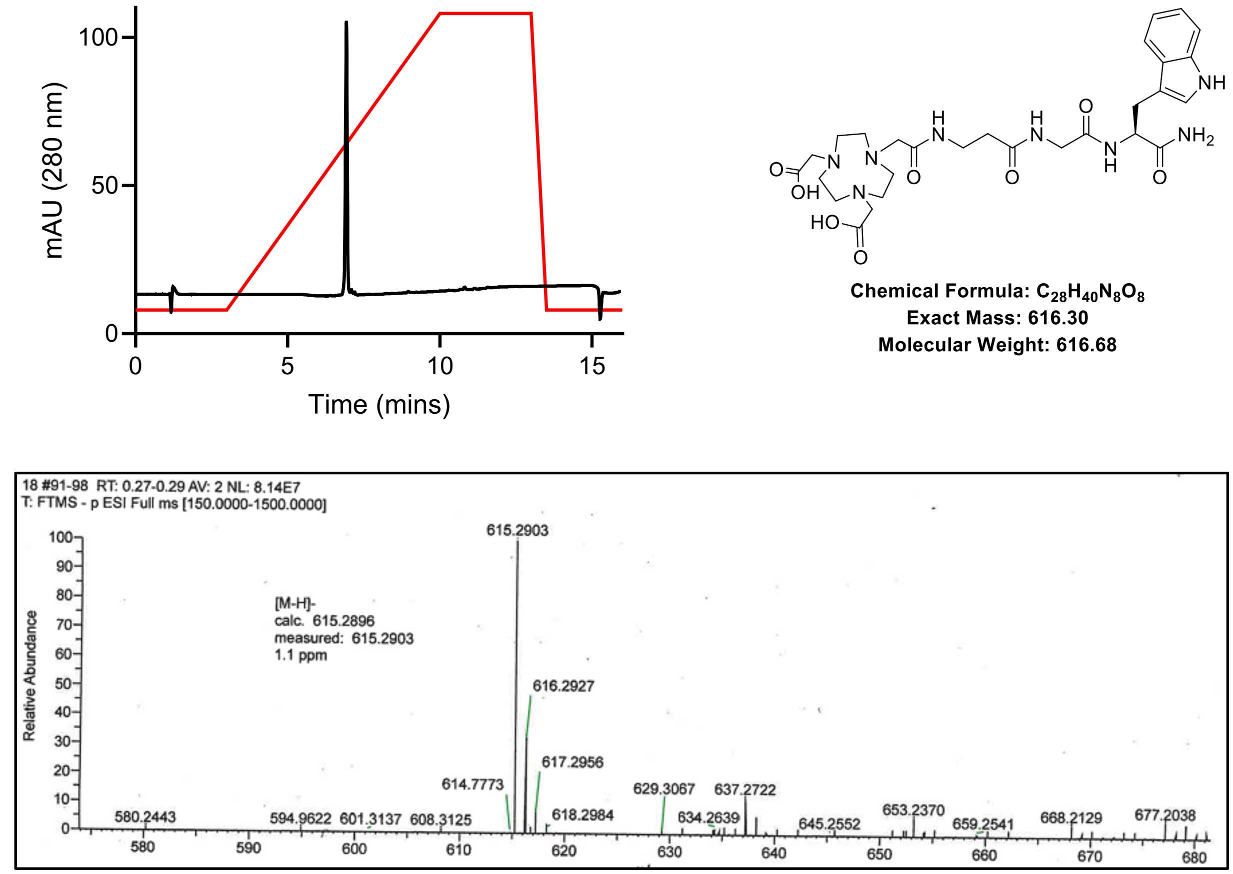

Figure S11. Characterization data of compound **11**.

2,2'-(7-(2-(((R)-1-((2-(((R)-1-amino-3-(1H-indol-3-yl)-1-oxopropan-2-yl)amino)-2-oxoethyl)amino)-1-oxopropan-2-yl)amino)-2-oxoethyl)-1,4,7-triazonane-1,4-diyl)diacetic acid, **NOTA-A-G-W-CONH₂**. Compound **12** (157 mg, 0.100 mmol) was synthesized using the general coupling strategy outlined in **Section 2.1** and **Scheme S2** from compound **Fmoc-Trp(Boc)-RA**. The product was isolated as a white solid following the deprotection and purification using semi-preparative HPLC (Method D). Yield (24 mg, 0.039 mmol, 39%). R_t (Method A): 6.34 min. ^{1}H NMR (500 MHz, D_2O) δ 7.68 (d, 1H), 7.52 (d, 1H), 7.27 (t, 1H), 7.24 – 7.16 (m, 2H), 4.70 (dd, 1H), 4.28 (q, 1H), 4.08 – 3.84 (m, 2H), 3.70 (s, 4H), 3.47 – 3.32 (m, 3H), 3.30 – 3.15 (m, 5H), 3.00 (s, 4H), 2.76 (s, 4H), 1.33 (d, 3H). ^{13}C NMR (126 MHz, D_2O) δ 176.25 (C^{20}), 175.64 (C^{22}), 173.56 (C^{10}), 172.52 ($C^{12,14}$), 171.42 (C^{17}), 136.08 (C^{1}), 126.96 (C^{6}), 123.89 (C^{7}), 122.04 (C^{3}), 119.39 (C^{4}), 118.41 (C^{5}), 111.88 (C^{2}), 109.29 (C^{8}), 58.15 (C^{18}), 57.08 ($C^{19,21}$), 53.89 (C^{101}), 50.82 ($C^{cycl.}$), 49.95 ($C^{cycl.}$), 49.27 ($C^{cycl.}$), 48.15 (C^{15}), 42.56 (C^{13}), 26.92 (C^{9}), 16.07 (C^{16}).HR-ESI-MS $[M-H]^-$ calc. for $C_{28}H_{40}N_8O_8$ 615.2896, found 615.2905.

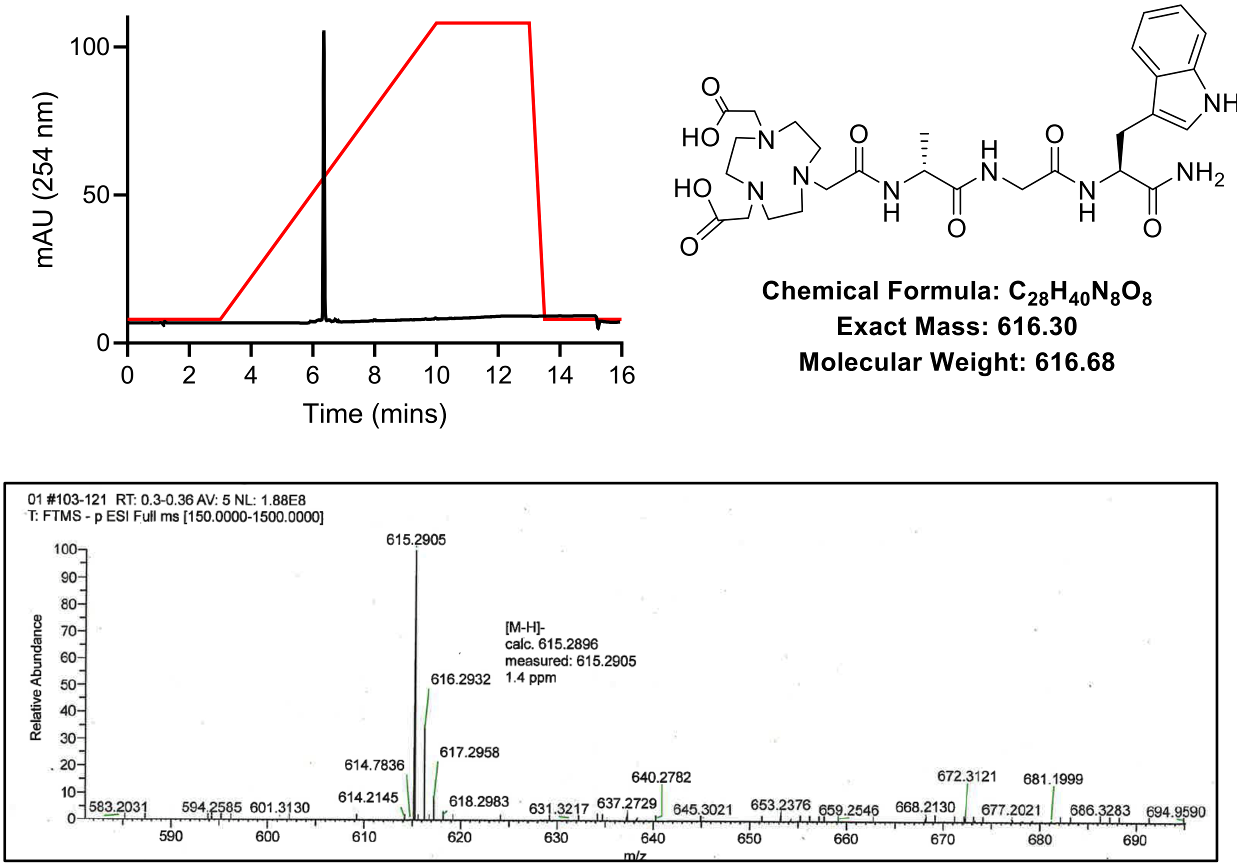

Figure S12. Characterization data of compound **12.**

2,2'-(7-(2-(((R)-1-((2-(((S)-1-amino-3-(1H-indol-3-yl)-1-oxopropan-2-yl)amino)-2-oxoethyl)amino)-3-hydroxy-1-oxopropan-2-yl)amino)-2-oxoethyl)-1,4,7-triazonane-1,4-diyl)diacetic acid, **NOTA-S-G-W-CONH$_2$**. Compound **13** was synthesized using the general coupling strategy outlined in **Section 2.1** and **Scheme S2** from compound **Fmoc-Trp(Boc)-RA**. The product was isolated as a white solid following purification using semi-preparative HPLC (Method D). Yield (26 mg, 0.041 mmol, 36%). R_t (Method A): 6.86 min. ^{1}H NMR (500 MHz, D_2O): δ 7.69 (d, 1H), 7.53 (d, 1H), 7.31 – 7.22 (m, 2H), 7.22 – 7.16 (m, 1H), 4.70 (dd, 1H), 4.44 (t, 1H), 3.99 – 3.87 (m, 2H), 3.86 – 3.71 (m, 6H), 3.59 – 3.45 (m, 2H), 3.36 (dd, 1H), 3.32-2.72 (m, 12H). ^{13}C NMR (126 MHz, D_2O): δ 176.19 (C^{20}), 173.84 (C^{22}), 172.50 (C^{10}), 172.23 (C^{14}), 171.24 ($C^{12,17}$), 136.09 (C^{1}), 126.91 (C^{6}), 124.07 (C^{7}), 122.01 (C^{3}), 119.38 (C^{4}), 118.41 (C^{5}), 111.87 (C^{2}), 109.23 (C^{8}), 60.66 (C^{18}), 58.55 (C^{16}), 56.94 (C^{15}), 56.00 ($C^{19,21}$), 53.94 (C^{11}), 50.91 ($C^{cycl.}$), 49.37 ($C^{cycl.}$), 48.45 ($C^{cycl.}$), 42.56 (C^{13}), 26.98 (C^{9}). HR-ESI-MS $[M-H]^-$ calc. for $C_{28}H_{40}N_8O_9$ 631.2845, found 631.2854.

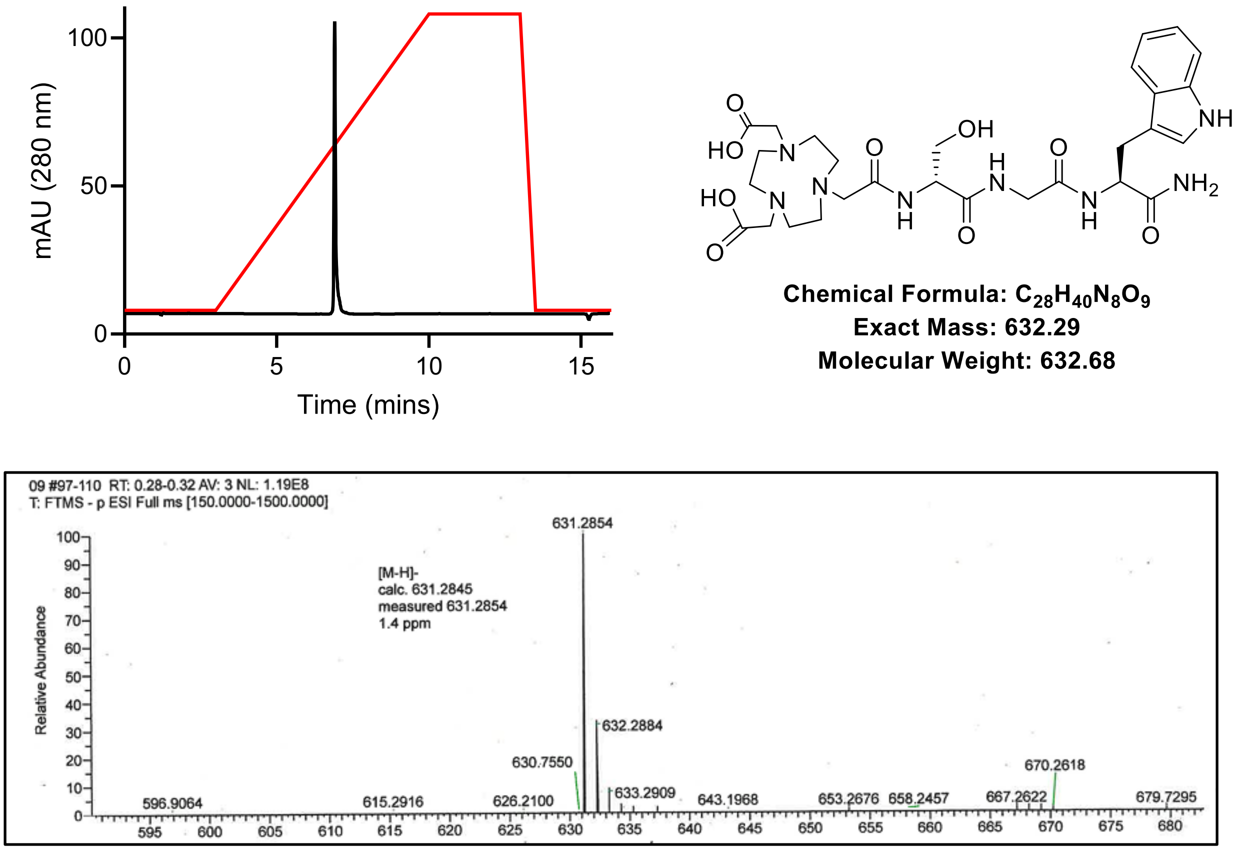

Figure S13. Characterization data for compound **13.**

2,2'-(7-(2-(((2R,3R)-1-((2-(((S)-1-amino-3-(1H-indol-3-yl)-1-oxopropan-2-yl)amino)-2-oxoethyl)amino)-3-hydroxy-1-oxobutan-2-yl)amino)-2-oxoethyl)-1,4,7-triazonane-1,4-diyl)diacetic acid, **NOTA-T-G-W-CONH$_2$**. Compound **14** was synthesized using the general coupling strategy outlined in **Section 2.1** and **Scheme S2** from compound **Fmoc-Trp(Boc)-RA**. The product was isolated as a white solid following purification using semi-preparative HPLC (Method D). Yield (17 mg, 0.026 mmol, 21%). R_t (Method A): 6.89 min. ^{1}H NMR (500 MHz, D_2O) δ 7.60 (d, 1H), 7.44 (d, 1H), 7.18 (ddd, 1H), 7.14 – 6.98 (m, 2H), 4.61 (dd, 1H), 4.16 (d, 1H), 4.01 (p, 1H), 3.96 – 3.76 (m, 2H), 3.65 (d, 4H), 3.52 – 3.33 (m, 2H), 3.31 – 2.39 (m, 13H), 1.11 (d, 3H). ^{13}C NMR (126 MHz, D_2O) δ 176.19 ($C^{21,23}$), 174.05 (C^{10}), 172.50 (C^{12}), 171.23 ($C^{14,18}$), 136.09 (C^1), 126.94 (C^6), 123.96 (C^7), 122.03 (C^3), 119.38 (C^4), 118.41 (C^5), 111.89 (C^2), 109.33 (C^8), 66.53 (C^{16}), 60.25 (C^{15}), 58.36 (C^{19}), 57.28 ($C^{20,22}$), 53.94 (C^{11}), 49.47 ($C^{cycl.}$), 48.41 ($C^{cycl.}$), 42.55 (C^{13}), 27.05 (C^9), 18.69 (C^{17}). HR-ESI-MS $[M+H]^+$ calc. for $C_{29}H_{42}N_8O_9$ 647.3147, found 647.3139.

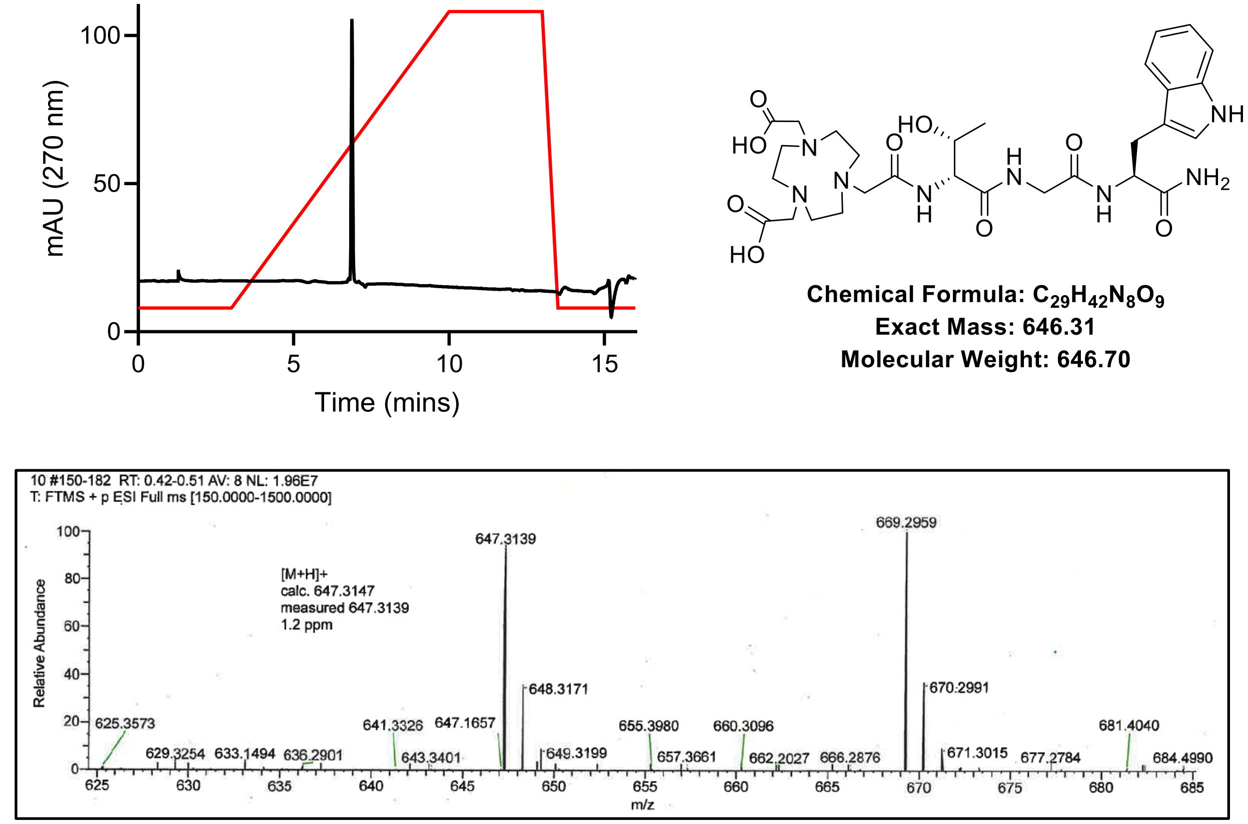

Figure S14. Characterization data for compound **14.**

2,2'-(7-(2-(((R)-1-((2-(((S)-1-amino-3-(1H-indol-3-yl)-1-oxopropan-2-yl)amino)-2-oxoethyl)amino)-4-(methylthio)-1-oxobutan-2-yl)amino)-2-oxoethyl)-1,4,7-triazonane-1,4-diyl)diacetic acid, **NOTA-M-G-W-CONH₂**. Compound **14** was synthesized using the general coupling strategy outlined in **Section 2.1** and **Scheme S2** from compound **Fmoc-Trp(Boc)-RA**. The product was isolated as a white solid following purification using semi-preparative HPLC (Method D). Yield (26 mg, 0.041 mmol, 36%). R_t (Method A): 7.20 min. ^{1}H NMR (500 MHz, D_2O) δ 7.69 (d, 1H), 7.53 (d, 1H), 7.32 – 7.22 (m, 2H), 7.20 (t, 2H), 4.71 (dd, 1H), 4.42 (dd, 1H), 3.99 – 3.85 (m, 2H), 3.72 (s, 5H), 3.50 – 3.40 (m, 2H), 3.40 – 3.16 (m, 7H), 3.14 – 2.69 (m, 9H), 2.62 – 2.48 (m, 2H), 2.10 (s, 3H), 2.07 – 1.91 (m, 2H). ^{13}C NMR (126 MHz, D_2O) δ 176.20 ($C^{22,24}$), 174.16 (C^{10}), 173.85 (C^{14}), 172.61 (C^{12}), 171.27 (C^{19}), 136.08 (C^{1}), 126.95 (C^{6}), 123.95 (C^{7}), 122.03 (C^{3}), 119.39 (C^{4}), 118.40 (C^{5}), 111.90 (C^{2}), 109.28 (C^{8}), 57.94 ($C^{21,23}$), 57.40 (C^{20}), 53.91 (C^{11}), 53.07 (C^{15}), 50.97 ($C^{cycl.}$), 49.43 ($C^{cycl.}$), 48.32 ($C^{cycl.}$), 42.62 (C^{13}), 29.70 (C^{16}), 29.40 (C^{17}), 26.97 (C^{9}), 14.12 (C^{18}). HR-ESI-MS $[M+H]^+$ calc. for $C_{30}H_{44}N_8O_{98}S$ 677.3076, found 677.3068.

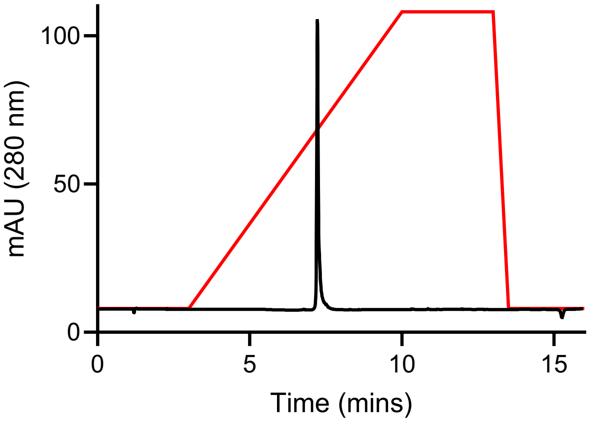

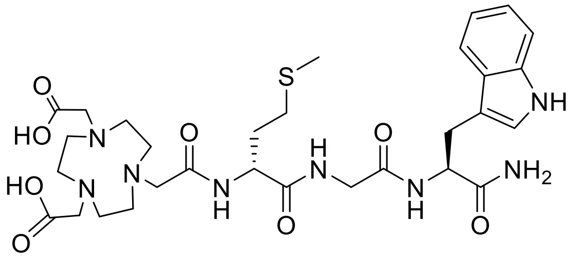

Chemical Formula: $C_{30}H_{44}N_8O_8S$
Exact Mass: 676.30
Molecular Weight: 676.79

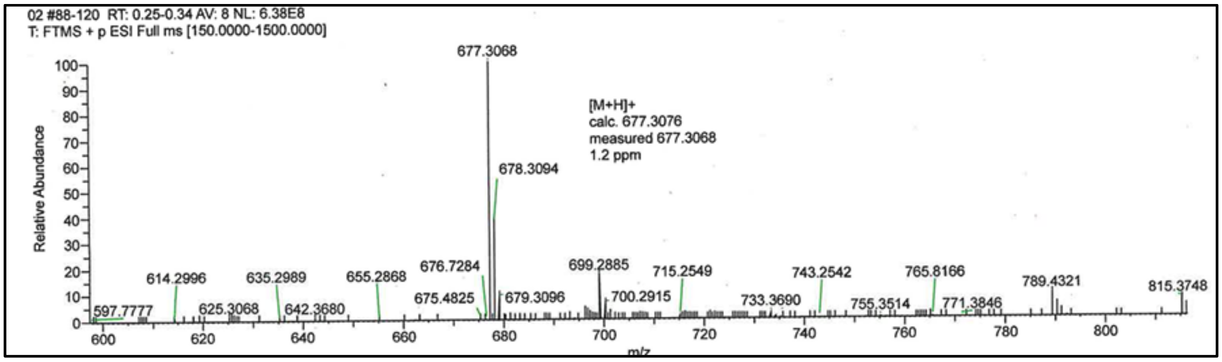

Figure S15. Characterization data for compound **15.**

2,2'-(7-(2-(((R)-1-((2-(((S)-1-amino-3-(1H-indol-3-yl)-1-oxopropan-2-yl)amino)-2-oxoethyl)amino)-3-hydroxy-1-oxopropan-2-yl)(methyl)amino)-2-oxoethyl)-1,4,7-triazonane-1,4-diyl)diacetic acid, **NOTA-^{NMe}S-G-W-CONH$_2$**. Compound **16** was synthesized using the general coupling strategy outlined in **Section 2.1** and **Scheme S2** from compound **Fmoc-Trp(Boc)-RA**. The product was isolated as a white solid following purification using semi-preparative HPLC (Method D). Yield: 26 mg, 0.041 mmol, 36%. R_t (Method A): 6.93 min. ^{1}H NMR (500 MHz, D_2O): δ 7.61 (d, 1H), 7.49 – 7.43 (d, 1H), 7.39 – 7.16 (m, 2H), 7.15 – 7.09 (t, 1H), 4.82 – 4.76 (m, 1H), 4.61 (dd, 1H), 3.89 – 3.83 (m, 2H), 3.80 – 3.66 (m, 8H), 3.50 (s, 4H), 3.33 – 3.12 (m, 6H), 3.07 (d, 4H), 2.78 (s, 3H). ^{13}C NMR (126 MHz, D_2O): δ 176.21 ($C^{21,23}$), 174.43 (C^{10}), 171.32 ($C^{12,14}$), 171.22 (C^{18}), 136.12 (C^1), 126.98 (C^6), 124.27 (C^7), 122.04 (C^3), 119.45 (C^4), 118.42 (C^5), 111.91 (C^2), 109.02 (C^8), 61.04 (C^{15}), 58.75 ($C^{20,22}$), 58.02 (C^{16}), 55.67 (C^{19}), 53.85 (C^{11}), 51.91 ($C^{cylc.}$), 50.22 ($C^{cycl.}$), 49.21 ($C^{cycl.}$), 42.51 (C^{13}), 31.75 (C^{17}), 26.94 (C^9). HR-ESI-MS $[M+H]^+$ calc. for $C_{29}H_{42}N_8O_9$ 647.3147, found 647.3142.

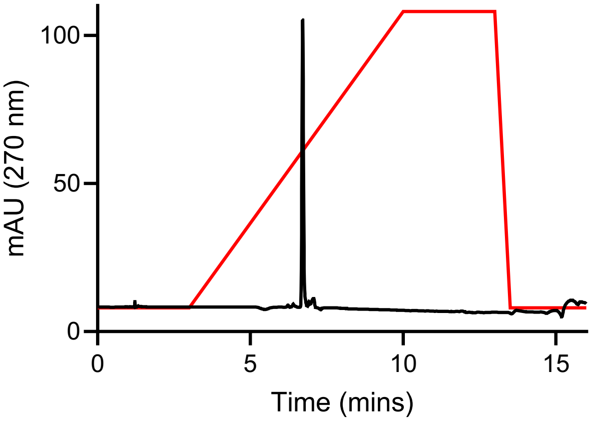

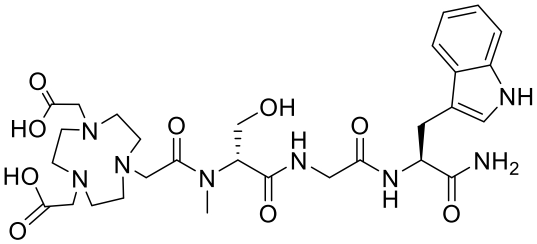

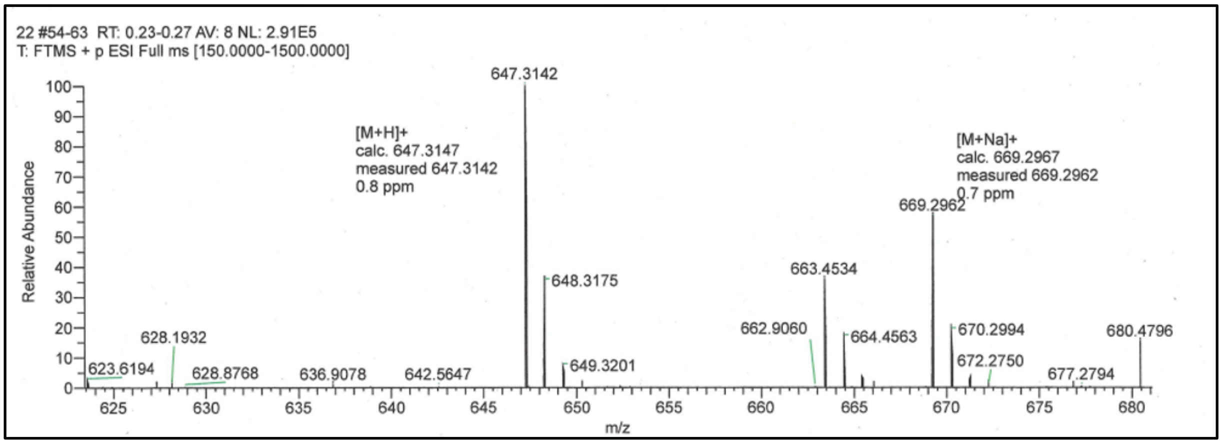

Figure S16. Characterization data for compound **16.**

(S)-2,2'-(7-(2-((2-((2-((1-amino-3-(1H-indol-3-yl)-1-oxopropan-2-yl)amino)-2-oxoethyl)amino)-2-oxoethyl)(methyl)amino)-2-oxoethyl)-1,4,7-triazonane-1,4-diyl)diacetic acid, **NOTA-^{NMe}G-G-W-CONH$_2$**. Compound **17** was synthesized using the general coupling strategy outlined in **Section 2.1** and **Scheme S2** from compound **Fmoc-Trp(Boc)-RA**. The product was isolated as a white solid following purification using semi-preparative HPLC (Method D). Yield: 26 mg, 0.041 mmol, 36%. R_t (Method A): 6.61 min. ^{1}H NMR (500 MHz, D_2O) δ 7.72 (d, 1H), 7.55 (d, 1H), 7.35 – 7.26 (m, 2H), 7.21 (t, 1H), 4.74 – 4.67 (m, 1H), 4.19 – 4.00 (m, 2H), 4.00 – 3.88 (m, 2H), 3.81 (s, 2H), 3.70 – 3.49 (m, 4H), 3.45 – 3.03 (m, 9H), 3.00 (s, 2H), 2.91 (s, 1H). ^{13}C NMR (126 MHz, D2O) δ 176.21, 173.93, 171.75, 171.18, 170.79, 136.11, 126.92, 124.37, 122.01, 119.42, 118.39, 111.91, 109.00, 58.65, 55.40, 53.91, 51.83, 51.65, 50.19, 49.18, 42.46, 35.33, 26.93.HR-ESI-MS $[M+H]^+$ calc. for $C_{28}H_{40}N_8O_8$ 617.3042, found 617.3035.

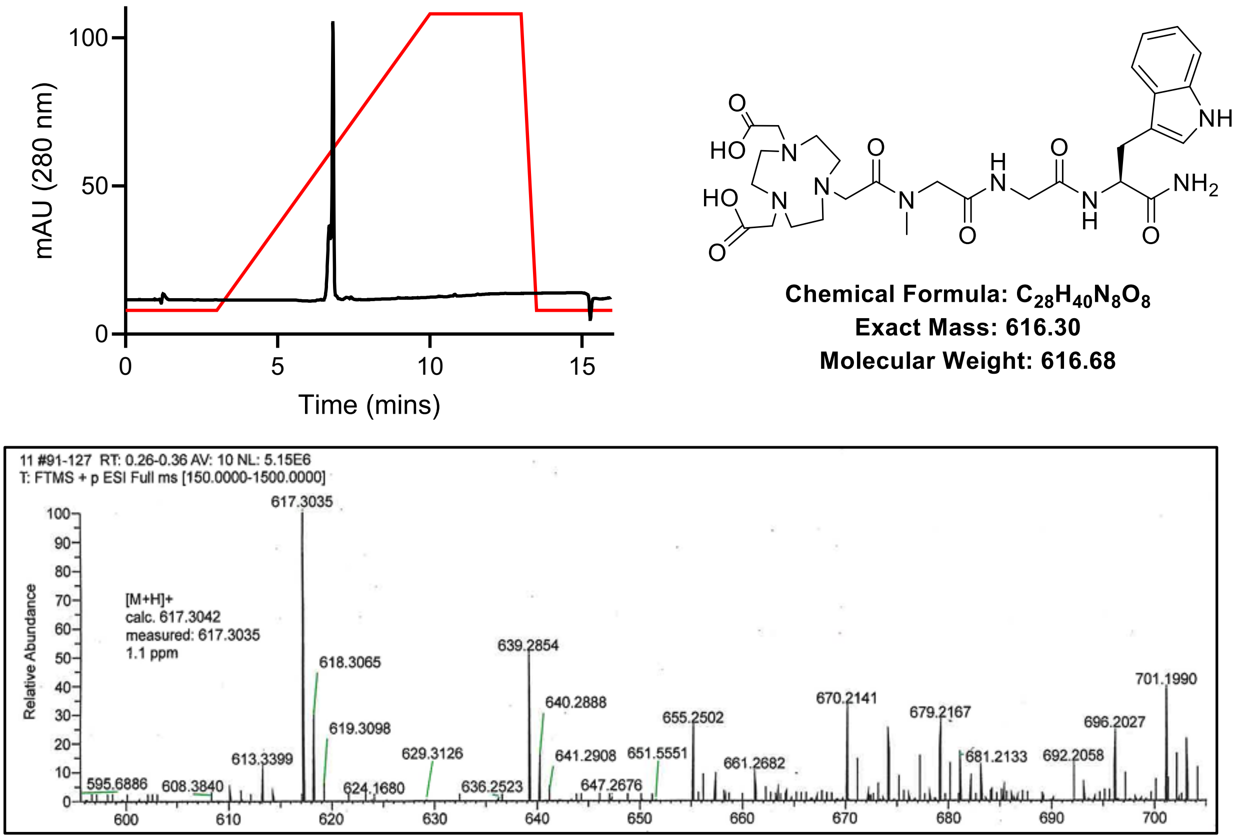

Figure S17. Characterization data for compound **17.**

2,2'-(7-(2-(((R)-1-((2-(((S)-1-amino-3-(1H-indol-3-yl)-1-oxopropan-2-yl)amino)-2-oxoethyl)amino)-4-(methylthio)-1-oxobutan-2-yl)(methyl)amino)-2-oxoethyl)-1,4,7-triazonane-1,4-diyl)diacetic aci, **NOTA-^{NMe}M-G-W-CONH$_2$**. Compound **18** was synthesized using the general coupling strategy outlined in **Section 2.1** and **Scheme S2** from compound **Fmoc-Trp(Boc)-RA**. The product was isolated as a white solid following purification using semi-preparative HPLC (Method D). Yield: 26 mg, 0.041 mmol, 36%. R_t (Method A): 6.93 min. ^{1}H NMR (500 MHz, D_2O) δ 7.69 (d, 1H), 7.55 (d, 1H), 7.33 – 7.24 (m, 2H), 7.21 (t, 1H), 5.03 (dd, 1H), 4.75 – 4.68 (m, 1H), 4.16 – 3.90 (m, 2H), 3.94 – 3.82 (m, 6H), 3.55 – 3.28 (m, 14H), 2.76 (s, 3H), 2.54 – 2.36 (m, 2H), 2.10 (s, 3H), 2.01 – 1.88 (m, 1H). ^{13}C NMR (126 MHz, D_2O): δ 176.14 ($C^{23,25}$), 172.26 (C^{10}), 172.15 (C^{12}), 171.66 (C^{14}), 170.83 (C^{20}), 136.12 (C^{1}), 126.98 (C^{6}), 124.32 (C^{7}), 122.04 (C^{3}), 119.46 (C^{4}), 118.38 (C^{5}), 111.92 (C^{5}), 108.84 (C^{8}), 57.65 ($C^{22,24}$), 56.99 (C^{15}), 56.34 (C^{21}), 53.73 (C^{11}), 51.09 (C^{cycl}), 50.41 ($C^{cycl.}$), 42.66 (C^{13}), 30.60 (C^{19}), 29.48 (C^{17}), 26.96 (C^{9}), 26.06 (C^{18}), 14.10 (C^{16}). HR-ESI-MS $[M-H]^-$ calc. for $C_{31}H_{46}N_8O_8S$ 689.3087, found 689.3094.

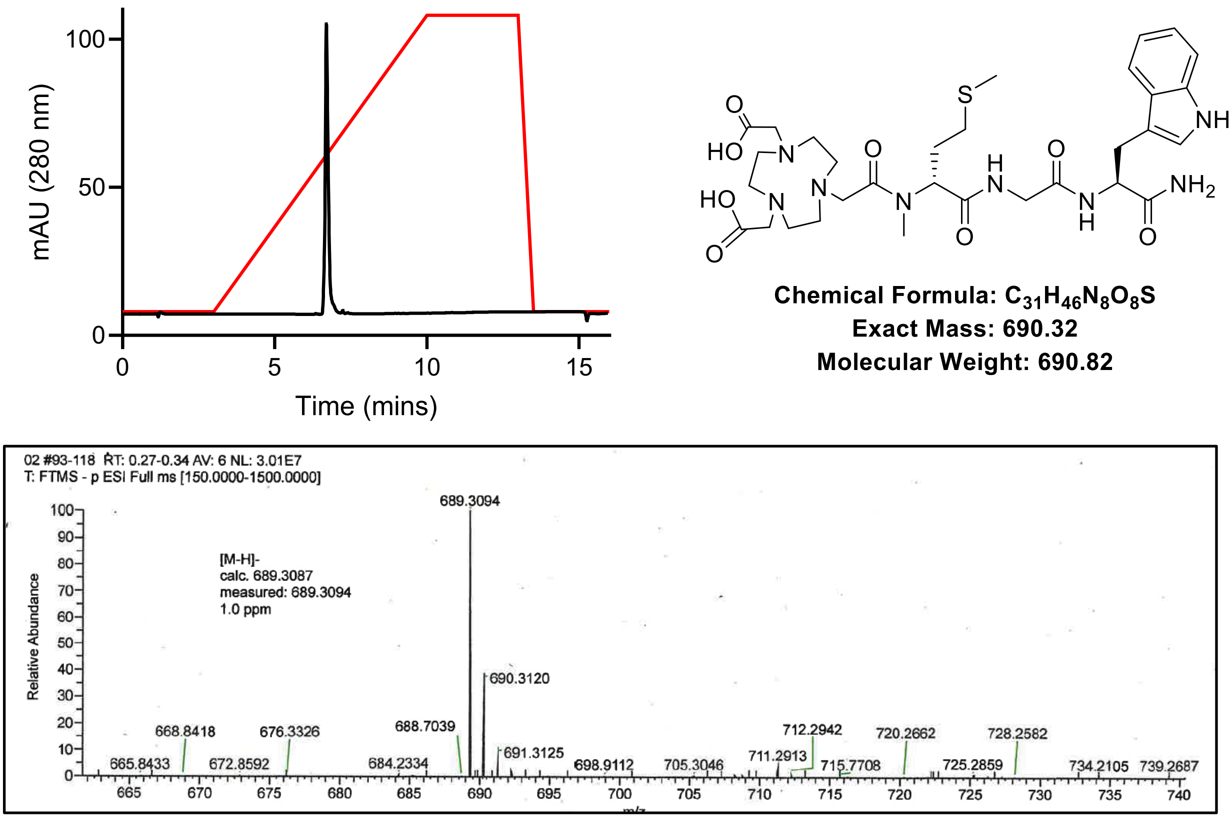

Figure S18. Characterization data for compound **18**.

(S)-2,2'-(7-(2-((3-((2-((1-amino-3-(1H-indol-3-yl)-1-oxopropan-2-yl)amino)-2-oxoethyl)amino)-3-oxopropyl)(methyl)amino)-2-oxoethyl)-1,4,7-triazonane-1,4-diyl)diacetic acid, **NOTA-$^{NMe\beta}$A-G-W-CONH$_2$**. Compound **20** was synthesized using the general coupling strategy outlined in **Section 2.1** and **Scheme S2** from compound **Fmoc-Trp(Boc)-RA**. The product was isolated as a white solid following purification using semi-preparative HPLC (Method D) . Yield: 26 mg, 0.041 mmol, 36%. R_t (Method A): 6.22 min. ^{1}H NMR (500 MHz, D_2O): δ 7.83 (d, 1H), 7.65 (d, 1H), 7.40 (m, 2H), 7.32 (ddd, 1H), 4.82 – 4.74 (m, 1H), 4.04 – 3.95 (m, 4H), 3.93 (d, 2H), 3.79 (d, 4H), 3.68 – 3.14 (m, 14H), 3.00 (d, 3H), 2.58 (td, 2H). ^{13}C NMR (126 MHz, D_2O): δ 176.34 ($C^{21,23}$), 174.52 (C^{10}), 171.32 ($C^{12,14}$), 169.46 (C^{18}), 136.17 (C^1), 126.88 (C^6), 124.42 (C^7), 122.02 (C^3), 119.44 (C^4), 118.38 (C^5), 111.92 (C^2), 109.07 (C^8), 58.96 ($C^{20,22}$), 55.79 (C^{19}), 53.96 (C^{11}), 52.01 ($C^{cycl.}$), 51.73 ($C^{cycl.}$), 50.27 ($C^{cycl.}$), 45.80 (C^{16}), 42.53 (C^{13}), 35.18 (C^{17}), 33.24 (C^{15}), 26.93 (C^9). HR-ESI-MS $[M+H]^+$ calc. for $C_{29}H_{42}N_8O_8$ 631.3198, found 631.3194.

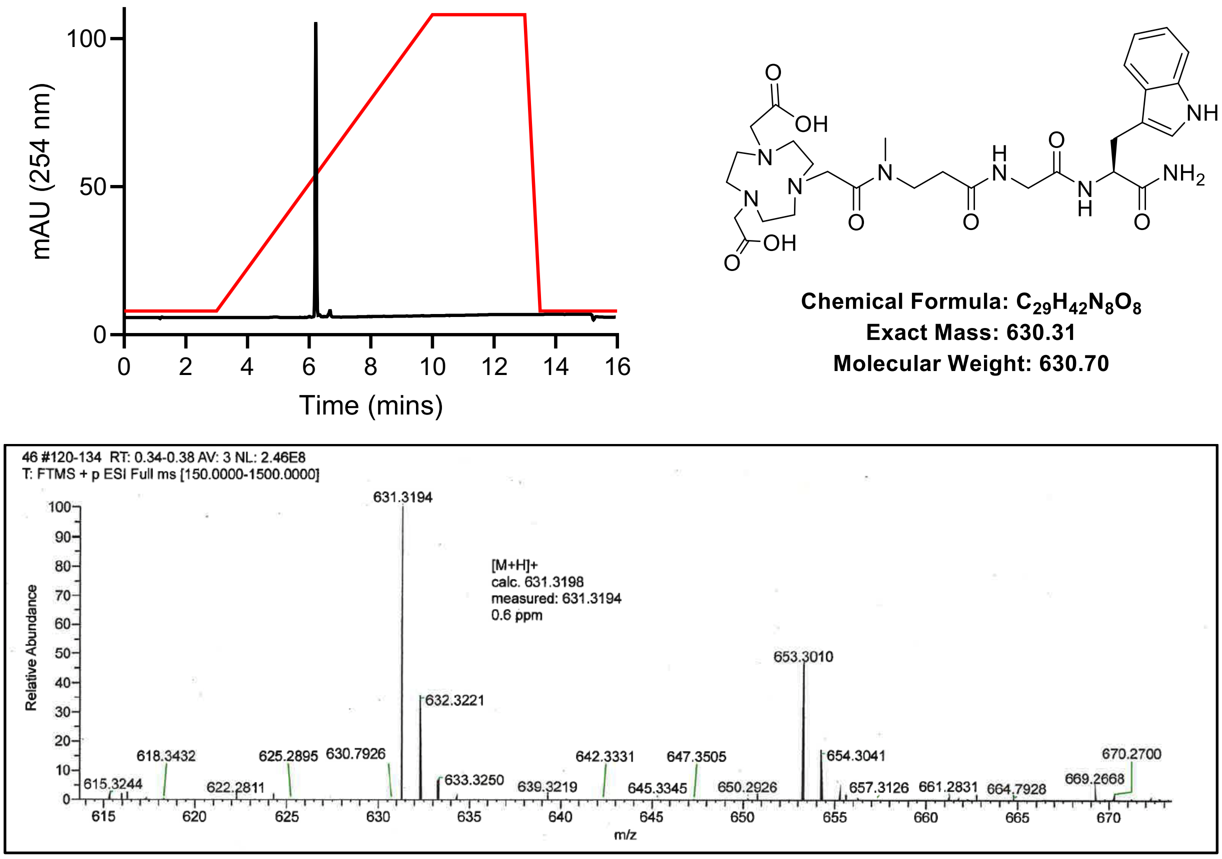

Figure S19. Characterization data for compound **19.**

(7R,21S,25S)-1-(4,7-bis(carboxymethyl)-1,4,7-triazonan-1-yl)-7-(4-hydroxy-3-iodobenzyl)-2,5,8,15,23-pentaoxo-3,6,9,16,22,24-hexaazaheptacosane-21,25,27-tricarboxylic acid, **NO2A-G-Tyr(3-I)-Ahx-KuE**. Compound **19** (200 mg,0.128 mmol) was synthesized using the general coupling strategy outlined in **Section 2.2** and **Scheme S3** and **Scheme S4.** The product was isolated as a white solid following the deprotection and purification using semi-preparative HPLC (Method D) and characterized using mass spectrometry and HPLC chromatography. Yield: (0.027 mg, 0.025 mmol, 16%). R_t (Method A): 6.51 min. HR-ESI-MS $[M-H]^-$ calc. for $C_{41}H_{62}IN_9O_{16}$ 1062.3286, found 1062.3275.

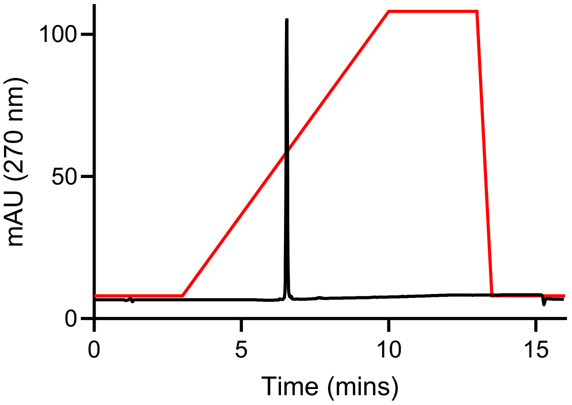

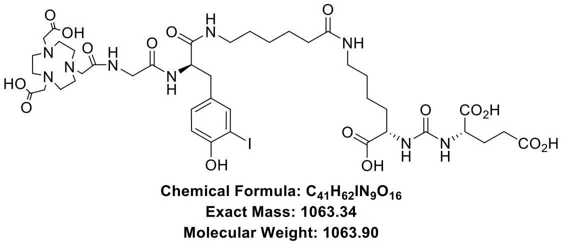

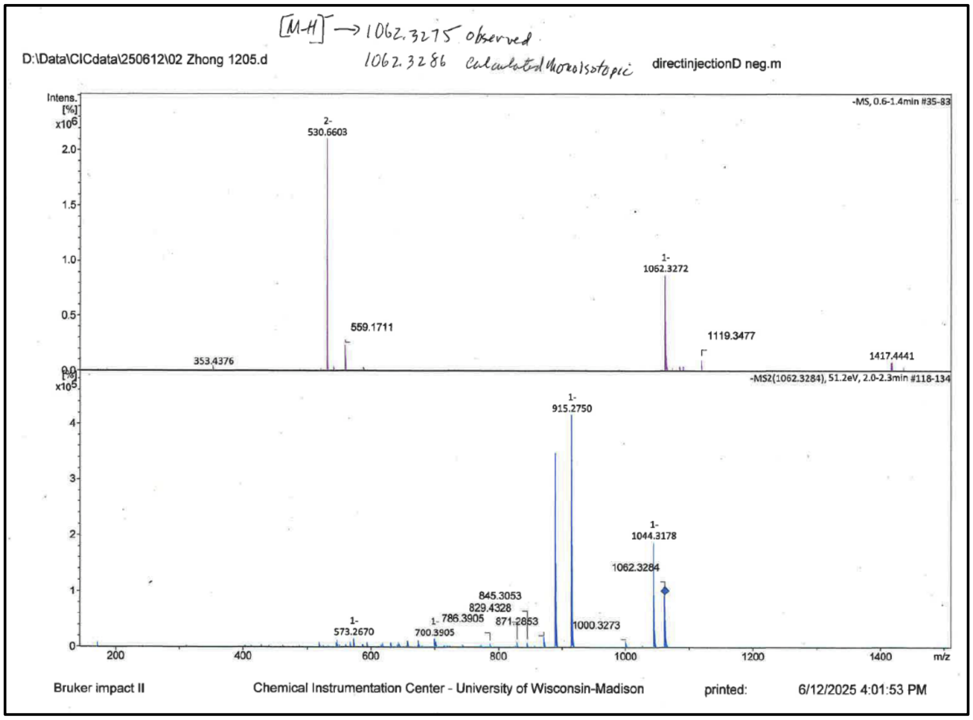

Figure S20. Characterization data for compound **20**.

(4S,7R,21S,25S)-1-(4,7-bis(carboxymethyl)-1,4,7-triazonan-1-yl)-7-(4-hydroxy-3-iodobenzyl)-4-(hydroxymethyl)-2,5,8,15,23-pentaoxo-3,6,9,16,22,24-hexaazaheptacosane-21,25,27-tricarboxylic acid, **NO2A-S-Tyr(3-I)-Ahx-KuE**. Compound **21** (200 mg,0.128 mmol) was synthesized using the general coupling strategy outlined in **Section 2.2** and **Scheme S3** and **Scheme S4**. The product was isolated as a white solid following the deprotection and purification using Semi-preparative HPLC (Method D) and characterized using mass spectrometry and HPLC chromatography. Yield: (0.020 mg, 0.016 mmol, 11%). R_t (Method A): 7.20 min. HR-ESI-MS $[M-H]^-$ calc. for $C_{42}H_{64}IN_9O_{17}$ 1092.3392, found 1092.3376.

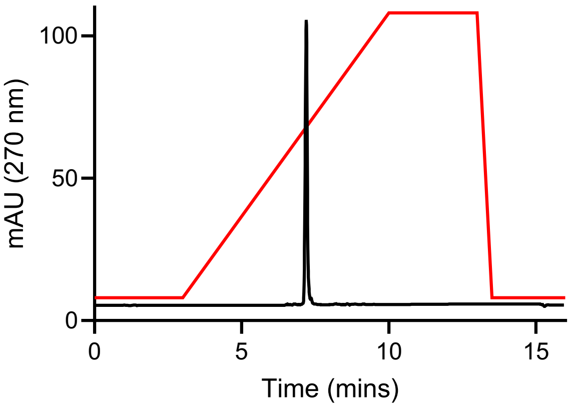

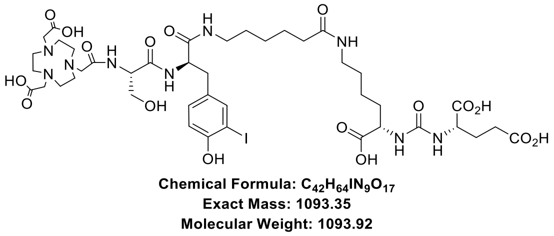

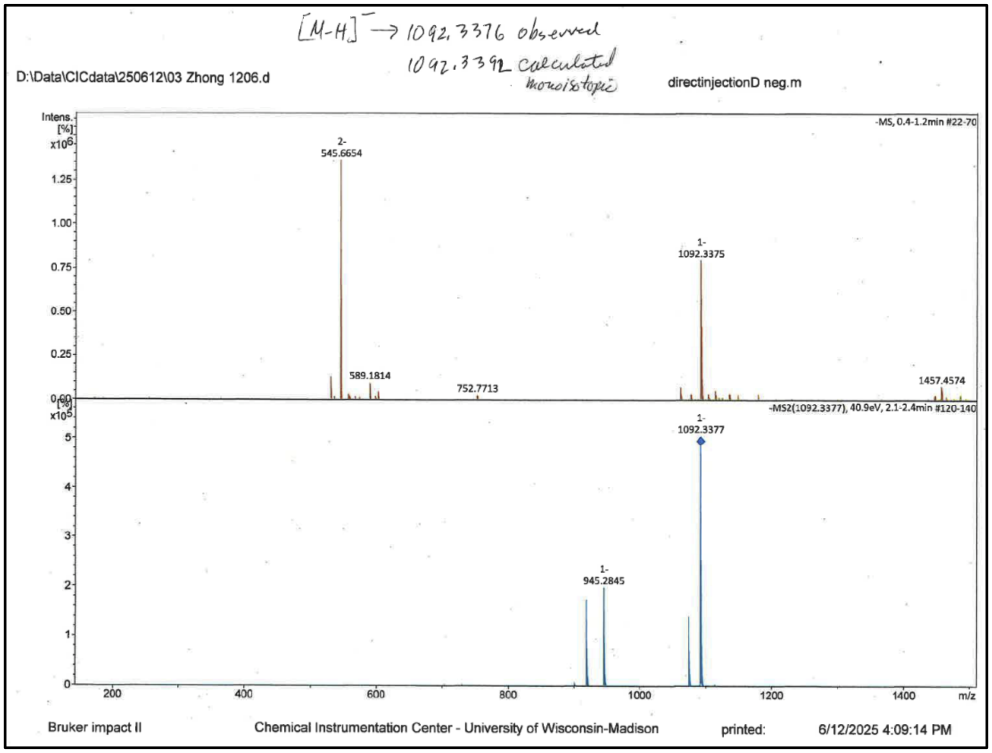

Figure S21. Characterization data for compound **21.**

(5S,8R,22S,26S)-5-(2-(4,7-bis(carboxymethyl)-1,4,7-triazonan-1-yl)acetamido)-8-(4-hydroxy-3-iodobenzyl)-6,9,16,24-tetraoxo-2-thia-7,10,17,23,25-pentaazaoctacosane-22,26,28-tricarboxylic acid, **NO2A-M-Tyr(3-I)-Ahx-KuE**. Compound **22** (200 mg,0.128 mmol) was synthesized using the general coupling strategy outlined in **Section 2.2** and **Scheme S3** and **Scheme S4**. The product was isolated as a white solid following the deprotection and purification using semi-preparative HPLC (Method D) and characterized using mass spectrometry and HPLC chromatography. Yield: (0.023 mg, 0.020 mmol, 13%). R_t (Method A): 6.55 min. HR-ESI-MS $[M-H]^-$ calc. for $C_{44}H_{68}IN_9O_{16}S$ 1136.3477, found 1136.3469.

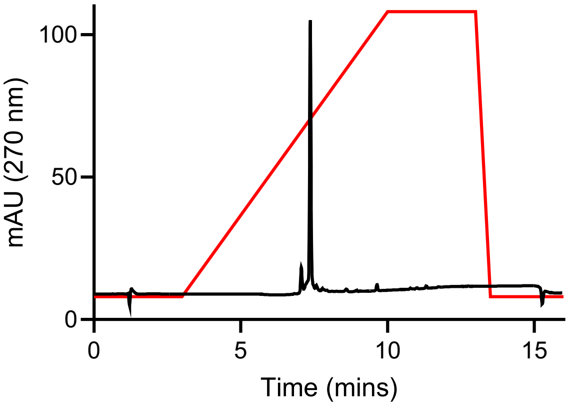

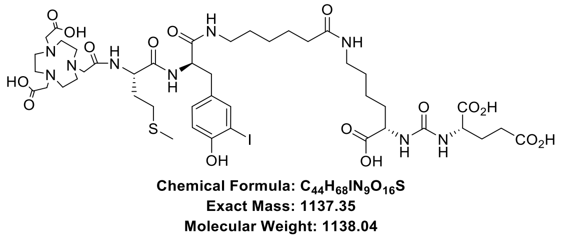

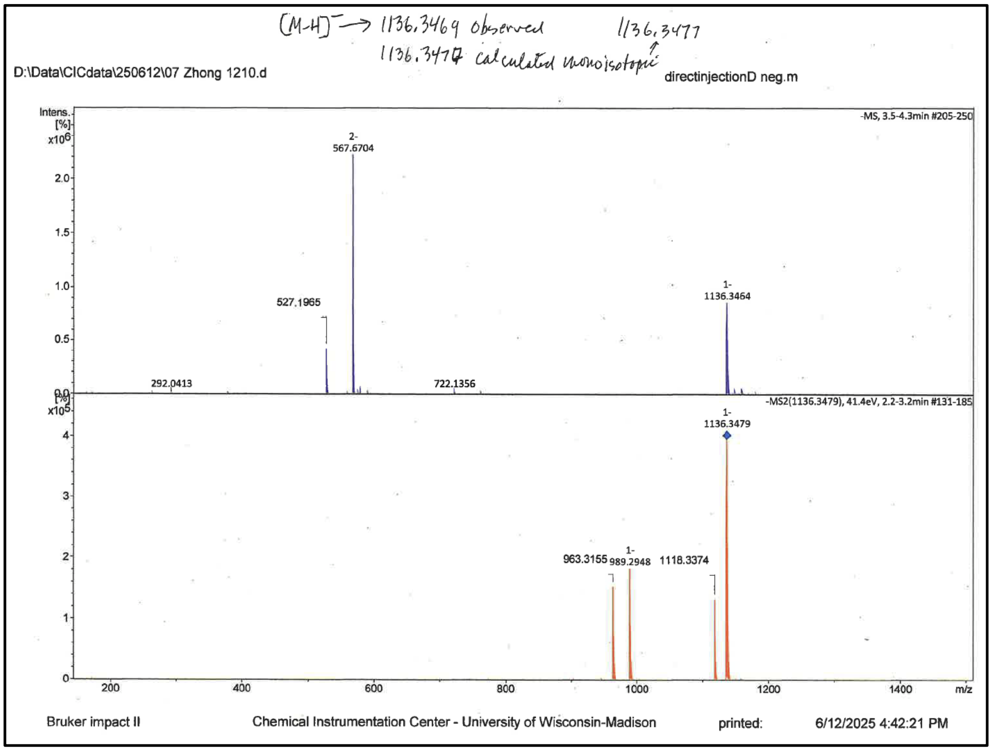

Figure S22. Characterization data for compound **22.**

(7R,21S,25S)-1-(4,7-bis(carboxymethyl)-1,4,7-triazonan-1-yl)-7-(4-hydroxy-3-iodobenzyl)-3-methyl-2,5,8,15,23-pentaoxo-3,6,9,16,22,24-hexaazaheptacosane-21,25,27-tricarboxylic acid, **NO2A-$^{\mathbf{NMe}}$G-Tyr(3-I)-Ahx-KuE**. Compound **22** (200 mg,0.128 mmol) was synthesized using the general coupling strategy outlined in **Section 2.2** and **Scheme S3** and **Scheme S4**. The product was isolated as a white solid following the deprotection and purification using semi-preparative HPLC (Method D) and characterized using mass spectrometry and HPLC chromatography. Yield: (0.026 mg, 0.024 mmol, 15%). R_t (Method A): 7.18 min. HR-ESI-MS $[M-H]^-$ calc. for $C_{42}H_{64}IN_9O_{16}$ 1076.3442, found 1076.3430.

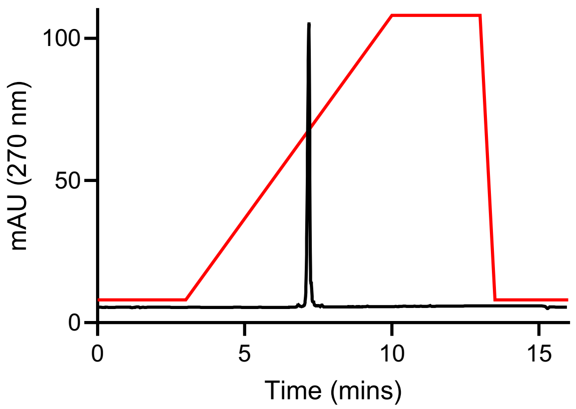

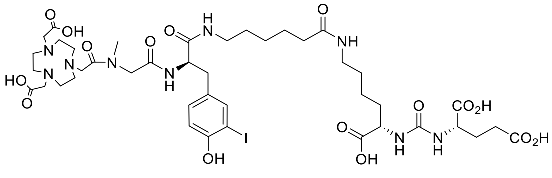

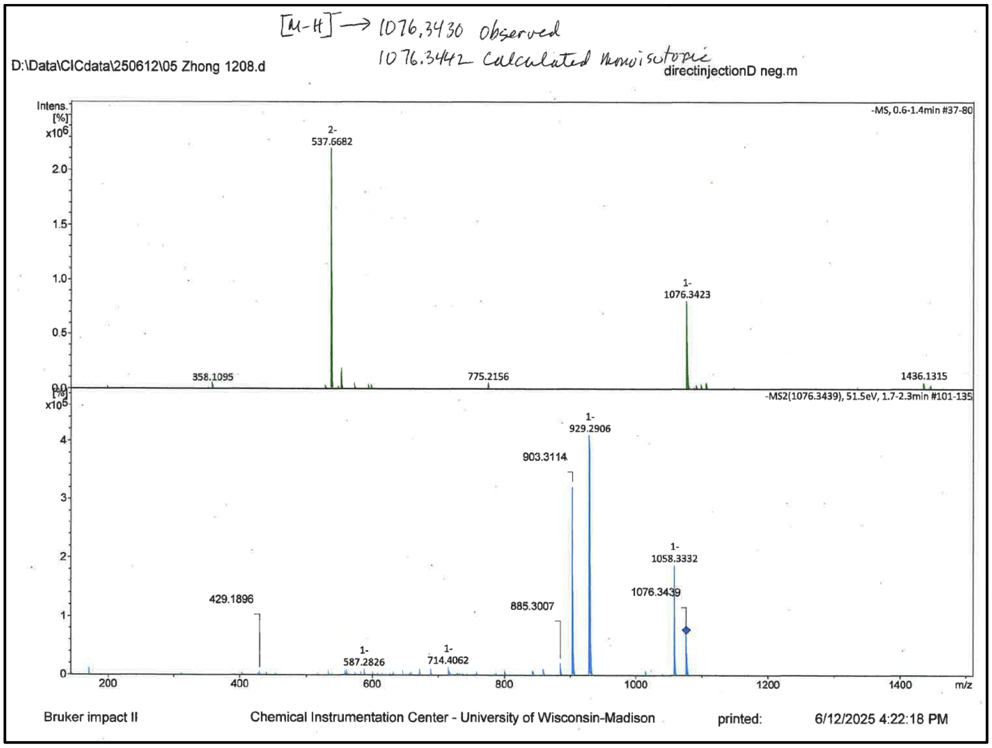

Figure S23. Characterization data for compound **23.**

(4S,7R,21S,25S)-1-(4,7-bis(carboxymethyl)-1,4,7-triazonan-1-yl)-7-(4-hydroxy-3-iodobenzyl)-4-(hydroxymethyl)-3-methyl-2,5,8,15,23-pentaoxo-3,6,9,16,22,24-hexaazaheptacosane-21,25,27-tricarboxylic acid, **NO2A-^{NMe}S-Tyr(3-I)-Ahx-KuE**. Compound **24** was synthesized using the general coupling strategy outlined in **Section 2.2** and **Scheme S3** and **Scheme S4**. The product was isolated as a white solid following the deprotection and purification using semi-preparative HPLC (Method D) and characterized using mass spectrometry and HPLC chromatography. Yield: (0.028 mg, 0.025 mmol, 16%). R_t (Method A): 6.53 min. HR-ESI-MS $[M-H]^-$ calc. for $C_{43}H_{66}IN_9O_{17}$ 1106.3549, found 1106.3581.

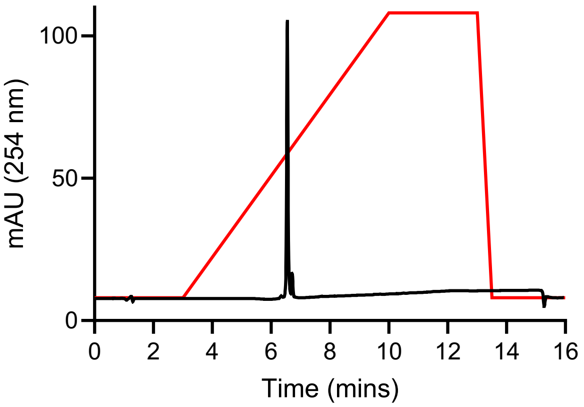

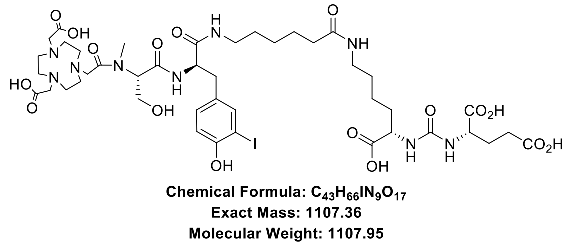

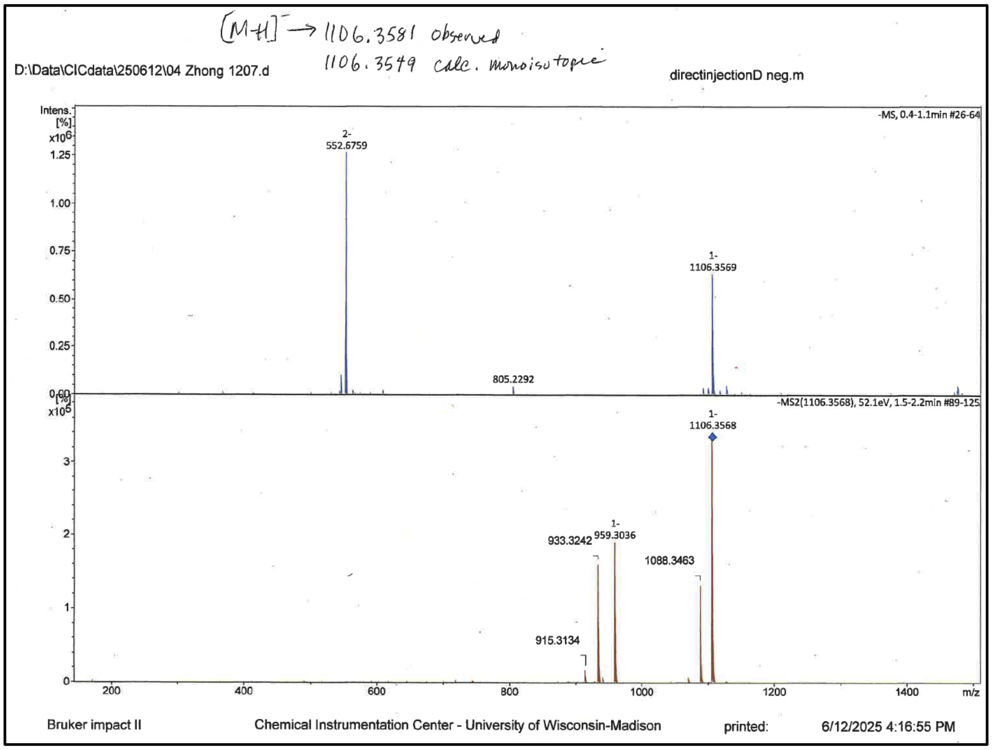

Figure S24. Characterization data for compound **24.**

(5S,8R,22S,26S)-5-(2-(4,7-bis(carboxymethyl)-1,4,7-triazonan-1-yl)-N-methylacetamido)-8-(4-hydroxy-3-iodobenzyl)-6,9,16,24-tetraoxo-2-thia-7,10,17,23,25-pentaazaoctacosane-22,26,28-tricarboxylic acid, **NO2A-$^{\mathbf{NMe}}$M-T-Ahx-KuE**. Compound **25** (200 mg,0.128 mmol) was synthesized using the general coupling strategy outlined in **Section 2.2** and **Scheme S3** and **Scheme S4**. The product was isolated as a white solid following the deprotection and purification using semi-preparative HPLC (Method D) and characterized using mass spectrometry and HPLC chromatography. Yield: (0.024 mg, 0.021 mmol, 13%). R_t (Method A): 7.44 min. HR-ESI-MS $[M-H]^-$ calc. for $C_{45}H_{70}IN_9O_{16}S$ 1150.3633, found 1150.3602.

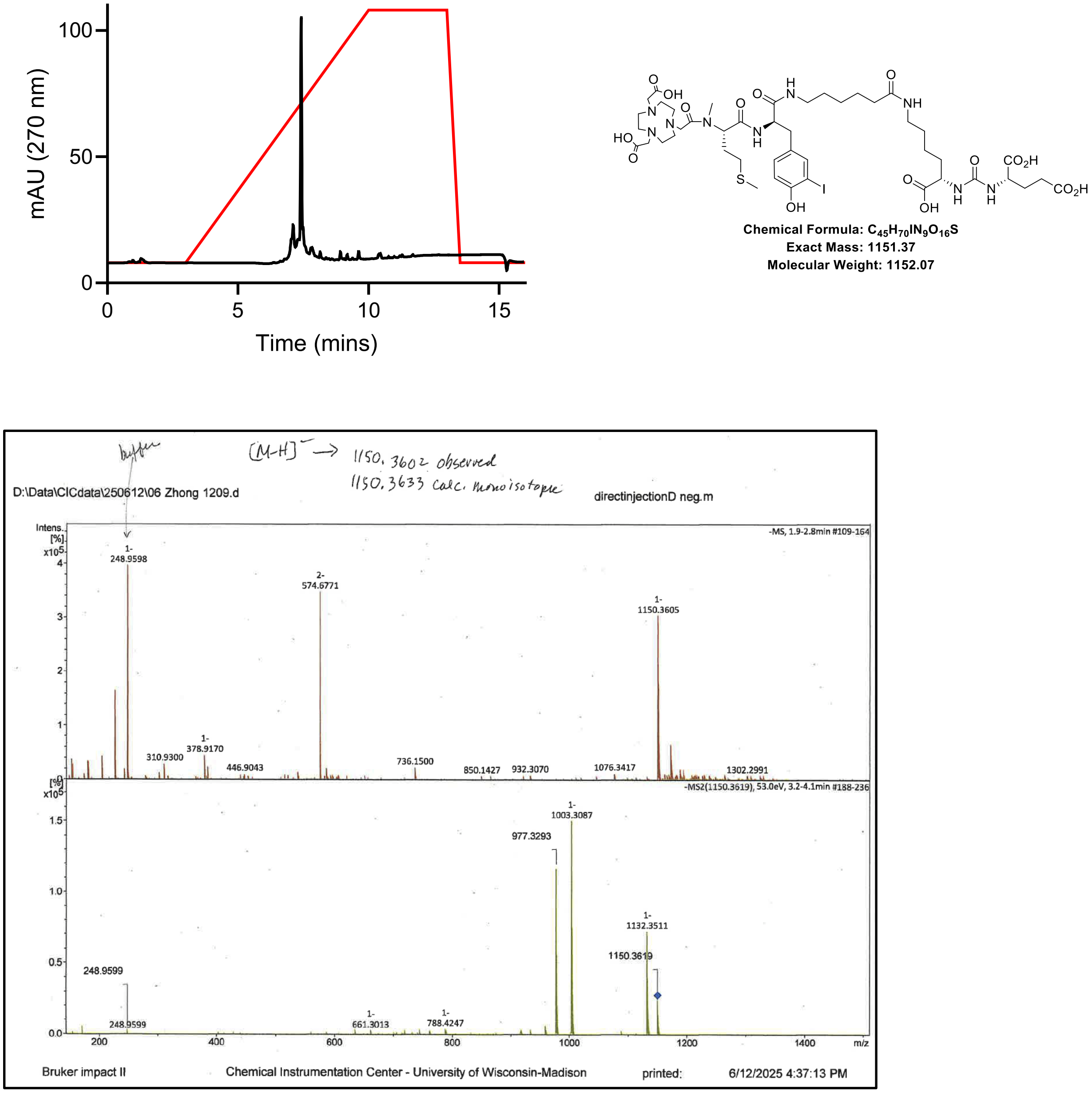

Figure S25. Characterization data for compound **25.**

2,2'-(7-(2-(benzylamino)-2-oxoethyl)-1,4,7-triazonane-1,4-diyl)diacetic acid, **26**. Compound **26** was synthesized using the general alkylation strategy outlined in **Section 2.3** and **Scheme S5**. The product was isolated as a white solid following the deprotection and purification using flash chromatography (Method E) and characterized using mass spectrometry and HPLC chromatography. Yield: 178 mg, 0.72 mmol, 63%. R_t (Method A): 6.05 min. ^{1}H NMR (500 MHz, D_2O) δ 7.52 – 7.26 (m, 5H), 4.46 (s, 2H), 3.81 (d, 6H), 3.40 – 2.98 (m, 12H). ^{13}C NMR (126 MHz, D_2O) δ 171.90 ($C^{11,13}$), 171.32 (C^8), 137.70 (C^6), 128.88 ($C^{1,5}$), 127.66 ($C^{2,4}$), 127.37 (C^3), 58.79 (C^9), 56.05 ($C^{10,12}$), 50.61 ($C^{cycl.}$), 49.72 ($C^{cycl.}$), 43.05 (C^7). HR-ESI-MS $[M+H]^-$ calc. for $C_{19}H_{28}N_4O_5S$: 393.2132, found 393.2128.

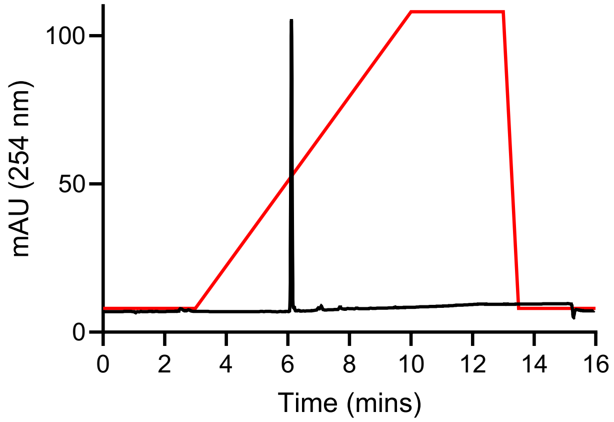

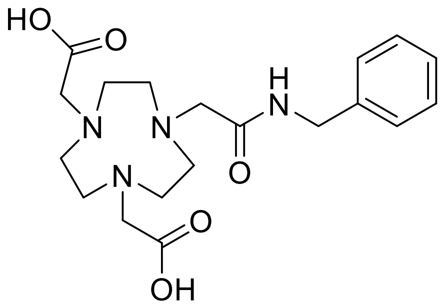

Chemical Formula: $C_{19}H_{28}N_4O_5$
Exact Mass: 392.21
Molecular Weight: 392.46

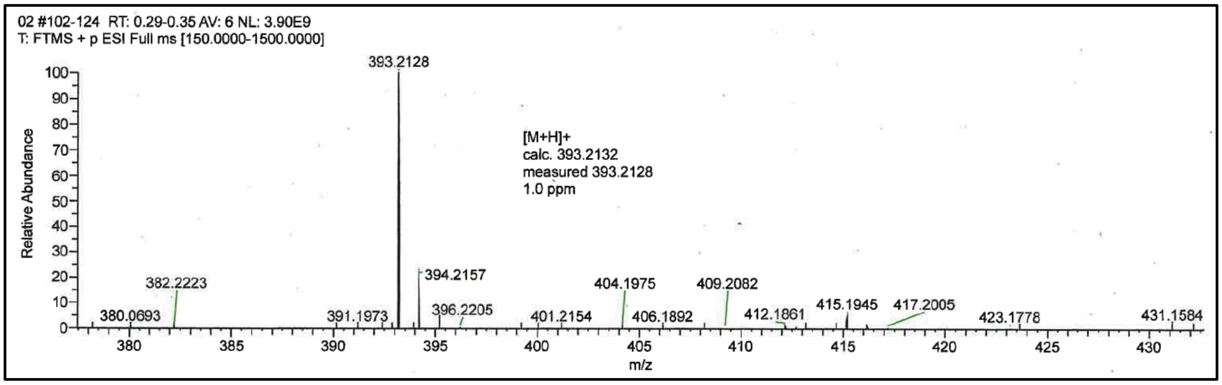

Figure S26. Characterization data for compound **26**.

2,2'-(7-(2-(benzyl(methyl)amino)-2-oxoethyl)-1,4,7-triazonane-1,4-diyl)diacetic acid,. Compound **27** was synthesized using the general coupling strategy outlined in **Section 2.3** and **Scheme S6**. The product was isolated as a white solid following the deprotection and purification using flash chromatography (Method E) and characterized using mass spectrometry and HPLC chromatography. Yield: (181 mg, mmol, 38%). R_t (Method E): 6.39 min. ^{1}H NMR (500 MHz, MeOD) δ 7.45 – 7.26 (m, 5H), 4.61 (d, 2H), 4.18 (d, 2H), 3.75 (s, 4H), 3.33 (p, 2H), 3.29 – 3.02 (m, 12H), 2.97 (d, 3H). ^{13}C NMR (126 MHz, MeOD) δ 172.18 (C^{12}), 172.11 (C^{14}), 167.94 (C^9),136.57 (C^6), 128.72 (C^1), 128.36 (C^5), 127.67 (C^2), 127.25 (C^4), 126.43 (C^3), 55.16 (C^{10}), 54.59 (C^{11}), 54.55 (C^{13}), 50.97 (C^7), 50.69 ($C^{cycl.}$), 49.71 ($C^{cycl.}$), 32.79 (C^8). HR-ESI-MS $[M-H]^-$ calc. for $C_{20}H_{30}N_4O_5$ 405.2143, found 405.2145.

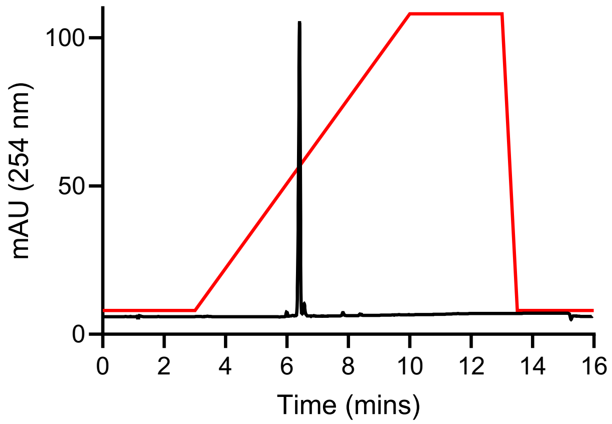

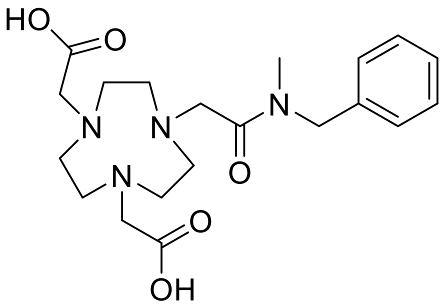

Chemical Formula: $C_{20}H_{30}N_4O_5$
Exact Mass: 406.22
Molecular Weight: 406.48

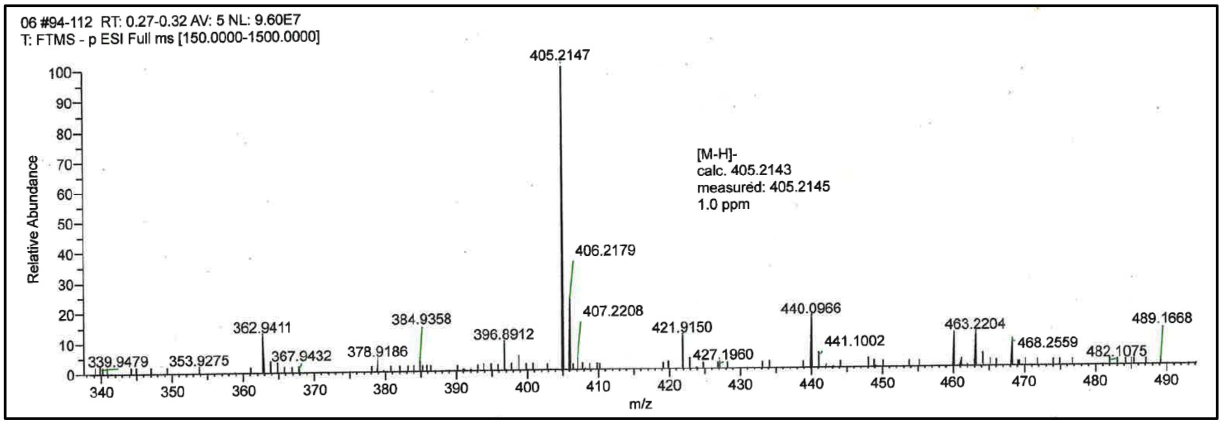

Figure S27. Characterization data for compound **27**.

2.5 NMR Spectra of Peptides

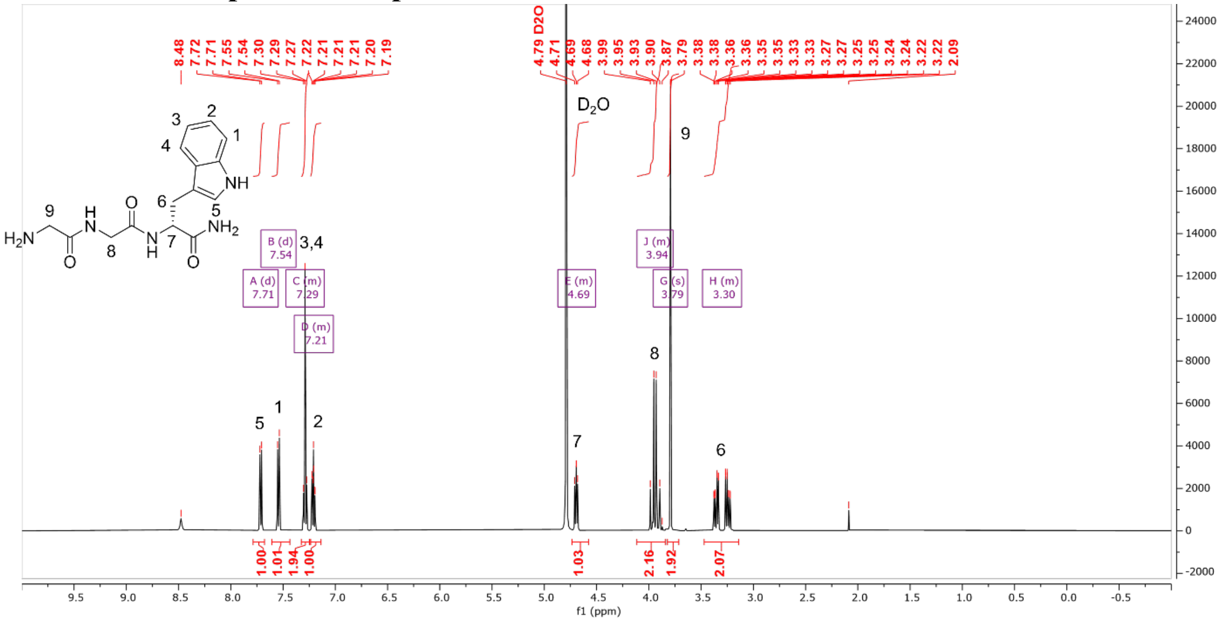

Figure S28. ^{1}H NMR (500 MHz, D_2O) for compound **1**.

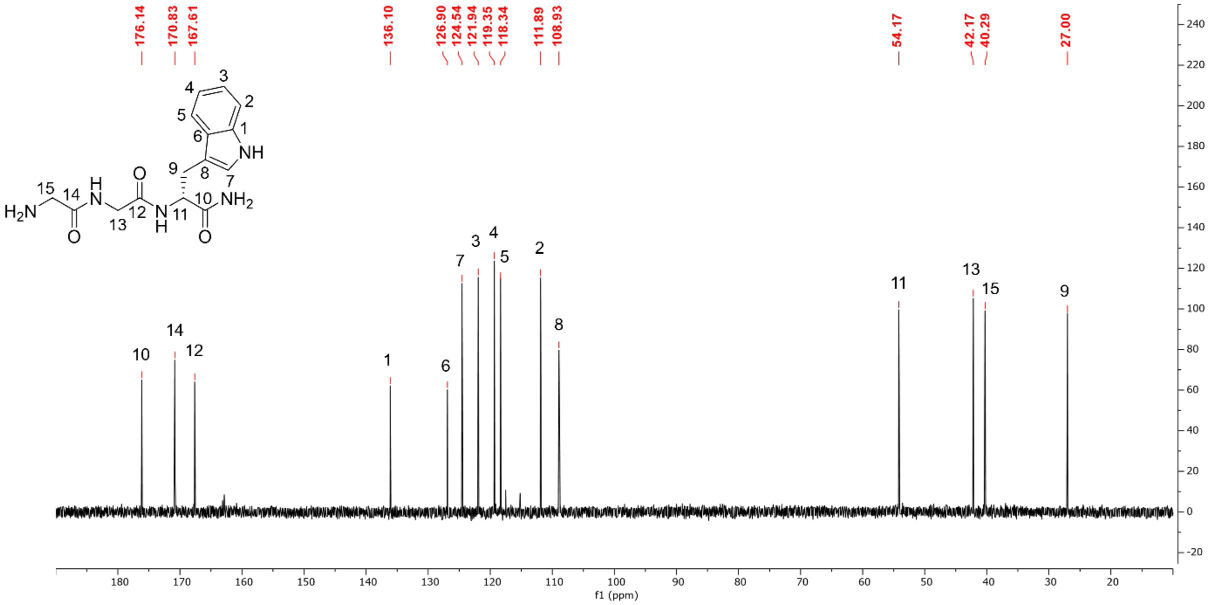

Figure S29. ^{13}C NMR (126 MHz, D_2O) for compound **1**.

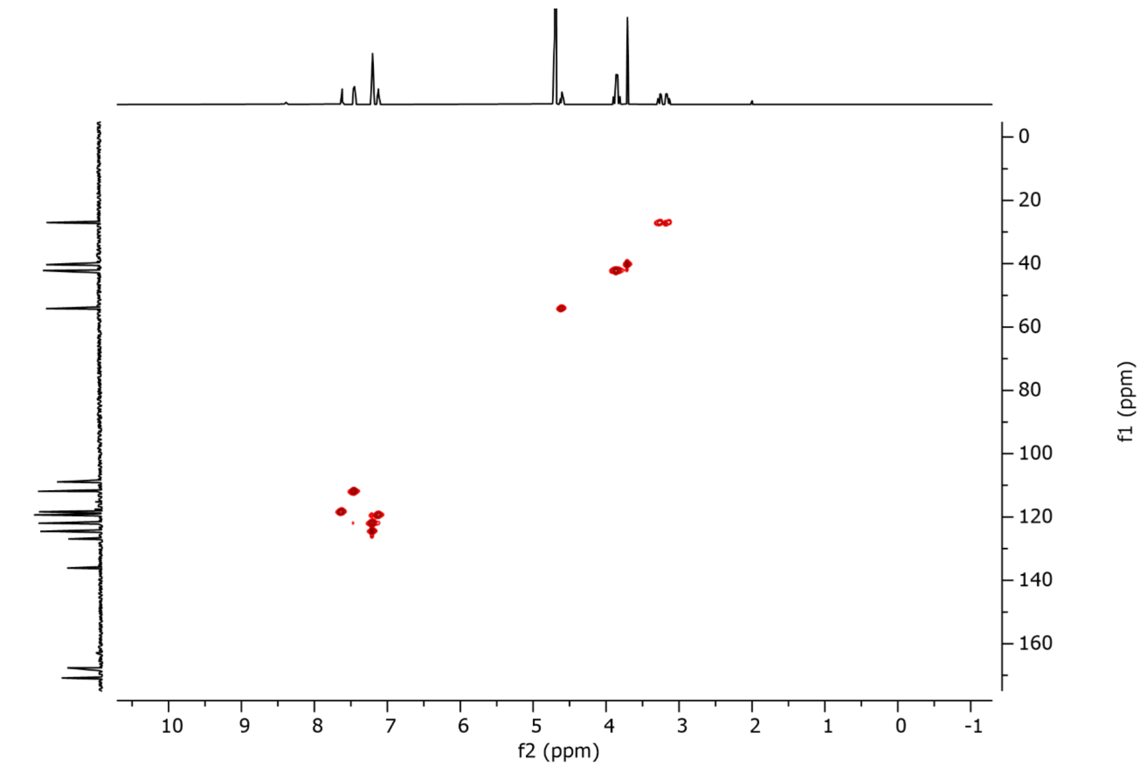

Figure S30. The HSQC NMR spectrum of compound **1** in D_2O.

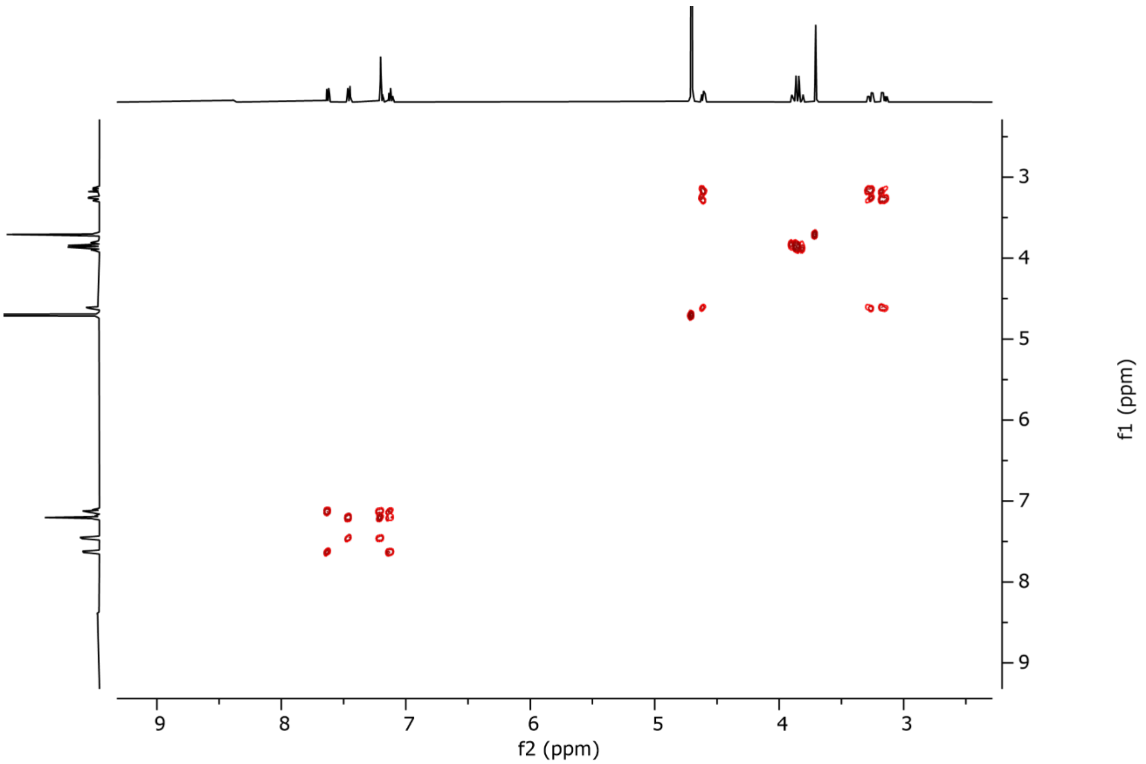

Figure S31. The ^1H-^1H COSY NMR spectrum of compound **1** in D_2O.

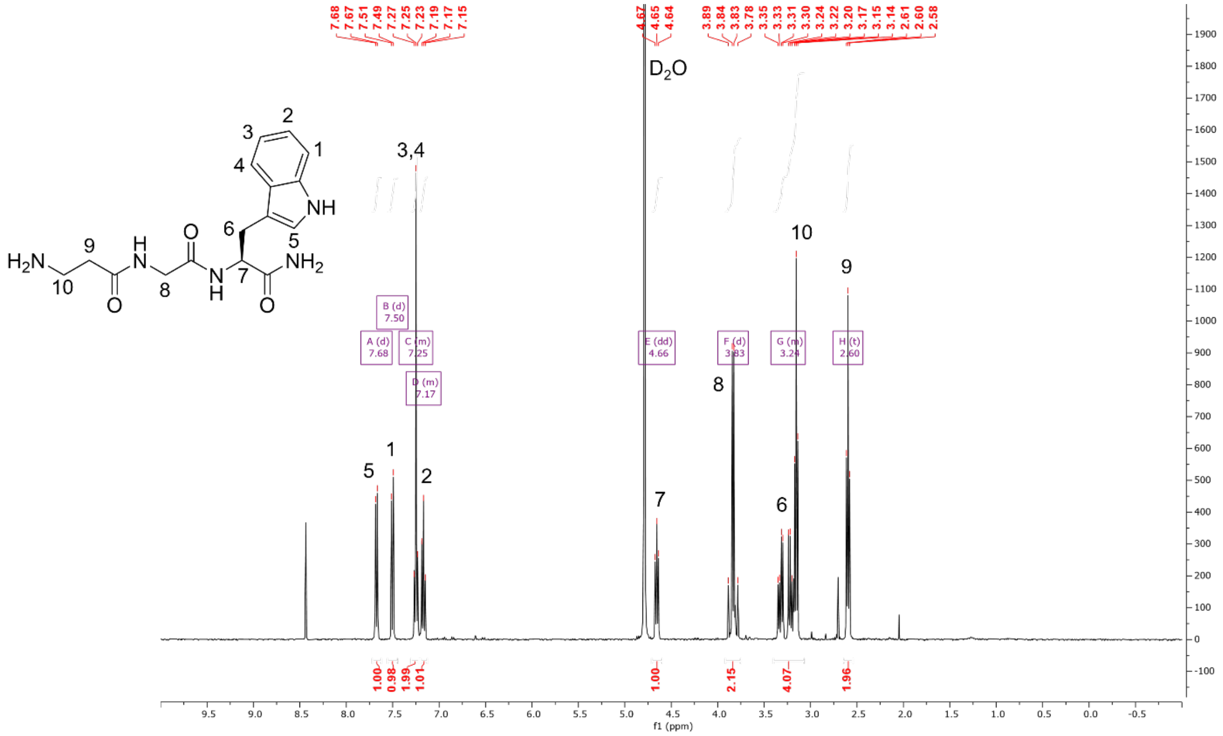

Figure S32. ^{1}H NMR (500 MHz, D_2O) for compound **2**.

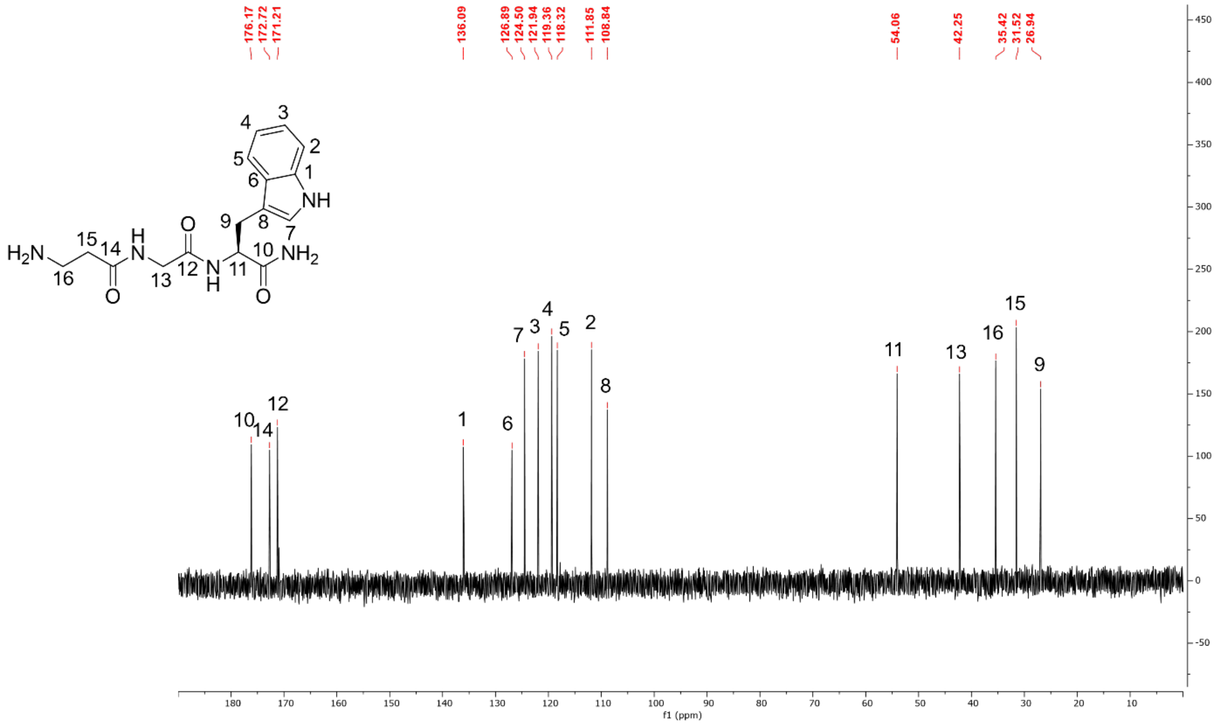

Figure S33. ^{13}C NMR (126 MHz, D_2O) for compound **2**.

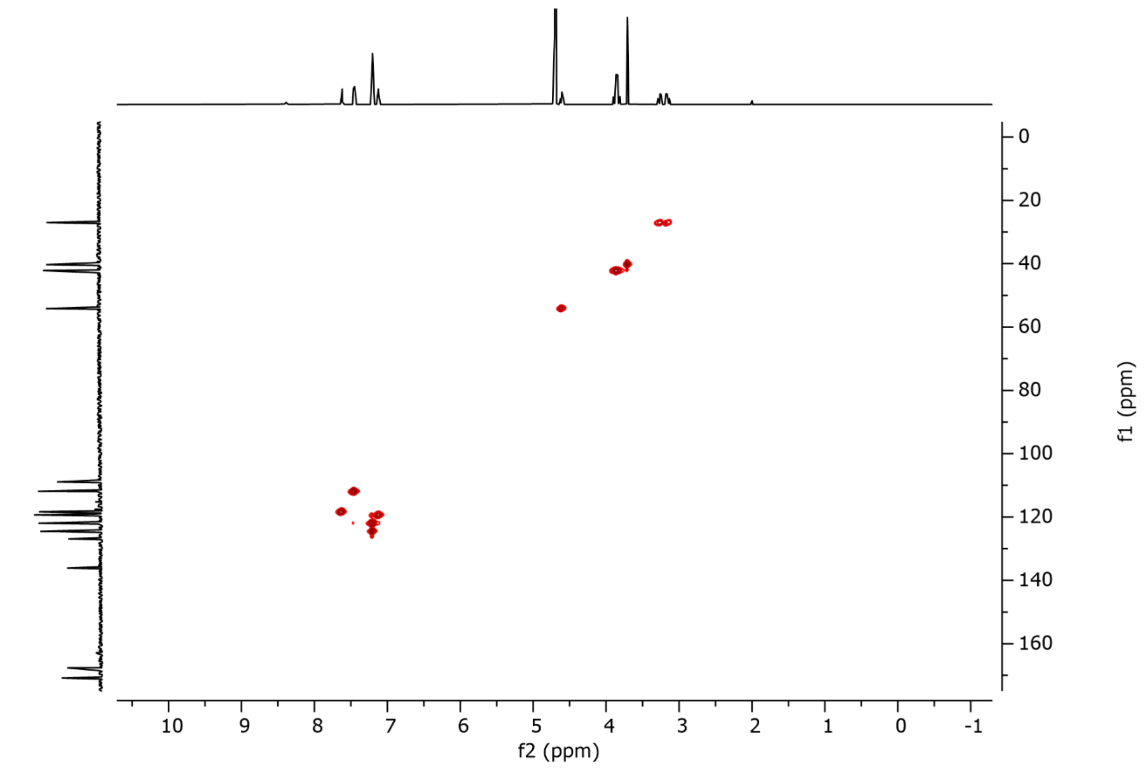

Figure S34. The HSQC NMR spectrum of compound **2** in D_2O.

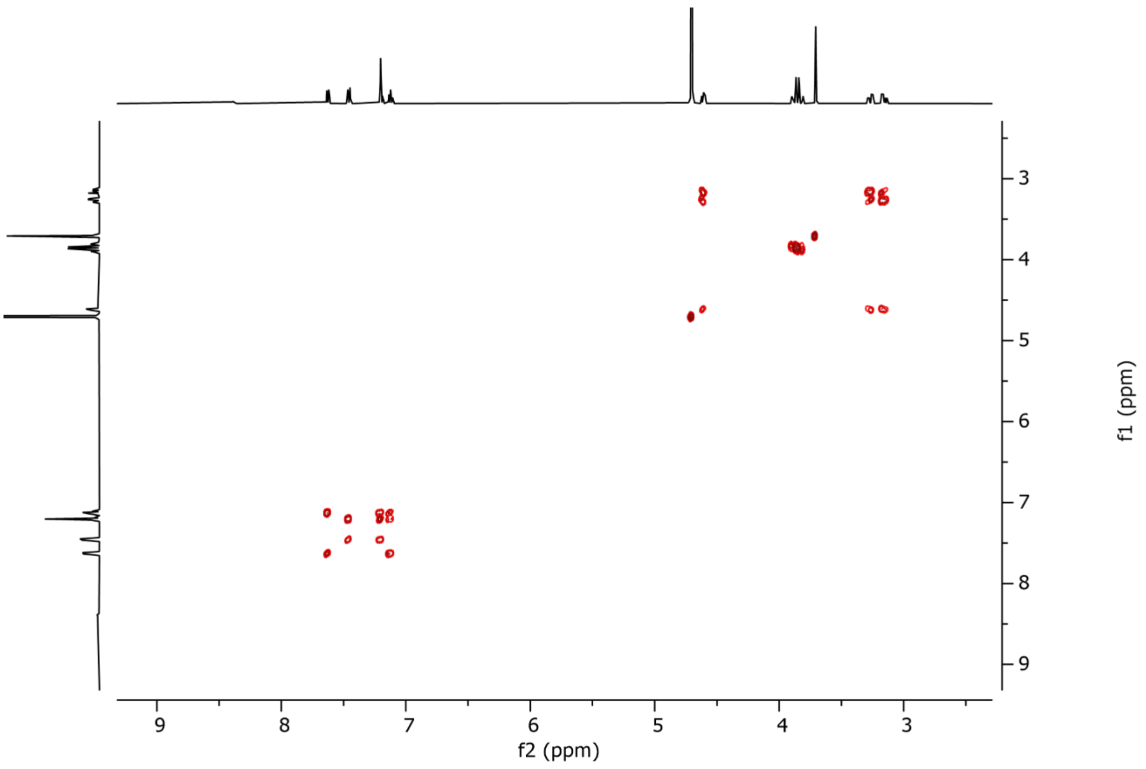

Figure S35. The ^1H-^1H COSY NMR spectrum of compound **2** in D_2O.

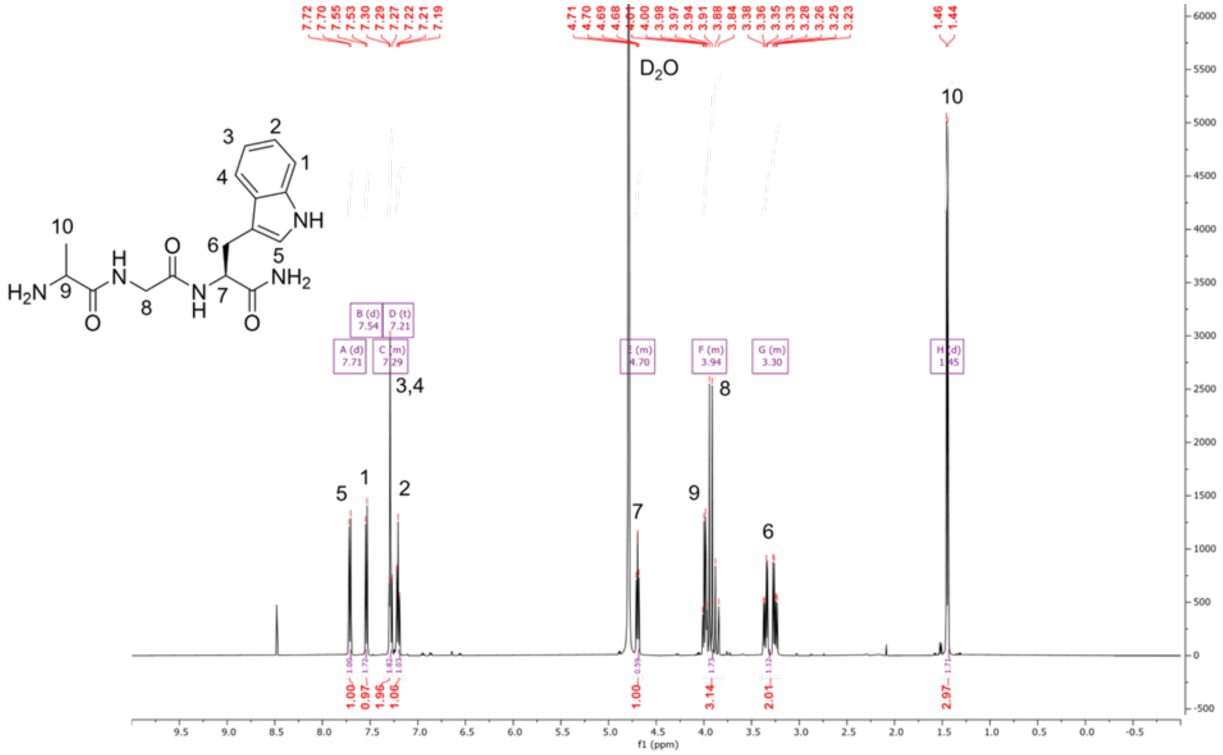

Figure S36. ^{1}H NMR (500 MHz, D_2O) for compound **3**.

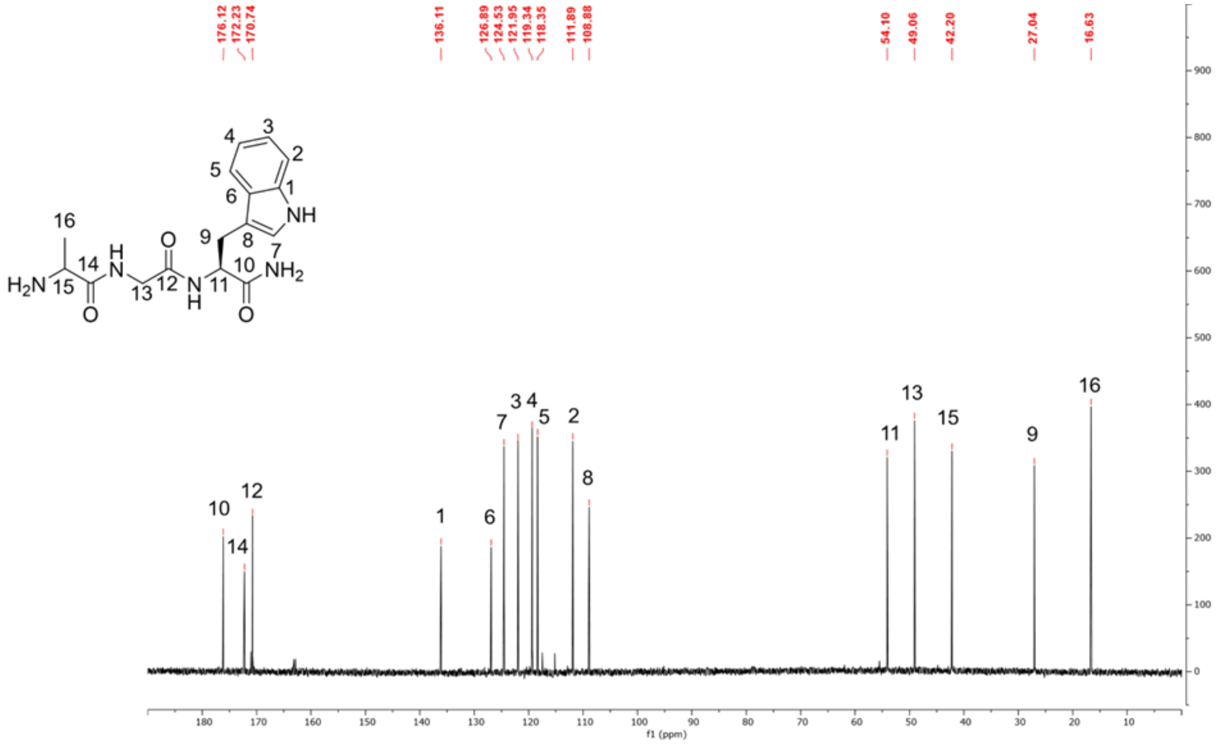

Figure S37. ^{13}C NMR (126 MHz, D_2O) for compound **3**.

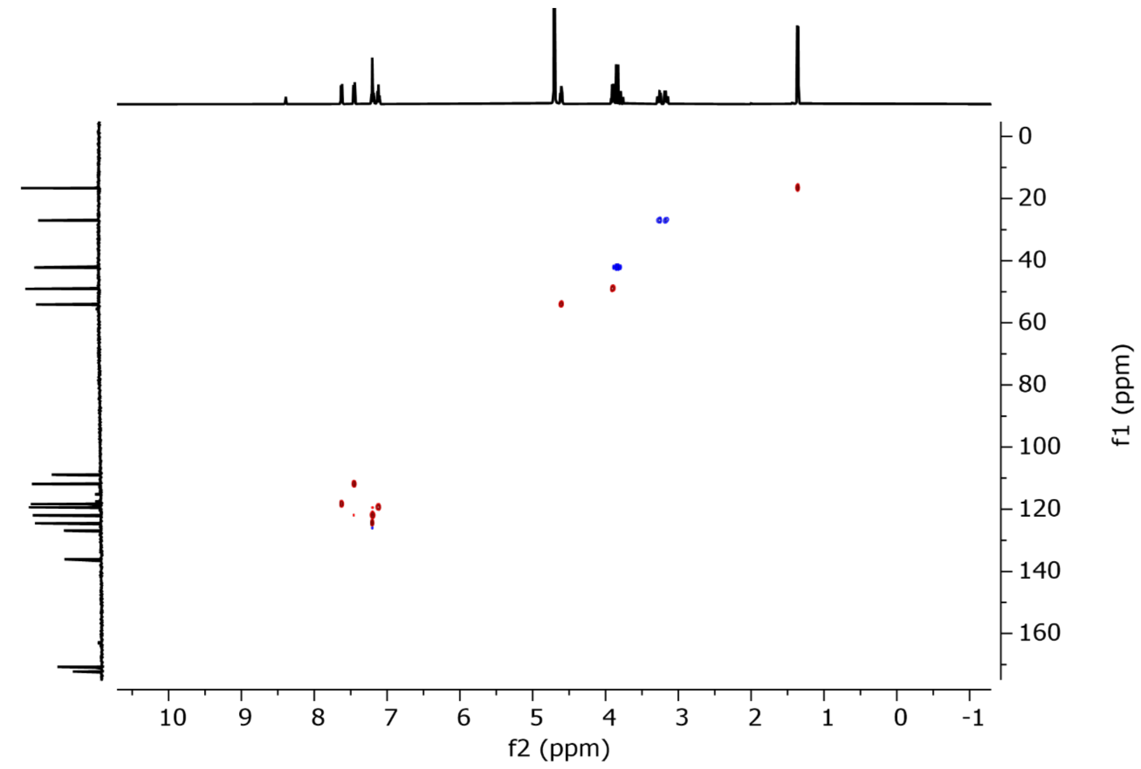

Figure S38. The HSQC NMR spectrum of compound **3** in D_2O.

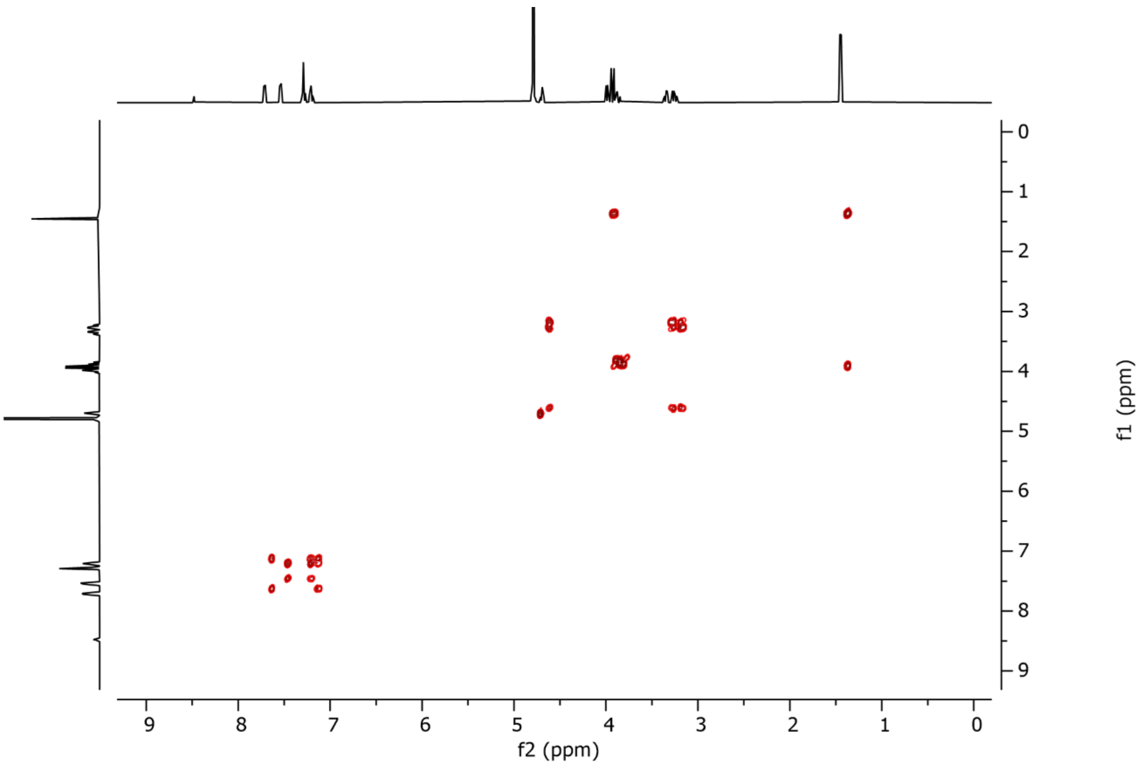

Figure S39. The ^{1}H-^{1}H COSY NMR spectrum of compound **3** in D_2O.

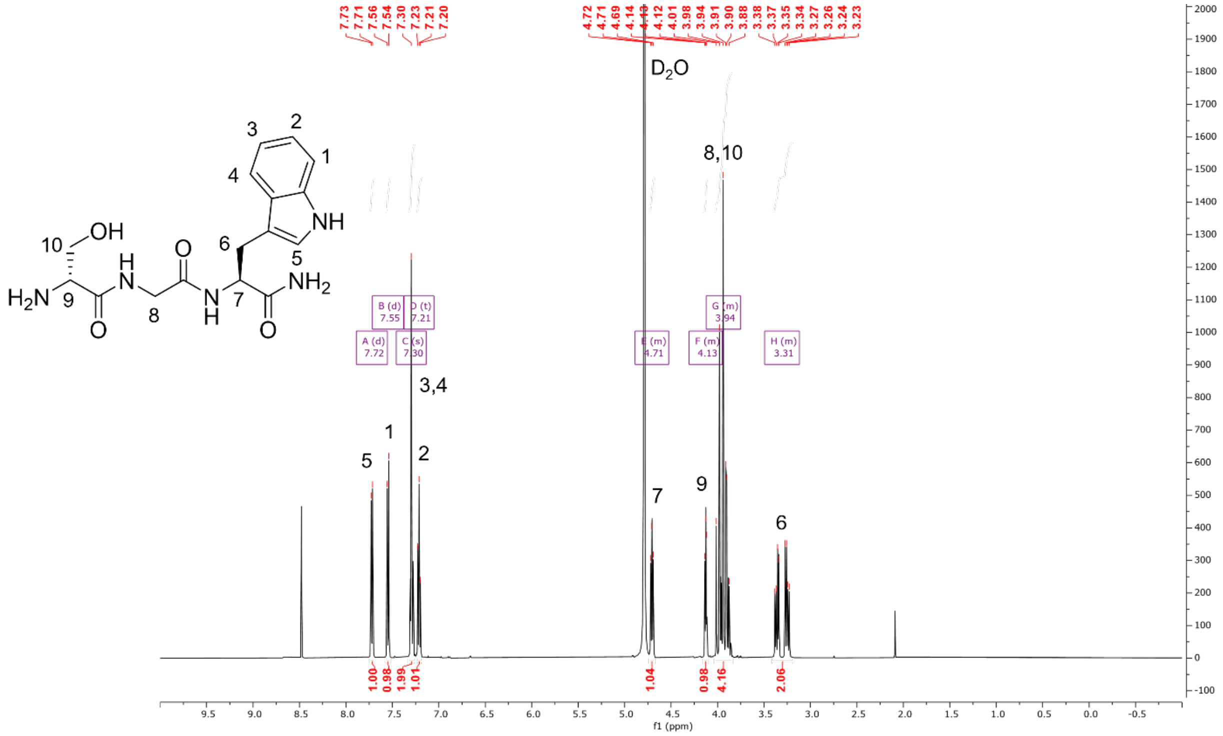

Figure S40. ^{1}H NMR (500 MHz, D_2O) for compound **4**.

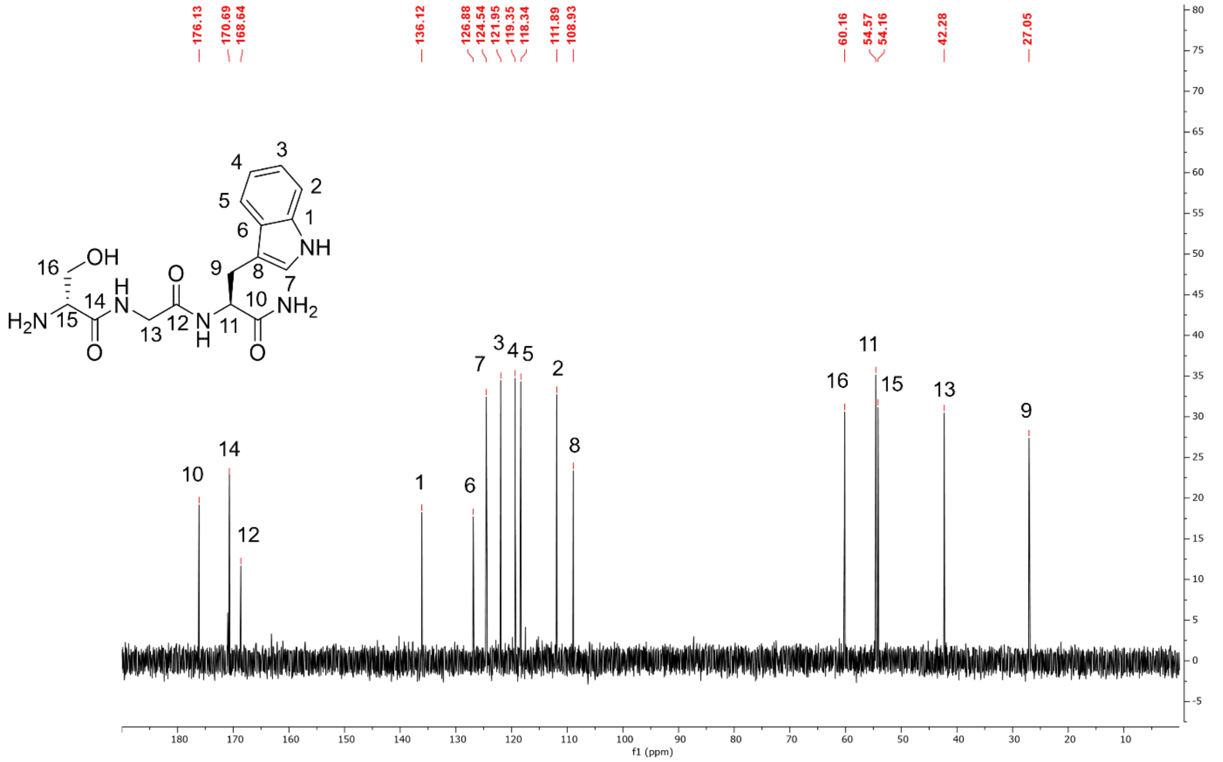

Figure S41. ^{13}C NMR (126 MHz, D_2O) for compound **4**.

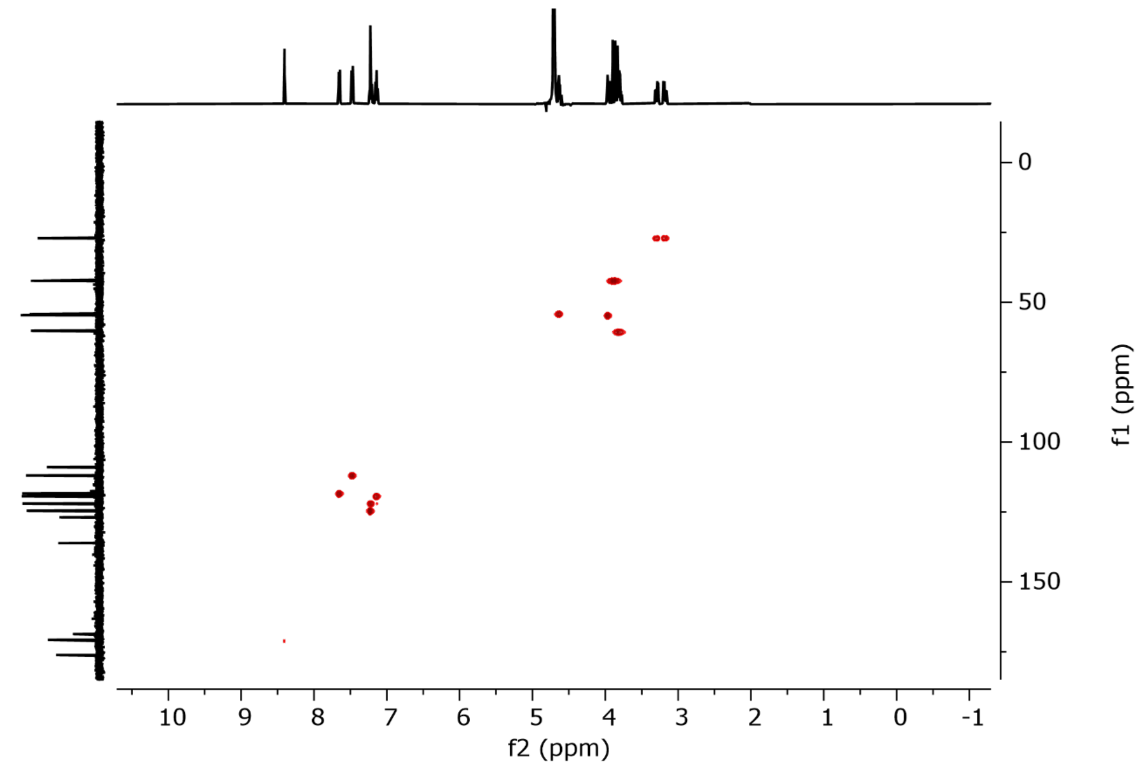

Figure S42. The HSQC NMR spectrum of compound **4** in D_2O.

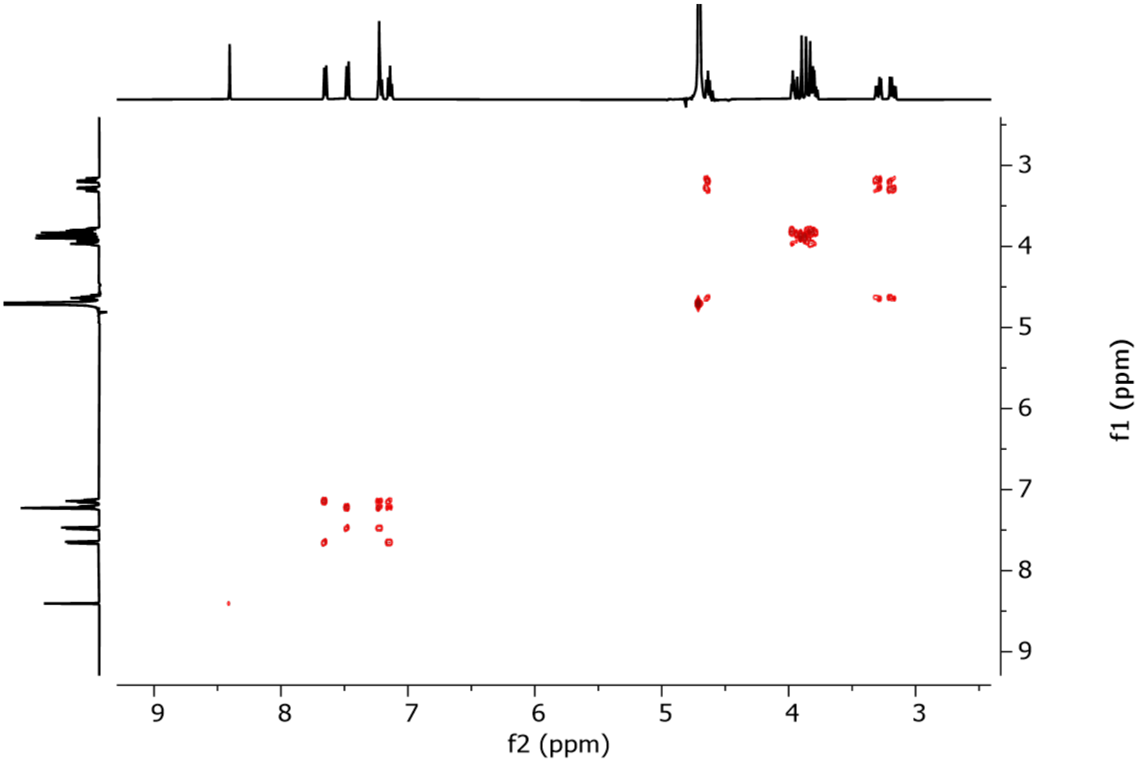

Figure S43. The ^1H-^1H COSY NMR spectrum of compound **4** in D_2O.

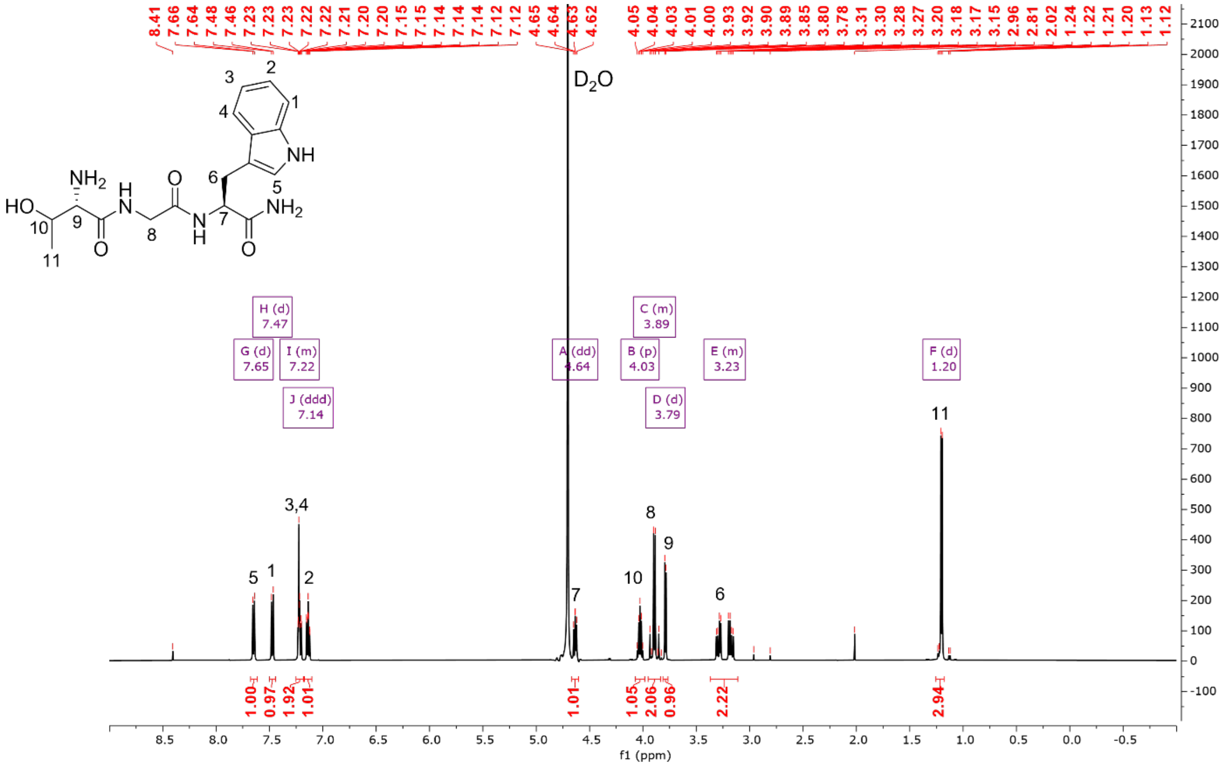

Figure S44. ^{1}H NMR (500 MHz, D_2O) for compound **5**.

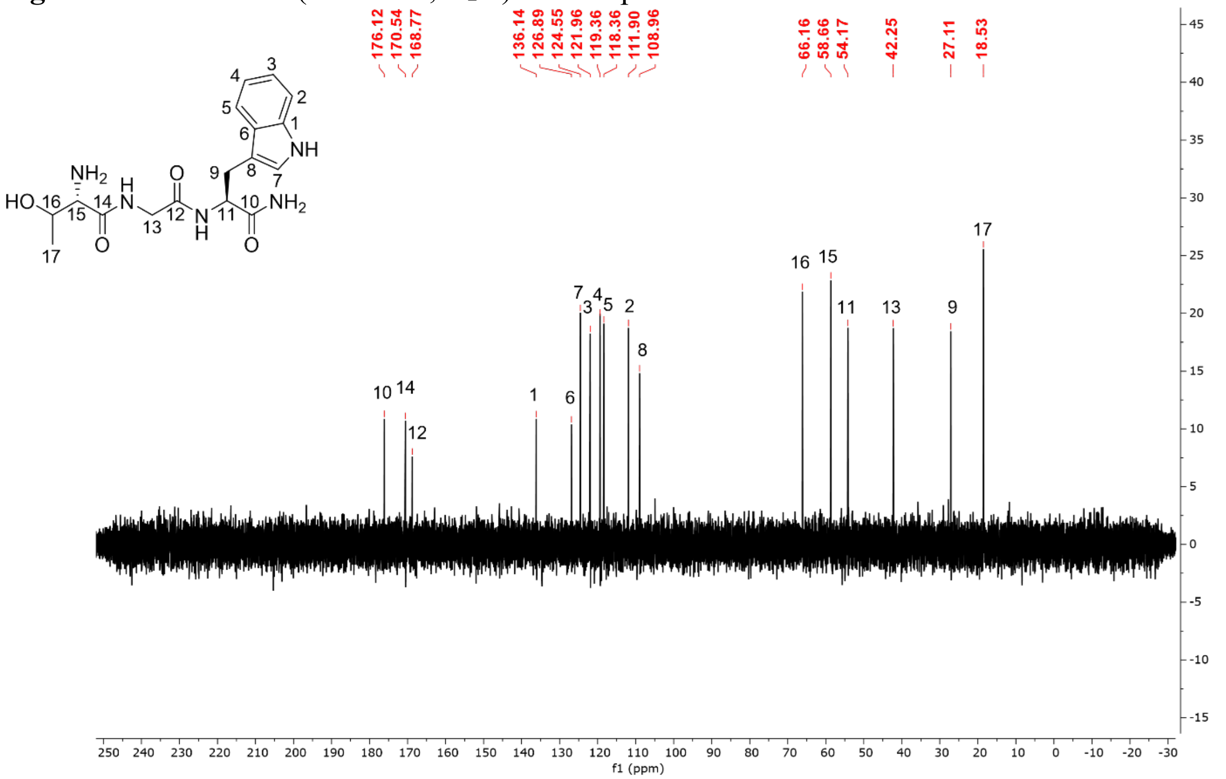

Figure S45. ^{13}C NMR (126 MHz, D_2O) for compound **5**.

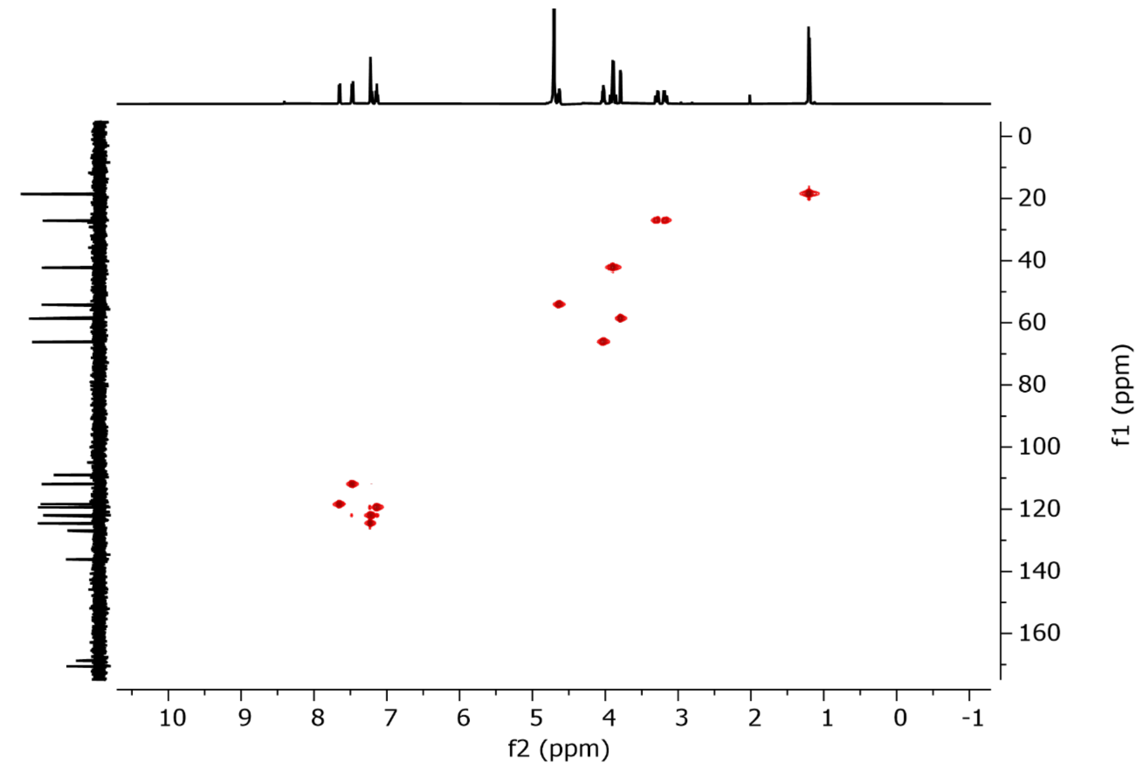

Figure S46. The HSQC NMR spectrum of compound **5** in D_2O.

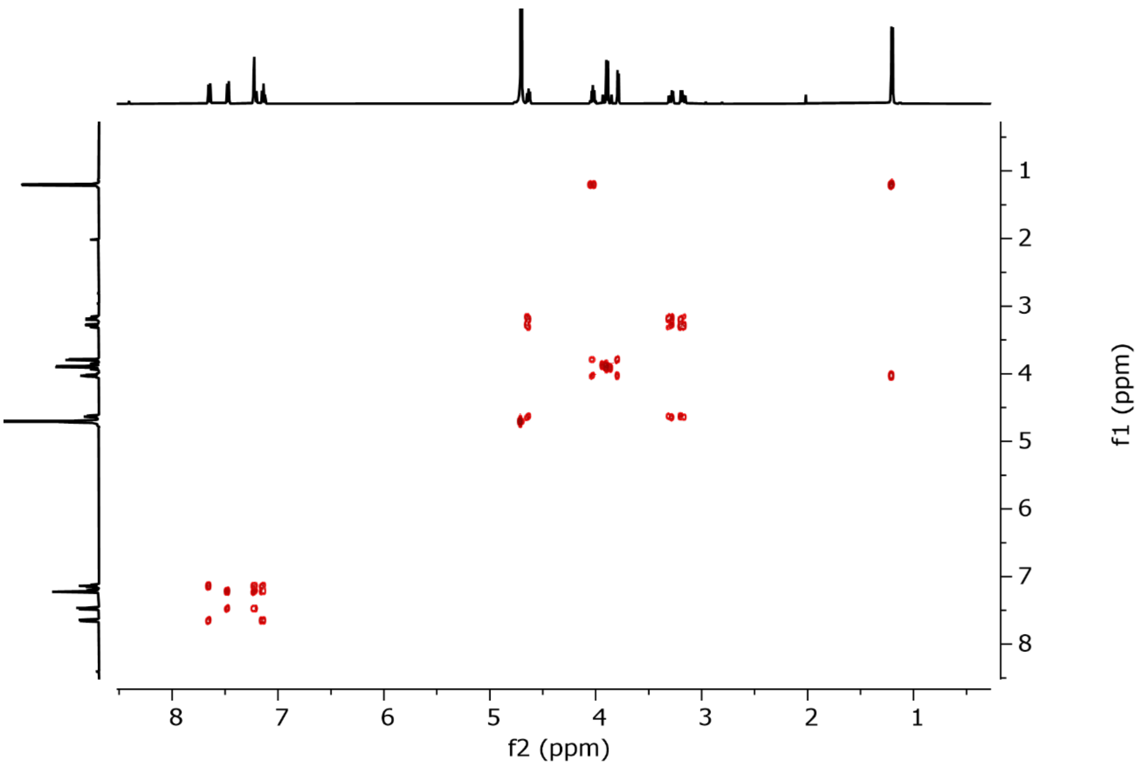

Figure S47. The ^1H-^1H COSY NMR spectrum of compound **5** in D_2O.

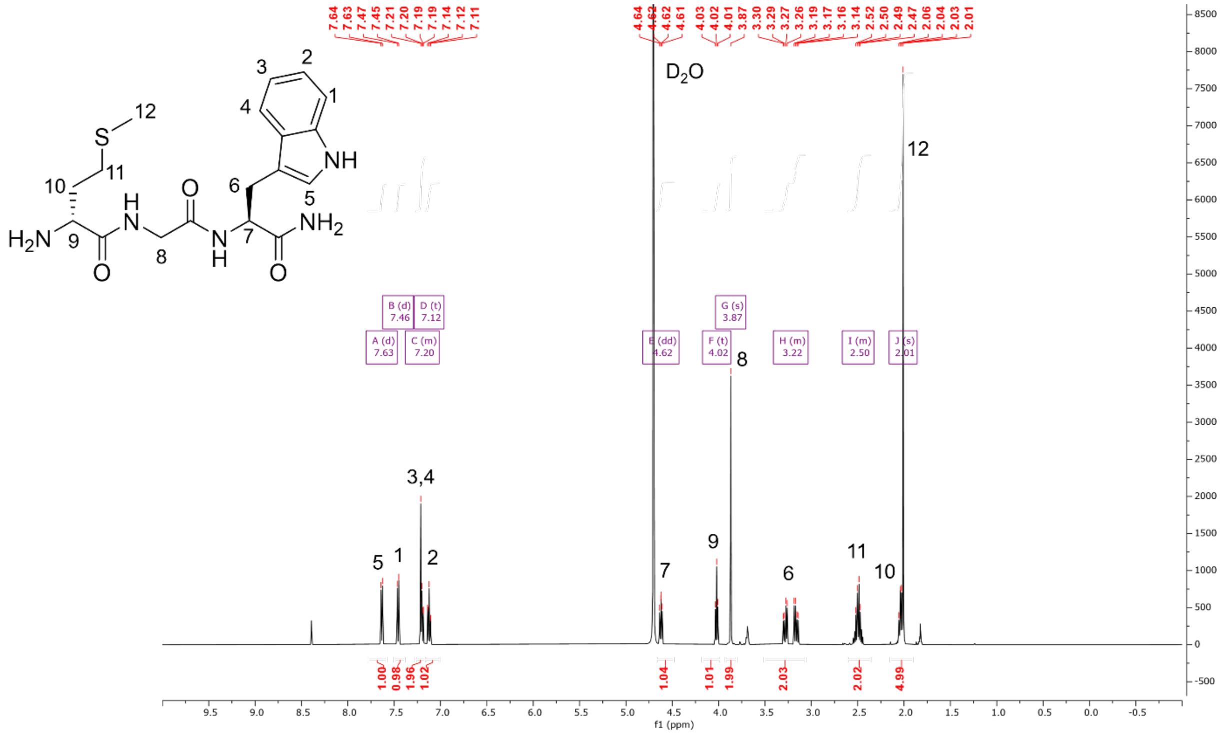

Figure S48. ^{1}H NMR (500 MHz, D_2O) for compound **6**.

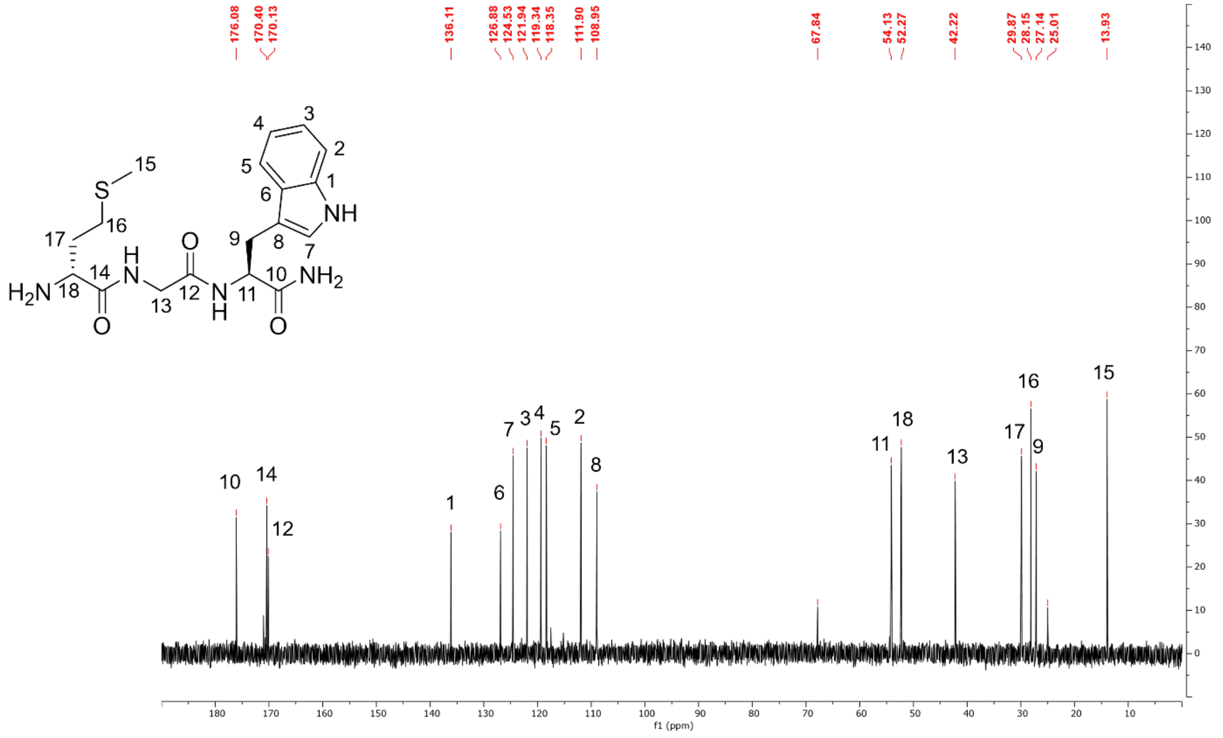

Figure S49. ^{13}C NMR (126 MHz, D_2O) for compound **6**.

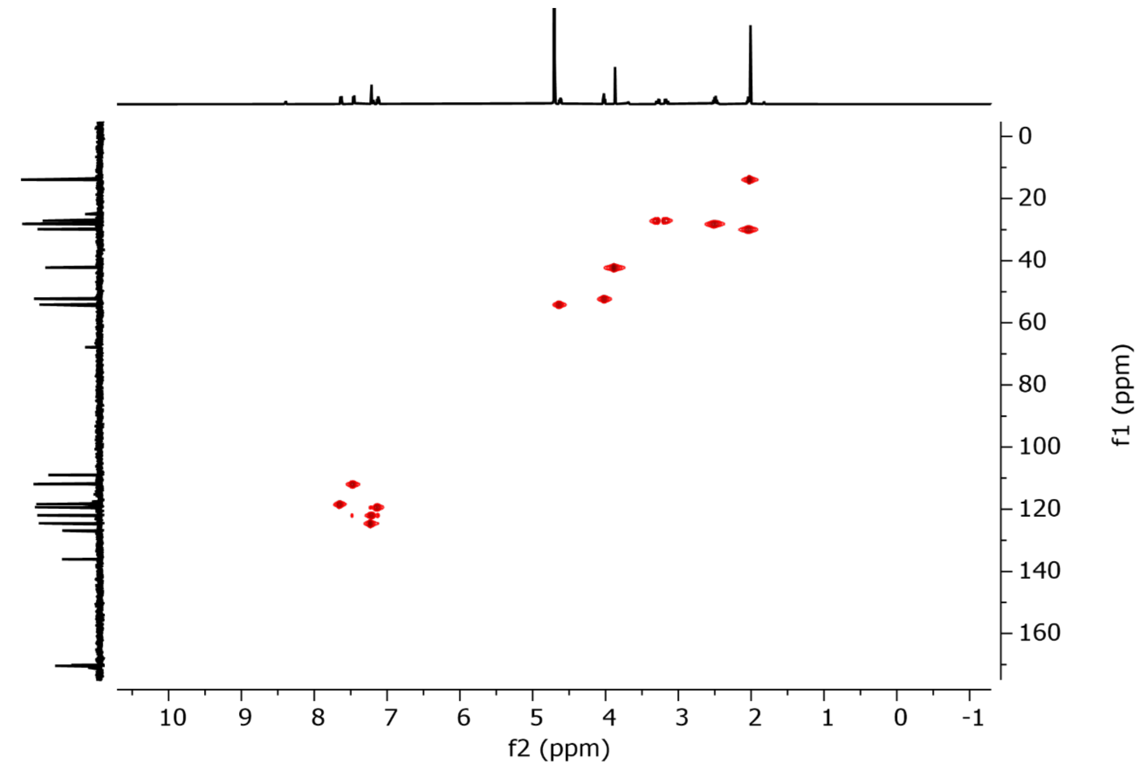

Figure S50. The HSQC NMR spectrum of compound **6** in D_2O.

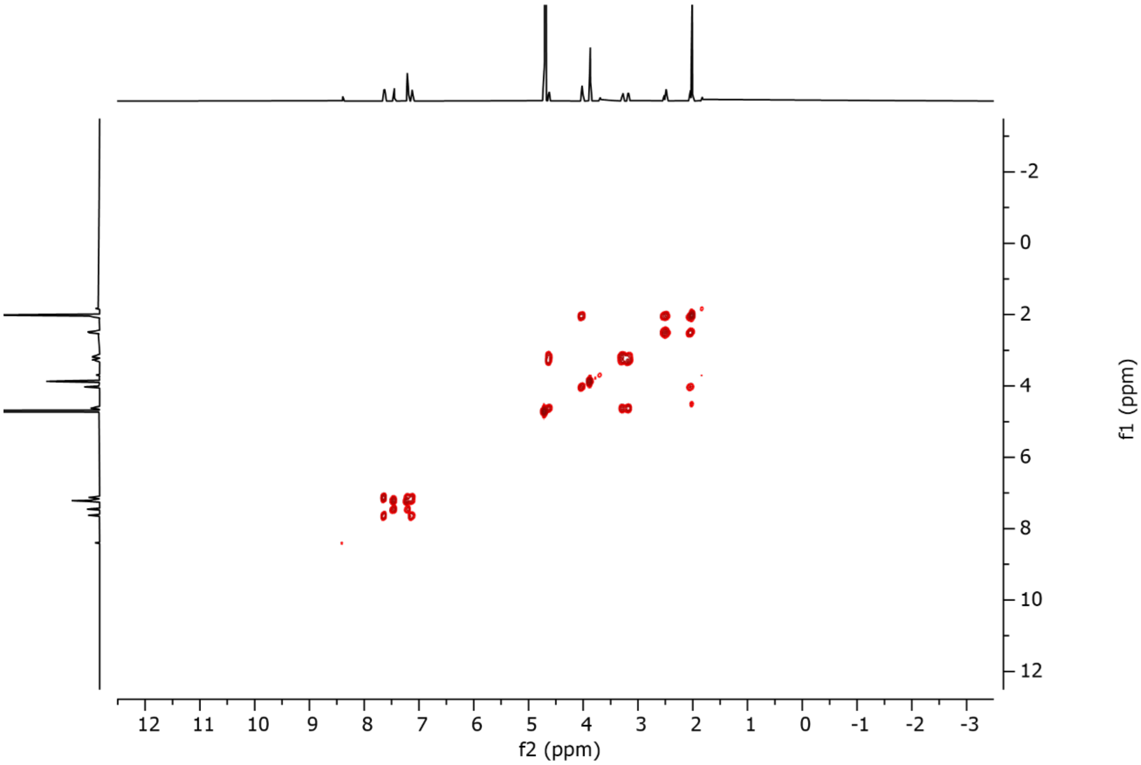

Figure S51. The ^1H-^1H COSY NMR spectrum of compound **6** in D_2O.

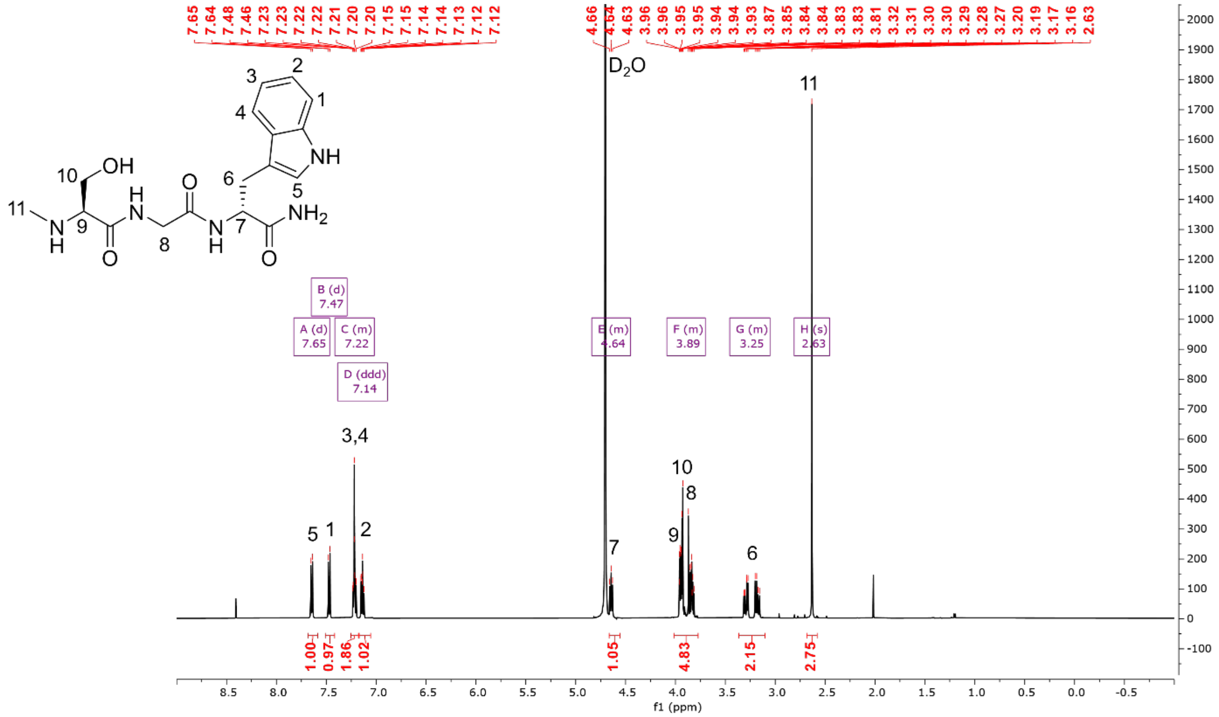

Figure S52. ^{1}H NMR (500 MHz, D_2O) for compound **7**.

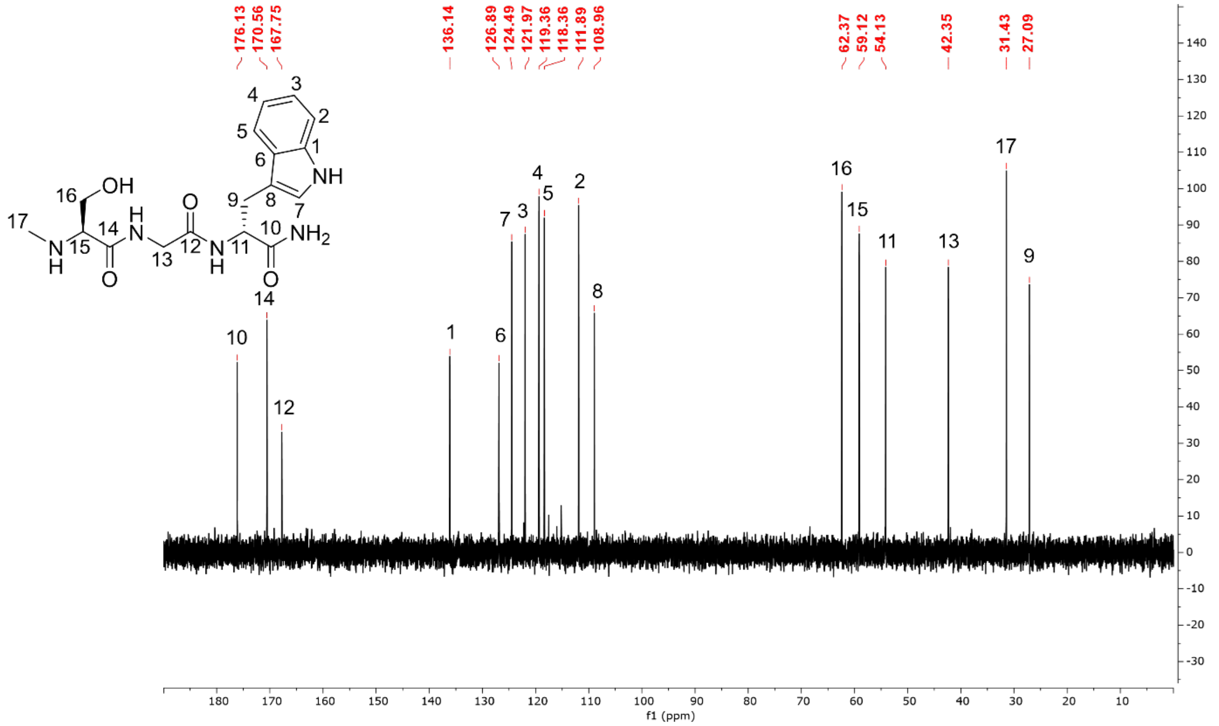

Figure S53. ^{13}C NMR (126 MHz, D_2O) for compound **7**.

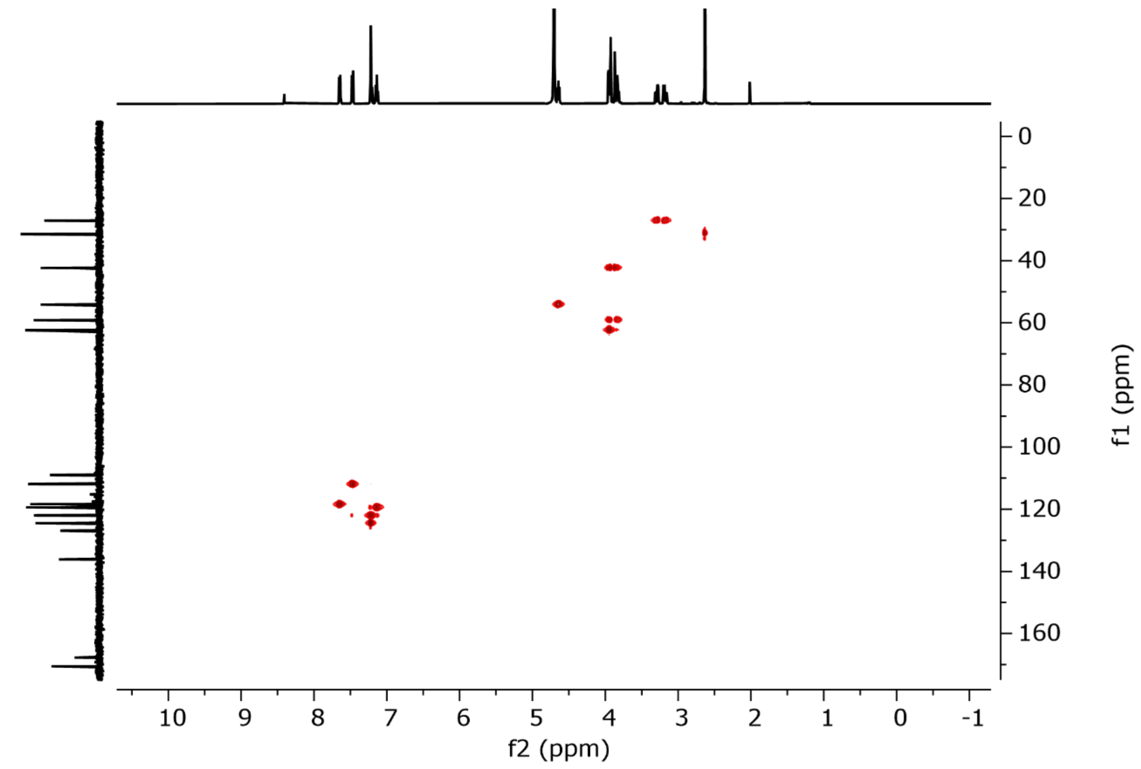

Figure S54. The HSQC NMR spectrum of compound **7** in D_2O.

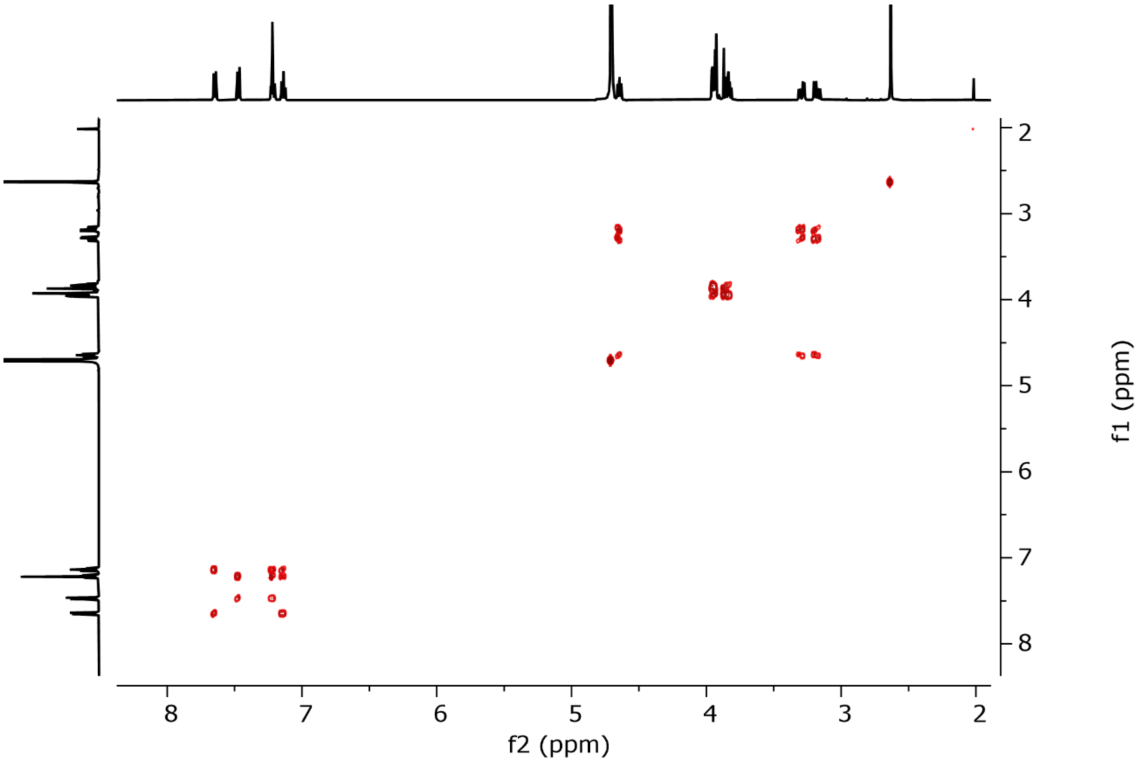

Figure S55. The ^1H-^1H COSY NMR spectrum of compound **7** in D_2O.

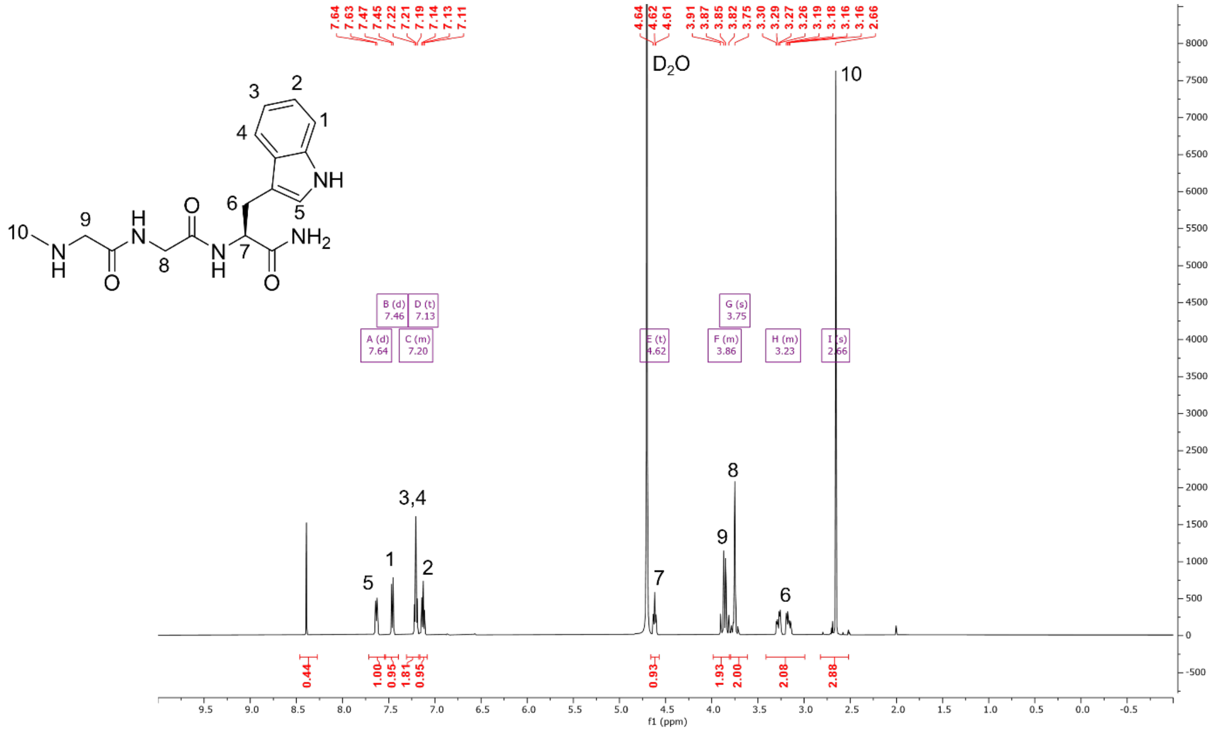

Figure S56. ^{1}H NMR (500 MHz, D_2O) for compound **8**.

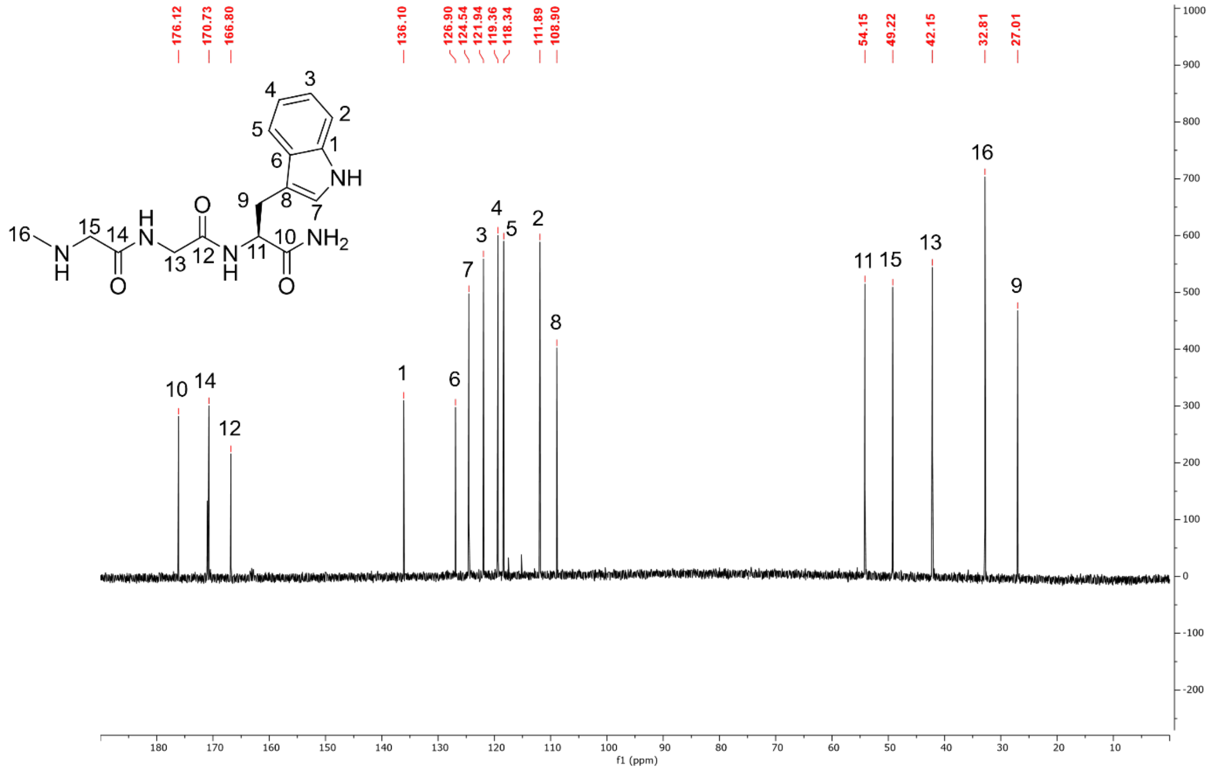

Figure S57. ^{13}C NMR (126 MHz, D_2O) for compound **8**.

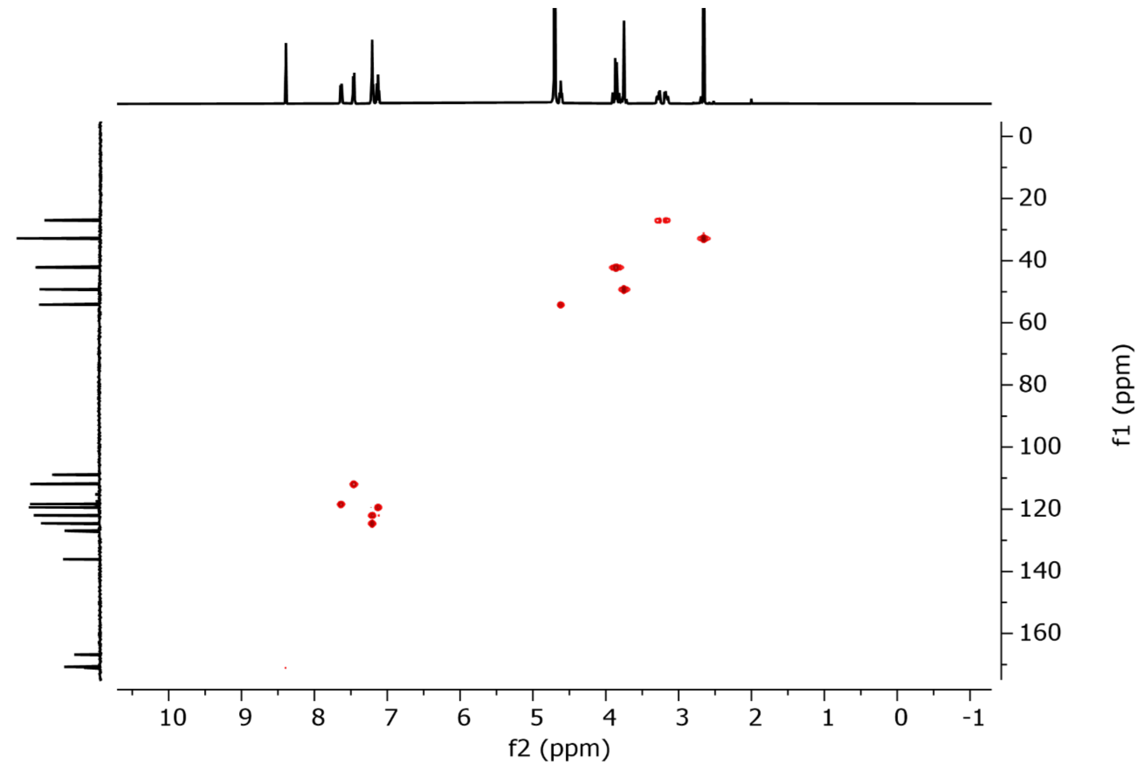

Figure S58. The HSQC NMR spectrum of compound **8** in D_2O.

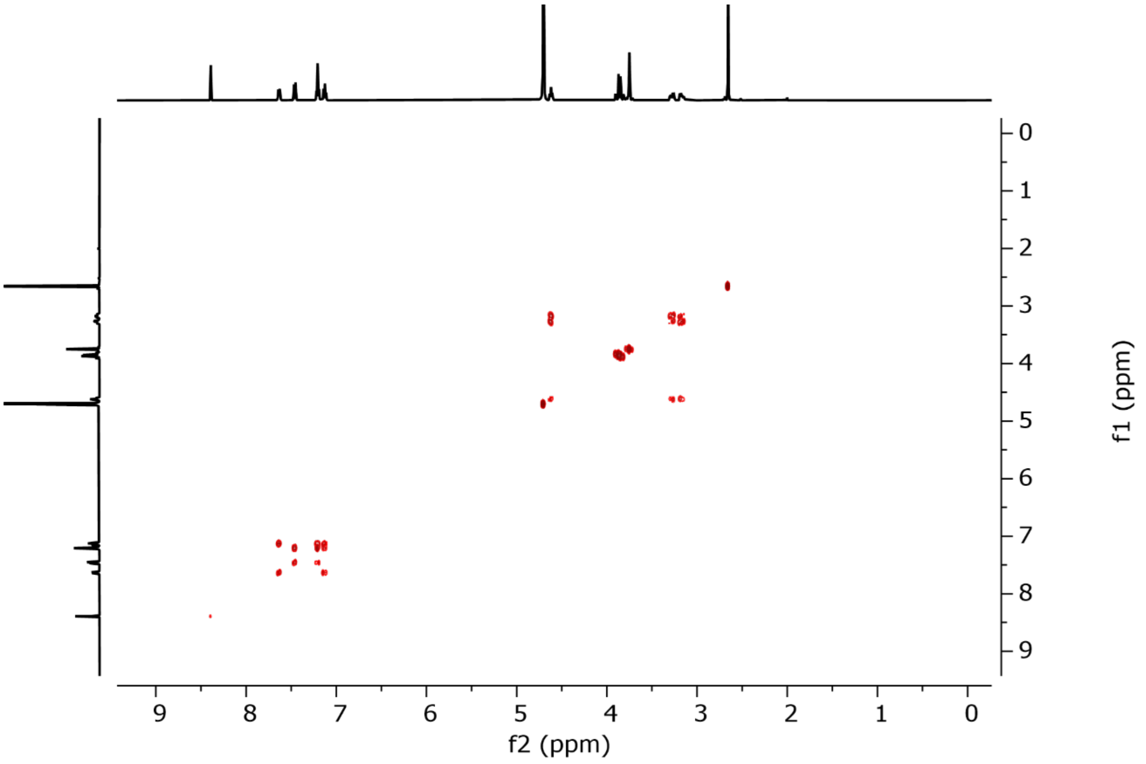

Figure S59. The ^{1}H-^{1}H COSY NMR spectrum of compound **8** in D_2O.

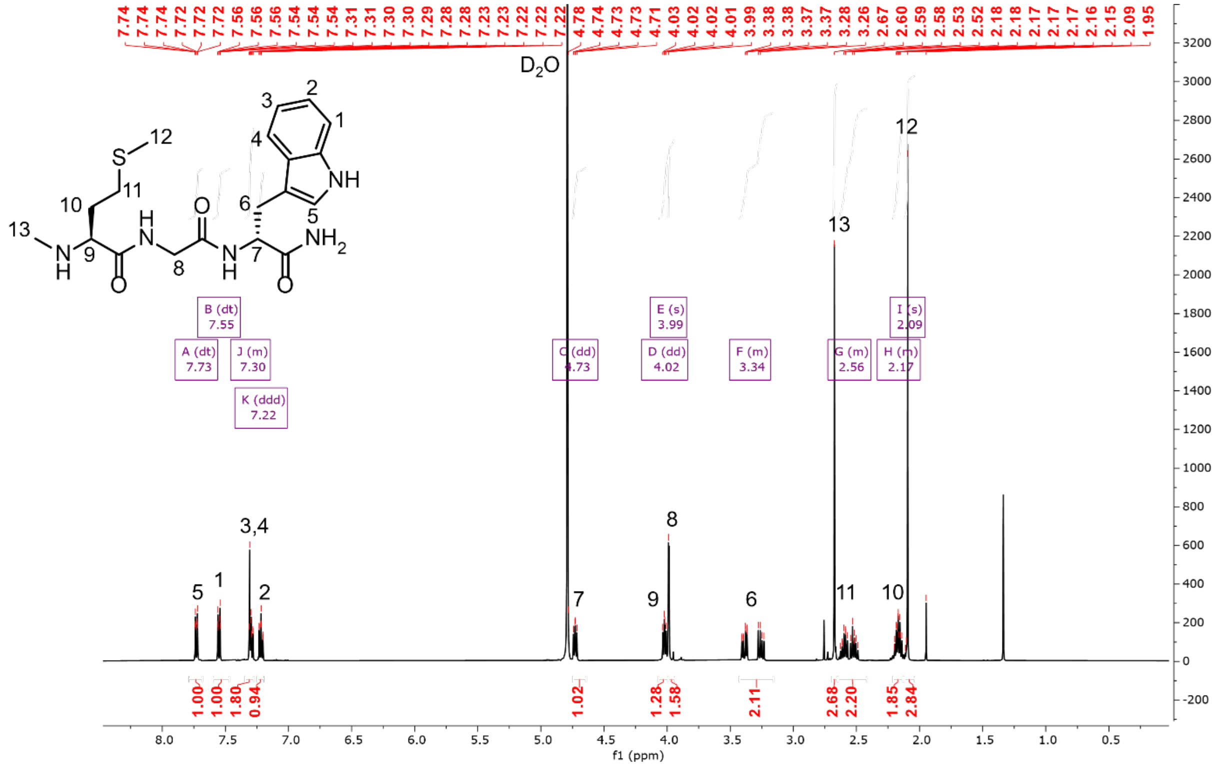

Figure S60. ^{1}H NMR (500 MHz, D_2O) for compound **9**.

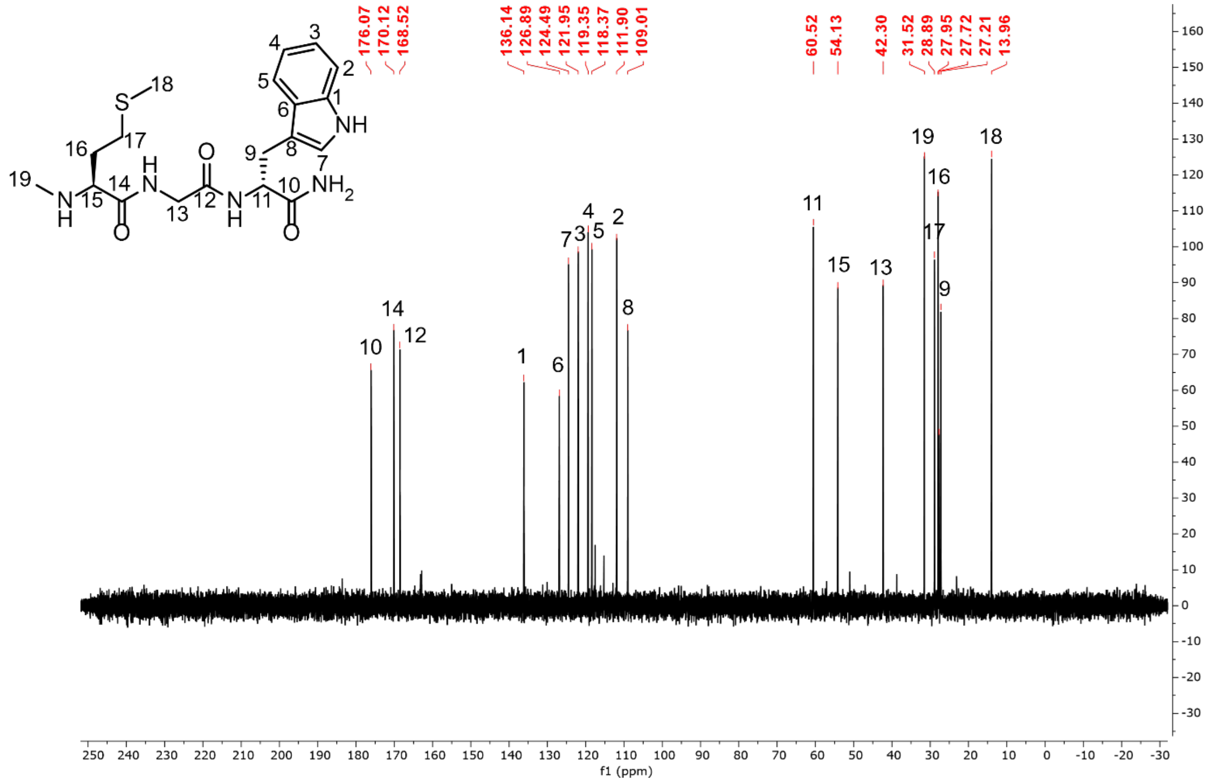

Figure S61. ^{13}C NMR (126 MHz, D_2O) for compound **9**.

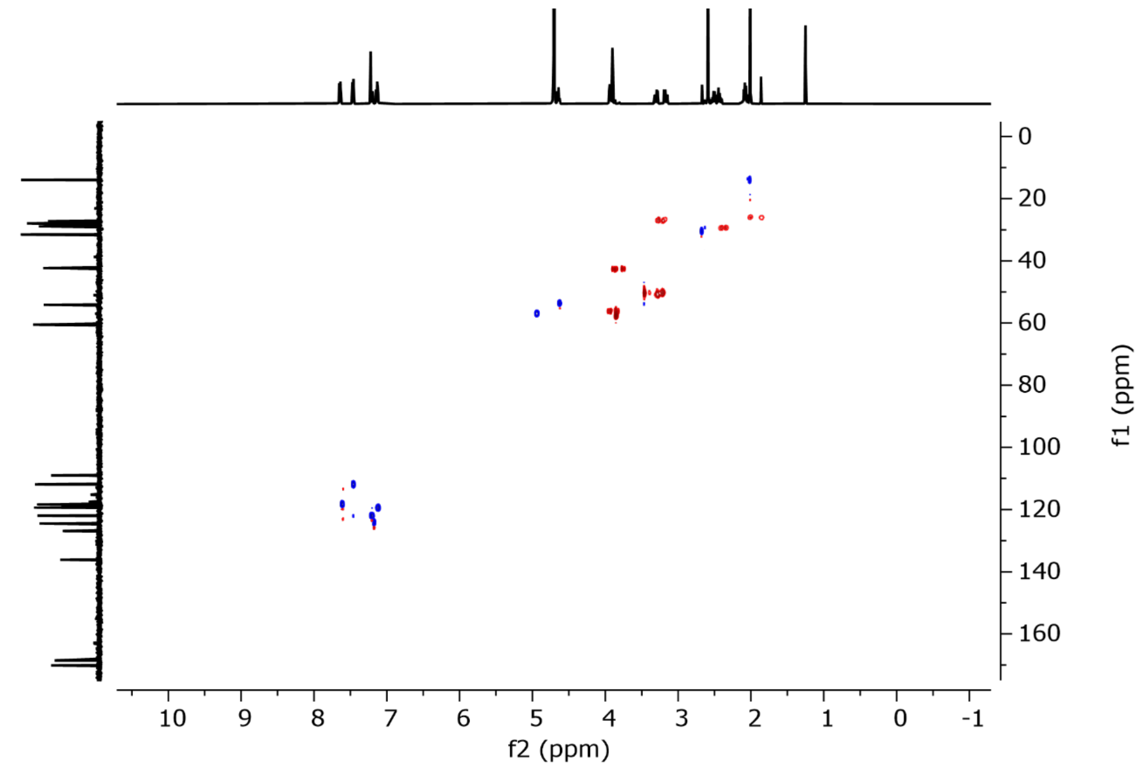

Figure S62. The HSQC NMR spectrum of compound **9** in D_2O.

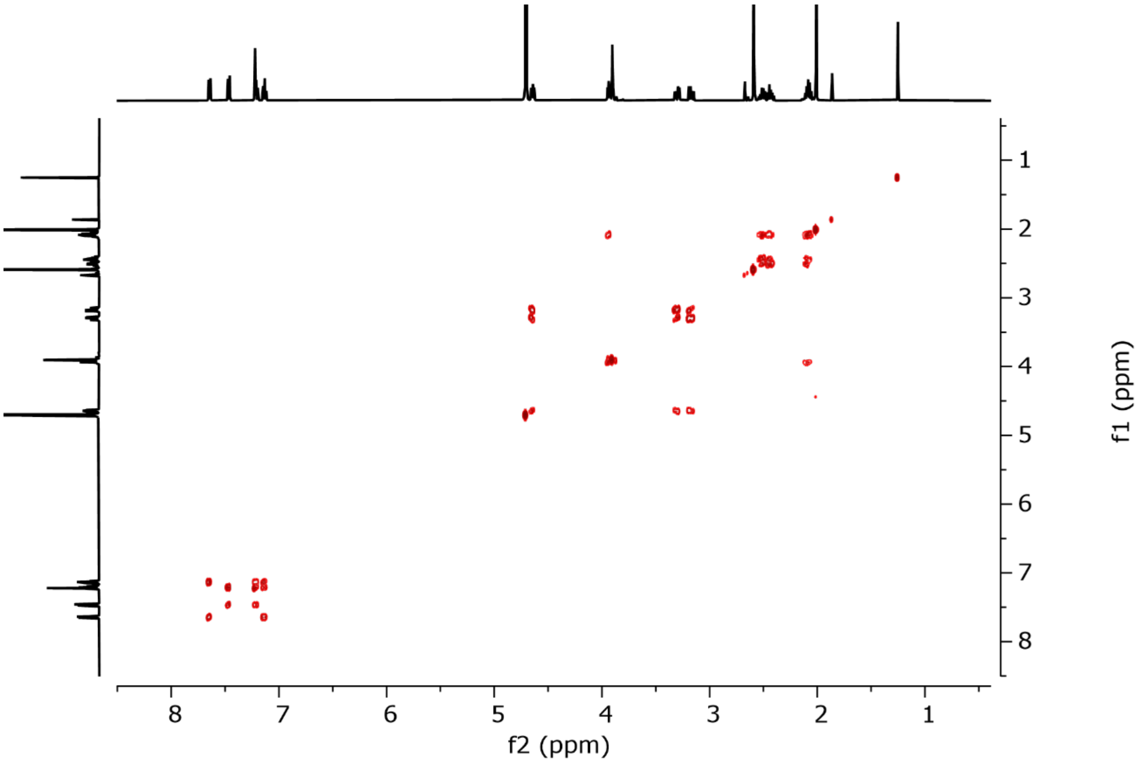

Figure S63. The ^1H-^1H COSY NMR spectrum of compound **9** in D_2O.

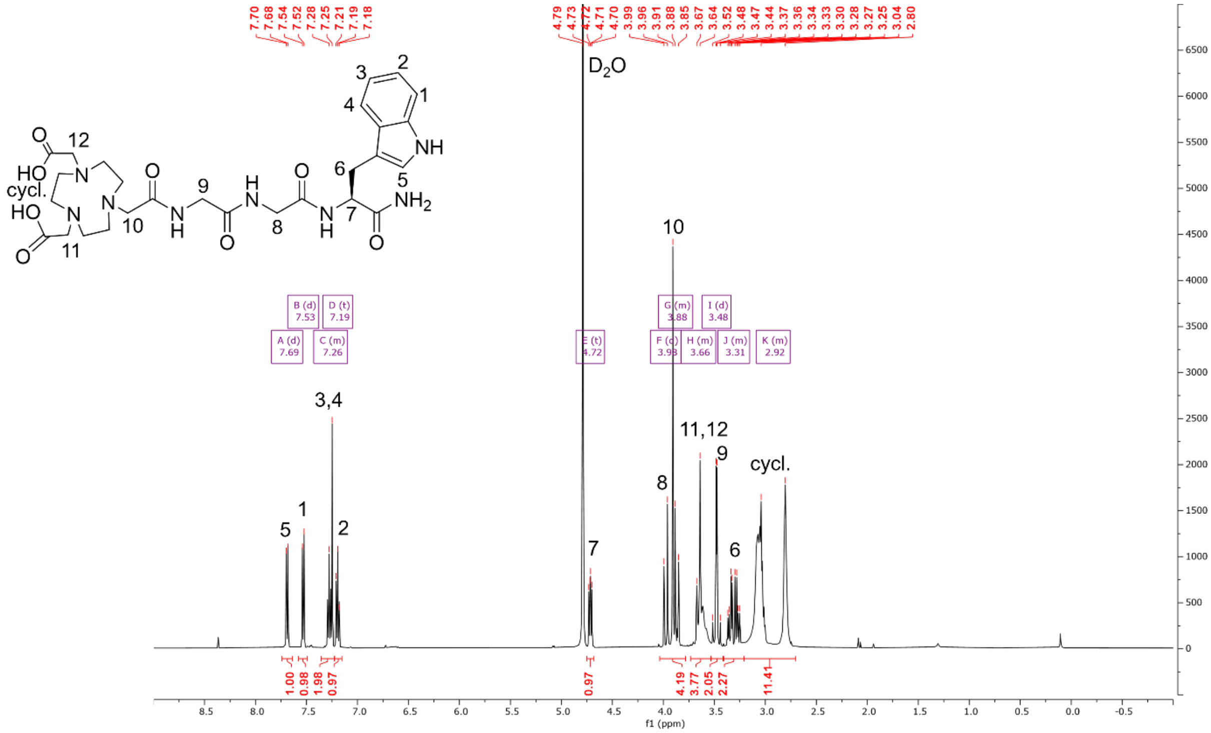

Figure S64. ^{1}H NMR (500 MHz, D_2O) for compound **10**.

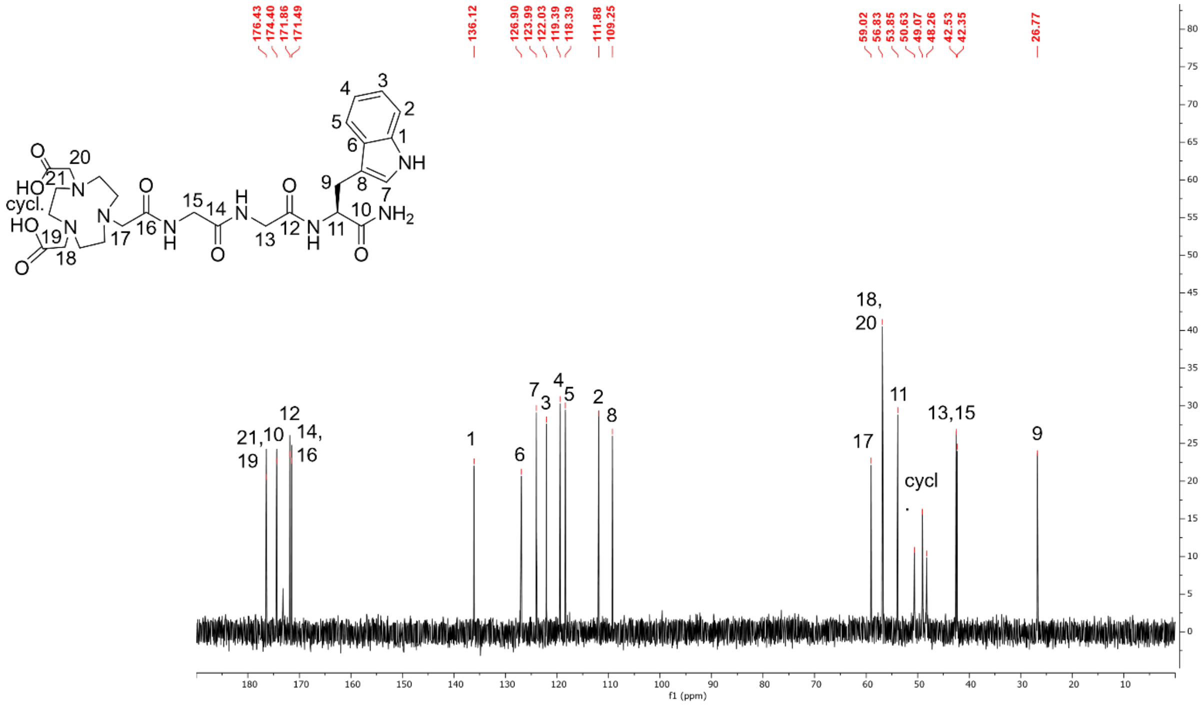

Figure S65. ^{13}C NMR (126 MHz, D_2O) for compound **10**.

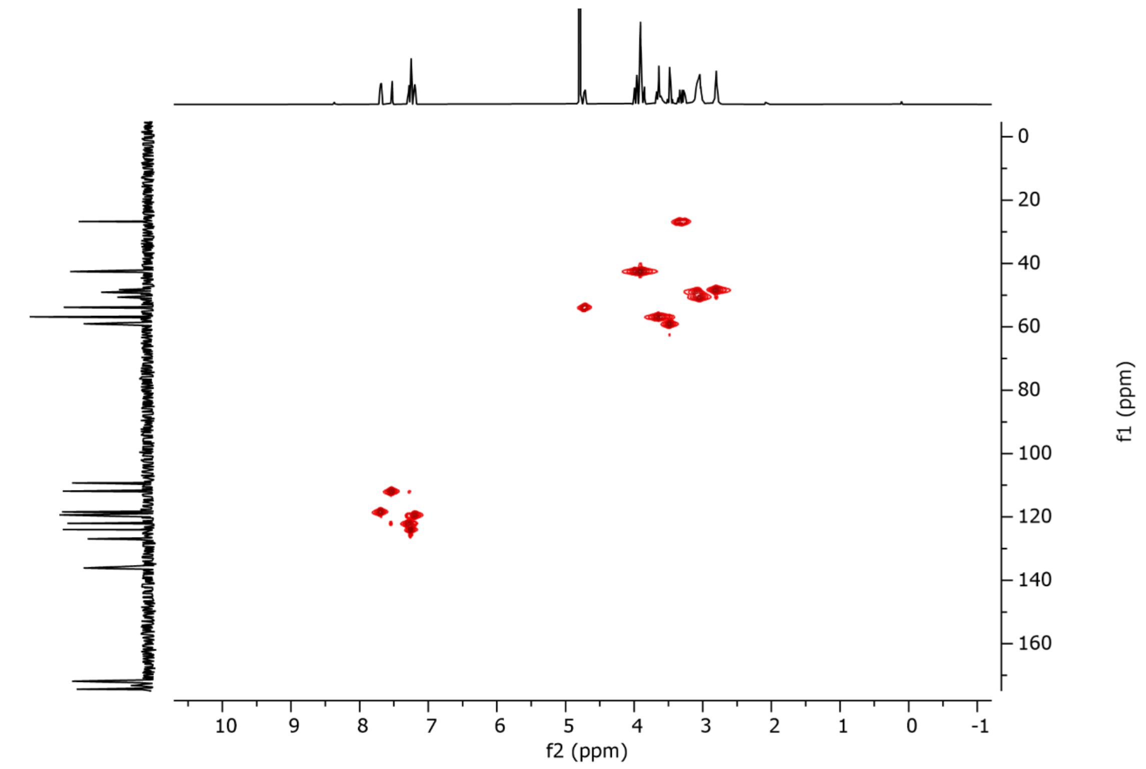

Figure S66. The HSQC NMR spectrum of compound **10** in D_2O.

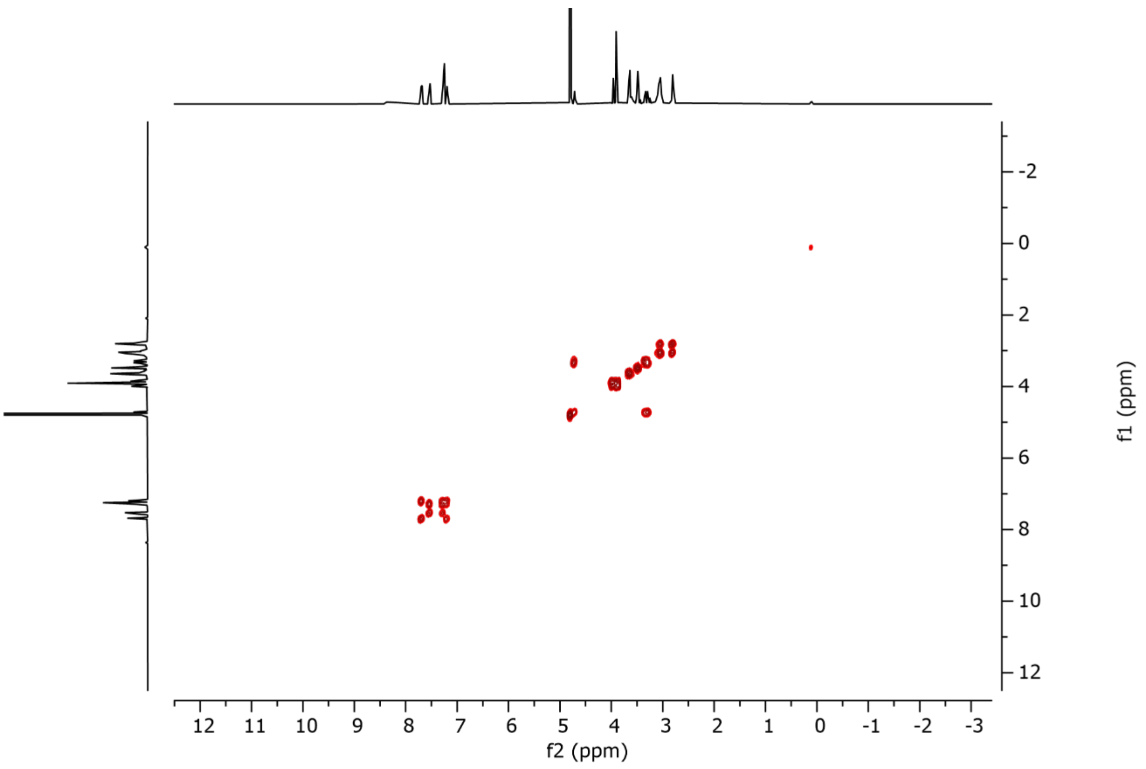

Figure S67. The ^{1}H-^{1}H COSY NMR spectrum of compound **10** in D_2O.

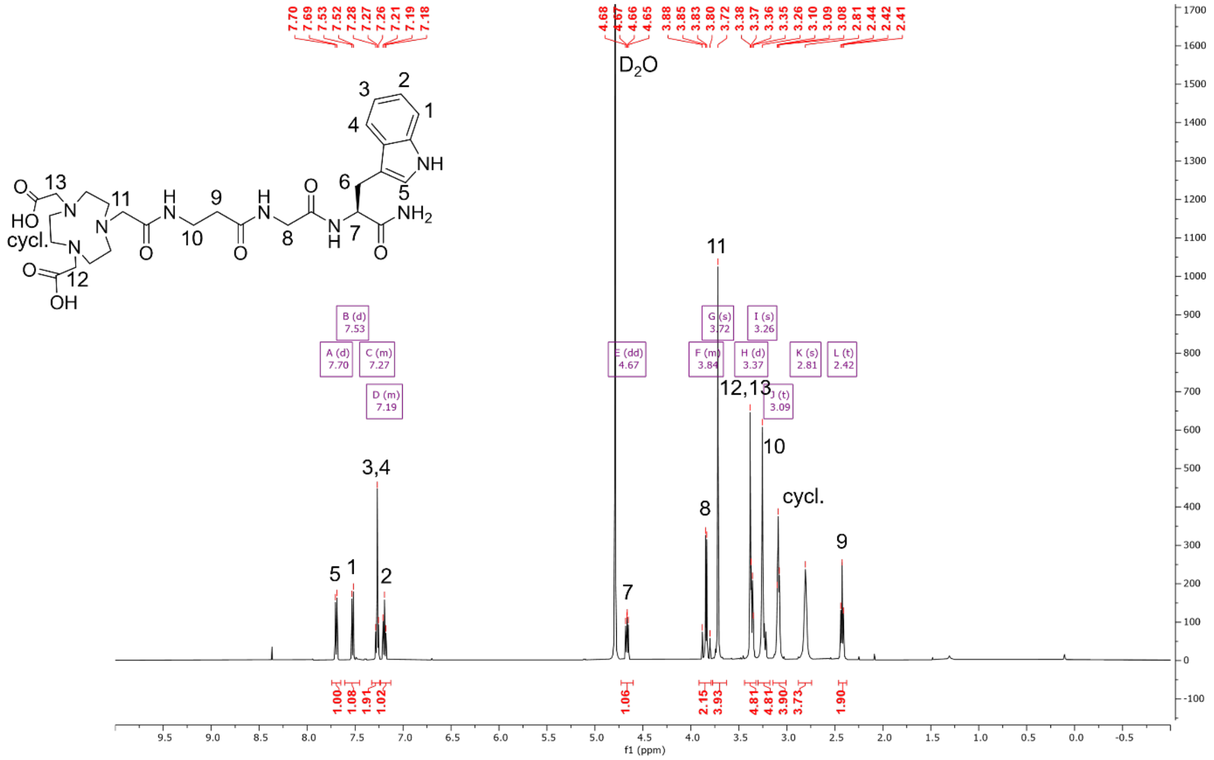

Figure S68. ^{1}H NMR (500 MHz, D_2O) for compound **11**.

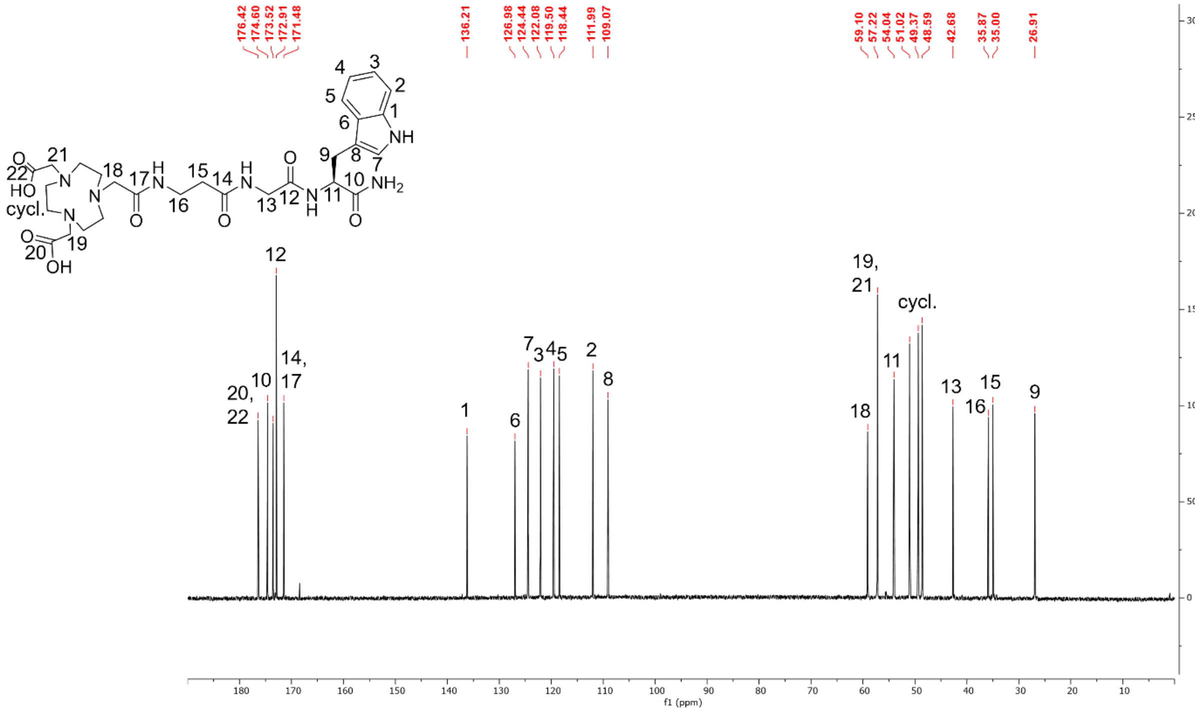

Figure S69. ^{13}C NMR (126 MHz, D_2O) for compound **11**.

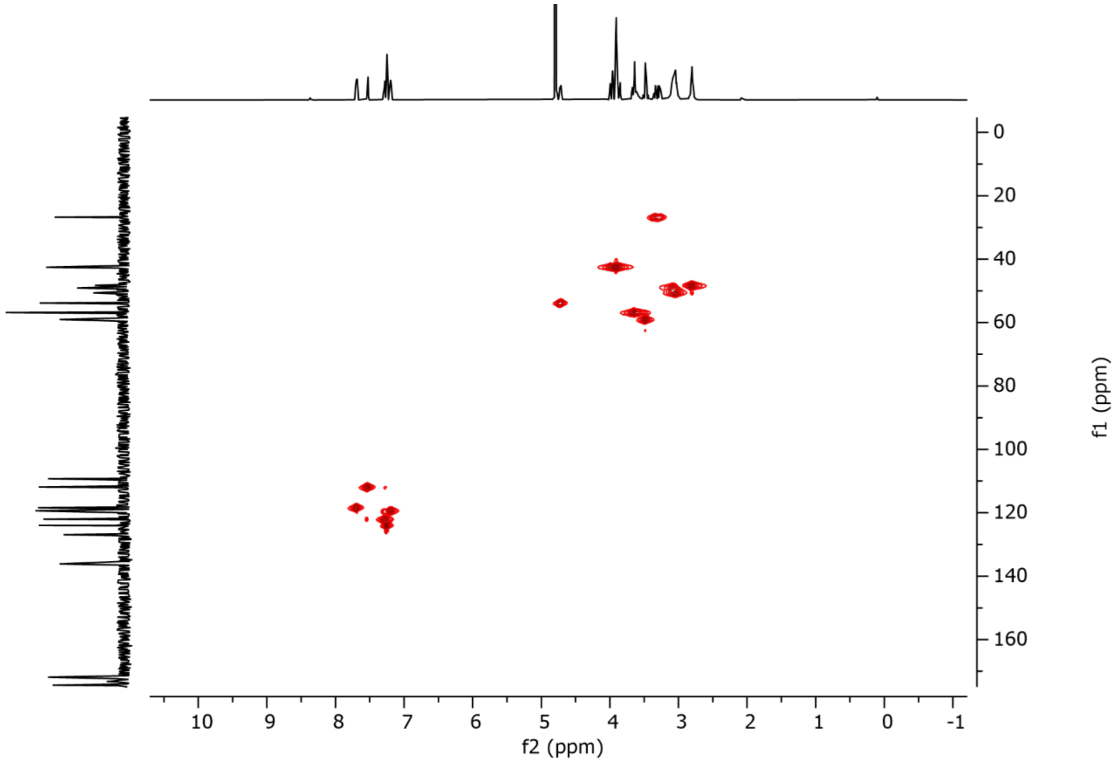

Figure S70. The HSQC NMR spectrum of compound **11** in D2O.

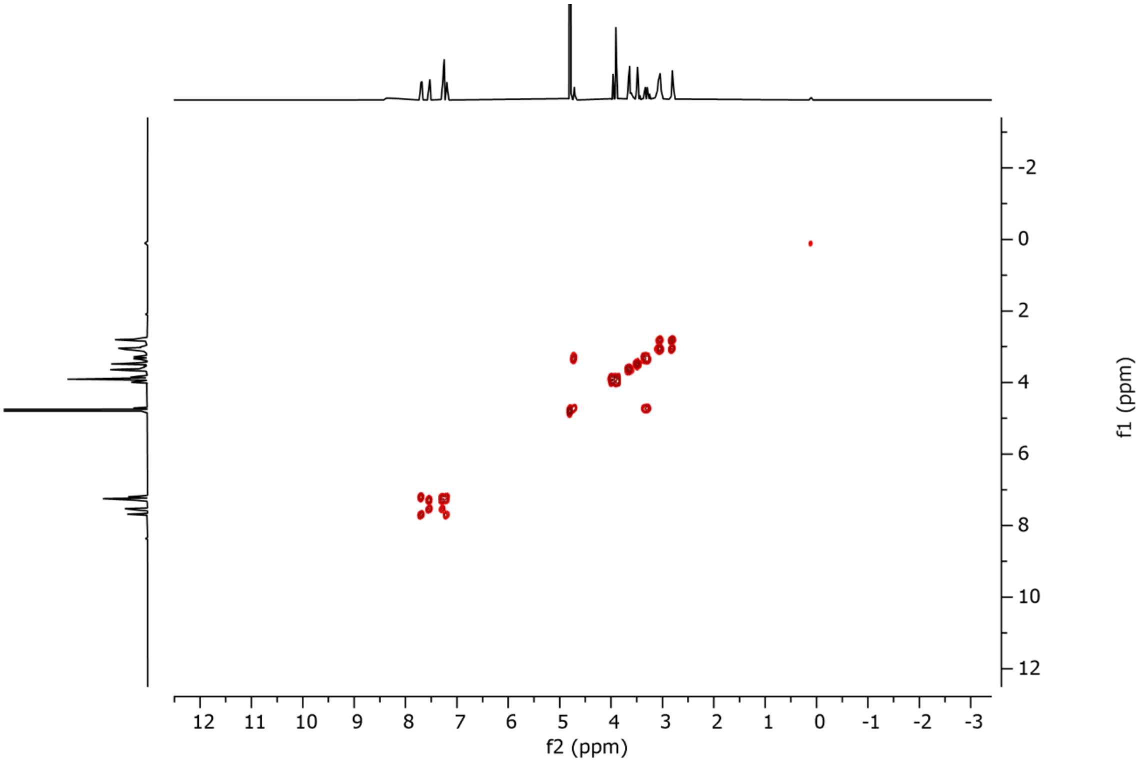

Figure S71. The ^{1}H-^{1}H COSY NMR of compound **11** in D_2O.

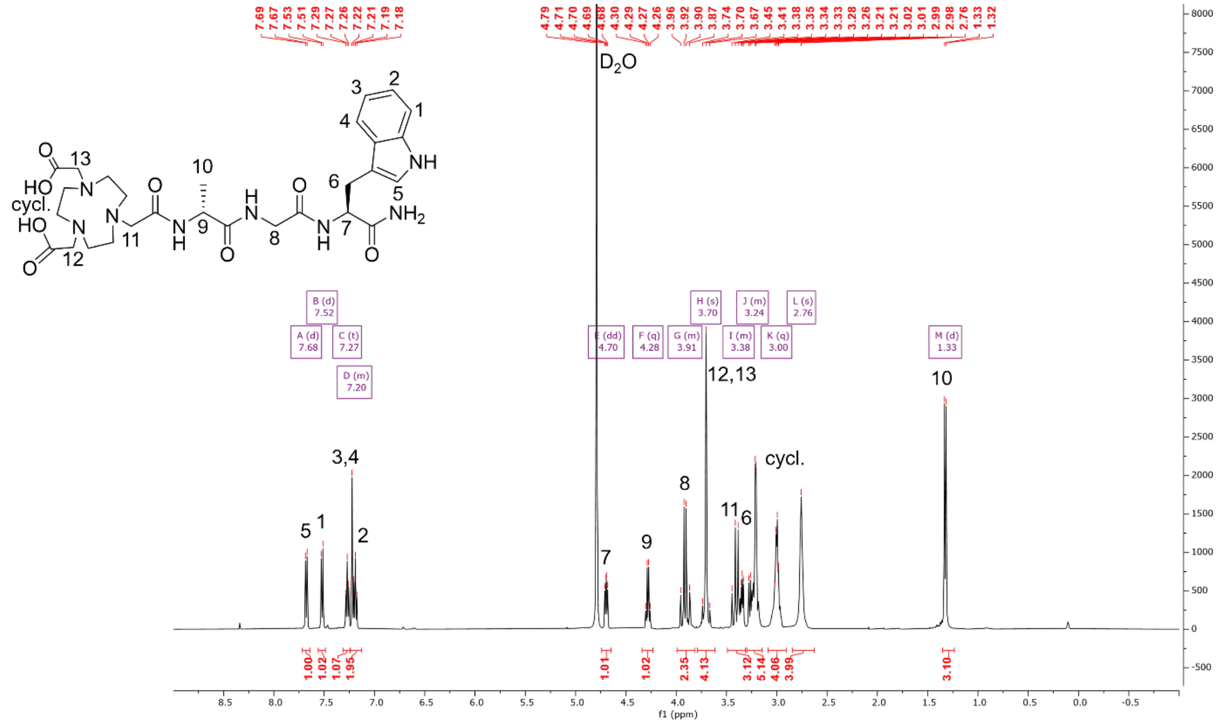

Figure S72. ^{1}H NMR (500 MHz, D_2O) for compound **12**.

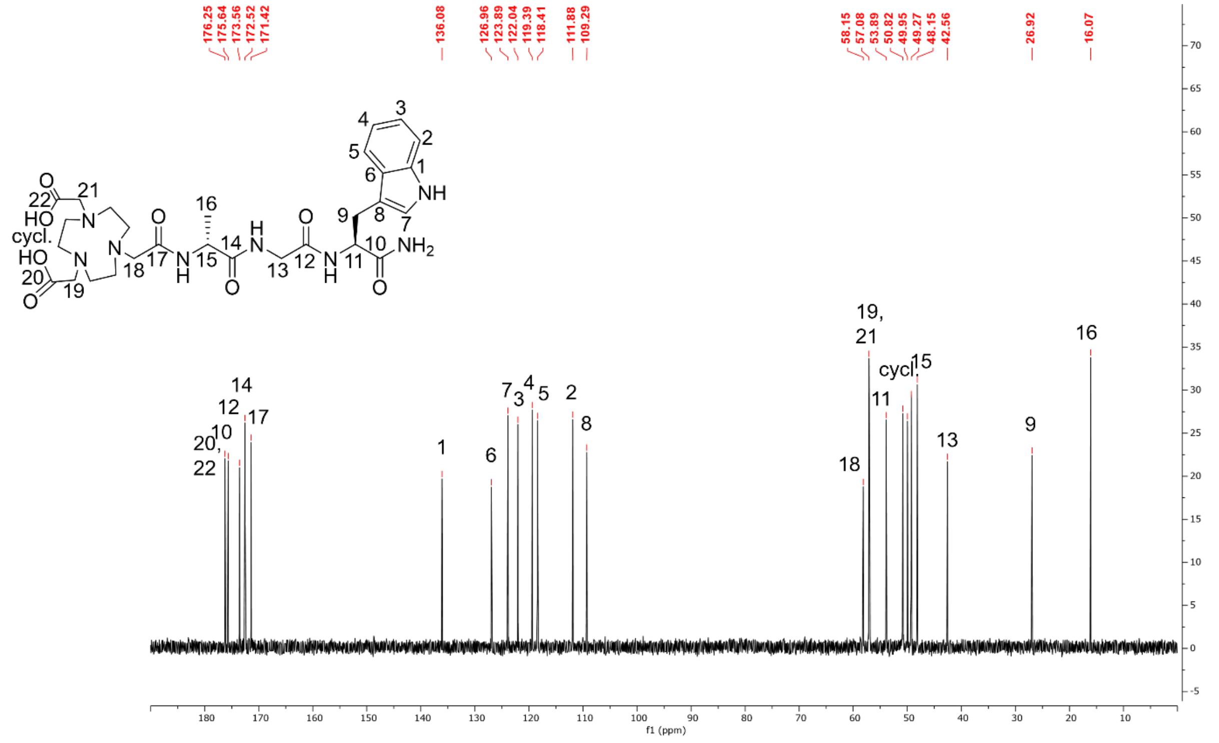

Figure S73. ^{13}C NMR (126 MHz, D_2O) for compound **12**.

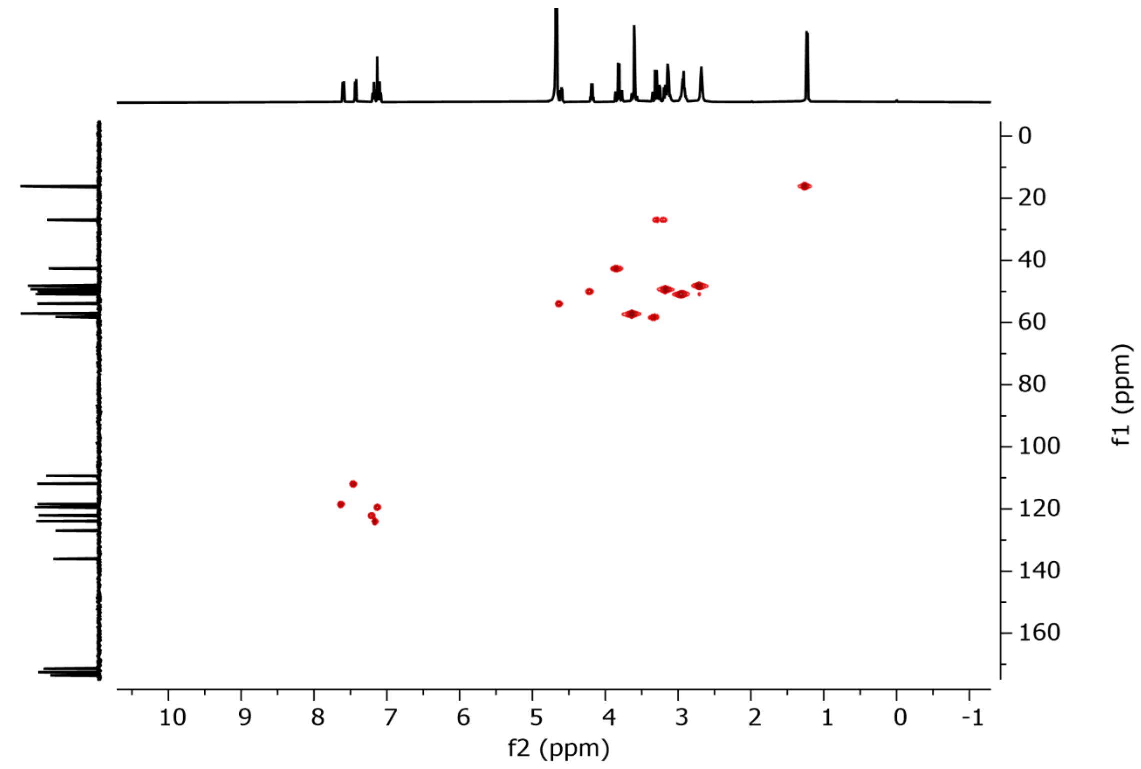

Figure S74. The HSQC NMR spectrum for compound **12**.

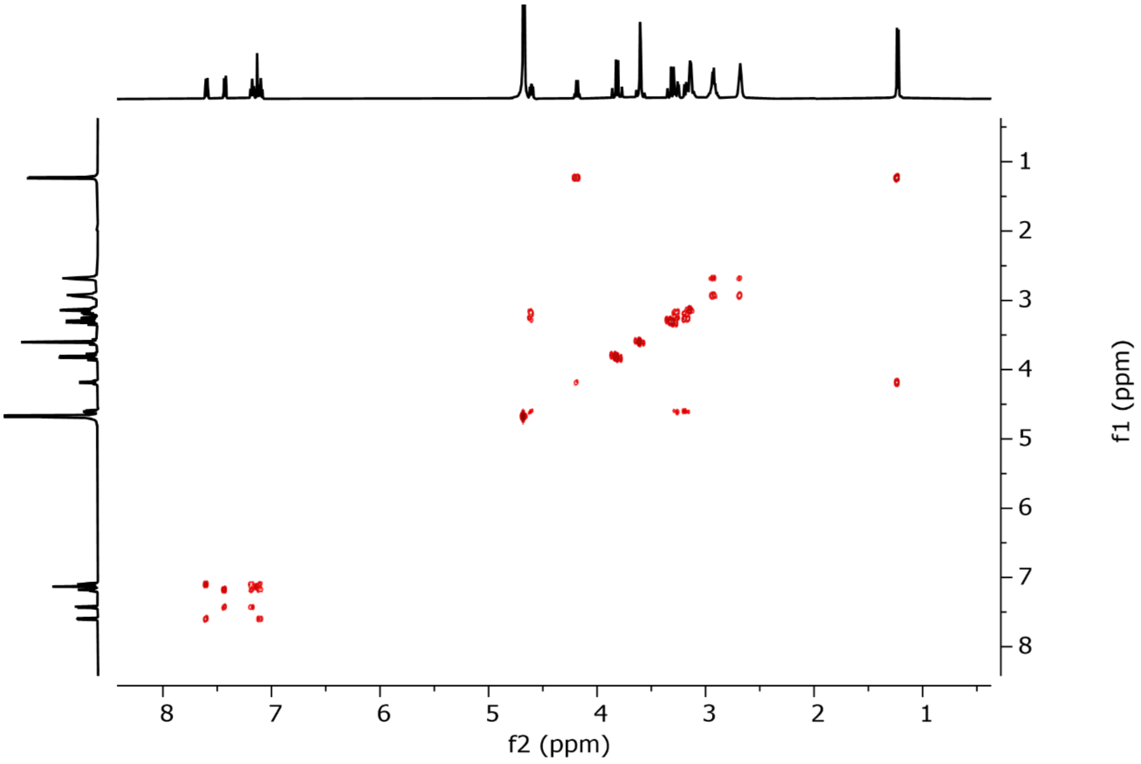

Figure S75. The ^1H-^1H COSY NMR spectrum for compound **12**.

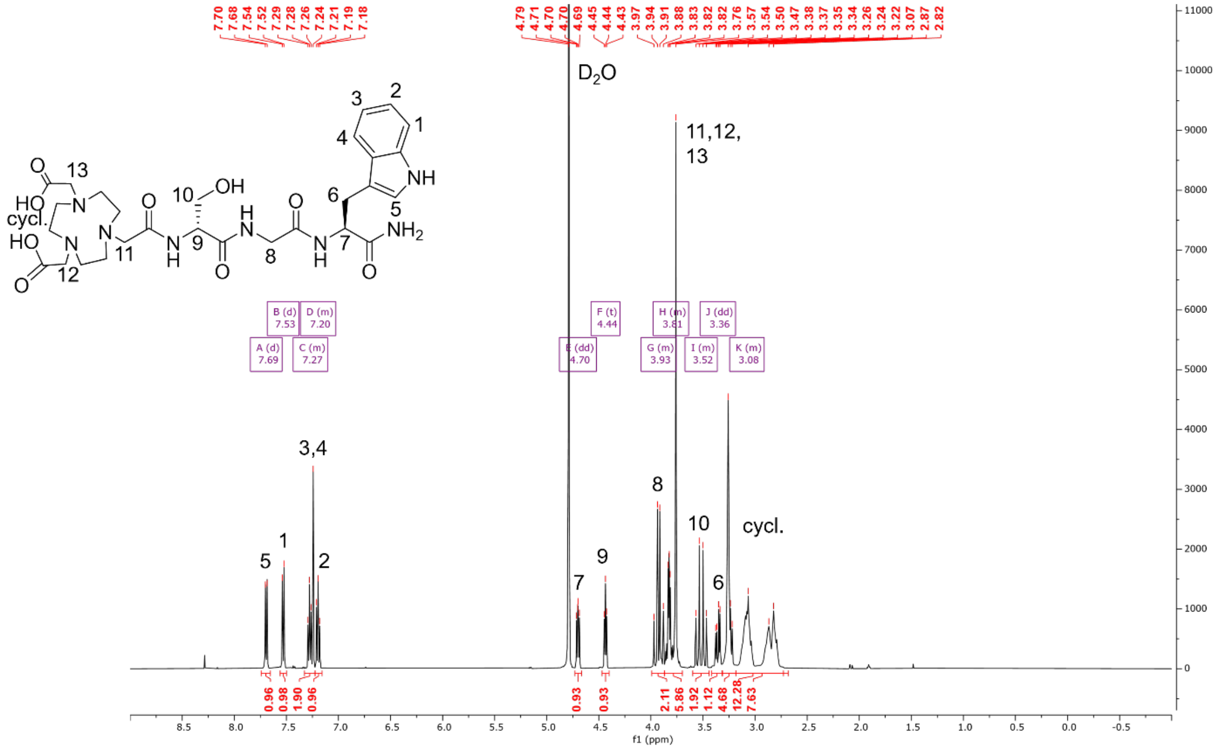

Figure S76. ^{1}H NMR (500 MHz, D_2O) for compound **13**.

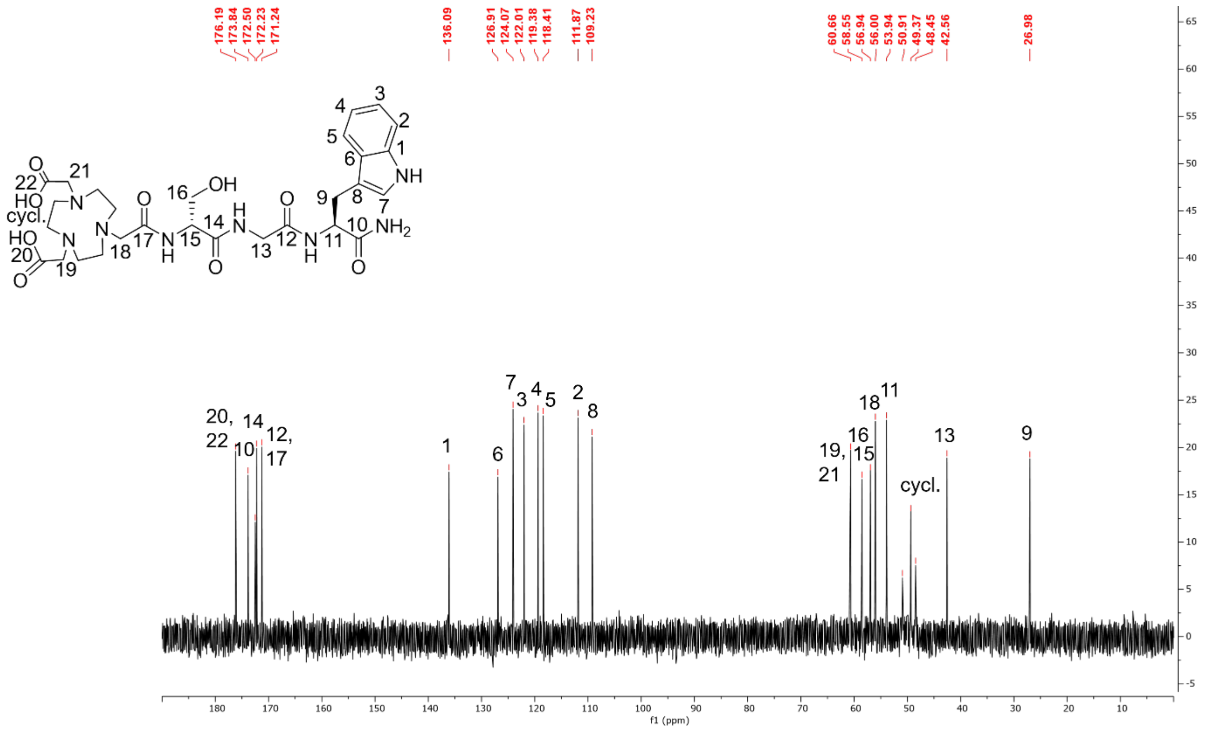

Figure S77. ^{13}C NMR (126 MHz, D_2O) for compound **13**.

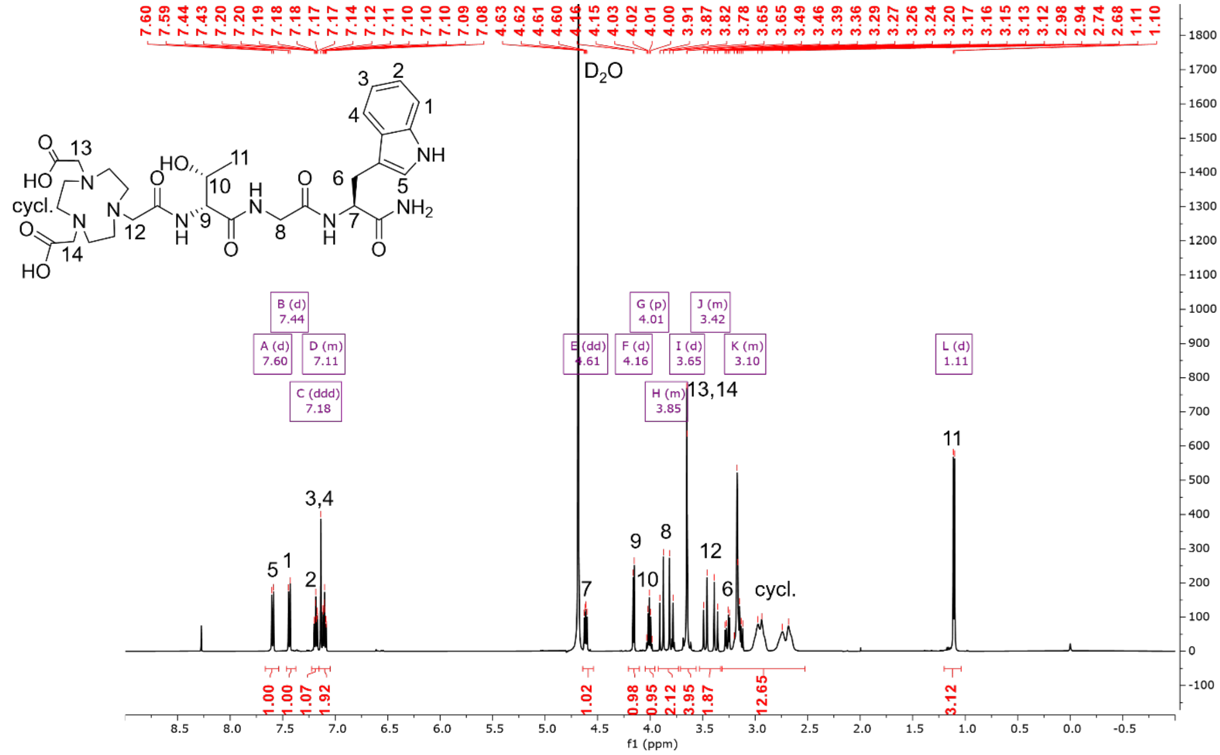

Figure S78. ^{1}H NMR (500 MHz, D$_2$O) for compound **14**.

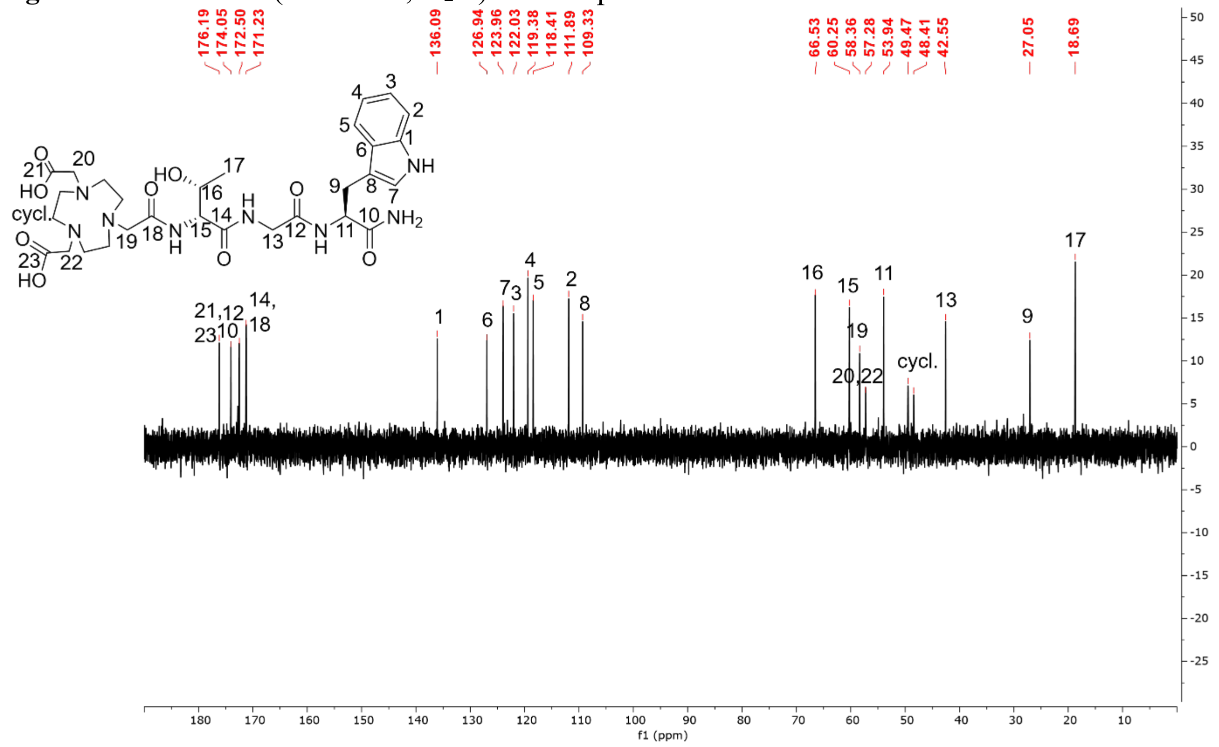

Figure S79. ^{13}C NMR (126 MHz, D$_2$O) for compound **14**.

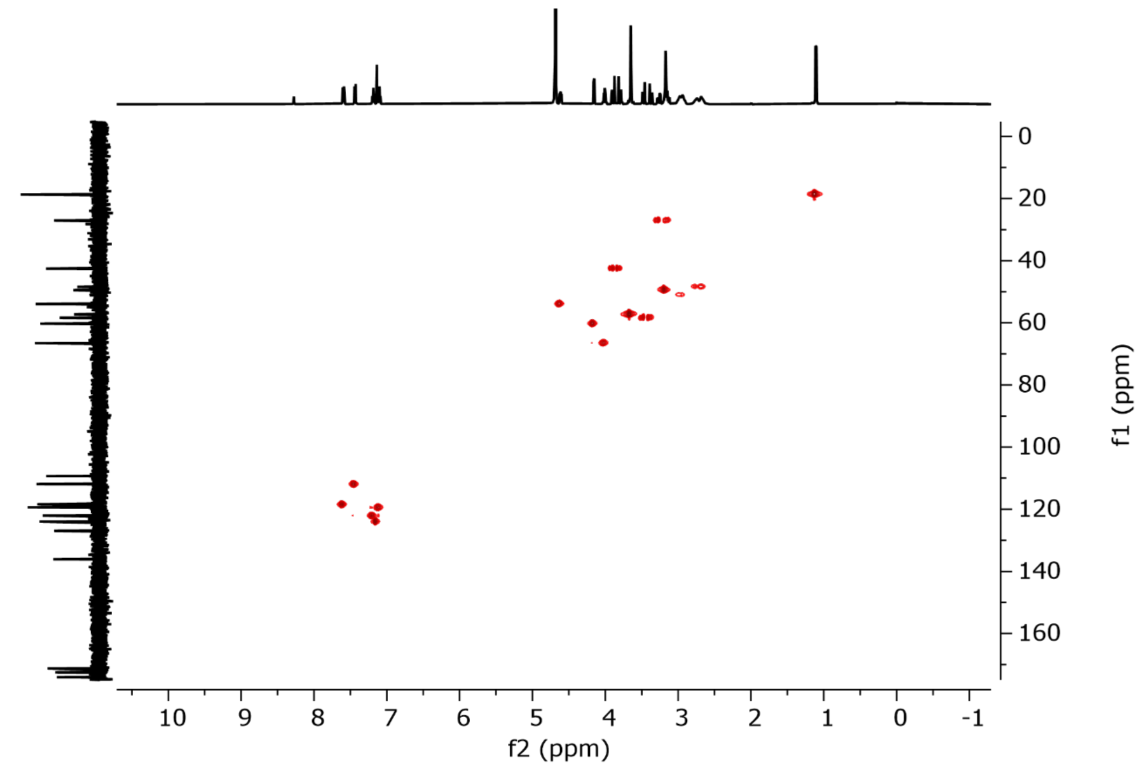

Figure S80. The HSQC NMR spectrum for compound **14**.

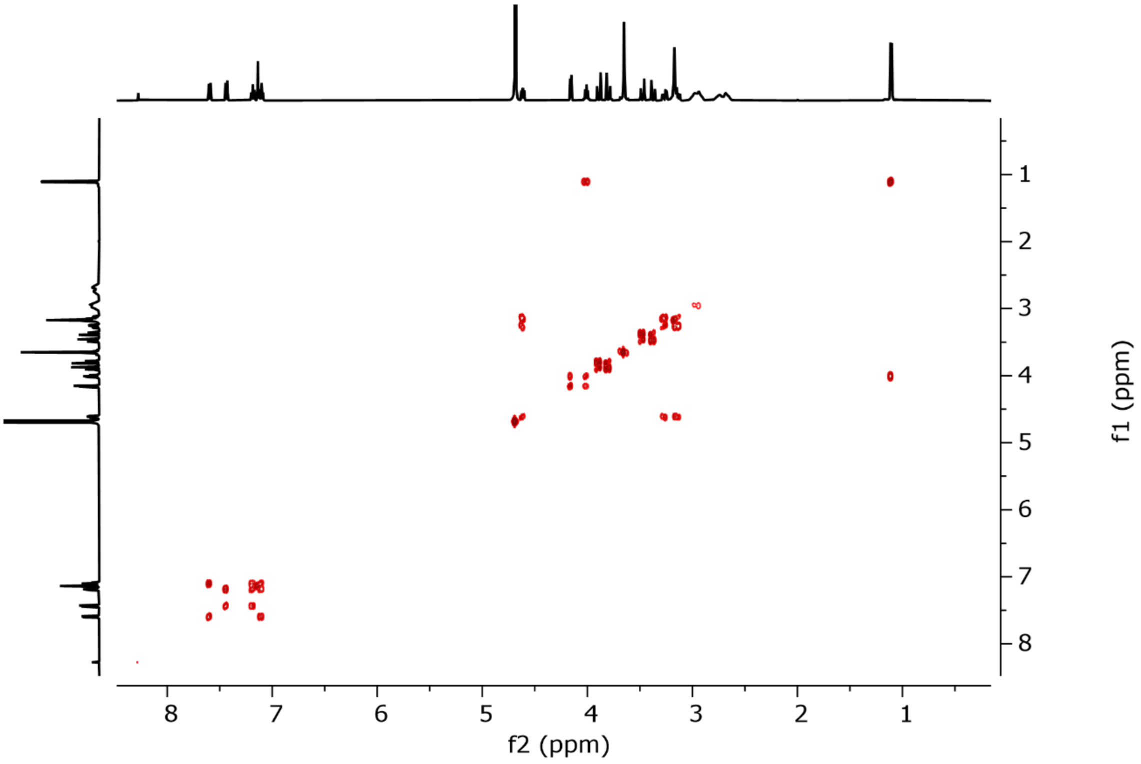

Figure S81. The ^{1}H-^{1}H COSY NMR spectrum for compound **14**.

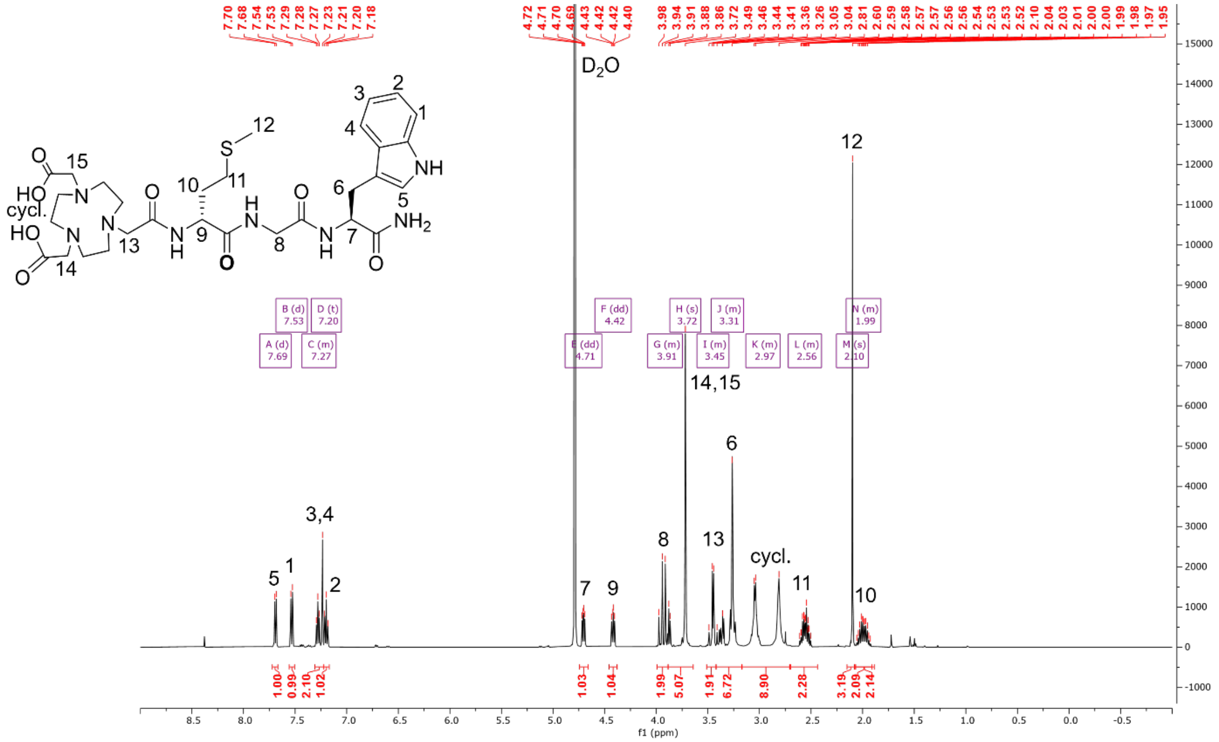

Figure S82. ^{1}H NMR (500 MHz, D_2O) for compound **15**.

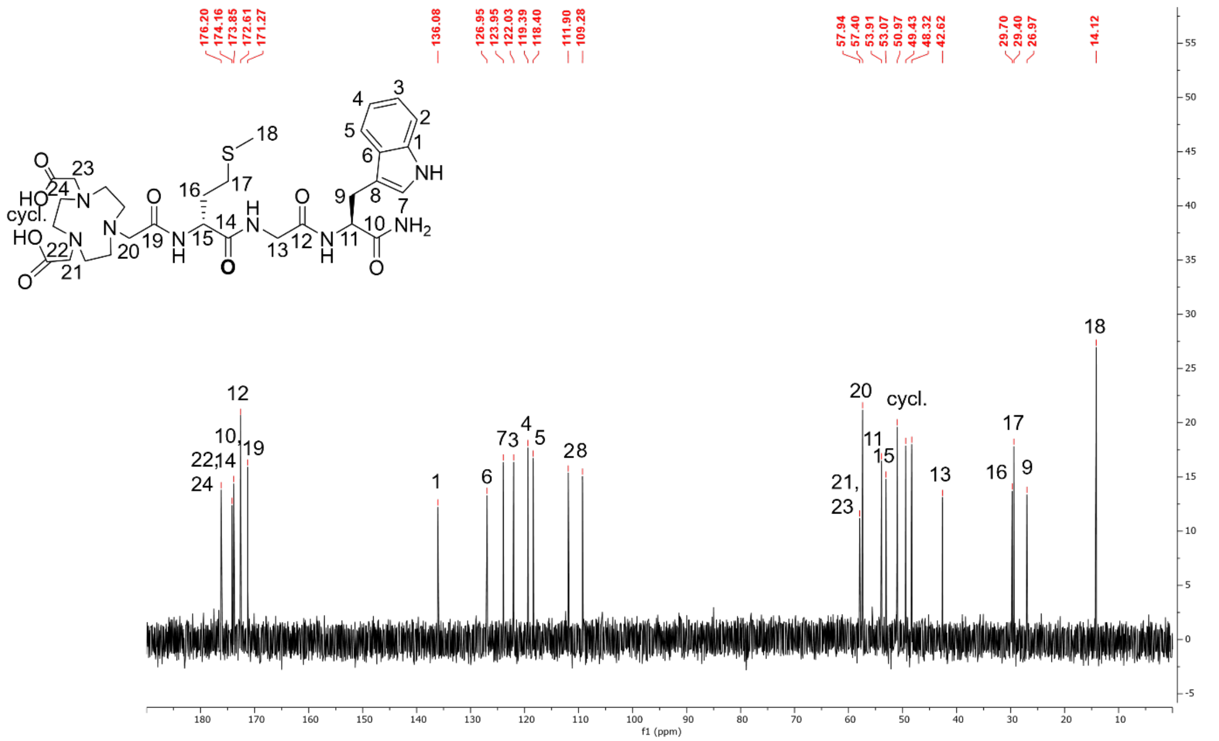

Figure S83. ^{13}C NMR (126 MHz, D_2O) for compound **15**.

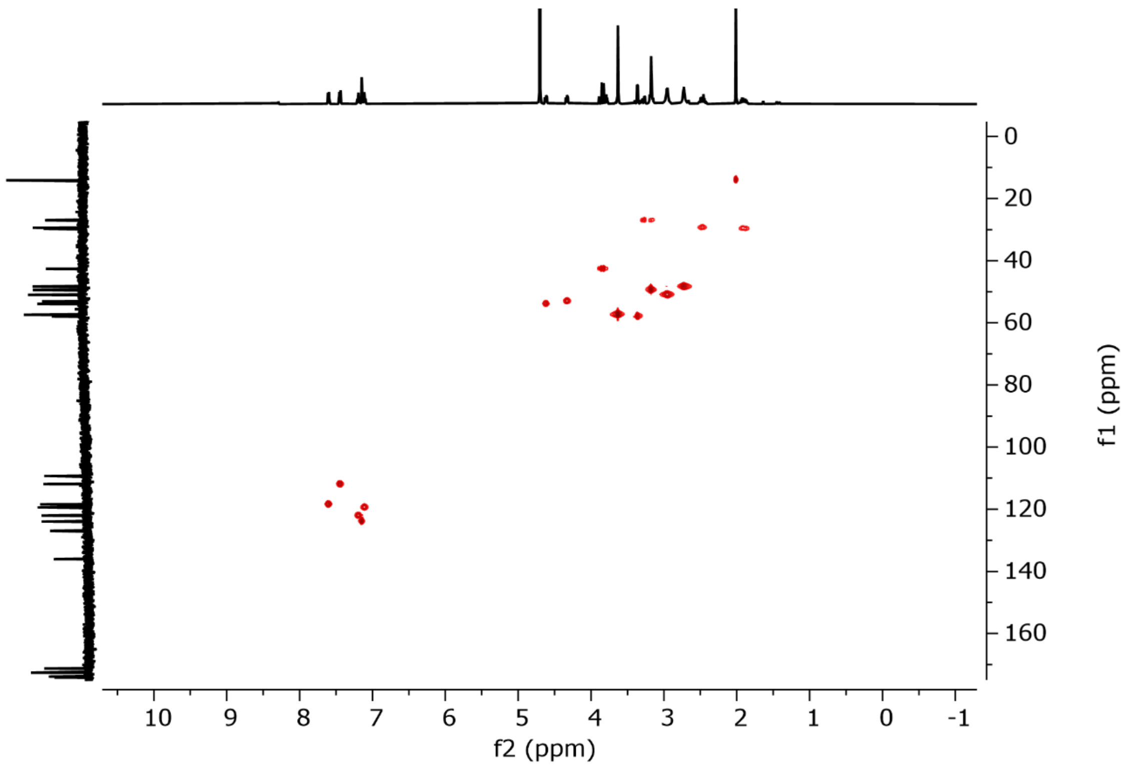

Figure S84. The HSQC NMR spectrum for compound **15**.

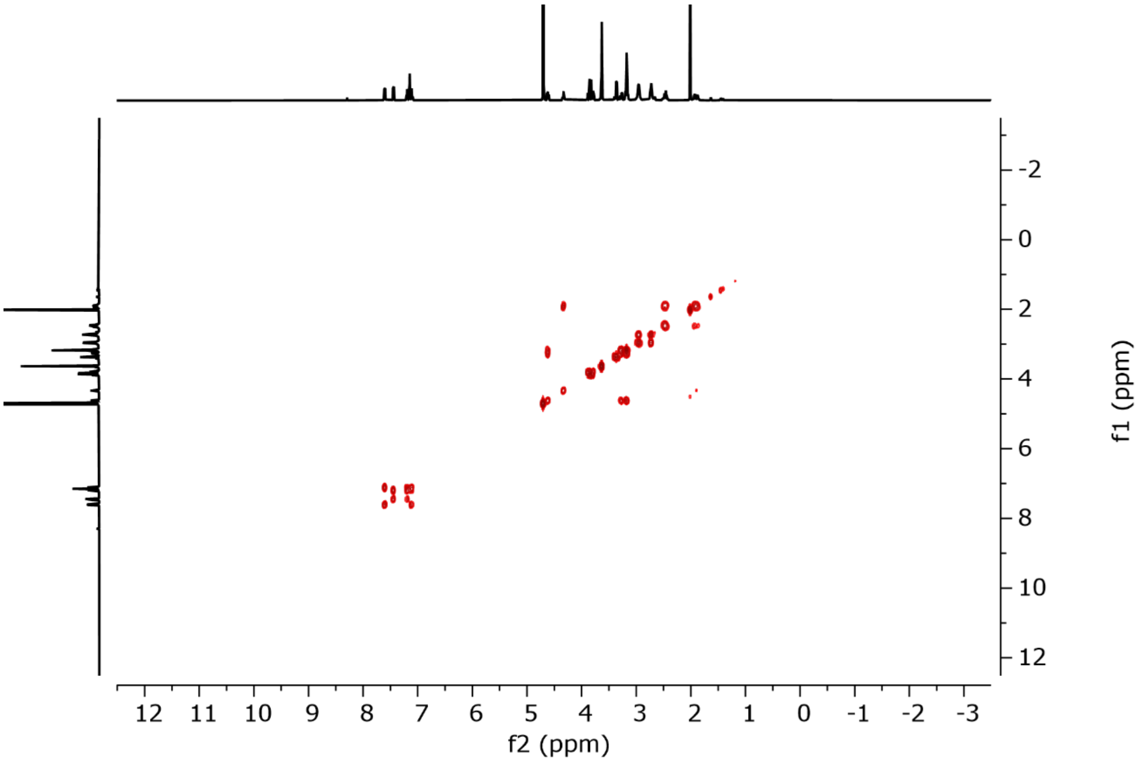

Figure S85. The ^{1}H-^{1}H COSY NMR spectrum for compound **15**.

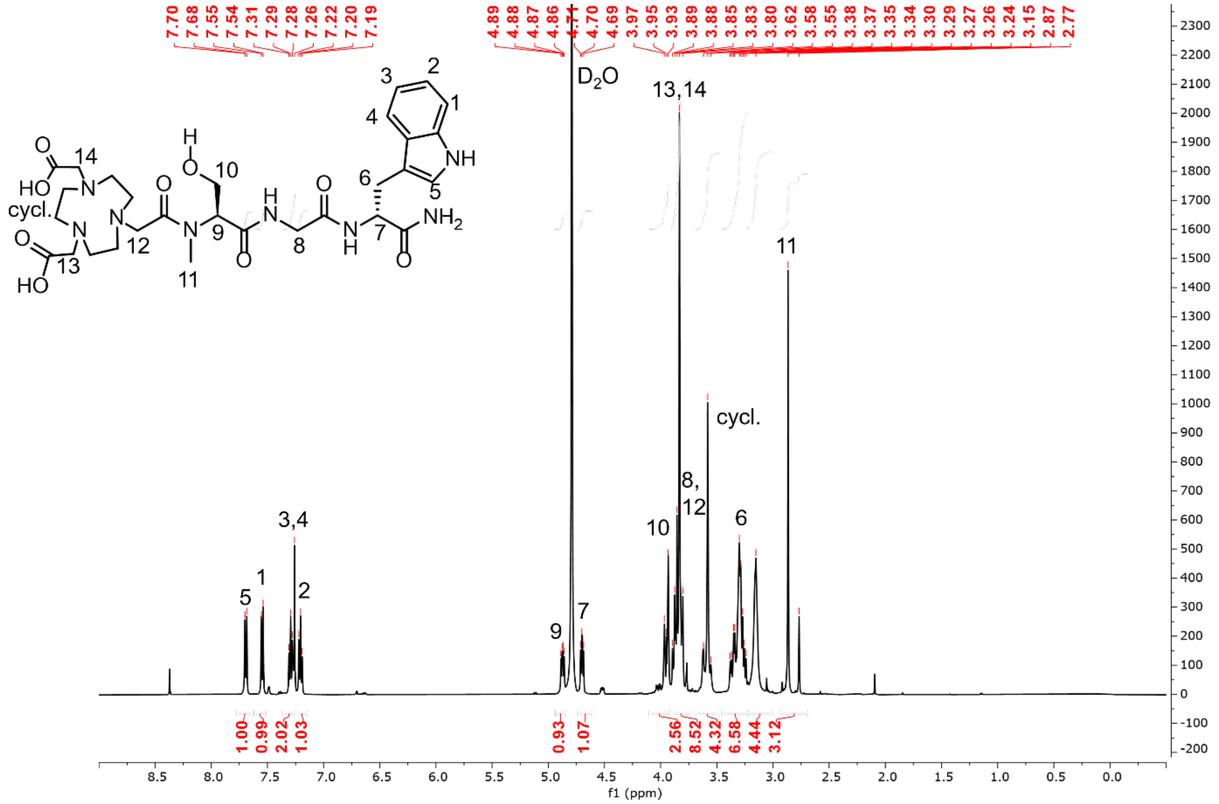

Figure S86. ^{1}H NMR (500 MHz, D_2O) for compound **16**.

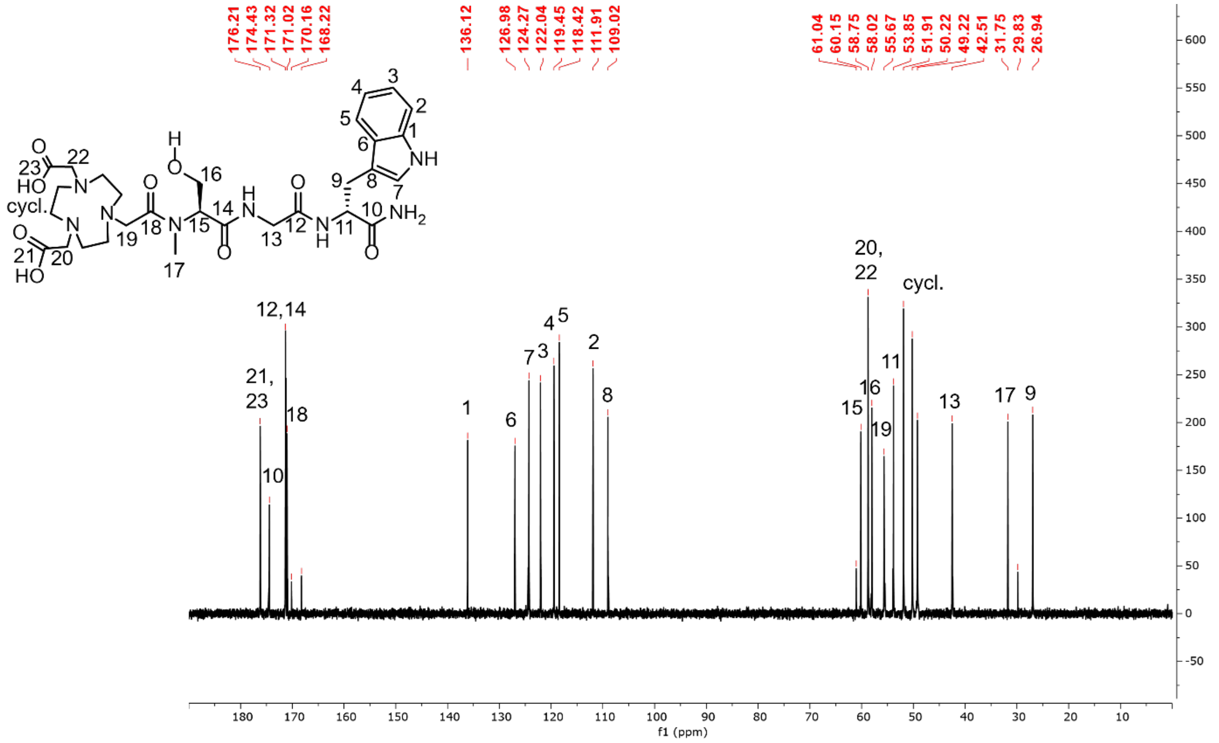

Figure S87. ^{13}C NMR (126 MHz, D_2O) for compound **16**.

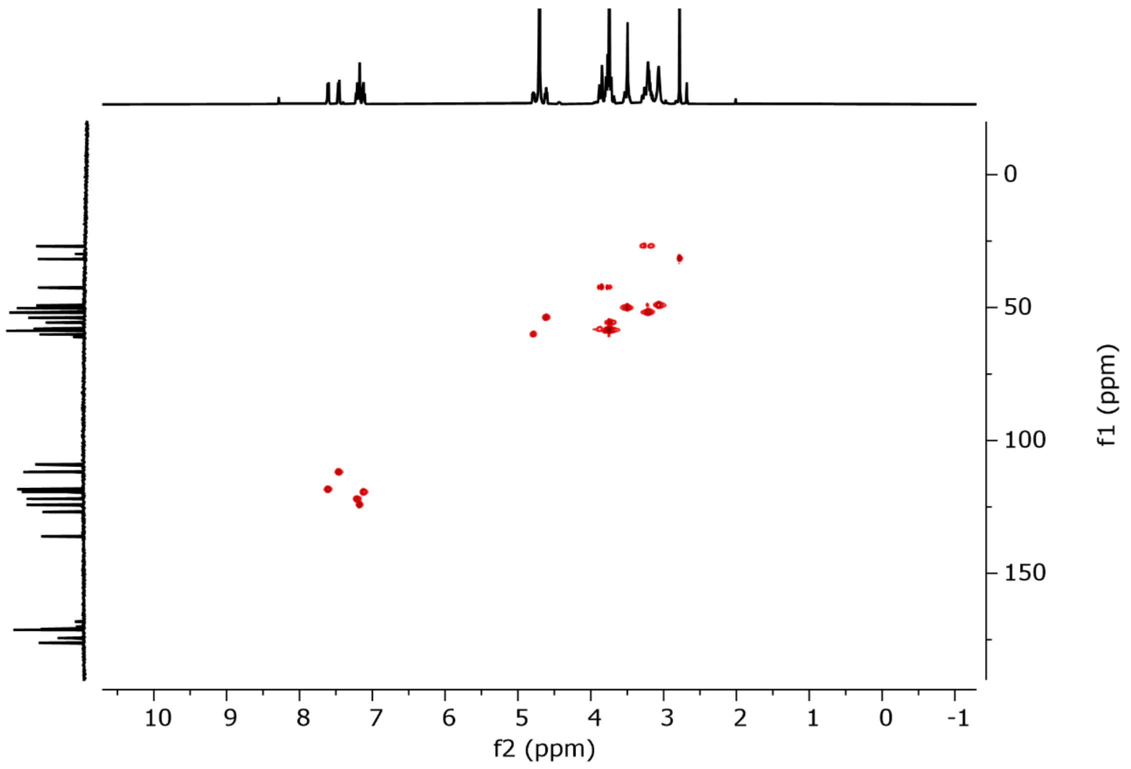

Figure S88. The HSQC NMR spectrum for compound **16**.

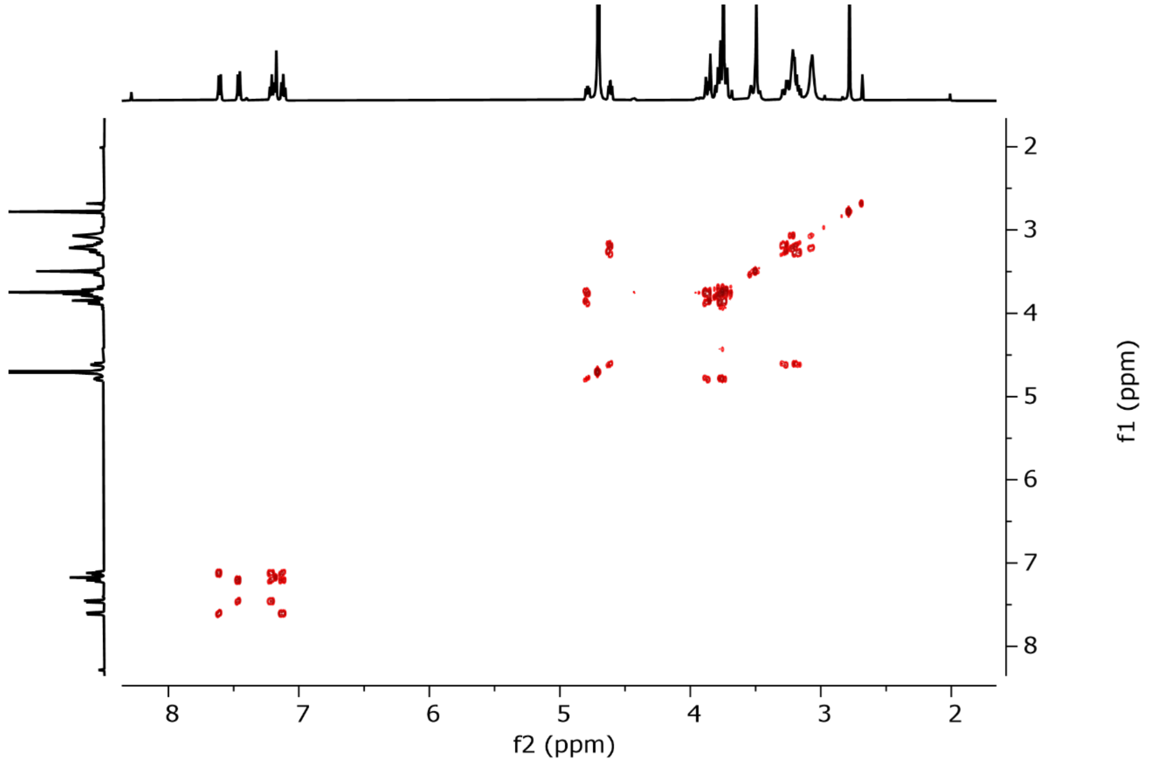

Figure S89. The ^{1}H-^{1}H COSY NMR spectrum for compound **16**.

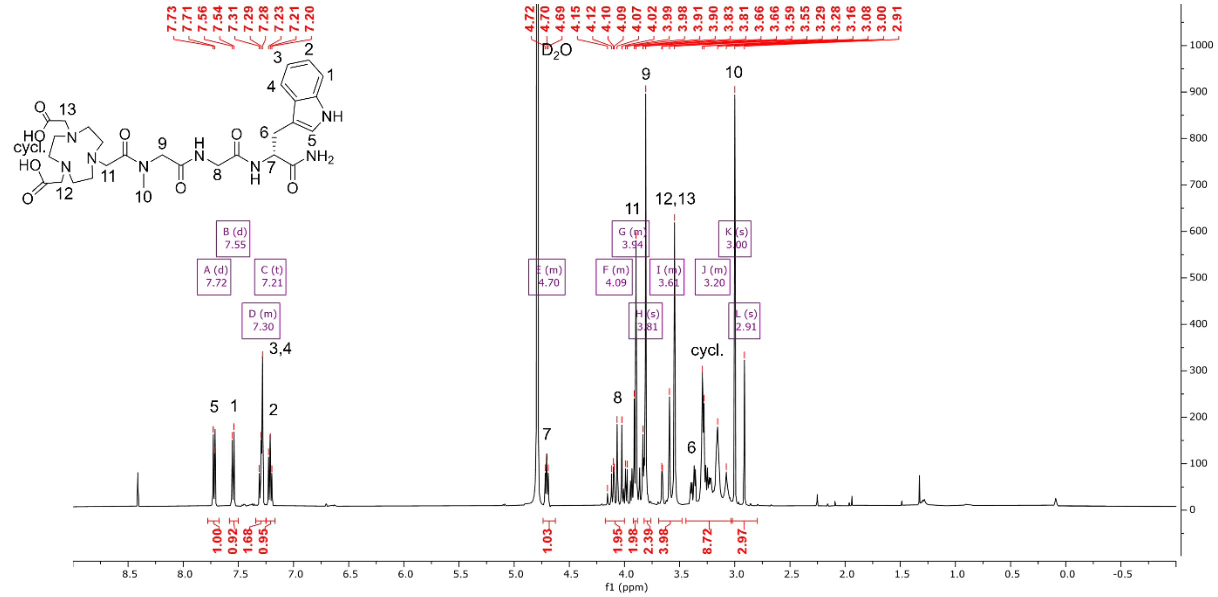

Figure S90. ^1H NMR (500 MHz, D_2O) for compound **17**.

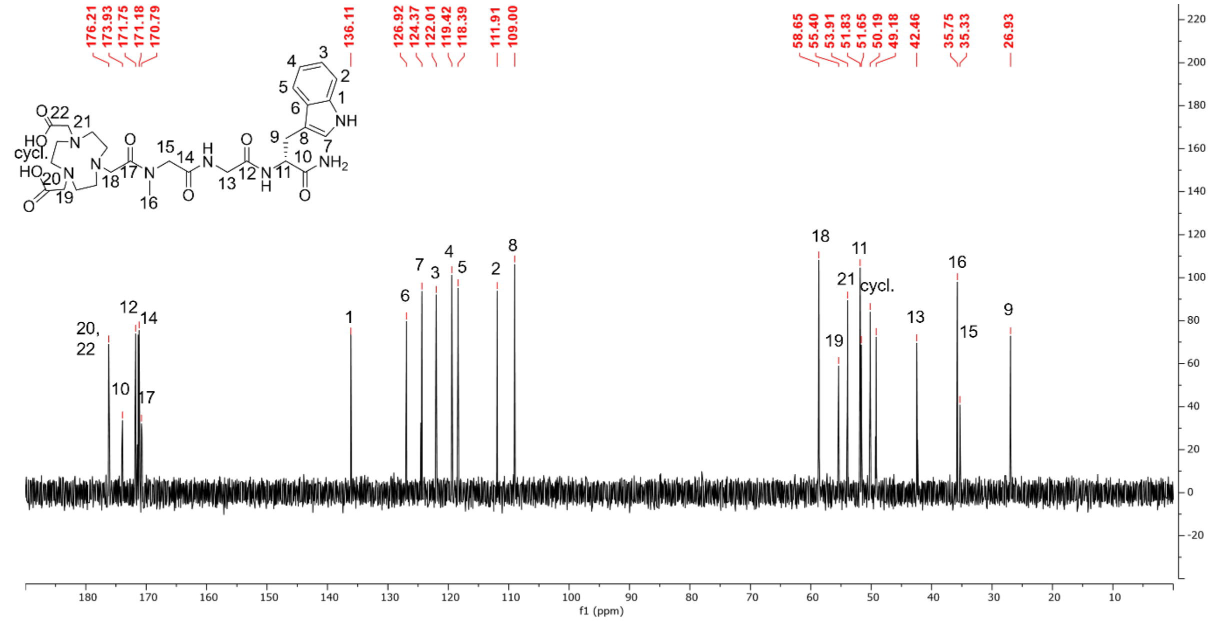

Figure S91. ^{13}C NMR (126 MHz, D_2O) for compound **17**.

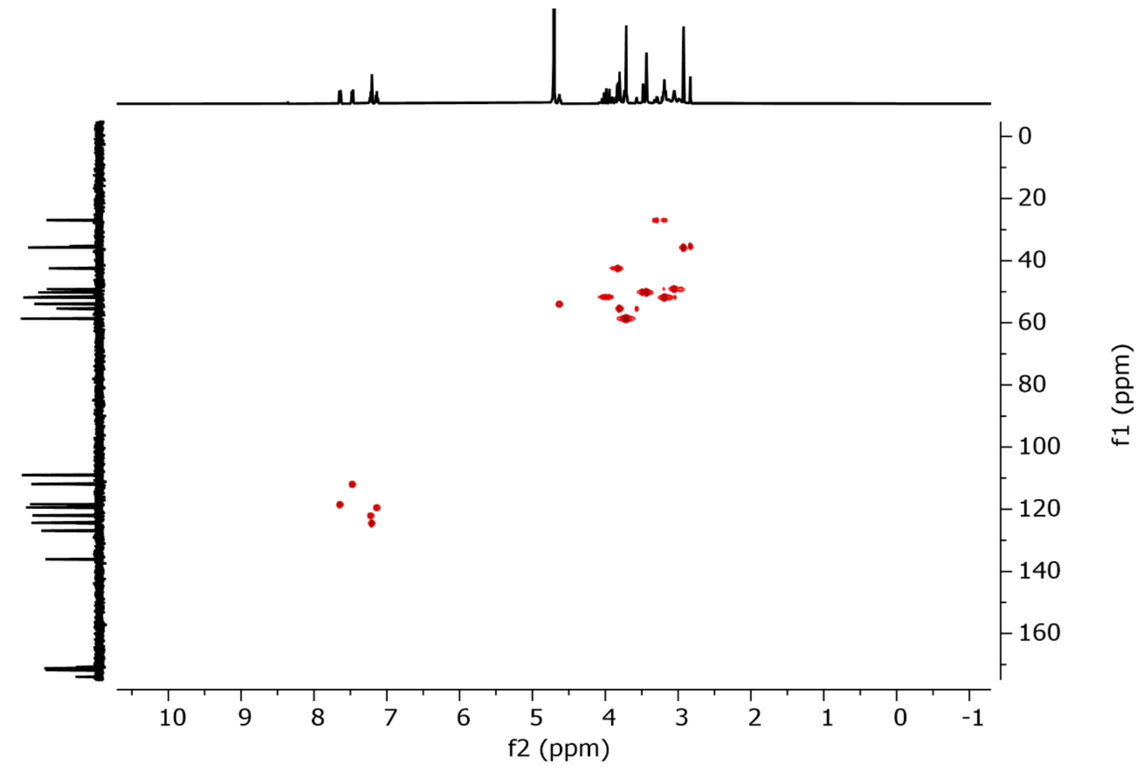

Figure S92. The HSQC NMR spectrum for compound **17**.

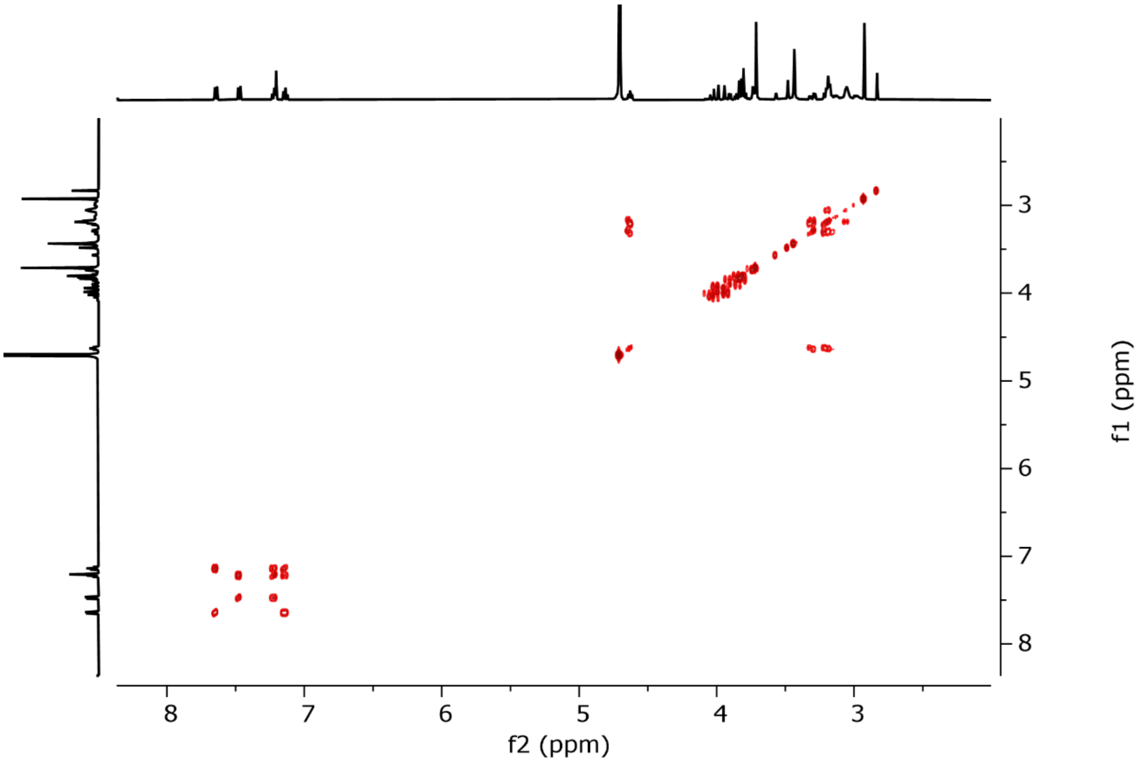

Figure S93. The ^{1}H-^{1}H COSY NMR spectrum for compound **17**.

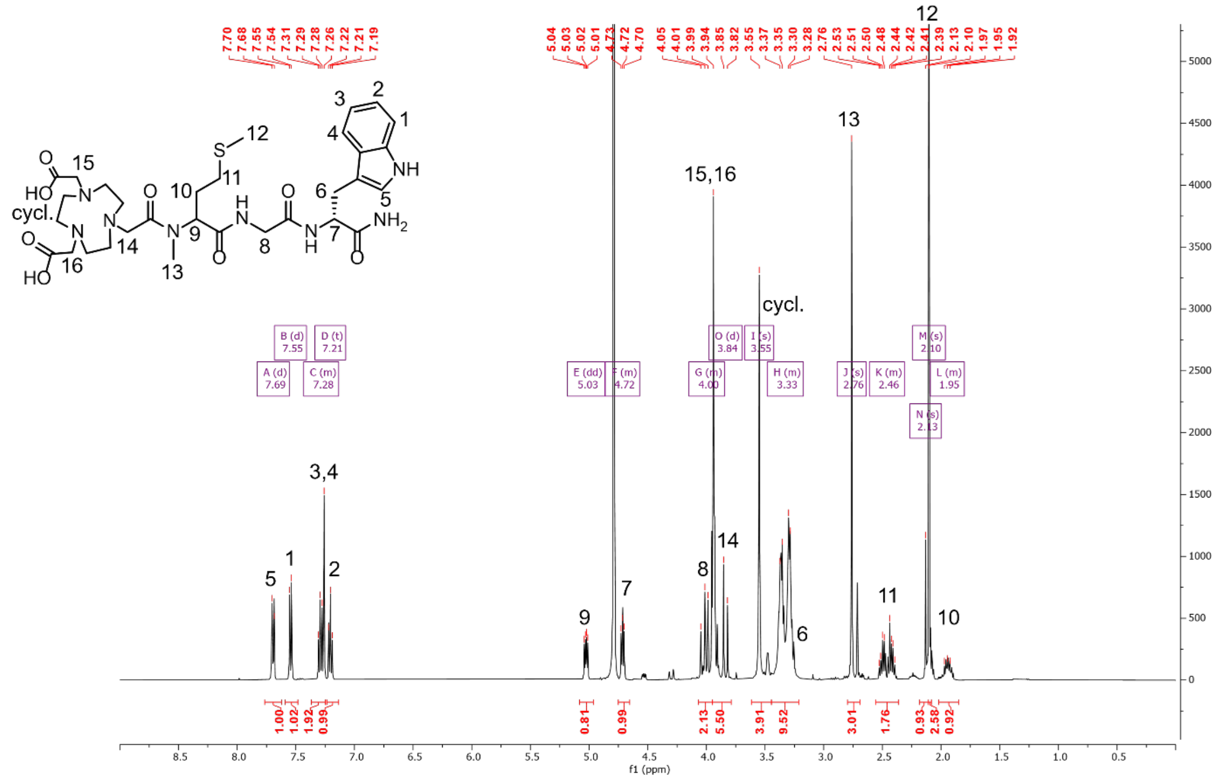

Figure S94. ^{1}H NMR (500 MHz, D_2O) for compound **18**.

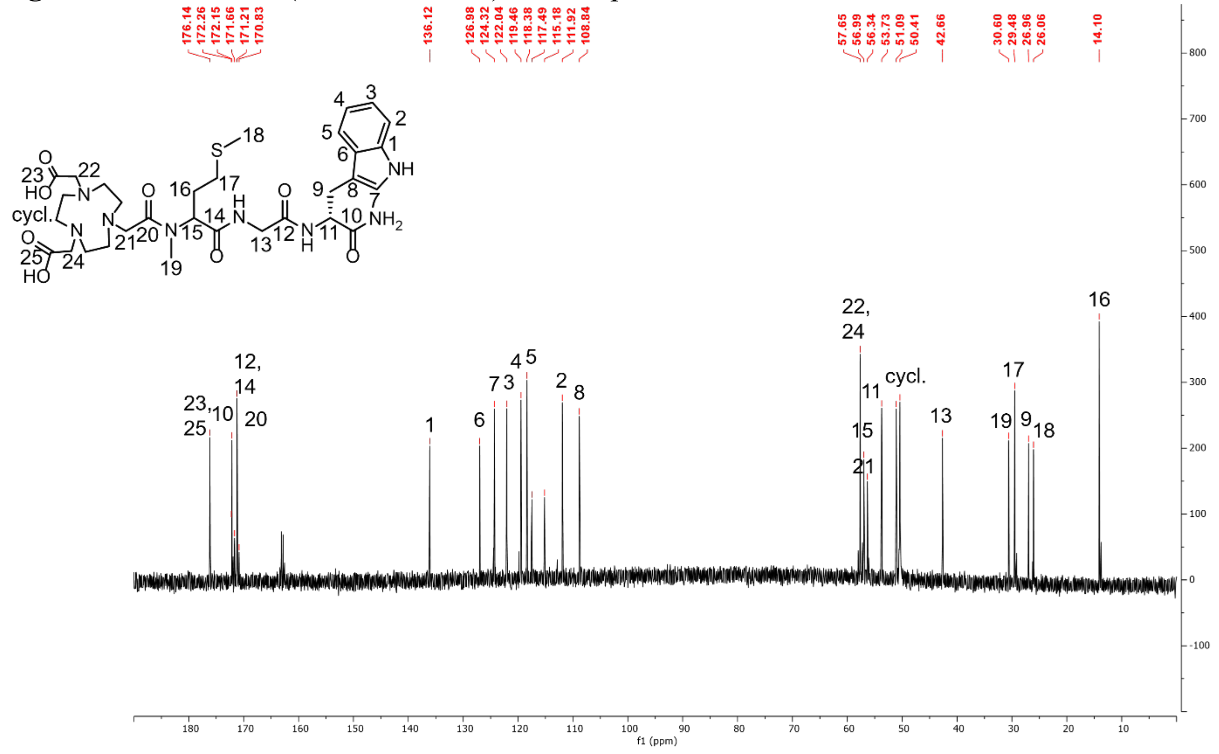

Figure S95. ^{13}C NMR (126 MHz, D_2O) for compound **18**.

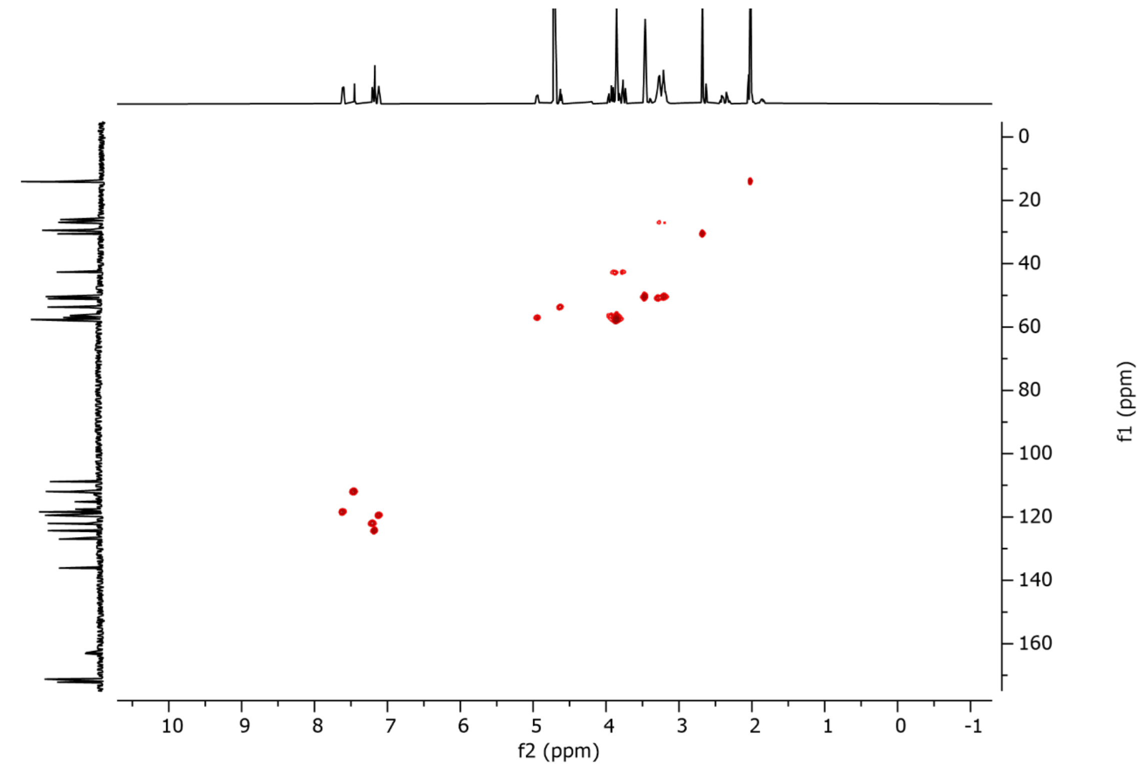

Figure S96. The HSQC NMR spectrum for compound **18**.

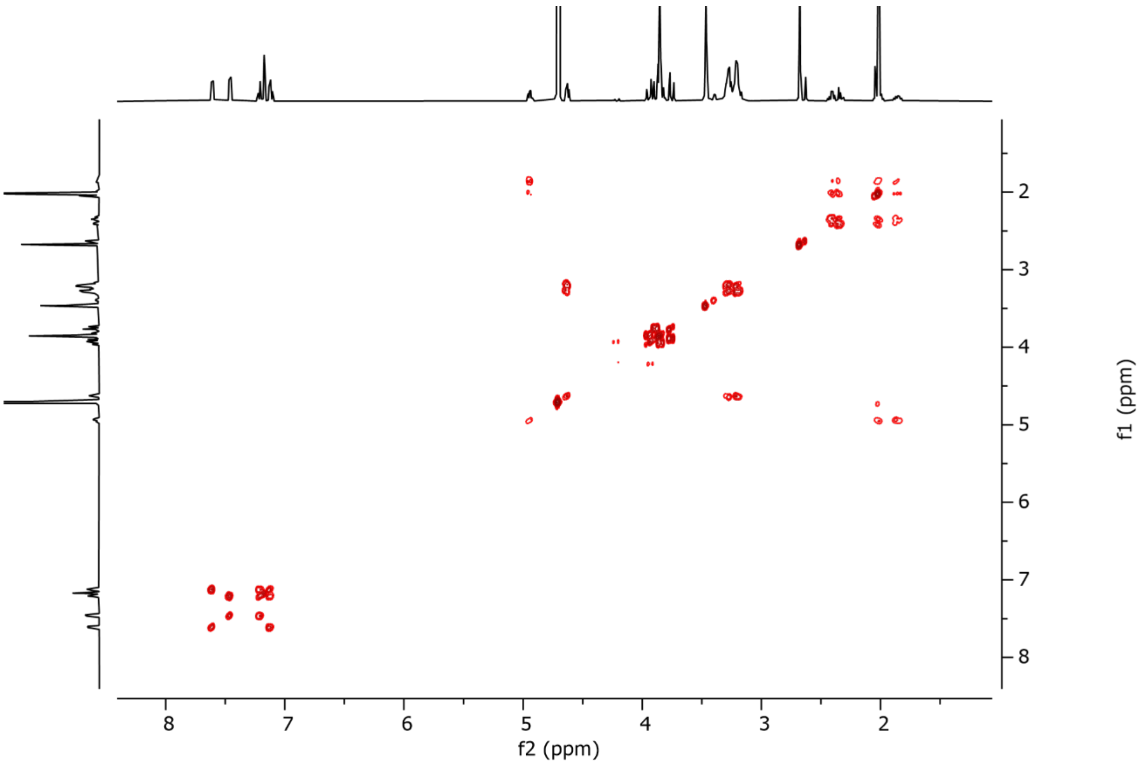

Figure S97. The ^{1}H-^{1}H COSY NMR spectrum for compound **18**.

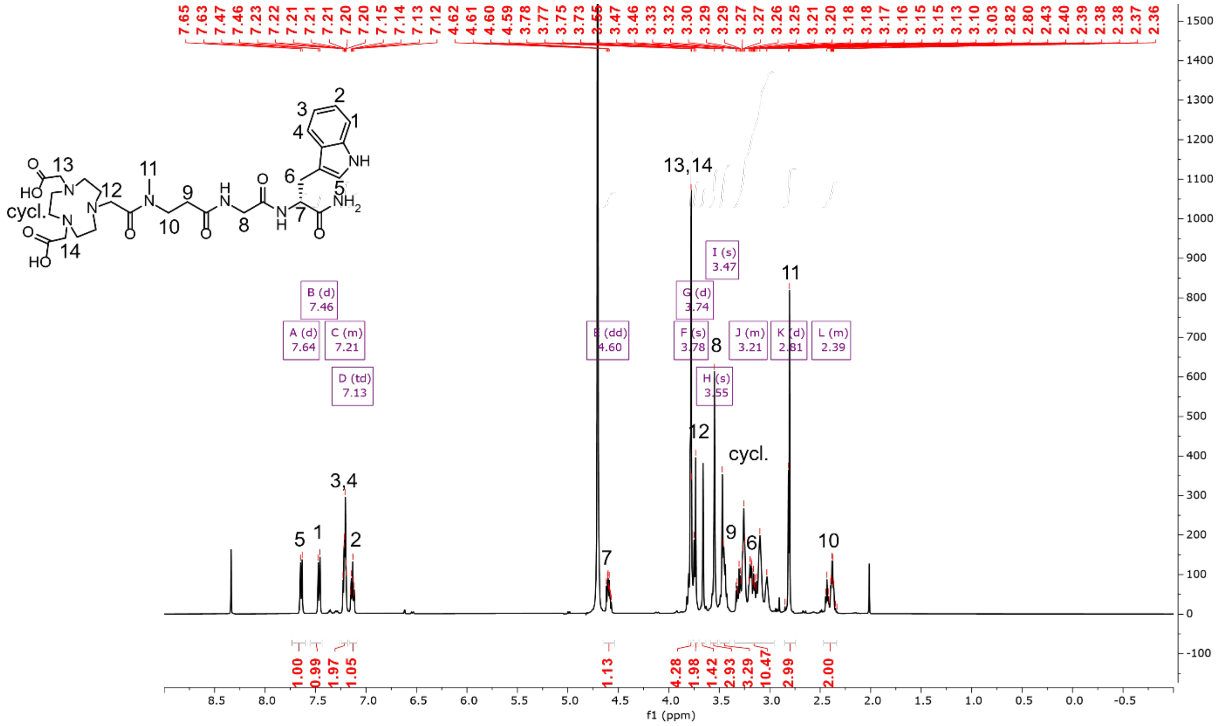

Figure S98. ^{1}H NMR (500 MHz, D_2O) for compound **19**.

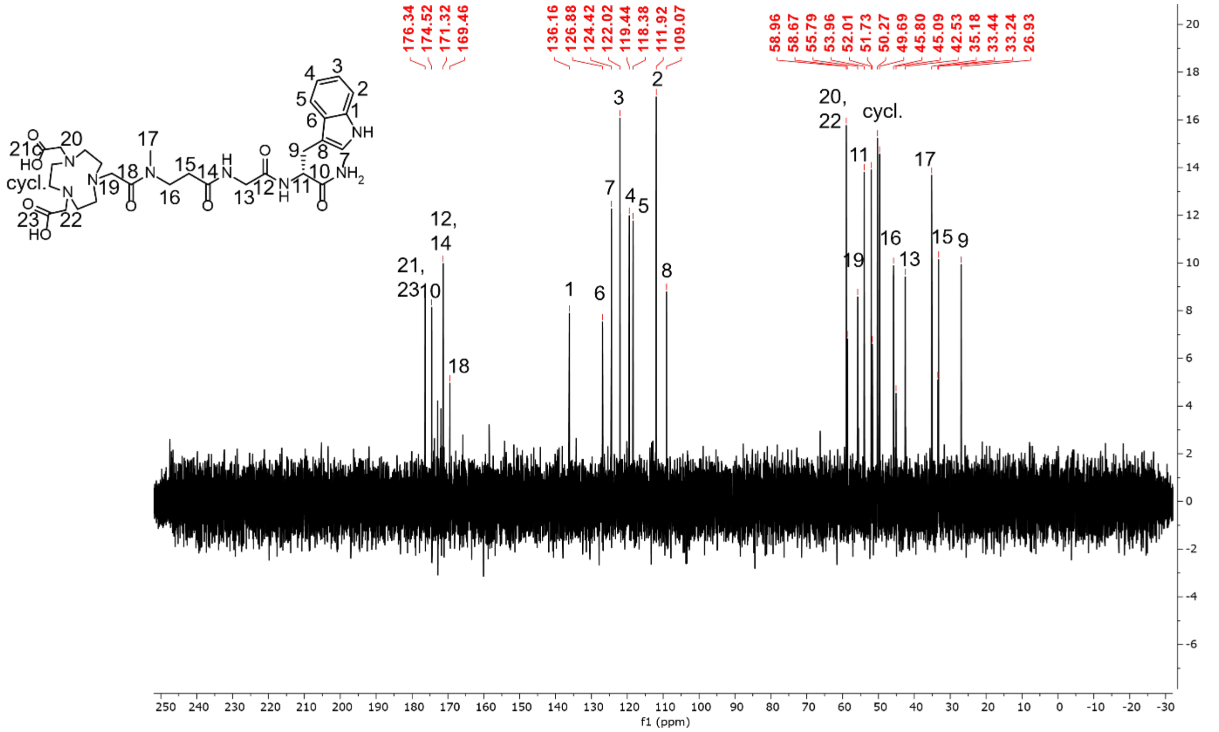

Figure S99. ^{13}C NMR (126 MHz, D_2O) for compound **19**.

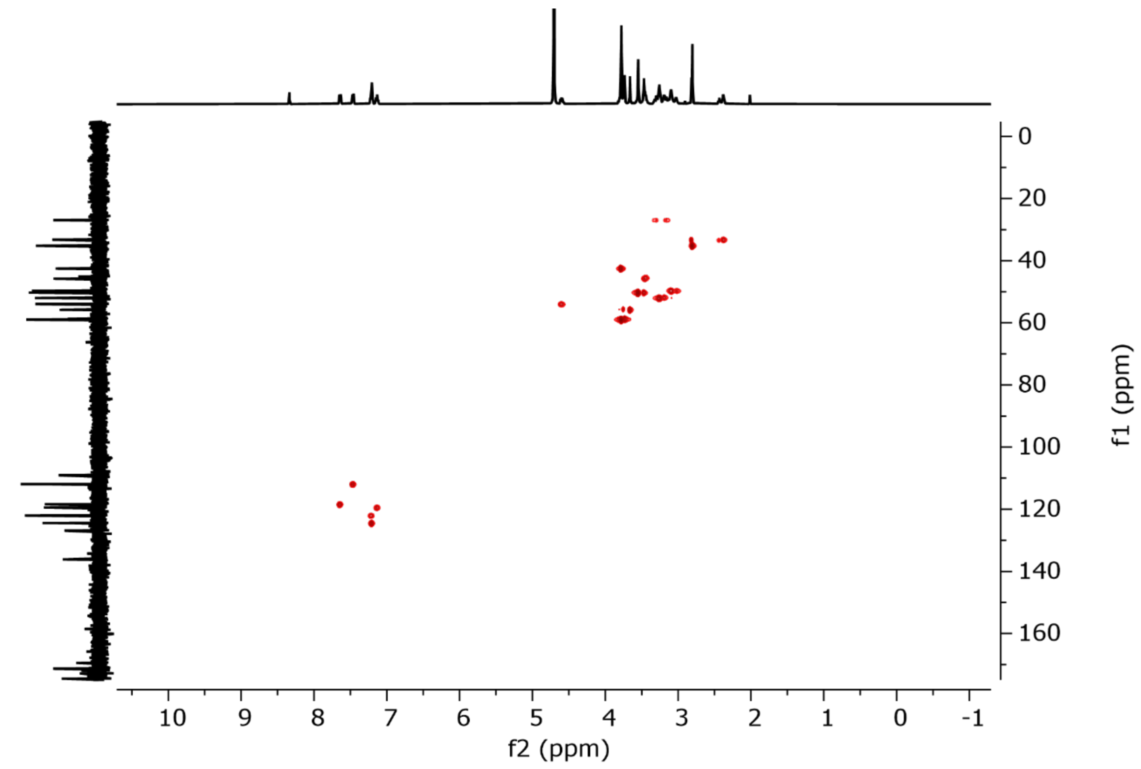

Figure S100. The HSQC NMR spectrum for compound **19**.

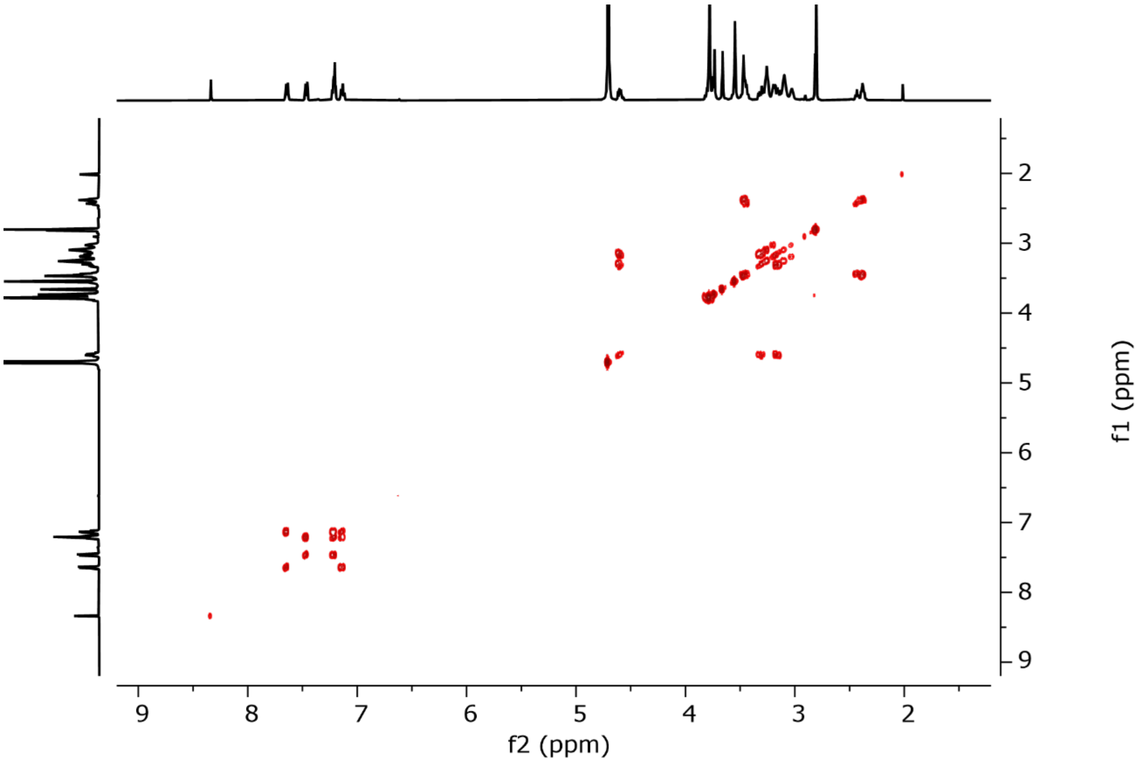

Figure S101. The ^{1}H-^{1}H COSY NMR spectrum for compound **19**.

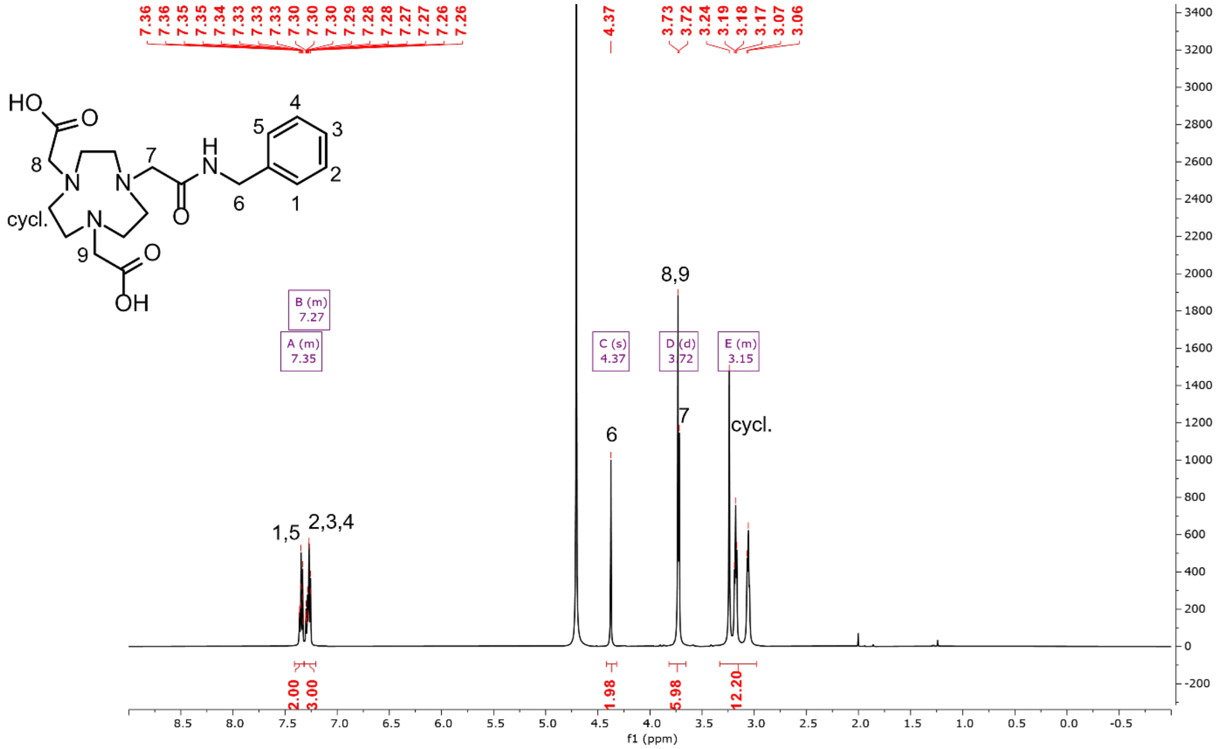

Figure S102. ^{1}H NMR (500 MHz, D_2O) for compound **26**.

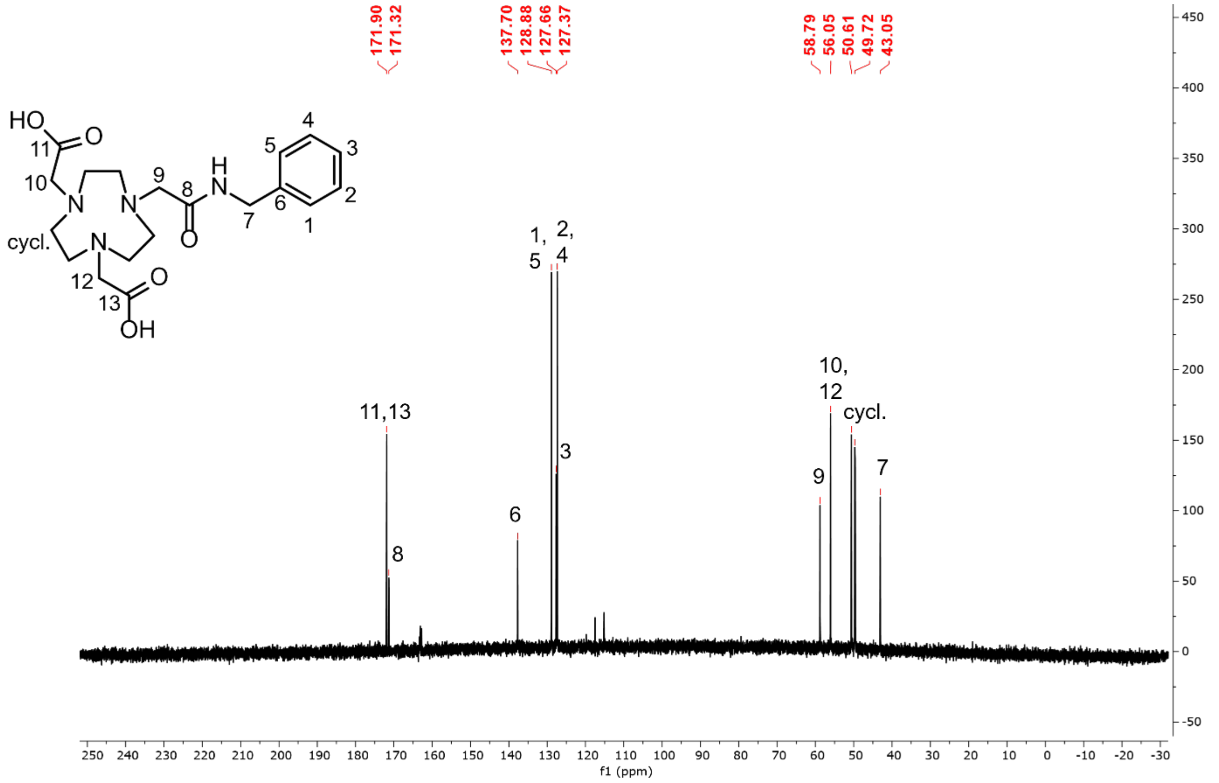

Figure S103. ^{13}C NMR (126 MHz, D_2O) for compound **26.**

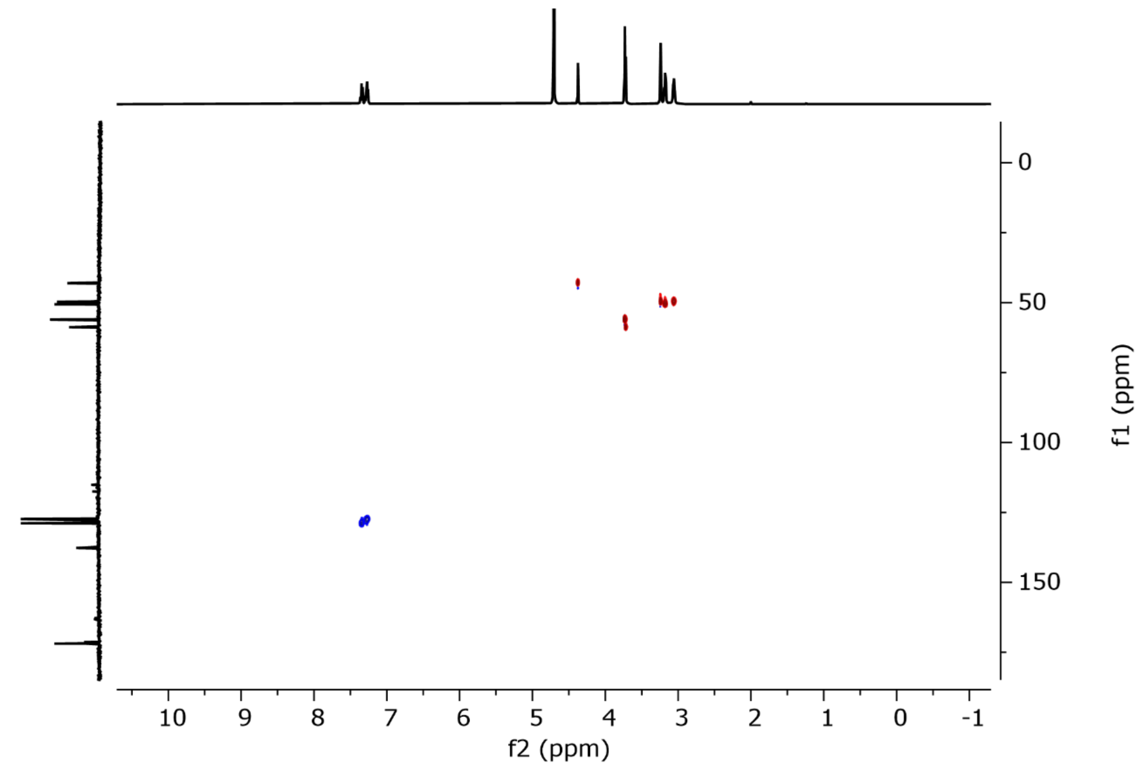

Figure S104. The HSQC NMR spectrum for compound **26**.

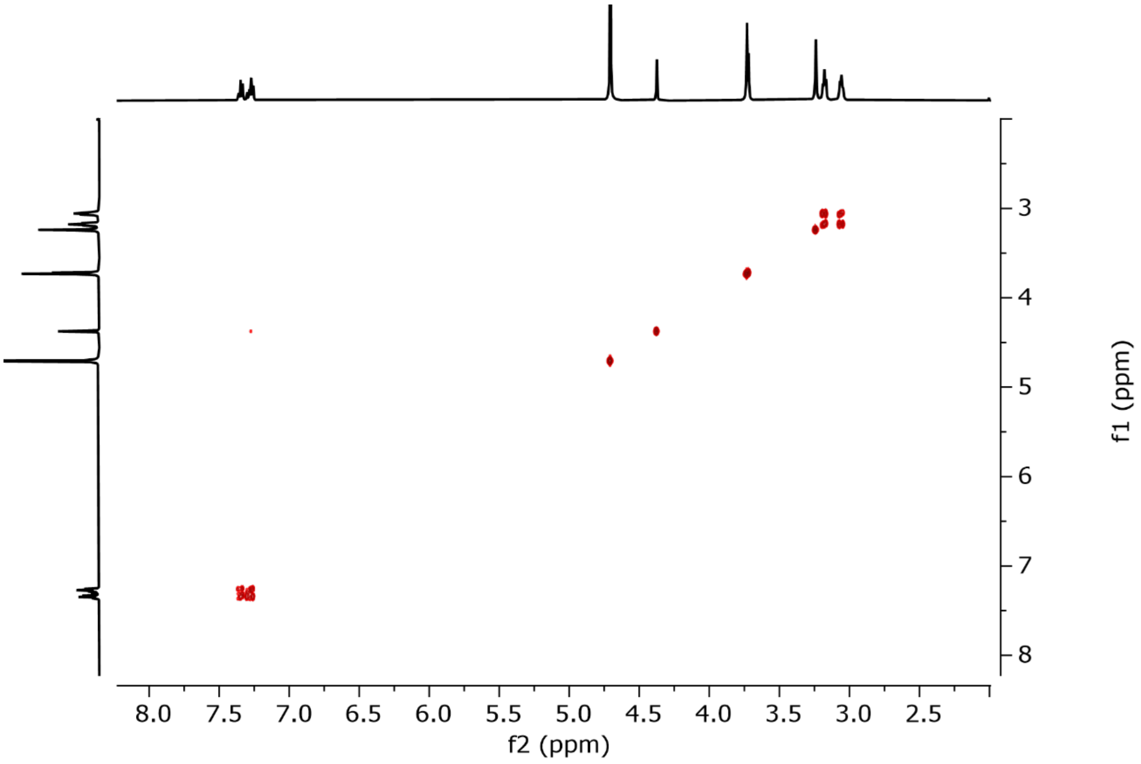

Figure S105. The ^{1}H-^{1}H COSY NMR spectrum for compound **26**.

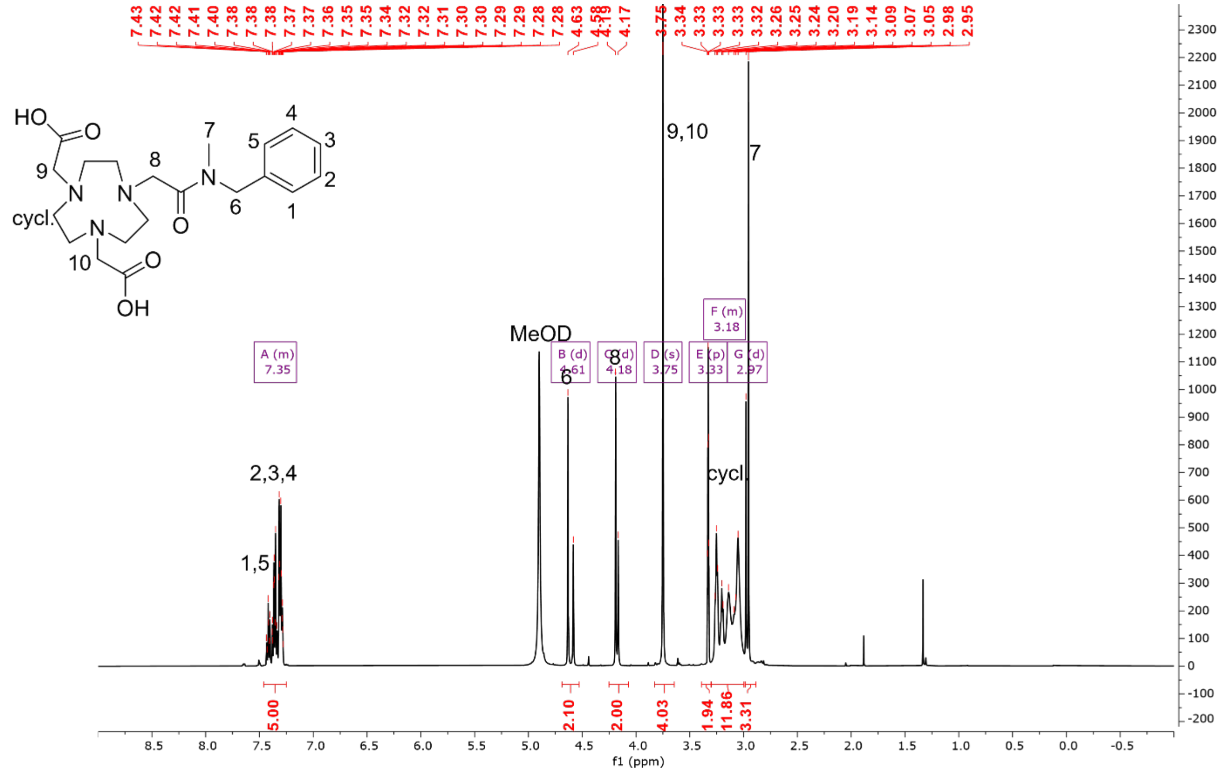

Figure S106. ^{1}H NMR (500 MHz, MeOD) for compound **27**.

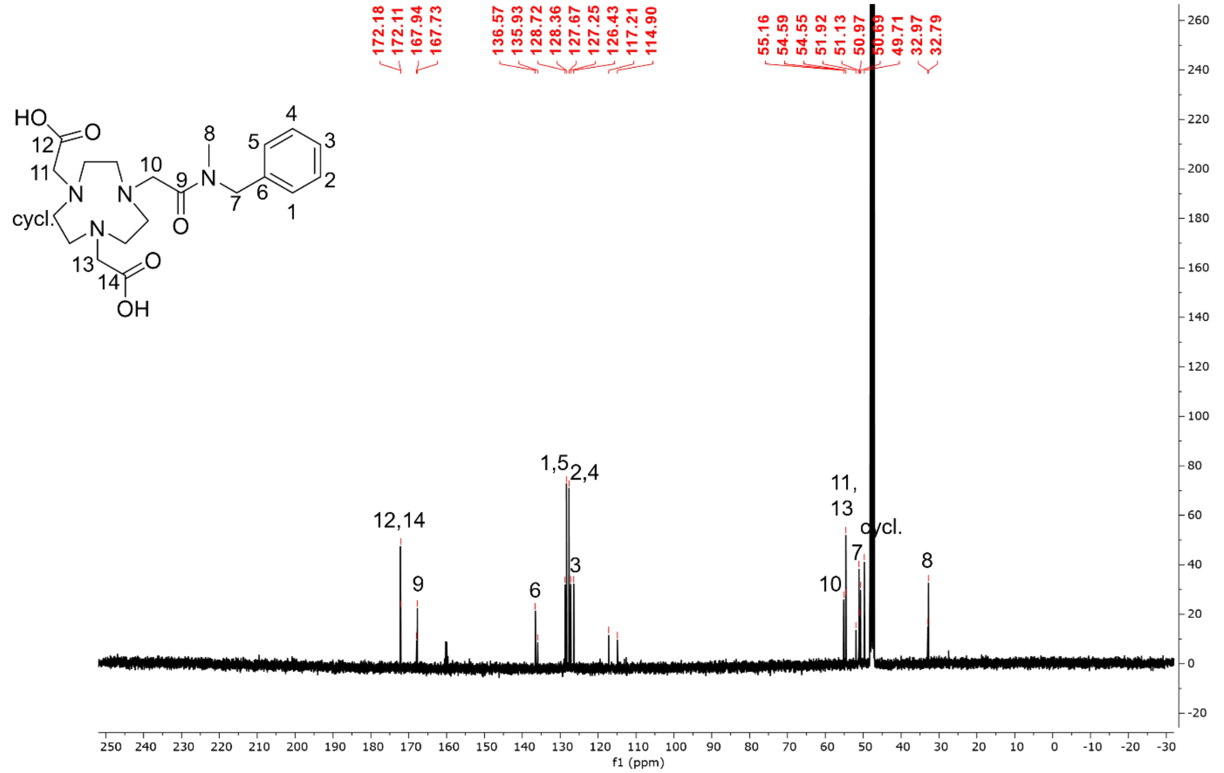

Figure S107. ^{13}C NMR (126 MHz, MeOD) for compound **27**.

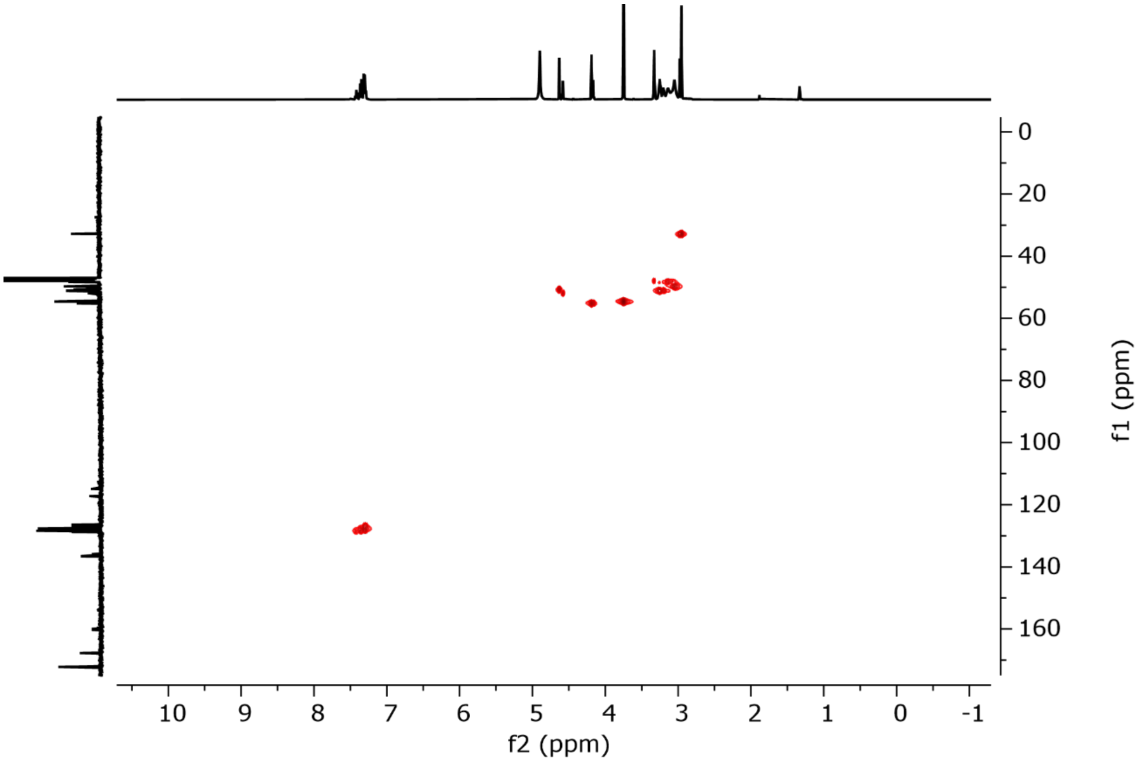

Figure S108. The HSQC NMR spectrum for compound **27**.

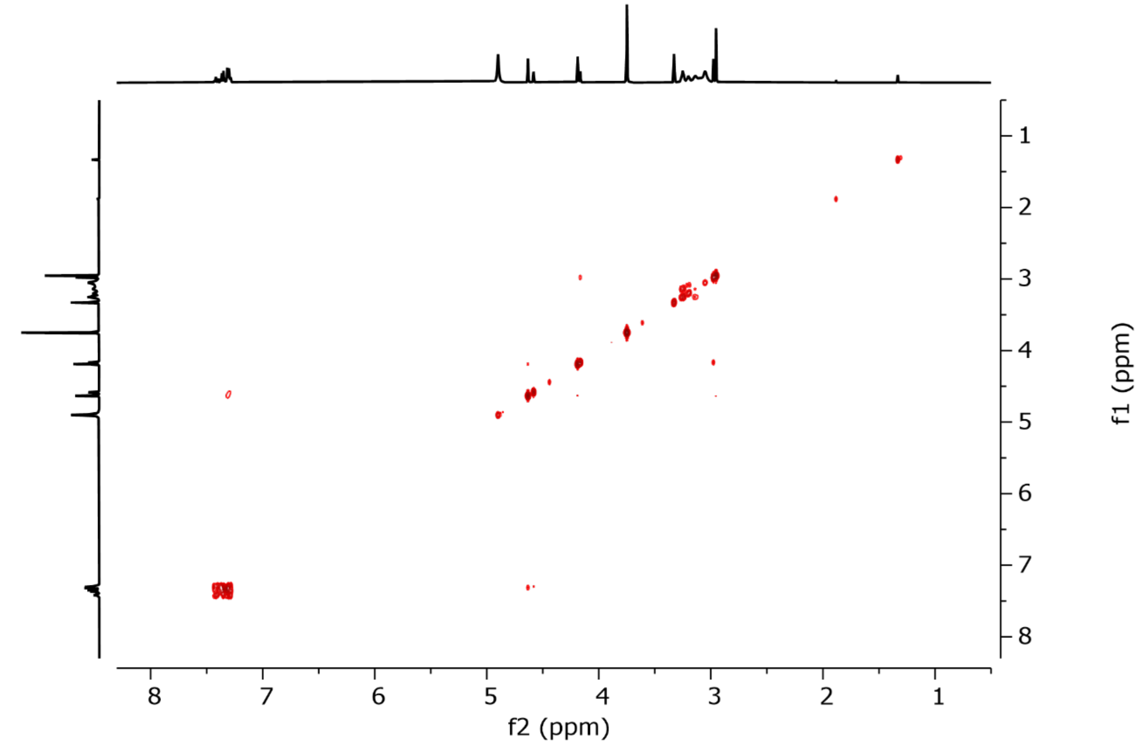

Figure S109. The ^{1}H-^{1}H COSY NMR spectrum for compound **27**.

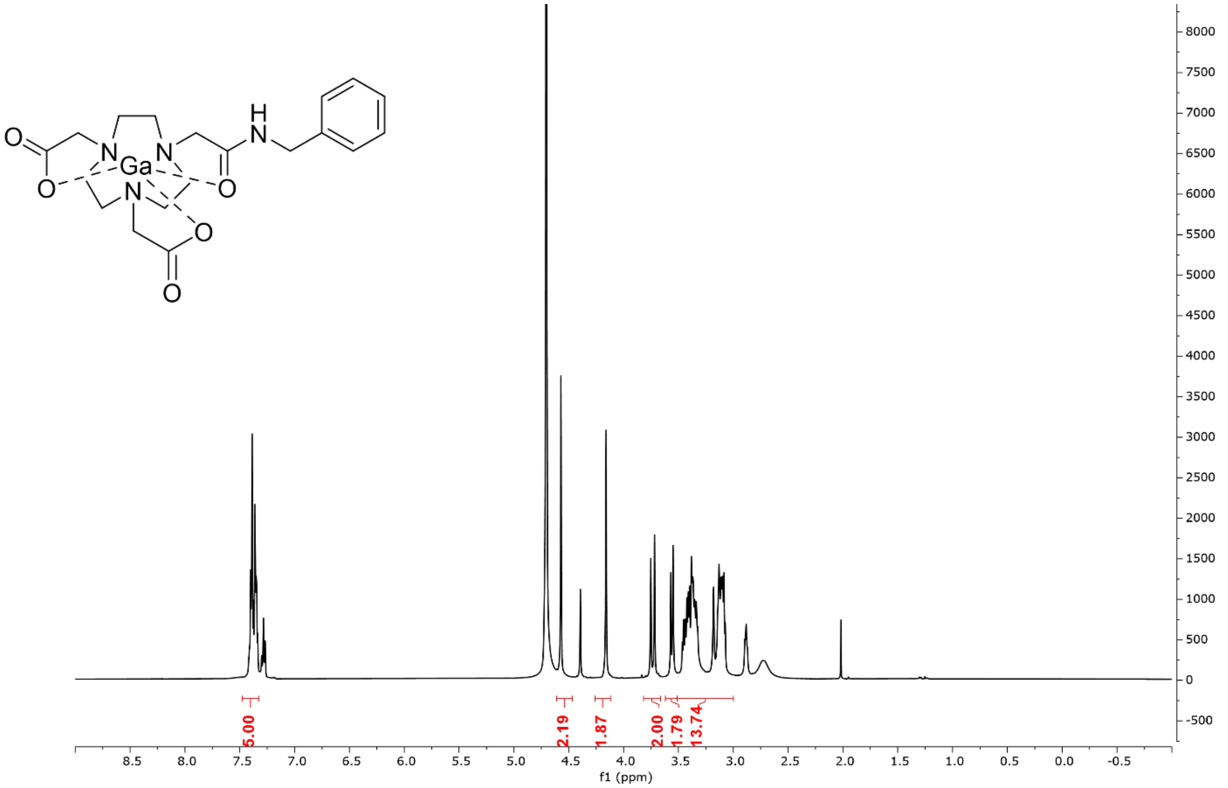

Figure S110. ^{1}H NMR (500 MHz, D_2O) for compound **[Ga(26)]$^+$** at pH 2.

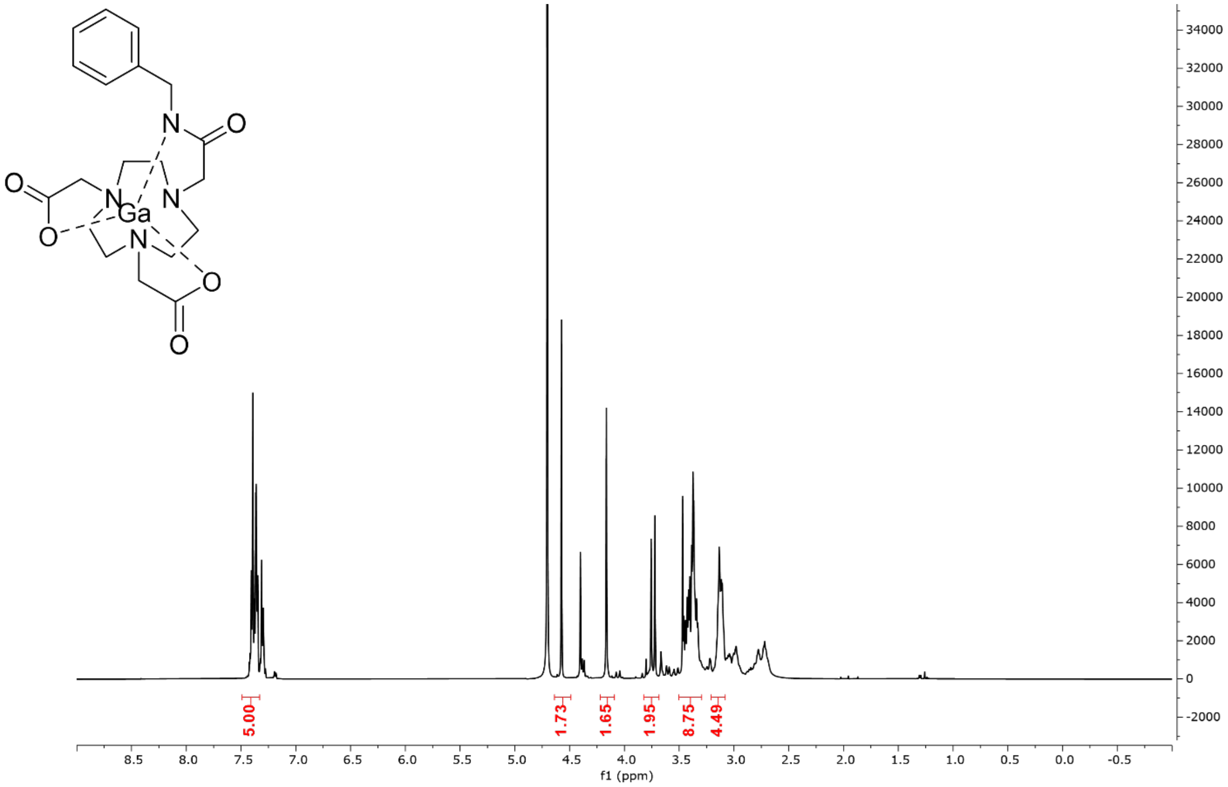

Figure S111. ^{1}H NMR (500 MHz, D_2O) for compound **Ga(26)** at pH 7.

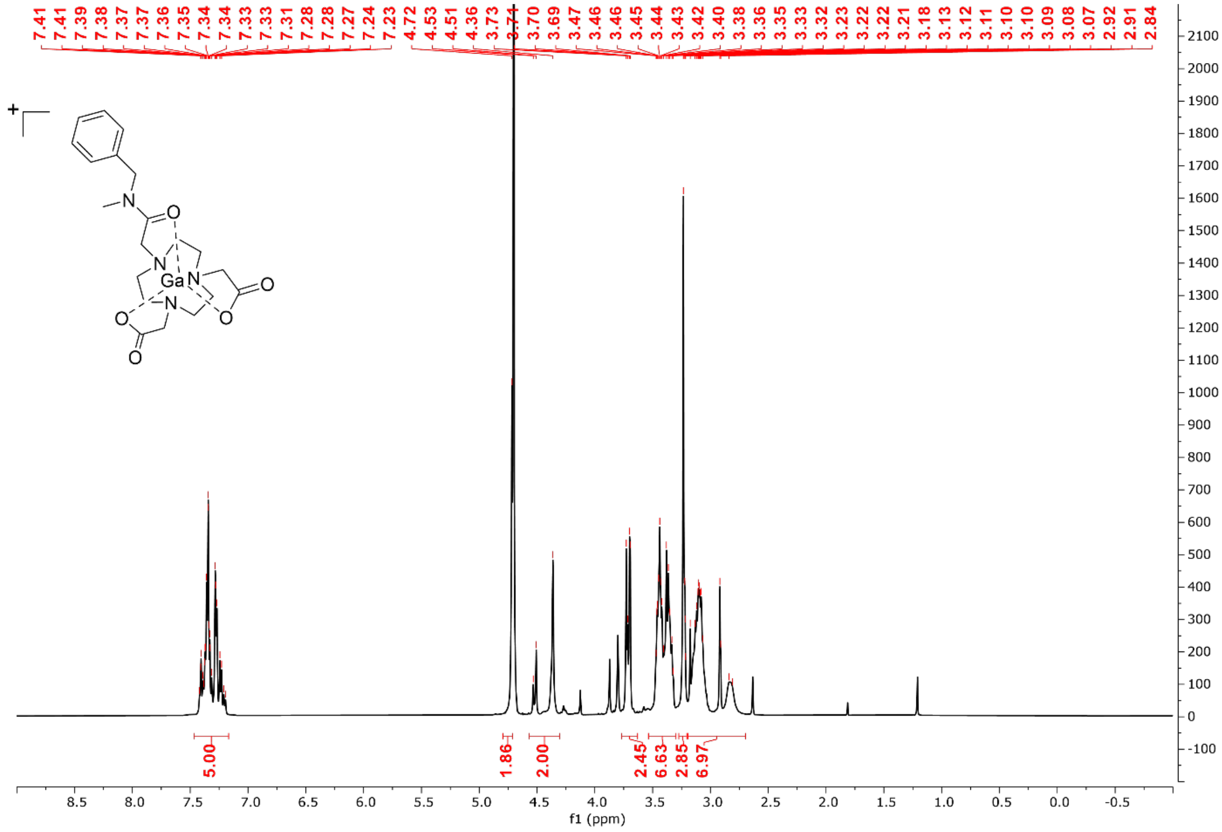

Figure S112. ^{1}H NMR (500 MHz, D_2O) for compound **[Ga(27)]**$^{+}$ at pH 4.

2.6 Cleavage Assay for Ligands

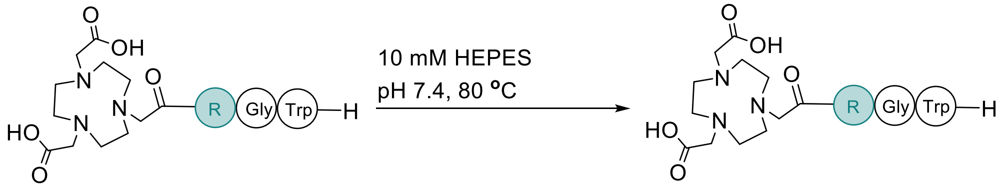

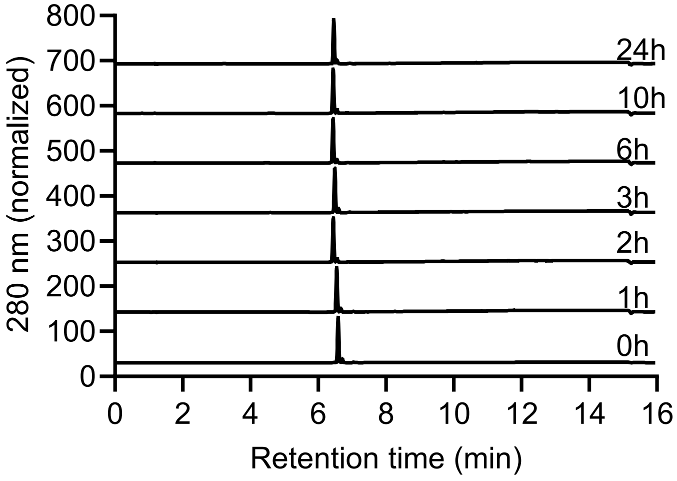

Figure S113. Schematic description of incubating ligands at pH 7.4 and 80 °C. Analytical HPLC chromatograms show no autolytic release of NOTA during compound **10** incubation.

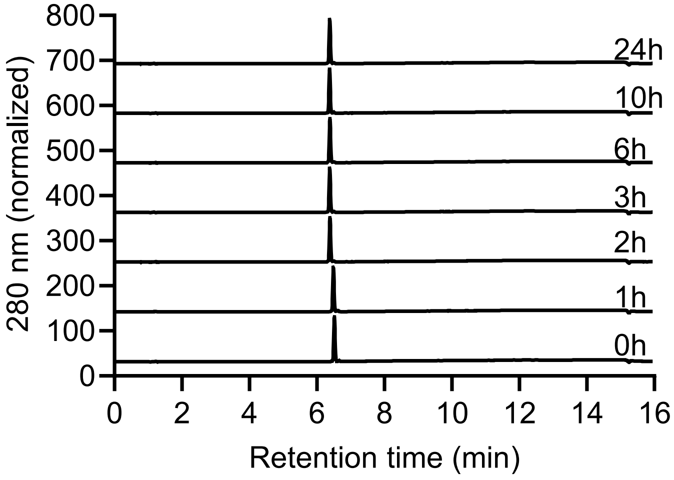

Figure S114. Analytical HPLC chromatograms show no autolytic release of NOTA during compound **11** incubation.

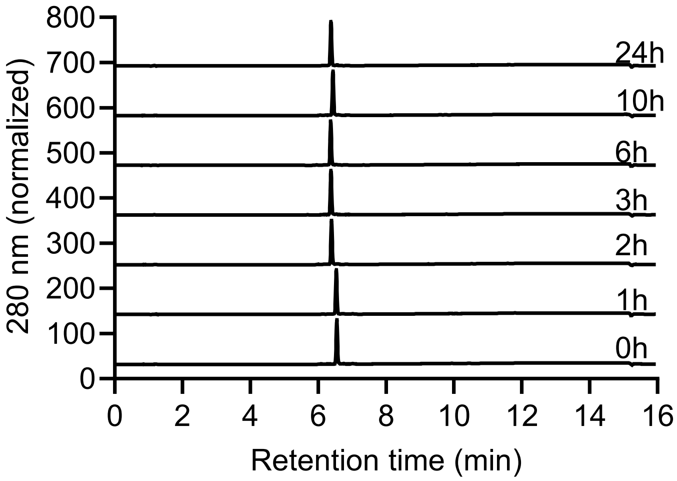

Figure S115. Analytical HPLC chromatograms show no autolytic release of NOTA during compound **12** incubation.

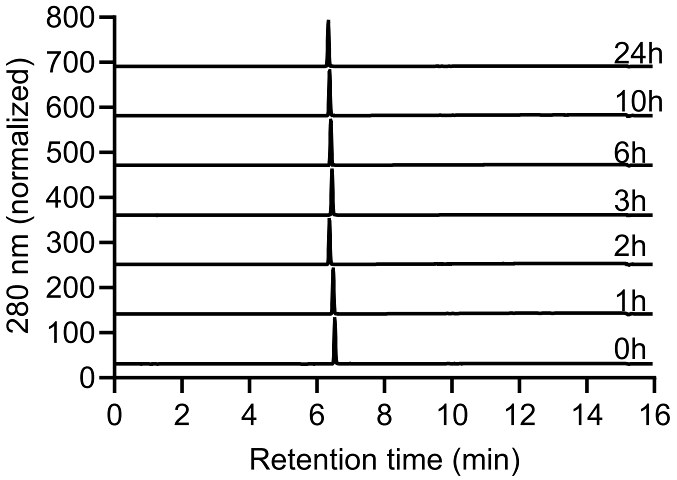

Figure S116. Analytical HPLC chromatograms show no autolytic release of NOTA during compound **13** incubation.

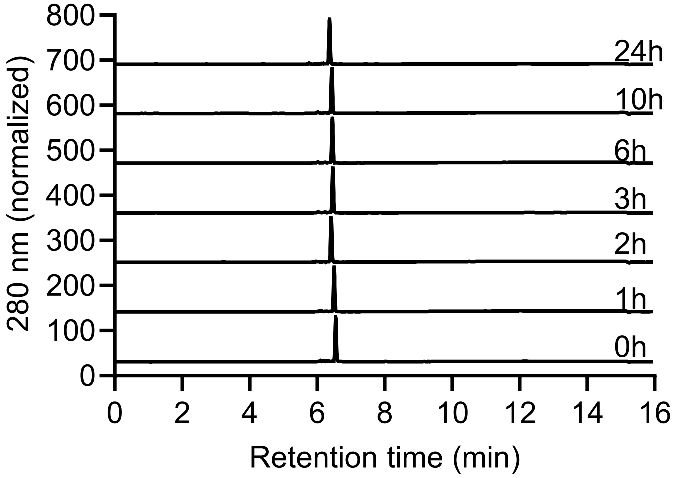

Figure S117. Analytical HPLC chromatograms show no autolytic release of NOTA during compound **14** incubation.

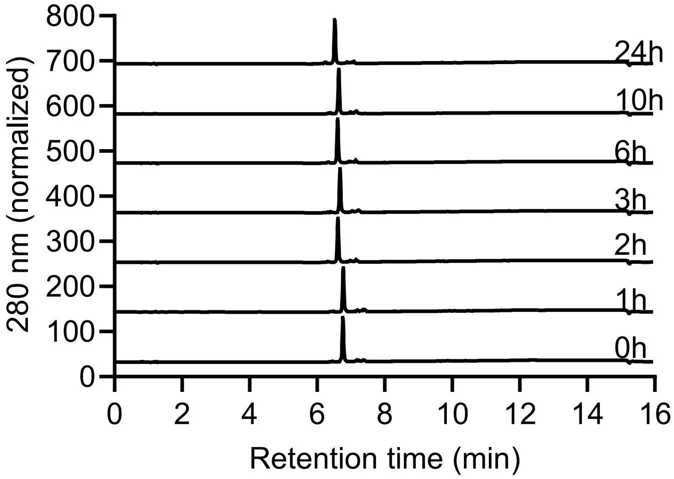

Figure S118. Analytical HPLC chromatograms show no autolytic release of NOTA during compound **15** incubation.

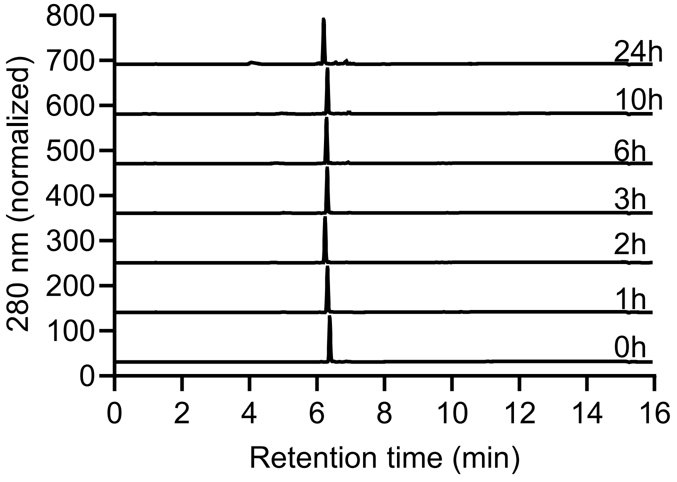

Figure S119. Analytical HPLC chromatograms show no autolytic release of NOTA during compound **16** incubation.

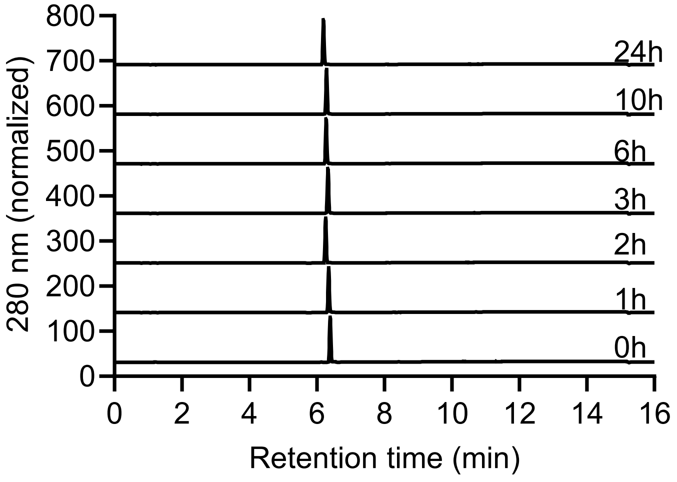

Figure S120. Analytical HPLC chromatograms show no autolytic release of NOTA during compound **17** incubation.

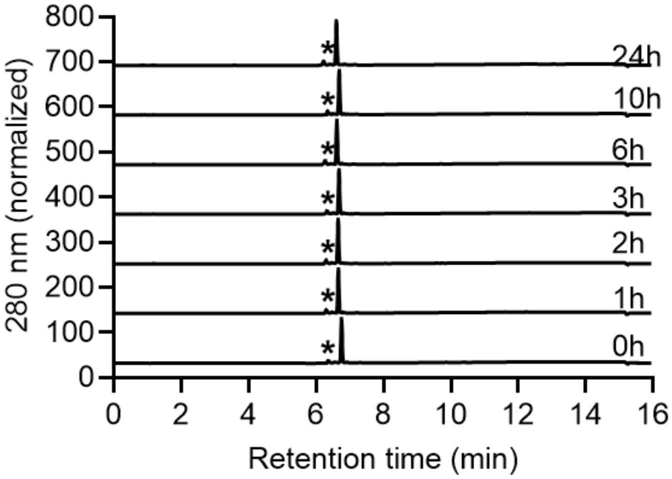

Figure S121. Analytical HPLC chromatograms showing no autolytic release of NOTA during compound **18** incubation. The asterisk * symbol denotes a minor chromatographic impurity.

3 Complex synthesis and characterization

3.1 General Complexation Protocol and Cleavage Assay Procedure

A 5 mg aliquot of the compound, **10-25,** was dissolved in 500 µL of room-temperature distilled water. Two equivalents of the $^{nat}Ga^{3+}$salt were added to the solution, which was left to react for 1 hour under room temperature. Full complexation was confirmed by LCMS. The complex was then purified via Sep-Pak C18 short cartridge. The concentration was determined by ICP-OES. The complexes were further characterized via HPLC and HR-ESI-MS.

Cleavage of the cold model tripeptide complexes (0.75 mM, determined by ICP-OES) was observed under 80 ºC and pH 7.4. Aqueous buffer solutions were prepared using 10 mM NaOAc (pH 4.5), 10 mM MOPS (pH 6.5), 10 mM HEPES (pH 7.4), and 10 mM EPPS (pH 8.5) which were subsequently adjusted to the desired pH using NaOH and/or HCl. The complex was formed in pH 4.5 10 mM NaOAc buffer under room temperature. An aliquot of the complex was placed in an aqueous solution of the desired pH and incubated for 24 hours at 80 ºC. The reaction was monitored via analytical HPLC (Method A).

3.2 Complexation Protocol for Model Ligands

^{nat}Gallium 2,2'-(7-(2-(benzylamino)-2-oxoethyl)-1,4,7-triazonane-1,4-diyl)diacetic acid, **[Ga(26)]ClO$_4$**. A 15 mg aliquot of compound **26** was dissolved in 500 µL of room temperature distilled water. The concentration of **26** was determined by UV-Vis using the extinction coefficient (299.7 M^{-1} cm^{-1} at 257 nm) previously reported.[2] One equivalent of gallium(III) perchlorate hydrate, Ga(ClO4)$_3$, was added to the solution. The concentration of $Ga(ClO_4)_3$ solution was determined by ICP-OES. Crystals suitable for single crystal X-ray diffraction were grown from slow evaporation of the aqueous reaction solution.

^{nat}Gallium 2,2'-(7-(2-(benzylamino)-2-oxoethyl)-1,4,7-triazonane-1,4-diyl)diacetic acid, **Ga(26)**. A 15 mg aliquot of compound **26** was dissolved in 500 µL of room temperature distilled water. The concentration of **26** was determined by UV-Vis (299.7 M^{-1} cm^{-1} at 257 nm).[2] One equivalent of gallium(III) nitrate hydrate, $Ga(NO_3)_3$, was added to the solution. The concentration of $Ga(NO_3)_3$ was determined by ICP-OES. The pH of the solution was adjusted to pH = 5 by adding 4 equivalents of 1 M KOH solution. The complexation was confirmed by ^{1}H NMR and ^{13}C NMR. Crystals suitable for single crystal X-ray diffraction were grown from slow evaporation of the aqueous reaction solution.

^{nat}Gallium 2,2'-(7-(2-(benzyl(methyl)amino)-2-oxoethyl)-1,4,7-triazonane-1,4-diyl)diacetic acid, **[Ga(27)]ClO$_4$**. A 15 mg aliquot of compound **27** was dissolved in 500 µL of room temperature distilled water. The concentration of **27** was determined by UV-Vis (299.7 M^{-1} cm^{-1} at 257 nm).[2] One equivalent of $Ga(ClO_4)_3$ was added to the solution. The concentration of $Ga(ClO_4)_3$ solution was determined by ICP-OES. The pH of the solution was adjusted to pH = 5 by adding 4 equivalents of 1 M KOH solution. The complexation was confirmed by ^{1}H NMR and ^{13}C NMR. Crystals suitable for single crystal X-ray diffraction were grown from adding MeOH into sample dissolved in H_2O (layering method in NMR tube).

3.3 HRMS, HR-ESI-MS and HPLC Data for Coordination Complexes

natGallium (R)-2,2'-(7-(2-((2-((2-((1-amino-3-(1H-indol-3-yl)-1-oxopropan-2-yl)amino)-2-oxoethyl)amino)-2-oxoethyl)amino)-2-oxoethyl)-1,4,7-triazonane-1,4-diyl)diacetate, **[natGa(10)]+**. Complex **[natGa(10)]+** was synthesized using the general complexation protocol outlined in **Section 3.1** from compound **10** and $Ga(NO_3)_3 \cdot 3H_2O$. The complex was purified using a Sep-Pak Plus C18 short cartridge and characterized by HPLC chromatography and mass spectrometry. R_t (Method B): 6.37 min. ESI-MS $[M]^+$ calc. for $C_{27}H_{36}GaN_8O_8$ 669.1906, found 669.1901.

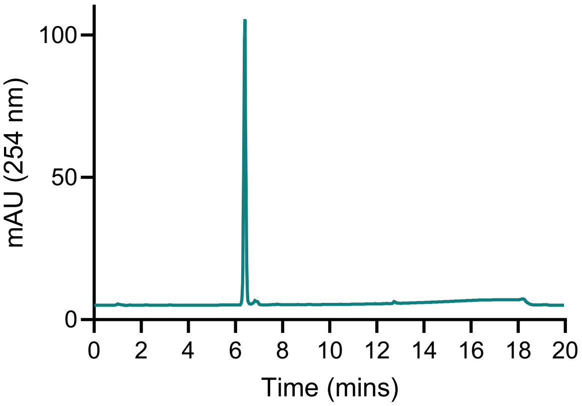

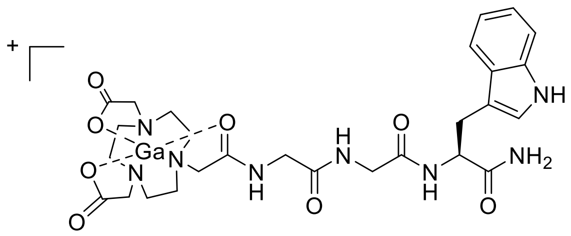

Chemical Formula: $C_{27}H_{36}GaN_8O_8$
Exact Mass: 669.19
Molecular Weight: 670.36

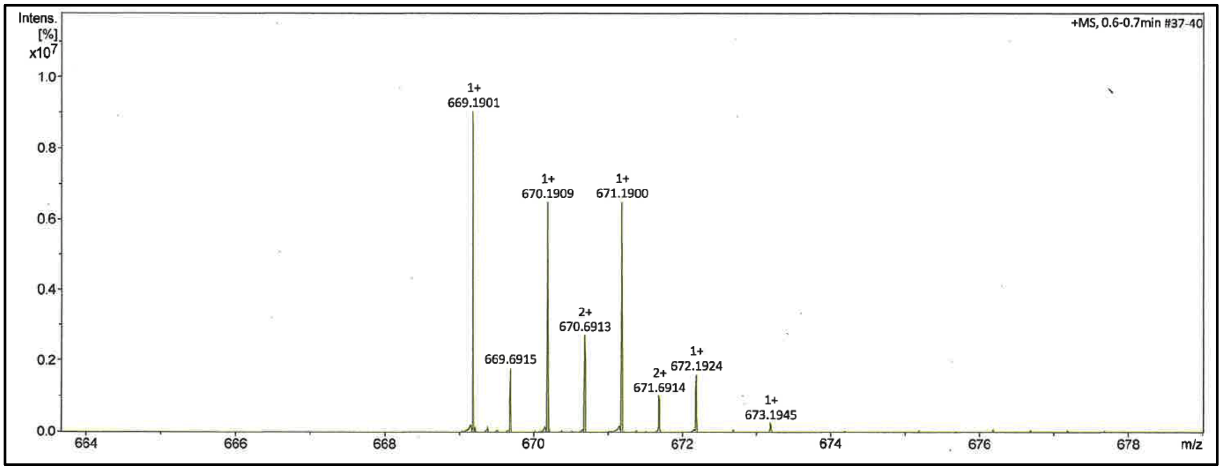

Figure S122. LC-MS data for compound **[natGa(10)]+**.

^{nat}*Zinc (R)-2,2'-(7-(2-((2-((2-((1-amino-3-(1H-indol-3-yl)-1-oxopropan-2-yl)amino)-2-oxoethyl)amino)-2-oxoethyl)amino)-2-oxoethyl)-1,4,7-triazonane-1,4-diyl)diacetate*, **[^{nat}Zn(10)]**. Complex **[^{nat}Zn(10)]** was synthesized using the general complexation protocol outlined in **Section 3.1** from compound **10** and $ZnSO_4 \cdot 7H_2O$. The complex was purified using a Sep-Pak Plus C18 short cartridge and characterized by HPLC chromatography and mass spectrometry. R_t (Method A): 6.22 min. ESI-MS $[M+H]^+$ calc. for $C_{27}H_{36}N_8O_8Zn$ 665.2020 found 665.2016.

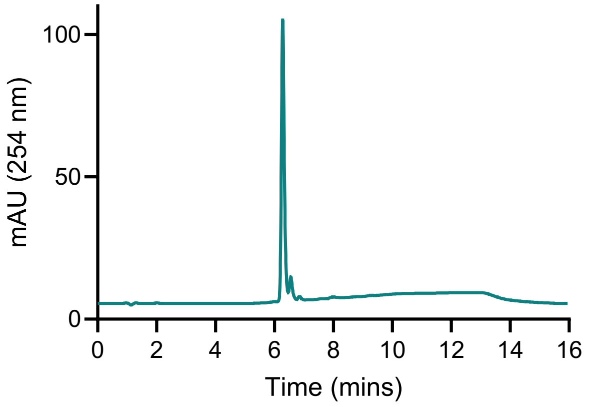

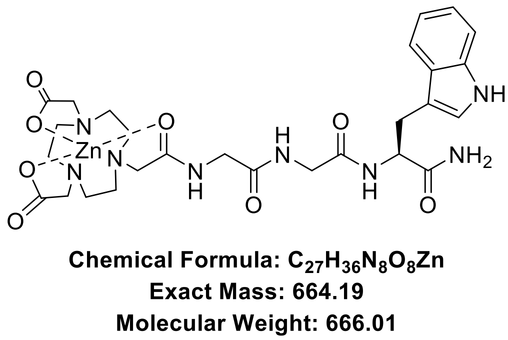

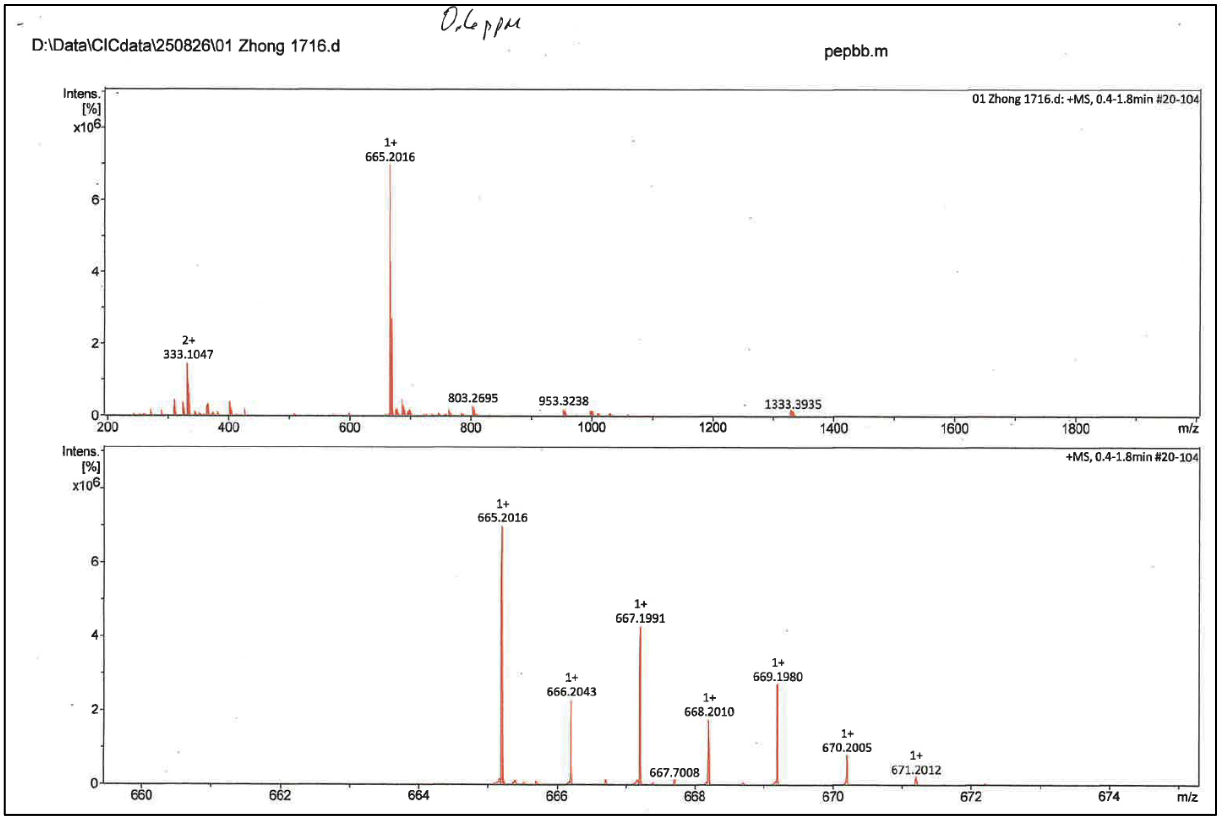

Figure S123. LC-MS data for compound **[^{nat}Zn(10)]**.

natIron (R)-2,2'-(7-(2-((2-((2-((1-amino-3-(1H-indol-3-yl)-1-oxopropan-2-yl)amino)-2-oxoethyl)amino)-2-oxoethyl)amino)-2-oxoethyl)-1,4,7-triazonane-1,4-diyl)diacetate, **[^{nat}Fe(10)]$^+$**. Complex **[^{nat}Fe(10)]$^+$** was synthesized using the general complexation protocol outlined in **Section 3.1** from compound **10** and $FeCl_3$. The complex was purified using a Sep-Pak Plus C18 short cartridge and characterized by HPLC chromatography and mass spectrometry. R_t (Method A): 5.98 min. ESI-MS $[M+H]^+$ calc. for $C_{27}H_{36}FeN_8O_8$ 656.2000 found 656.1971.

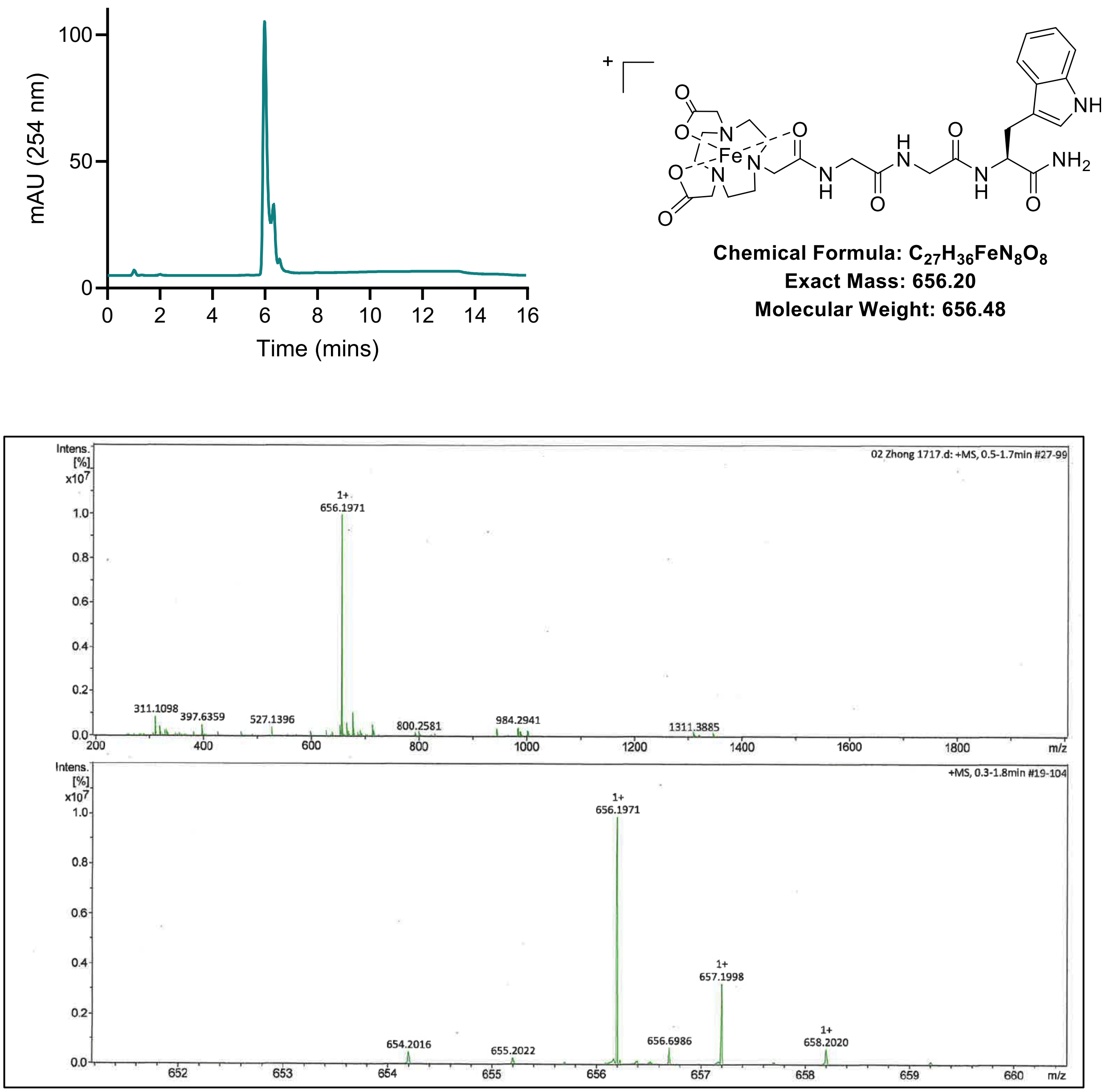

Figure S124. LC-MS data for compound **[^{nat}Fe(10)]$^+$**.

[nat]Indium (R)-2,2'-(7-(2-((2-((2-((1-amino-3-(1H-indol-3-yl)-1-oxopropan-2-yl)amino)-2-oxoethyl)amino)-2-oxoethyl)amino)-2-oxoethyl)-1,4,7-triazonane-1,4-diyl)diacetate, **$[^{nat}In(10)]^+$**. Complex **$[^{nat}In(10)]^+$** was synthesized using the general complexation protocol outlined in **Section 3.1** from compound **10** and $InCl_3$. The complex was purified using a Sep-Pak Plus C18 short cartridge and characterized by HPLC chromatography and mass spectrometry. R_t (Method A): 6.15 min. ESI-MS $[M+H]^+$ calc. for $C_{27}H_{36}InN_8O_8$ 715.1690 found 715.1684.

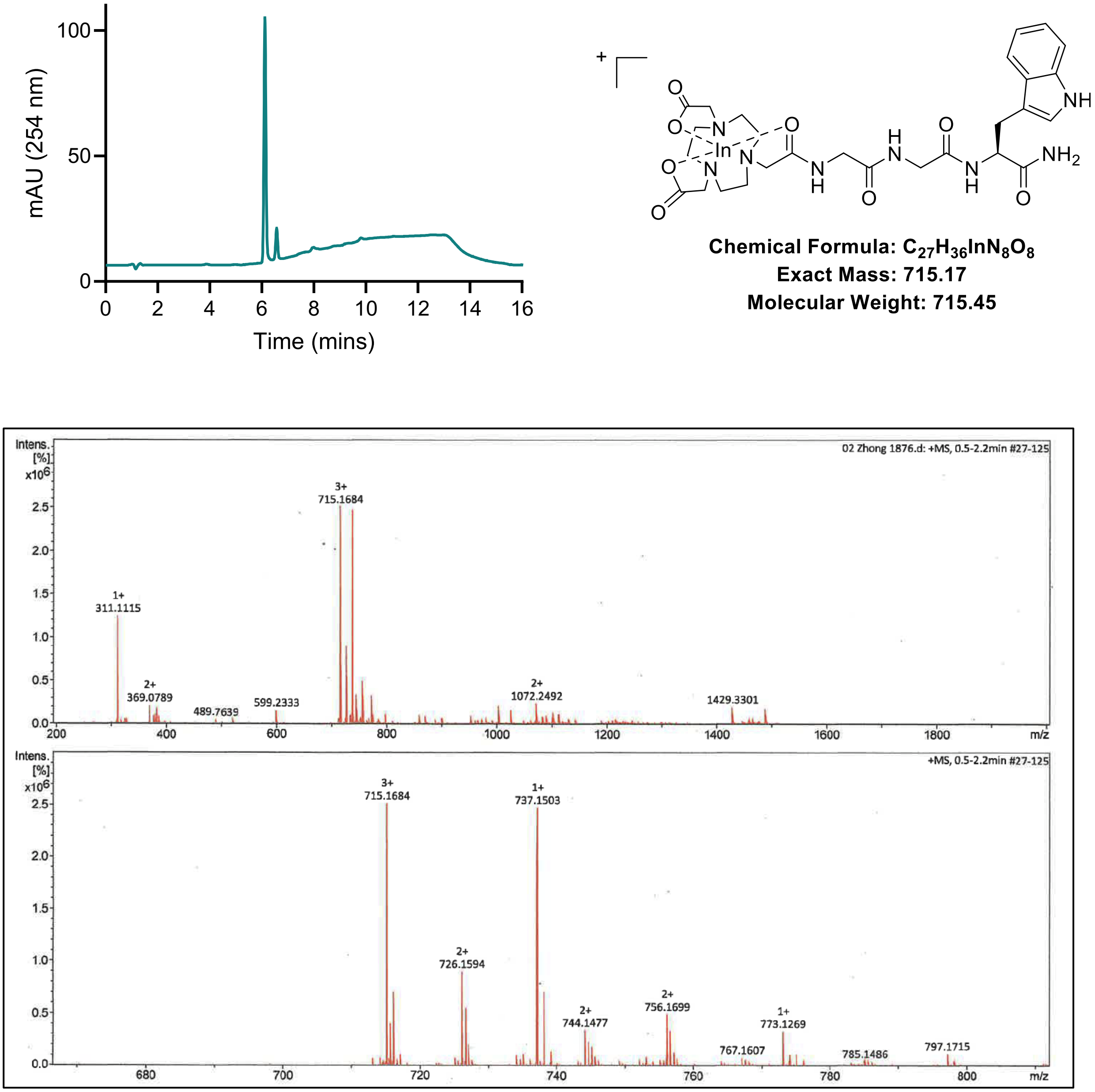

Figure S125. LC-MS data for compound **$[^{nat}In(10)]^+$**.

[nat]Gallium (S)-2,2'-(7-(2-((3-((2-((1-amino-3-(1H-indol-3-yl)-1-oxopropan-2-yl)amino)-2-oxoethyl)amino)-3-oxopropyl)amino)-2-oxoethyl)-1,4,7-triazonane-1,4-diyl)diacetate, **[^{nat}Ga(11)]$^+$**. Complex **[^{nat}Ga(11)]$^+$** was synthesized using the general complexation protocol outlined in **Section 3.1** from compound **11** and $Ga(NO_3)_3 \cdot 3H_2O$. The complex was purified using a Sep-Pak Plus C18 short cartridge and characterized by HPLC chromatography and mass spectrometry. R_t (Method B): 6.43 min. ESI-MS $[M]^+$ calc. for $C_{28}H_{38}GaN_8O_8$ 683.2063, found 683.2056.

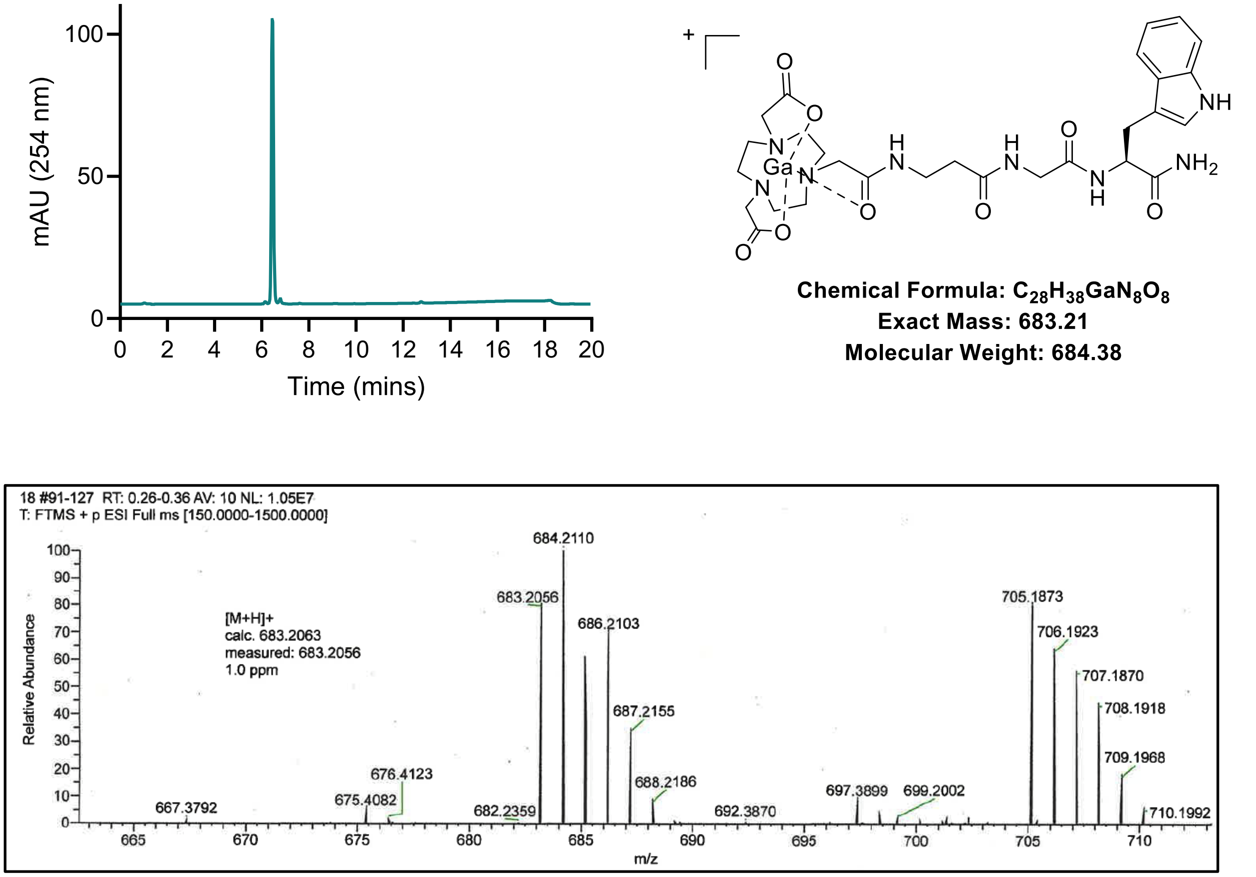

Figure S126. LC-MS data for compound **[^{nat}Ga(11)]$^+$**.

^{nat}Gallium 2,2'-(7-(2-(((R)-1-((2-(((S)-1-amino-3-(1H-indol-3-yl)-1-oxopropan-2-yl)amino)-2-oxoethyl)amino)-1-oxopropan-2-yl)amino)-2-oxoethyl)-1,4,7-triazonane-1,4-diyl)diacetate, **[^{nat}Ga(12)]$^+$**. Complex **[^{nat}Ga(12)]$^+$** was synthesized using the general complexation protocol outlined in **Section 3.1** from compound **12** and $Ga(NO_3)_3 \cdot 3H_2O$. The complex was purified using a Sep-Pak Plus C18 short cartridge and characterized by HPLC chromatography and mass spectrometry. R_t (Method B): 6.54 min. ESI-MS $[M]^+$ calc. for $C_{28}H_{38}GaN_8O_8$ 683.2063, found 683.2060.

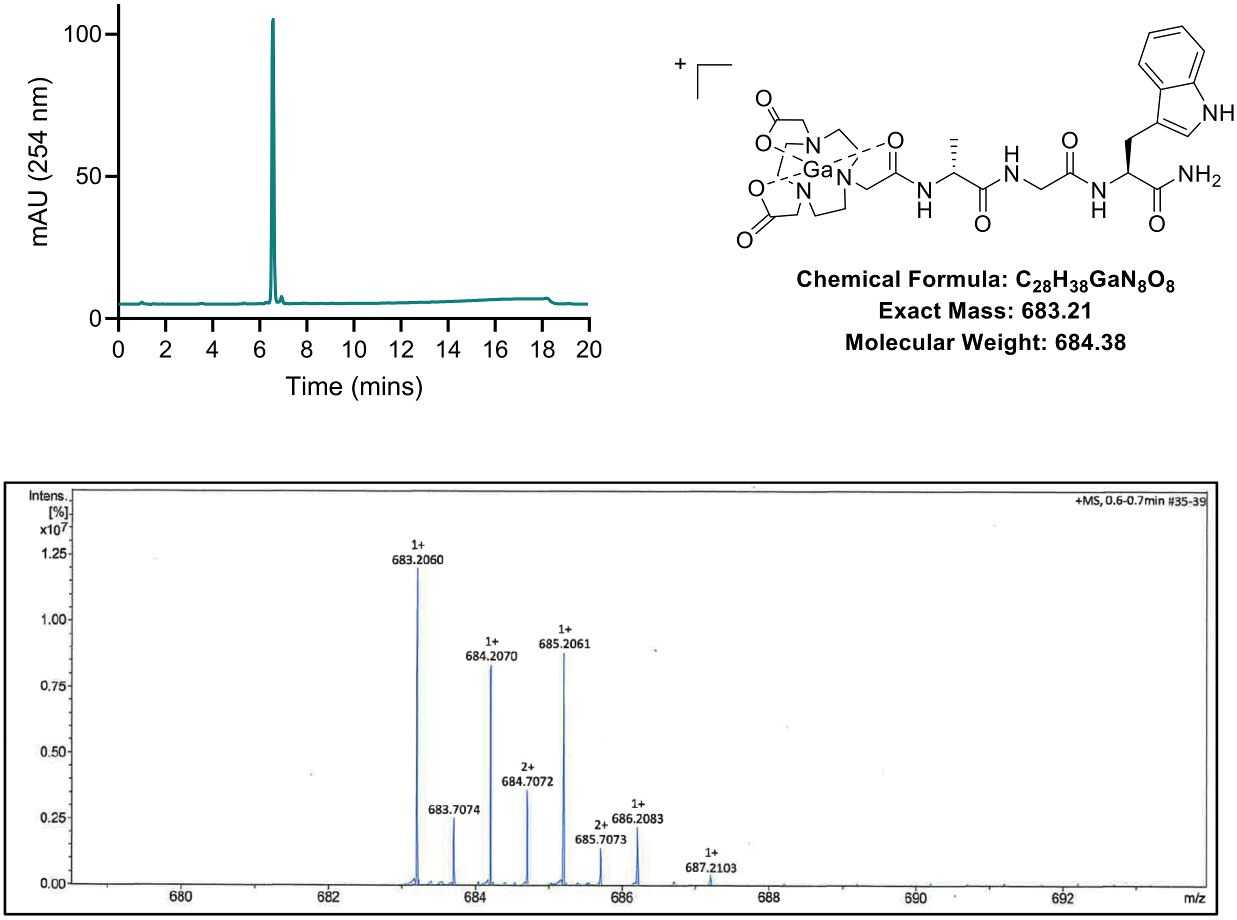

Figure S127. LC-MS data for compound **[^{nat}Ga(12)]$^+$**.

natGallium 2,2'-(7-(2-(((R)-1-((2-(((S)-1-amino-3-(1H-indol-3-yl)-1-oxopropan-2-yl)amino)-2-oxoethyl)amino)-3-hydroxy-1-oxopropan-2-yl)amino)-2-oxoethyl)-1,4,7-triazonane-1,4-diyl)diacetate, **[^{nat}Ga(13)]$^+$**. Complex **[^{nat}Ga(13)]$^+$** was synthesized using the general complexation protocol outlined in **Section 3.1** from compound **13** and $Ga(NO_3)_3 \cdot 3H_2O$. The complex was purified using a Sep-Pak Plus C18 short cartridge and characterized by HPLC chromatography and mass spectrometry. R_t (Method B): 6.43 min. ESI-MS $[M]^+$ calc. for $C_{28}H_{38}GaN_8O_9$ 699.2012, found 699.2009.

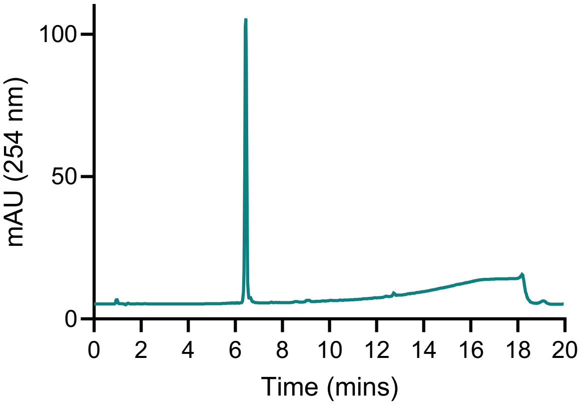

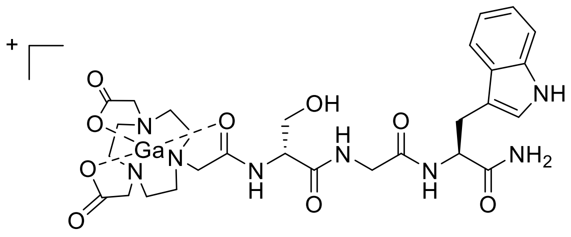

Chemical Formula: $C_{28}H_{38}GaN_8O_9$
Exact Mass: 699.20
Molecular Weight: 700.38

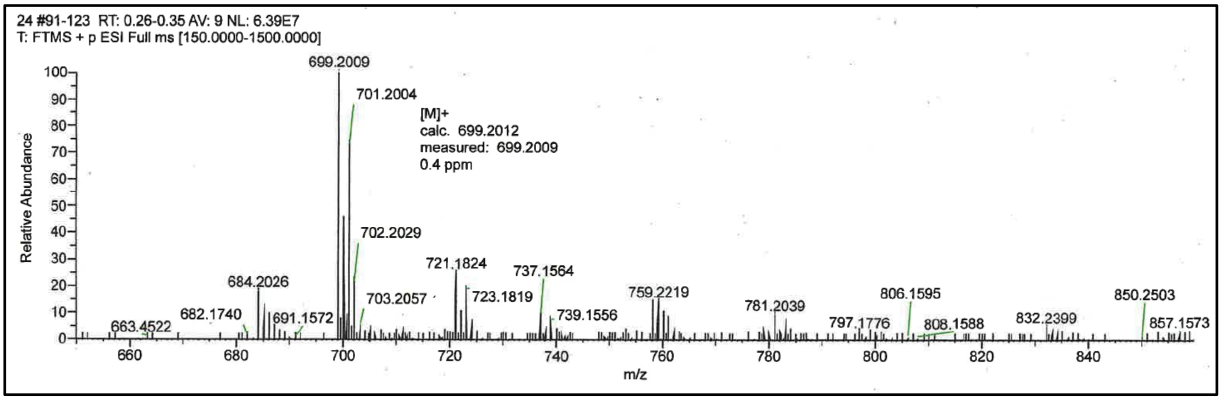

Figure S128. LC-MS data for compound **[^{nat}Ga(13)]$^+$**.

^{nat}Gallium 2,2'-(7-(2-(((2R,3S)-1-((2-(((S)-1-amino-3-(1H-indol-3-yl)-1-oxopropan-2-yl)amino)-2-oxoethyl)amino)-3-hydroxy-1-oxobutan-2-yl)amino)-2-oxoethyl)-1,4,7-triazonane-1,4-diyl)diacetate, **[^{nat}Ga(14)]$^+$**. Complex **[^{nat}Ga(14)]$^+$** was synthesized using the general complexation protocol outlined in **Section 3.1** from compound **14** and $Ga(NO_3)_3 \cdot 3H_2O$. The complex was purified using a Sep-Pak Plus C18 short cartridge and characterized by HPLC chromatography and mass spectrometry. R_t (Method B): 6.50 min. ESI-MS $[M]^+$ calc. for $C_{28}H_{38}GaN_8O_9$ 713.2169, found 713.2173.

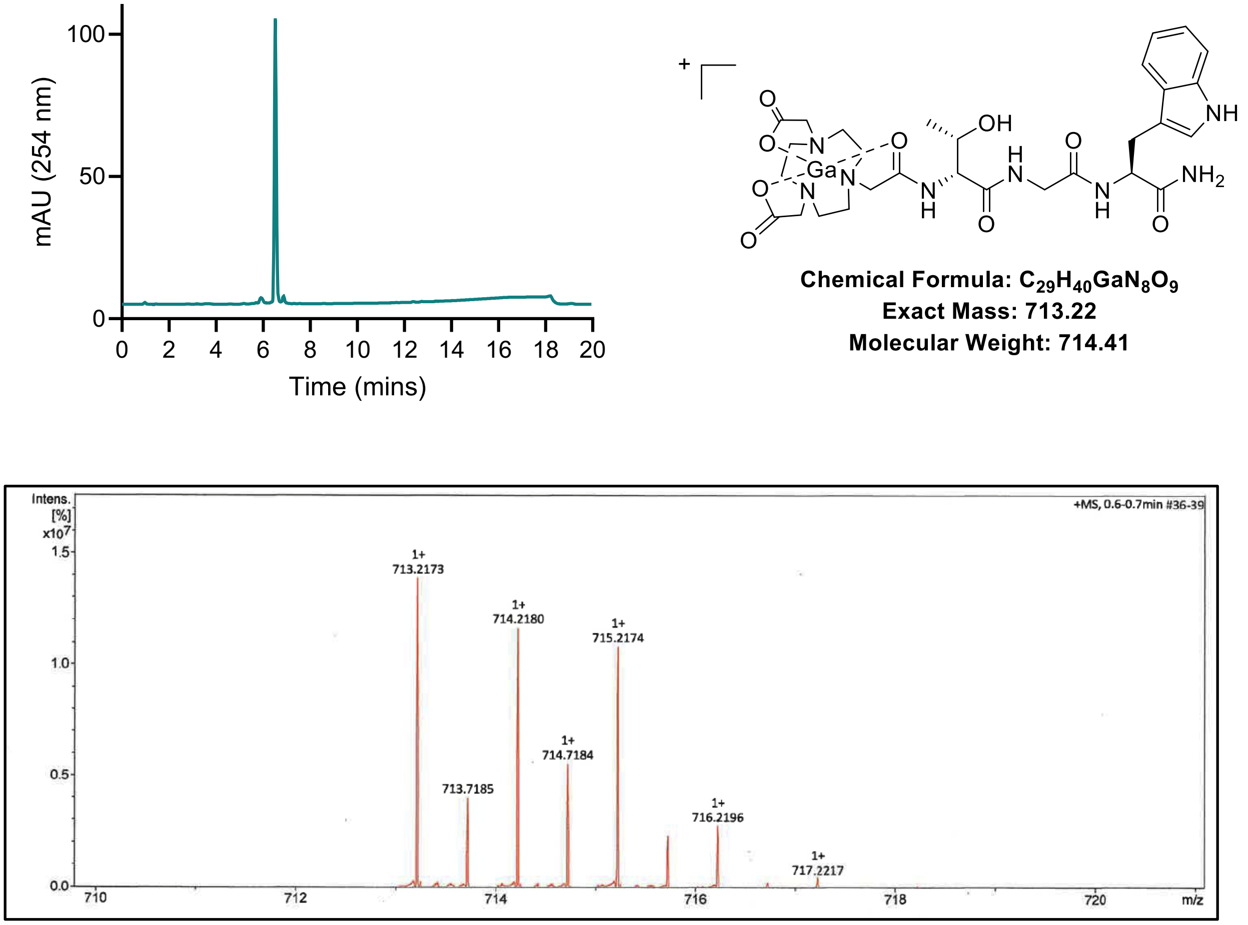

Figure S129. LC-MS data for compound **[^{nat}Ga(14)]$^+$**.

natGallium 2,2'-(7-(2-(((R)-1-((2-(((S)-1-amino-3-(1H-indol-3-yl)-1-oxopropan-2-yl)amino)-2-oxoethyl)amino)-4-(methylthio)-1-oxobutan-2-yl)amino)-2-oxoethyl)-1,4,7-triazonane-1,4-diyl)diacetate, **[natGa(15)]**$^+$. Complex **[natGa(15)]**$^+$ was synthesized using the general complexation protocol outlined in **Section 3.1** from compound **15** and $Ga(NO_3)_3 \cdot 3H_2O$. The complex was purified using a Sep-Pak Plus C18 short cartridge and characterized by HPLC chromatography and mass spectrometry. R_t (Method B): 7.20 min. ESI-MS $[M]^+$ calc. for $C_{30}H_{42}GaN_8O_8S$ 743.2097, found 743.2096.

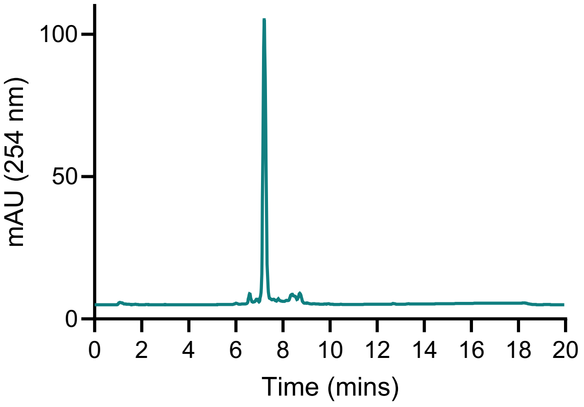

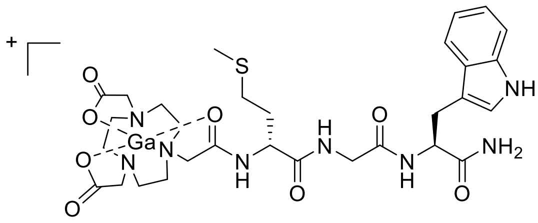

Chemical Formula: $C_{30}H_{42}GaN_8O_8S$
Exact Mass: 743.21
Molecular Weight: 744.50

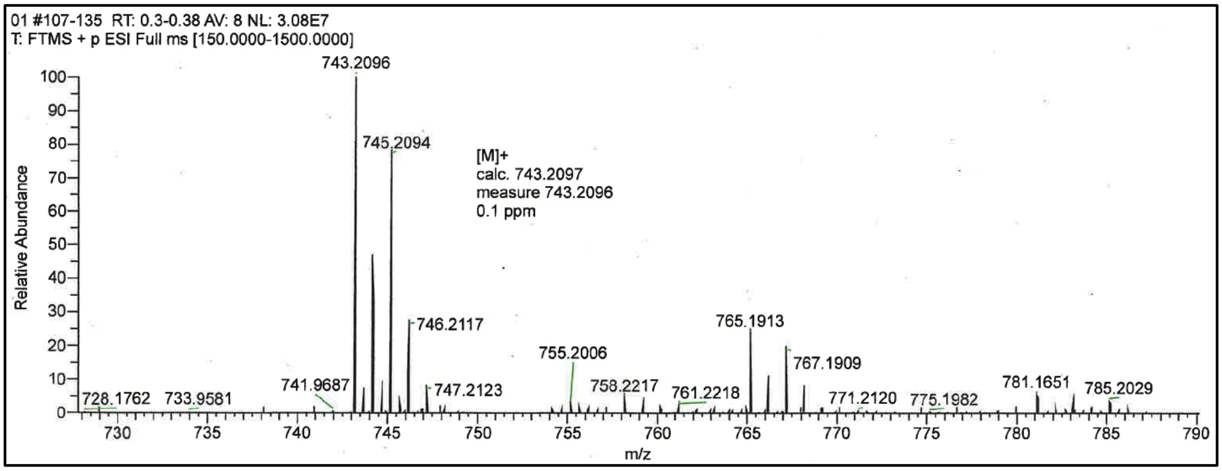

Figure S130. LC-MS data for compound **[natGa(15)]**$^+$.

[nat]Gallium 2,2'-(7-(2-(((R)-1-((2-(((S)-1-amino-3-(1H-indol-3-yl)-1-oxopropan-2-yl)amino)-2-oxoethyl)amino)-3-hydroxy-1-oxopropan-2-yl)(methyl)amino)-2-oxoethyl)-1,4,7-triazonane-1,4-diyl)diacetate, **[^{nat}Ga(16)]$^+$**. Complex **[^{nat}Ga(16)]$^+$** was synthesized using the general complexation protocol outlined in **Section 3.1** from compound **16** and $Ga(NO_3)_3 \cdot 3H_2O$. The complex was purified using a Sep-Pak Plus C18 short cartridge and characterized by HPLC chromatography and mass spectrometry. R_t (Method B): 6.43 min. ESI-MS $[M]^+$ calc. for $C_{29}H_{40}GaN_8O_9$ 713.21685, found 713.21481.

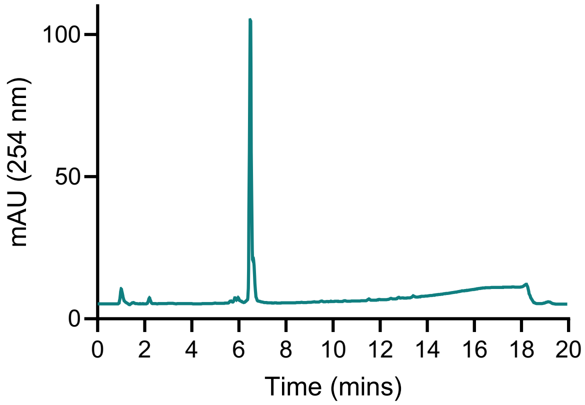

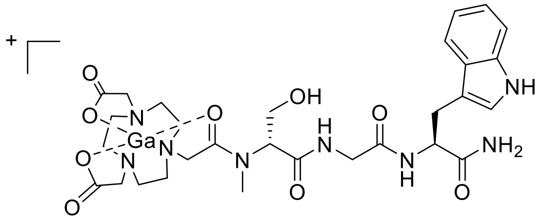

Chemical Formula: $C_{29}H_{40}GaN_8O_9$
Exact Mass: 713.22
Molecular Weight: 714.41

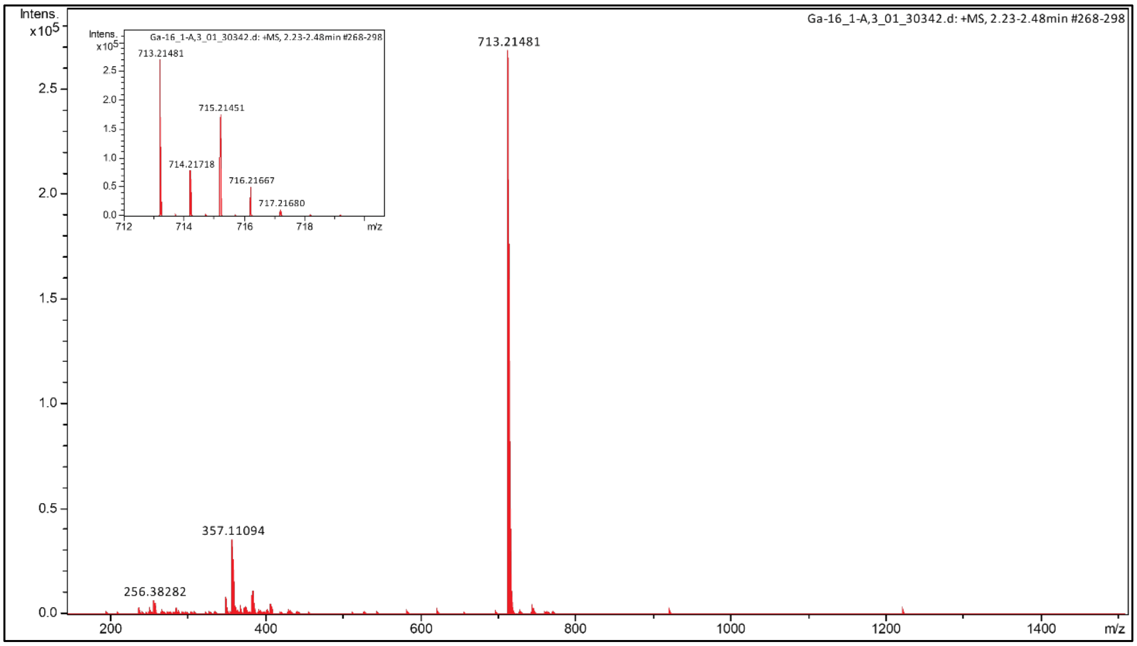

Figure S131. LC-MS data for compound **[^{nat}Ga(16)]$^+$**.

natGallium (S)-2,2'-(7-(2-((2-((2-((1-amino-3-(1H-indol-3-yl)-1-oxopropan-2-yl)amino)-2-oxoethyl)amino)-2-oxoethyl)(methyl)amino)-2-oxoethyl)-1,4,7-triazonane-1,4-diyl)diacetate, **[^{nat}Ga(17)]$^{+}$**. Complex **[^{nat}Ga(17)]$^{+}$** was synthesized using the general complexation protocol outlined in **Section 3.1** from compound **17** and $Ga(NO_3)_3 \cdot 3H_2O$. The complex was purified using a Sep-Pak Plus C18 short cartridge and characterized by HPLC chromatography and mass spectrometry. R_t (Method B): 6.39 min. ESI-MS $[M]^+$ calc. for $C_{28}H_{38}GaN_8O_8$ 683.20628, found 683.20481.

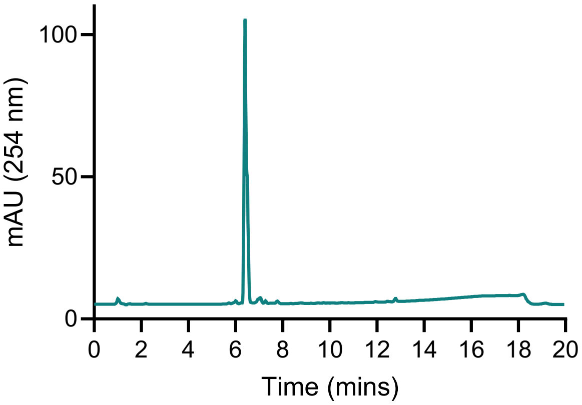

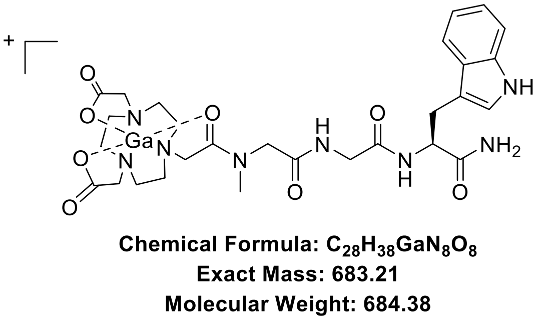

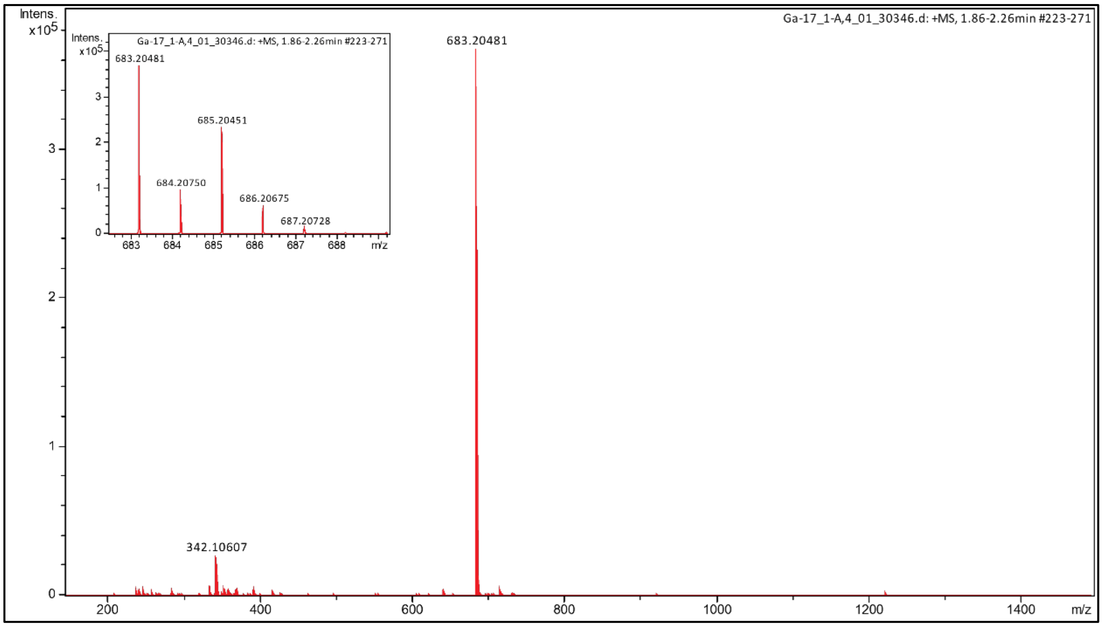

Figure S132. LC-MS data for compound **[^{nat}Ga(17)]$^{+}$**.

natZinc (S)-2,2'-(7-(2-((2-((2-((1-amino-3-(1H-indol-3-yl)-1-oxopropan-2-yl)amino)-2-oxoethyl)amino)-2-oxoethyl)(methyl)amino)-2-oxoethyl)-1,4,7-triazonane-1,4-diyl)diacetate, **[natZn(17)]**. Complex **[natZn(17)]** was synthesized using the general complexation protocol outlined in **Section 3.1** from compound **17** and $ZnSO_4 \cdot 7H_2O$. The complex was purified using a Sep-Pak Plus C18 short cartridge and characterized by HPLC chromatography and mass spectrometry. R_t (Method A): 6.39 min. ESI-MS $[M]^+$ calc. for $C_{28}H_{38}N_8O_8Zn$ 679.2177, found 679.2178.

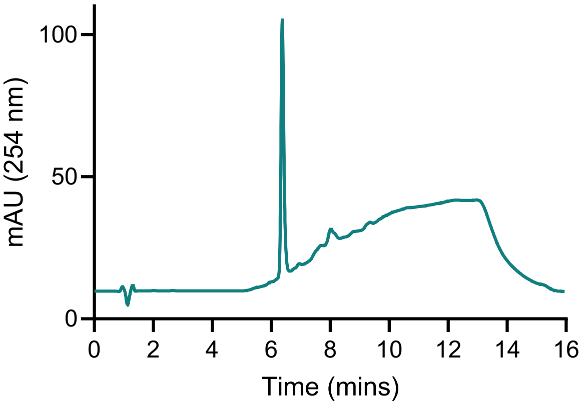

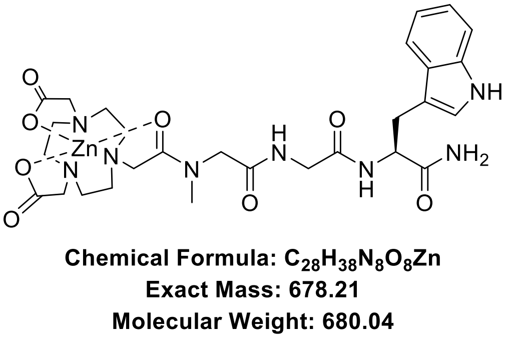

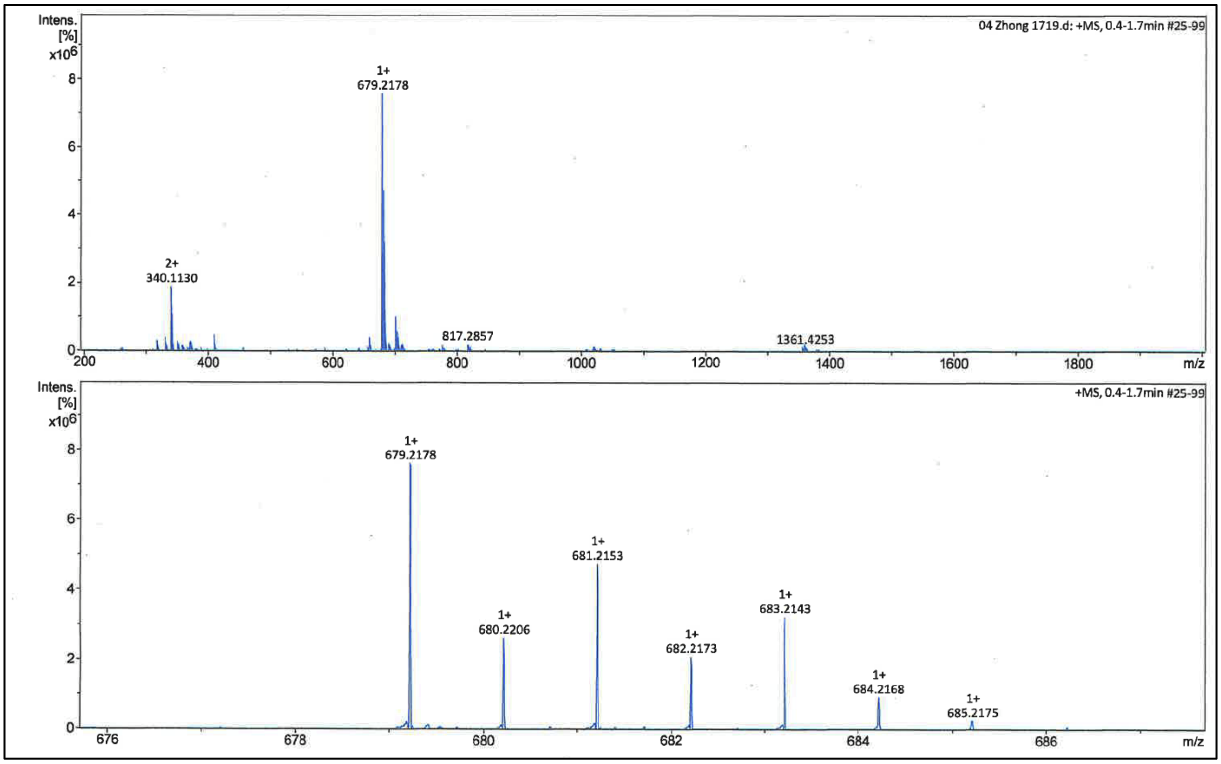

Figure S133. LC-MS data for compound **[natZn(17)]**.

natIron (S)-2,2'-(7-(2-((2-((2-((1-amino-3-(1H-indol-3-yl)-1-oxopropan-2-yl)amino)-2-oxoethyl)amino)-2-oxoethyl)(methyl)amino)-2-oxoethyl)-1,4,7-triazonane-1,4-diyl)diacetate, **[^{nat}Fe(17)]$^+$**. Complex **[^{nat}Fe(17)]$^+$** was synthesized using the general complexation protocol outlined in **Section 3.1** from compound **17** and **FeCl$_3$**. The complex was purified using a Sep-Pak Plus C18 short cartridge and characterized by HPLC chromatography and mass spectrometry. R_t (Method A): 6.00 min. ESI-MS $[M]^+$ calc. for $C_{28}H_{38}FeN_8O_8$ 670.2157, found 670.2149.

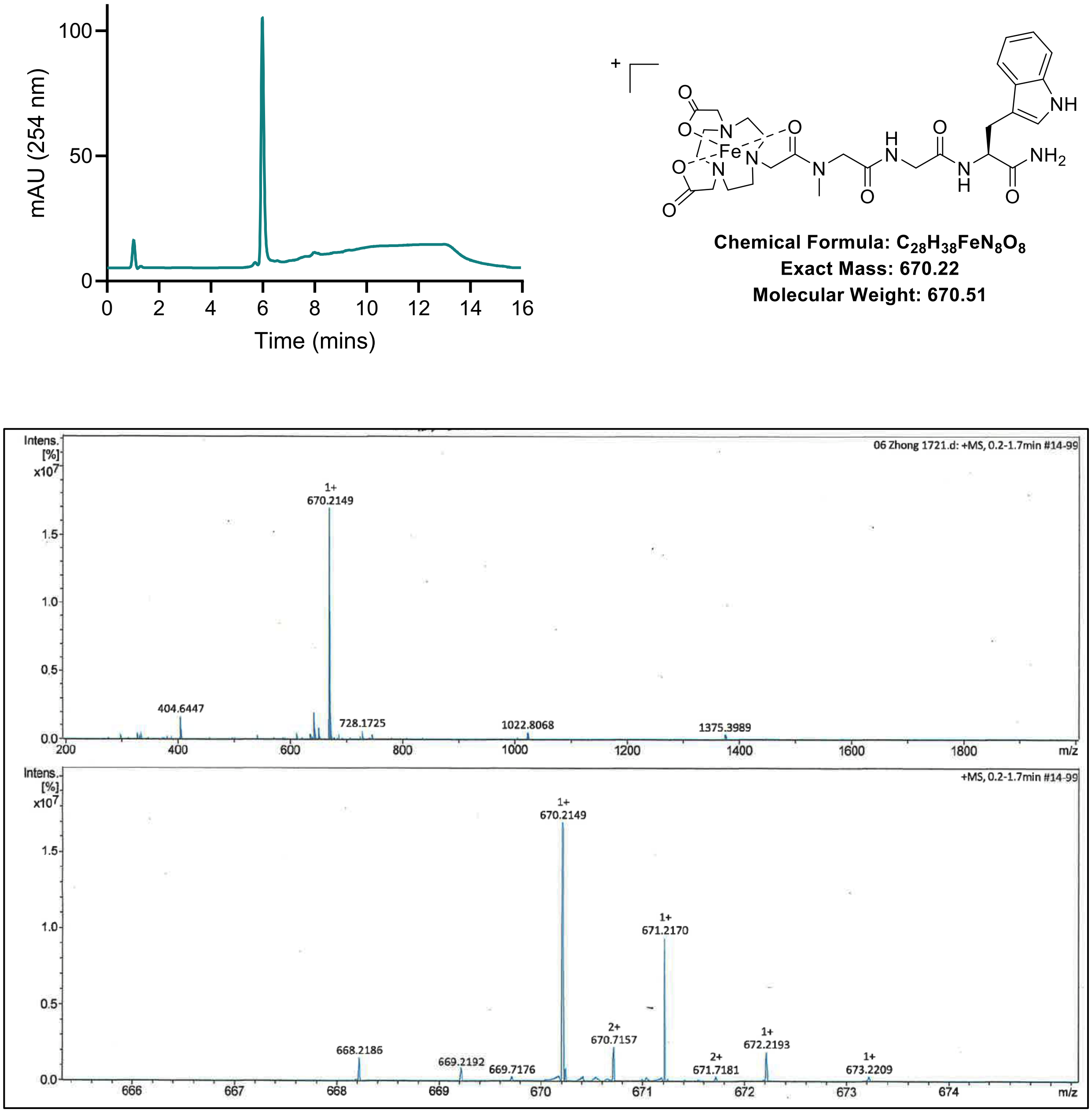

Figure S134. LC-MS data for compound **[^{nat}Fe(17)]$^+$**.

natIndium (S)-2,2'-(7-(2-((2-((2-((1-amino-3-(1H-indol-3-yl)-1-oxopropan-2-yl)amino)-2-oxoethyl)amino)-2-oxoethyl)(methyl)amino)-2-oxoethyl)-1,4,7-triazonane-1,4-diyl)diacetate, **[^{nat}In(17)]$^+$**. Complex **[^{nat}In(17)]$^+$** was synthesized using the general complexation protocol outlined in **Section 3.1** from compound **17** and **$InCl_3$**. The complex was purified using a Sep-Pak Plus C18 short cartridge and characterized by HPLC chromatography and mass spectrometry. R_t (Method A): 6.19 min. ESI-MS $[M]^+$ calc. for $C_{28}H_{38}InN_8O_8$ 729.1846, found 729.1850.

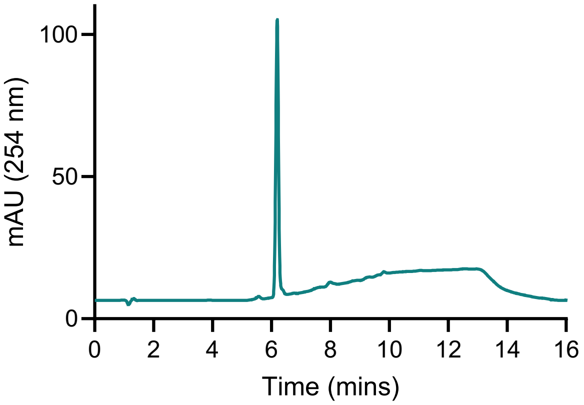

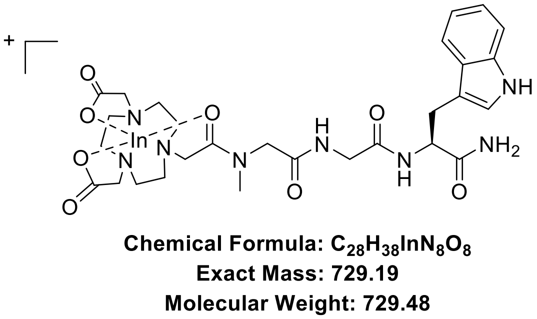

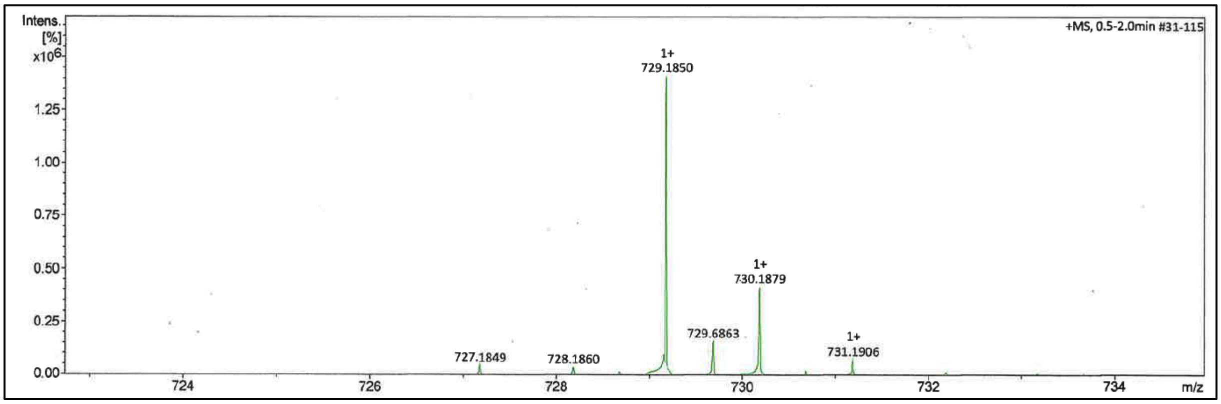

Figure S135. LC-MS data for compound **[^{nat}In(17)]$^+$**.

natGallium 2,2'-(7-(2-(((R)-1-((2-(((S)-1-amino-3-(1H-indol-3-yl)-1-oxopropan-2-yl)amino)-2-oxoethyl)amino)-4-(methylthio)-1-oxobutan-2-yl)(methyl)amino)-2-oxoethyl)-1,4,7-triazonane-1,4-diyl)diacetate, **[natGa(18)]**$^+$. Complex **[natGa(18)]**$^+$ was synthesized using the general complexation protocol outlined in **Section 3.1** from compound **18** and $Ga(NO_3)_3 \cdot 3H_2O$. The complex was purified using a Sep-Pak Plus C18 short cartridge and characterized by HPLC chromatography and mass spectrometry. Rt (Method B): 7.18 min. ESI-MS $[M]^+$ calc. for $C_{31}H_{44}GaN_8O_8S$ 757.22530, found 757.22683.

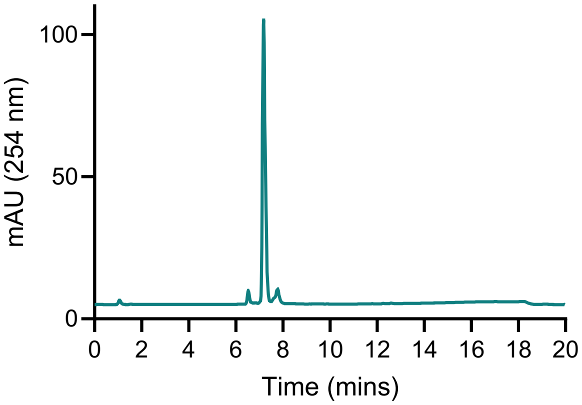

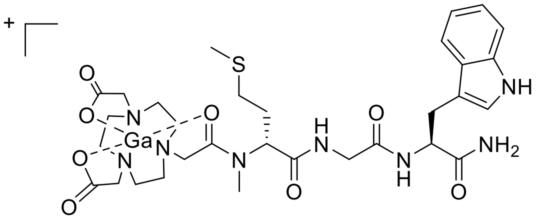

Chemical Formula: $C_{31}H_{44}GaN_8O_8S$
Exact Mass: 757.23
Molecular Weight: 758.52

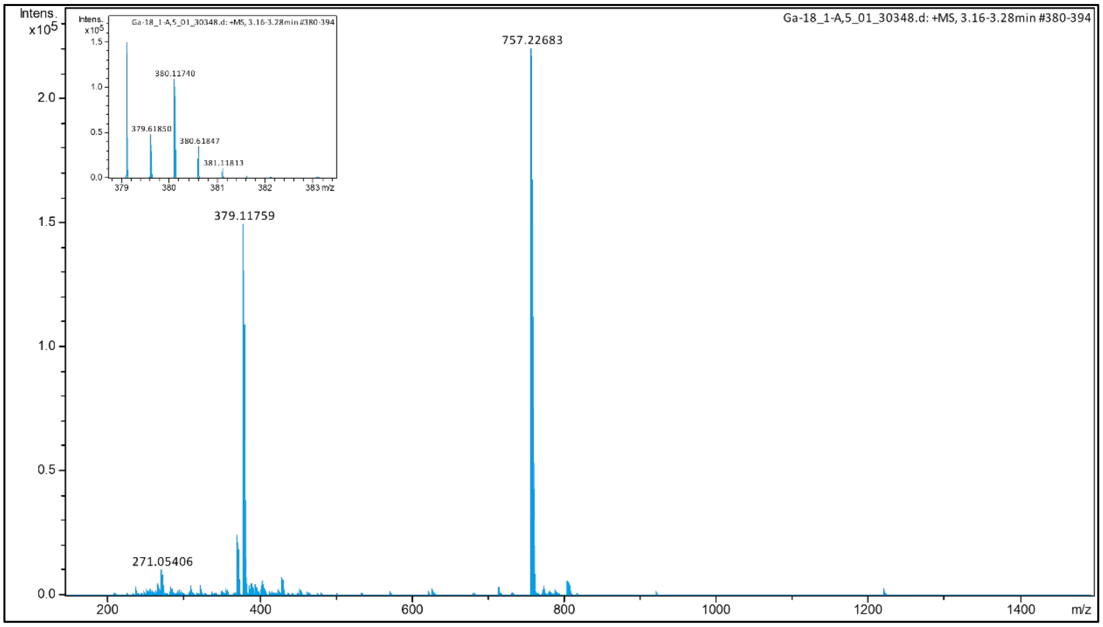

Figure S136. LC-MS data for compound **[natGa(18)]**$^+$.

[nat]Gallium (S)-2,2'-(7-(2-((3-((2-((1-amino-3-(1H-indol-3-yl)-1-oxopropan-2-yl)amino)-2-oxoethyl)amino)-3-oxopropyl)(methyl)amino)-2-oxoethyl)-1,4,7-triazonane-1,4-diyl)diaceate, **[^{nat}Ga(19)]$^+$**. Complex **[^{nat}Ga(19)]$^+$** was synthesized using the general complexation protocol outlined in **Section 3.1** from compound **19** and $Ga(NO_3)_3 \cdot 3H_2O$. The complex was purified using a Sep-Pak Plus C18 short cartridge and characterized by HPLC chromatography and mass spectrometry. R_t (Method B): 6.22 min. ESI-MS $[M]^+$ calc. for $C_{29}H_{40}GaN_8O_8$ 697.22193, found 697.22477.

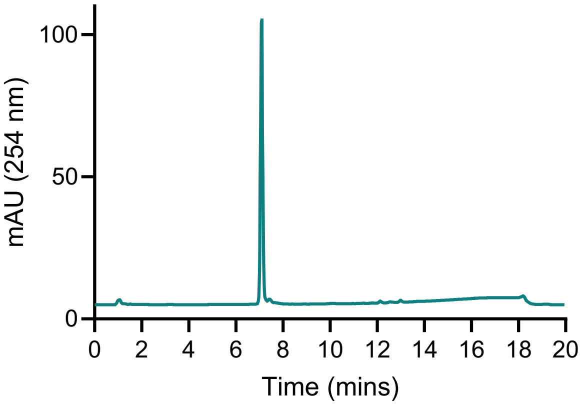

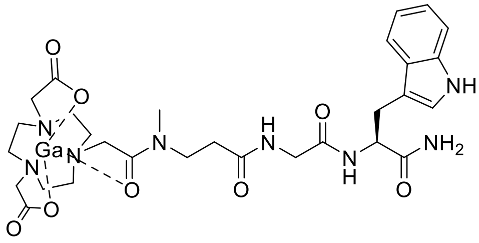

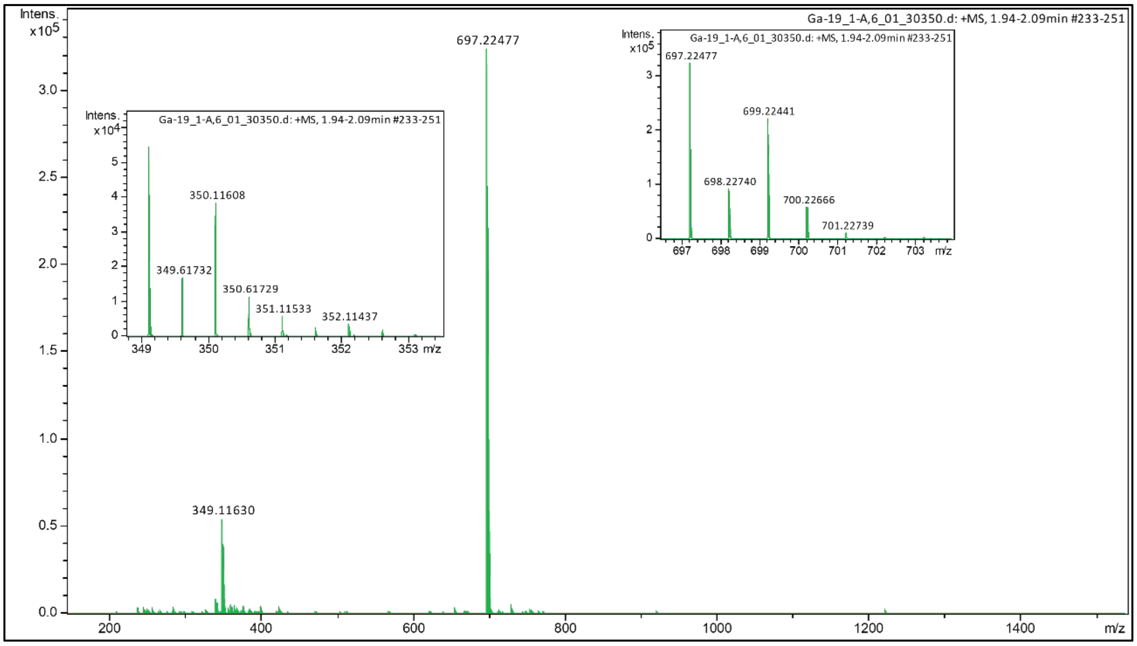

Figure S137. LC-MS data for compound **[^{nat}Ga(19)]$^+$**.

[nat]Gallium (7R,21S,25S)-1-(4,7-bis(carboxymethyl)-1,4,7-triazonan-1-yl)-7-(4-hydroxy-3-iodobenzyl)-2,5,8,15,23-pentaoxo-3,6,9,16,22,24-hexaazaheptacosane-21,25,27-tricarboxylic acid, **[natGa(20)]$^+$**. Complex **[natGa(20)]$^+$** was synthesized using the general complexation protocol outlined in **Section 3.1** from compound **20** and $Ga(NO_3)_3 \cdot 3H_2O$. The complex was purified using a Sep-Pak Plus C18 short cartridge and characterized by HPLC chromatography and mass spectrometry. R_t (Method B): 7.08 min. ESI-MS $[M]^+$ calc. for $C_{41}H_{60}GaIN_9O_{16}$, found 743.1.

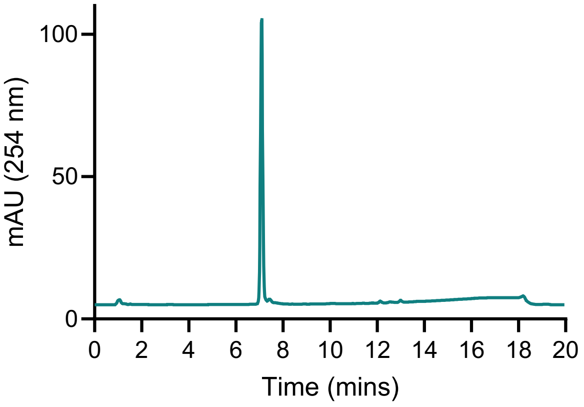

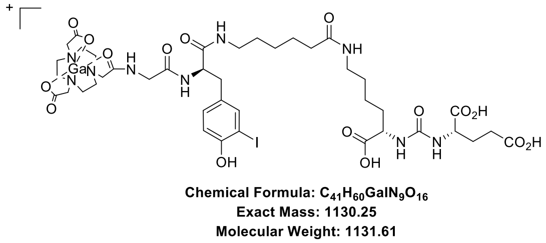

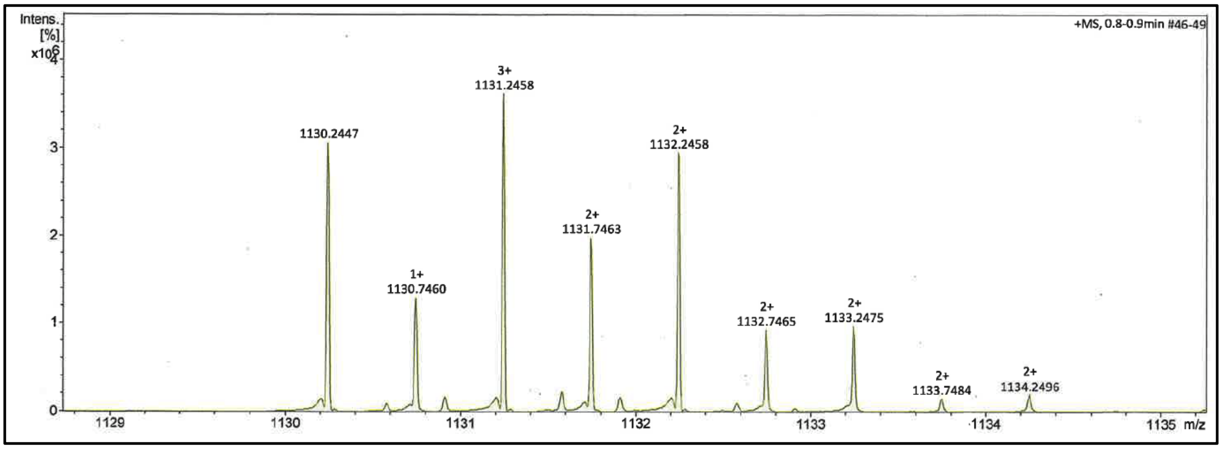

Figure S138. LC-MS data for compound **[natGa(20)]$^+$**.

[nat]Gallium (4S,7R,21S,25S)-1-(4,7-bis(carboxymethyl)-1,4,7-triazonan-1-yl)-7-(4-hydroxy-3-iodobenzyl)-4-(hydroxymethyl)-2,5,8,15,23-pentaoxo-3,6,9,16,22,24-hexaazaheptacosane-21,25,27-tricarboxylic acid, **[^{nat}Ga(21)]$^+$**. Complex **[^{nat}Ga(21)]$^+$** was synthesized using the general complexation protocol outlined in **Section 3.1** from compound **21** and $Ga(NO_3)_3 \cdot 3H_2O$. The complex was purified using a Sep-Pak Plus C18 short cartridge and characterized by HPLC chromatography and mass spectrometry. R_t (Method B): 6.88 min. ESI-MS $[M]^+$ calc. for $C_{42}H_{62}GaIN_9O_{17}$ 1160.2559, found 1160.2546.

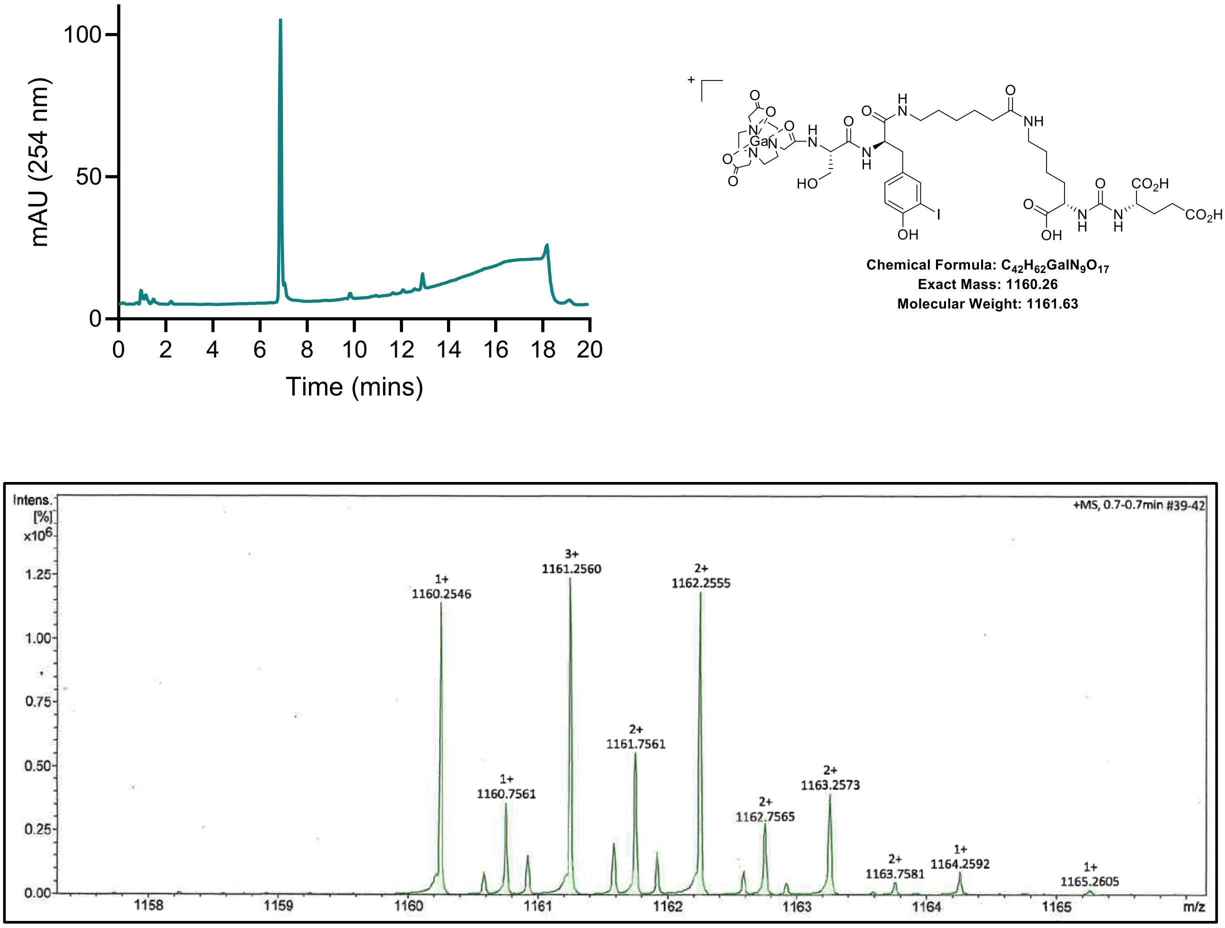

Figure S139. LC-MS data for compound **[^{nat}Ga(21)]$^+$**.

natGallium (5S,8R,22S,26S)-5-(2-(4,7-bis(carboxymethyl)-1,4,7-triazonan-1-yl)acetamido)-8-(4-hydroxy-3-iodobenzyl)-6,9,16,24-tetraoxo-2-thia-7,10,17,23,25-pentaazaoctacosane-22,26,28-tricarboxylic acid, **[^{nat}Ga(22)]$^+$**. Complex **[^{nat}Ga(22)]$^+$** was synthesized using the general complexation protocol outlined in **Section 3.1** from compound **22** and $Ga(NO_3)_3 \cdot 3H_2O$. The complex was purified using a Sep-Pak Plus C18 short cartridge and characterized by HPLC chromatography and mass spectrometry. R_t (Method B): 7.54 min. ESI-MS $[M]^+$ calc. for $C_{44}H_{66}GaIN_9O_{16}S$ 1204.2643, found 1204.2640.

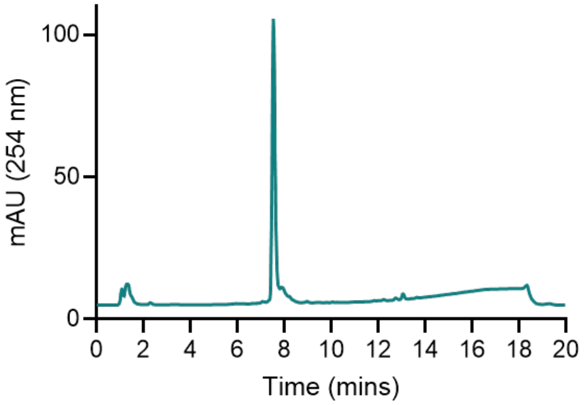

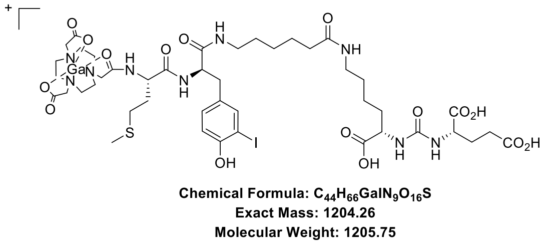

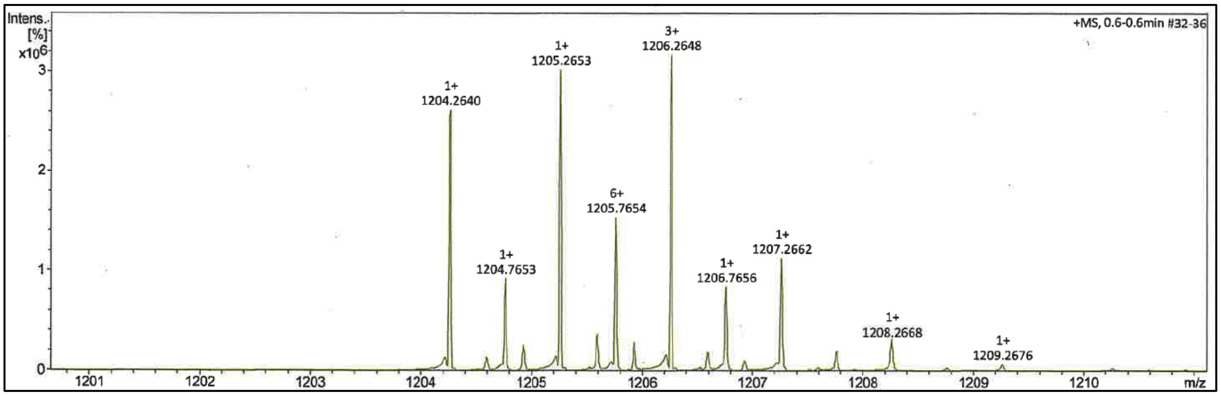

Figure S140. LC-MS data for compound **[^{nat}Ga(22)]$^+$**.

^{nat}Gallium (7R,21S,25S)-1-(4,7-bis(carboxymethyl)-1,4,7-triazonan-1-yl)-7-(4-hydroxy-3-iodobenzyl)-3-methyl-2,5,8,15,23-pentaoxo-3,6,9,16,22,24-hexaazaheptacosane-21,25,27-tricarboxylic acid, **[^{nat}Ga(23)]$^+$**. Complex **[^{nat}Ga(23)]$^+$** was synthesized using the general complexation protocol outlined in **Section 3.1** from compound **23** and $Ga(NO_3)_3 \cdot 3H_2O$. The complex was purified using a Sep-Pak Plus C18 short cartridge and characterized by HPLC chromatography and mass spectrometry. R_t (Method B): 7.06 min. ESI-MS $[M]^+$ calc. for $C_{42}H_{62}GaIN_9O_{16}$ 1144.2610, found 1144.2600.

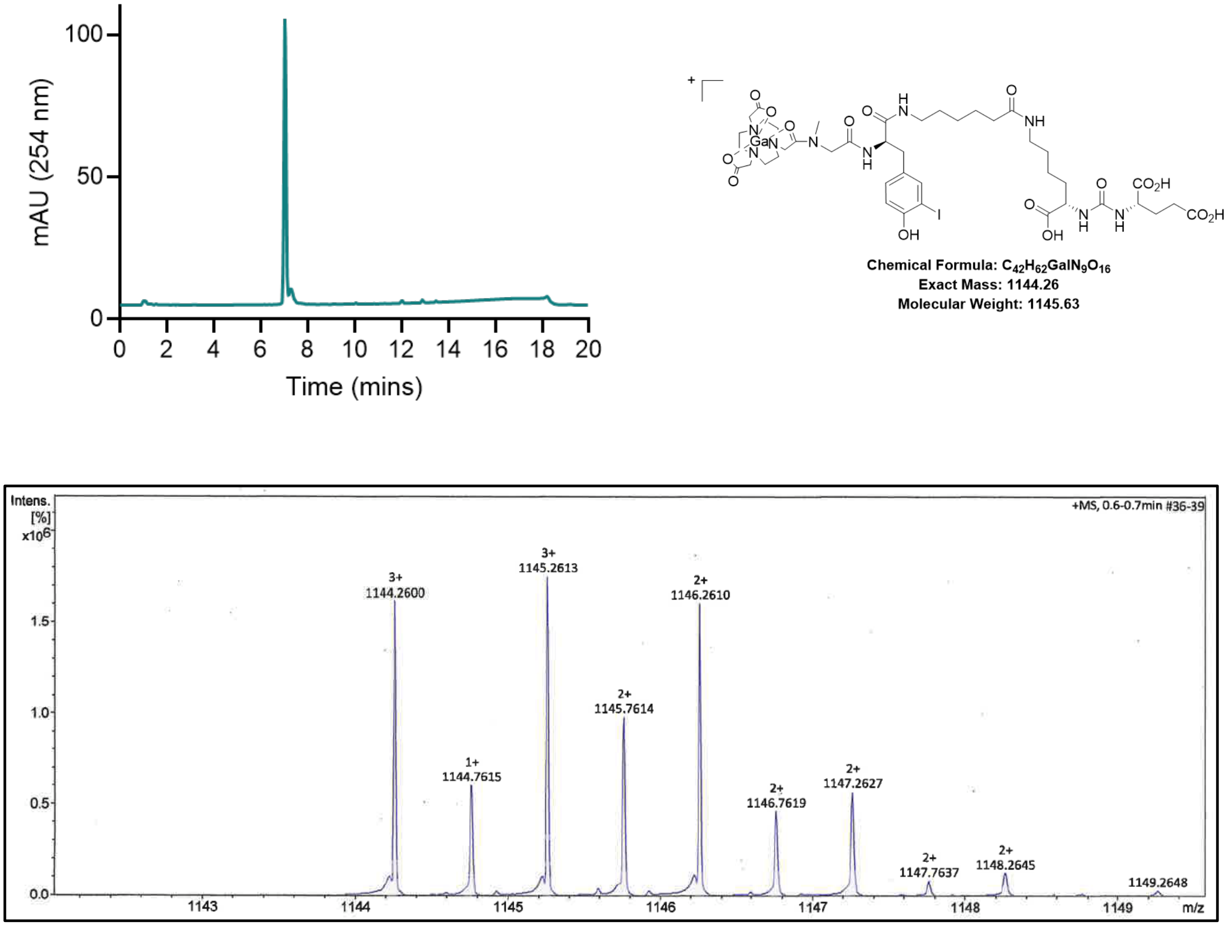

Figure S141. LC-MS data for compound **[^{nat}Ga(23)]$^+$**.

natGallium (4S,7R,21S,25S)-1-(4,7-bis(carboxymethyl)-1,4,7-triazonan-1-yl)-7-(4-hydroxy-3-iodobenzyl)-4-(hydroxymethyl)-3-methyl-2,5,8,15,23-pentaoxo-3,6,9,16,22,24-hexaazaheptacosane-21,25,27-tricarboxylic acid, **[natGa(24)]**$^+$. Complex **[natGa(24)]**$^+$ was synthesized using the general complexation protocol outlined in **Section 3.1** from compound **24** and $Ga(NO_3)_3 \cdot 3H_2O$. The complex was purified using a Sep-Pak Plus C18 short cartridge and characterized by HPLC chromatography and mass spectrometry. Rt (Method B): 7.01 min. ESI-MS $[M]^+$ calc. for $C_{43}H_{64}GaIN_9O_{17}$ 1174.2715, found 1174.2710.

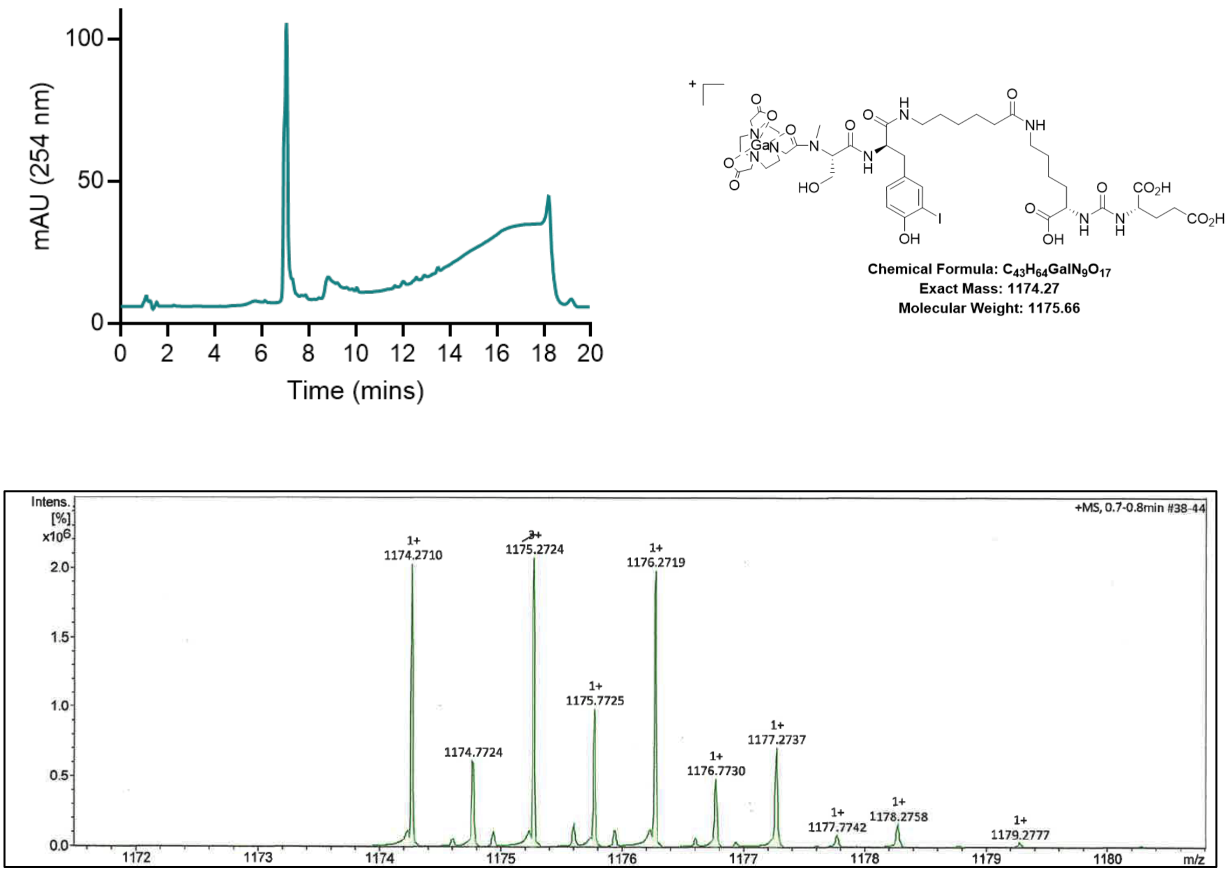

Figure S142. LC-MS data for compound **[natGa(24)]**$^+$.

natGallium (5S,8R,22S,26S)-5-(2-(4,7-bis(carboxymethyl)-1,4,7-triazonan-1-yl)-N-methylacetamido)-8-(4-hydroxy-3-iodobenzyl)-6,9,16,24-tetraoxo-2-thia-7,10,17,23,25-pentaazaoctacosane-22,26,28-tricarboxylic acid, **[natGa(25)]**$^+$. Complex **[natGa(25)]**$^+$ was synthesized using the general complexation protocol outlined in **Section 3.1** from compound **25** and $Ga(NO_3)_3 \cdot 3H_2O$. The complex was purified using a Sep-Pak Plus C18 short cartridge and characterized by HPLC chromatography and mass spectrometry. R_t (Method B): 7.58 min. ESI-MS $[M]^+$ calc. for $C_{45}H_{68}GaIN_9O_{16}S$ 1218.2800, found 1218.2791.

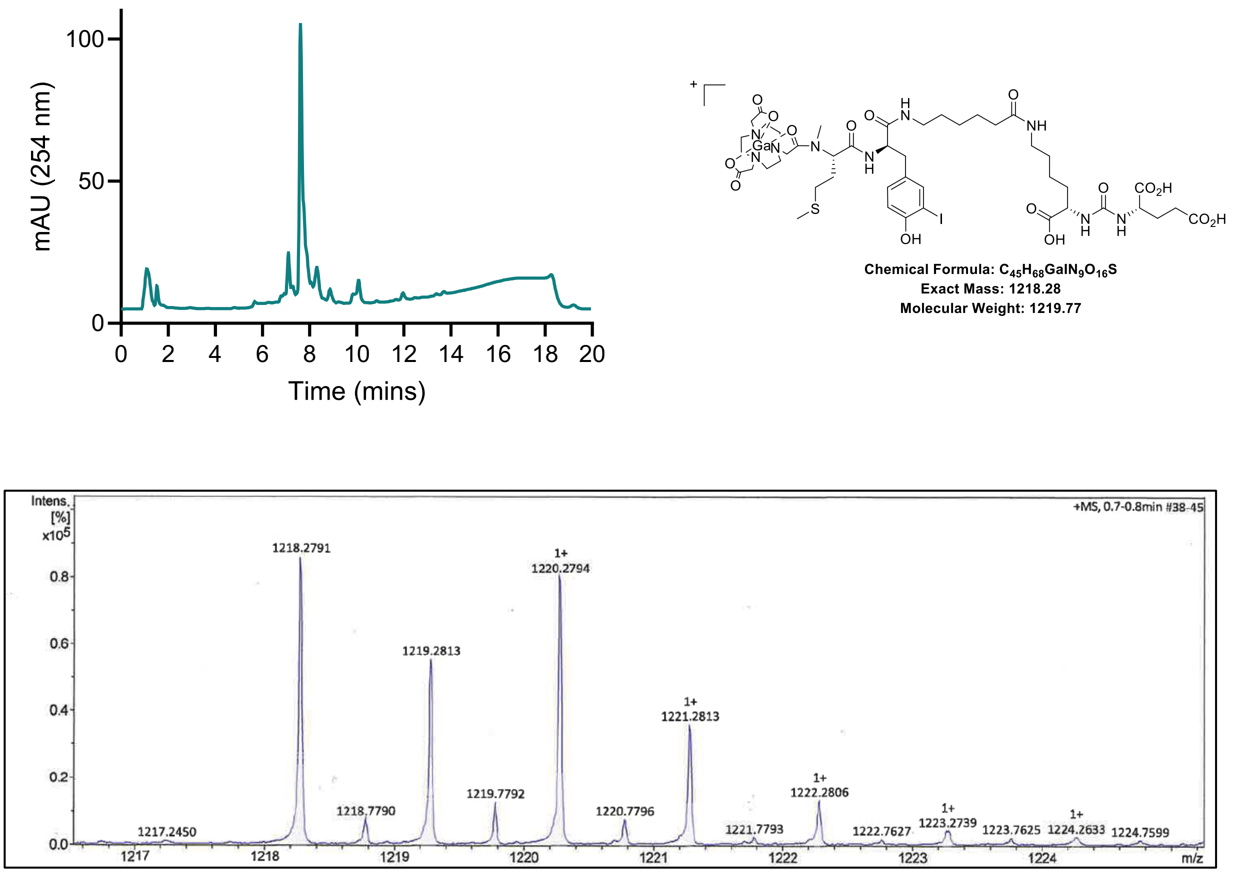

Figure S143. LC-MS data for compound **[natGa(25)]**$^+$.

3.4 HPLC-Monitored Complex Cleavage Experiments

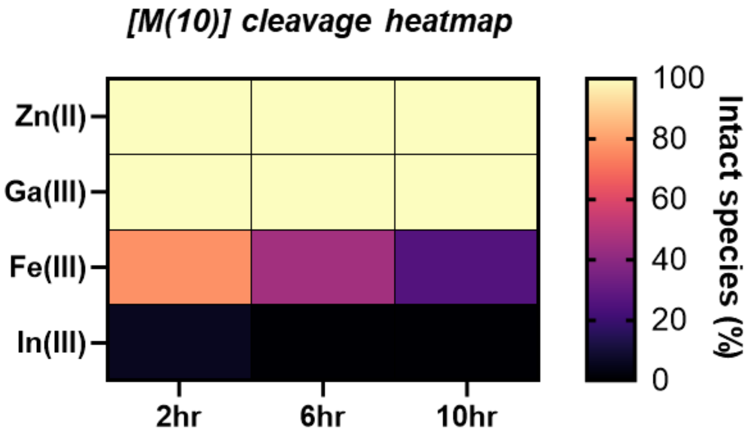

Figure S144. Comparative heatmap illustrating time-dependent various metal-driven autolytic amide bond cleavage kinetics of Gly conjugate (**10**).

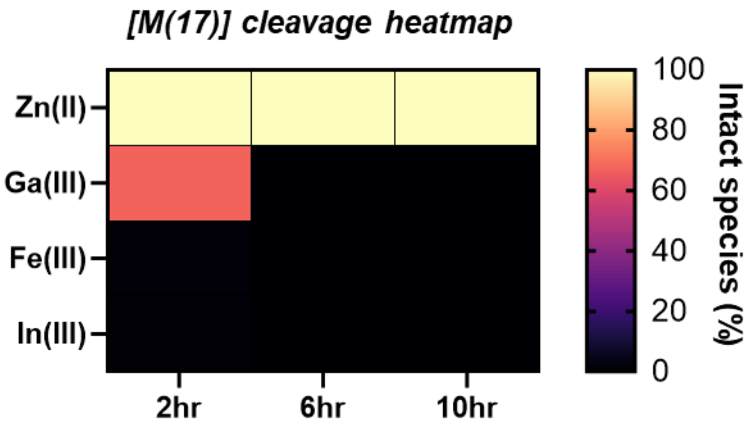

Figure S145. Comparative heatmap illustrating time-dependent various metal-driven autolytic amide bod cleavage kinetics of ^{NMe}Gly conjugate (**17**).

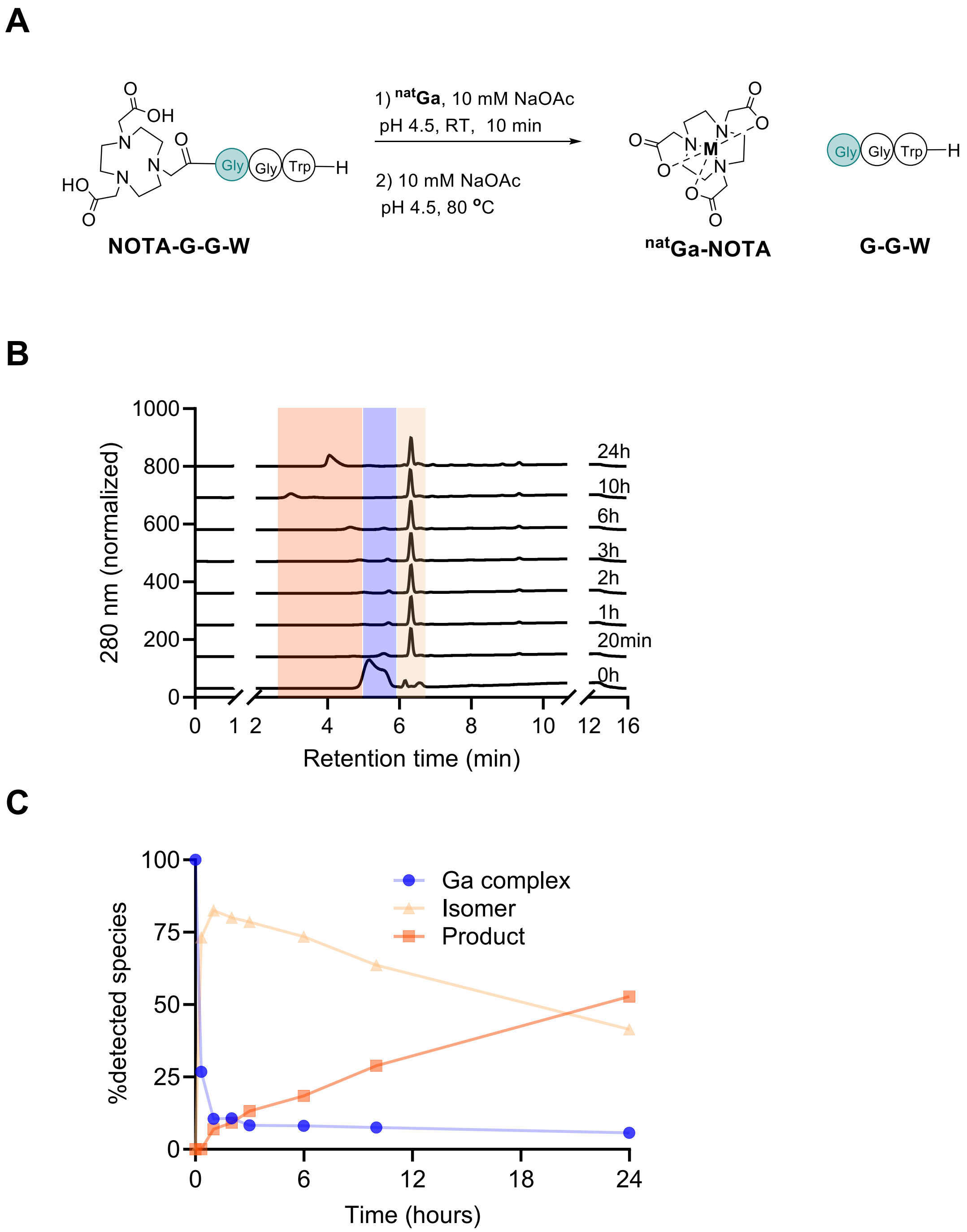

Figure S146. (A) Schematic description of preparation and direct complexation procedure of **[^{nat}Ga(10)]**$^{+}$. (B) Analytical HPLC chromatograms showing autolytic release of the ^{nat}Ga-NOTA. (C) Quantification of the pH-dependent cleavage at 80 °C and pH 4.5.

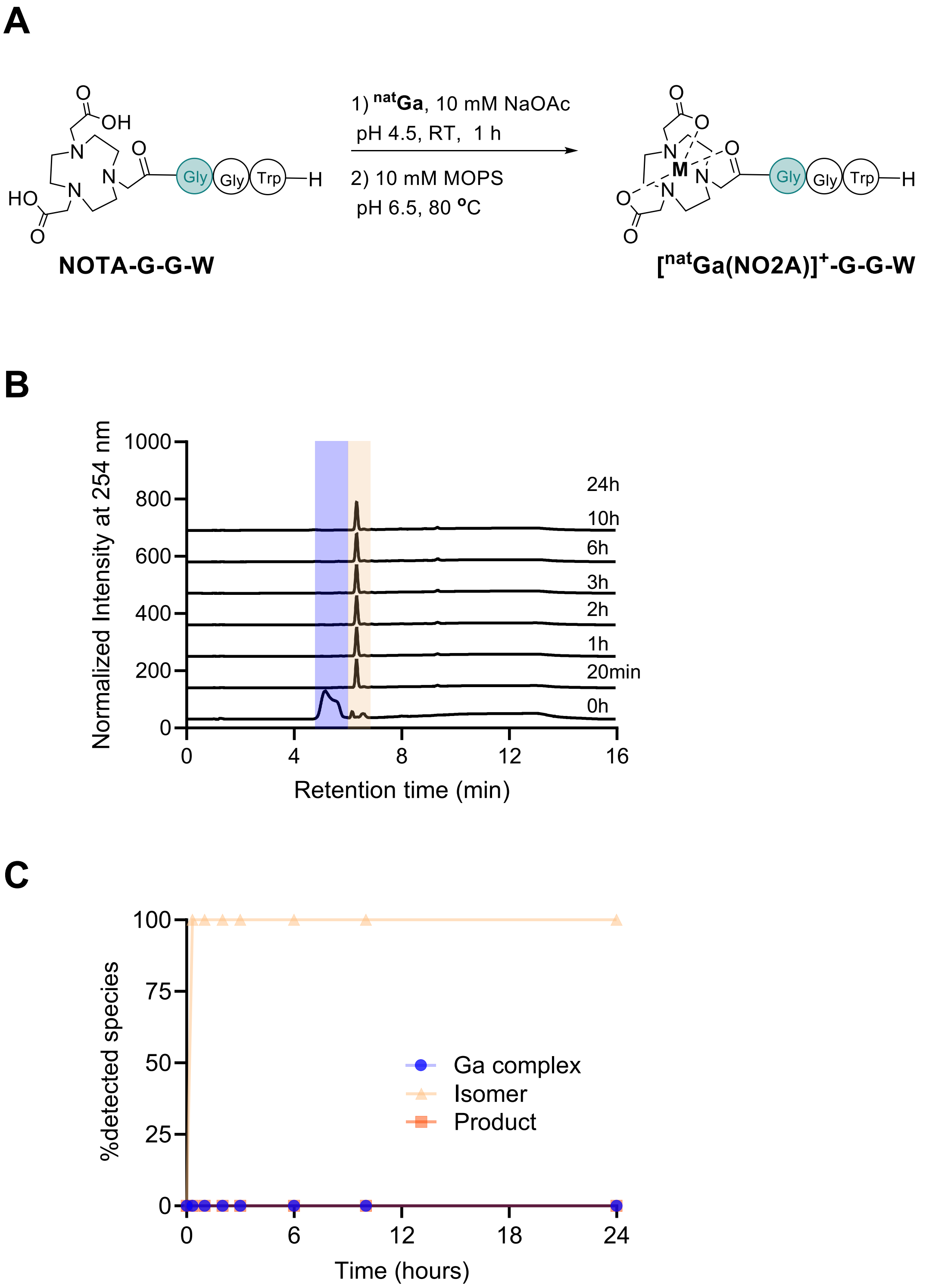

Figure S147. (A) Schematic description of preparation and direct complexation procedure of **[^{nat}Ga(10)]$^{+}$**. (B) Analytical HPLC chromatograms showing no autolytic release of the ^{nat}Ga-NOTA. (C) Quantification of the pH-dependent cleavage at 80 °C and pH 6.5.

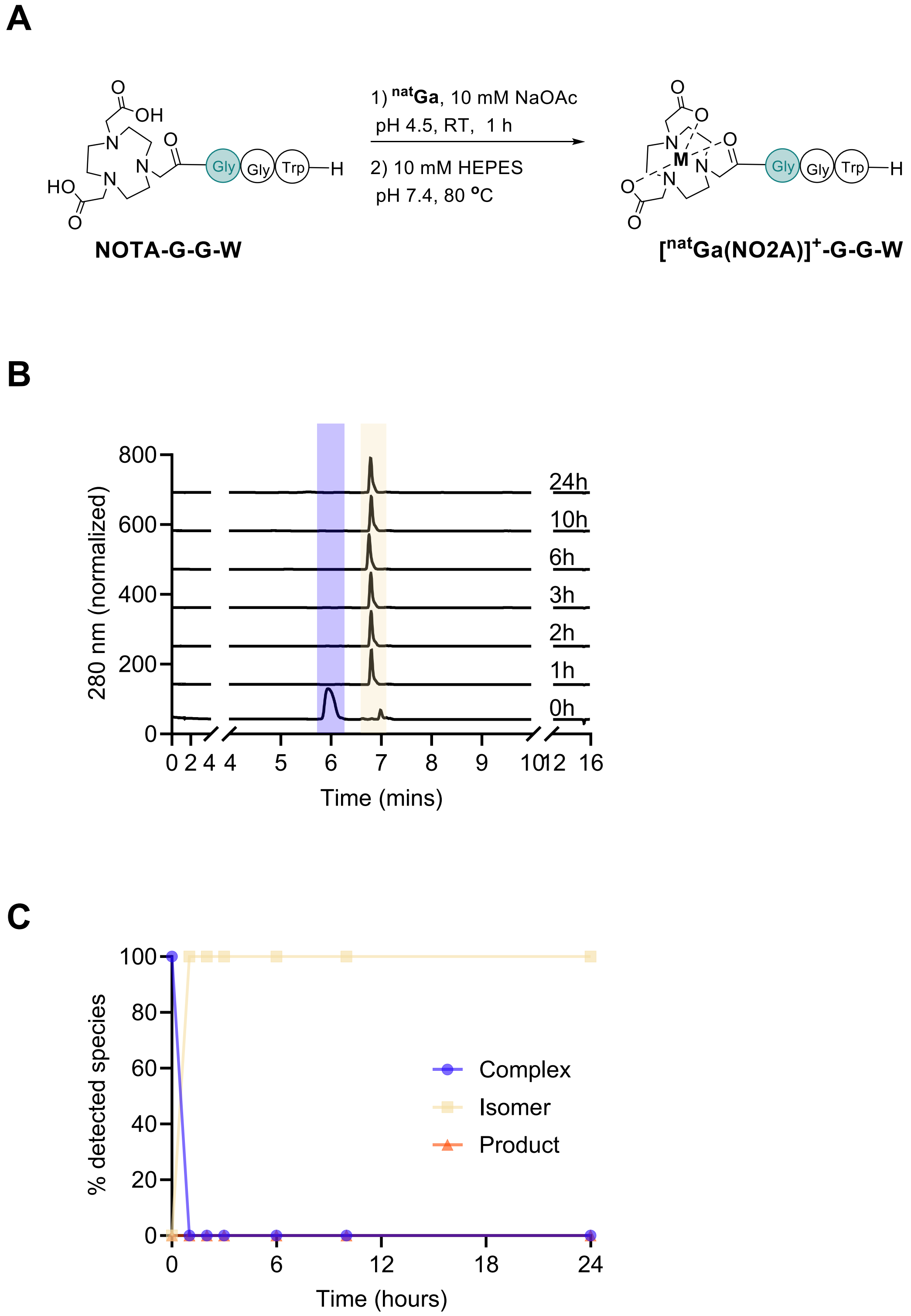

Figure S148. (A) Schematic description of preparation and direct complexation procedure of **[^{nat}Ga(10)]$^{+}$**. **(B)** Analytical HPLC chromatograms showing no autolytic release of the ^{nat}Ga-NOTA. **(C)** Quantification of the pH-dependent cleavage at 80 °C and pH 7.4.

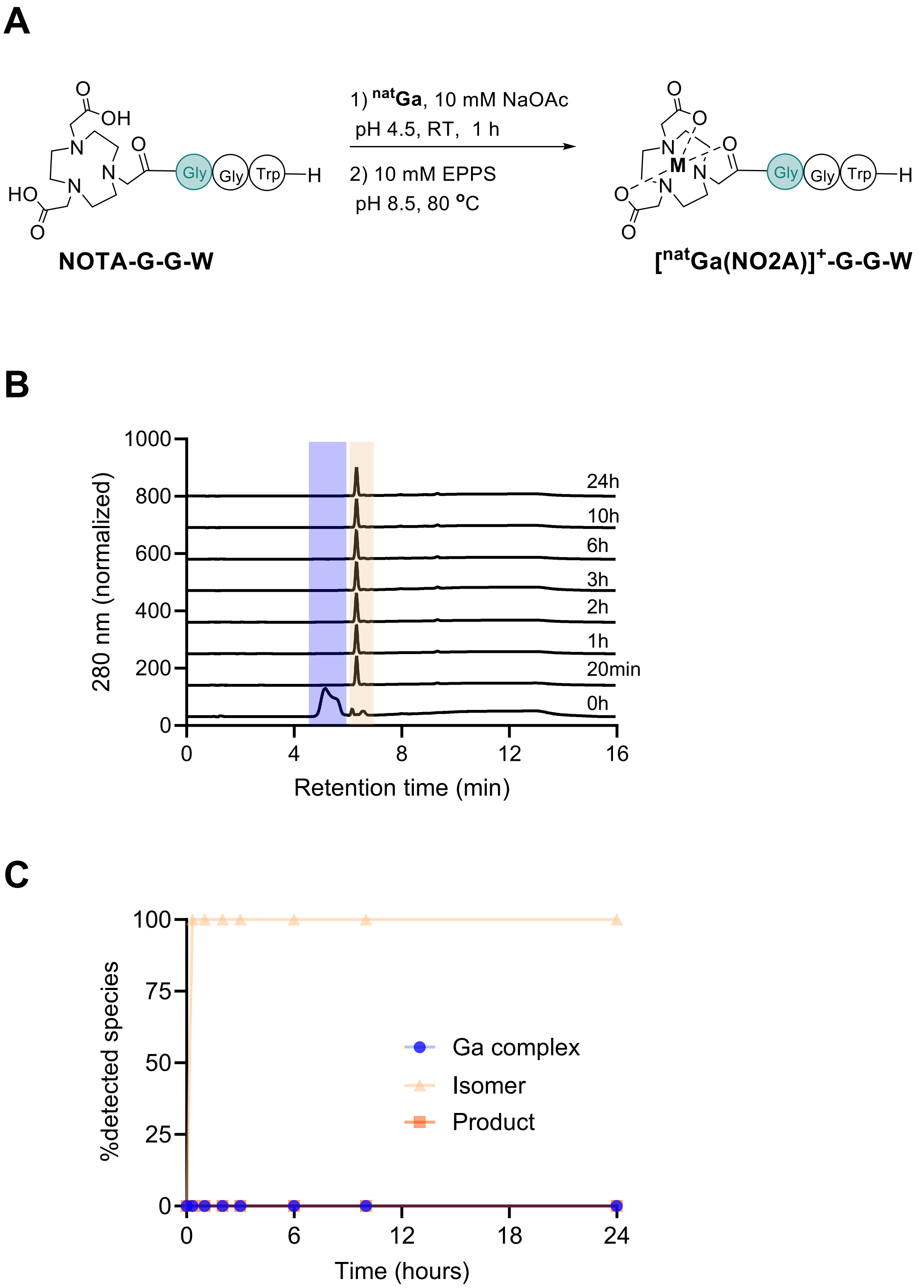

Figure S149. (A) Schematic description of preparation and direct complexation procedure of **[^{nat}Ga(10)]$^{+}$**. **(B)** Analytical HPLC chromatograms showing no autolytic release of the ^{nat}Ga-NOTA. **(C)** Quantification of the pH-dependent cleavage at 80 °C and pH 8.5.

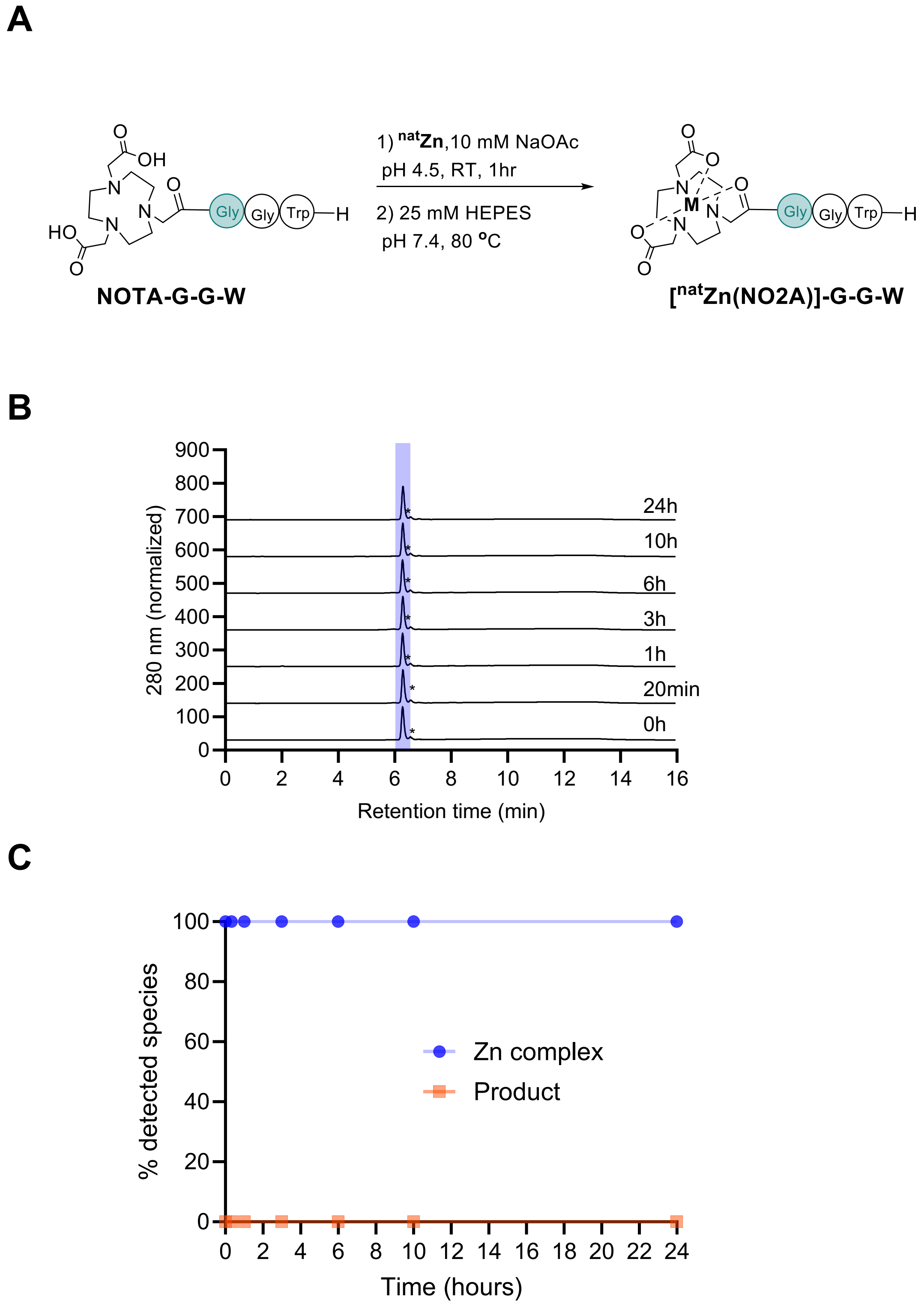

Figure S150. (A) Schematic description of preparation and direct complexation procedure of **[^{nat}Zn(10)]**. **(B)** Analytical HPLC chromatograms showing no autolytic release of the ^{nat}Zn-NOTA. **(C)** Quantification of the pH-dependent cleavage at 80 °C and pH 7.4.

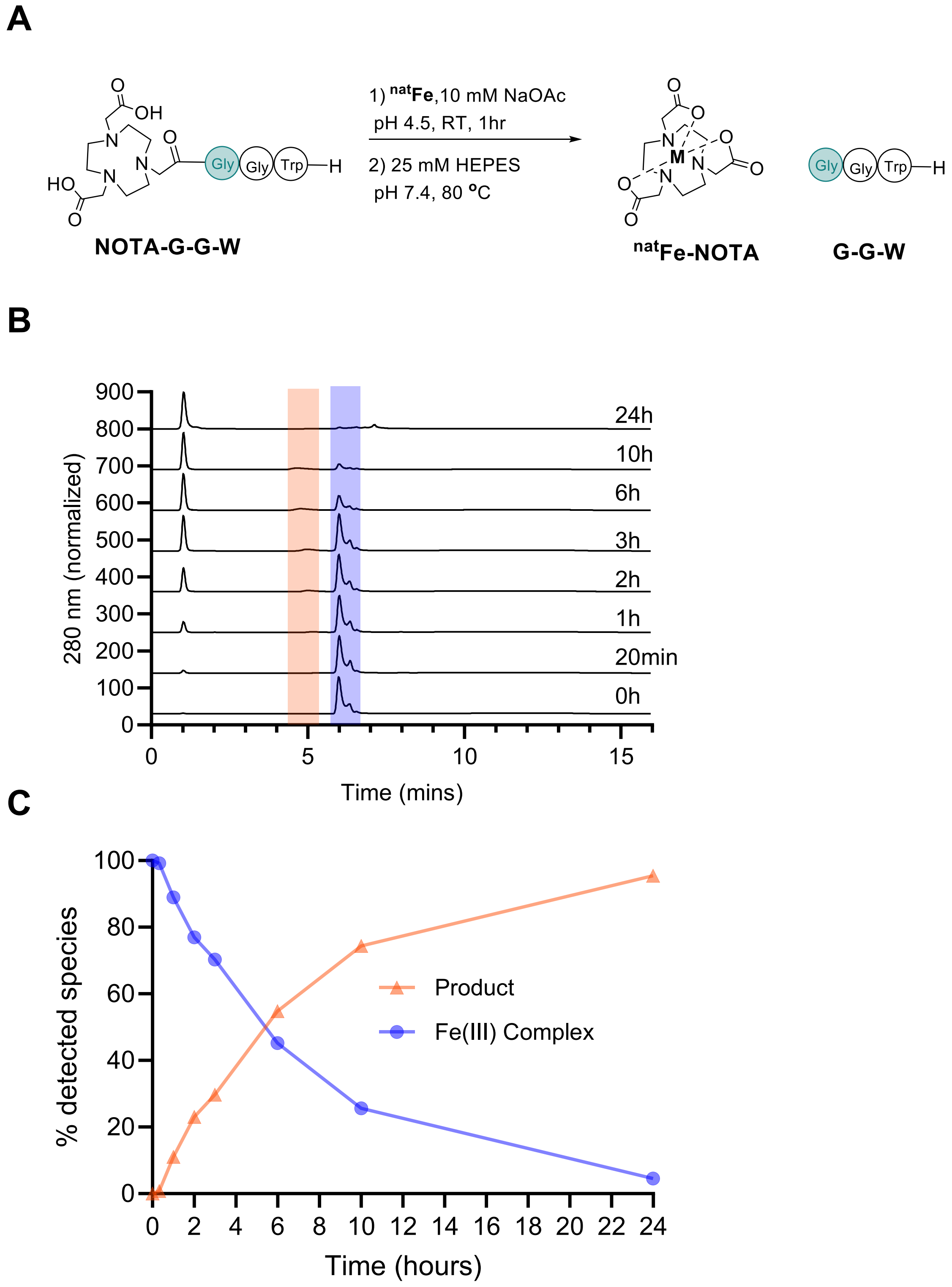

Figure S151. (A) Schematic description of preparation and direct complexation procedure of **[natFe(10)]+**. **(B)** Analytical HPLC chromatograms showing autolytic release of the natFe-NOTA. **(C)** Quantification of the pH-dependent cleavage at 80 °C and pH 7.4.

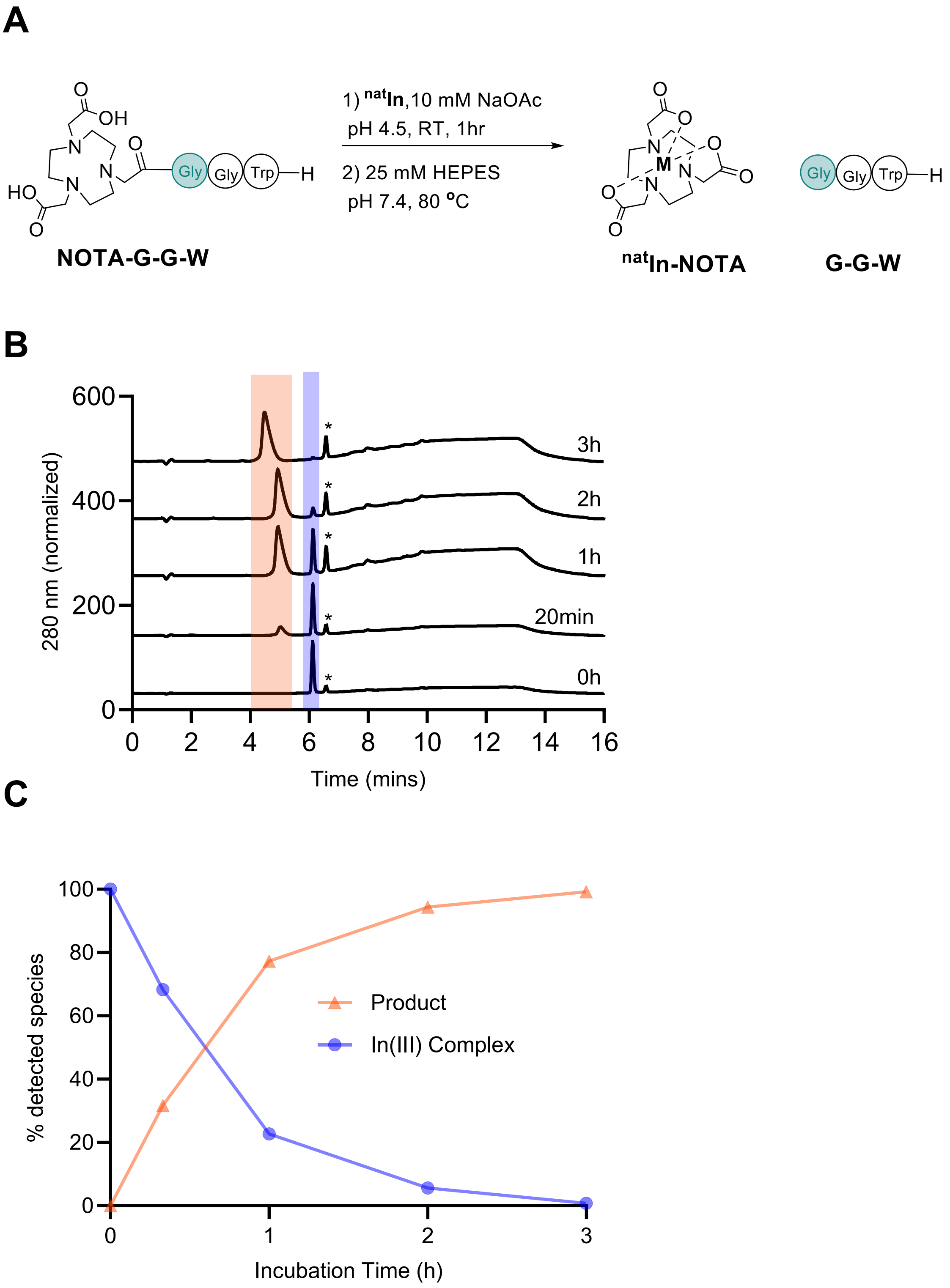

Figure S152. **(A)** Schematic description of preparation and direct complexation procedure of **[^{nat}In(10)]$^{+}$**. **(B)** Analytical HPLC chromatograms showing autolytic release of the ^{nat}In-NOTA. **(C)** Quantification of the pH-dependent cleavage at 80 °C and pH 7.4.

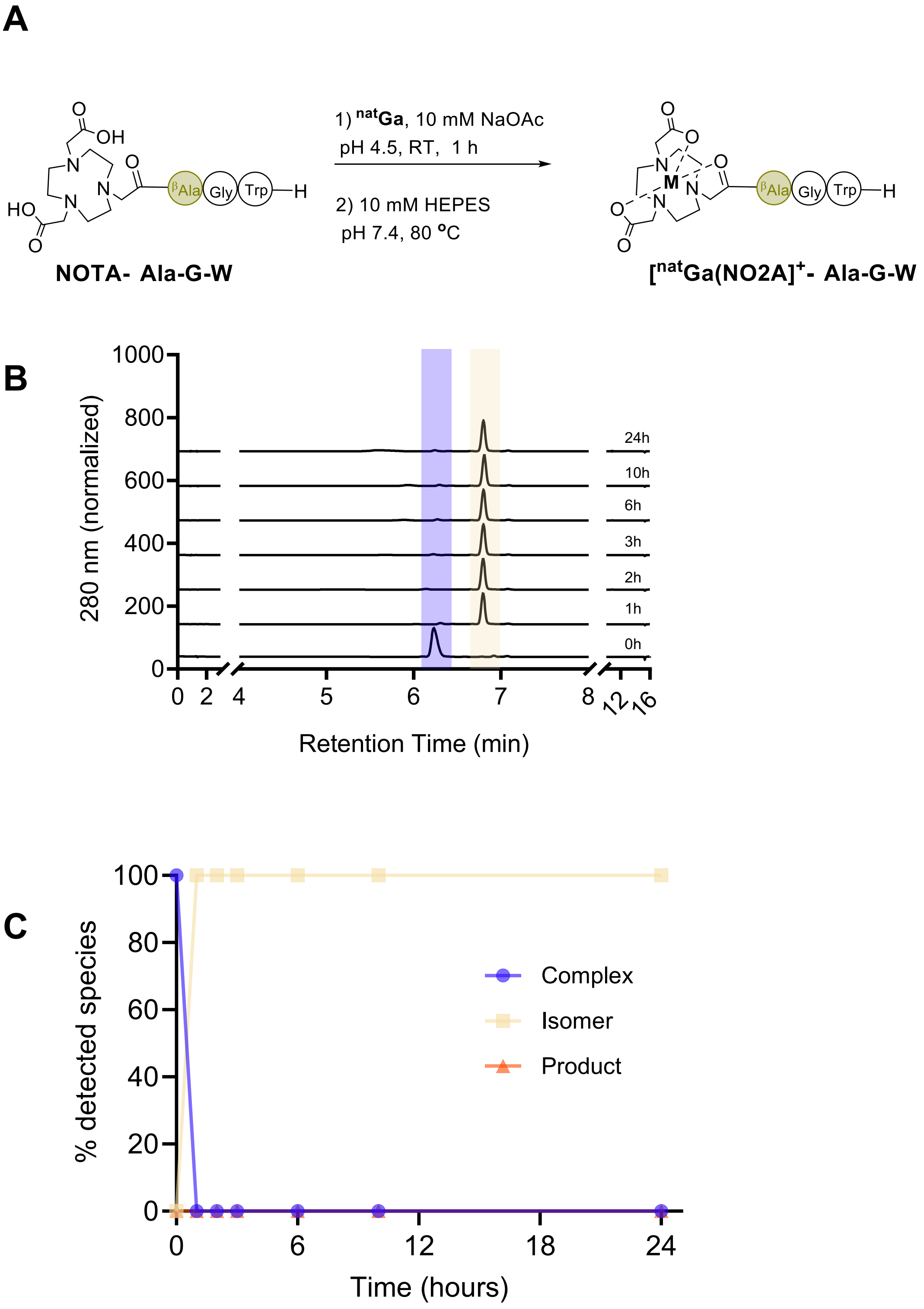

Figure S153. (A) Schematic description of preparation and direct complexation procedure of **[natGa(11)]+**. **(B)** Analytical HPLC chromatograms showing no autolytic release of the natGa-NOTA. **(C)** Quantification of the pH-dependent cleavage at 80 ºC and pH 7.4.

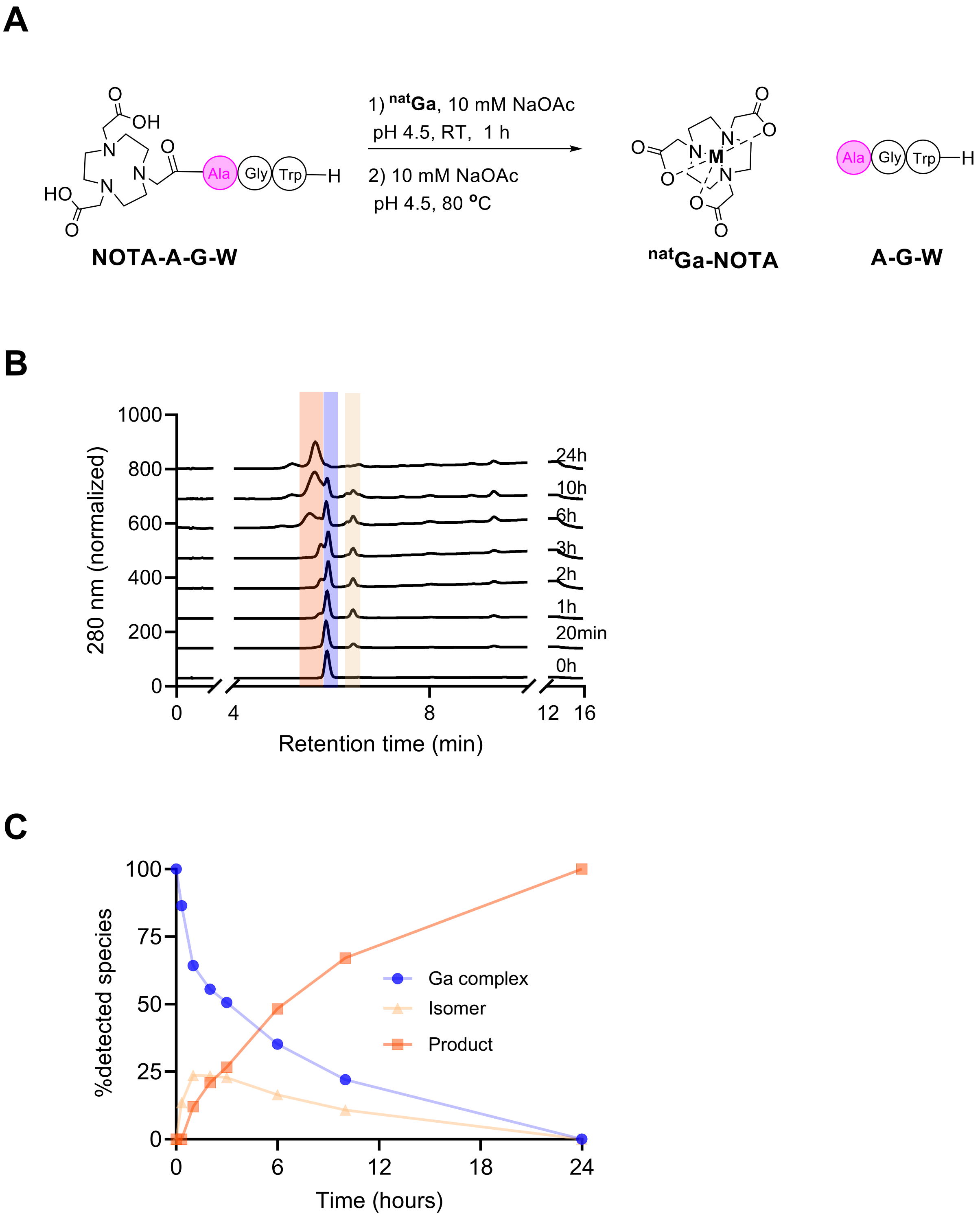

Figure S154. (A) Schematic description of preparation and direct complexation procedure of **[^{nat}Ga(12)]$^{+}$**. (B) Analytical HPLC chromatograms showing autolytic release of the ^{nat}Ga-NOTA. (C) Quantification of the pH-dependent cleavage at 80 °C and pH 4.5.

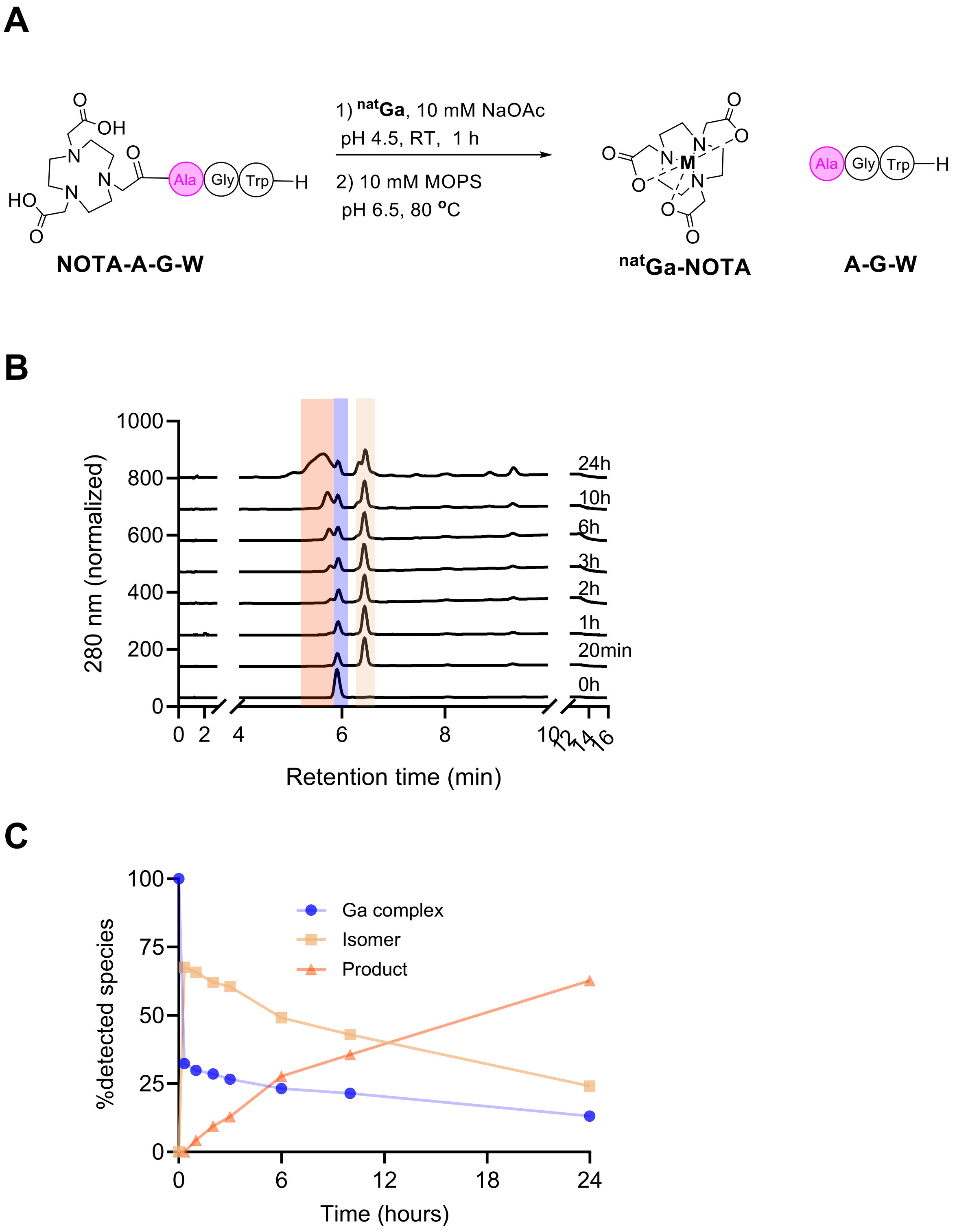

‘

Figure S155. (A) Schematic description of preparation and direct complexation procedure of **[natGa(12)]**$^{+}$. (B) Analytical HPLC chromatograms showing autolytic release of the natGa-NOTA. (C) Quantification of the pH-dependent cleavage at 80 °C and pH 6.5.

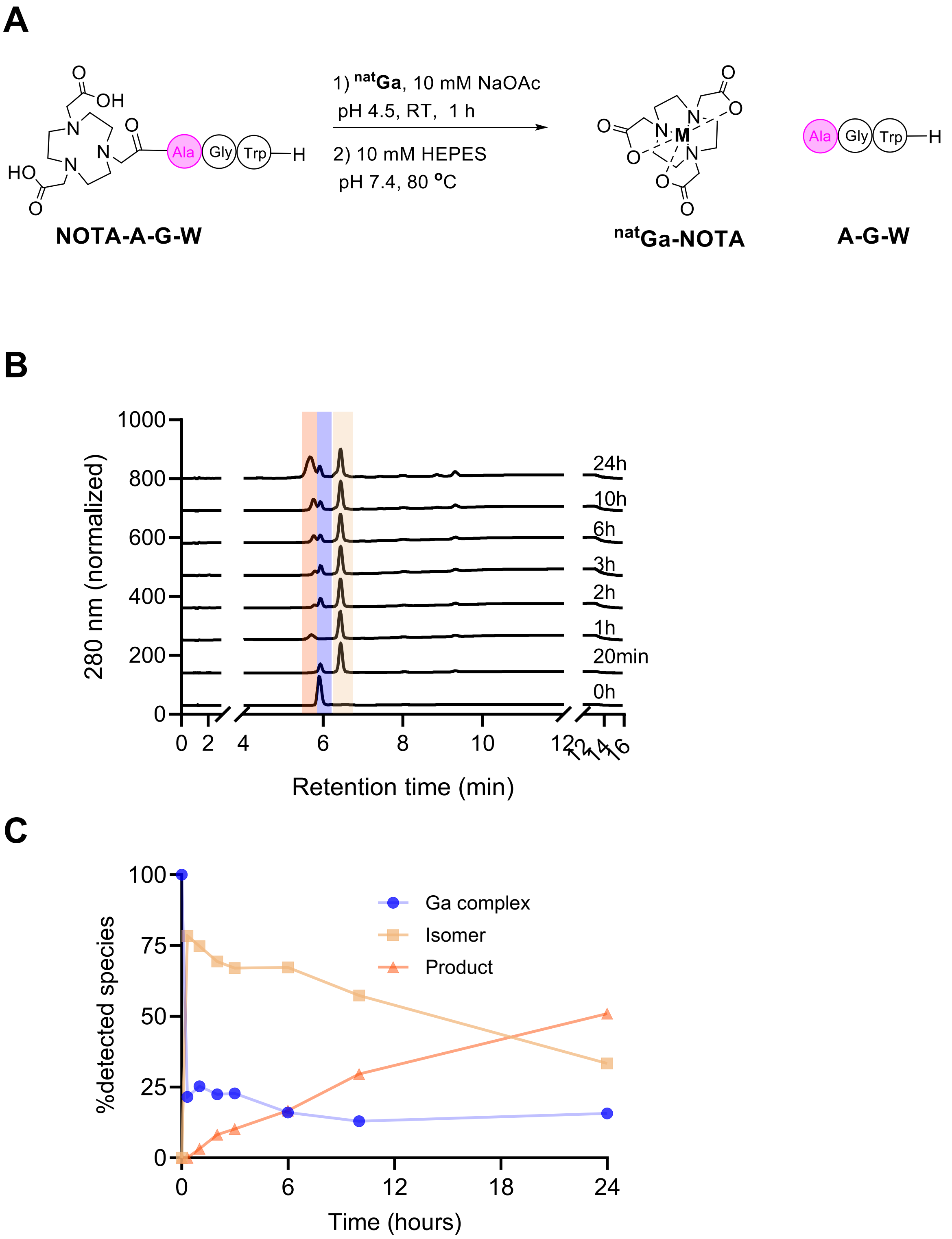

Figure S156. **(A)** Schematic description of preparation and direct complexation procedure of **[^{nat}Ga(12)]$^{+}$**. **(B)** Analytical HPLC chromatograms showing the autolytic release of the ^{nat}Ga-NOTA. **(C)** Quantification of the pH-dependent cleavage at 80 °C and pH 7.4.

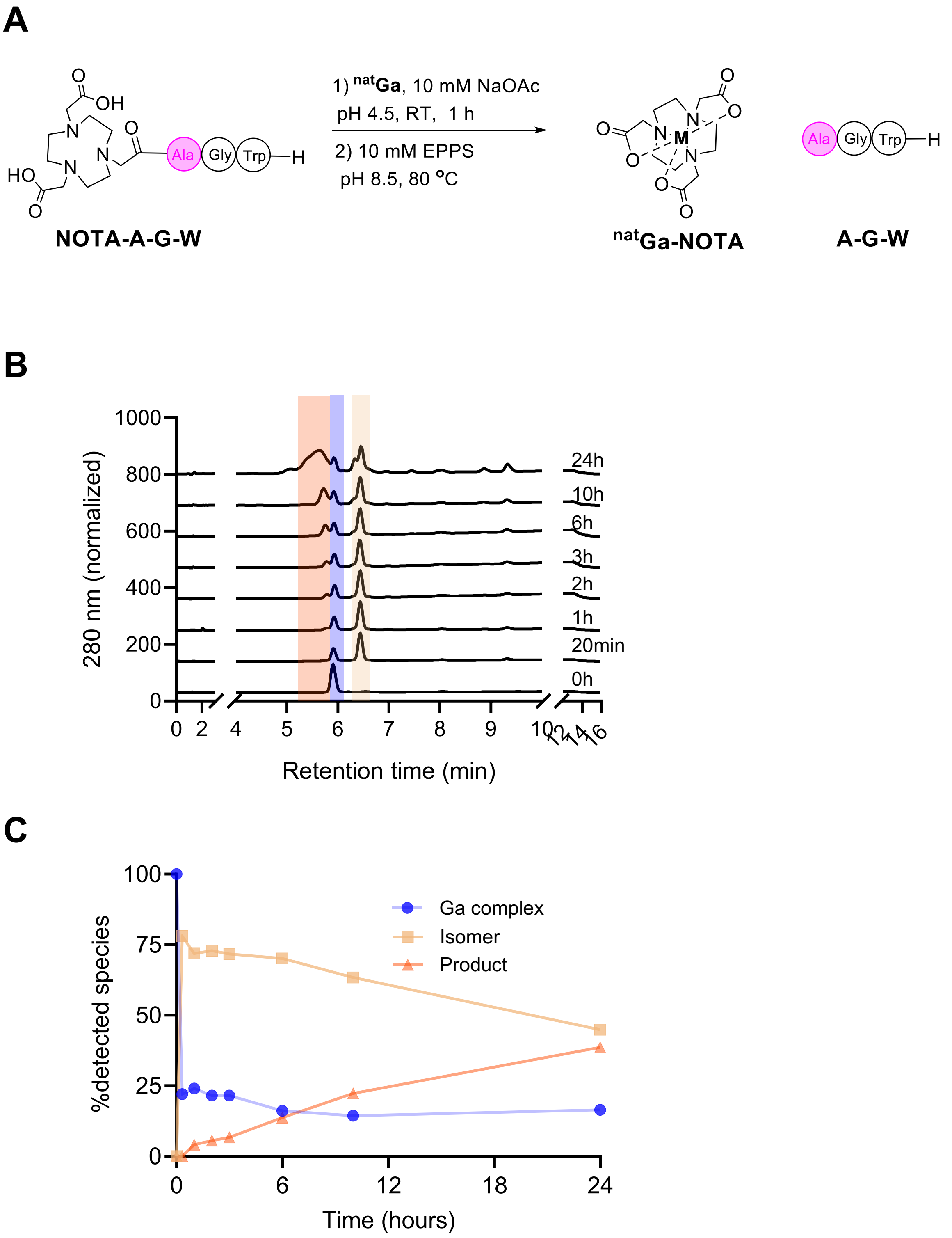

Figure S157. **(A)** Schematic description of preparation and direct complexation procedure of **[^{nat}Ga(12)]$^{+}$**. **(B)** Analytical HPLC chromatograms showing the autolytic release of the ^{nat}Ga-NOTA. **(C)** Quantification of the pH-dependent cleavage at 80 °C and pH 8.5.

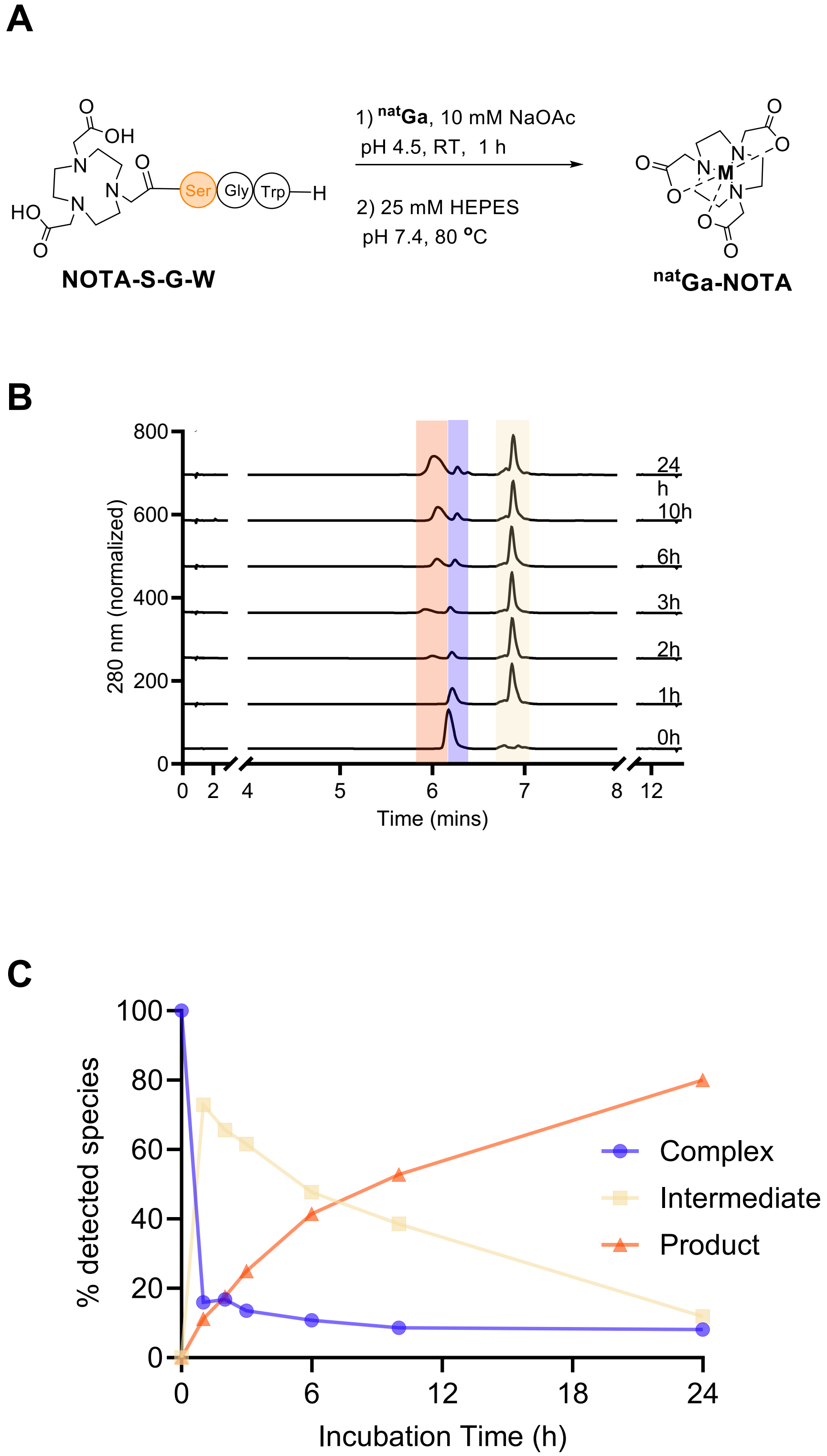

Figure S158. (A) Schematic description of preparation and direct complexation procedure of [^{nat}Ga(13)]$^{+}$. **(B)** Analytical HPLC chromatograms showing the autolytic release of the ^{nat}Ga-NOTA. **(C)** Quantification of the pH-dependent cleavage at 80 °C and pH 7.4.

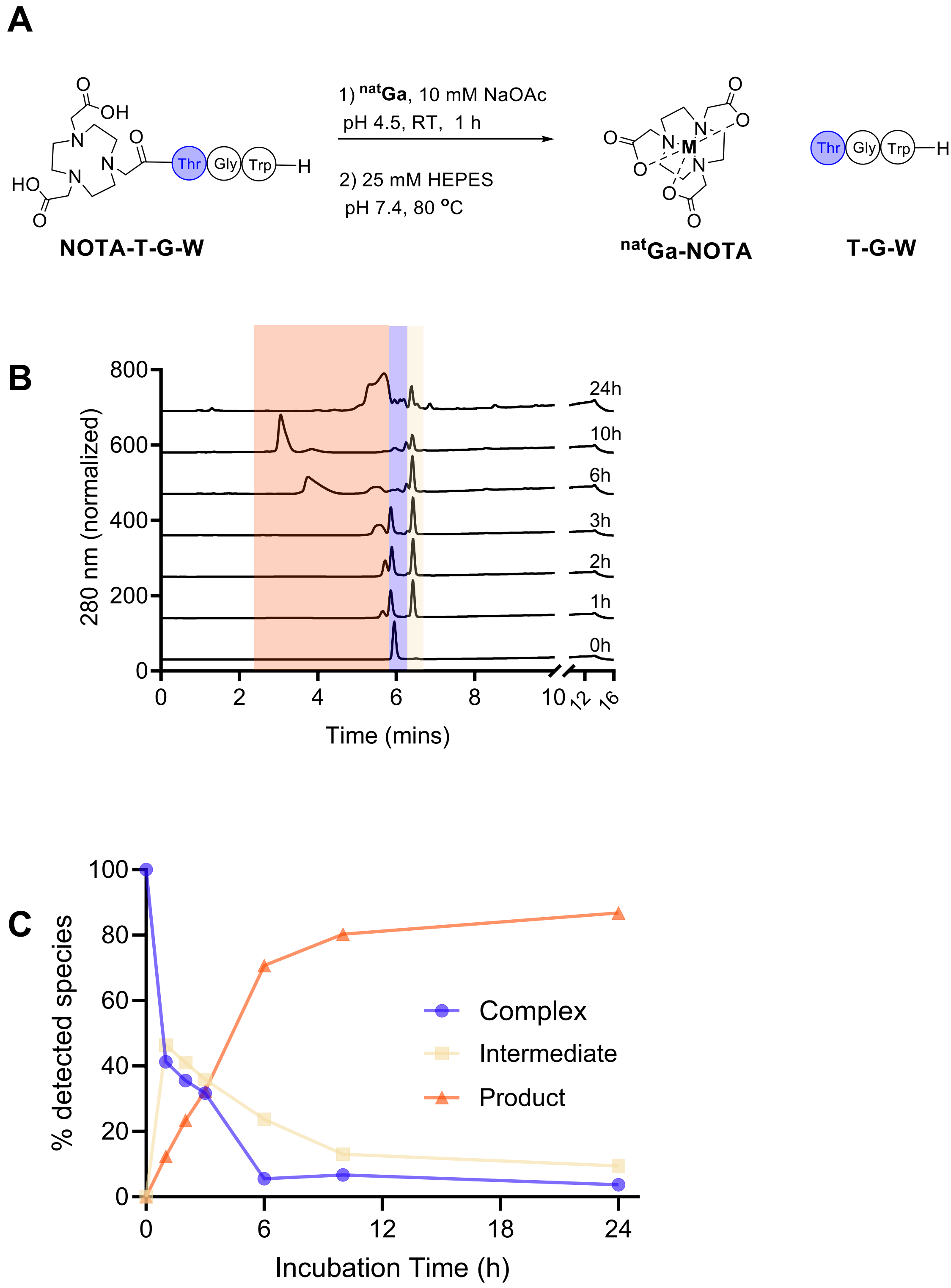

Figure S159. (A) Schematic description of preparation and direct complexation procedure of $[^{nat}Ga(14)]^{+}$. **(B)** Analytical HPLC chromatograms showing the autolytic release of the ^{nat}Ga-NOTA. **(C)** Quantification of the pH-dependent cleavage at 80 °C and pH 7.4.

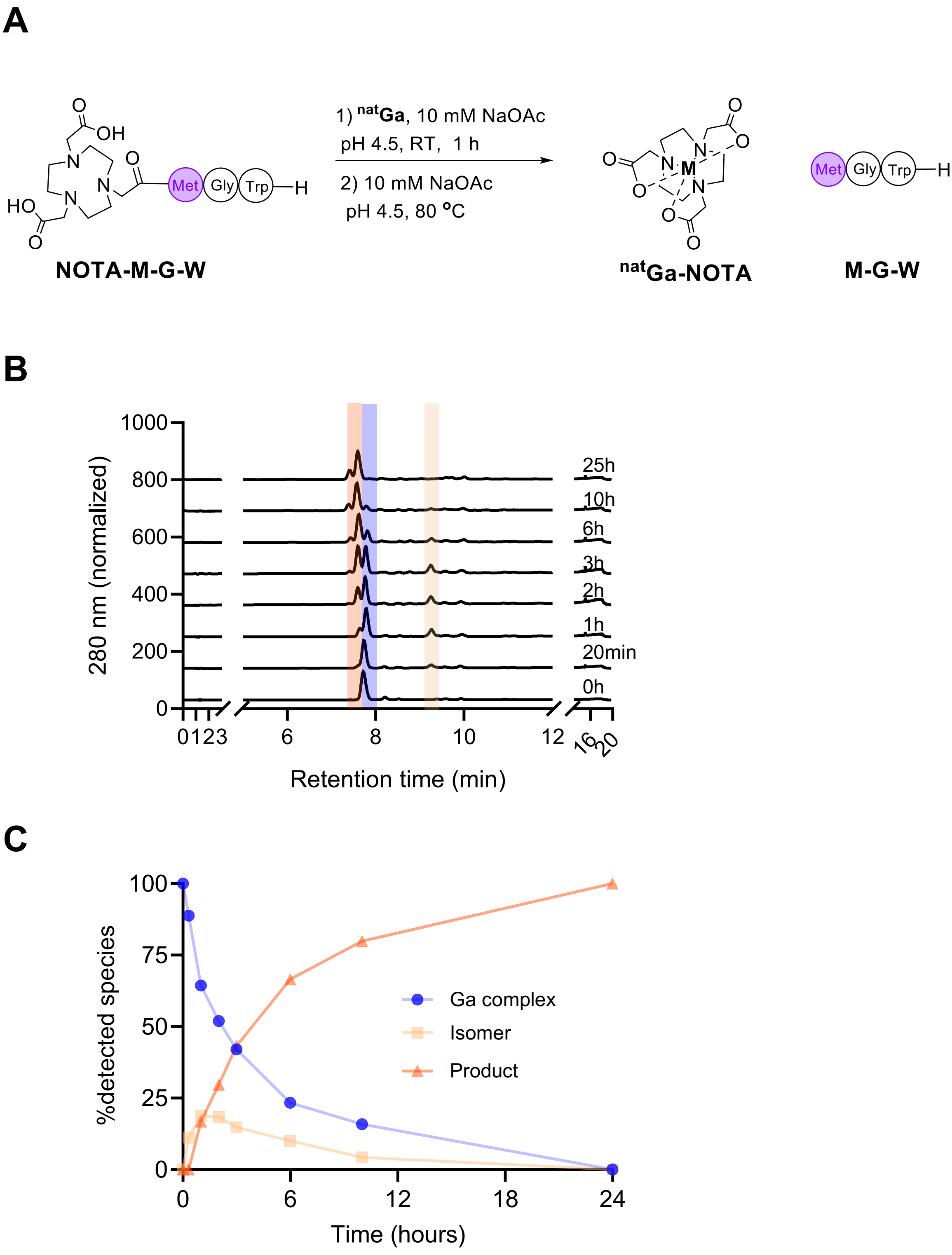

Figure S160. (A) Schematic description of preparation and direct complexation procedure of **[^{nat}Ga(15)]$^{+}$**. **(B)** Analytical HPLC chromatograms showing the autolytic release of the ^{nat}Ga-NOTA. **(C)** Quantification of the pH-dependent cleavage at 80 °C and pH 4.5.

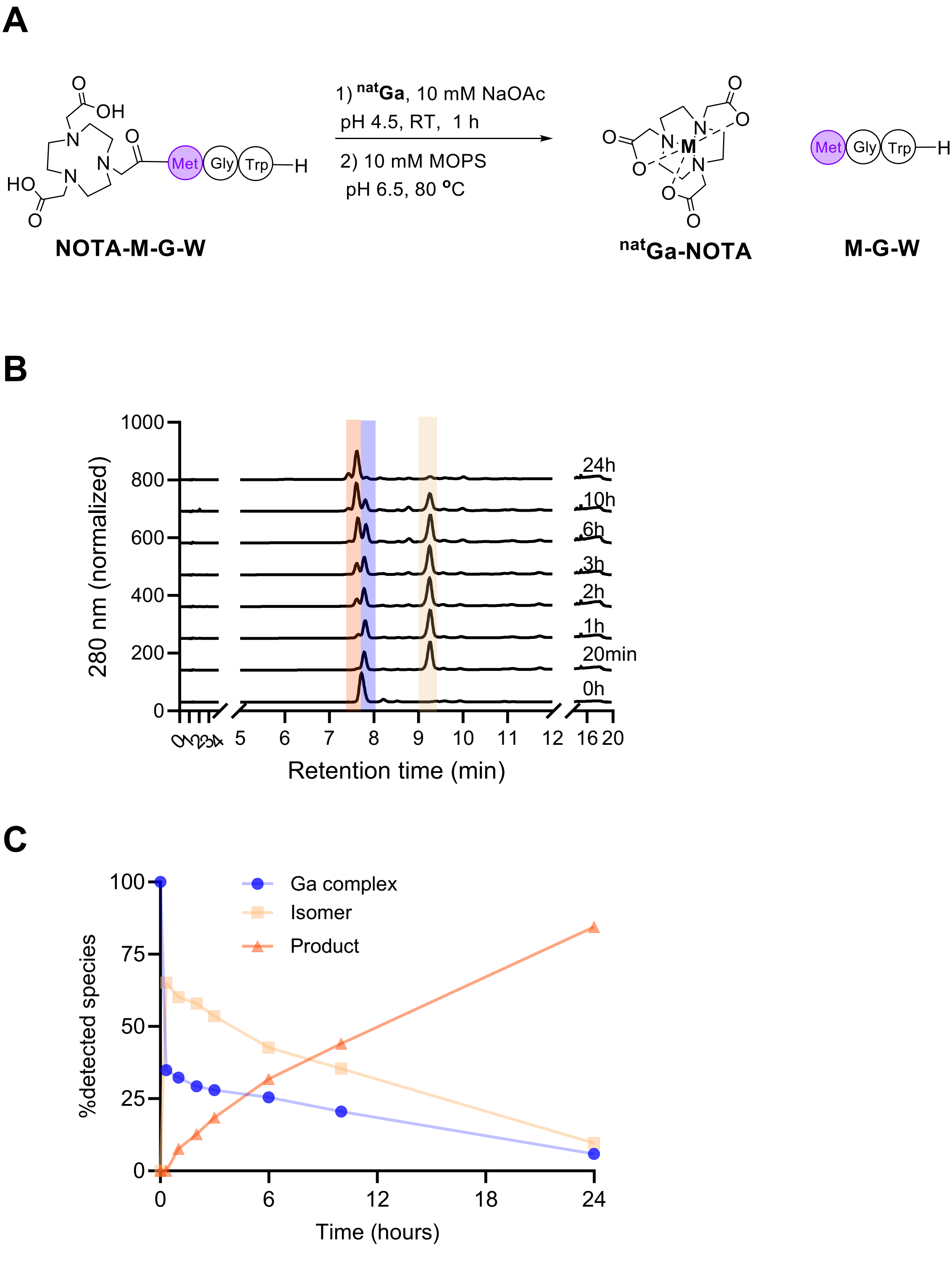

Figure S161. (A) Schematic description of preparation and direct complexation procedure of **[natGa(15)]+**. **(B)** Analytical HPLC chromatograms showing the autolytic release of the natGa-NOTA. **(C)** Quantification of the pH-dependent cleavage at 80 °C and pH 6.5.

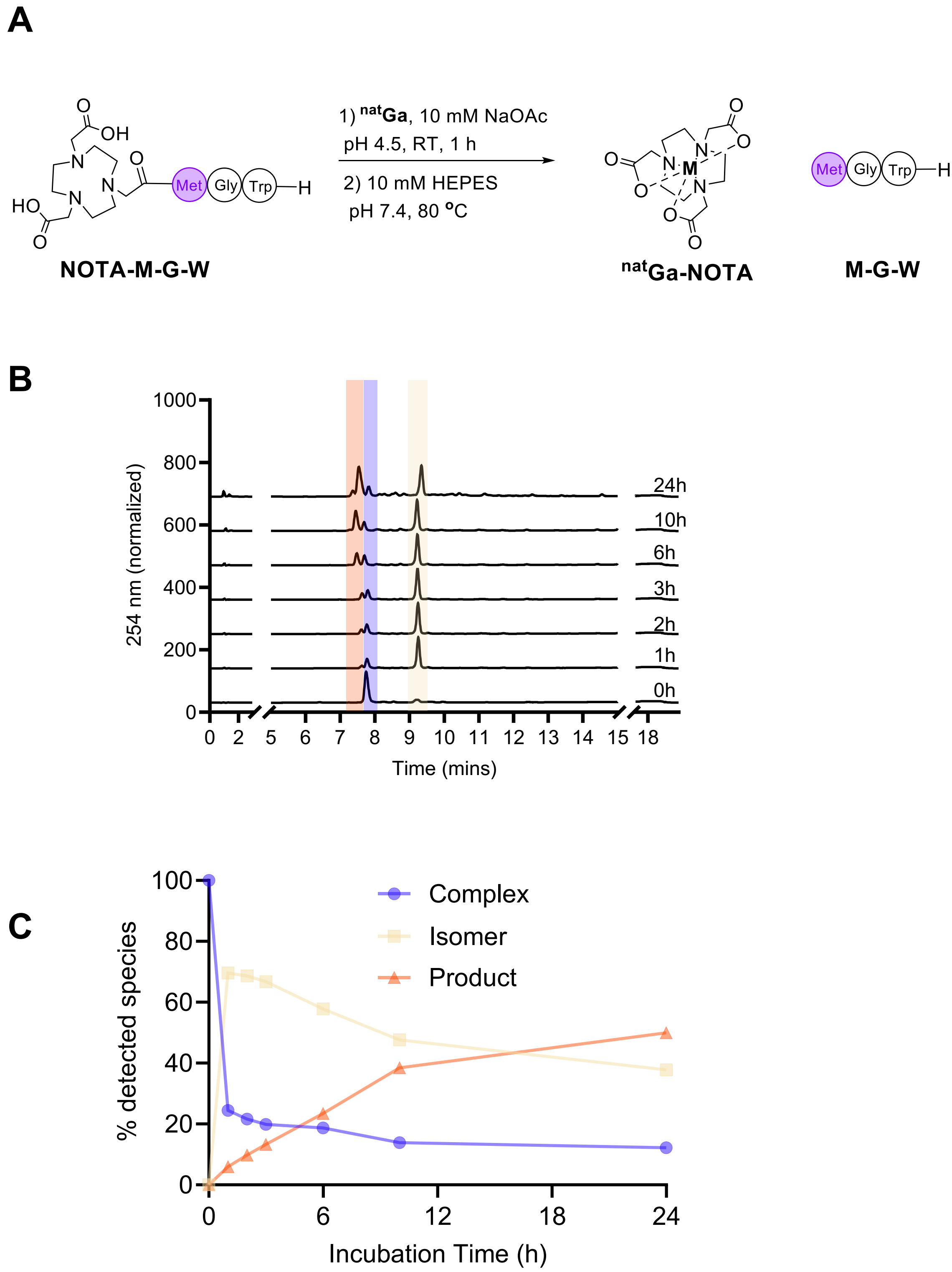

Figure S162. (A) Schematic description of preparation and direct complexation procedure of **[natGa(15)]**$^{+}$. **(B)** Analytical HPLC chromatograms showing the autolytic release of the natGa-NOTA. **(C)** Quantification of the pH-dependent cleavage at 80 °C and pH 7.4.

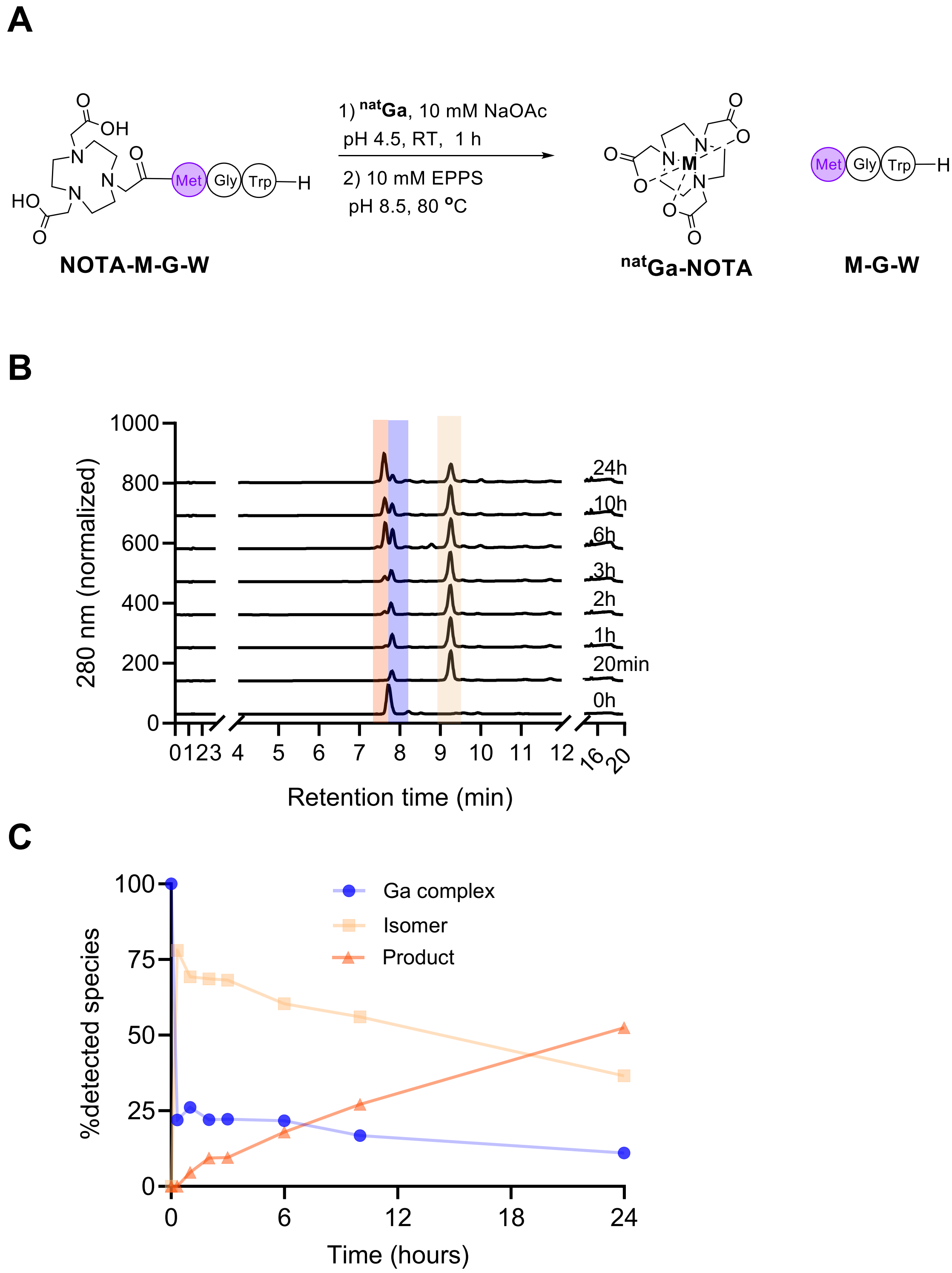

Figure S163. (A) Schematic description of preparation and direct complexation procedure of **[^{nat}Ga(15)]**$^+$. **(B)** Analytical HPLC chromatograms showing the autolytic release of the ^{nat}Ga-NOTA. **(C)** Quantification of the pH-dependent cleavage at 80 °C and pH 8.5.

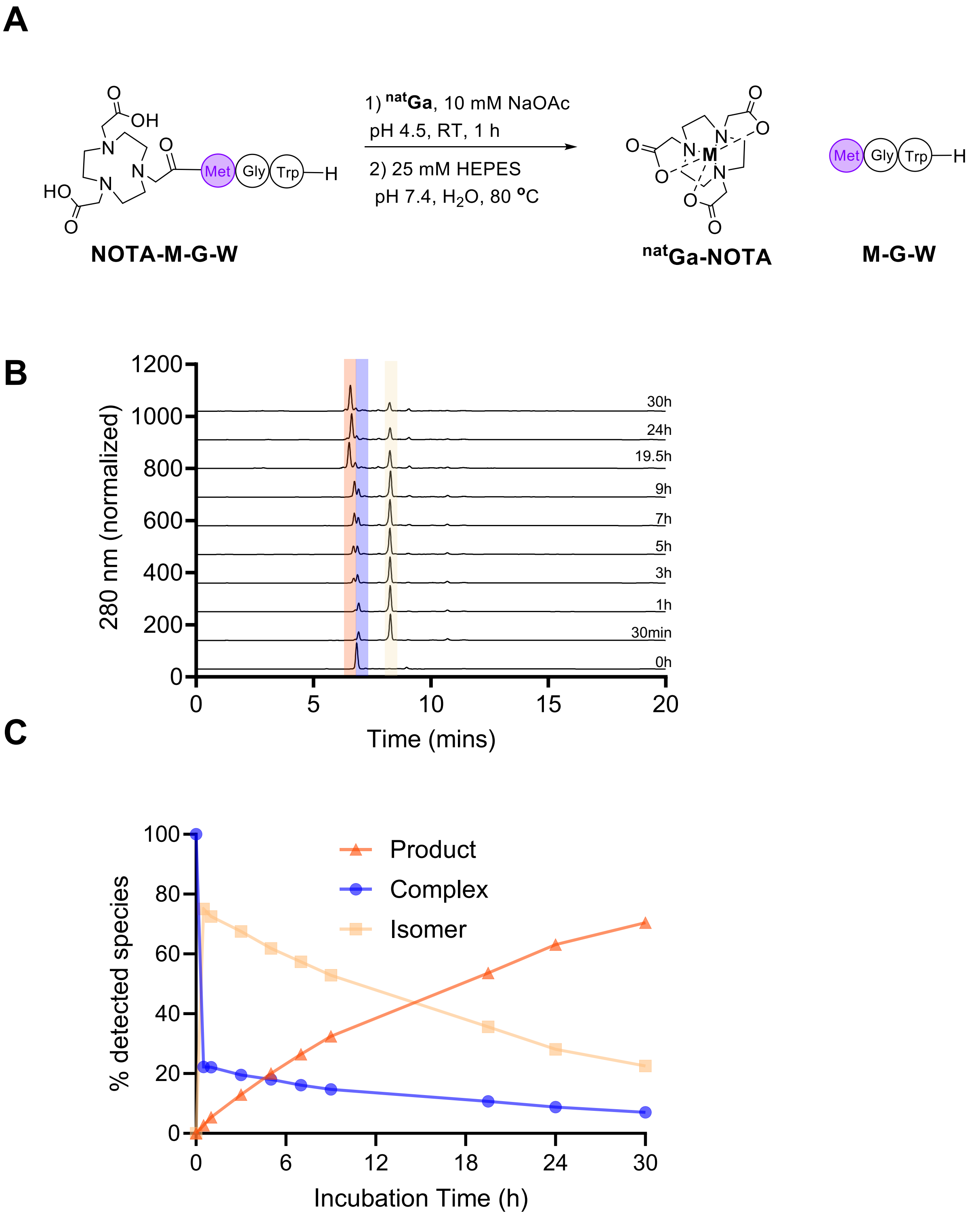

Figure S164. (A) Schematic description of preparation and direct complexation procedure of **[^{nat}Ga(15)]$^{+}$**. **(B)** Analytical HPLC chromatograms showing the autolytic release of the ^{nat}Ga-NOTA. **(C)** Quantification of the pH-dependent cleavage at 80 °C and 25 mM pH 7.4 HEPES buffer.

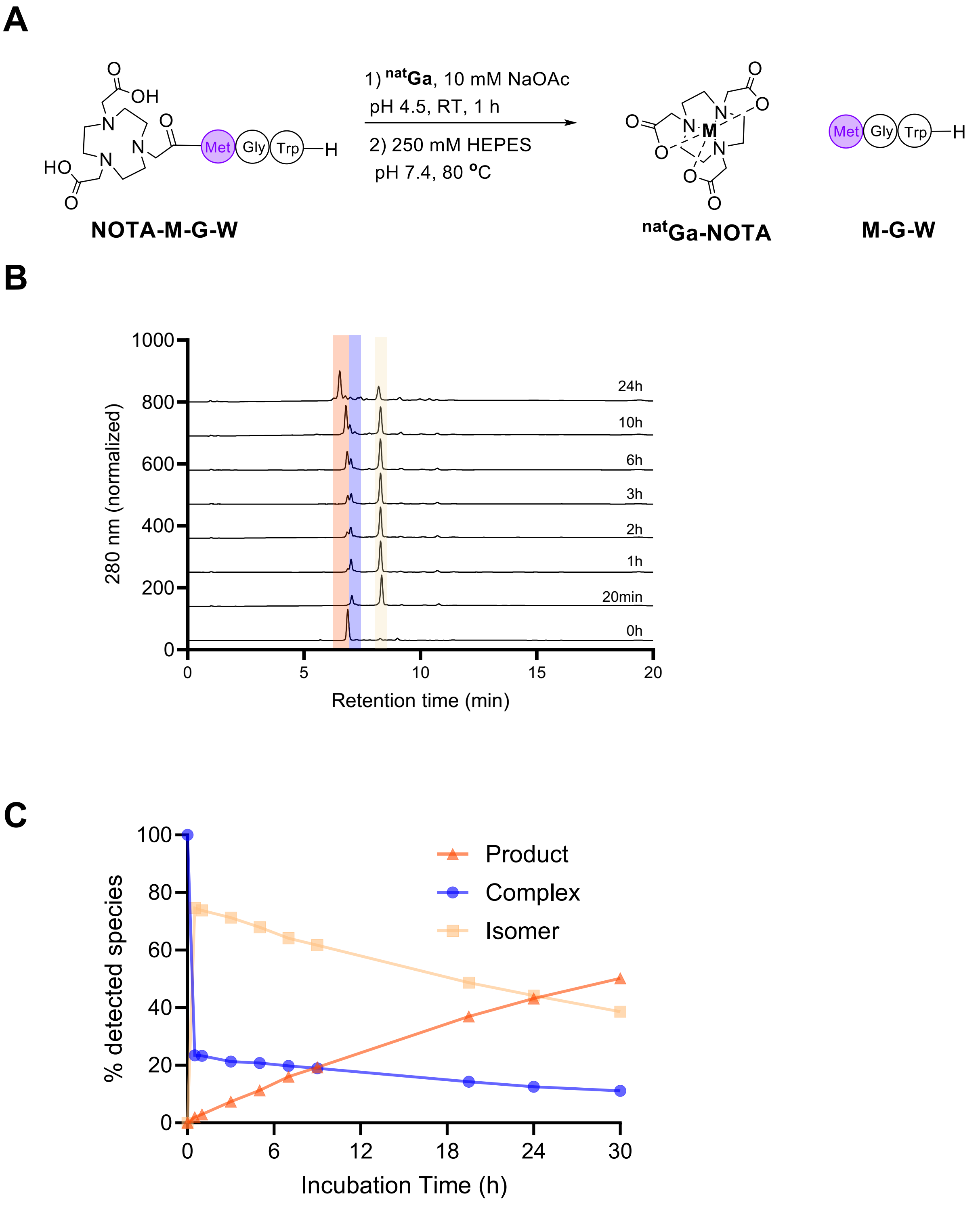

Figure S165. (A) Schematic description of preparation and direct complexation procedure of **[natGa(15)]**$^{+}$. **(B)** Analytical HPLC chromatograms showing the autolytic release of the natGa-NOTA. **(C)** Quantification of the pH-dependent cleavage at 80 °C and 250 mM pH 7.4 HEPES buffer.

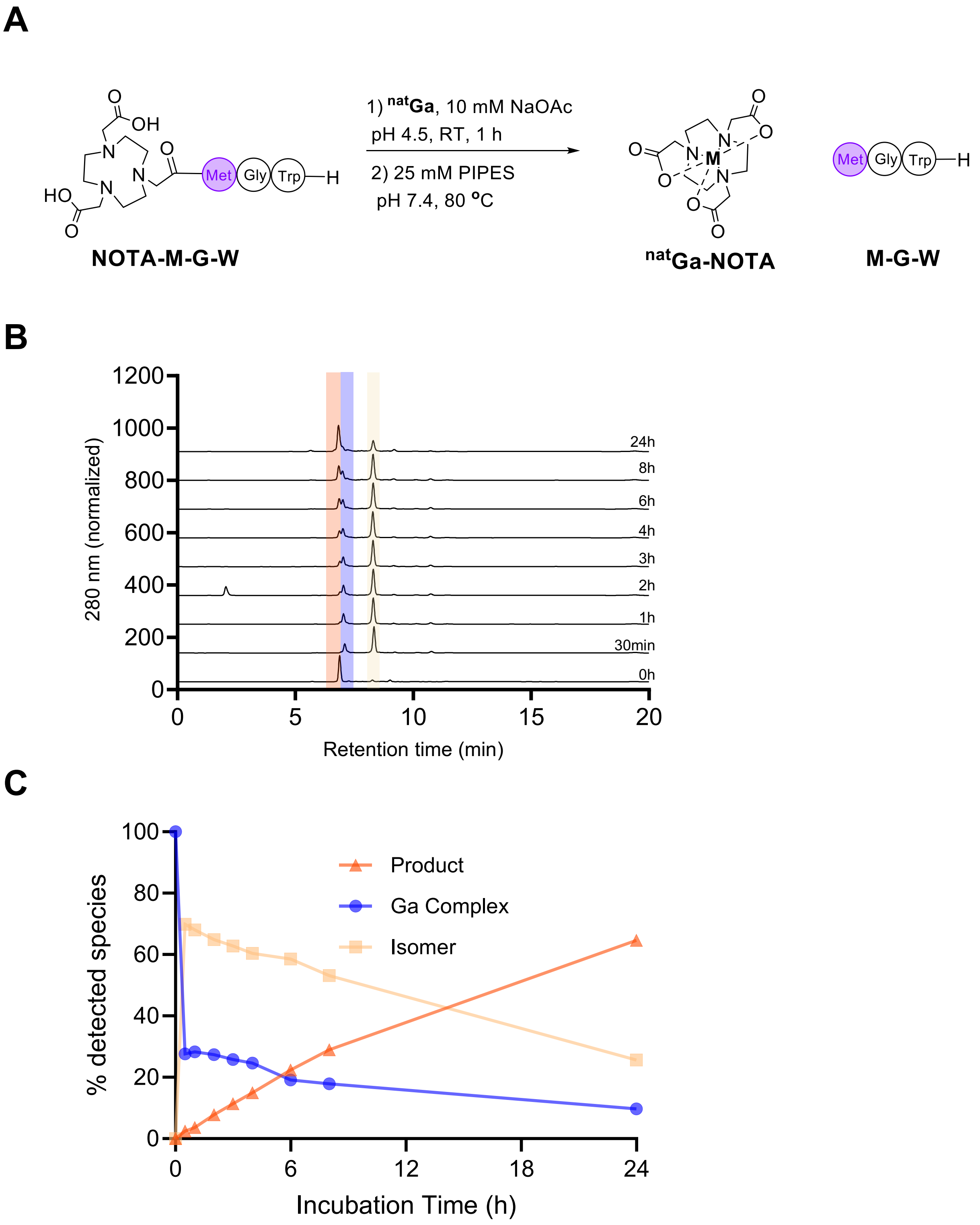

Figure S166. (A) Schematic description of preparation and direct complexation procedure of **$[^{nat}Ga(15)]^{+}$**. **(B)** Analytical HPLC chromatograms showing the autolytic release of the ^{nat}Ga-NOTA. **(C)** Quantification of the pH-dependent cleavage at 80 °C and 25 mM pH 7.4 PIPES buffer.

A

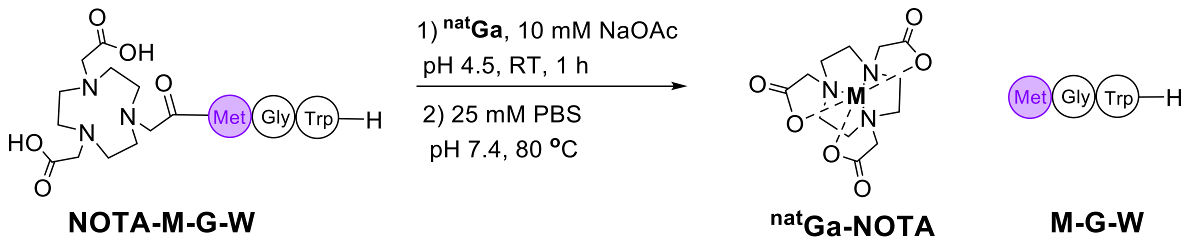

B

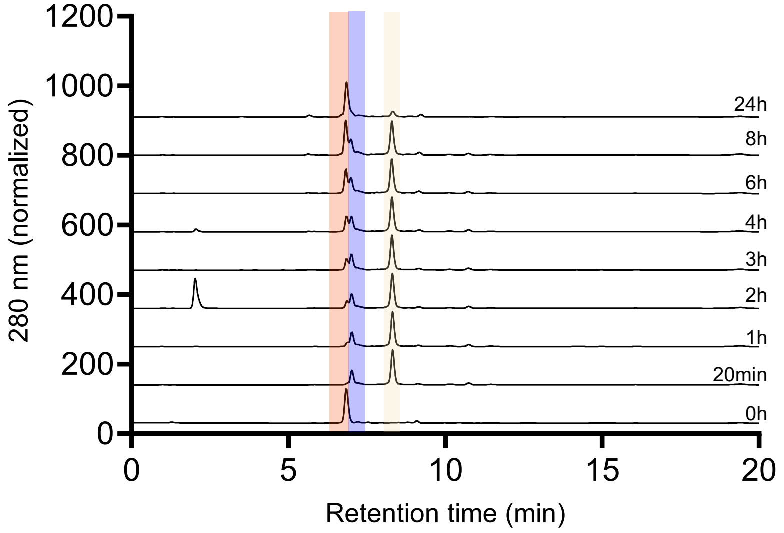

C

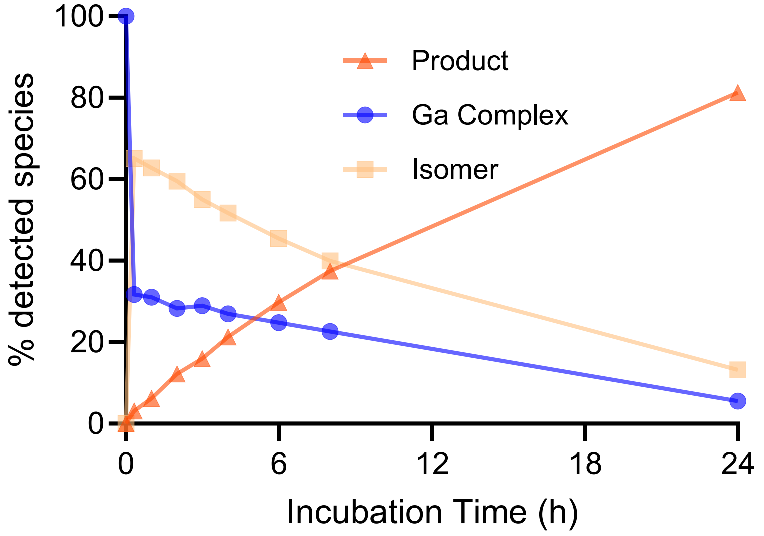

Figure S167. (A) Schematic description of preparation and direct complexation procedure of **[^{nat}Ga(15)]$^{+}$**. **(B)** Analytical HPLC chromatograms showing the autolytic release of the ^{nat}Ga-NOTA. **(C)** Quantification of the pH-dependent cleavage at 80 °C and 25 mM pH 7.4 PBS buffer.

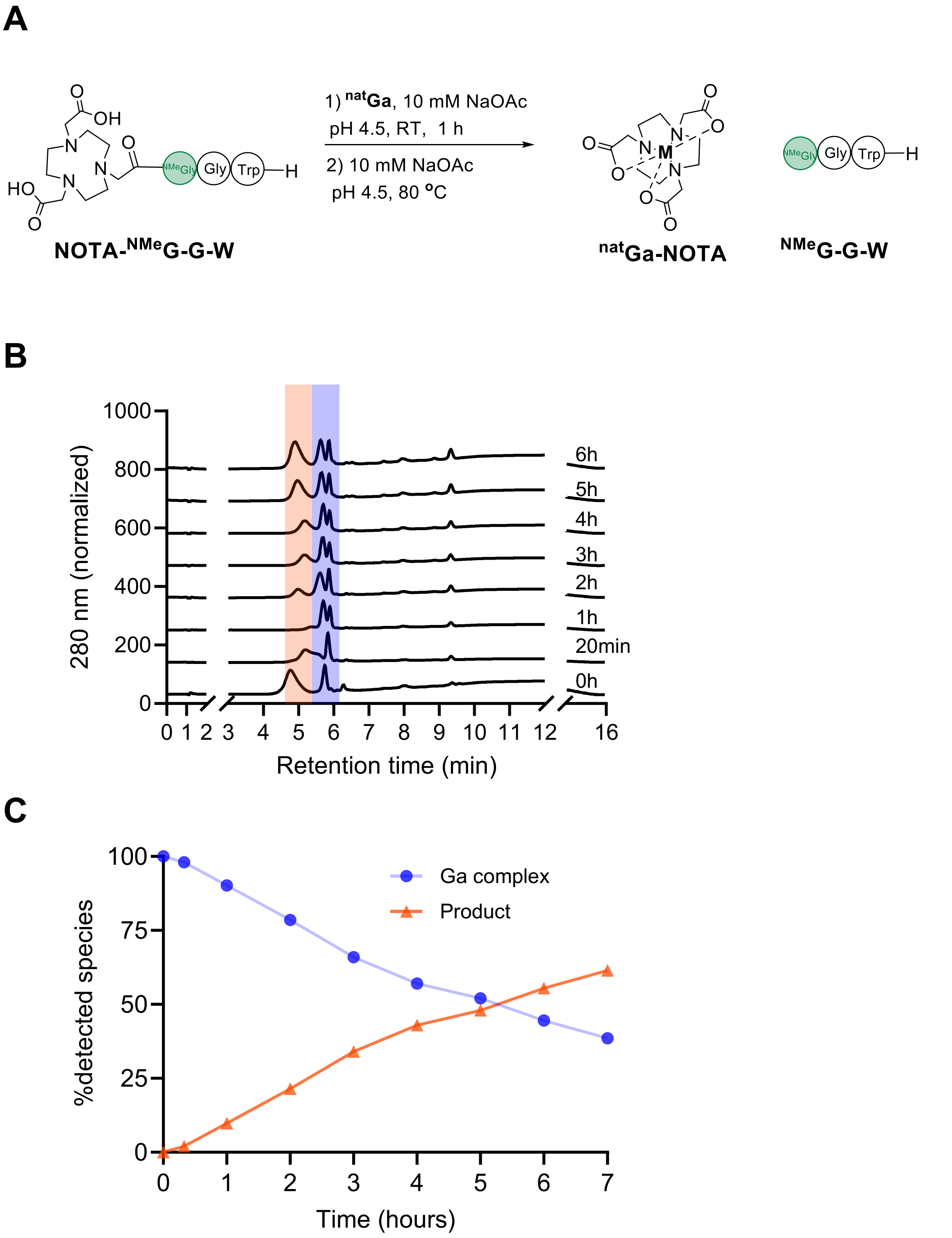

Figure S168. (A) Schematic description of preparation and direct complexation procedure of **[^{nat}Ga(17)]$^{+}$**. **(B)** Analytical HPLC chromatograms showing the autolytic release of the ^{nat}Ga-NOTA. **(C)** Quantification of the pH-dependent cleavage at 80 °C and pH 4.5.

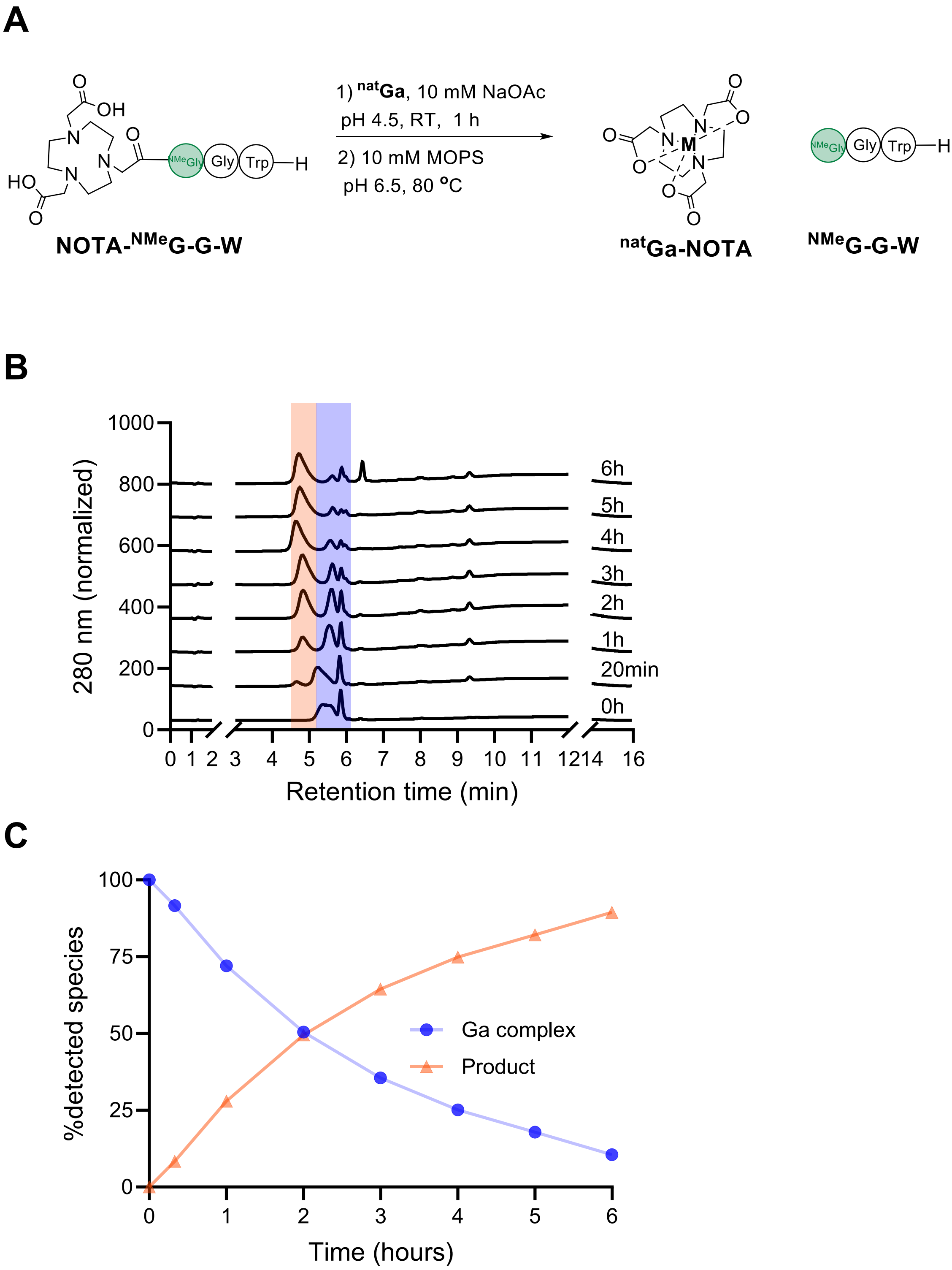

Figure S169. (A) Schematic description of preparation and direct complexation procedure of **[^{nat}Ga(17)]$^{+}$**. **(B)** Analytical HPLC chromatograms showing the autolytic release of the ^{nat}Ga-NOTA. (C) Quantification of the pH-dependent cleavage at 80 °C and pH 6.5.

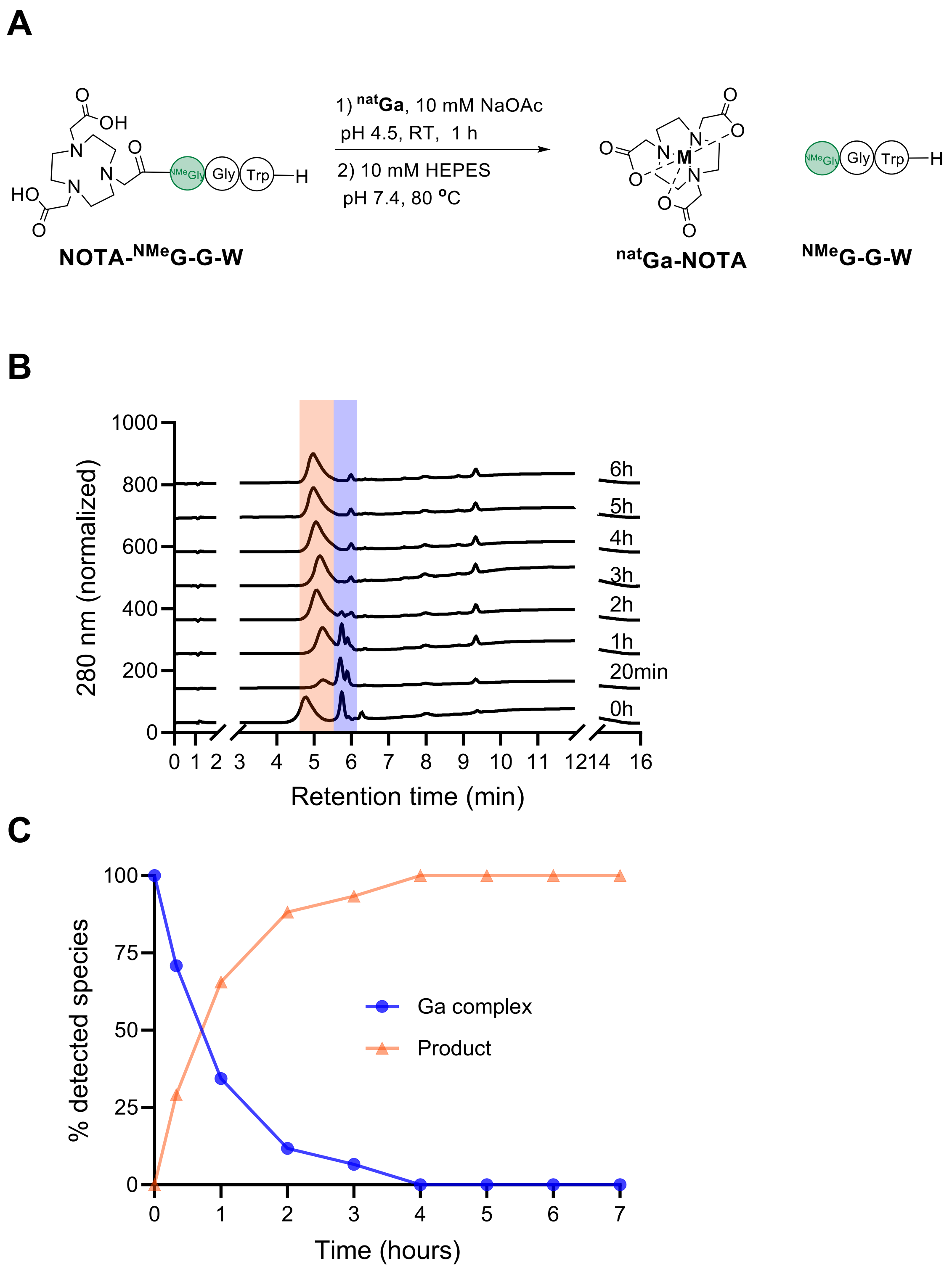

Figure S170. (A) Schematic description of preparation and direct complexation procedure of **[^{nat}Ga(17)]$^{+}$**. **(B)** Analytical HPLC chromatograms showing the autolytic release of the ^{nat}Ga-NOTA. (C) Quantification of the pH-dependent cleavage at 80 °C and pH 7.4.

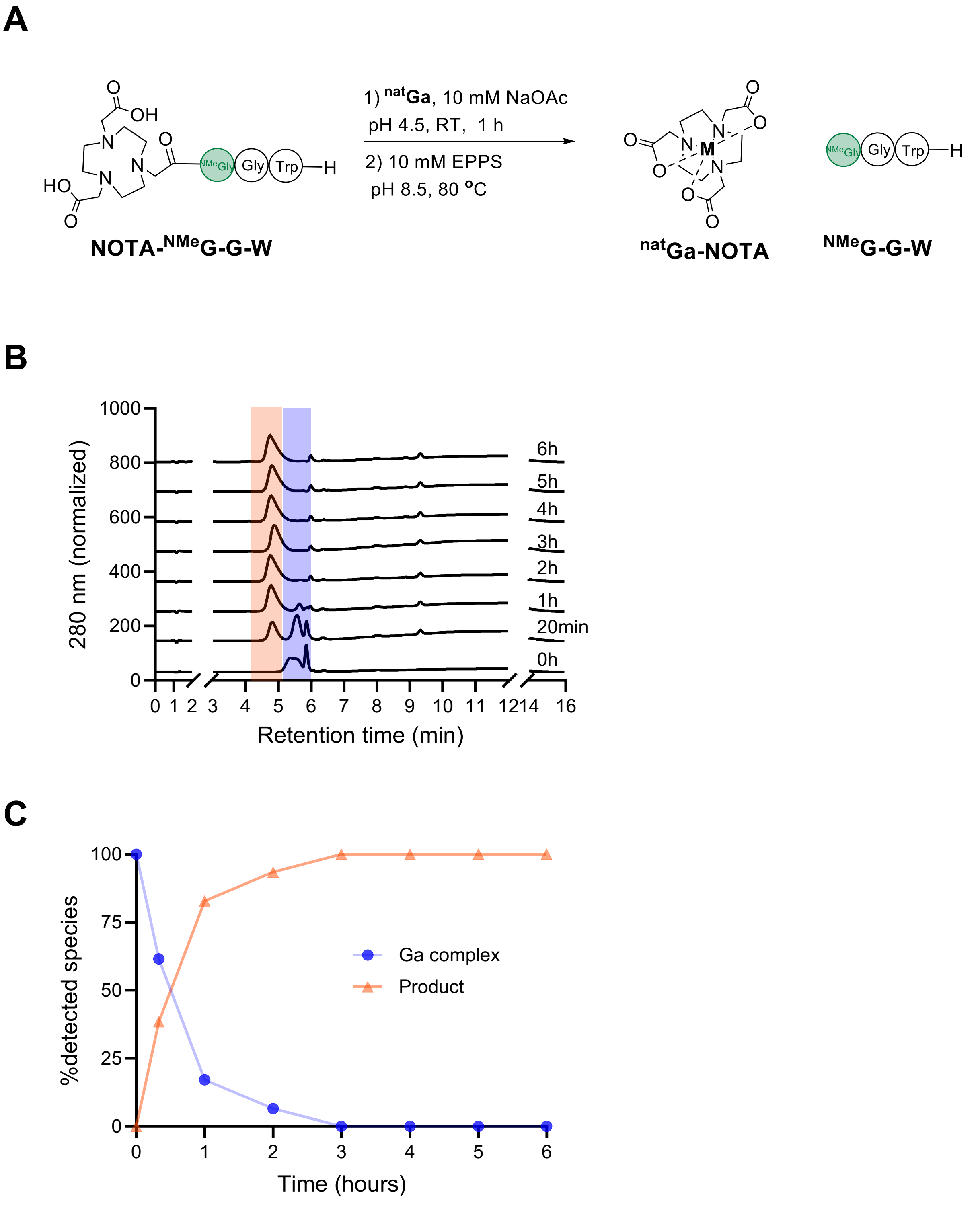

Figure S171. (A) Schematic description of preparation and direct complexation procedure of **[natGa(17)]**$^{+}$. **(B)** Analytical HPLC chromatograms showing the autolytic release of the natGa-NOTA. **(C)** Quantification of the pH-dependent cleavage at 80 °C and pH 8.5.

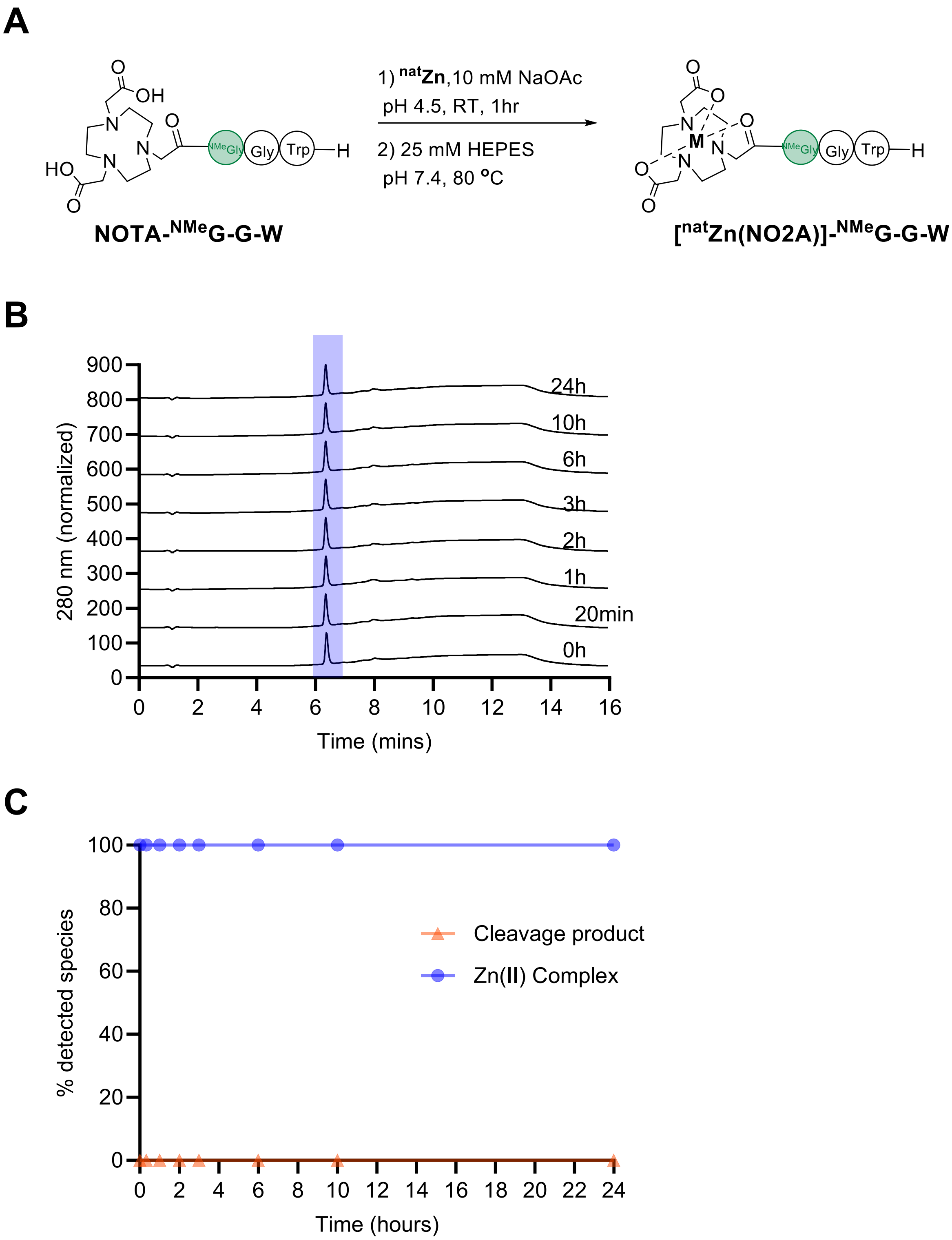

Figure S172. (A) Schematic description of preparation and direct complexation procedure of **[^{nat}Zn(17)]**. **(B)** Analytical HPLC chromatograms showing no autolytic release of the ^{nat}Zn-NOTA. **(C)** Quantification of the pH-dependent cleavage at 80 °C and pH 7.4.

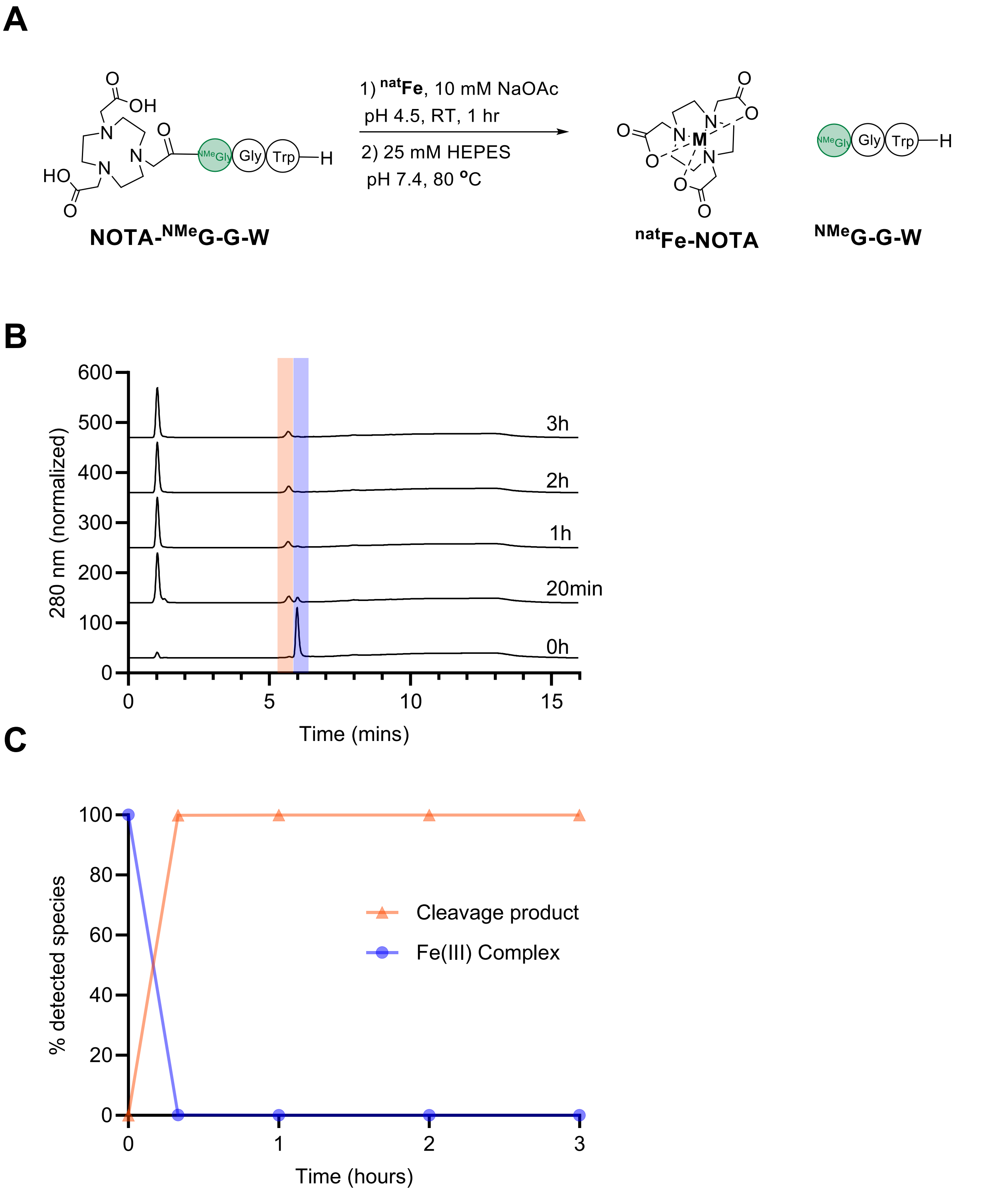

Figure S173. (A) Schematic description of preparation and direct complexation procedure of **$[^{nat}Fe(17)]^{+}$**. **(B)** Analytical HPLC chromatograms showing the autolytic release of the ^{nat}Fe-NOTA. **(C)** Quantification of the pH-dependent cleavage at 80 °C and pH 7.4.

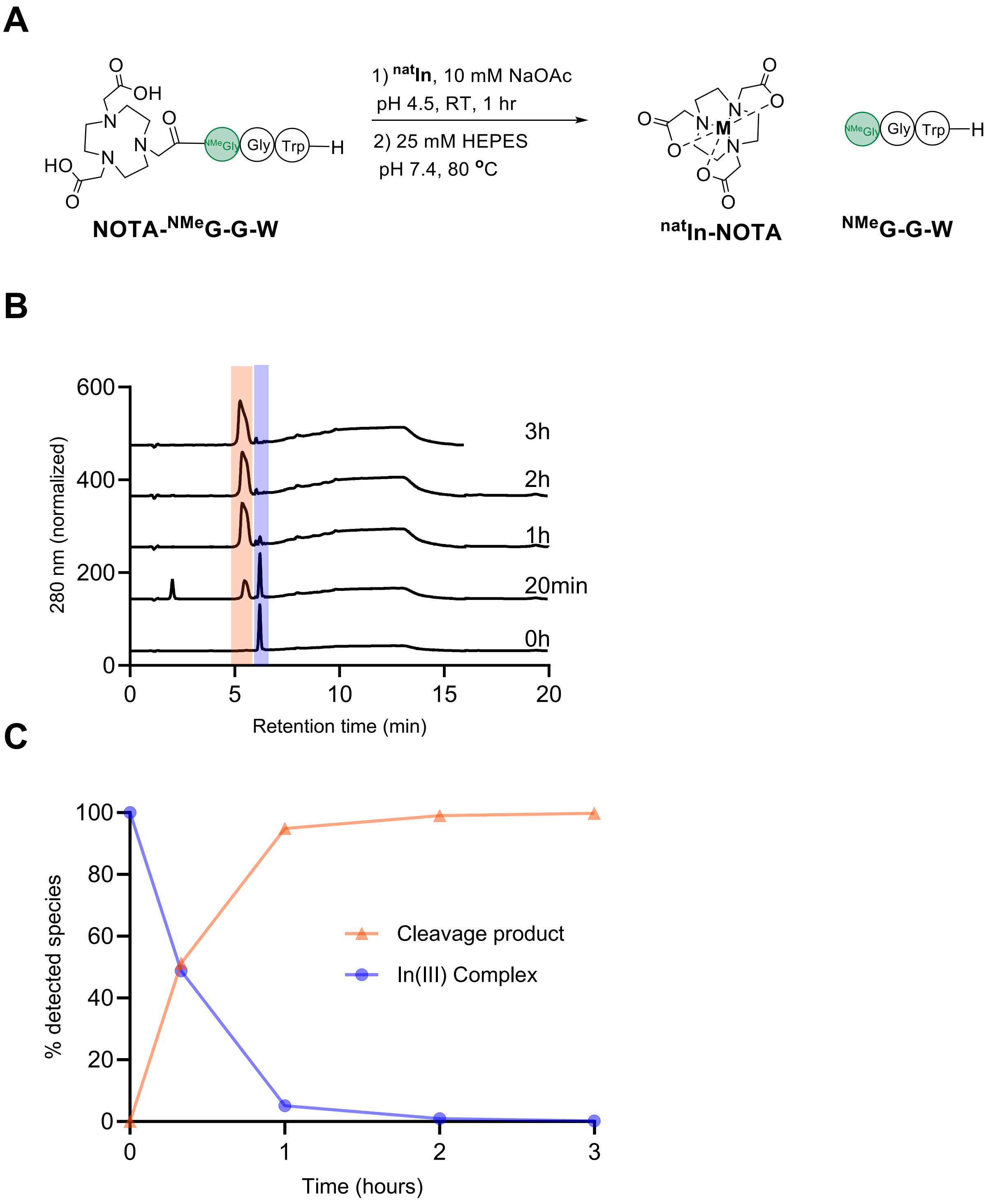

Figure S174. (A) Schematic description of preparation and direct complexation procedure of **$[^{nat}In(17)]^{+}$**. **(B)** Analytical HPLC chromatograms showing the autolytic release of the ^{nat}In-NOTA. **(C)** Quantification of the pH-dependent cleavage at 80 °C and pH 7.4.

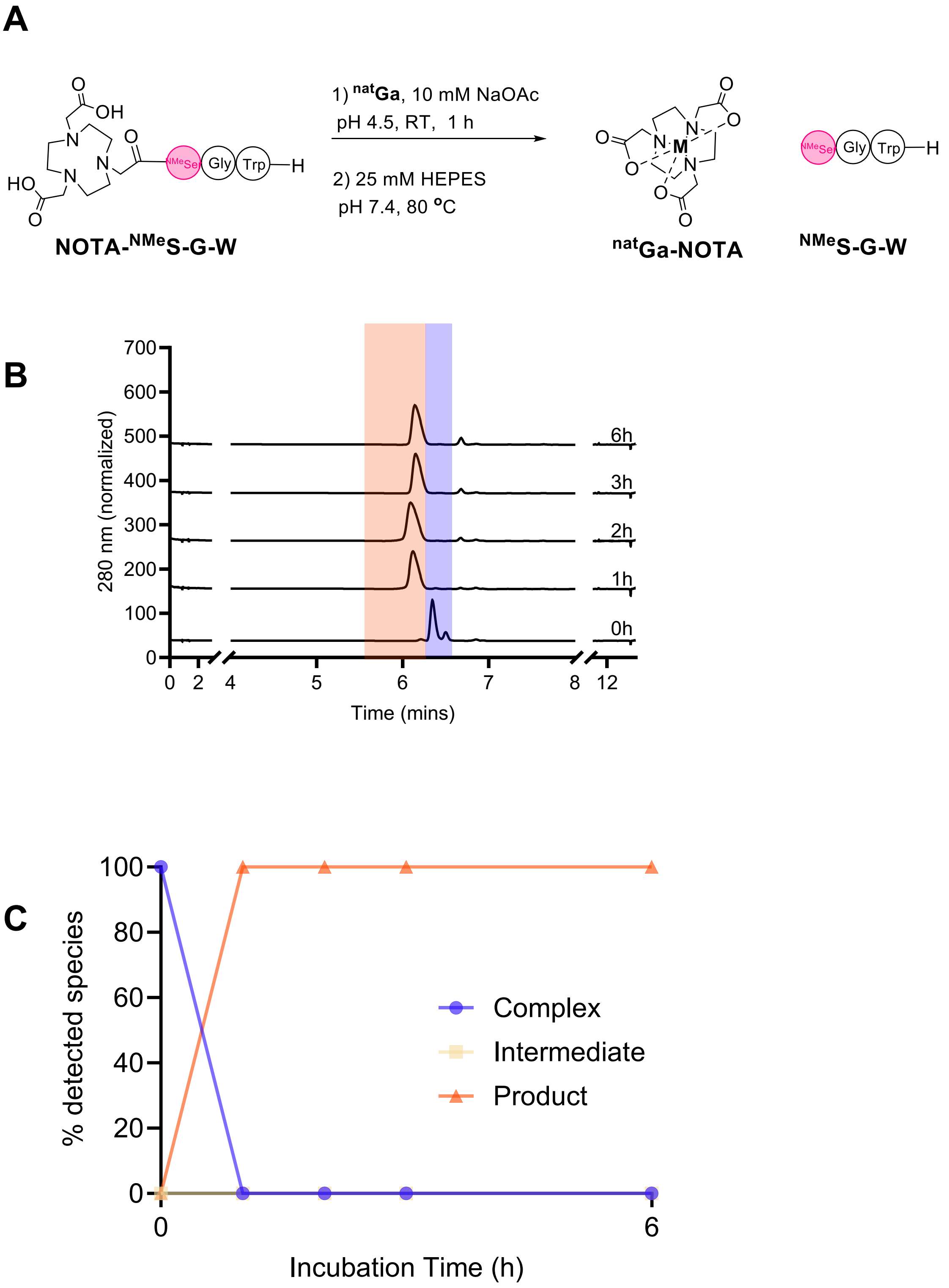

Figure S175. **(A)** Schematic description of preparation and direct complexation procedure of **[natGa(18)]**$^{+}$. **(B)** Analytical HPLC chromatograms showing the autolytic release of the natGa-NOTA. **(C)** Quantification of the pH-dependent cleavage at 80 °C and pH 7.4.

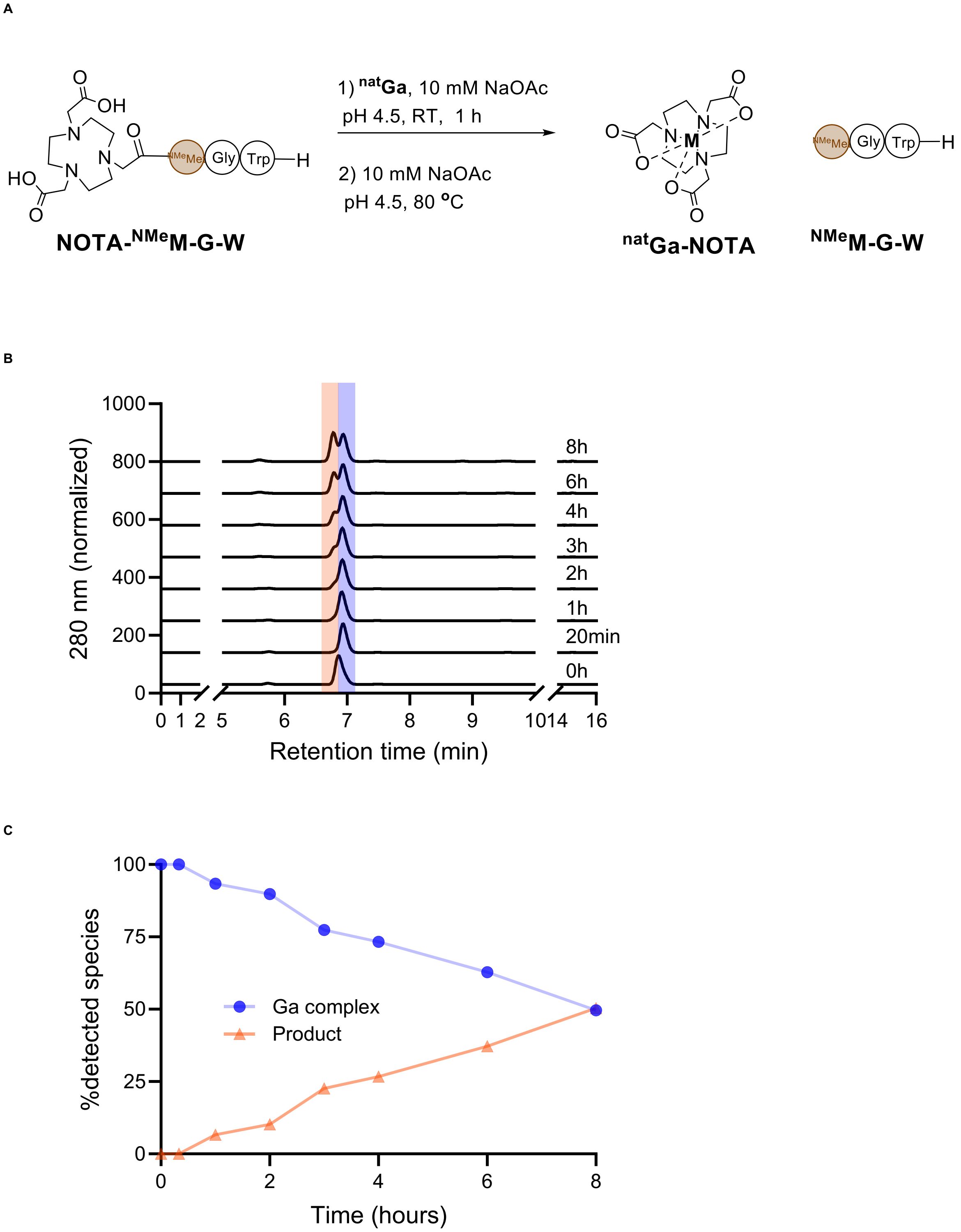

Figure S176. (A) Schematic description of preparation and direct complexation procedure of **[^{nat}Ga(19)]$^{+}$**. **(B)** Analytical HPLC chromatograms showing the autolytic release of the ^{nat}Ga-NOTA. **(C)** Quantification of the pH-dependent cleavage at 80 °C and pH 4.5.

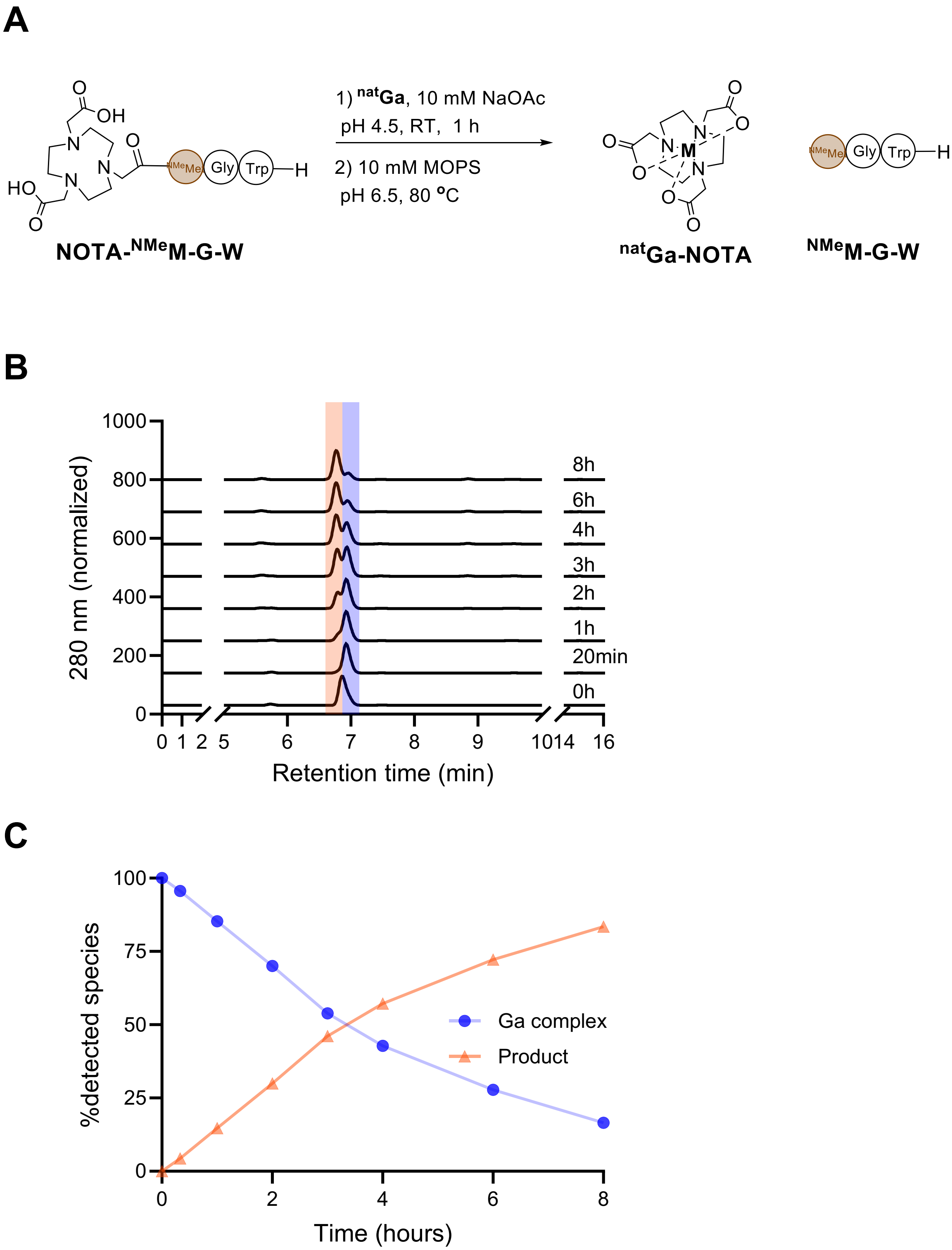

Figure S177. (A) Schematic description of preparation and direct complexation procedure of **[^{nat}Ga(19)]$^{+}$**. **(B)** Analytical HPLC chromatograms showing the autolytic release of the ^{nat}Ga-NOTA. **(C)** Quantification of the pH-dependent cleavage at 80 °C and pH 6.5.

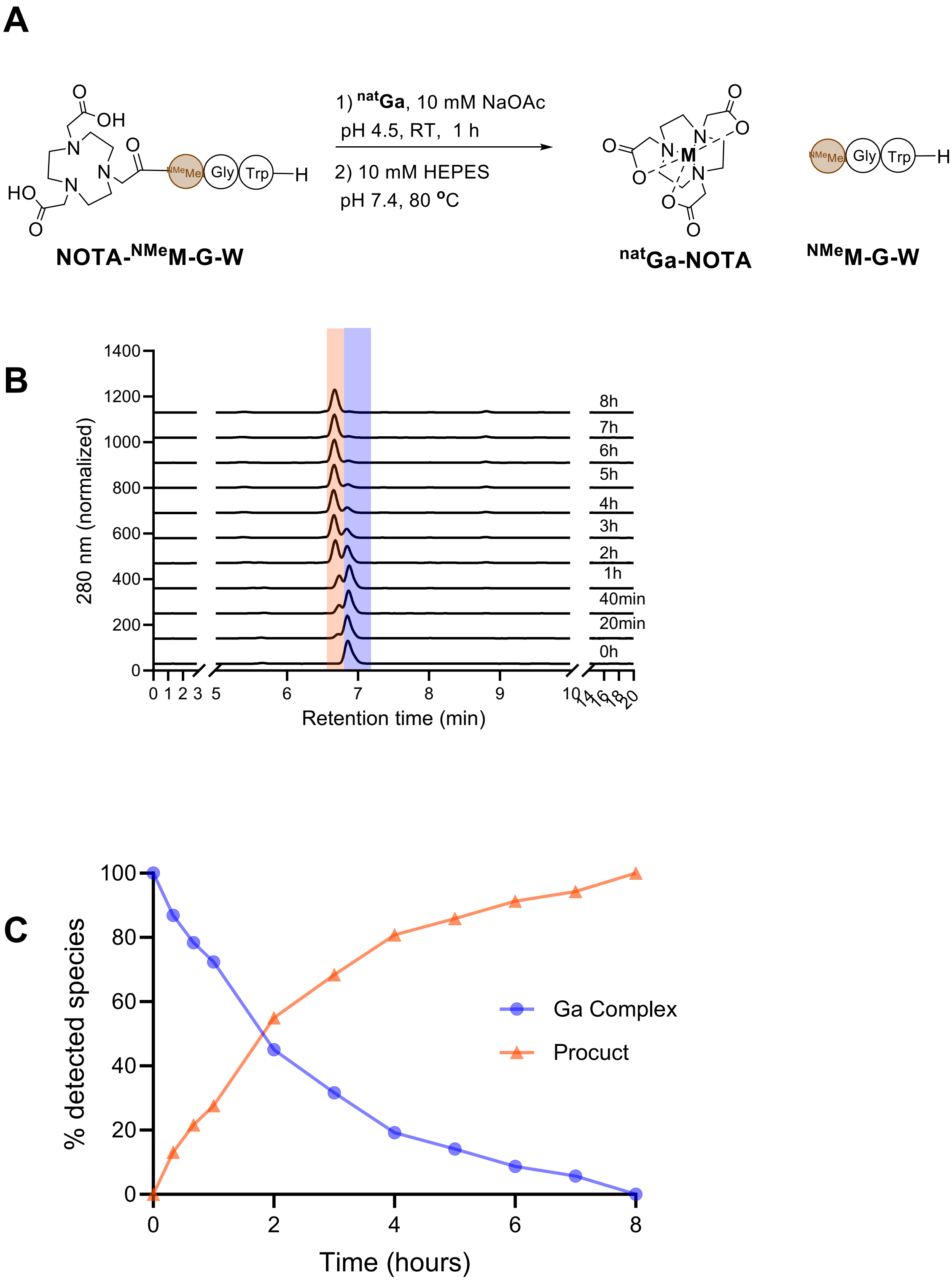

Figure S178. (A) Schematic description of preparation and direct complexation procedure of **[natGa(19)]**$^{+}$. **(B)** Analytical HPLC chromatograms showing the autolytic release of the natGa-NOTA. **(C)** Quantification of the pH-dependent cleavage at 80 °C and pH 7.4.

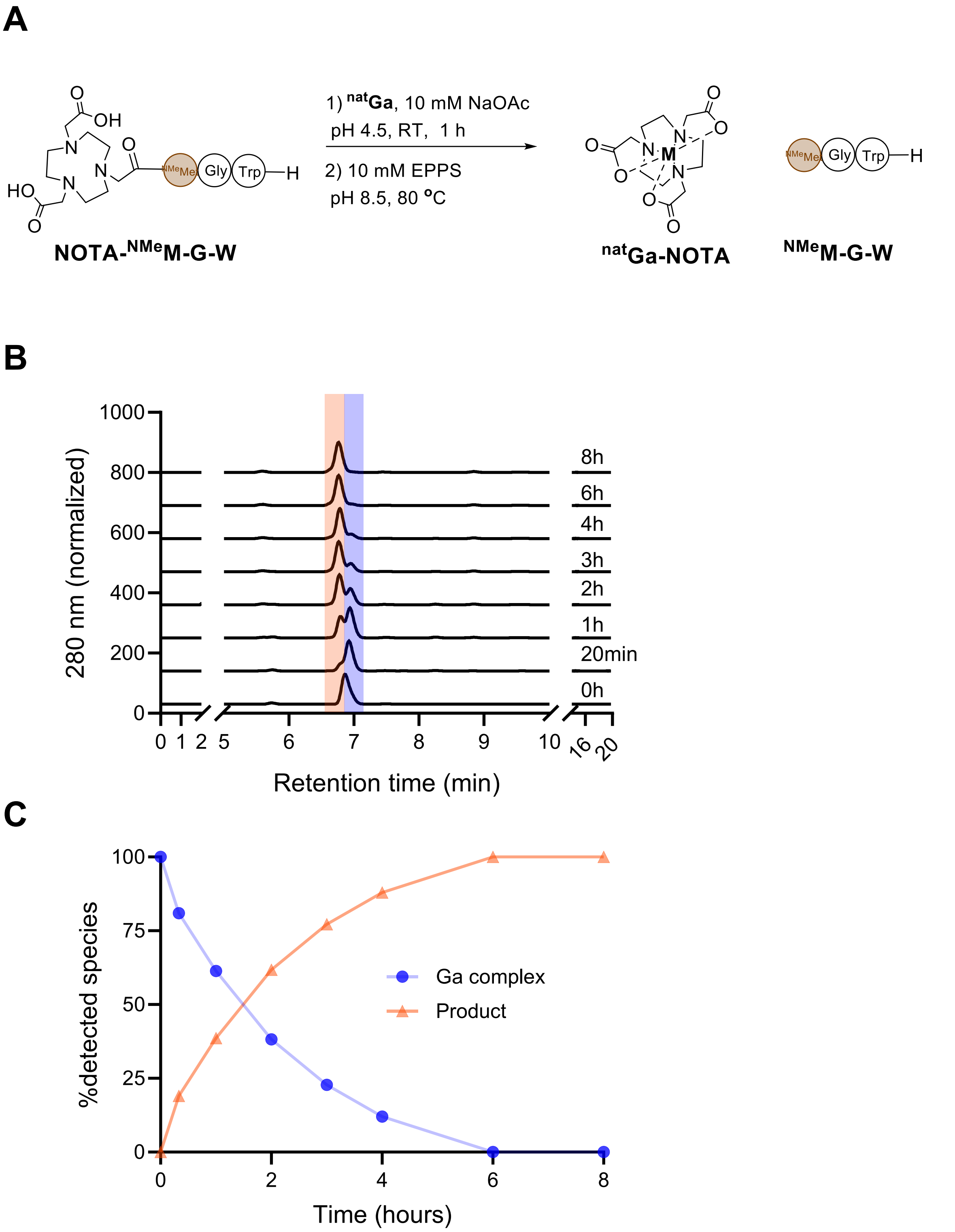

Figure S179. (A) Schematic description of preparation and direct complexation procedure of **$[^{nat}Ga(19)]^{+}$**. **(B)** Analytical HPLC chromatograms showing the autolytic release of the ^{nat}Ga-NOTA. **(C)** Quantification of the pH-dependent cleavage at 80 °C and pH 8.5.

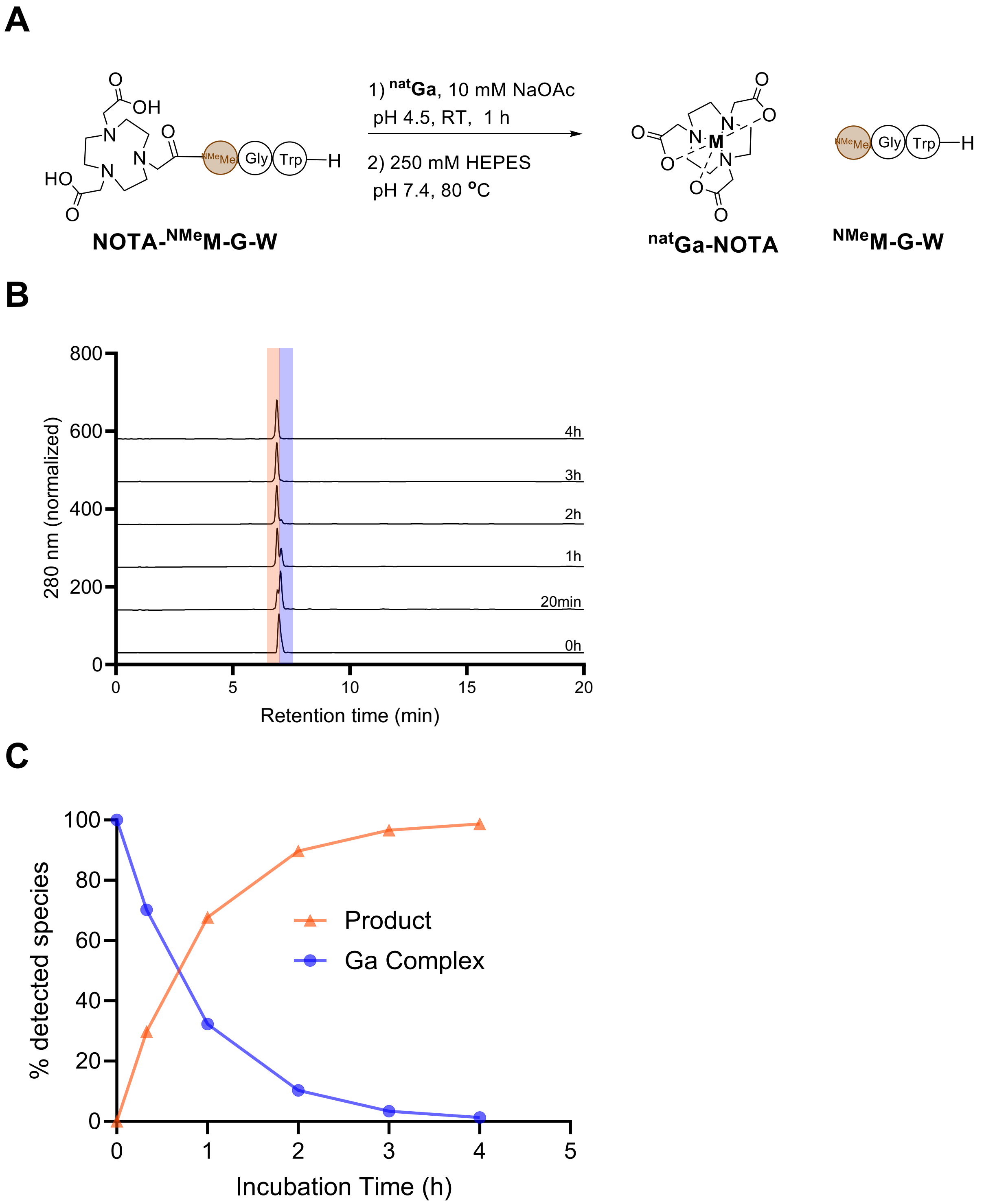

Figure S180. (A) Schematic description of preparation and direct complexation procedure of **[natGa(19)]$^{+}$**. **(B)** Analytical HPLC chromatograms showing the autolytic release of the natGa-NOTA. **(C)** Quantification of the pH-dependent cleavage at 80 °C and 250 mM pH 7.4 HEPES buffer.

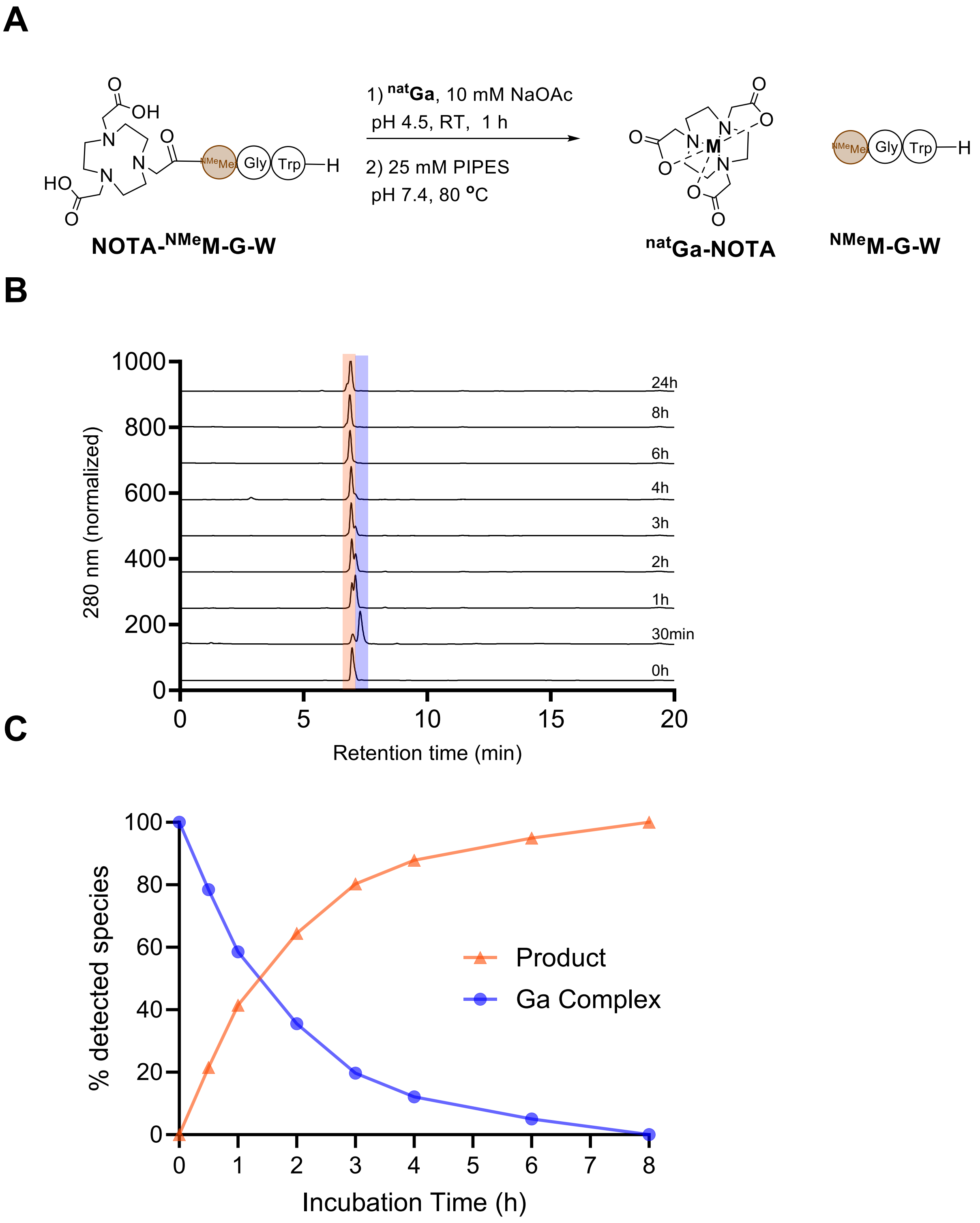

Figure S181. (A) Schematic description of preparation and direct complexation procedure of **[natGa(19)]+**. **(B)** Analytical HPLC chromatograms showing the autolytic release of the natGa-NOTA. **(C)** Quantification of the pH-dependent cleavage at 80 °C and 25 mM pH 7.4 PIPES buffer.

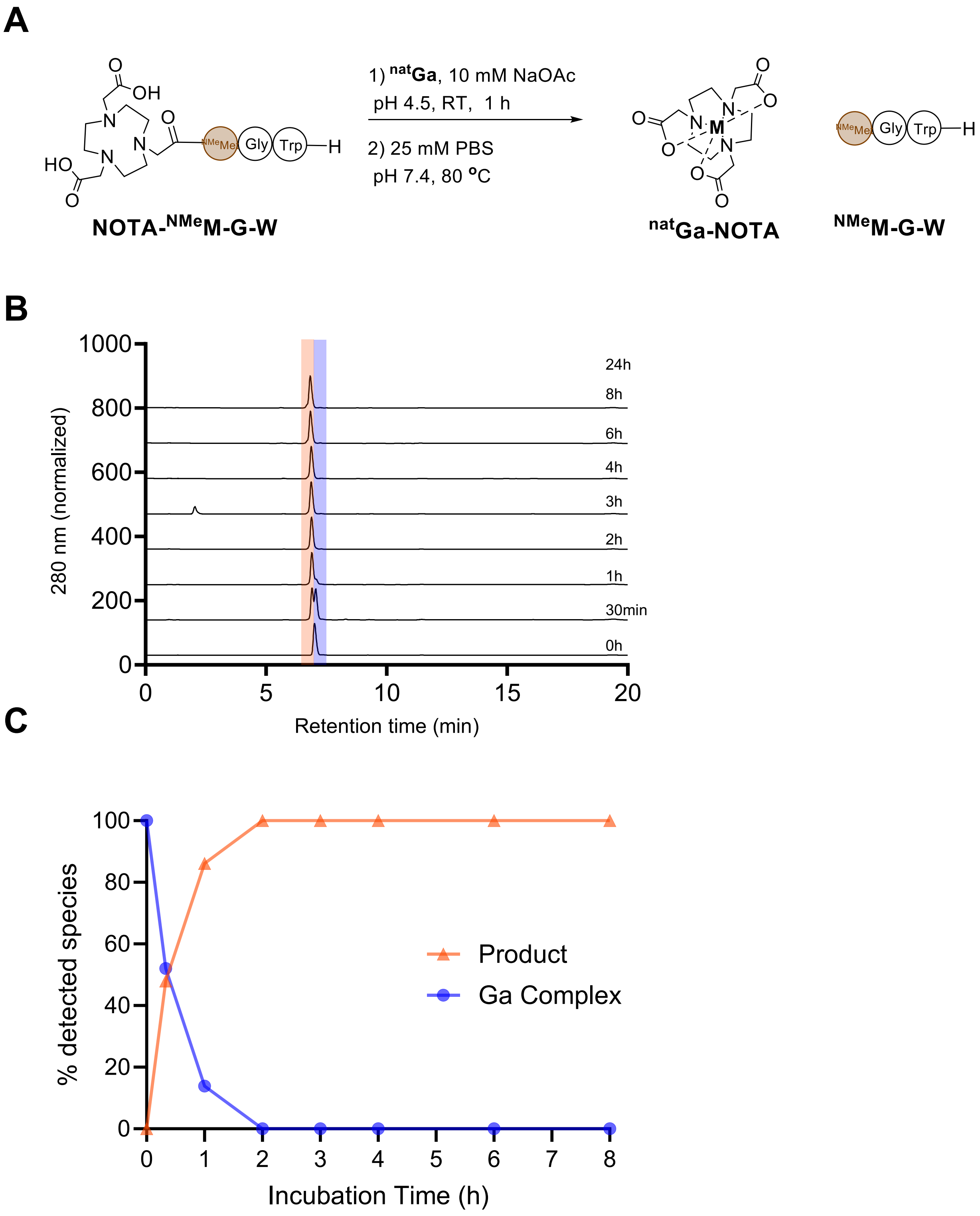

Figure S182. (A) Schematic description of preparation and direct complexation procedure of **[^{nat}Ga(19)]$^+$**. **(B)** Analytical HPLC chromatograms showing the autolytic release of the ^{nat}Ga-NOTA. **(C)** Quantification of the pH-dependent cleavage at 80 °C and 25 mM pH 7.4 PBS buffer.

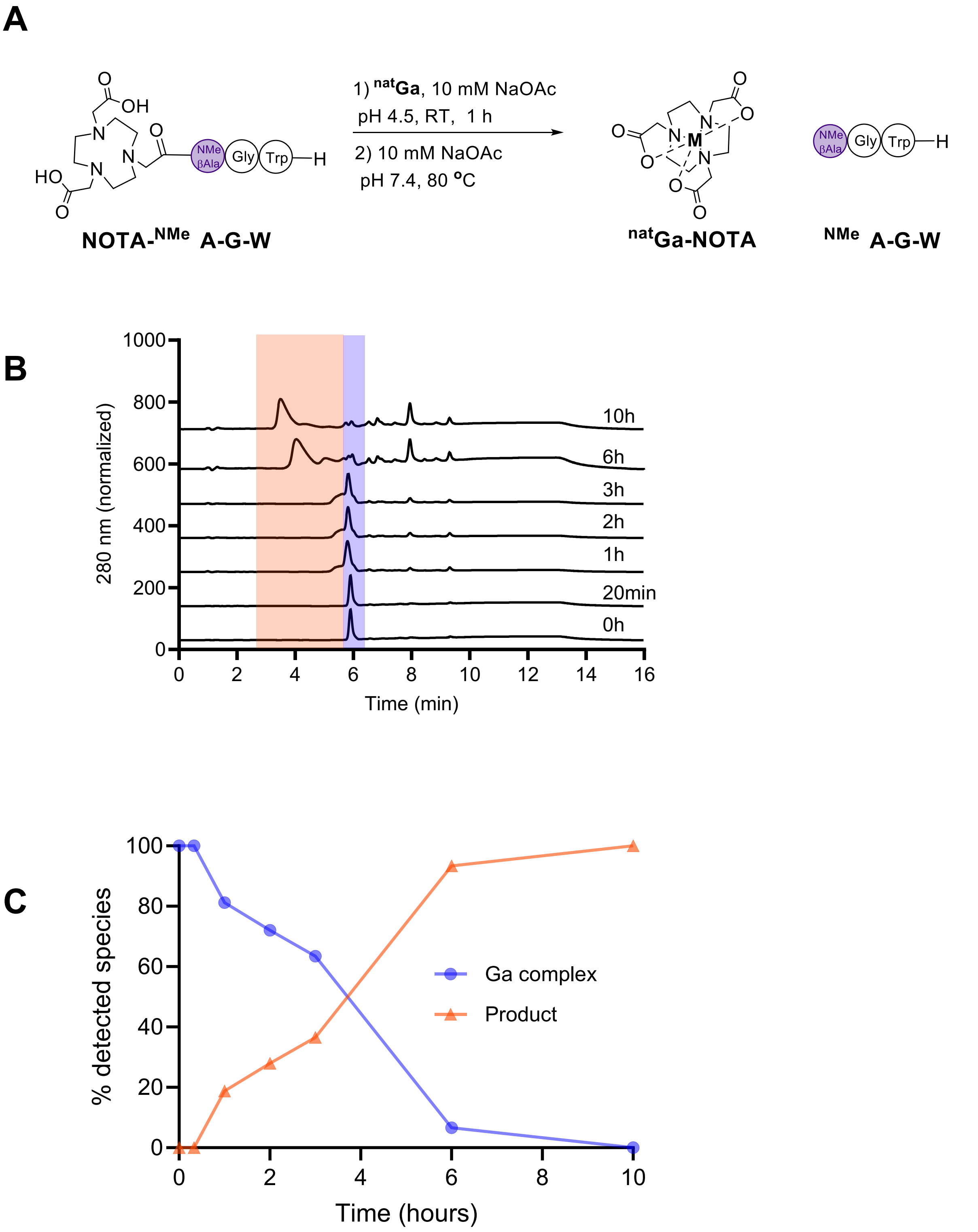

Figure S183. (A) Schematic description of preparation and direct complexation procedure of **$[^{nat}$Ga(20)$]^{+}$**. **(B)** Analytical HPLC chromatograms showing the autolytic release of the ^{nat}Ga-NOTA. **(C)** Quantification of the pH-dependent cleavage at 80 °C and pH 8.5.

Table S1. Relative rate enhancement for Ga-mediated amide bond cleavage at pH 7.4 and 80 ºC

Amino acid	Buffers	k_{obs}, hour^{-1a}	k_{rel}, hour^{-1b}
**Met (15)**	25 mM HEPES	0.038	1
	250 mM HEPES	0.061	1.61
	25 mM PIPES	0.047	1.24
	25 mM PBS	1.09	28.7
**^{NMe}Met (18)**	25 mM HEPES	0.45	1
	250 mM HEPES	1.10	2.44
	25 mM PIPES	0.51	1.13
	25 mM PBS	1.98	4.4

[a]The pseudo first-order reaction rates of Met (15) and ^{NMe}Met (18) Ga complexes cleavage in various buffer conditions at 80 ºC, $r^2 \geq 0.99$. Cleavage reactions of Met (15) and ^{NMe}Met (18) were examined under various buffer conditions. These conjugates were chosen to avoid confounding factors, such as N, O-acyl shift, that we have previously investigated. [b]Relative rate enhancement versus in 25 mM pH 7.4 HEPES buffer.

4 Radiochemical Synthesis and Characterization

4.1 Radiolabeling with ^{68}Ga and Cleavage Assay in HEPES buffer

^{68}Ga-chloride was obtained from the University of Wisconsin Radiopharmaceutical Production Facility, at an average specific activity of 20 mCi/mL. For radio-labeling of model tripeptides and Ahx-KuE peptide conjugates, a 35 μL aliquot containing 0.7 mCi of $^{68}GaCl_3$ was added to the ligand (10 nmol, 265 μL) in 0.067 M of sodium acetate (NaOAc) buffer pH 4.5. Radiolabeling was completed after 15 minutes at room temperature. The pH of the solution was then adjusted to 7.4 by pH 7.4 1 M HEPES buffer. The radiolabeled conjugates were further characterized with radio-HPLC; if the radiochemical yield was <98%, a chromatographic purification of the crude radiolabeling mixture was performed. The cleavage of the radiolabeled conjugates was monitored via radio-HPLC at 80 °C and pH 7.4.

4.2 Radiolabeling with ^{67}Ga and Cleavage Assay in HEPES buffer

^{67}Ga-citrate was purchased from Jubilant Radiopharma at an average specific activity of 140.0 MBq/mL. The ^{67}Ga-citrate solution was first converted to $^{67}GaCl$ using an established solid phase extraction protocol. The resulting converted $^{67}GaCl_3$ fraction with the specific activity of 962 MBq/mL was used for radiolabeling. For radio-labeling of model tripeptides and Ahx-KuE peptide conjugates, a 25 μL aliquot containing 0.45 mCi of $^{67}GaCl_3$ was added to the ligand (10 nmol, 110 μL) in 0.15 M of sodium acetate (NaOAc) buffer pH 4.5. Radiolabeling was completed after 10 minutes at room temperature. The pH of the solution was then adjusted to 7.4 by adding pH 7.4 1 M HEPES buffer (final concentration: 0.25 M). The radiolabeled conjugates were further characterized with radio-HPLC; if the radiochemical yield was <98%, a chromatographic purification of the crude radiolabeling mixture was performed. The cleavage of the radiolabeled conjugates was monitored via radio-HPLC at 37 °C and 80 °C at pH 7.4.

4.3 Radiolabeling with ^{67}Ga and Cleavage Assay in Mouse Plasma

For radio-labeling of model tripeptides and Ahx-KuE peptide conjugates, a 25 μL aliquot containing 0.3 mCi of $^{67}GaCl_3$ was added to the ligand (10 nmol, 110 μL) in 0.15 M of sodium acetate (NaOAc) buffer pH 4.5. Radiolabeling was completed after 10 minutes at room temperature. The radiolabeled complexes were further characterized by radio-HPLC. The pH of the solution was then adjusted to 7.4 by adding pH 7.4 1M HEPES buffer. Into the buffered mixture was added equal volume of mouse plasma (buffered radiolabeled complex : mouse plasma 1:1). The cleavage of the radiolabeled conjugates was monitored via radio-HPLC at 37 °C. At each time point, an aliquot of 20 – 40 μL sample was removed from the tube. Plasma protein was precipitated by adding 20 – 40 μL of cold acetonitrile. After centrifugation at 4000 rpm for 10 minutes, the supernatant was collected and diluted in 40 – 70 μL H_2O for radio-HPLC analysis (Method B).

4.4 Characterization of ^{68}Ga-radiolabeled complexes

^{68}Gallium (R)-2,2'-(7-(2-((2-((2-((1-amino-3-(1H-indol-3-yl)-1-oxopropan-2-yl)amino)-2-oxoethyl)amino)-2-oxoethyl)amino)-2-oxoethyl)-1,4,7-triazonane-1,4-diyl)diacetate, **[^{68}Ga][Ga(NO2A)]$^+$-G-G-W-CONH$_2$, [^{68}Ga][Ga(10)]$^+$**. Compound **[^{68}Ga][Ga(10)]$^+$** was synthesized using the general radiolabeling protocol from compound **10**. The product was characterized using radio-HPLC chromatography. R$_t$ (Method B): 6.63 min. RCY: 97%.

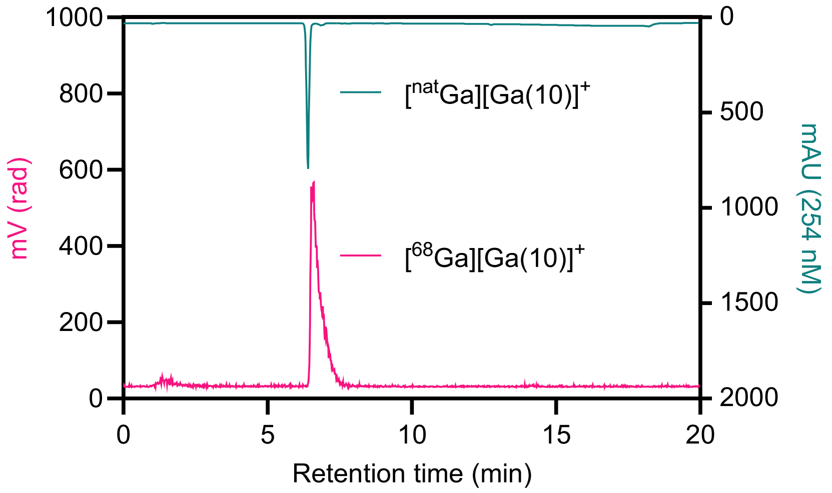

Figure S184. Radio-HPLC traces of **[^{nat}Ga][Ga(10)]$^+$** and **[^{68}Ga][Ga(10)]$^+$**.

^{68}Gallium (S)-2,2'-(7-(2-((3-((2-((1-amino-3-(1H-indol-3-yl)-1-oxopropan-2-yl)amino)-2-oxoethyl)amino)-3-oxopropyl)amino)-2-oxoethyl)-1,4,7-triazonane-1,4-diyl)diacetate, **[^{68}Ga][Ga(NO2A)]$^+$-$^{\beta}$A-G-W-CONH$_2$, [^{68}Ga][Ga(11)]$^+$**. Compound **[^{68}Ga][Ga(11)]$^+$** was synthesized using the general radiolabeling protocol from compound **11**. The product was characterized using radio-HPLC chromatography. R$_t$ (Method B): 6.65 min. RCY: 95%.

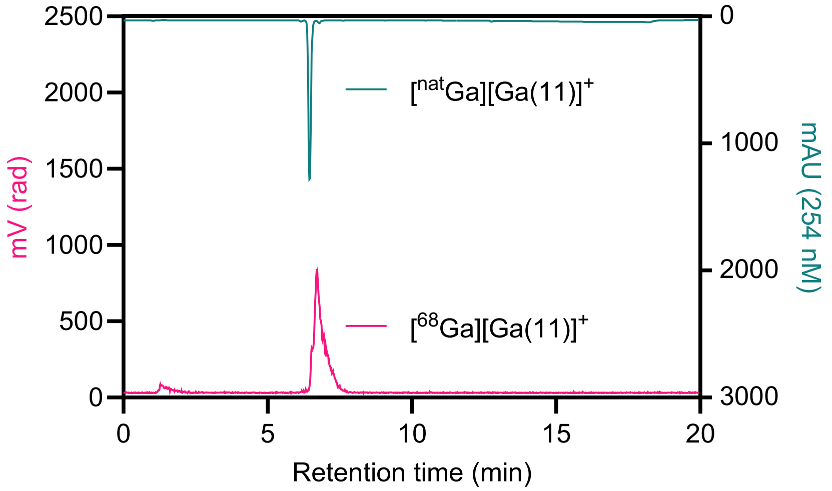

Figure S185. Radio-HPLC traces of **[^{nat}Ga][Ga(11)]$^+$** and **[^{68}Ga][Ga(11)]$^+$**.

^{68}Gallium 2,2'-(7-(2-(((R)-1-((2-(((S)-1-amino-3-(1H-indol-3-yl)-1-oxopropan-2-yl)amino)-2-oxoethyl)amino)-1-oxopropan-2-yl)amino)-2-oxoethyl)-1,4,7-triazonane-1,4-diyl)diacetate, **[^{68}Ga][Ga(NO2A)]$^+$-A-G-W-CONH$_2$**, **[^{68}Ga][Ga(12)]$^+$**. Compound **[^{68}Ga][Ga(12)]$^+$** was synthesized using the general radiolabeling protocol from compound **12**. The product was characterized using radio-HPLC chromatography. Rt (Method B): 6.82 min. RCY: 80%.

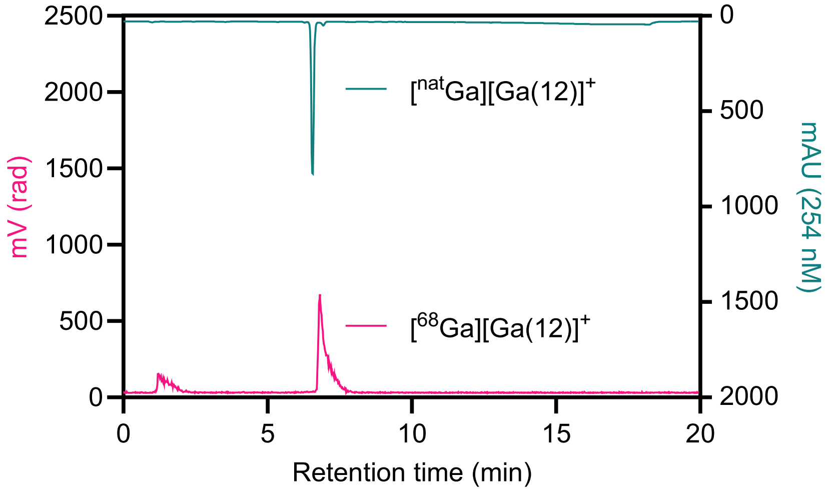

Figure S186. Radio-HPLC traces of **[^{nat}Ga][Ga(12)]$^+$** and **[^{68}Ga][Ga(12)]$^+$**.

^{68}Gallium 2,2'-(7-(2-(((R)-1-((2-(((S)-1-amino-3-(1H-indol-3-yl)-1-oxopropan-2-yl)amino)-2-oxoethyl)amino)-3-hydroxy-1-oxopropan-2-yl)amino)-2-oxoethyl)-1,4,7-triazonane-1,4-diyl)diacetate, **[^{68}Ga][Ga(NO2A)]$^+$-S-G-W-CONH$_2$**, **[^{68}Ga][Ga(13)]$^+$**. Compound **[^{68}Ga][Ga(13)]$^+$** was synthesized using the general radiolabeling protocol from compound **13**. The product was characterized using radio-HPLC chromatography. R_t (Method B): 6.78 min. RCY: 95%.

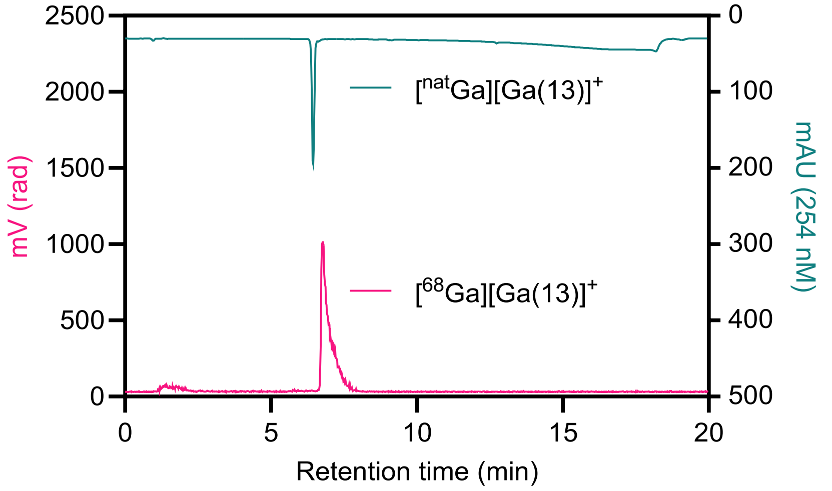

Figure S187. Radio-HPLC traces of [^{nat}Ga][Ga(**13**)]$^+$ and [^{68}Ga][Ga(**13**)]$^+$.

[68]Gallium 2,2'-(7-(2-(((2R,3S)-1-((2-(((S)-1-amino-3-(1H-indol-3-yl)-1-oxopropan-2-yl)amino)-2-oxoethyl)amino)-3-hydroxy-1-oxobutan-2-yl)amino)-2-oxoethyl)-1,4,7-triazonane-1,4-diyl)diacetate, **[^{68}Ga][Ga(NO2A)]$^{+}$-T-G-W-CONH$_2$, [^{68}Ga][Ga(14)]$^{+}$**. Compound **[^{68}Ga][Ga(14)]$^{+}$** was synthesized using the general radiolabeling protocol from compound **14**. The product was characterized using radio-HPLC chromatography. R_t (Method B): 6.50 min. RCY: 96%.

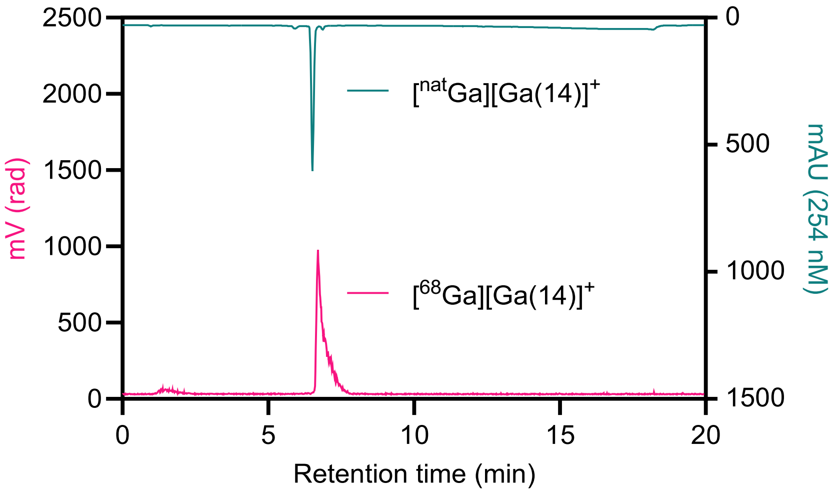

Figure S188. Radio-HPLC traces of **[^{nat}Ga][Ga(14)]$^{+}$** and **[^{68}Ga][Ga(14)]$^{+}$**.

[68]Gallium 2,2'-(7-(2-(((R)-1-((2-(((S)-1-amino-3-(1H-indol-3-yl)-1-oxopropan-2-yl)amino)-2-oxoethyl)amino)-4-(methylthio)-1-oxobutan-2-yl)amino)-2-oxoethyl)-1,4,7-triazonane-1,4-diyl)diacetate, **[^{68}Ga][Ga(NO2A)]$^{+}$-M-G-W-CONH$_2$, [^{68}Ga][Ga(15)]$^{+}$**. Compound **[^{68}Ga][Ga(15)]$^{+}$** was synthesized using the general radiolabeling protocol from compound **15**. The product was characterized using radio-HPLC chromatography. R_t (Method B): 7.55 min. RCY: 99%.

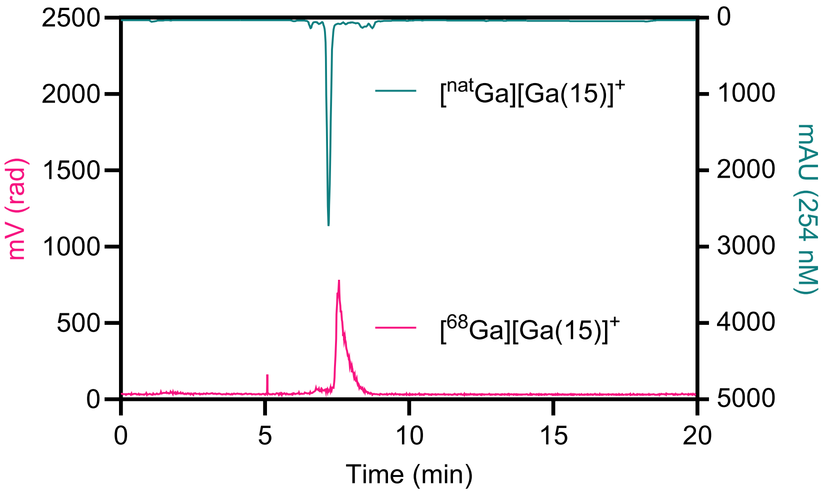

Figure S189. Radio-HPLC traces of **[^{nat}Ga][Ga(15)]$^{+}$** and **[^{68}Ga][Ga(15)]$^{+}$**.

[68]Gallium 2,2'-(7-(2-(((R)-1-((2-(((S)-1-amino-3-(1H-indol-3-yl)-1-oxopropan-2-yl)amino)-2-oxoethyl)amino)-3-hydroxy-1-oxopropan-2-yl)(methyl)amino)-2-oxoethyl)-1,4,7-triazonane-1,4-diyl)diacetate, **[^{68}Ga][Ga(NO2A)]$^{+}$-^{NMe}S-G-W-CONH$_2$**. Compound was synthesized using the general radiolabeling protocol from compound **16**. The product was characterized using radio-HPLC chromatography. R_t (Method B): 6.76 min. RCY: 95%.

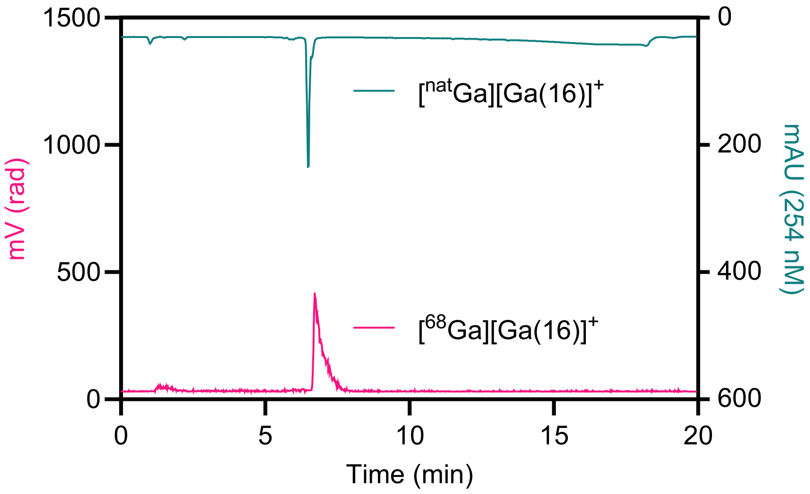

Figure S190. Radio-HPLC traces of **[^{nat}Ga][Ga(16)]$^{+}$** and **[^{68}Ga][Ga(16)]$^{+}$**.

[68]Gallium (S)-2,2'-(7-(2-((2-((2-((1-amino-3-(1H-indol-3-yl)-1-oxopropan-2-yl)amino)-2-oxoethyl)amino)-2-oxoethyl)(methyl)amino)-2-oxoethyl)-1,4,7-triazonane-1,4-diyl)diacetate, **[^{68}Ga][Ga(NO2A)]$^{+}$-^{NMe}G-G-W-CONH$_2$**, **[^{68}Ga][Ga(17)]$^{+}$**. Compound **[^{68}Ga][Ga(17)]$^{+}$** was synthesized using the general radiolabeling protocol from compound **17**. The product was characterized using radio-HPLC chromatography. R_t (Method B): 6.64 min. RCY: 95%.

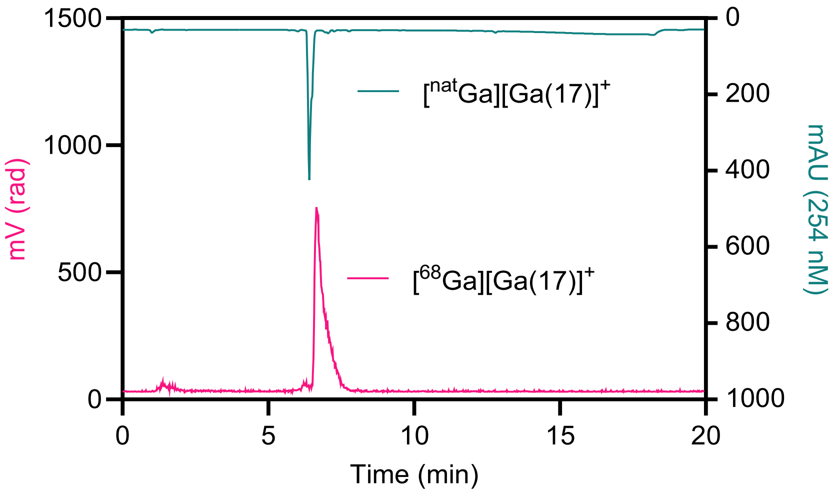

Figure S191. Radio-HPLC traces of **[^{nat}Ga][Ga(17)]$^{+}$** and **[^{68}Ga][Ga(17)]$^{+}$**.

[68]Gallium 2,2'-(7-(2-(((R)-1-((2-(((S)-1-amino-3-(1H-indol-3-yl)-1-oxopropan-2-yl)amino)-2-oxoethyl)amino)-4-(methylthio)-1-oxobutan-2-yl)(methyl)amino)-2-oxoethyl)-1,4,7-triazonane-1,4-diyl)diacetate, **[^{68}Ga][Ga(NO2A)]$^{+}$-^{NMe}M-G-W-CONH$_2$**, **[^{68}Ga][Ga(18)]$^{+}$**. Compound **[^{68}Ga][Ga(18)]$^{+}$** was synthesized using the general radiolabeling protocol from compound **17**. The product was characterized using radio-HPLC chromatography. R_t (Method B): 7.48 min. RCY: 99%.

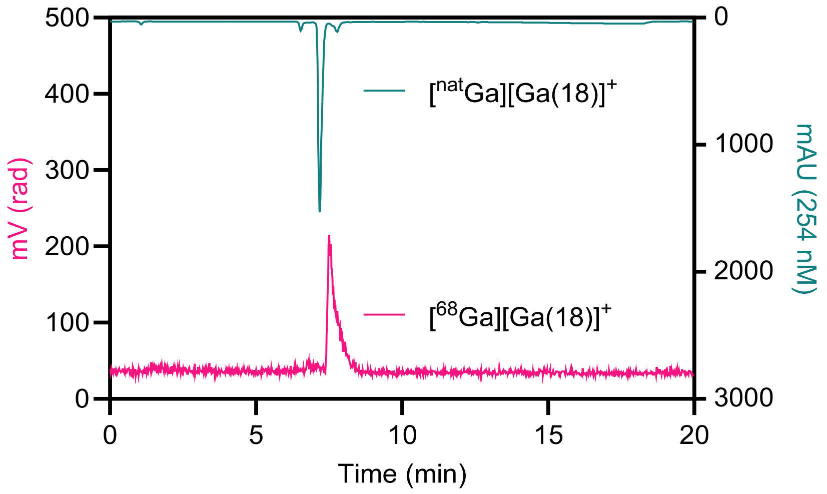

Figure S192. Radio-HPLC traces of **[^{nat}Ga][Ga(18)]$^{+}$** and **[^{68}Ga][Ga(18)]$^{+}$**.

[68]Gallium (7R,21S,25S)-1-(4,7-bis(carboxymethyl)-1,4,7-triazonan-1-yl)-7-(4-hydroxy-3-iodobenzyl)-2,5,8,15,23-pentaoxo-3,6,9,16,22,24-hexaazaheptacosane-21,25,27-tricarboxylic acid, **[^{68}Ga][Ga(NO2A)]$^{+}$-G-Tyr(3-I)-Ahx-KuE**, **[^{68}Ga][Ga(20)]$^{+}$**. Compound **[^{68}Ga][Ga(20)]$^{+}$** was synthesized using the general radiolabeling protocol from compound **20**. The product was characterized using radio-HPLC chromatography. R_t (Method B): 7.23 min. RCY: 99%.

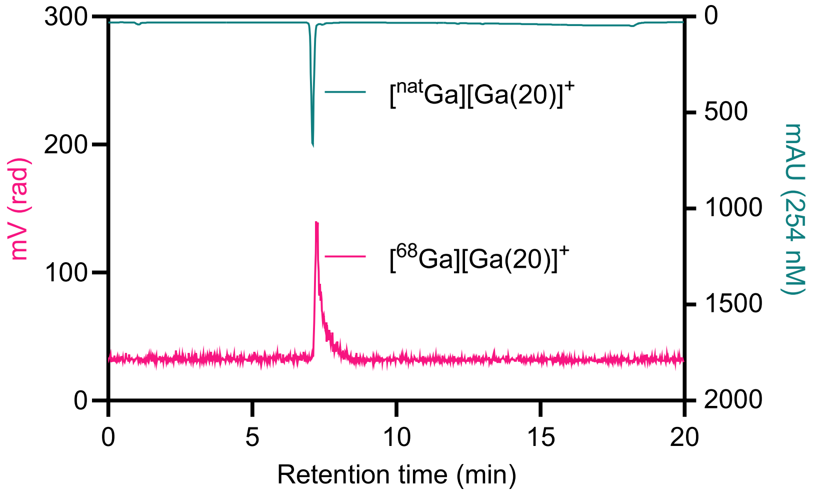

Figure S193. Radio-HPLC traces of **[^{nat}Ga][Ga(20)]$^{+}$** and **[^{68}Ga][Ga(20)]$^{+}$**.

^{68}*Gallium (4S,7R,21S,25S)-1-(4,7-bis(carboxymethyl)-1,4,7-triazonan-1-yl)-7-(4-hydroxy-3-iodobenzyl)-4-(hydroxymethyl)-2,5,8,15,23-pentaoxo-3,6,9,16,22,24-hexaazaheptacosane-21,25,27-tricarboxylic acid*, **[^{68}Ga][Ga(NO2A)]$^{+}$-S-Tyr(3-I)-Ahx-KuE**, **[^{68}Ga][Ga(21)]**$^{+}$. Compound **[^{68}Ga][Ga(21)]**$^{+}$ was synthesized using the general radiolabeling protocol from compound **21**. The product was characterized using radio-HPLC chromatography. R_t (Method B): 7.08 min. RCY: 95%.

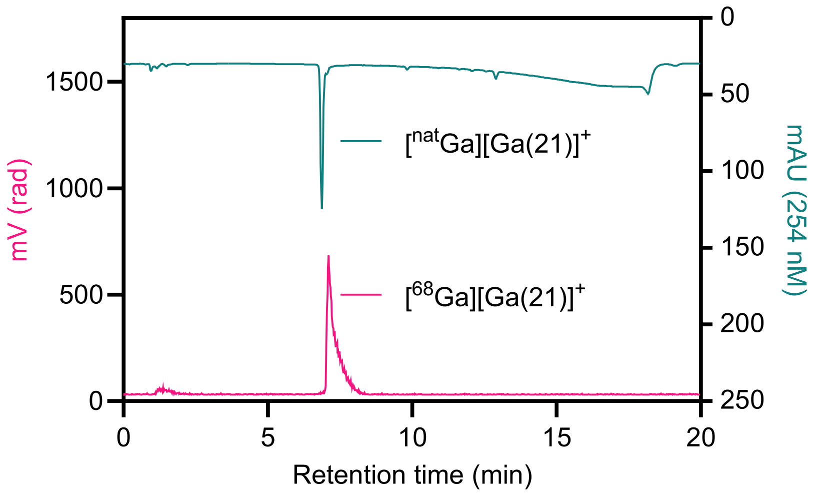

Figure S194. Radio-HPLC traces of **[^{nat}Ga][Ga(21)]**$^{+}$ and **[^{68}Ga][Ga(21)]**$^{+}$.

^{68}*Gallium (5S,8R,22S,26S)-5-(2-(4,7-bis(carboxymethyl)-1,4,7-triazonan-1-yl)acetamido)-8-(4-hydroxy-3-iodobenzyl)-6,9,16,24-tetraoxo-2-thia-7,10,17,23,25-pentaazaoctacosane-22,26,28-tricarboxylic acid*, **[^{68}Ga][Ga(NO2A)]$^{+}$-M-Tyr(3-I)-Ahx-KuE**, **[^{68}Ga][Ga(22)]**$^{+}$. Compound **[^{68}Ga][Ga(22)]**$^{+}$ was synthesized using the general radiolabeling protocol from compound **22**. The product was characterized using radio-HPLC chromatography. R_t (Method B): 7.78 min. RCY: 99%.

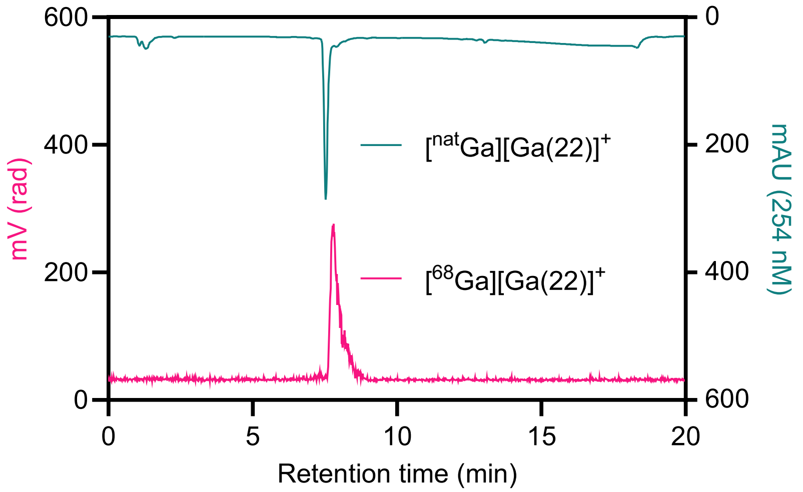

Figure S195. Radio-HPLC traces of **[^{nat}Ga][Ga(22)]**$^{+}$ and **[^{68}Ga][Ga(22)]**$^{+}$.

[68]Gallium (7R,21S,25S)-1-(4,7-bis(carboxymethyl)-1,4,7-triazonan-1-yl)-7-(4-hydroxy-3-iodobenzyl)-3-methyl-2,5,8,15,23-pentaoxo-3,6,9,16,22,24-hexaazaheptacosane-21,25,27-tricarboxylic acid, **[^{68}Ga][Ga(NO2A)]$^{+}$-^{NMe}G-Tyr(3-I)-Ahx-KuE**, **[^{68}Ga][Ga(23)]$^{+}$**. Compound **[^{68}Ga][Ga(23)]$^{+}$** was synthesized using the general radiolabeling protocol from compound **23**. The product was characterized using radio-HPLC chromatography. R_t (Method B): 7.29 min. RCY: 96%.

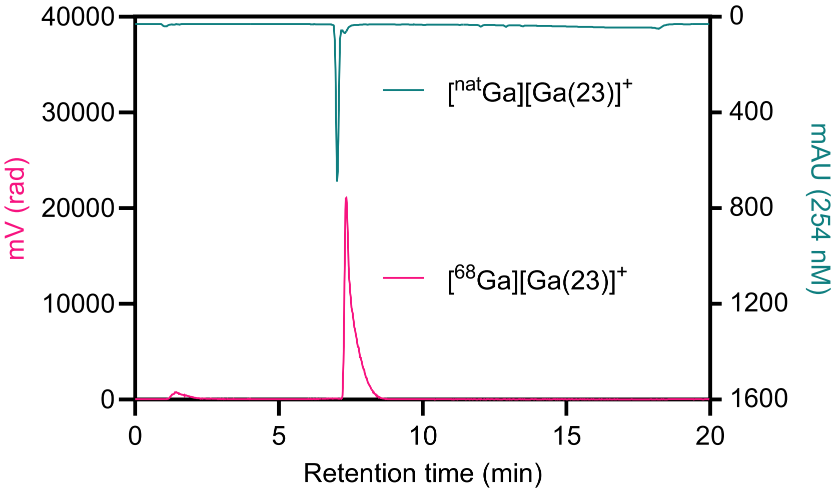

Figure S196. Radio-HPLC traces of **[^{nat}Ga][Ga(23)]$^{+}$** and **[^{68}Ga][Ga(23)]$^{+}$**.

[68]Gallium (4S,7R,21S,25S)-1-(4,7-bis(carboxymethyl)-1,4,7-triazonan-1-yl)-7-(4-hydroxy-3-iodobenzyl)-4-(hydroxymethyl)-3-methyl-2,5,8,15,23-pentaoxo-3,6,9,16,22,24-hexaazaheptacosane-21,25,27-tricarboxylic acid, **[^{68}Ga][Ga(NO2A)]$^{+}$-^{NMe}S-Tyr(3-I)-Ahx-KuE, [^{68}Ga][Ga(24)]$^{+}$**. Compound **[^{68}Ga][Ga(24)]$^{+}$** was synthesized using the general radiolabeling protocol from compound **24**. The product was characterized using radio-HPLC chromatography. R_t (Method B): 7.14 min. RCY: 95%.

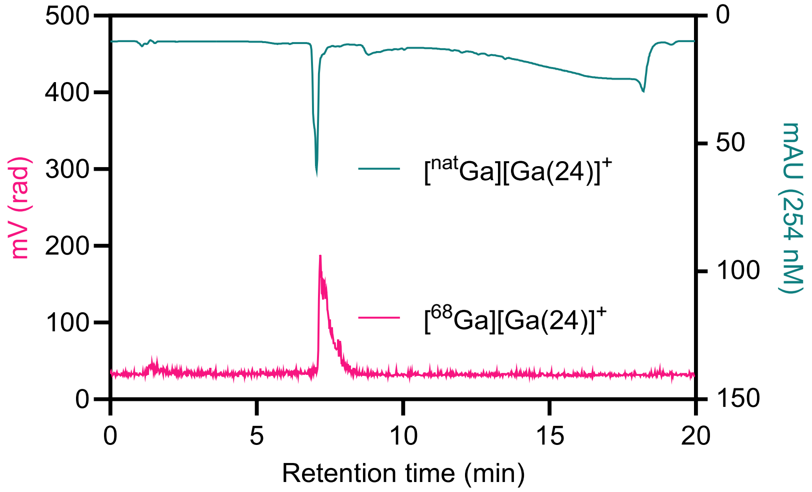

Figure S197. Radio-HPLC traces of **[^{nat}Ga][Ga(24)]$^{+}$** and **[^{68}Ga][Ga(24)]$^{+}$**.

^{68}Gallium (5S,8R,22S,26S)-5-(2-(4,7-bis(carboxymethyl)-1,4,7-triazonan-1-yl)-N-methylacetamido)-8-(4-hydroxy-3-iodobenzyl)-6,9,16,24-tetraoxo-2-thia-7,10,17,23,25-pentaazaoctacosane-22,26,28-tricarboxylic acid, **[^{68}Ga][Ga(NO2A)]$^{+}$-^{NMe}M-Tyr(3-I)-Ahx-KuE, [^{68}Ga][Ga(25)]$^{+}$**. Compound **[^{68}Ga][Ga(25)]$^{+}$** was synthesized using the general radiolabeling protocol from compound **25**. The product was characterized using radio-HPLC chromatography. R_t (Method B): 7.63 min. RCY:99%.

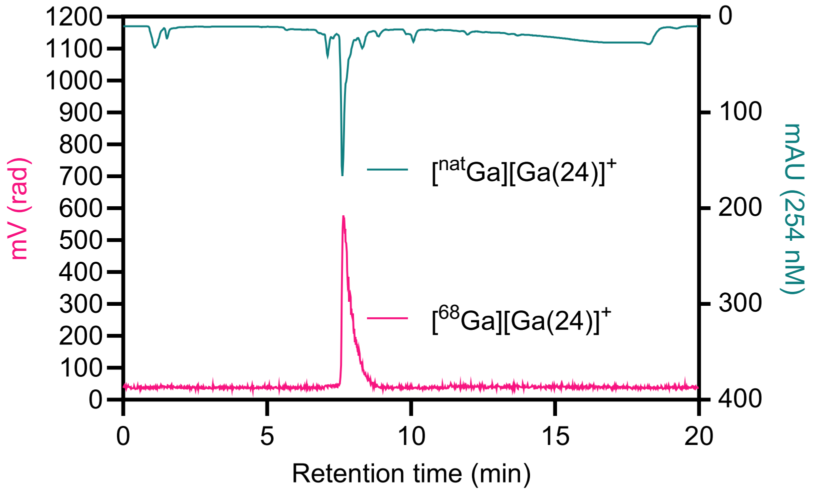

Figure S198. Radio-HPLC traces of **[^{nat}Ga][Ga(25)]$^{+}$** and **[^{68}Ga][Ga(25)]$^{+}$**.

^{67}Gallium (R)-2,2'-(7-(2-((2-((2-((1-amino-3-(1H-indol-3-yl)-1-oxopropan-2-yl)amino)-2-oxoethyl)amino)-2-oxoethyl)amino)-2-oxoethyl)-1,4,7-triazonane-1,4-diyl)diacetate, **[^{67}Ga][Ga(NO2A)]$^{+}$-G-G-W-CONH$_2$, [^{67}Ga][Ga(10)]$^{+}$**. Compound **[^{67}Ga][Ga(10)]$^{+}$** was synthesized using the general radiolabeling protocol from compound **10**. The product was characterized using radio-HPLC chromatography. R_t (Method B): 6.87 min. RCY: 99%.

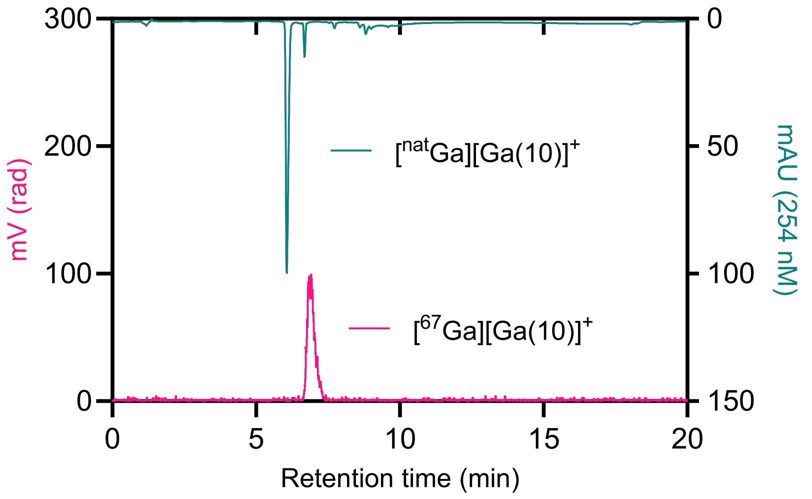

Figure S199. Radio-HPLC traces of **[^{nat}Ga][Ga(10)]$^{+}$** and **[^{67}Ga][Ga(10)]$^{+}$**.

[67]Gallium 2,2'-(7-(2-(((R)-1-((2-(((S)-1-amino-3-(1H-indol-3-yl)-1-oxopropan-2-yl)amino)-2-oxoethyl)amino)-3-hydroxy-1-oxopropan-2-yl)amino)-2-oxoethyl)-1,4,7-triazonane-1,4-diyl)diacetate, **[^{67}Ga][Ga(NO2A)]$^+$-S-G-W-CONH$_2$**, **[^{67}Ga][Ga(13)]$^+$**. Compound **[^{67}Ga][Ga(13)]$^+$** was synthesized using the general radiolabeling protocol from compound **13**. The product was characterized using radio-HPLC chromatography. R_t (Method B): 7.52 min. RCY: 99%.

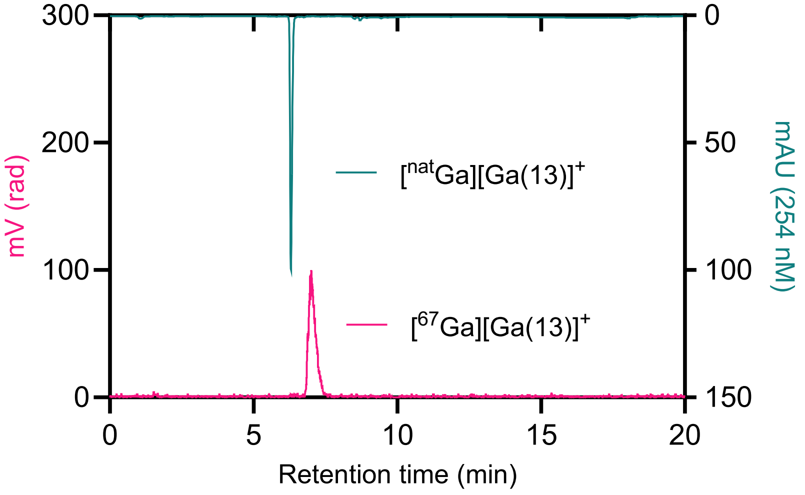

Figure S200. Radio-HPLC traces of **[^{nat}Ga][Ga(13)]$^+$** and **[^{67}Ga][Ga(13)]$^+$**.

[67]Gallium 2,2'-(7-(2-(((R)-1-((2-(((S)-1-amino-3-(1H-indol-3-yl)-1-oxopropan-2-yl)amino)-2-oxoethyl)amino)-4-(methylthio)-1-oxobutan-2-yl)amino)-2-oxoethyl)-1,4,7-triazonane-1,4-diyl)diacetate, **[^{67}Ga][Ga(NO2A)]$^+$-M-G-W-CONH$_2$**, **[^{67}Ga][Ga(15)]$^+$**. Compound **[^{67}Ga][Ga(15)]$^+$** was synthesized using the general radiolabeling protocol from compound **15**. The product was characterized using radio-HPLC chromatography. R_t (Method B): 7.74 min. RCY: 99%.

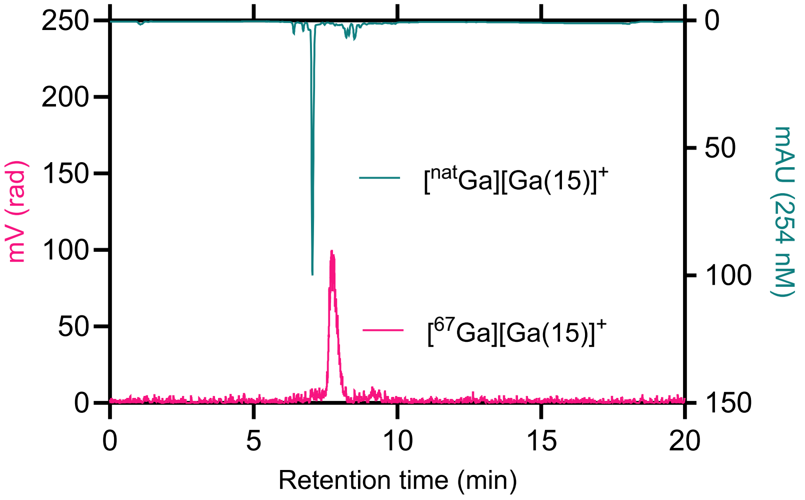

Figure S201. Radio-HPLC traces of **[^{nat}Ga][Ga(15)]$^+$** and **[^{67}Ga][Ga(15)]$^+$**.

[67]Gallium 2,2'-(7-(2-(((R)-1-((2-(((S)-1-amino-3-(1H-indol-3-yl)-1-oxopropan-2-yl)amino)-2-oxoethyl)amino)-3-hydroxy-1-oxopropan-2-yl)(methyl)amino)-2-oxoethyl)-1,4,7-triazonane-1,4-diyl)diacetate, **[^{67}Ga][Ga(NO2A)]$^{+}$-^{NMe}S-G-W-CONH$_2$**. Compound was synthesized using the general radiolabeling protocol from compound **16**. The product was characterized using radio-HPLC chromatography. R_t (Method B): 7.06 min. RCY: 98%.

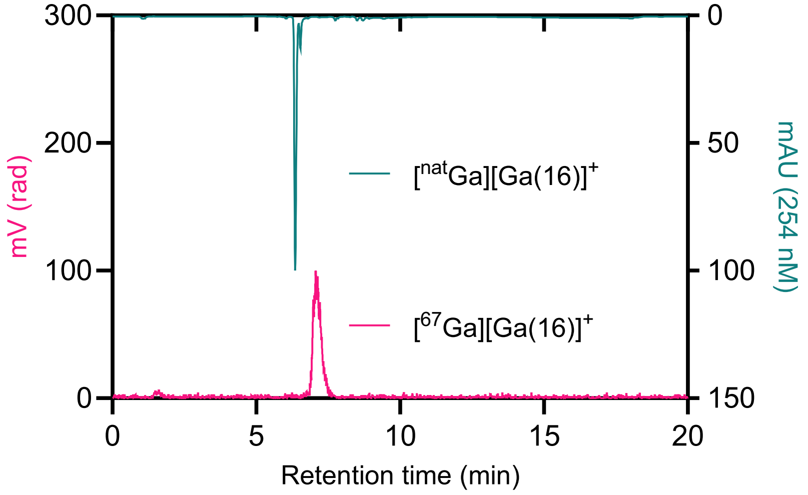

Figure S202. Radio-HPLC traces of **[^{nat}Ga][Ga(16)]$^{+}$** and **[^{67}Ga][Ga(16)]$^{+}$**.

[67]Gallium (S)-2,2'-(7-(2-((2-((2-((1-amino-3-(1H-indol-3-yl)-1-oxopropan-2-yl)amino)-2-oxoethyl)amino)-2-oxoethyl)(methyl)amino)-2-oxoethyl)-1,4,7-triazonane-1,4-diyl)diacetate, **[^{67}Ga][Ga(NO2A)]$^{+}$-^{NMe}G-G-W-CONH$_2$**, **[^{67}Ga][Ga(17)]$^{+}$**. Compound **[^{67}Ga][Ga(17)]$^{+}$** was synthesized using the general radiolabeling protocol from compound **17**. The product was characterized using radio-HPLC chromatography. R_t (Method B): 6.94 min. RCY: 99%.

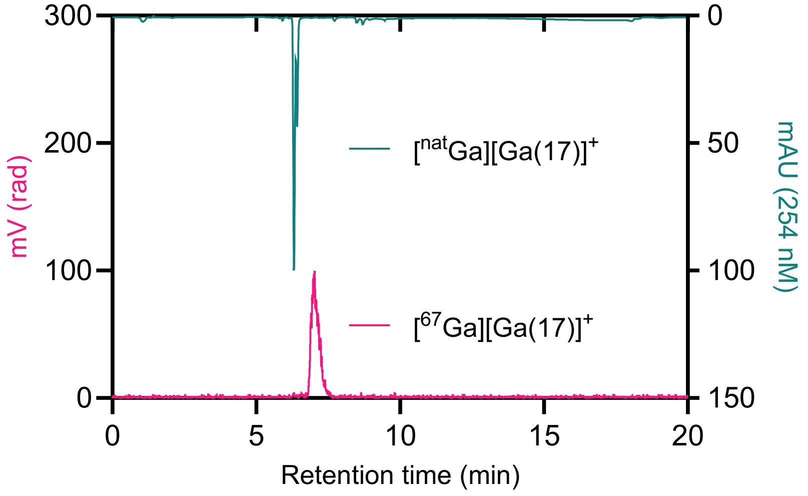

Figure S203. Radio-HPLC traces of **[^{nat}Ga][Ga(17)]$^{+}$** and **[^{67}Ga][Ga(17)]$^{+}$**.

[67]Gallium 2,2'-(7-(2-(((R)-1-((2-(((S)-1-amino-3-(1H-indol-3-yl)-1-oxopropan-2-yl)amino)-2-oxoethyl)amino)-4-(methylthio)-1-oxobutan-2-yl)(methyl)amino)-2-oxoethyl)-1,4,7-triazonane-1,4-diyl)diacetate, **[^{67}Ga][Ga(NO2A)]$^{+}$-^{NMe}M-G-W-CONH$_2$**, **[^{67}Ga][Ga(18)]$^{+}$**. Compound **[^{67}Ga][Ga(18)]$^{+}$** was synthesized using the general radiolabeling protocol from compound **17**. The product was characterized using radio-HPLC chromatography. R_t (Method B): 7.48 min. RCY: 99%.

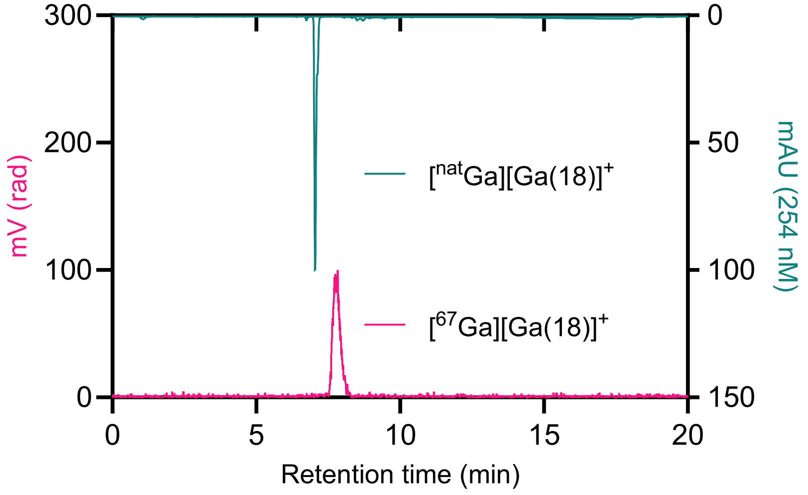

Figure S204. Radio-HPLC traces of **[^{nat}Ga][Ga(18)]$^{+}$** and **[^{67}Ga][Ga(18)]$^{+}$**.

[67]Gallium (7R,21S,25S)-1-(4,7-bis(carboxymethyl)-1,4,7-triazonan-1-yl)-7-(4-hydroxy-3-iodobenzyl)-2,5,8,15,23-pentaoxo-3,6,9,16,22,24-hexaazaheptacosane-21,25,27-tricarboxylic acid, **[^{67}Ga][Ga(NO2A)]$^{+}$-G-Tyr(3-I)-Ahx-KuE**, **[^{67}Ga][Ga(20)]$^{+}$**. Compound **[^{67}Ga][Ga(20)]$^{+}$** was synthesized using the general radiolabeling protocol from compound **20**. The product was characterized using radio-HPLC chromatography. R_t (Method B): 7.52 min. RCY: 99%.

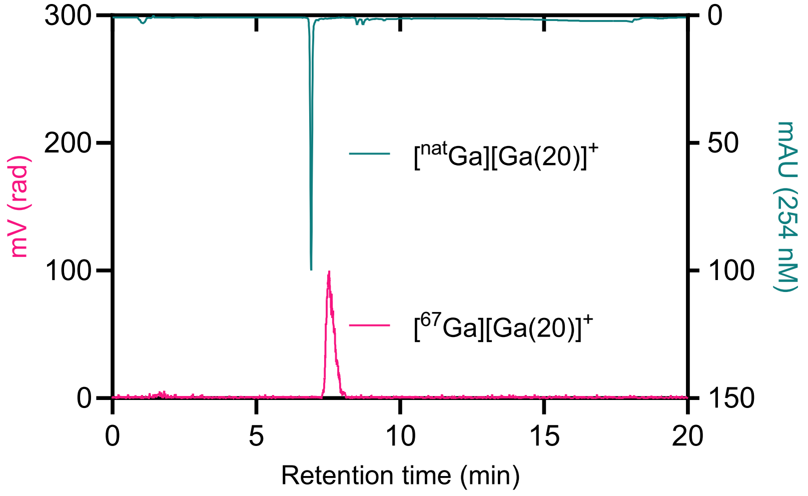

Figure S205. Radio-HPLC traces of **[^{nat}Ga][Ga(20)]$^{+}$** and **[^{67}Ga][Ga(20)]$^{+}$**.

^{67}Gallium (4S,7R,21S,25S)-1-(4,7-bis(carboxymethyl)-1,4,7-triazonan-1-yl)-7-(4-hydroxy-3-iodobenzyl)-4-(hydroxymethyl)-2,5,8,15,23-pentaoxo-3,6,9,16,22,24-hexaazaheptacosane-21,25,27-tricarboxylic acid, **[^{67}Ga][Ga(NO2A)]$^{+}$-S-Tyr(3-I)-Ahx-KuE**, **[^{67}Ga][Ga(21)]$^{+}$**. Compound **[^{67}Ga][Ga(21)]$^{+}$** was synthesized using the general radiolabeling protocol from compound **21**. The product was characterized using radio-HPLC chromatography. R_t (Method B): 7.08 min. RCY: 99%.

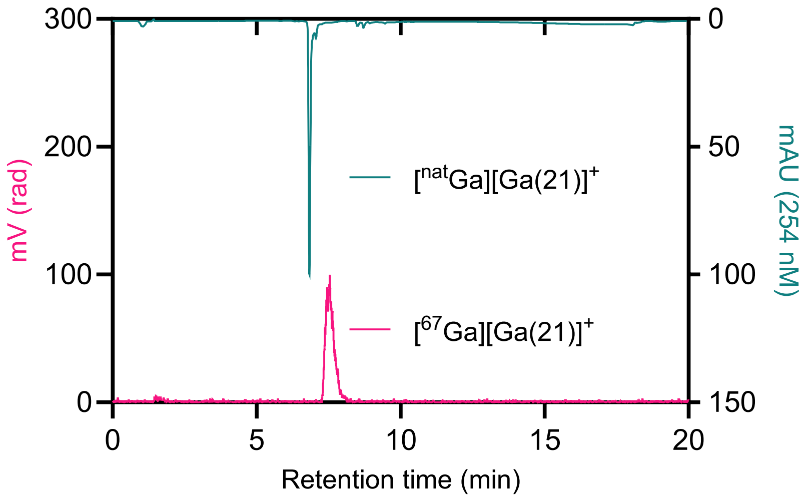

Figure S206. Radio-HPLC traces of **[^{nat}Ga][Ga(21)]$^{+}$** and **[^{67}Ga][Ga(21)]$^{+}$**.

^{67}Gallium (5S,8R,22S,26S)-5-(2-(4,7-bis(carboxymethyl)-1,4,7-triazonan-1-yl)acetamido)-8-(4-hydroxy-3-iodobenzyl)-6,9,16,24-tetraoxo-2-thia-7,10,17,23,25-pentaazaoctacosane-22,26,28-tricarboxylic acid, **[^{67}Ga][Ga(NO2A)]$^{+}$-M-Tyr(3-I)-Ahx-KuE**, **[^{67}Ga][Ga(22)]$^{+}$**. Compound **[^{67}Ga][Ga(22)]$^{+}$** was synthesized using the general radiolabeling protocol from compound **22**. The product was characterized using radio-HPLC chromatography. R_t (Method B): 7.87 min. RCY: 98%.

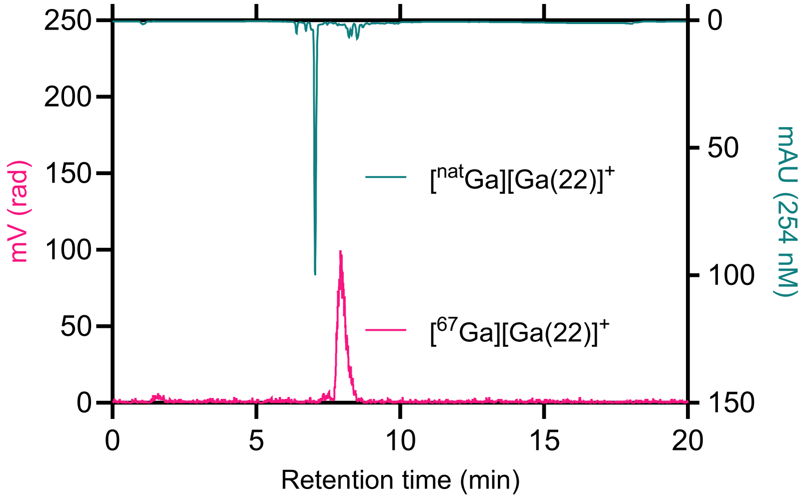

Figure S207. Radio-HPLC traces of **[^{nat}Ga][Ga(22)]$^{+}$** and **[^{67}Ga][Ga(22)]$^{+}$**.

^{67}*Gallium (7R,21S,25S)-1-(4,7-bis(carboxymethyl)-1,4,7-triazonan-1-yl)-7-(4-hydroxy-3-iodobenzyl)-3-methyl-2,5,8,15,23-pentaoxo-3,6,9,16,22,24-hexaazaheptacosane-21,25,27-tricarboxylic acid*, **[^{67}Ga][Ga(NO2A)]$^{+}$-^{NMe}G-Tyr(3-I)-Ahx-KuE**, **[^{67}Ga][Ga(23)]$^{+}$**. Compound **[^{67}Ga][Ga(23)]$^{+}$** was synthesized using the general radiolabeling protocol from compound **23**. The product was characterized using radio-HPLC chromatography. R_t (Method B): 7.57 min. RCY: 98%.

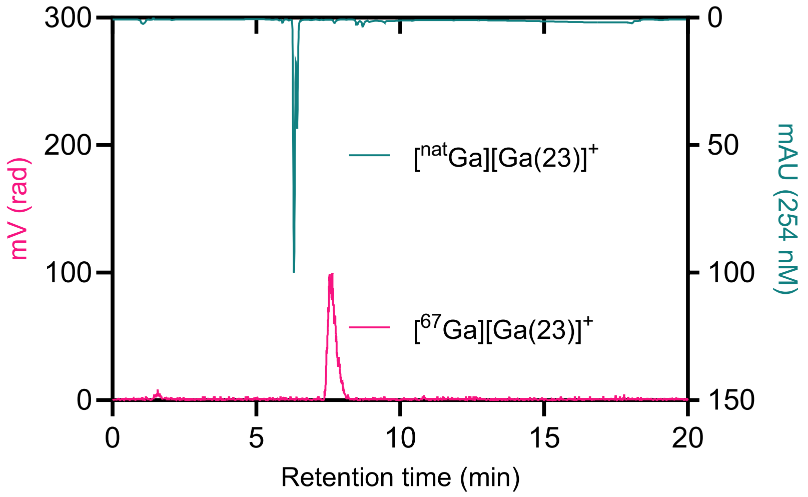

Figure S208. Radio-HPLC traces of **[^{nat}Ga][Ga(23)]$^{+}$** and **[^{67}Ga][Ga(23)]$^{+}$**.

^{67}*Gallium (4S,7R,21S,25S)-1-(4,7-bis(carboxymethyl)-1,4,7-triazonan-1-yl)-7-(4-hydroxy-3-iodobenzyl)-4-(hydroxymethyl)-3-methyl-2,5,8,15,23-pentaoxo-3,6,9,16,22,24-hexaazaheptacosane-21,25,27-tricarboxylic acid*, **[^{67}Ga][Ga(NO2A)]$^{+}$-^{NMe}S-Tyr(3-I)-Ahx-KuE, [^{67}Ga][Ga(24)]$^{+}$**. Compound **[^{67}Ga][Ga(24)]$^{+}$** was synthesized using the general radiolabeling protocol from compound **24**. The product was characterized using radio-HPLC chromatography. R_t (Method B): 7.52 min. RCY: 95%.

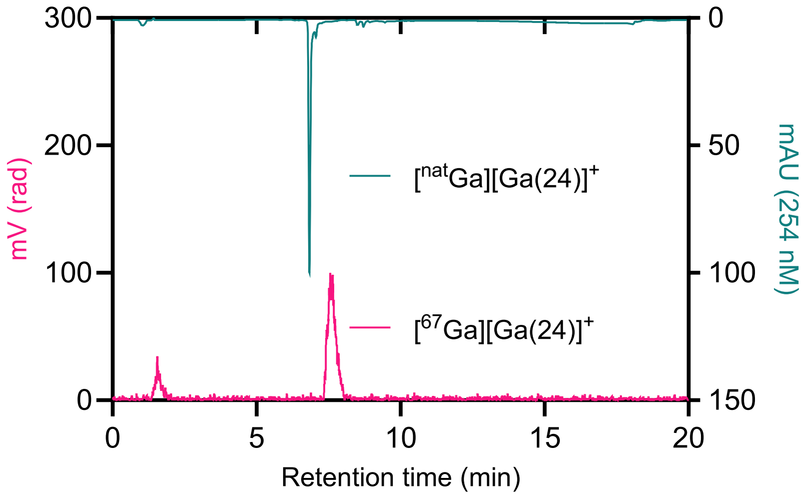

Figure S209. Radio-HPLC traces of **[^{nat}Ga][Ga(24)]$^{+}$** and **[^{67}Ga][Ga(24)]$^{+}$**.

^{67}*Gallium (5S,8R,22S,26S)-5-(2-(4,7-bis(carboxymethyl)-1,4,7-triazonan-1-yl)-N-methylacetamido)-8-(4-hydroxy-3-iodobenzyl)-6,9,16,24-tetraoxo-2-thia-7,10,17,23,25-pentaazaoctacosane-22,26,28-tricarboxylic acid*, **[^{67}Ga][Ga(NO2A)]$^{+}$-^{NMe}M-Tyr(3-I)-Ahx-KuE, [^{67}Ga][Ga(25)]$^{+}$**. Compound **[^{67}Ga][Ga(25)]$^{+}$** was synthesized using the general radiolabeling protocol from compound **25**. The product was characterized using radio-HPLC chromatography. R_t (Method B): 7.98 min. RCY:99%.

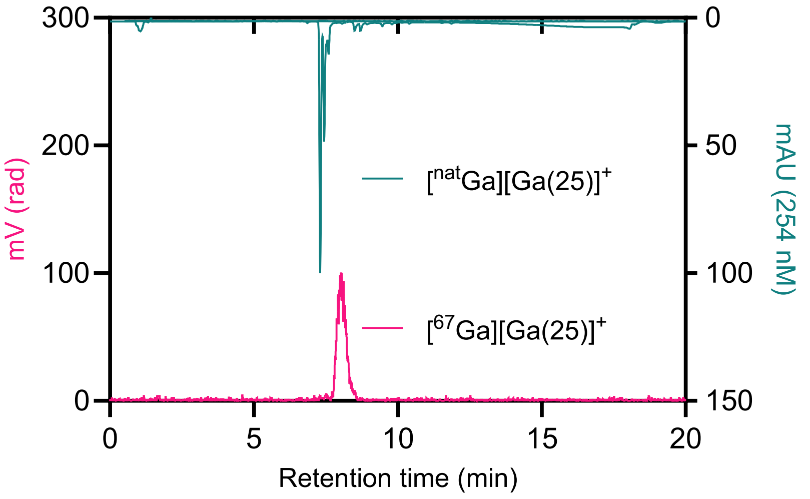

Figure S210. Radio-HPLC traces of **[^{nat}Ga][Ga(25)]$^{+}$** and **[^{67}Ga][Ga(25)]$^{+}$**.

4.5 Cleavage Assay of ^{68}Ga-Radiolabeled Complexes.

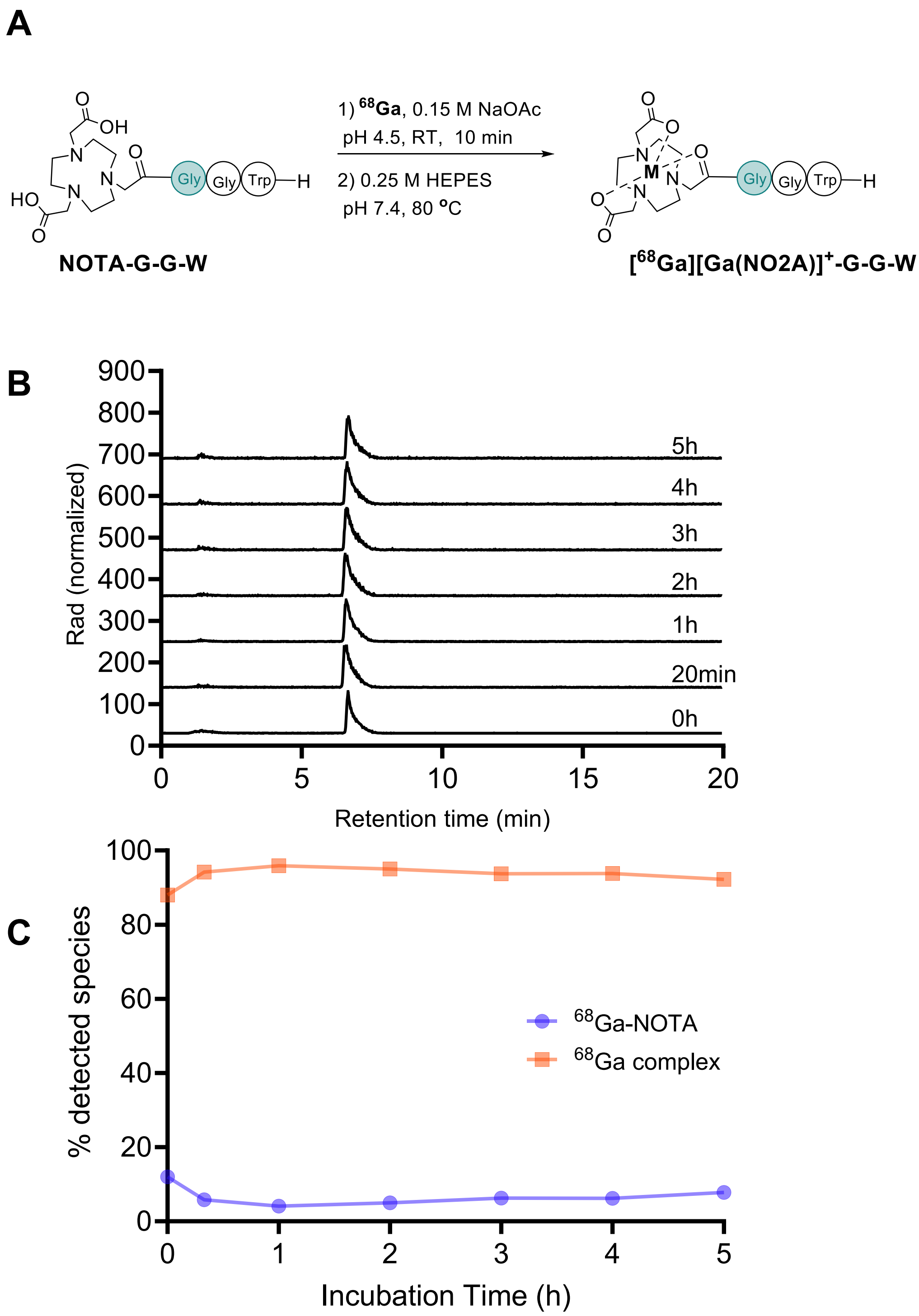

Figure S211. (A) Schematic description of preparation and direct radiolabeling procedure of Gly-containing radiopharmaceutical. **(B)** Analytical radioHPLC chromatograms showing no significant autolytic release of the desired product [^{68}Ga]Ga(NOTA). **(C)** Quantification of the temperature-dependent release of radiopharmaceutical from [^{68}Ga][Ga(NO2A)]$^{+}$-G-G-W-$CONH_2$ at 80 °C and pH 7.4.

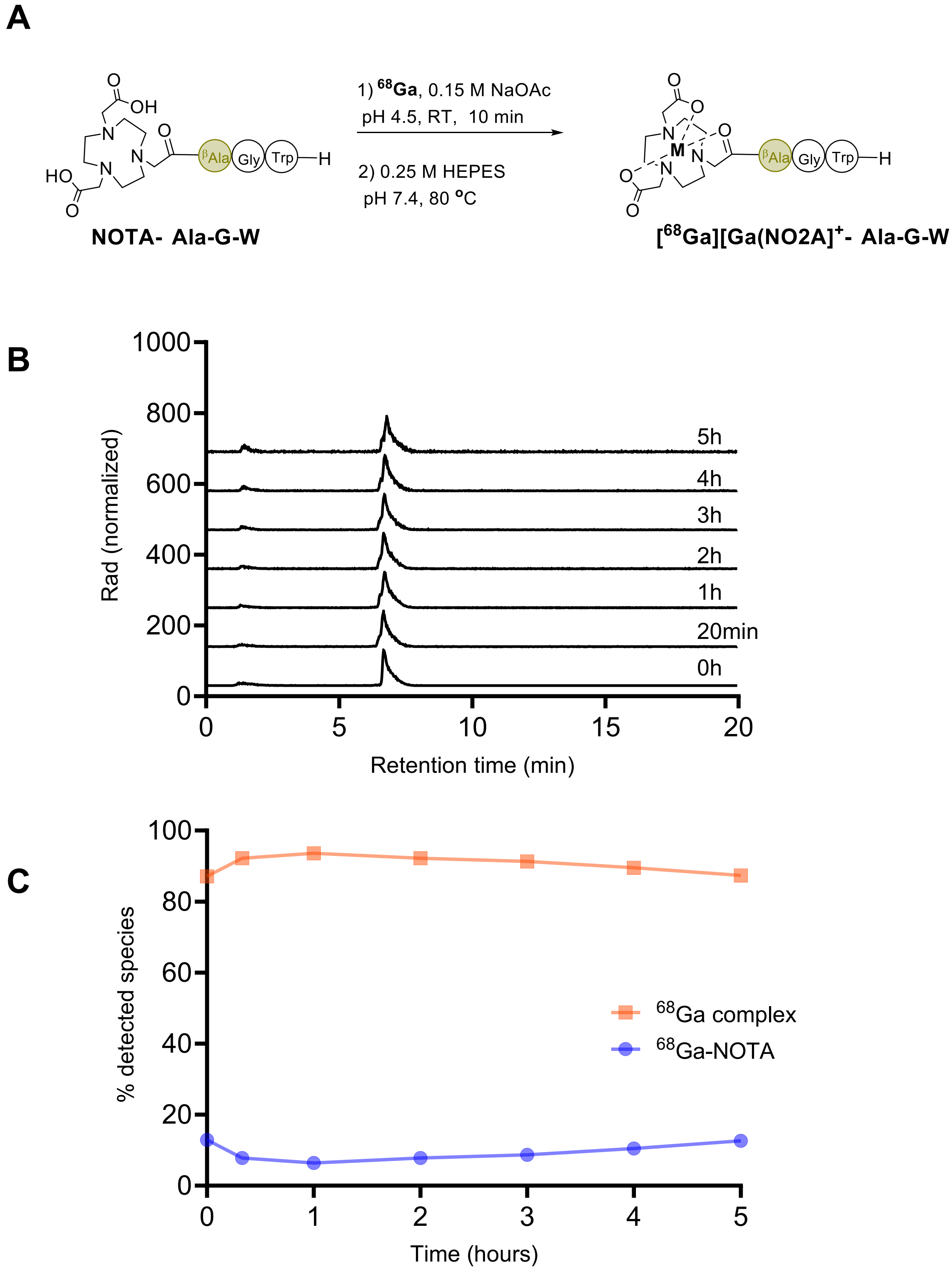

Figure S212. (A) Schematic description of preparation and direct radiolabeling procedure of beta-Ala-containing radiopharmaceutical. **(B)** Analytical radioHPLC chromatograms showing no autolytic release of the desired product [^{68}Ga]Ga(NOTA). **(C)** Quantification of the temperature-dependent release of radiopharmaceutical from [^{68}Ga][Ga(NO2A)]$^{+}$-$^{\beta}$A-G-W-CONH$_2$ at 80 °C and pH 7.4.

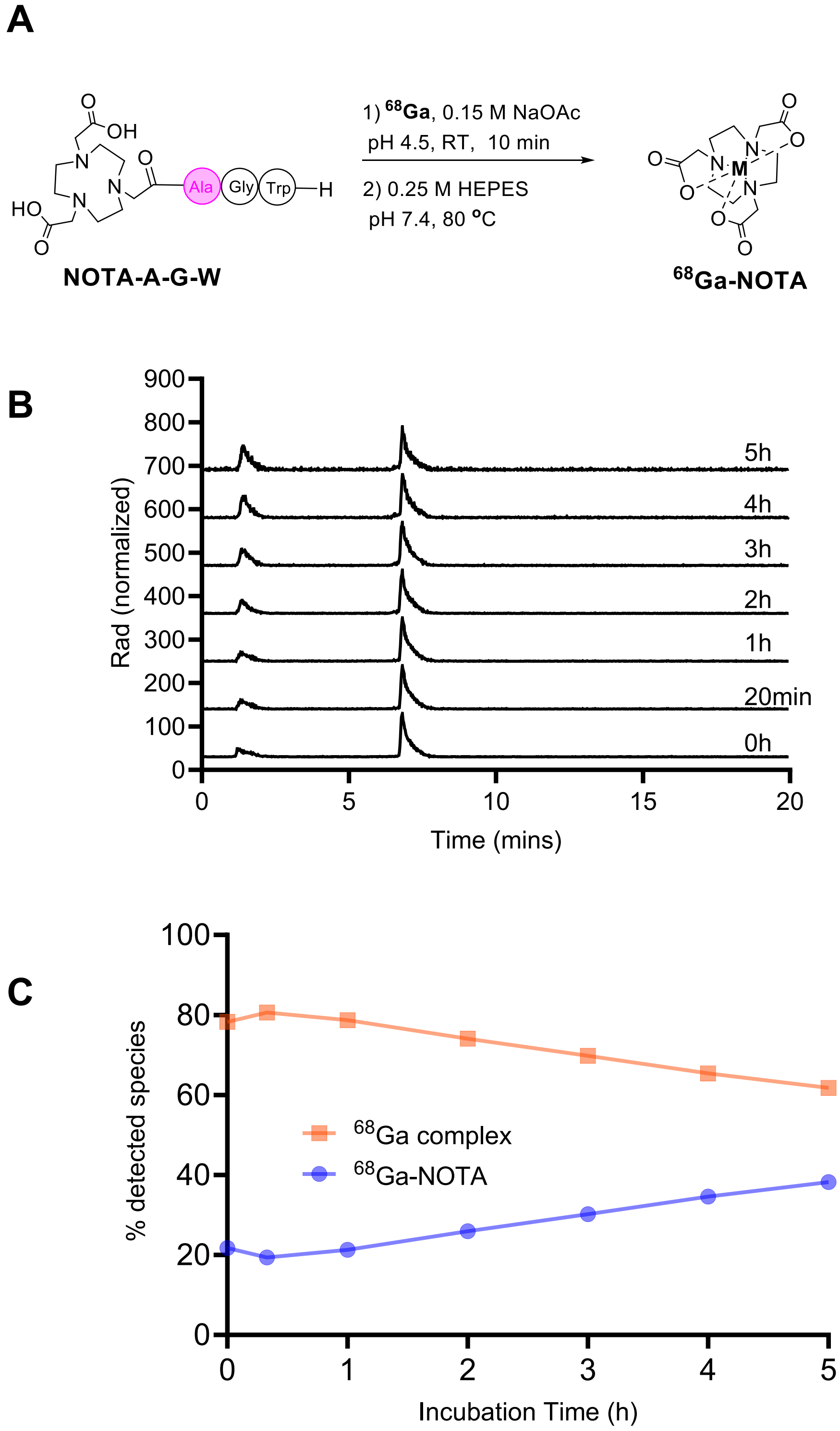

Figure S213. (A) Schematic description of preparation and direct radiolabeling procedure of Ala-containing radiopharmaceutical. **(B)** Analytical radioHPLC chromatograms showing autolytic release of the desired product [^{68}Ga]Ga(NOTA). **(C)** Quantification of the temperature-dependent release of radiopharmaceutical from [^{68}Ga][Ga(NO2A)]$^{+}$-A-G-W-CONH$_2$ at 80 °C and pH 7.4.

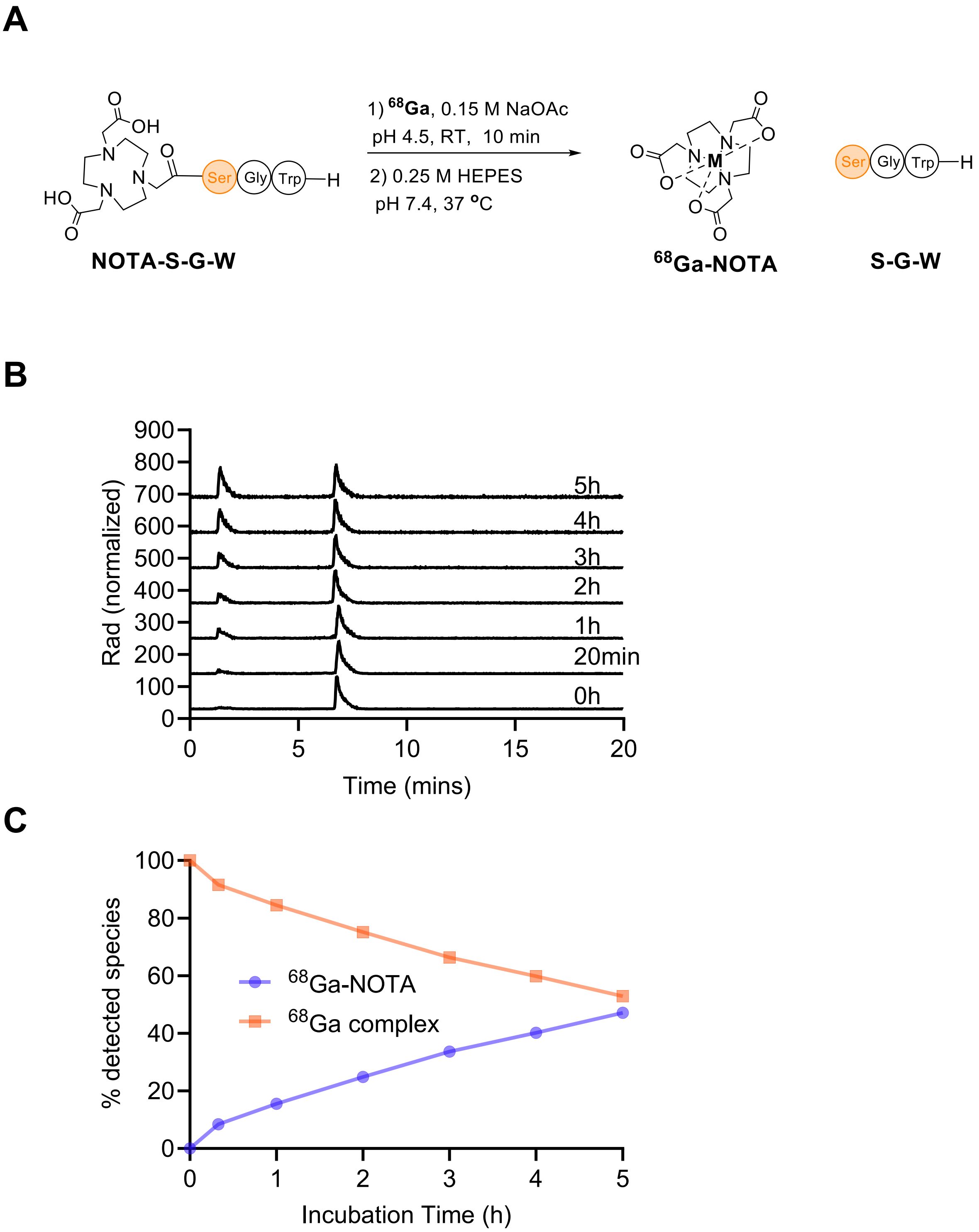

Figure S214. (A) Schematic description of preparation and direct radiolabeling procedure of Ser-containing radiopharmaceutical. **(B)** Analytical radioHPLC chromatograms showing autolytic release of the desired product [^{68}Ga]Ga(NOTA). **(C)** Quantification of the temperature-dependent release of radiopharmaceutical from [^{68}Ga][Ga(NO2A)]$^{+}$-S-G-W-$CONH_2$ at 80 ºC and pH 7.4.

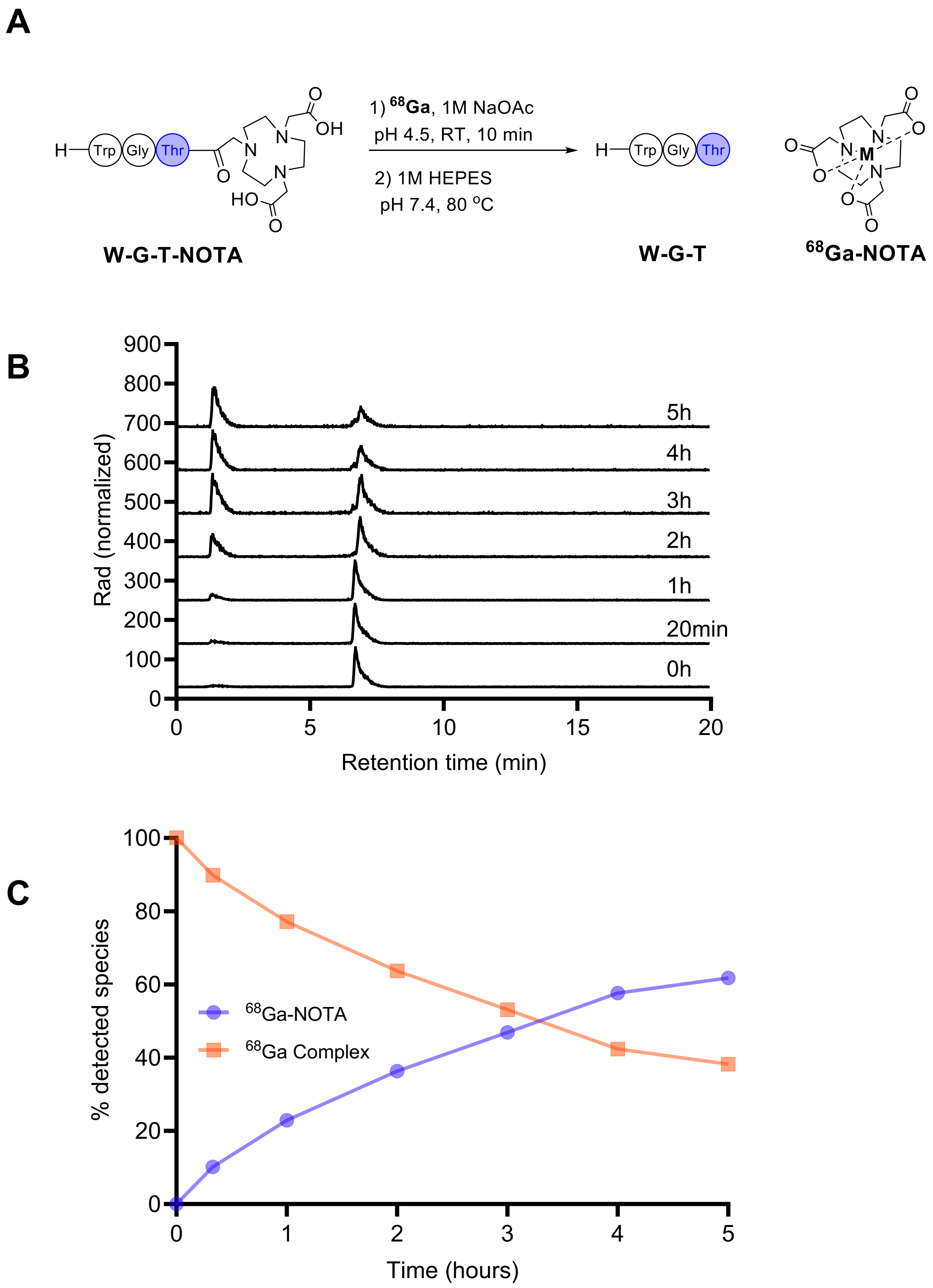

Figure S215. (A) Schematic description of preparation and direct radiolabeling procedure of Thr-containing radiopharmaceutical. **(B)** Analytical radioHPLC chromatograms showing autolytic release of the desired product [^{68}Ga]Ga(NOTA). **(C)** Quantification of the temperature-dependent release of radiopharmaceutical from [^{68}Ga][Ga(NO2A)]$^{+}$-T-G-W-CONH$_2$ at 80 °C and pH 7.4.

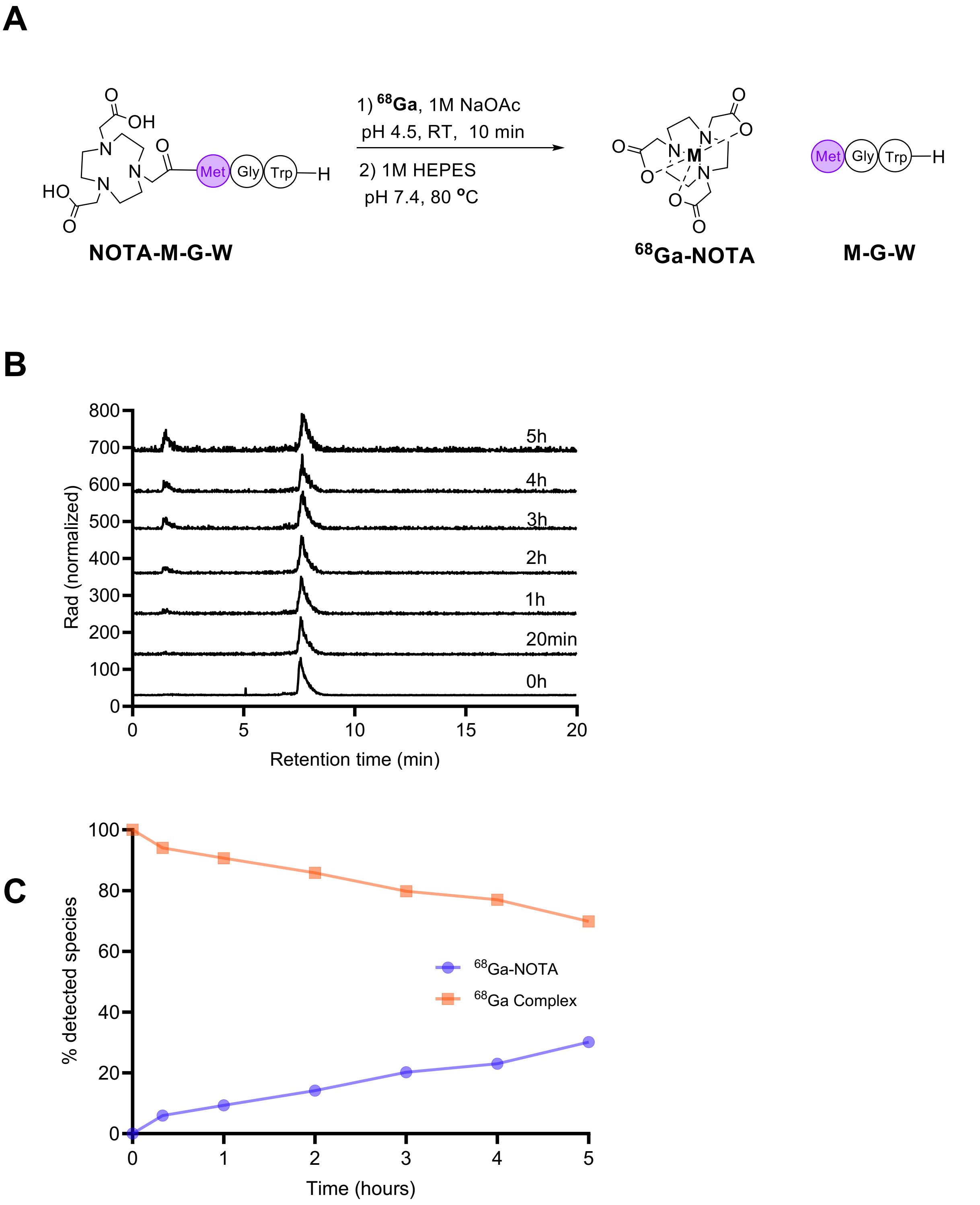

Figure S216. (A) Schematic description of preparation and direct radiolabeling procedure of Met-containing radiopharmaceutical. **(B)** Analytical radioHPLC chromatograms showing autolytic release of the desired product [^{68}Ga]Ga(NOTA). **(C)** Quantification of the temperature-dependent release of radiopharmaceutical from [^{68}Ga][Ga(NO2A)]$^{+}$-M-G-W-CONH$_2$ at 80 °C and pH 7.4.

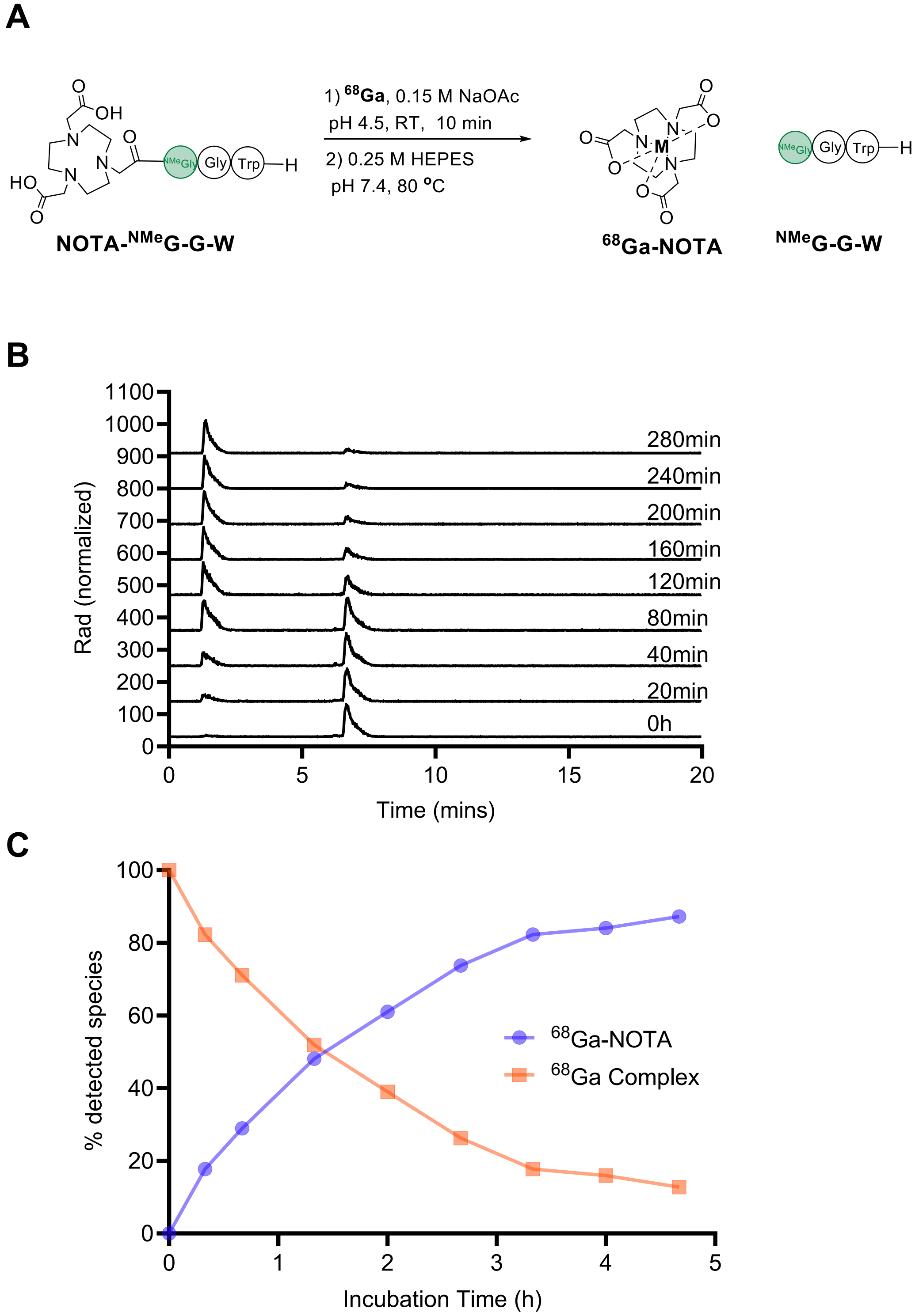

Figure S217. (A) Schematic description of preparation and direct radiolabeling procedure of ^{NMe}Gly-containing radiopharmaceutical. **(B)** Analytical radioHPLC chromatograms showing autolytic release of the desired product [^{68}Ga]Ga(NOTA). **(C)** Quantification of the temperature-dependent release of radiopharmaceutical from [^{68}Ga][Ga(NO2A)]$^{+}$-^{NMe}G-G-W-$CONH_2$ at 80 °C and pH 7.4.

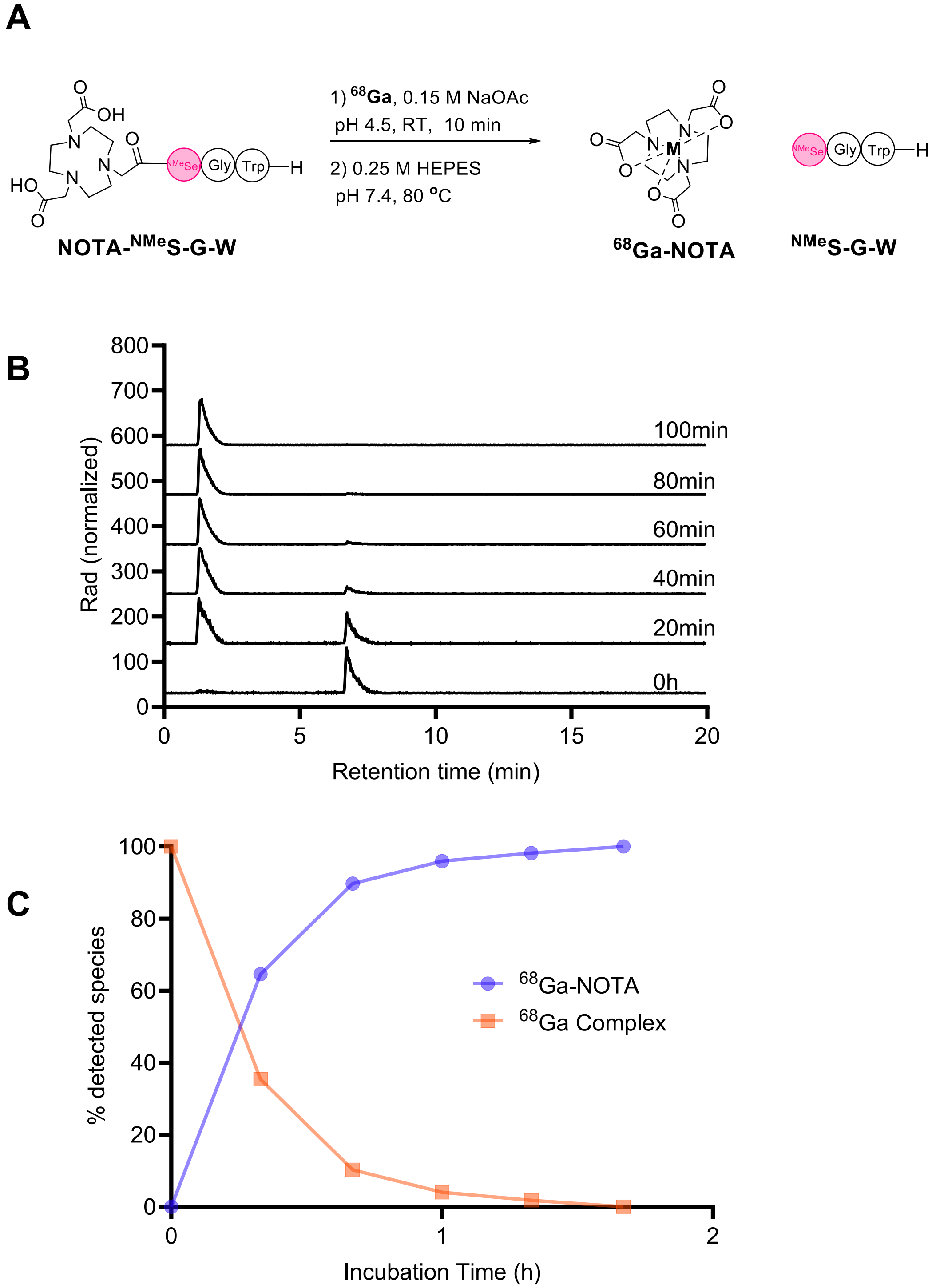

Figure S218. (A) Schematic description of preparation and direct radiolabeling procedure of ^{NMe}Ser-containing radiopharmaceutical. **(B)** Analytical radioHPLC chromatograms showing autolytic release of the desired product [^{68}Ga]Ga(NOTA). **(C)** Quantification of the temperature-dependent release of radiopharmaceutical from [^{68}Ga][Ga(NO2A)]$^{+}$-^{NMe}S-G-W-CONH$_2$ at 80 °C and pH 7.4.

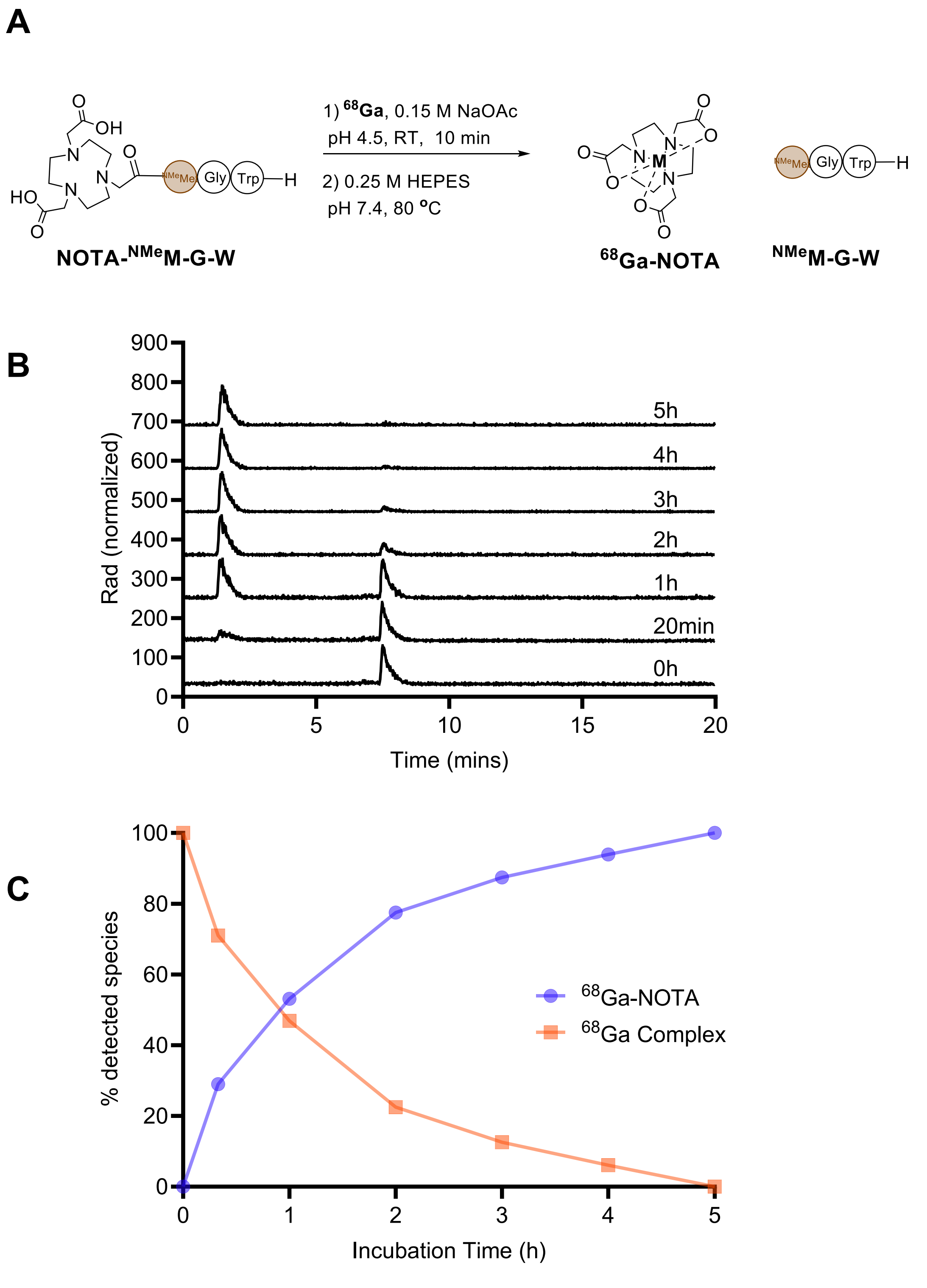

Figure S219. (A) Schematic description of preparation and direct radiolabeling procedure of ^{NMe}Met-containing radiopharmaceutical. **(B)** Analytical radioHPLC chromatograms showing autolytic release of the desired product [^{68}Ga]Ga(NOTA). **(C)** Quantification of the temperature-dependent release of radiopharmaceutical from [^{68}Ga][Ga(NO2A)]$^{+}$-^{NMe}M-G-W-CONH$_2$ at 80 °C and pH 7.4.

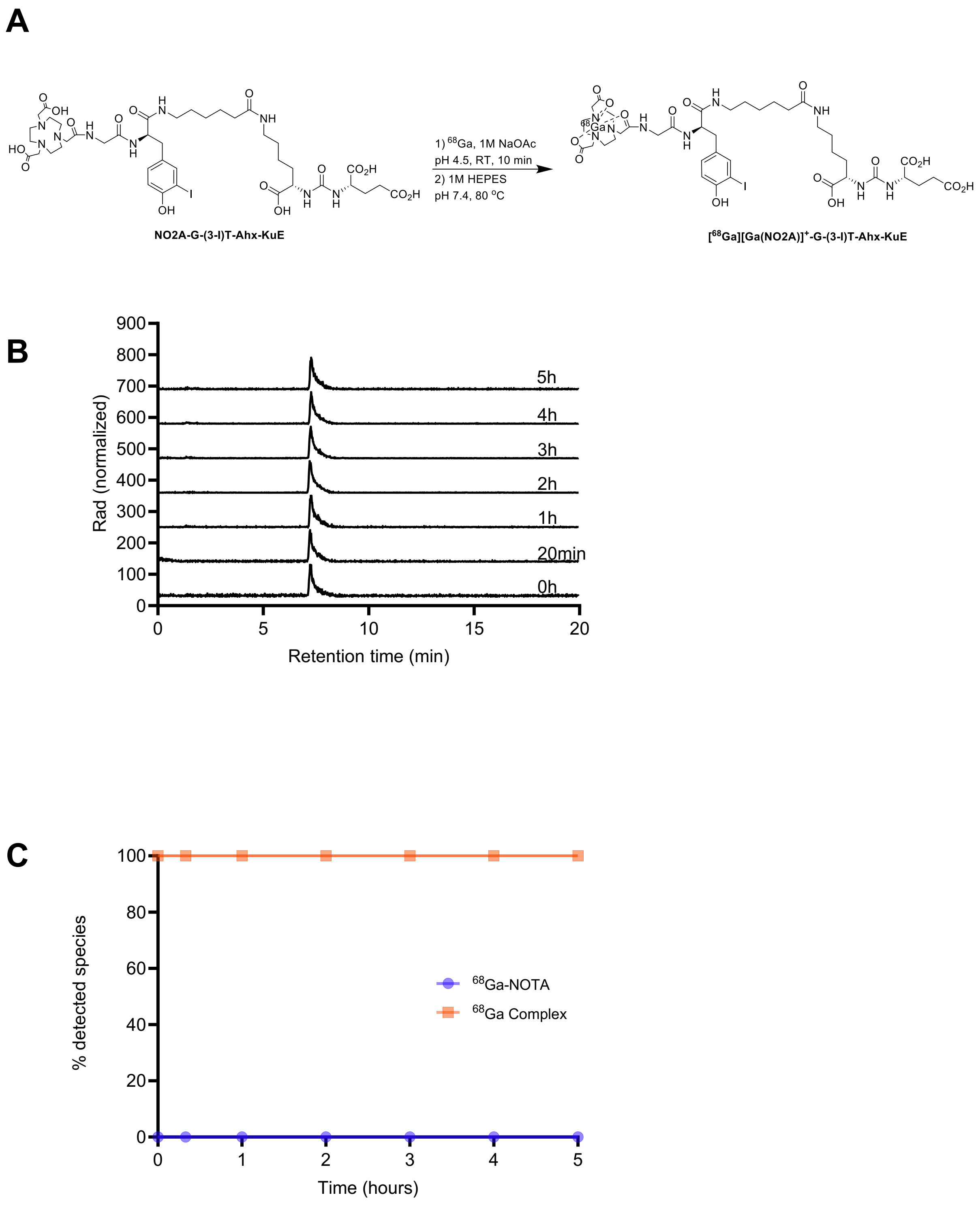

Figure S220. (A) Schematic description of preparation and direct radiolabeling procedure of conjugate **20**, NO2A-Gly-Tyr(3-I)-Ahx-KuE radiopharmaceutical. **(B)** Analytical HPLC chromatograms showing no autolytic release of the desired product [^{68}Ga]Ga(NOTA). **(C)** Quantification of the temperature-dependent cleavage at 80 °C and pH 7.4.

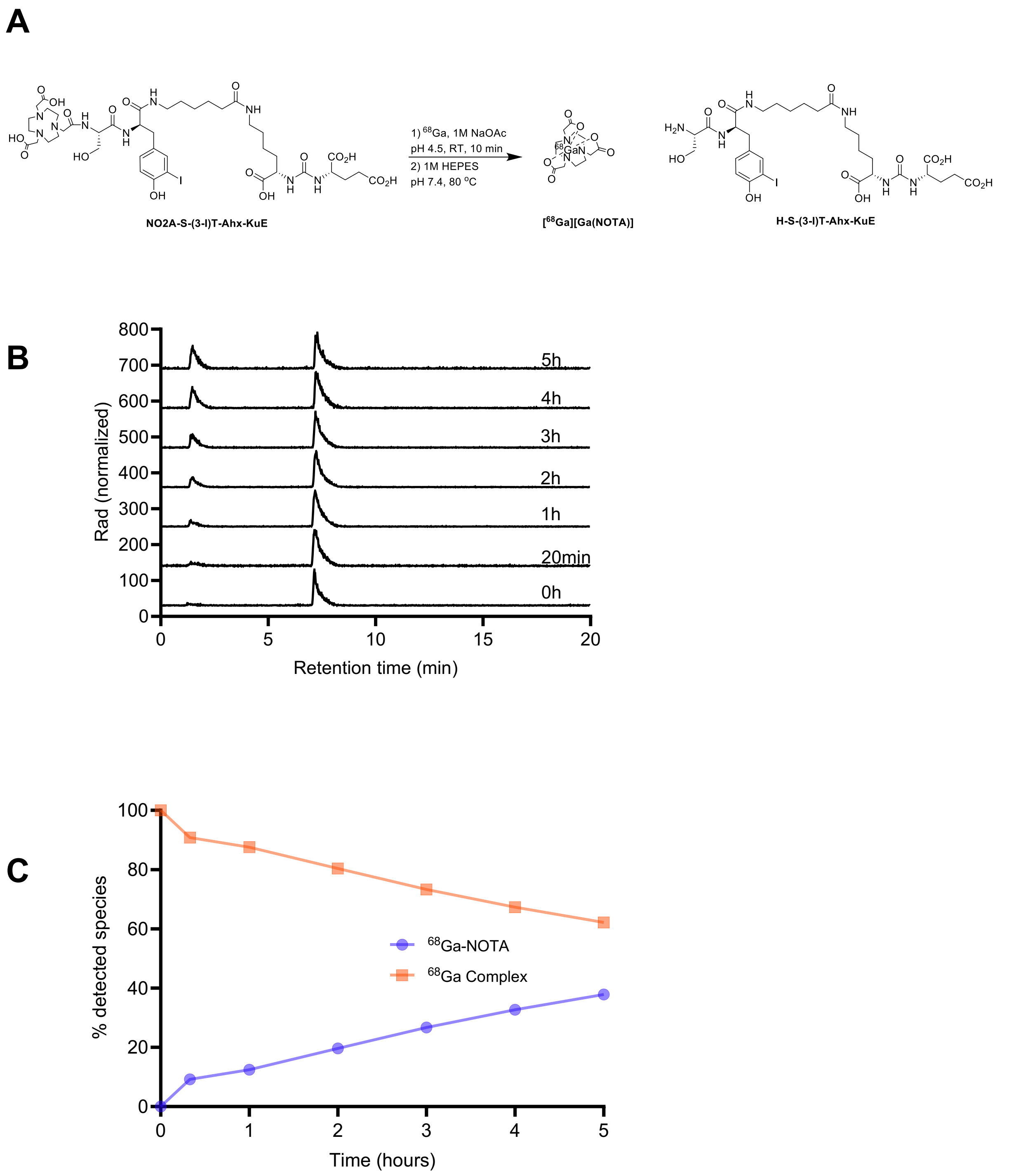

Figure S221. (A) Schematic description of preparation and direct radiolabeling procedure of **21**, NO2A-S-Tyr(3-I)-Ahx-KuE radiopharmaceutical. **(B)** Analytical radioHPLC chromatograms showing autolytic release of the desired product [^{68}Ga]Ga(NOTA). **(C)** Quantification of the temperature-dependent release of radiopharmaceutical from [^{68}Ga][Ga(NO2A)]$^{+}$-S-Tyr(3-I)-Ahx-KuE at 80 °C and pH 7.4.

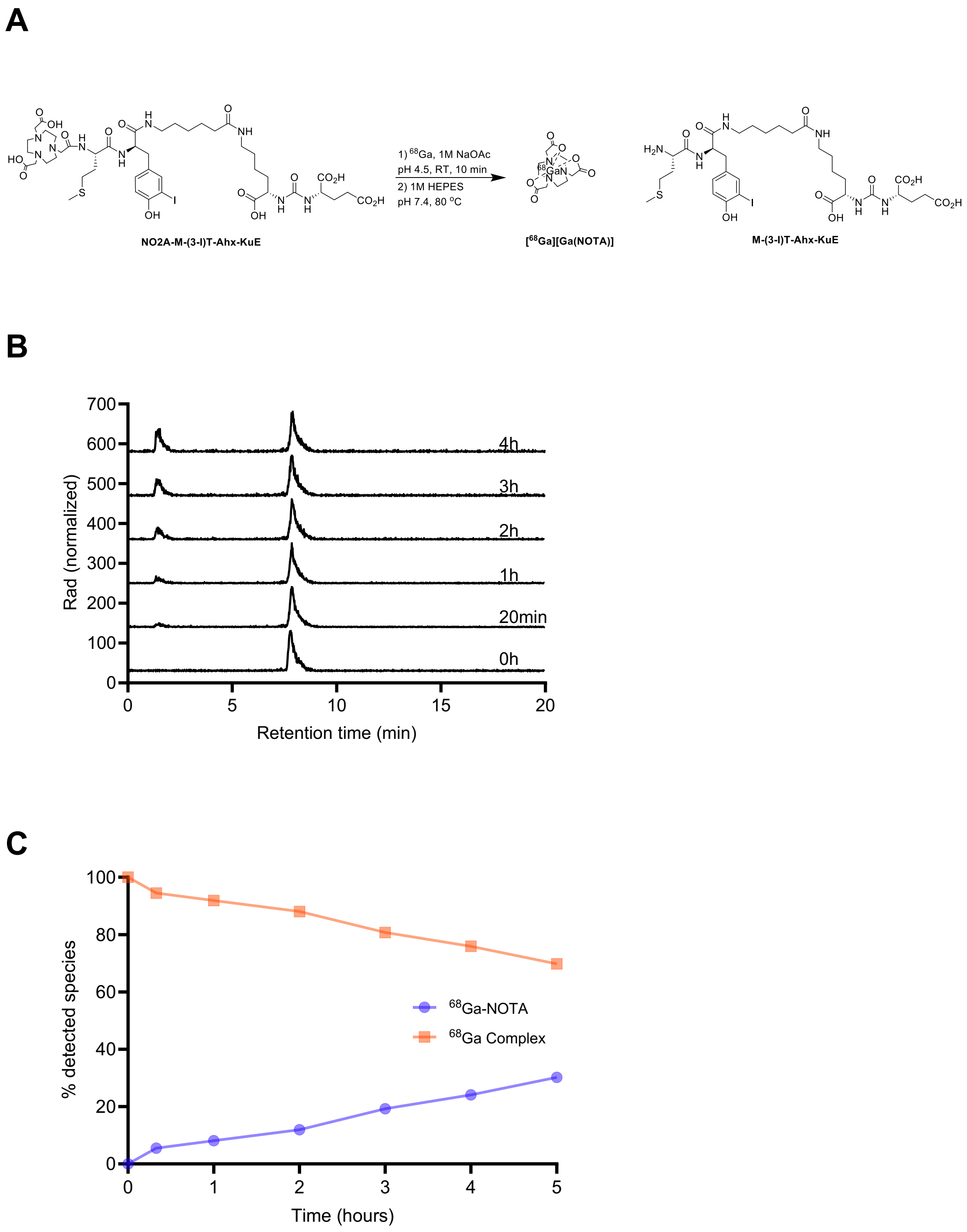

Figure S222. (A) Schematic description of preparation and direct radiolabeling procedure of **22**, NO2A-M-Tyr(3-I)-Ahx-KuE radiopharmaceutical. **(B)** Analytical radioHPLC chromatograms showing autolytic release of the desired product [^{68}Ga]Ga(NOTA). **(C)** Quantification of the temperature-dependent release of radiopharmaceutical from [^{68}Ga][Ga(NO2A)]$^{+}$-M-Tyr(3-I)-Ahx-KuE at 80 ºC and pH 7.4.

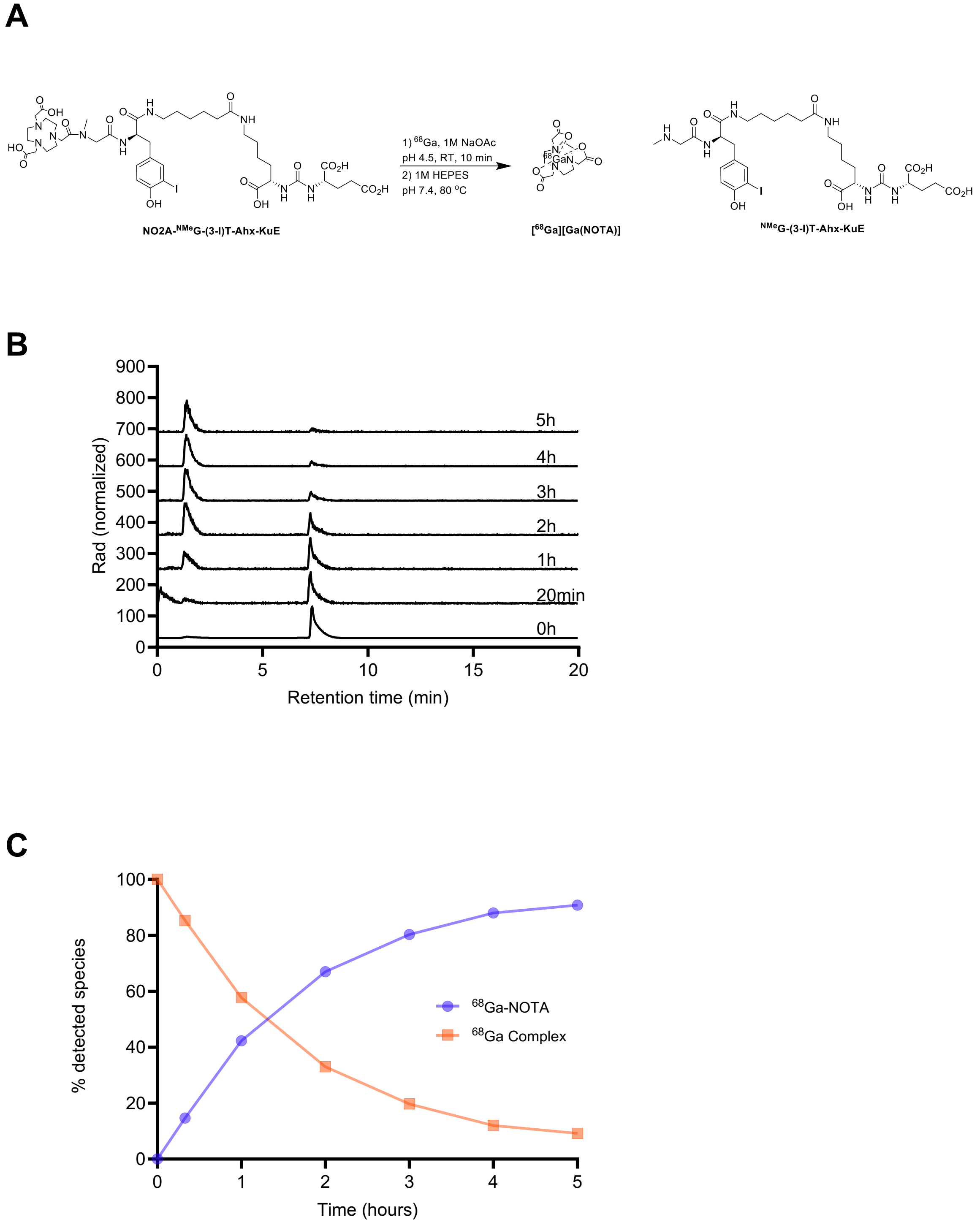

Figure S223. (A) Schematic description of preparation and direct radiolabeling procedure of **23**, NO2A-^{NMe}G-Tyr(3-I)-Ahx-KuE radiopharmaceutical. **(B)** Analytical radioHPLC chromatograms showing autolytic release of the desired product [^{68}Ga]Ga(NOTA). **(C)** Quantification of the temperature-dependent release of radiopharmaceutical from [^{68}Ga][Ga(NO2A)]$^{+}$-^{NMe}G-Tyr(3-I)-Ahx-KuE at 80 °C and pH 7.4.

A

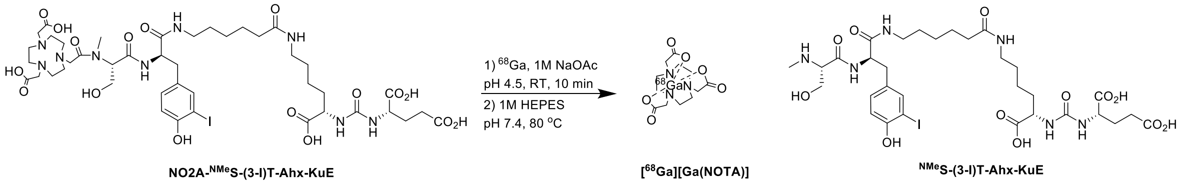

B

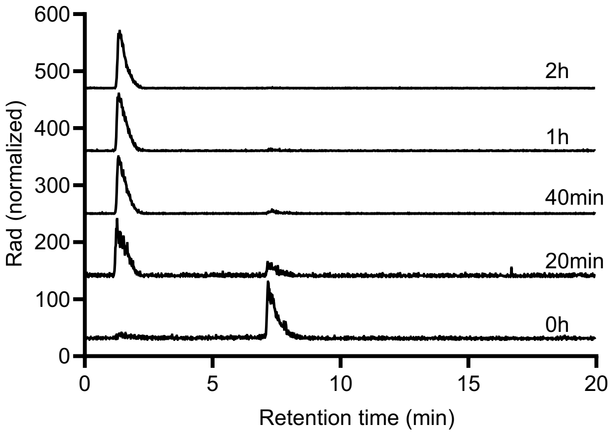

C

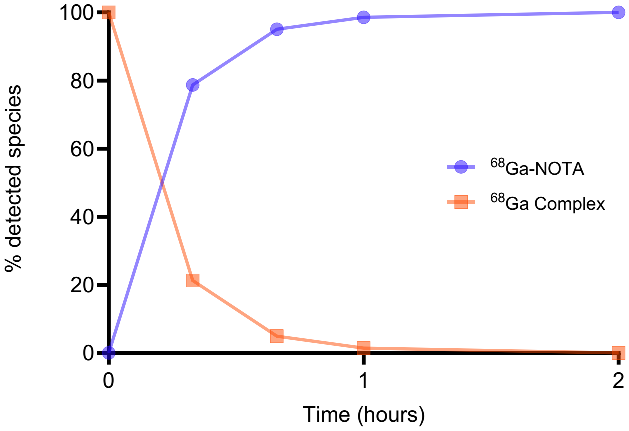

Figure S224. **(A)** Schematic description of preparation and direct radiolabeling procedure of **24**, NO2A-^{NMe}S-Tyr(3-I)-Ahx-KuE radiopharmaceutical. **(B)** Analytical radioHPLC chromatograms showing autolytic release of the desired product [^{68}Ga]Ga(NOTA). **(C)** Quantification of the temperature-dependent release of radiopharmaceutical from [^{68}Ga][Ga(NO2A)]$^{+}$-^{NMe}S-Tyr(3-I)-Ahx-KuE at 80 °C and pH 7.4.

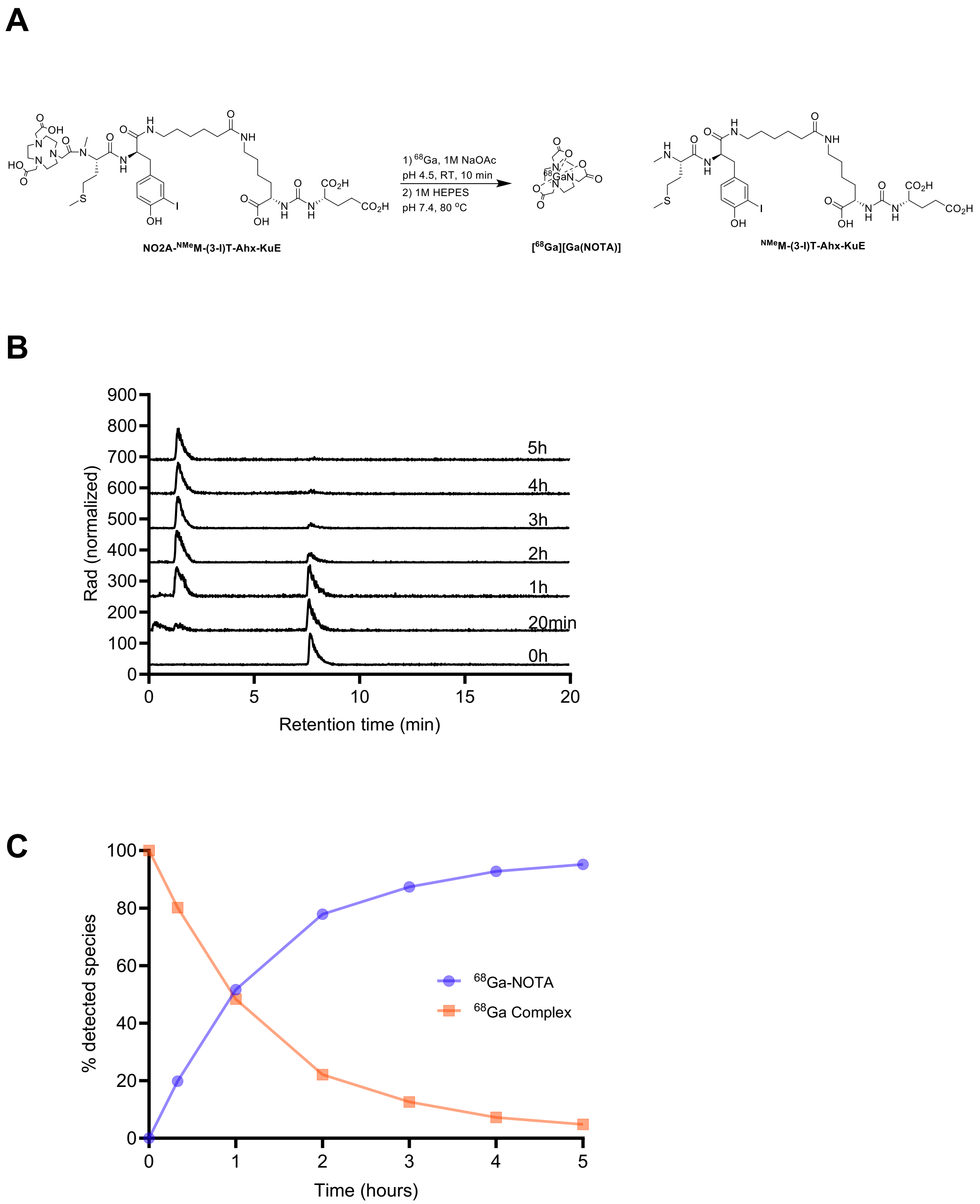

Figure S225. (A) Schematic description of preparation and direct radiolabeling procedure of **25**, NO2A-^{NMe}M-Tyr(3-I)-Ahx-KuE radiopharmaceutical. **(B)** Analytical radioHPLC chromatograms showing autolytic release of the desired product [^{68}Ga]Ga(NOTA). **(C)** Quantification of the temperature-dependent release of radiopharmaceutical from [^{68}Ga][Ga(NO2A)]$^{+}$-^{NMe}M-Tyr(3-I)-Ahx-KuE at 80 °C and pH 7.4.

Table S2. Comparative amide bond cleavage half-lives of ^{68}Ga-radiolabeled model tripeptide complexes and corresponding functionalized conjugates under pH 7.4 and 80 ^{o}C.

Amino acid linker R	Half-life of model tripeptide (h)	Half-life of PSMA-conjugate (h)
**Gly (20)**	*not observed*	*not observed*
**Ser (21)**	5.20	5.78
**Met (22)**	8.65	8.50
**^{NMe}Gly (23)**	1.13	1.18
**^{NMe}Ser (24)**	0.22	0.15
**^{NMe}Met (25)**	0.92	0.77

4.6 Cleavage Assay of ^{67}Ga-Radiolabeled Complexes.

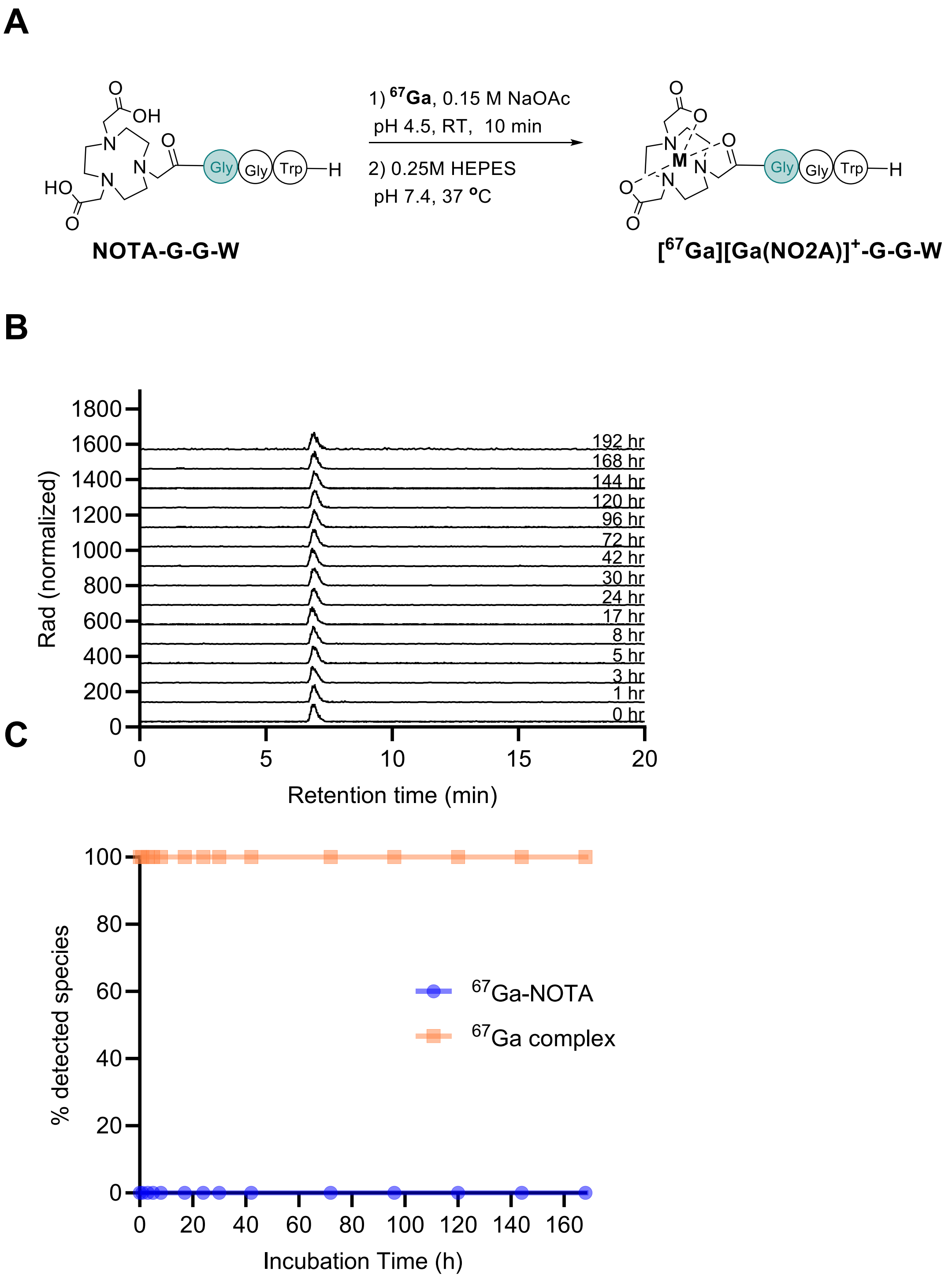

Figure S226. (A) Schematic description of preparation and direct radiolabeling procedure of Gly-containing radiopharmaceutical. **(B)** Analytical radioHPLC chromatograms showing no significant autolytic release of the desired product [^{67}Ga]Ga(NOTA). **(C)** Quantification of the temperature-dependent release of radiopharmaceutical from [^{67}Ga][Ga(NO2A)]$^{+}$-G-G-W-$CONH_2$ at 37 ºC and pH 7.4.

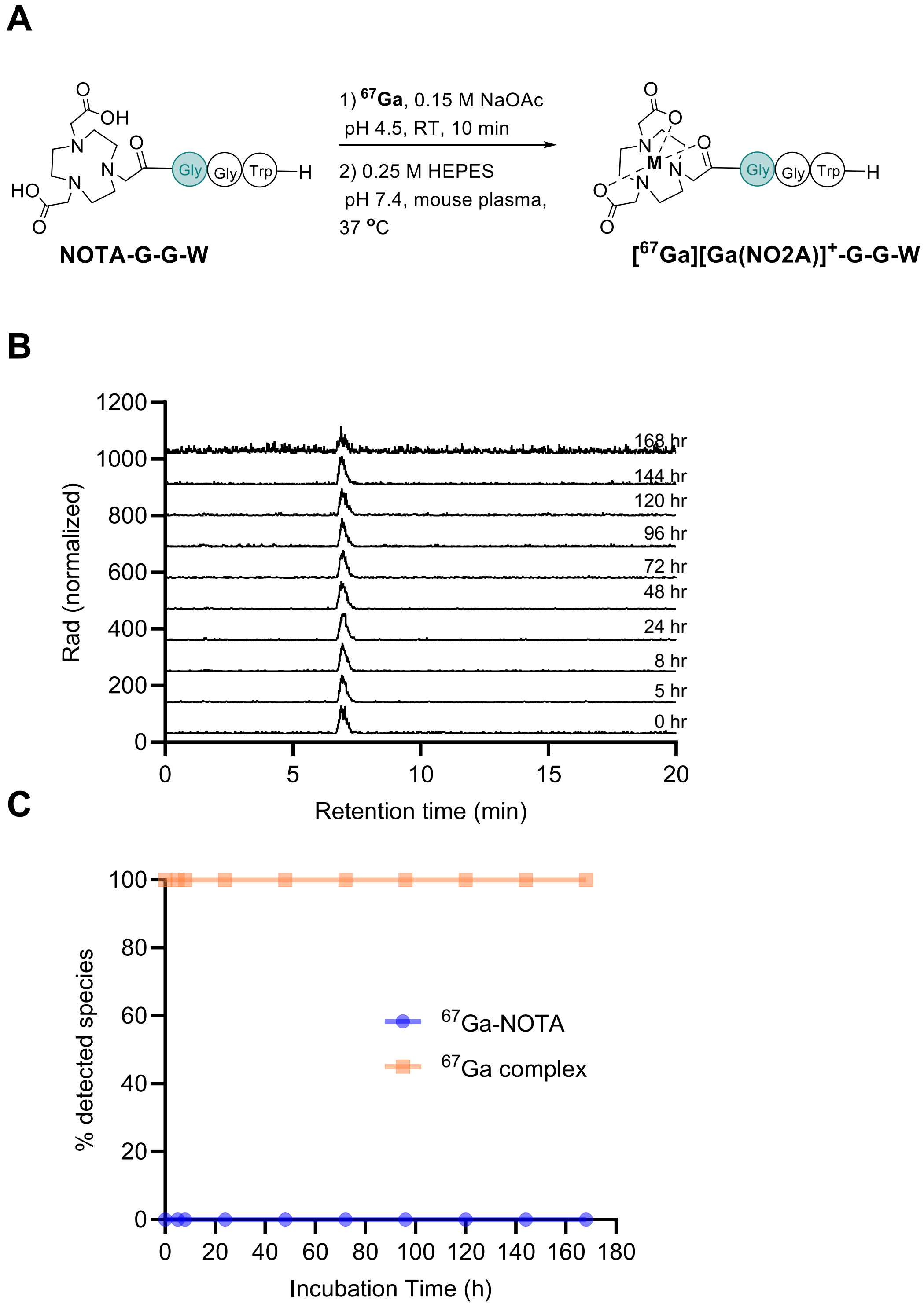

Figure S227. (A) Schematic description of preparation and direct radiolabeling procedure of Gly-containing radiopharmaceutical. **(B)** Analytical radioHPLC chromatograms showing no significant autolytic release of the desired product [^{67}Ga]Ga(NOTA). **(C)** Quantification of the temperature-dependent release of radiopharmaceutical from [^{67}Ga][Ga(NO2A)]$^{+}$-G-G-W-CONH$_2$ at 80 °C and pH 7.4.

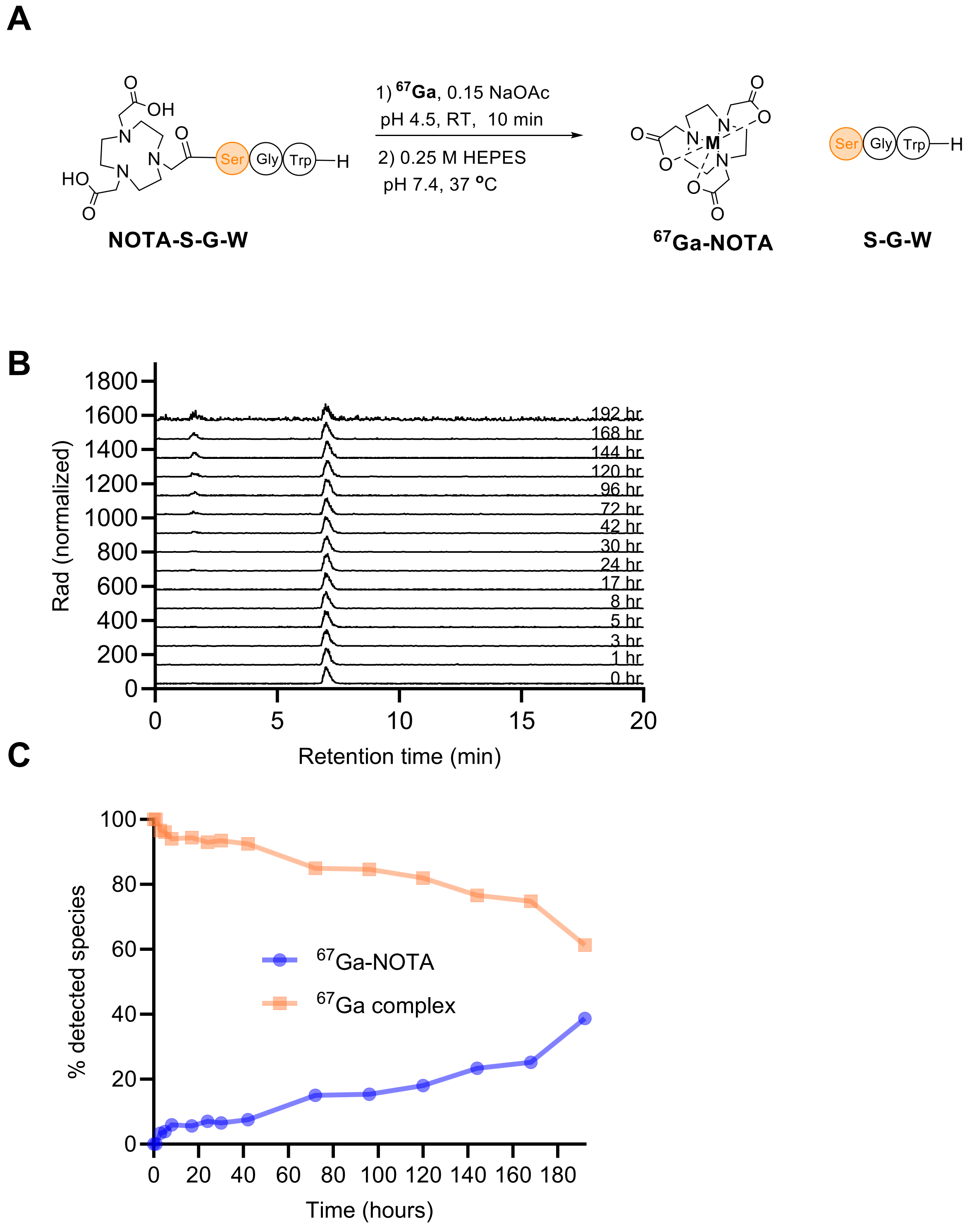

Figure S228. (A) Schematic description of preparation and direct radiolabeling procedure of Ser-containing radiopharmaceutical. **(B)** Analytical radioHPLC chromatograms showing autolytic release of the desired product [^{67}Ga]Ga(NOTA). **(C)** Quantification of the temperature-dependent release of radiopharmaceutical from [^{67}Ga][Ga(NO2A)]$^{+}$-S-G-W-CONH$_2$ at 37 °C and pH 7.4.

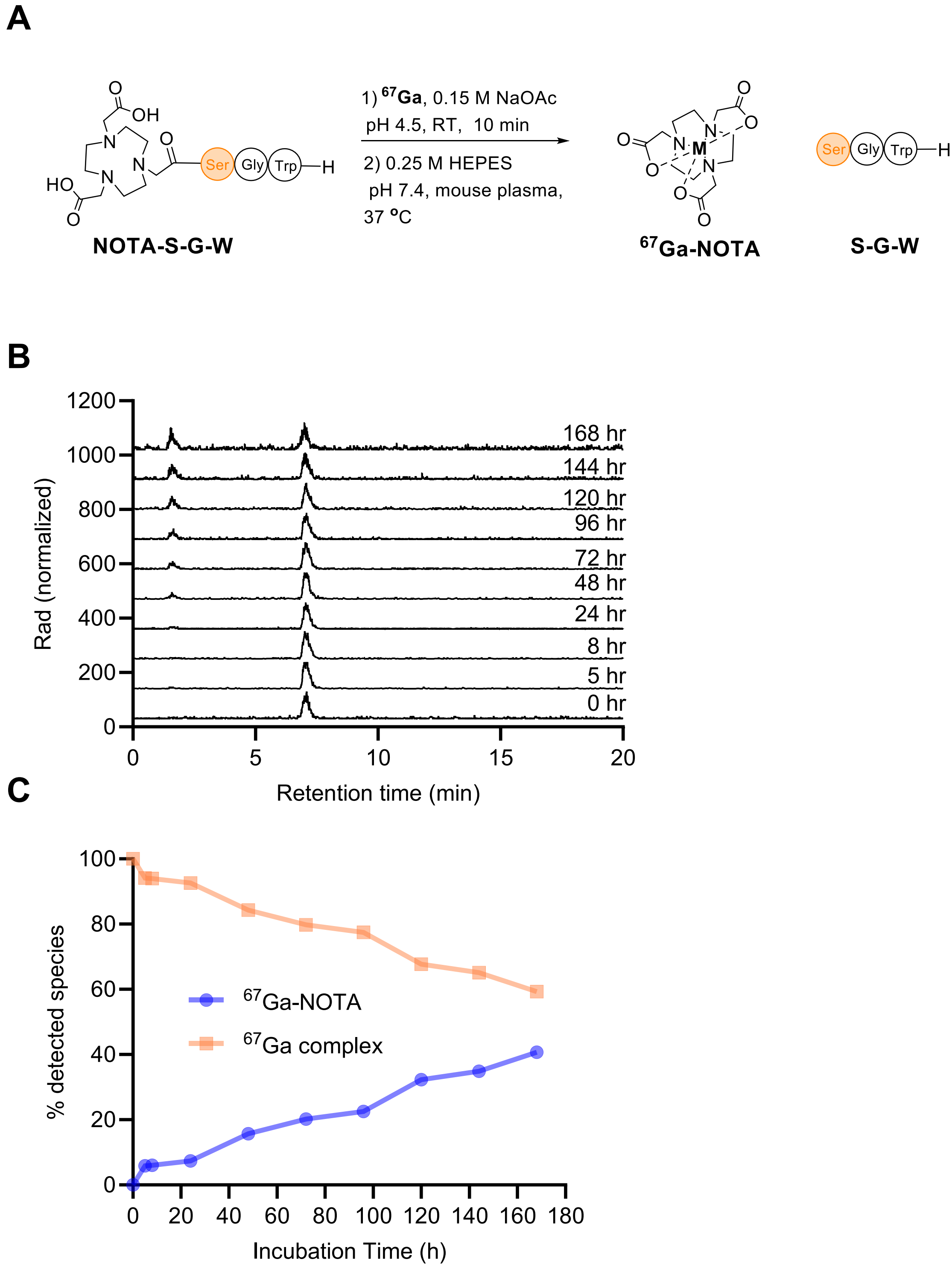

Figure S229. (A) Schematic description of preparation and direct radiolabeling procedure of Ser-containing radiopharmaceutical. **(B)** Analytical radioHPLC chromatograms showing autolytic release of the desired product [^{67}Ga]Ga(NOTA). **(C)** Quantification of the temperature-dependent release of radiopharmaceutical from [^{67}Ga][Ga(NO2A)]$^{+}$-S-G-W-CONH$_2$ at 37 ºC and pH 7.4.

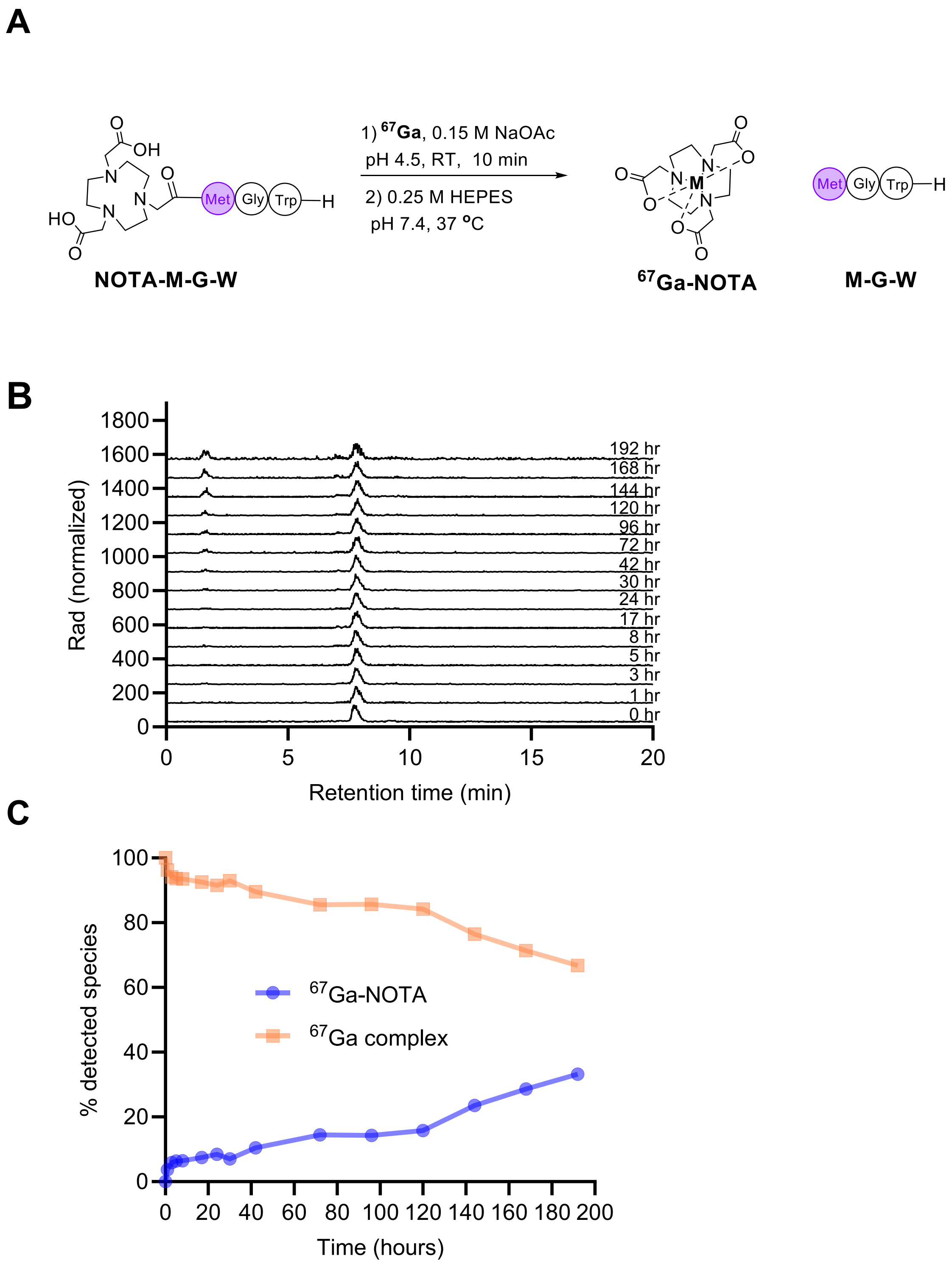

Figure S230. (A) Schematic description of preparation and direct radiolabeling procedure of Met-containing radiopharmaceutical. **(B)** Analytical radioHPLC chromatograms showing autolytic release of the desired product [^{67}Ga]Ga(NOTA). **(C)** Quantification of the temperature-dependent release of radiopharmaceutical from [^{67}Ga][Ga(NO2A)]$^{+}$-M-G-W-CONH$_2$ at 37 °C and pH 7.4.

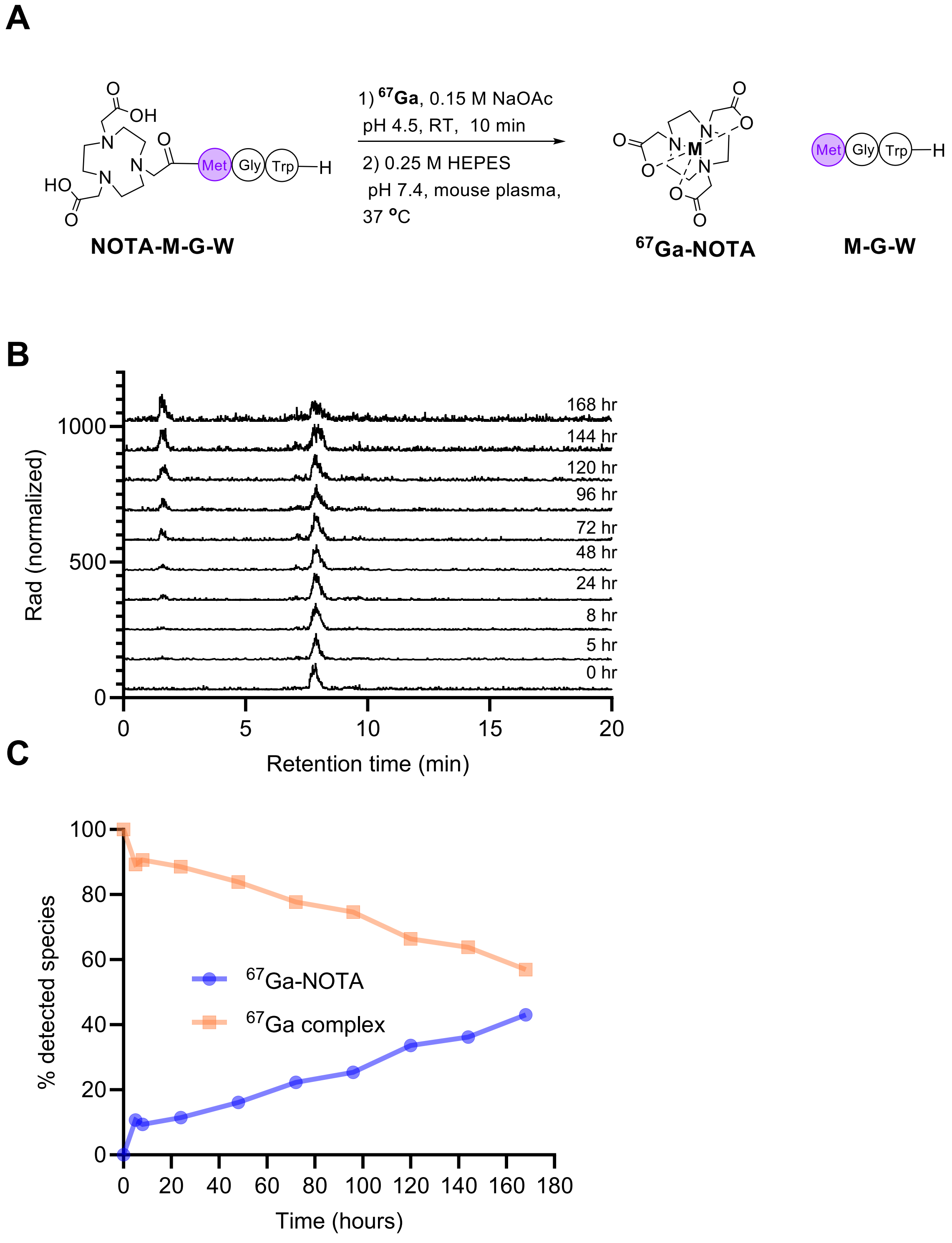

Figure S231. (A) Schematic description of preparation and direct radiolabeling procedure of Met-containing radiopharmaceutical. **(B)** Analytical radioHPLC chromatograms showing autolytic release of the desired product [^{67}Ga]Ga(NOTA). **(C)** Quantification of the temperature-dependent release of radiopharmaceutical from [^{67}Ga][Ga(NO2A)]$^{+}$-M-G-W-CONH$_2$ at 37 °C and pH 7.4.

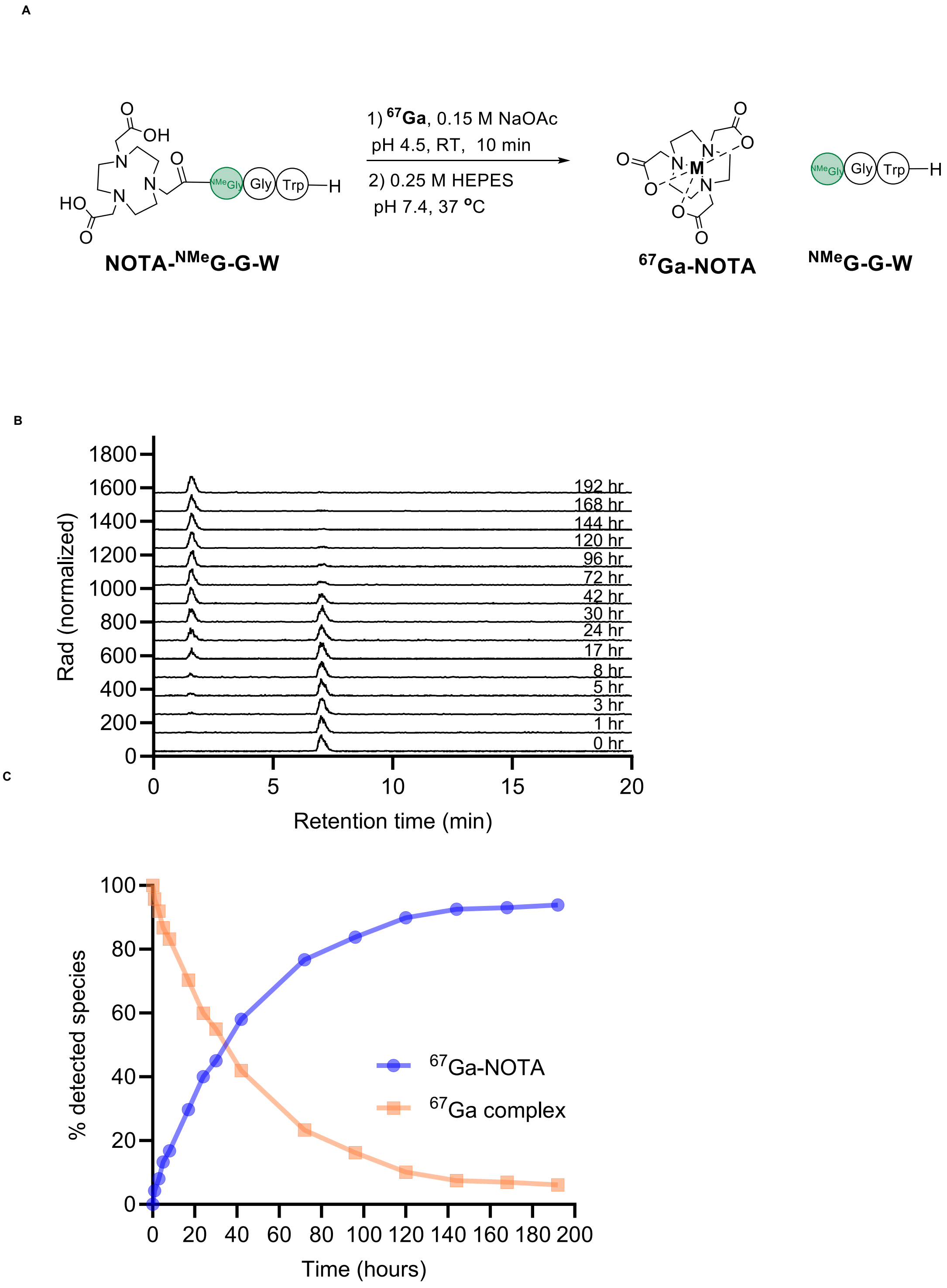

Figure S232. (A) Schematic description of preparation and direct radiolabeling procedure of ^{NMe}Gly-containing radiopharmaceutical. (B) Analytical radioHPLC chromatograms showing autolytic release of the desired product [^{67}Ga]Ga(NOTA). (C) Quantification of the temperature-dependent release of radiopharmaceutical from [^{67}Ga][Ga(NO2A)]$^{+}$-^{NMe}G-G-W-CONH$_2$ at 37 °C and pH 7.4.

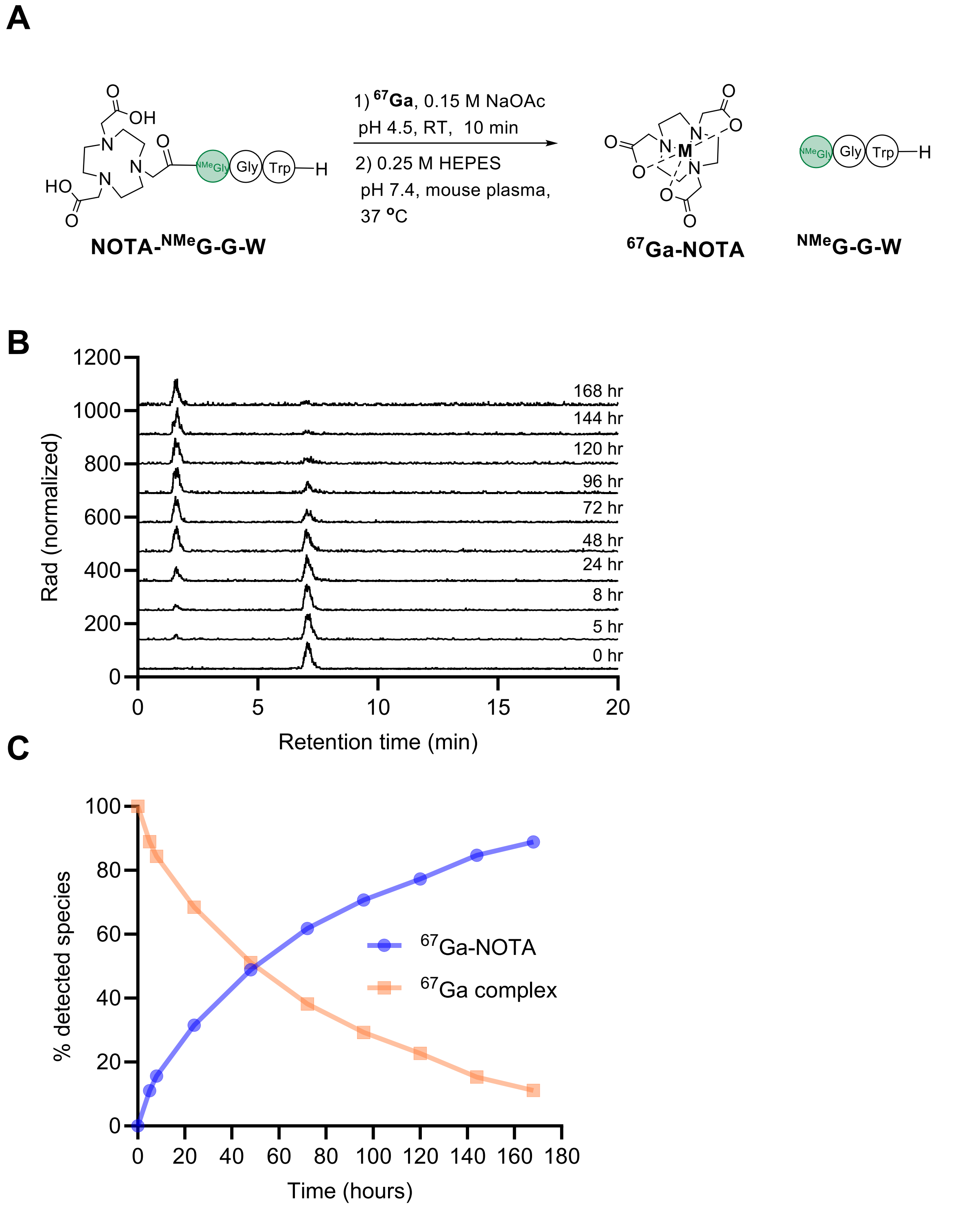

Figure S233. (A) Schematic description of preparation and direct radiolabeling procedure of ^{NMe}Gly-containing radiopharmaceutical. **(B)** Analytical radioHPLC chromatograms showing autolytic release of the desired product [^{67}Ga]Ga(NOTA). **(C)** Quantification of the temperature-dependent release of radiopharmaceutical from [^{67}Ga][Ga(NO2A)]$^{+}$-^{NMe}G-G-W-CONH$_2$ in mouse plasma at 37 °C and pH 7.4.

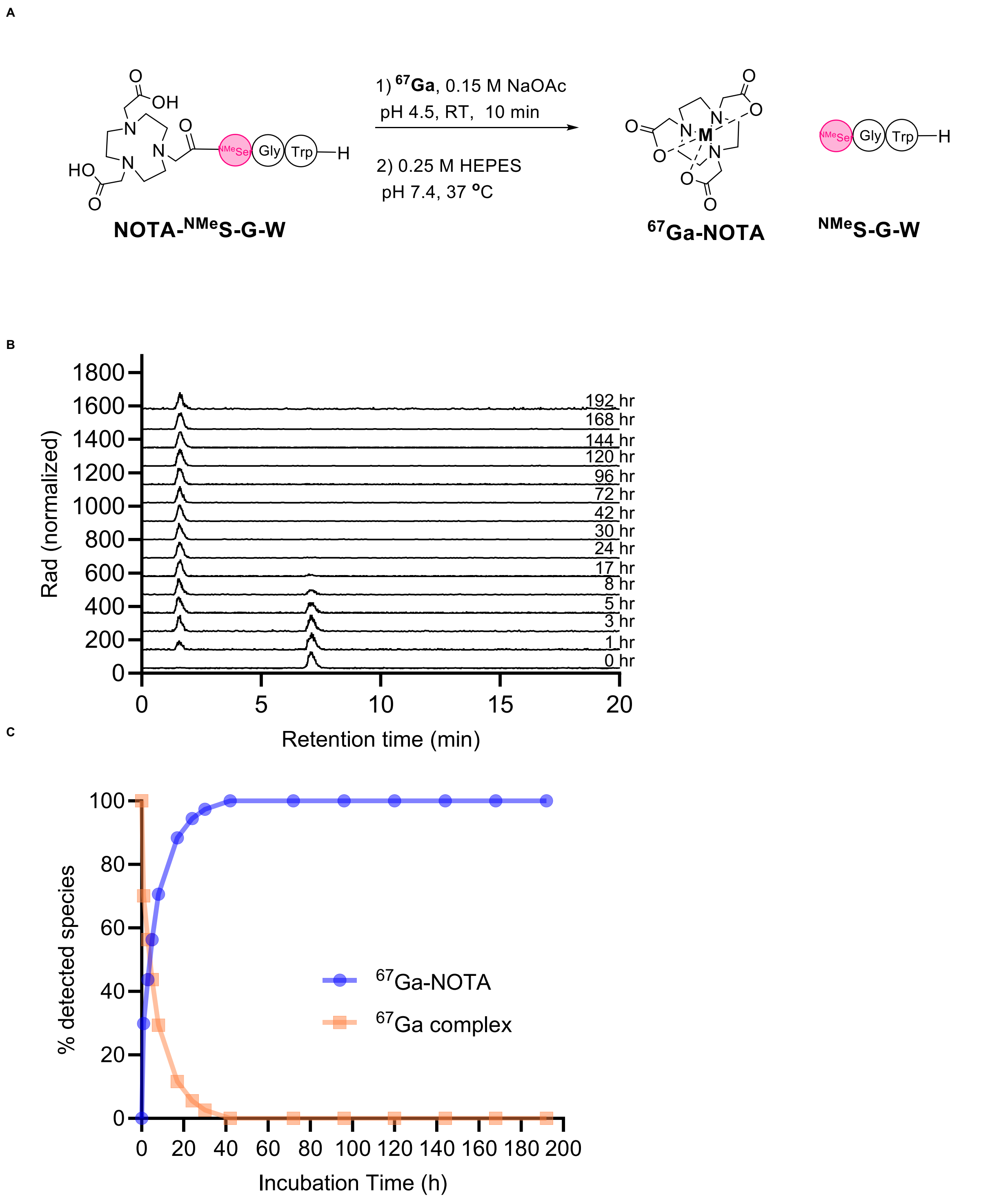

Figure S234. (A) Schematic description of preparation and direct radiolabeling procedure of ^{NMe}Ser-containing radiopharmaceutical. **(B)** Analytical radioHPLC chromatograms showing autolytic release of the desired product [^{67}Ga]Ga(NOTA). **(C)** Quantification of the temperature-dependent release of radiopharmaceutical from [^{67}Ga][Ga(NO2A)]$^{+}$-^{NMe}S-G-W-CONH$_2$ in mouse plasma at 37 ºC and pH 7.4.

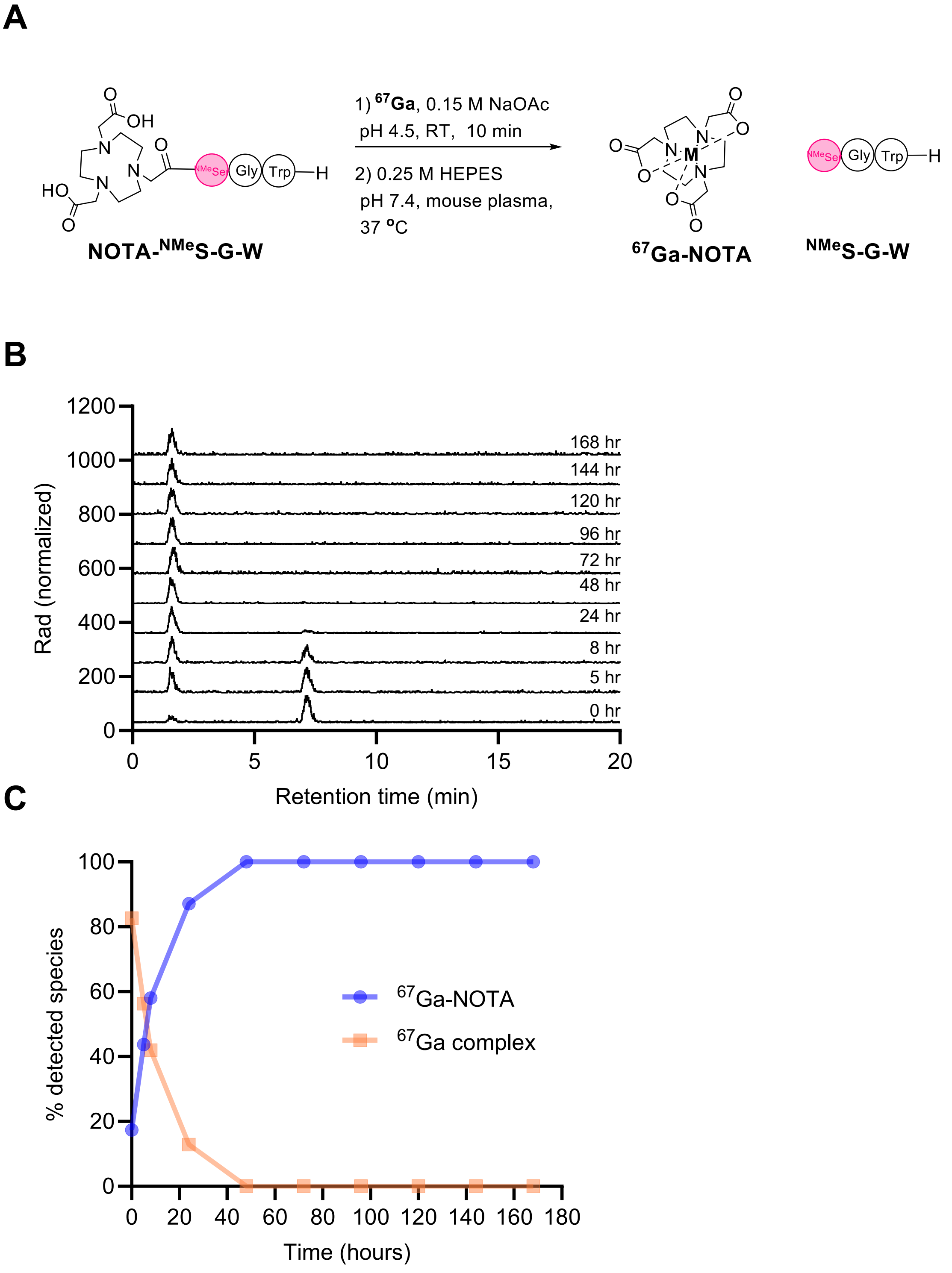

Figure S235. (A) Schematic description of preparation and direct radiolabeling procedure of ^{NMe}Ser-containing radiopharmaceutical. **(B)** Analytical radioHPLC chromatograms showing autolytic release of the desired product [^{67}Ga]Ga(NOTA). **(C)** Quantification of the temperature-dependent release of radiopharmaceutical from [^{67}Ga][Ga(NO2A)]$^{+}$-^{NMe}S-G-W-CONH$_2$ in mouse plasma at 37 °C and pH 7.4.

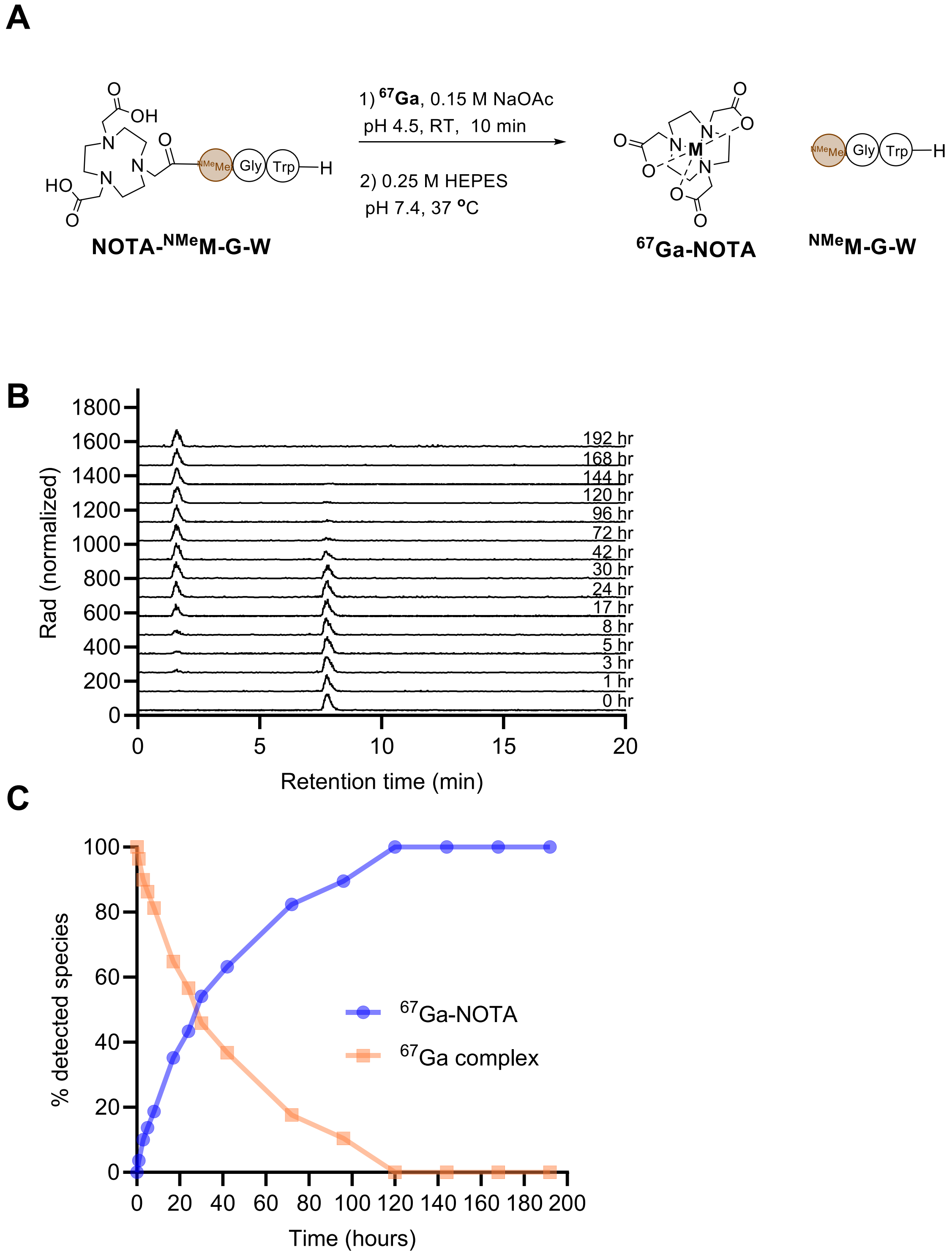

Figure S236. **(A)** Schematic description of preparation and direct radiolabeling procedure of ^{NMe}Met-containing radiopharmaceutical. **(B)** Analytical radioHPLC chromatograms showing autolytic release of the desired product [^{67}Ga]Ga(NOTA). **(C)** Quantification of the temperature-dependent release of radiopharmaceutical from [^{67}Ga][Ga(NO2A)]$^{+}$-^{NMe}M-G-W-$CONH_2$ at 37 °C and pH 7.4.

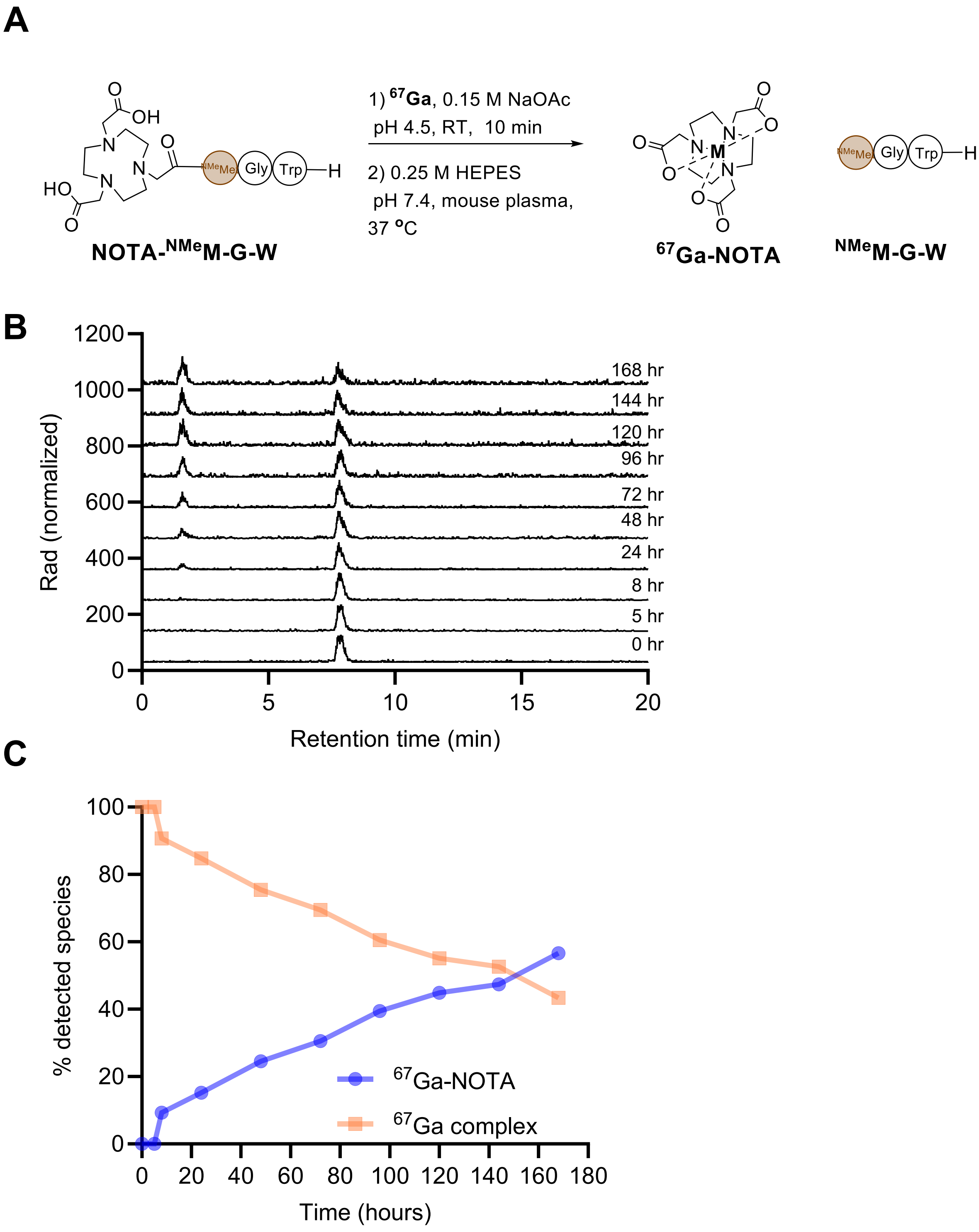

Figure S237. (A) Schematic description of preparation and direct radiolabeling procedure of ^{NMe}Met-containing radiopharmaceutical. **(B)** Analytical radioHPLC chromatograms showing autolytic release of the desired product [^{67}Ga]Ga(NOTA). **(C)** Quantification of the temperature-dependent release of radiopharmaceutical from [^{67}Ga][Ga(NO2A)]$^{+}$-^{NMe}M-G-W-CONH$_2$ in mouse plasma at 37 ºC and pH 7.4.

A

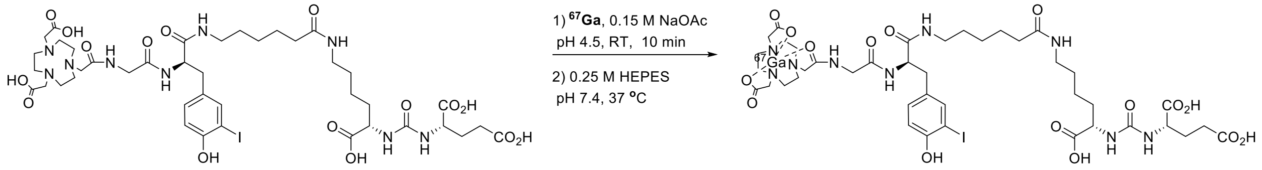

B

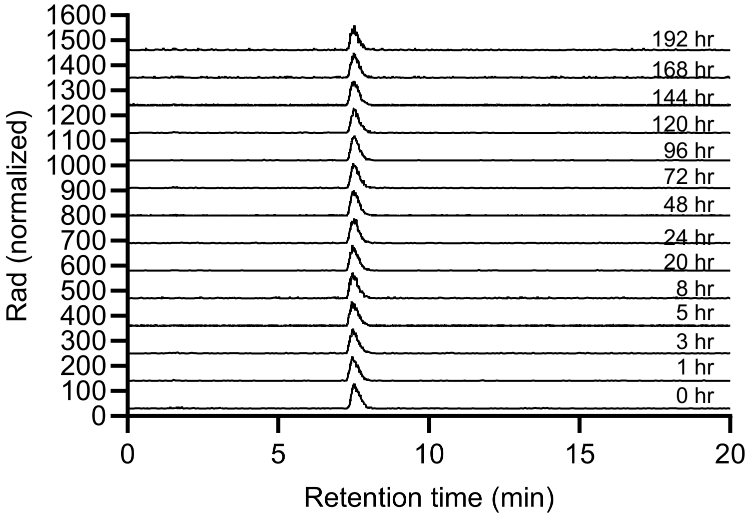

C

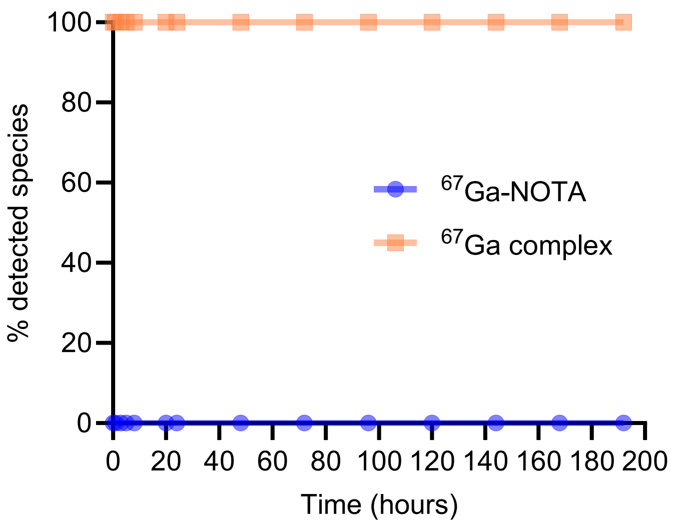

Figure S238. **(A)** Schematic description of preparation and direct radiolabeling procedure of conjugate **20**, NO2A-Gly-Tyr(3-I)-Ahx-KuE radiopharmaceutical. **(B)** Analytical HPLC chromatograms showing no autolytic release of the desired product [^{67}Ga]Ga(NOTA). **(C)** Quantification of the temperature-dependent cleavage at 37 °C and pH 7.4.

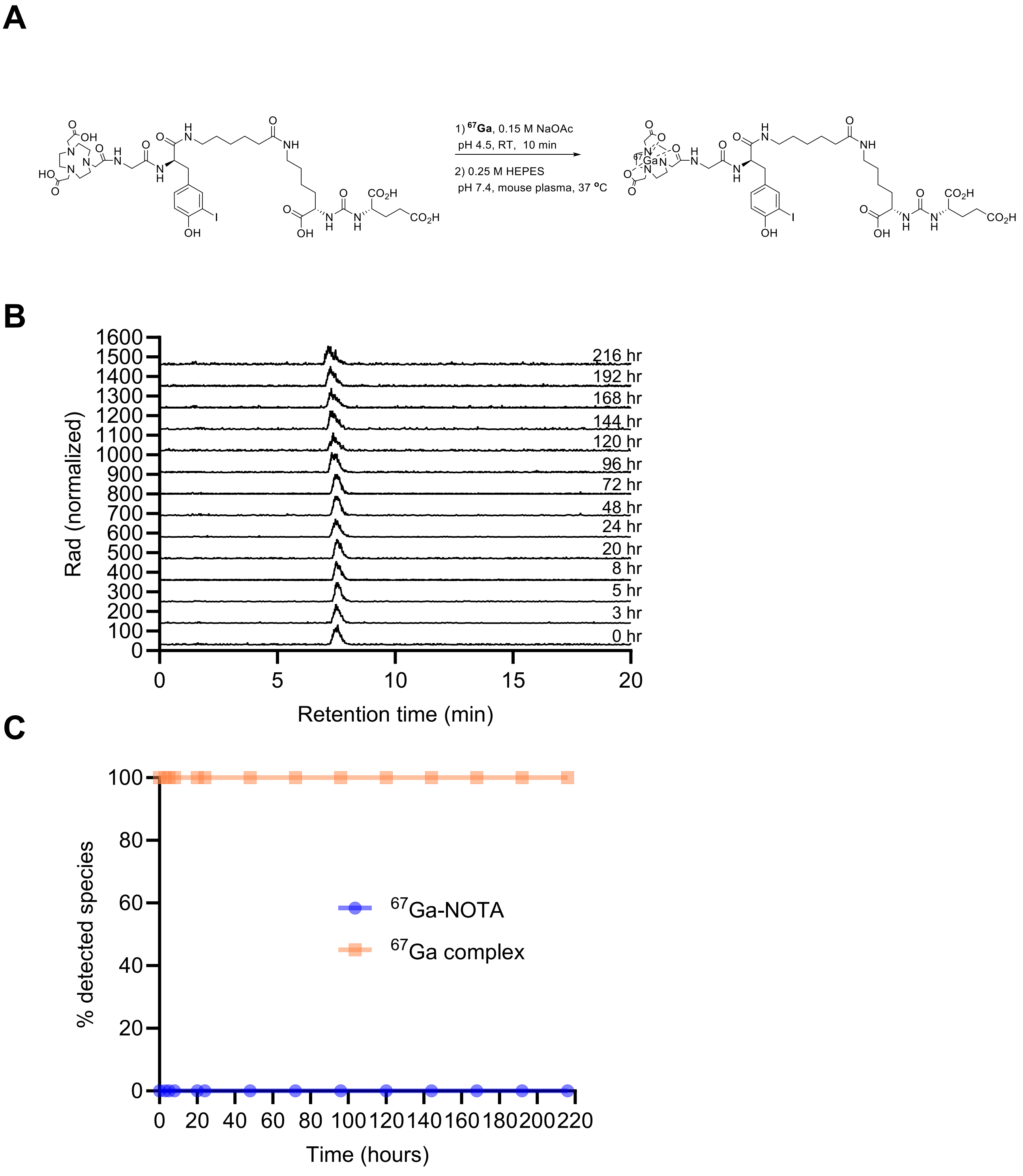

Figure S239. (A) Schematic description of preparation and direct radiolabeling procedure of conjugate **20**, NO2A-Gly-Tyr(3-I)-Ahx-KuE radiopharmaceutical. **(B)** Analytical HPLC chromatograms showing no autolytic release of the desired product [^{67}Ga]Ga(NOTA). **(C)** Quantification of the temperature-dependent cleavage in mouse plasma at 37 °C and pH 7.4.

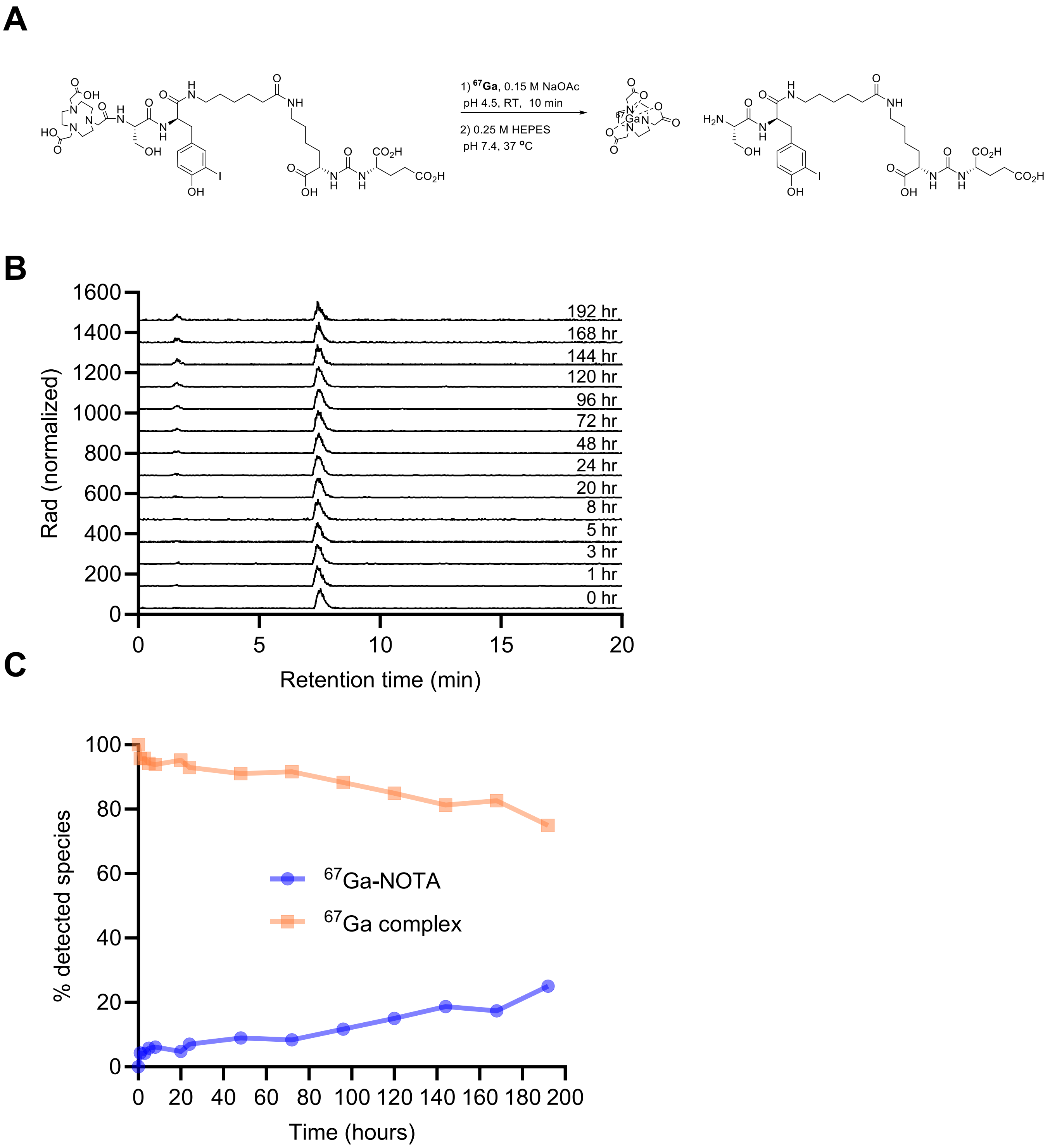

Figure S240. (A) Schematic description of preparation and direct radiolabeling procedure of **21**, NO2A-S-Tyr(3-I)-Ahx-KuE radiopharmaceutical. **(B)** Analytical radioHPLC chromatograms showing autolytic release of the desired product [^{67}Ga]Ga(NOTA). **(C)** Quantification of the temperature-dependent release of radiopharmaceutical from [^{67}Ga][Ga(NO2A)]$^{+}$-S-Tyr(3-I)-Ahx-KuE at 37 °C and pH 7.4.

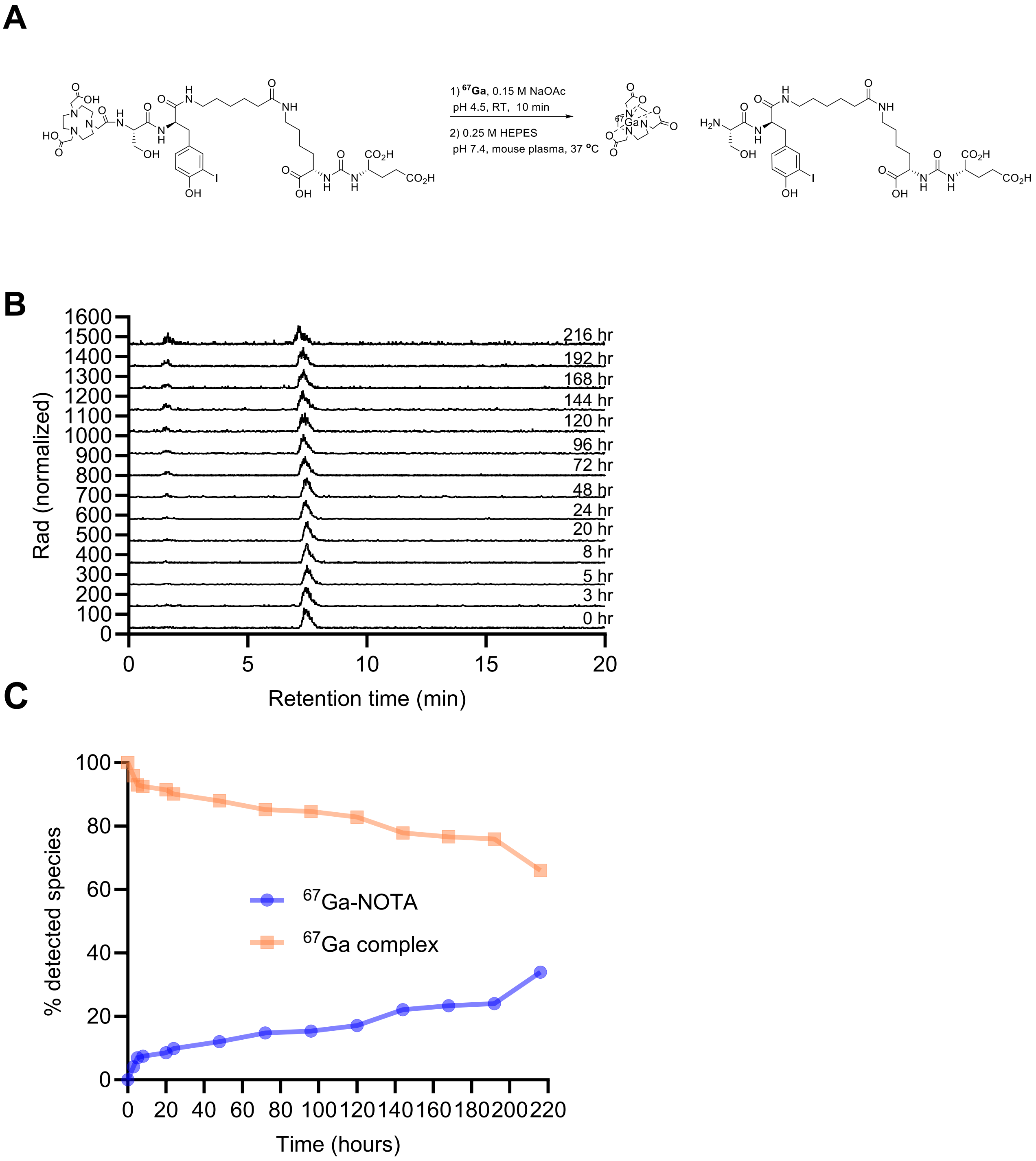

Figure S241. **(A)** Schematic description of preparation and direct radiolabeling procedure of **21**, NO2A-S-Tyr(3-I)-Ahx-KuE radiopharmaceutical. **(B)** Analytical radioHPLC chromatograms showing autolytic release of the desired product [^{67}Ga]Ga(NOTA). **(C)** Quantification of the temperature-dependent release of radiopharmaceutical from [^{67}Ga][Ga(NO2A)]$^{+}$-S-Tyr(3-I)-Ahx-KuE in mouse plasma at 37 ºC and pH 7.4.

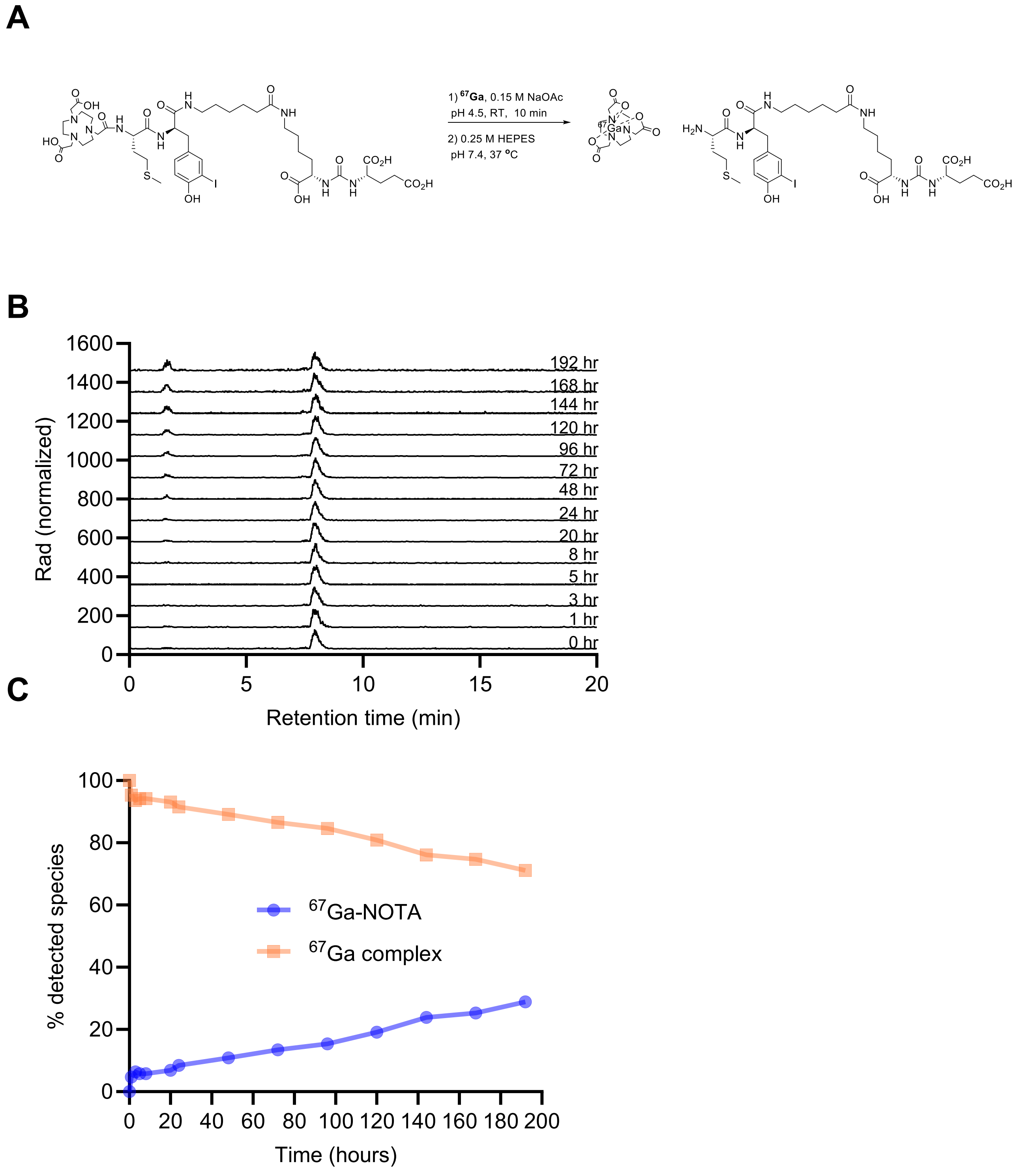

Figure S242. **(A)** Schematic description of preparation and direct radiolabeling procedure of **22**, NO2A-M-Tyr(3-I)-Ahx-KuE radiopharmaceutical. **(B)** Analytical radioHPLC chromatograms showing autolytic release of the desired product [^{67}Ga]Ga(NOTA). **(C)** Quantification of the temperature-dependent release of radiopharmaceutical from [^{67}Ga][Ga(NO2A)]$^{+}$-M-Tyr(3-I)-Ahx-KuE at 37 ºC and pH 7.4.

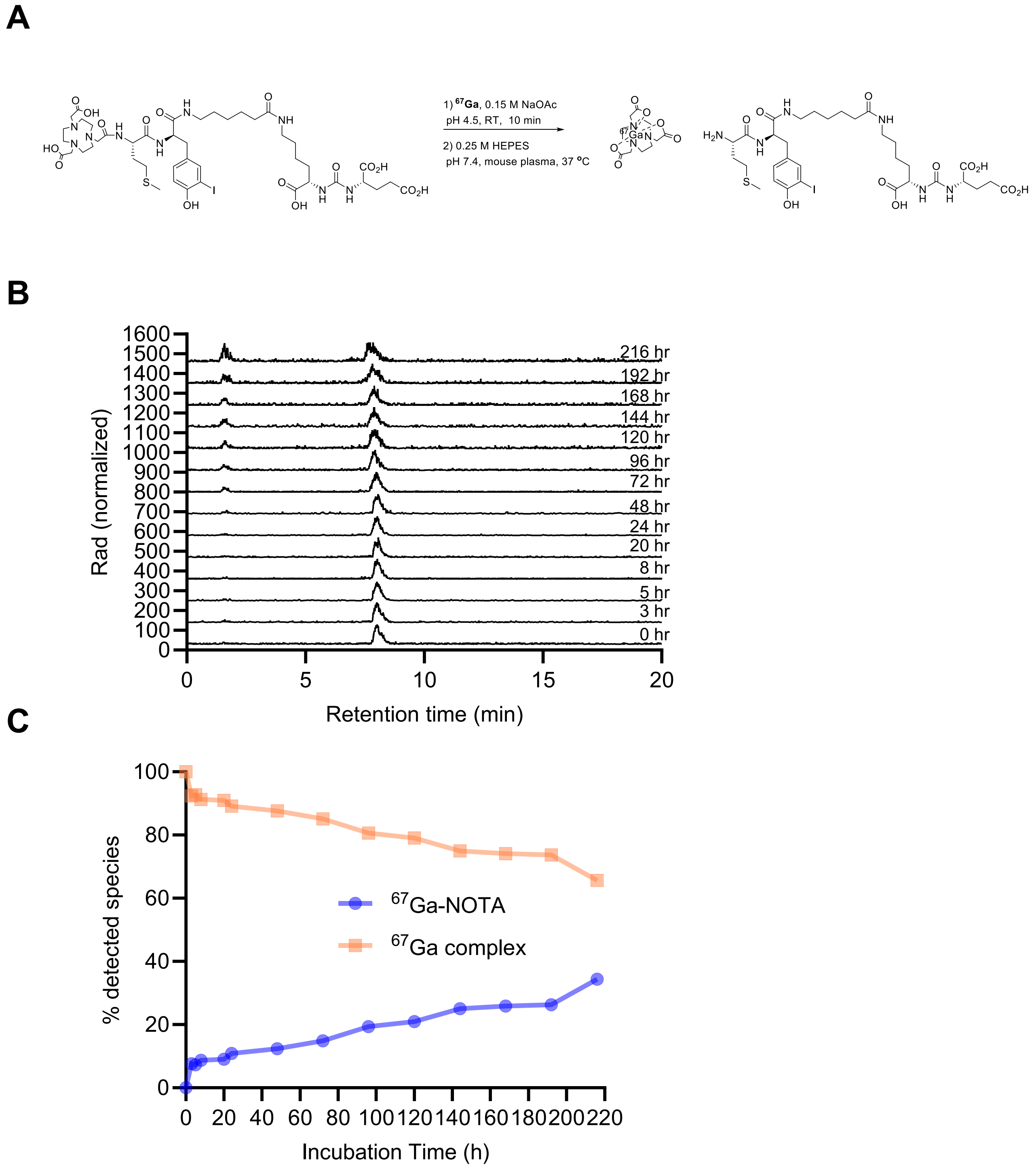

Figure S243. (A) Schematic description of preparation and direct radiolabeling procedure of **22**, NO2A-M-Tyr(3-I)-Ahx-KuE radiopharmaceutical. **(B)** Analytical radioHPLC chromatograms showing autolytic release of the desired product [^{67}Ga]Ga(NOTA). **(C)** Quantification of the temperature-dependent release of radiopharmaceutical from [^{67}Ga][Ga(NO2A)]$^{+}$-M-Tyr(3-I)-Ahx-KuE in mouse plasma at 37 ºC and pH 7.4.

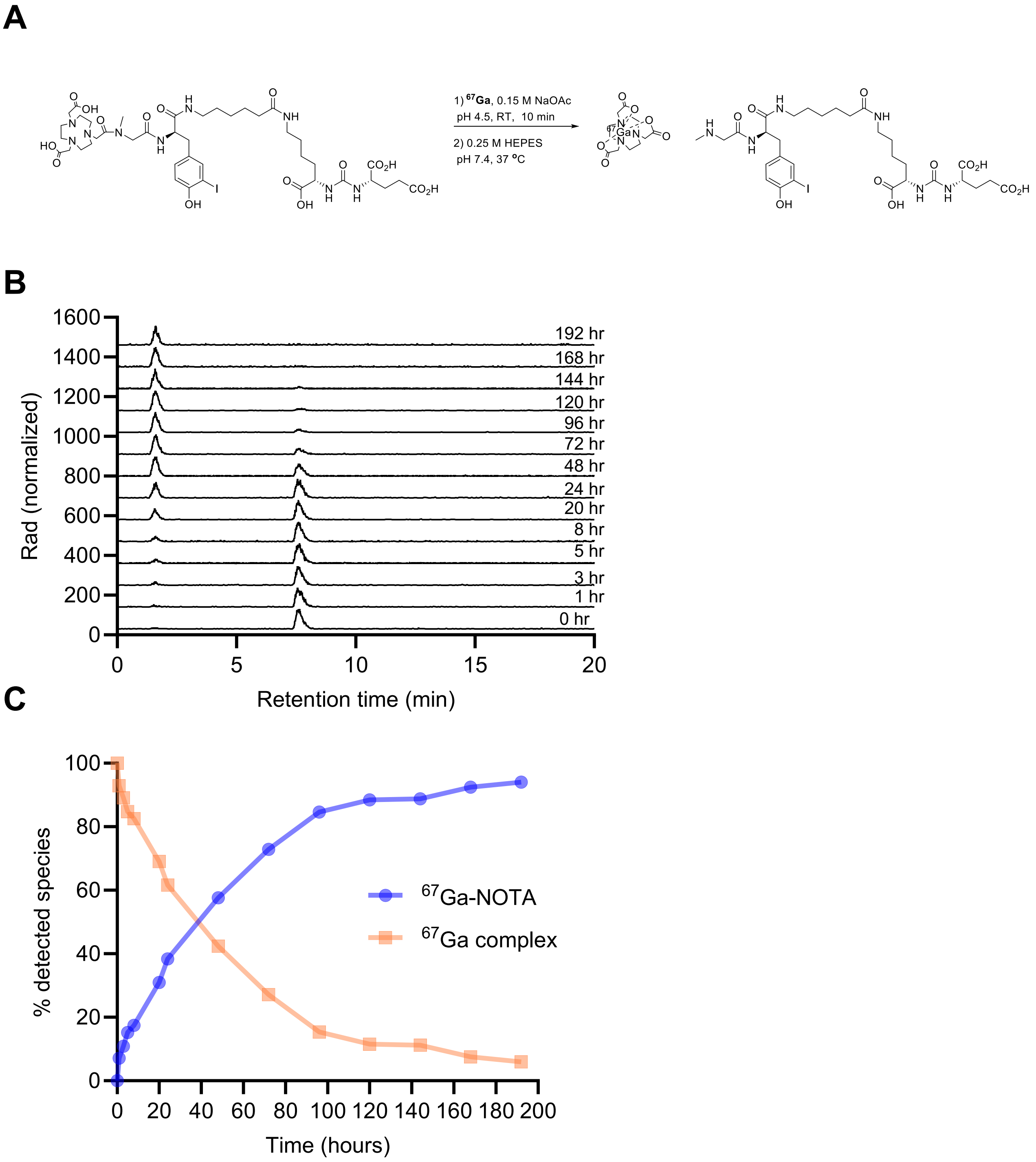

Figure S244. (A) Schematic description of preparation and direct radiolabeling procedure of **23**, NO2A-^{NMe}G-Tyr(3-I)-Ahx-KuE radiopharmaceutical. **(B)** Analytical radioHPLC chromatograms showing autolytic release of the desired product [^{67}Ga]Ga(NOTA). **(C)** Quantification of the temperature-dependent release of radiopharmaceutical from [^{67}Ga][Ga(NO2A)]$^{+}$-^{NMe}G-Tyr(3-I)-Ahx-KuE at 37 ºC and pH 7.4.

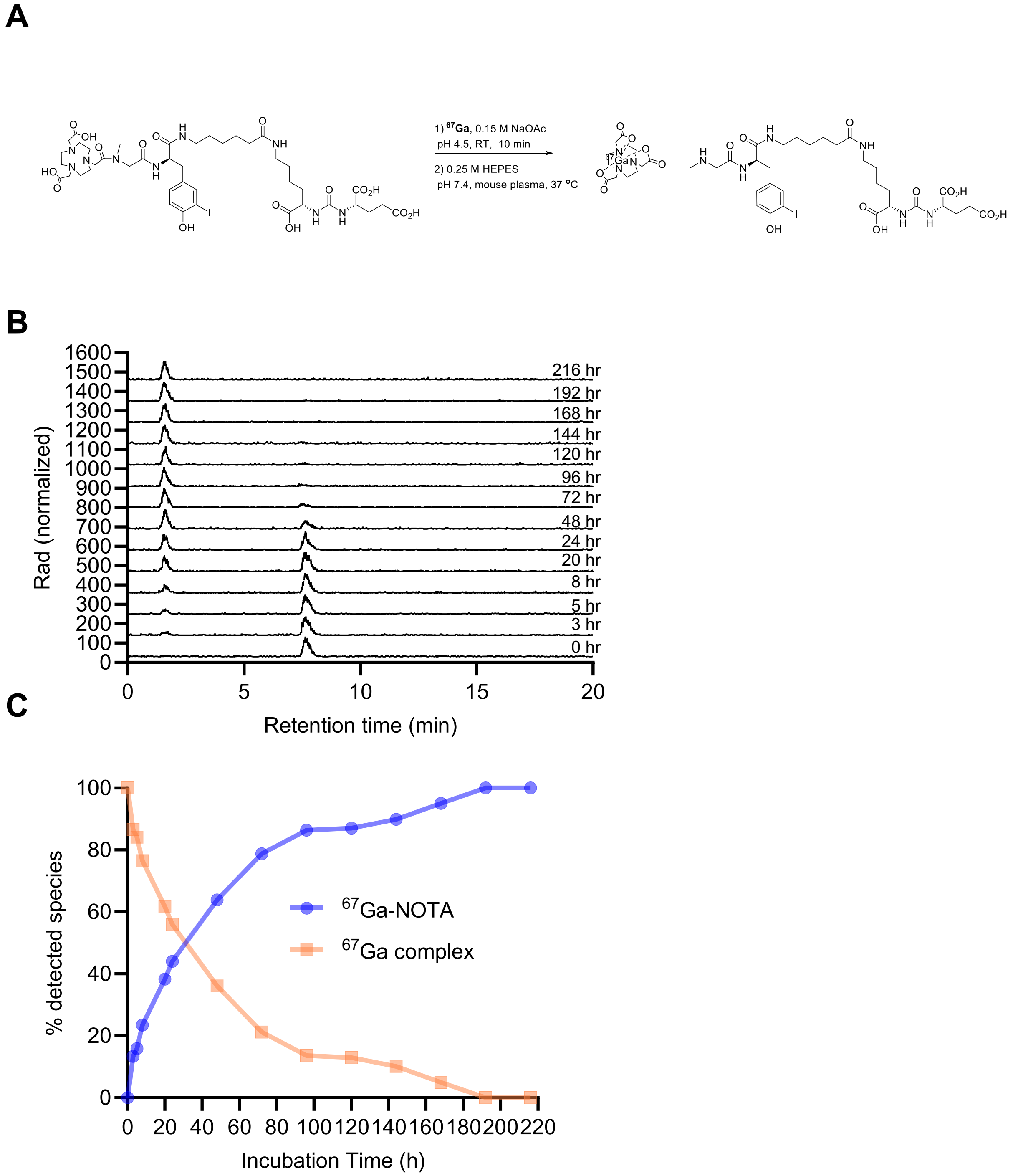

Figure S245. (A) Schematic description of preparation and direct radiolabeling procedure of **23**, NO2A-^{NMe}G-Tyr(3-I)-Ahx-KuE radiopharmaceutical. **(B)** Analytical radioHPLC chromatograms showing autolytic release of the desired product [^{67}Ga]Ga(NOTA). **(C)** Quantification of the temperature-dependent release of radiopharmaceutical from [^{67}Ga][Ga(NO2A)]$^{+}$-^{NMe}G-Tyr(3-I)-Ahx-KuE in mouse plasma at 37 °C and pH 7.4.

A

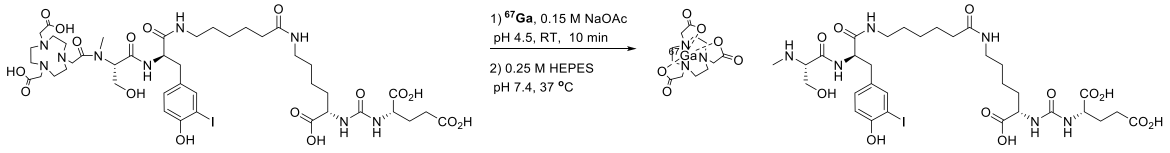

B

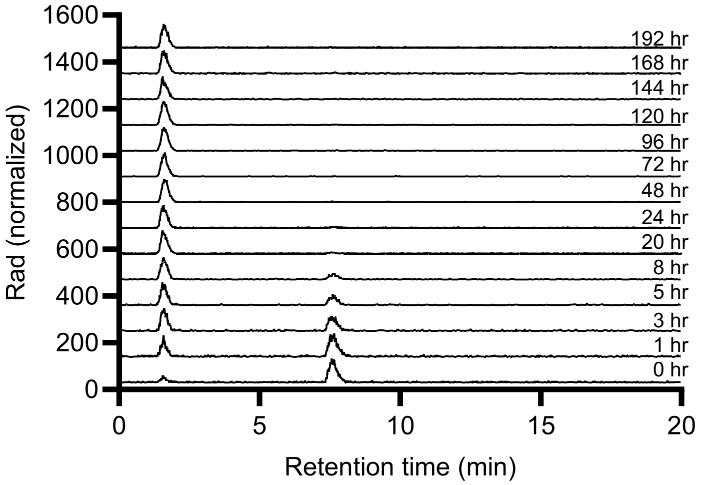

C

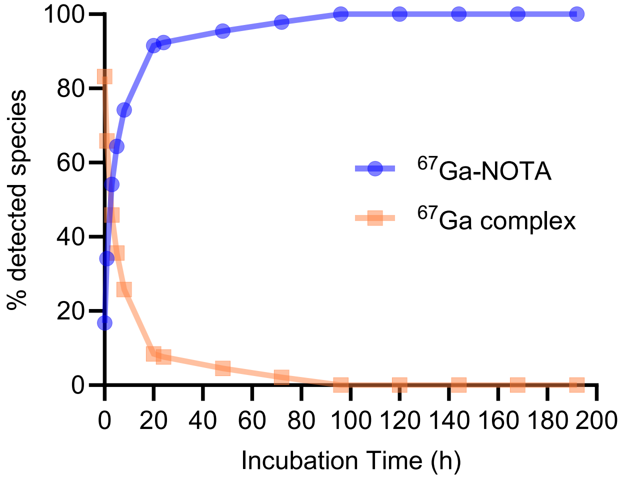

Figure S246. (A) Schematic description of preparation and direct radiolabeling procedure of **24**, NO2A-^{NMe}S-Tyr(3-I)-Ahx-KuE radiopharmaceutical. **(B)** Analytical radioHPLC chromatograms showing autolytic release of the desired product [^{68}Ga]Ga(NOTA). **(C)** Quantification of the temperature-dependent release of radiopharmaceutical from [^{68}Ga][Ga(NO2A)]$^{+}$-^{NMe}S-Tyr(3-I)-Ahx-KuE at 37 ºC and pH 7.4.

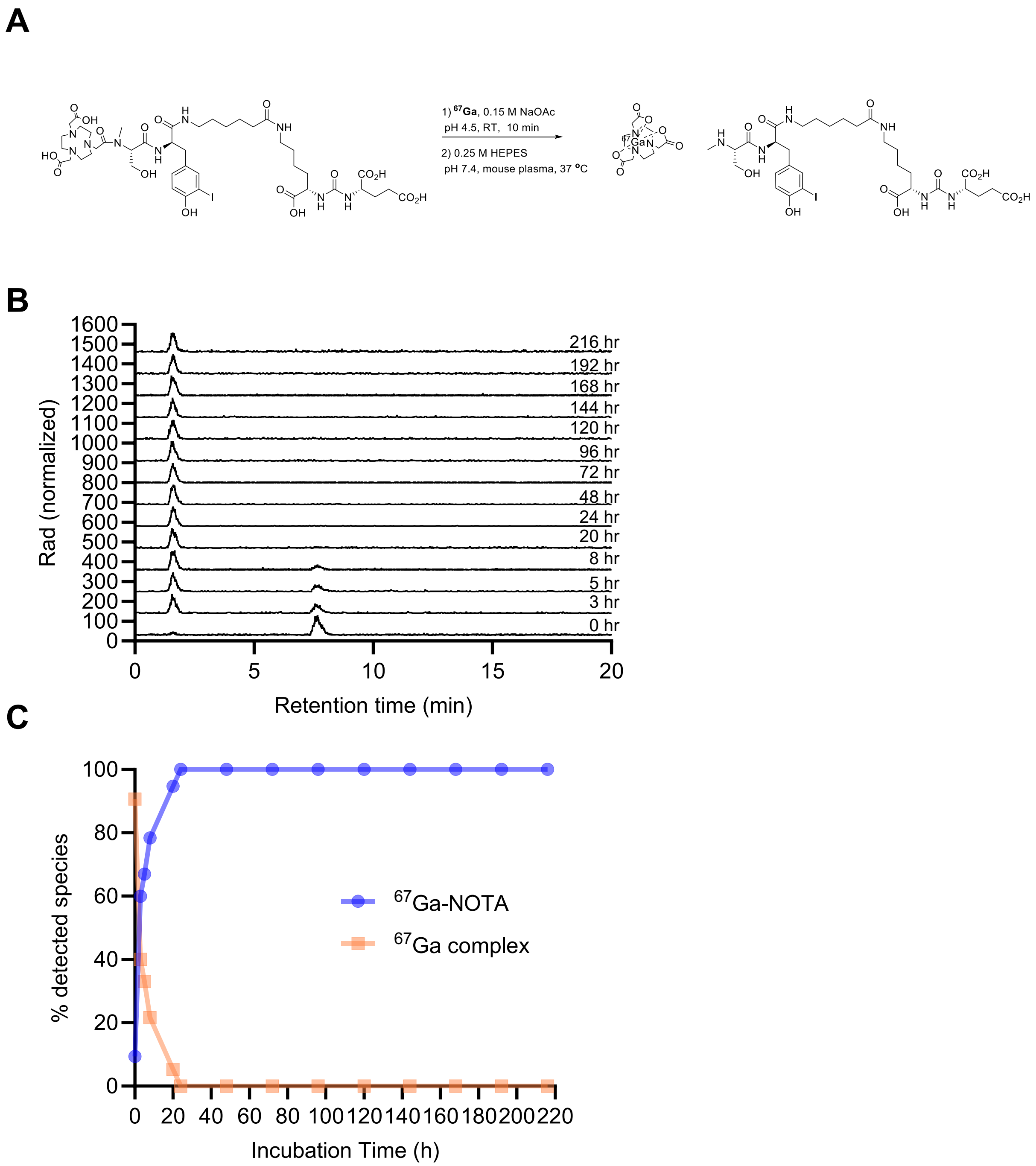

Figure S247. (A) Schematic description of preparation and direct radiolabeling procedure of **24**, NO2A-^{NMe}S-Tyr(3-I)-Ahx-KuE radiopharmaceutical. **(B)** Analytical radioHPLC chromatograms showing autolytic release of the desired product [^{68}Ga]Ga(NOTA). **(C)** Quantification of the temperature-dependent release of radiopharmaceutical from [^{68}Ga][Ga(NO2A)]$^{+}$-^{NMe}S-Tyr(3-I)-Ahx-KuE in mouse plasma at 37 °C and pH 7.4.

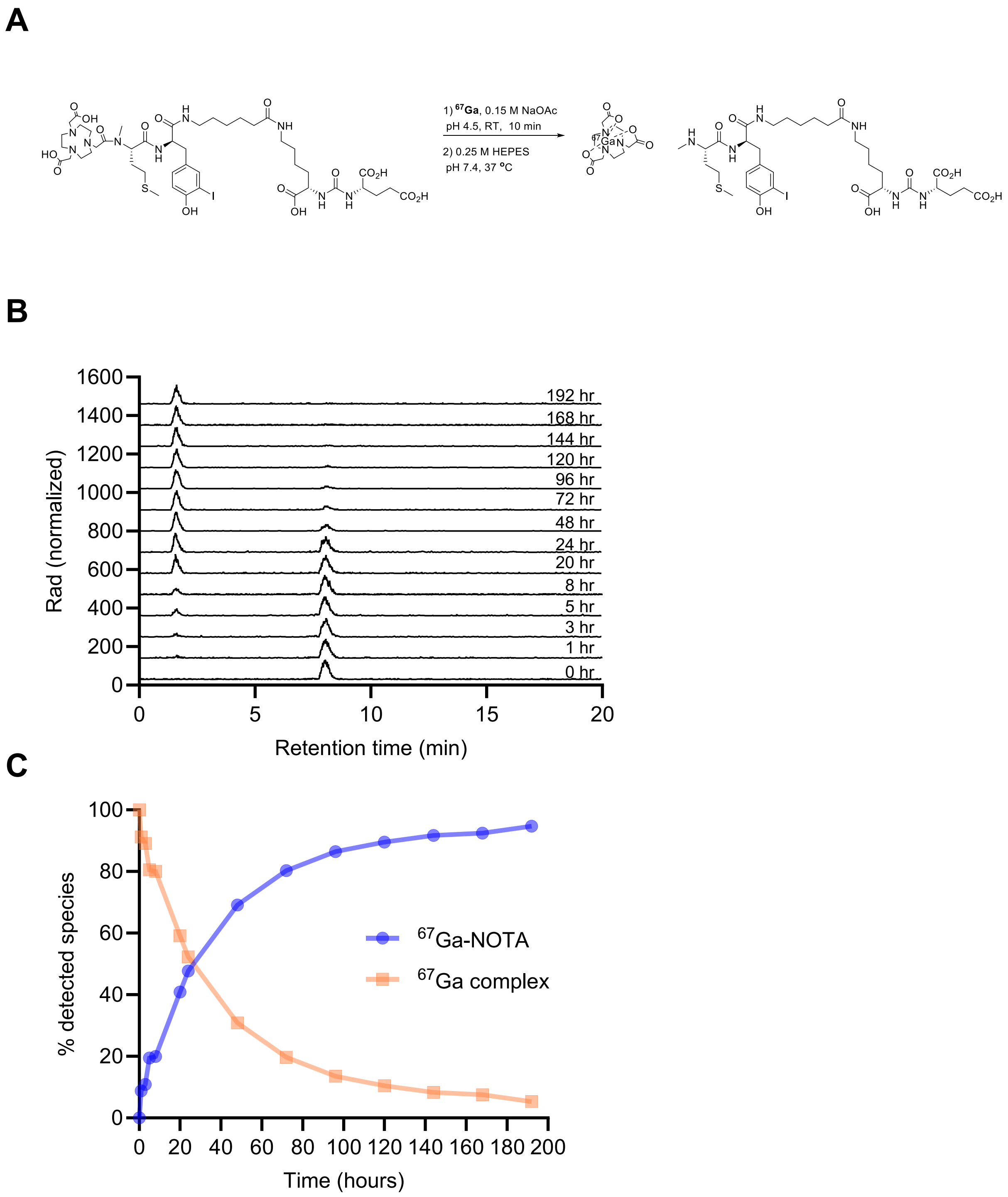

Figure S248. (A) Schematic description of preparation and direct radiolabeling procedure of **25**, NO2A-^{NMe}M-Tyr(3-I)-Ahx-KuE radiopharmaceutical. **(B)** Analytical radioHPLC chromatograms showing autolytic release of the desired product [^{68}Ga]Ga(NOTA). **(C)** Quantification of the temperature-dependent release of radiopharmaceutical from [^{68}Ga][Ga(NO2A)]$^{+}$-^{NMe}M-Tyr(3-I)-Ahx-KuE at 37 °C and pH 7.4.

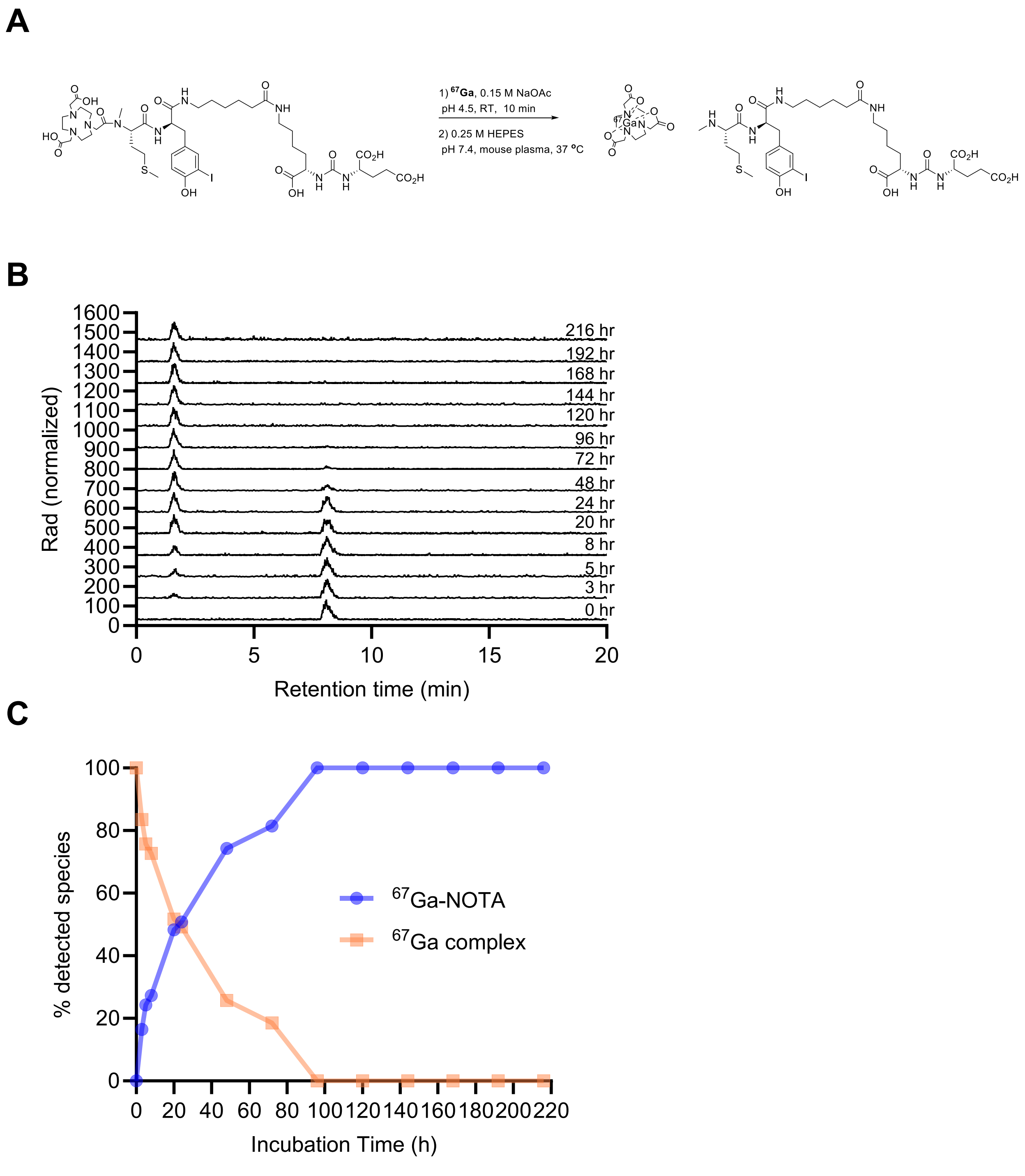

Figure S249. **(A)** Schematic description of preparation and direct radiolabeling procedure of **25**, NO2A-^{NMe}M-Tyr(3-I)-Ahx-KuE radiopharmaceutical. **(B)** Analytical radioHPLC chromatograms showing autolytic release of the desired product [^{68}Ga]Ga(NOTA). **(C)** Quantification of the temperature-dependent release of radiopharmaceutical from [^{68}Ga][Ga(NO2A)]$^{+}$-^{NMe}M-Tyr(3-I)-Ahx-KuE in mouse plasma at 37 °C and pH 7.4.

5 Mechanistic Studies

5.1 Metal Complex Speciation

NMR (^1H) spectroscopic titrations were performed on a Bruker Avance 400 instrument at 25 °C. Each NMR sample was prepared by adding TMSP-d$_4$ (10 µL, 10 µM) to 600 µL of the pH-adjusted sample in water. For metal-ligand complex speciation, pH-adjusted aliquots were prepared from stock solutions ([Ligand] = 0.1 mM; [Ga^{3+}] = 0.1 mM; 0.1 M KCl; 0.01 M HCl). A water suppression method was employed in the absence of the deuterated solvent. ^{1}H spectra were referenced to TMSP-d$_4$ (0.00 pm).

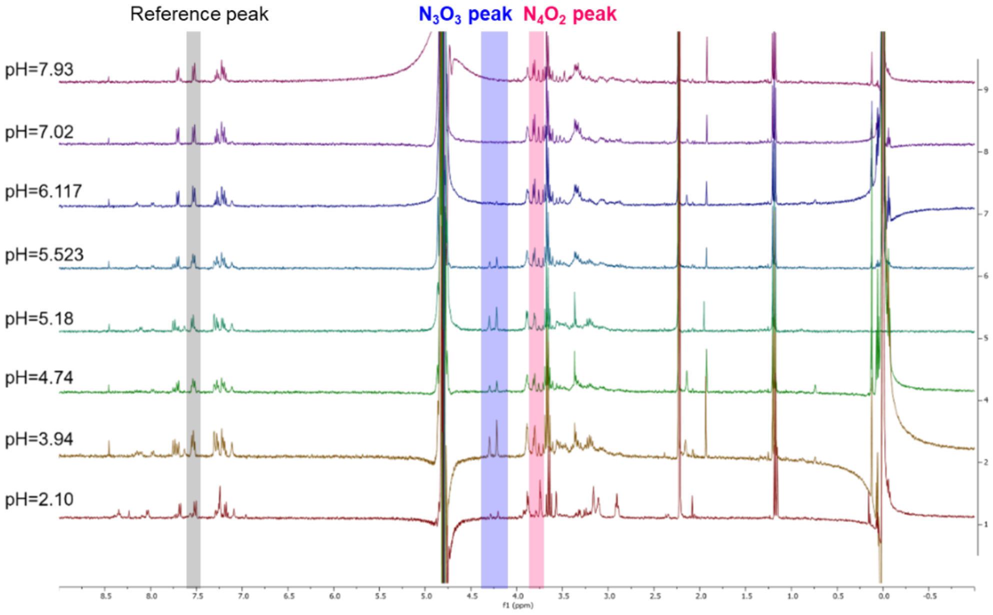

Figure S250. [Ga(NO2A)]$^+$-Gly-Gly-Trp (**[Ga(10)]**$^+$) ^{1}H NMR spectra as a function of pH in H_2O. [NO2A-Gly-Gly-Trp] = 0.1 mM, [Ga^{3+}] = 0.1 mM, [HCl] = 0.01 M, [KCl] = 0.1 M, T = 25 °C.

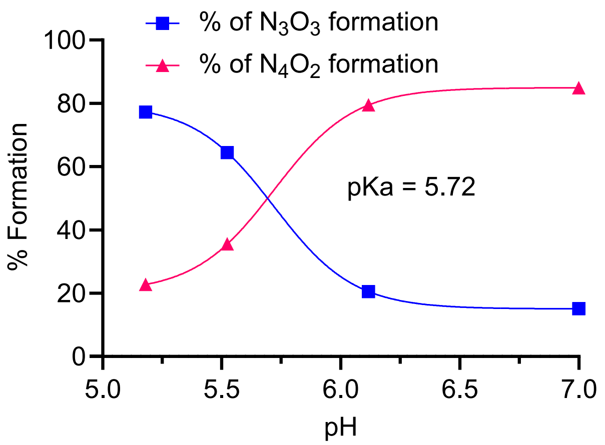

Figure S251. Speciation curves derived from pH-dependent NMR spectra showing the isomeric transformation between the N_3O_3 and N_4O_2 forms of [Ga(NO2A)]$^+$-Gly-Gly-Trp model tripeptide in H_2O.

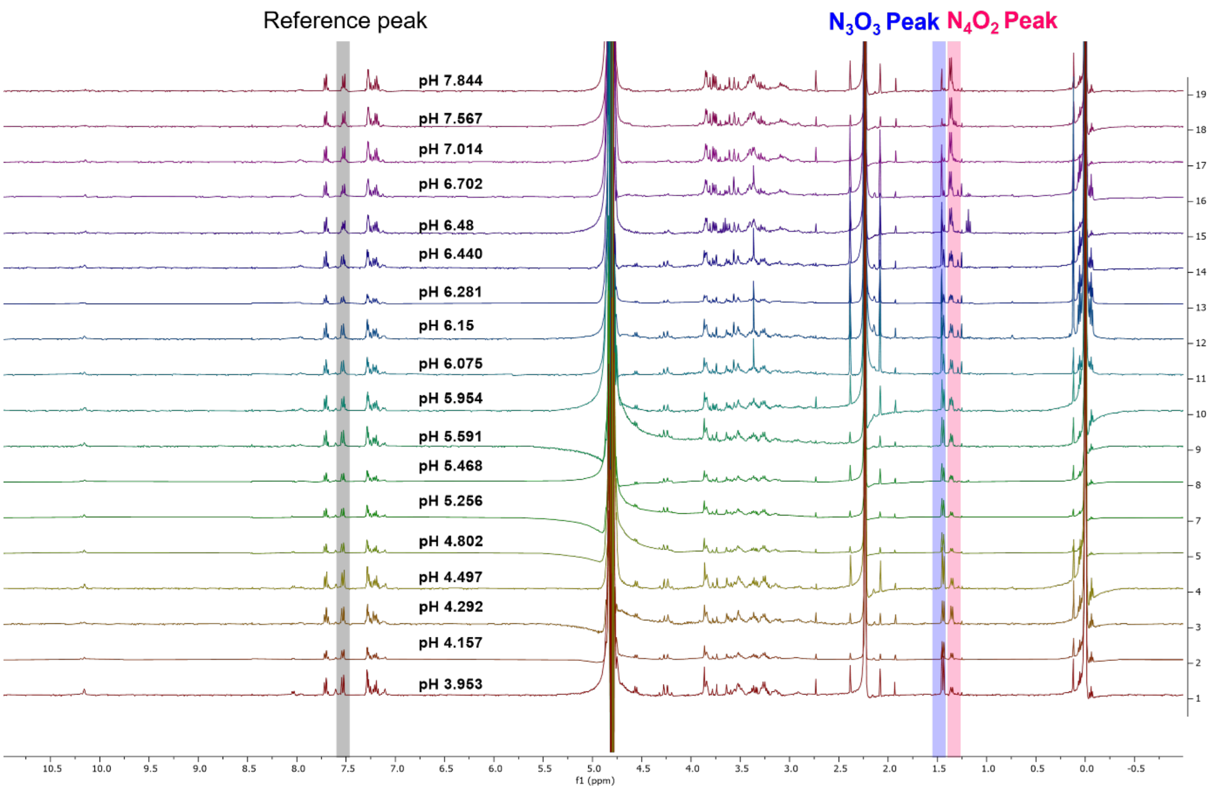

Figure S252. $[Ga(NO2A)]^+$-Ala-Gly-Trp (**[Ga(12)]**$^+$) ^{1}H NMR spectra as a function of pH in H_2O. [NO2A-Ala-Gly-Trp] = 0.1 mM, $[Ga^{3+}]$ = 0.1 mM, [HCl] = 0.01 M, [KCl] = 0.1 M, T = 25 °C.

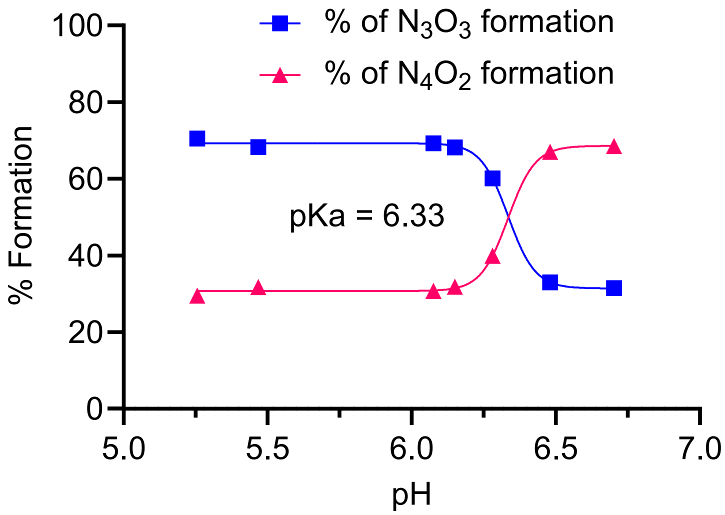

Figure S253. Speciation curves derived from pH-dependent NMR spectra showing the isomeric transformation between the N_3O_3 and N_4O_2 forms of $[Ga(NO2A)]^+$-Ala-Gly-Trp model tripeptide in H_2O.

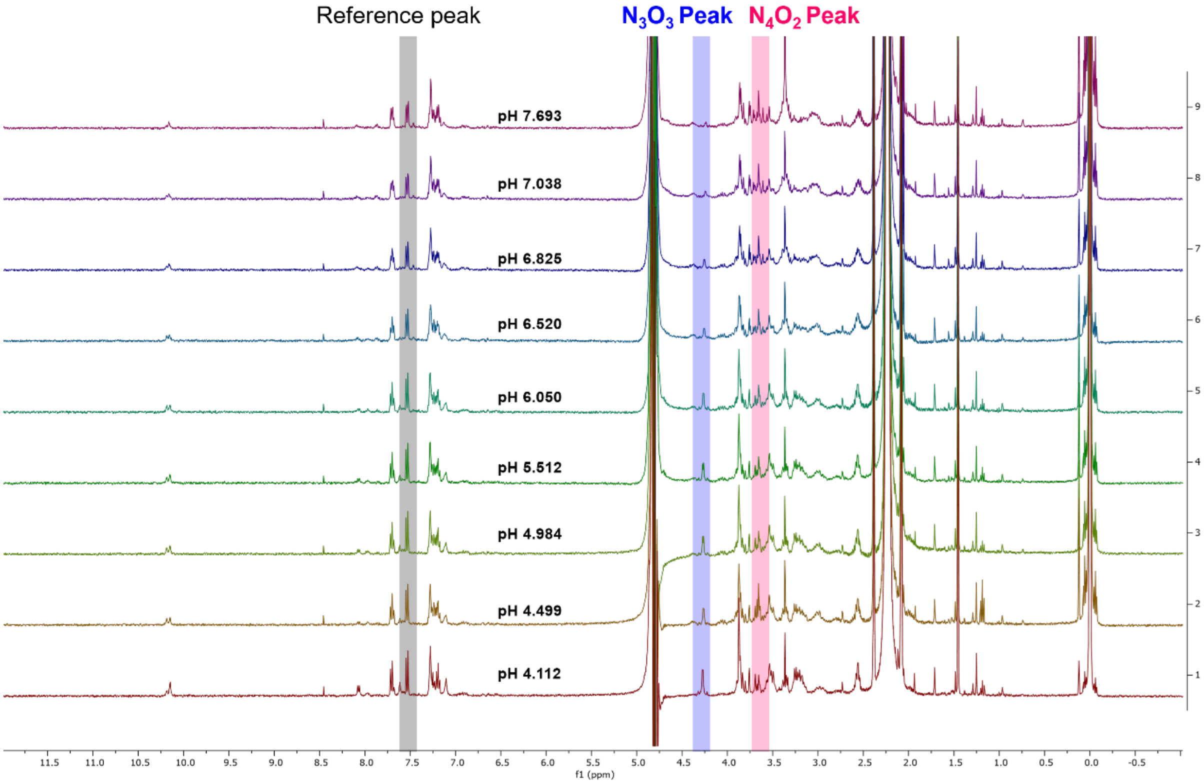

Figure S254. $[Ga(NO2A)]^+$-Met-Gly-Trp (**$[Ga(15)]^+$**) ^1H NMR spectra as a function of pH in H_2O. [NO2A-Met-Gly-Trp] = 0.1 mM, $[Ga^{3+}]$ = 0.1 mM, [HCl] = 0.01 M, [KCl] = 0.1 M, T = 25 °C.

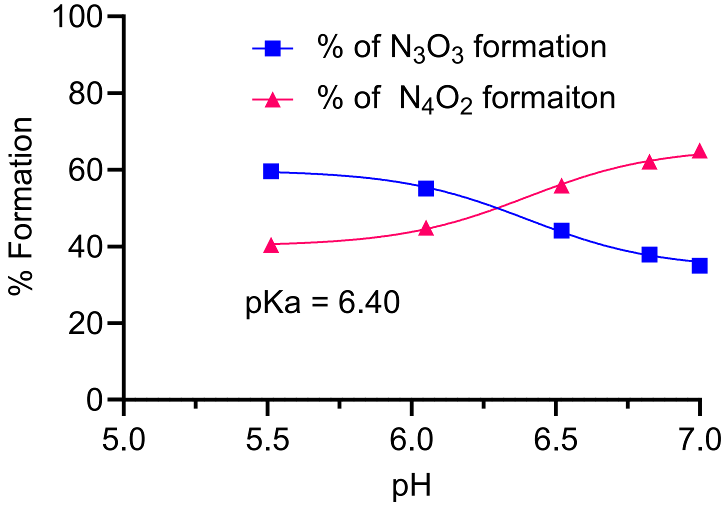

Figure S255. Speciation curves derived from pH-dependent NMR spectra showing the isomeric transformation between the N_3O_3 and N_4O_2 forms of $[Ga(NO2A)]^+$-Met-Gly-Trp model tripeptide in H_2O.

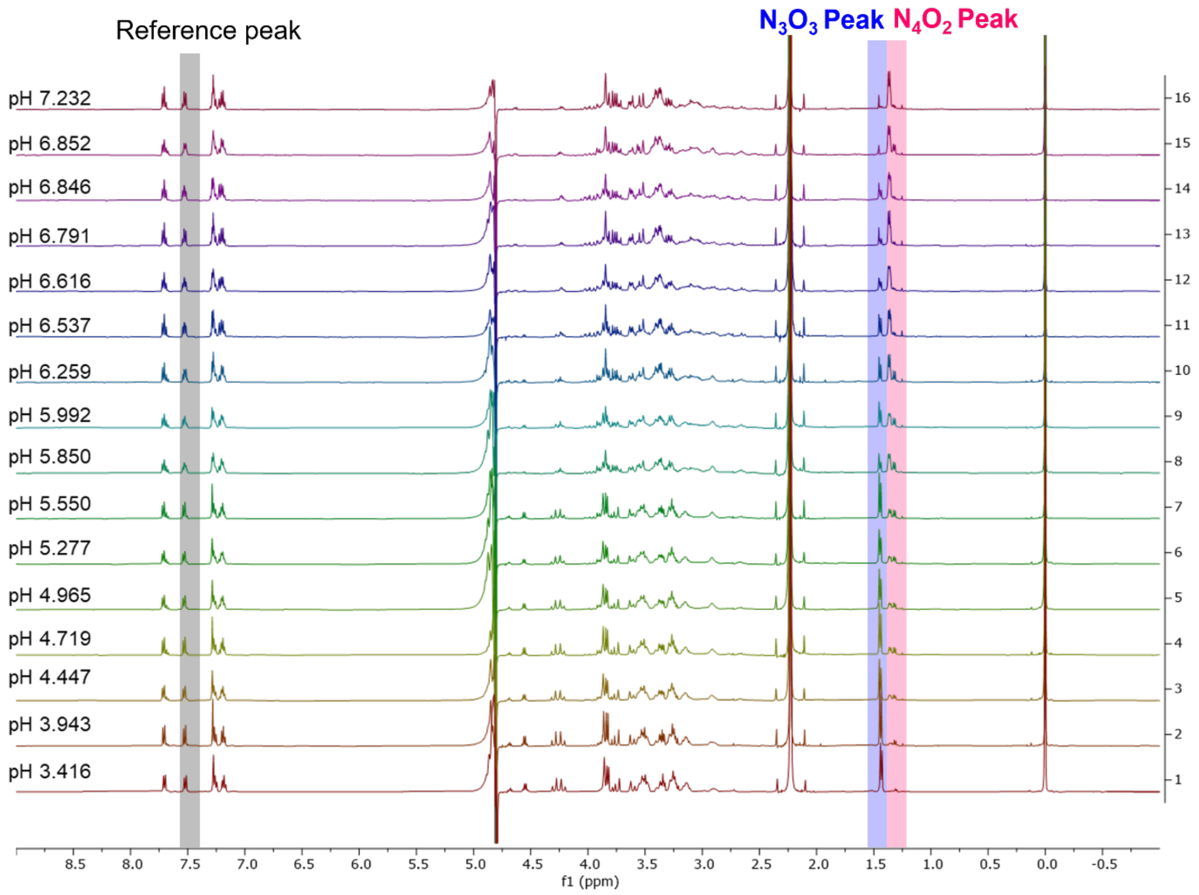

Figure S256. $[Ga(NO2A)]^+$-Ala-Gly-Trp (**$[Ga(12)]^+$**) ^1H NMR spectra as a function of pH in D_2O. [NO2A-Ala-Gly-Trp] = 0.1 mM, $[Ga^{3+}]$ = 0.1 mM, [HCl] = 0.01 M, [KCl] = 0.1 M, T = 25 °C.

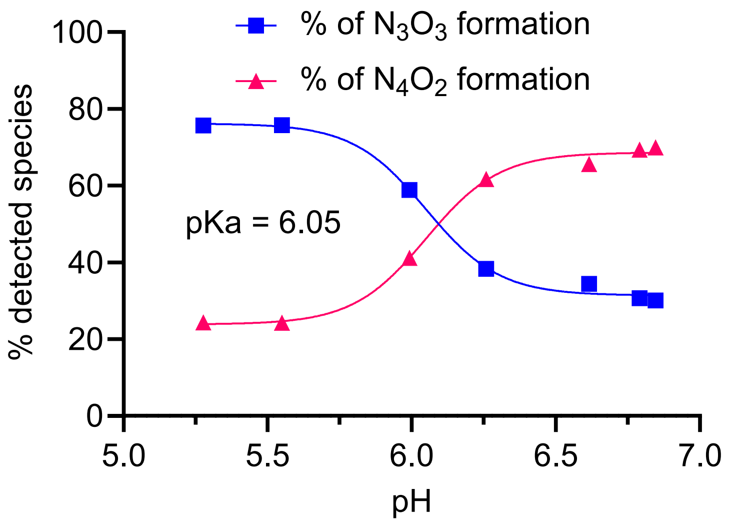

Figure S257. Speciation curves derived from pH-dependent NMR spectra showing the isomeric transformation between the N_3O_3 and N_4O_2 forms of $[Ga(NO2A)]^+$-Ala-Gly-Trp model tripeptide in D_2O.

5.2 H_2O and $H_2{}^{18}O$ Cleavage Experiments

[Ga(13)]$^+$ cleavage assay with regular H_2O analysis. To determine whether amide bond cleavage occurs via direct hydrolysis by exogenous nucleophiles, approximately 50 mM **[Ga(13)]$^+$** complex solution was buffered to pH 7.4 using 0.1 M HEPES buffer, and cleavage experiment was performed in regular H_2O. Solutions were incubated at 80 °C until the cleavage product formation was confirmed by HPLC-MS. HR-ESI-MS analysis was subsequently performed.

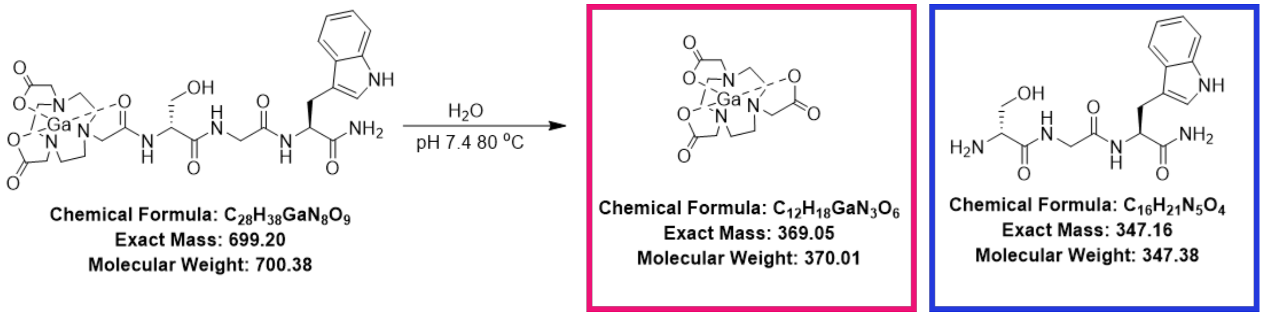

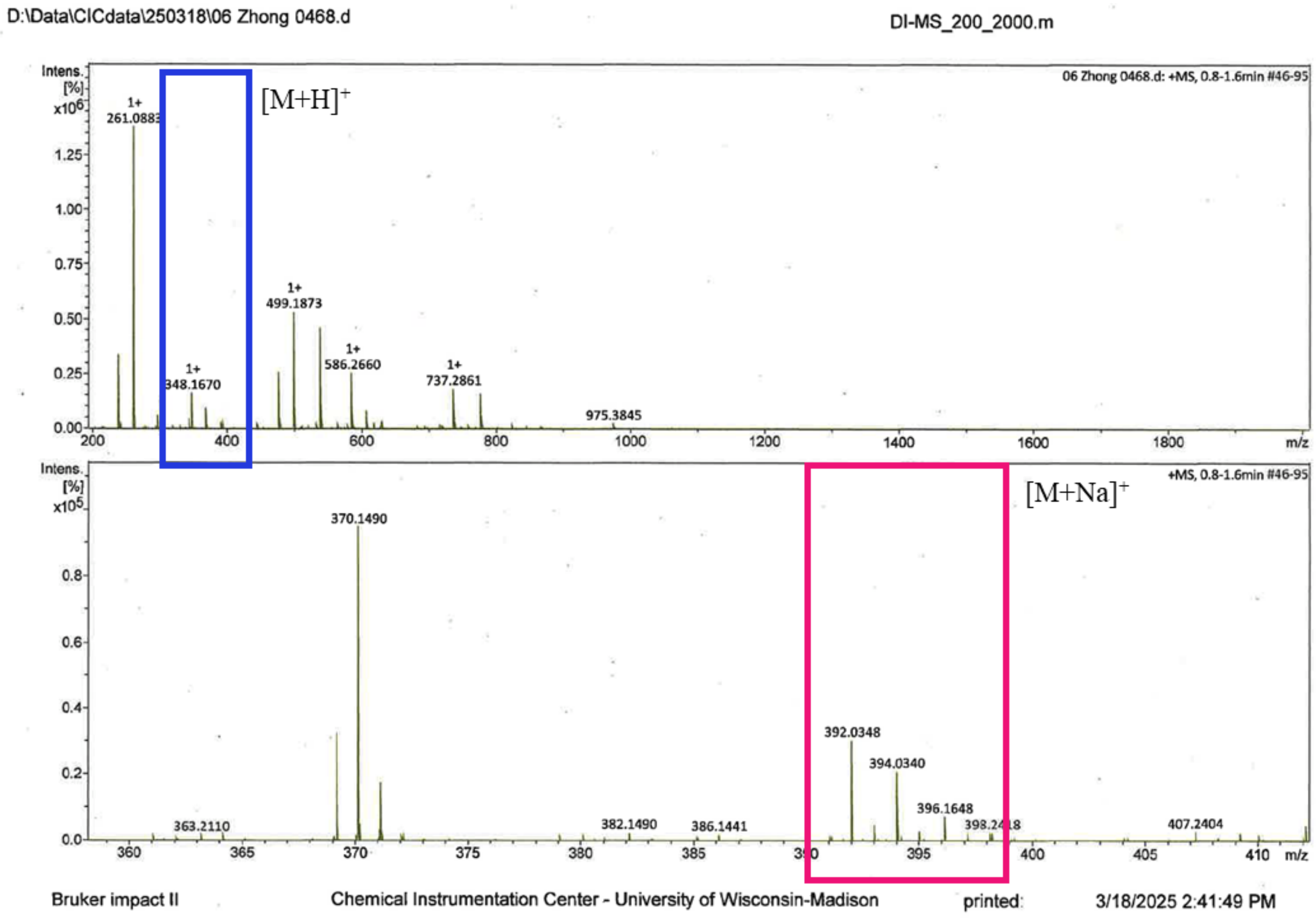

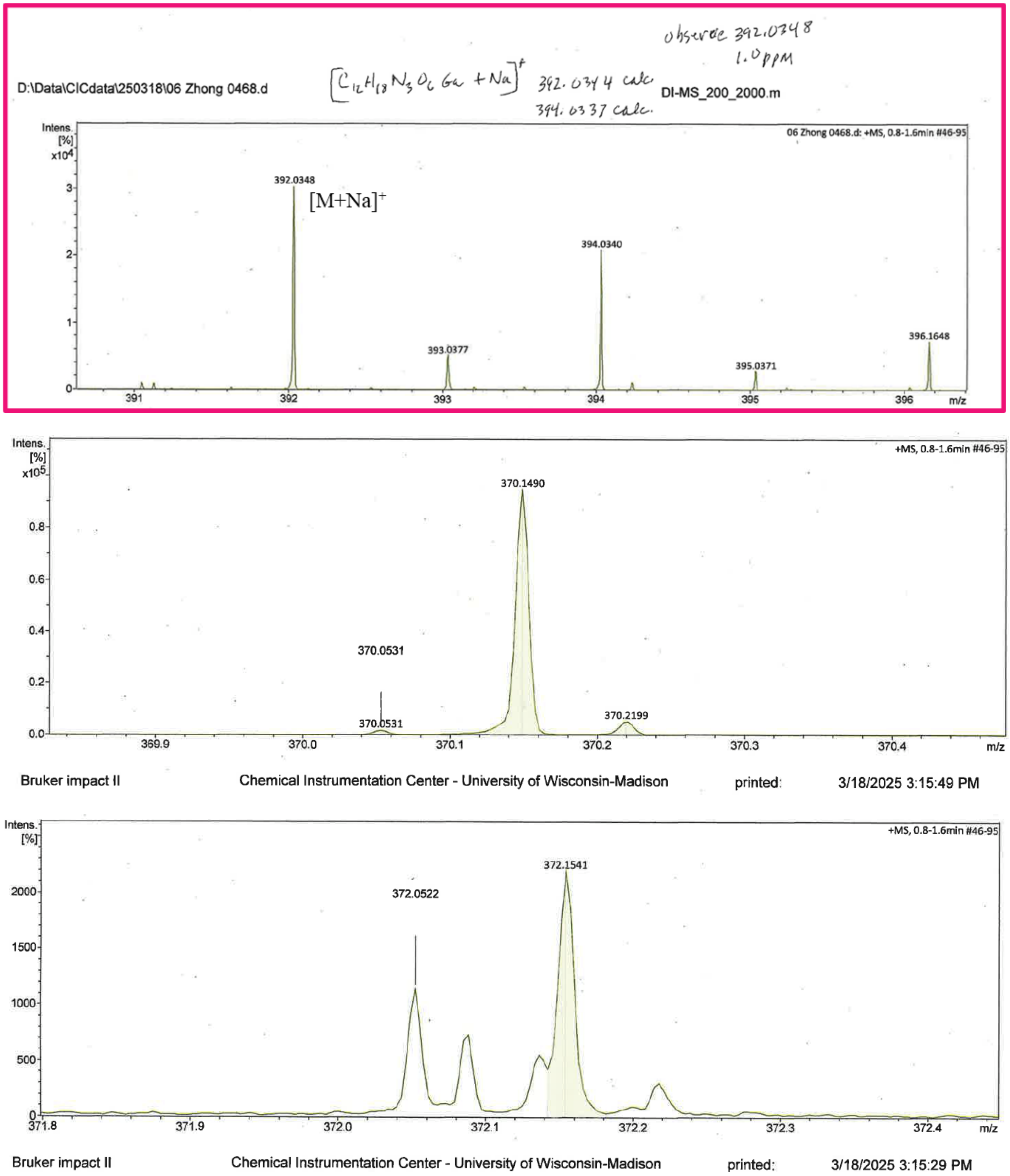

Figure S258. HR-ESI-MS of cleavage product of **[Ga(11)]$^+$**. HR-ESI-MS [M+Na]$^+$ calc. for [$C_{12}H_{18}N_3O_6Ga$+Na]$^+$ 392.0344 and 394.0337 , found 392.0348 and 394.0340 at 1.0 ppm resolution with ^{nat}Ga isotopic pattern.

[Ga(17)]$^+$ cleavage assay with regular H_2O analysis. To determine whether amide bond cleavage occurs via direct hydrolysis by exogenous nucleophiles, approximately 50 mM **[Ga(17)]$^+$** complex solution was buffered to pH 7.4 using 0.1 M HEPES buffer, and cleavage experiment was performed in regular H_2O. Solutions were incubated at 80 ºC until the cleavage product formation was confirmed by HPLC-MS. ESI-HRMS analysis was subsequently performed.

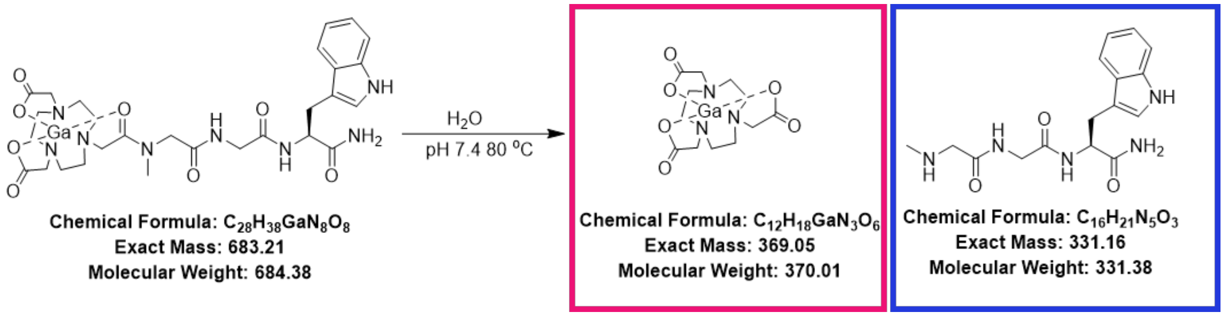

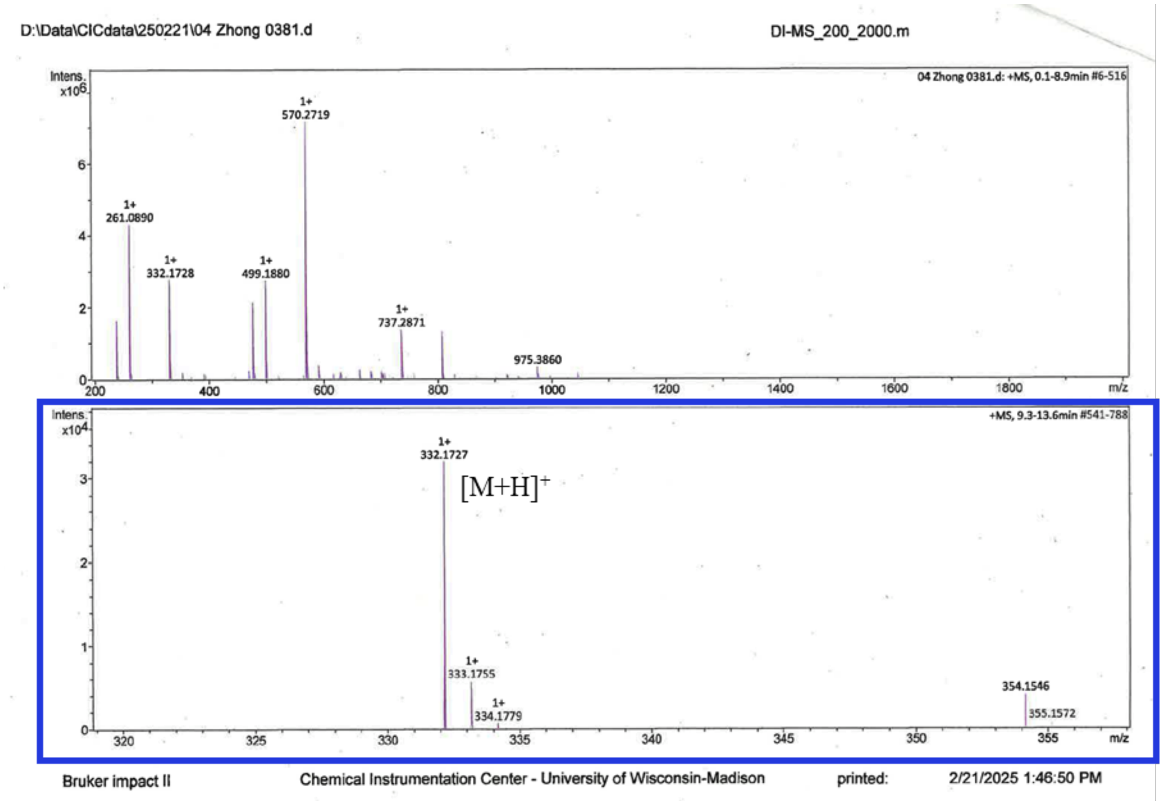

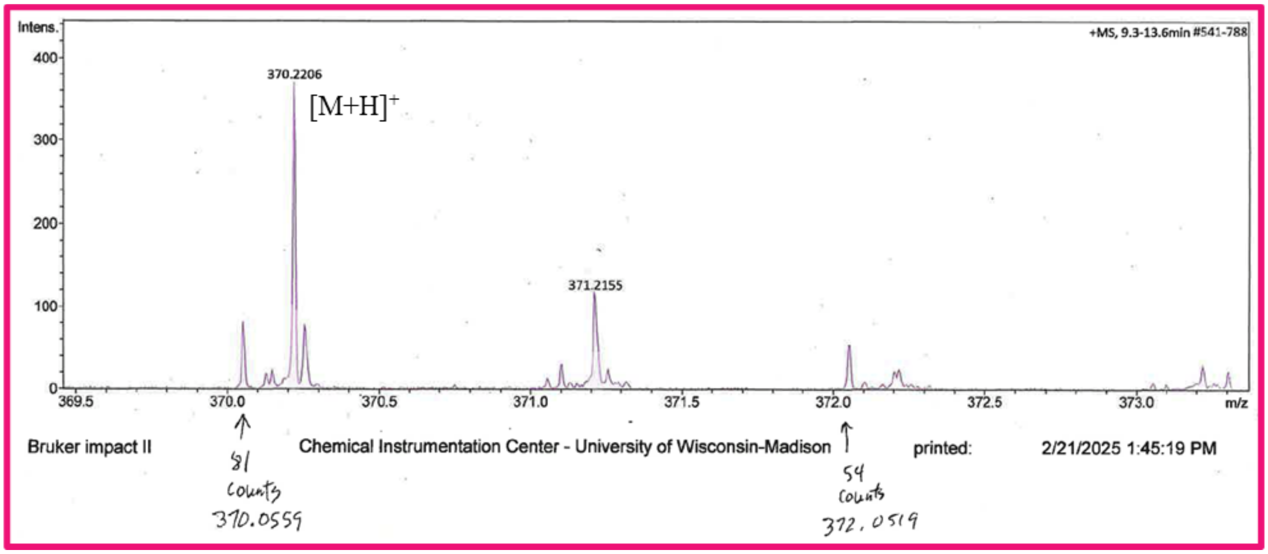

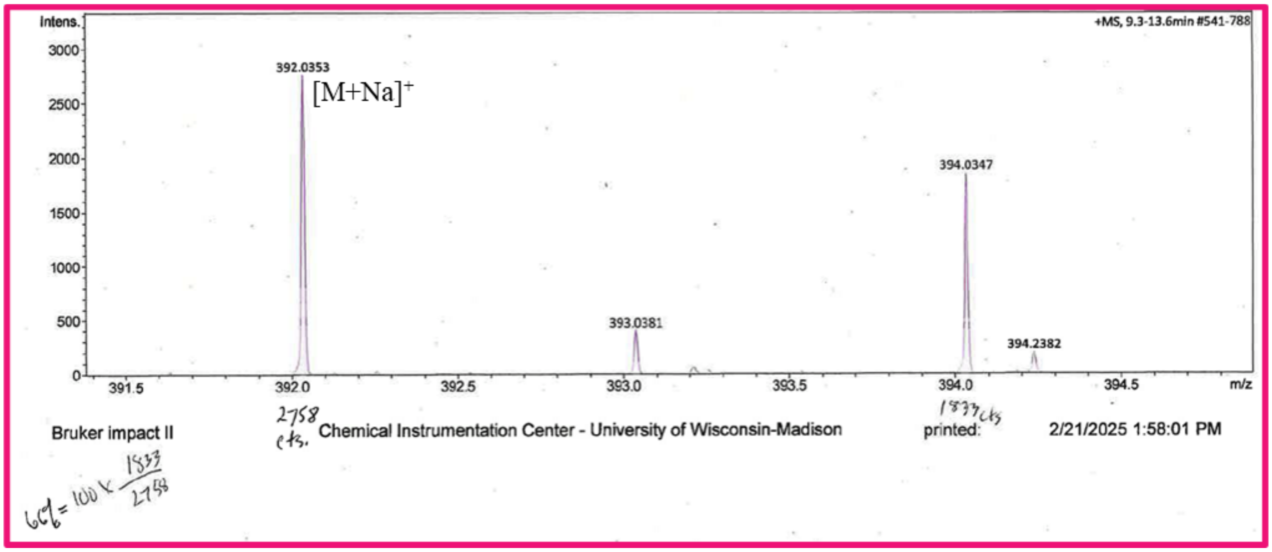

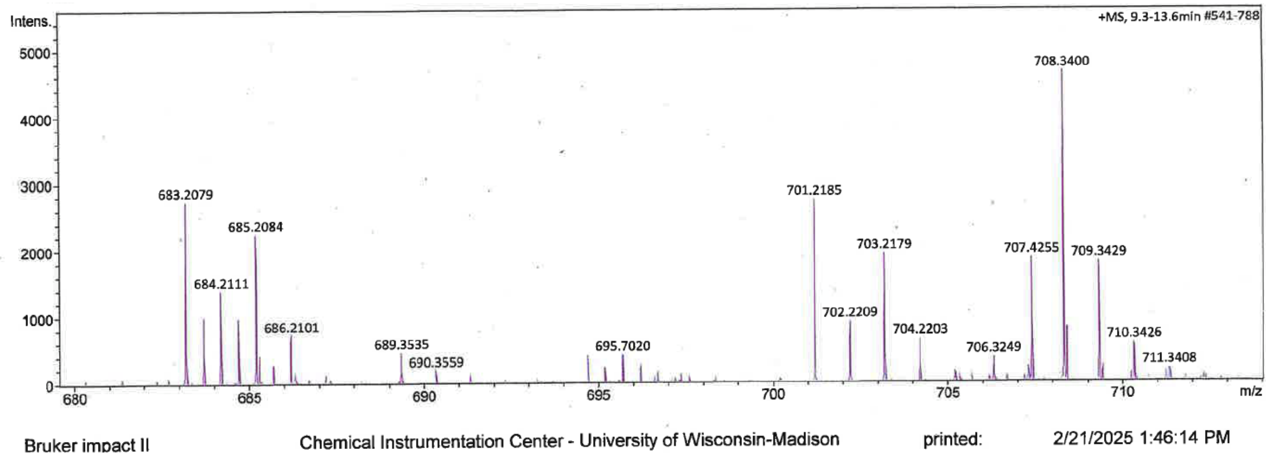

Figure S259. HR-ESI-MS of cleavage product of **[Ga(17)]$^{+}$**. HR-ESI-MS $[C_{12}H_{18}N_3O_6Ga+Na]^{+}$ found 392.0353 and 394.0347 with ^{nat}Ga isotopic pattern.

[Ga(17)]$^+$ cleavage assay with $H_2{}^{18}O$ analysis. To determine whether amide bond cleavage occurs via direct hydrolysis by exogenous nucleophiles, approximately 50 mM **[Ga(17)]$^+$** complex solution was buffered to pH 7.4 using 0.1 M HEPES buffer, and cleavage experiment was performed in $H_2{}^{18}O$ (97% atomic purity). Solutions were incubated at 80 °C until the cleavage product formation was confirmed by HPLC-MS. ESI-HRMS analysis was subsequently performed.

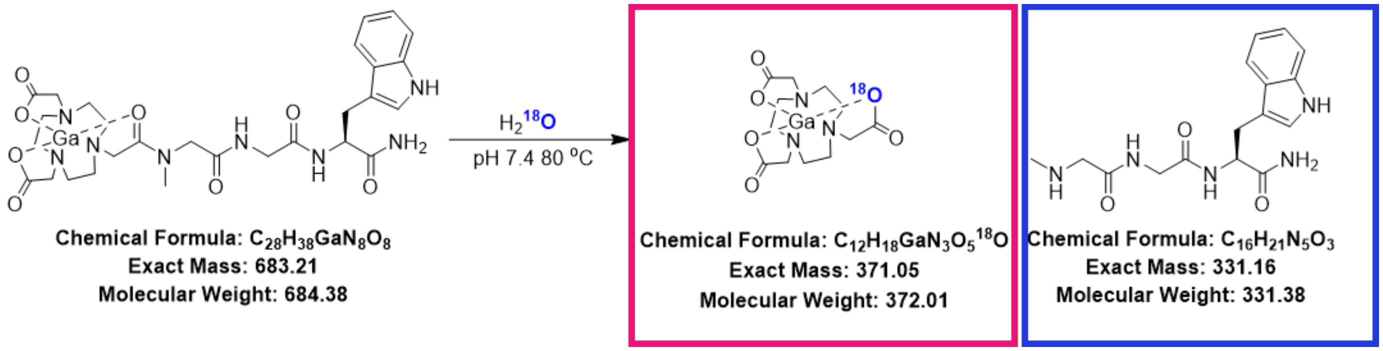

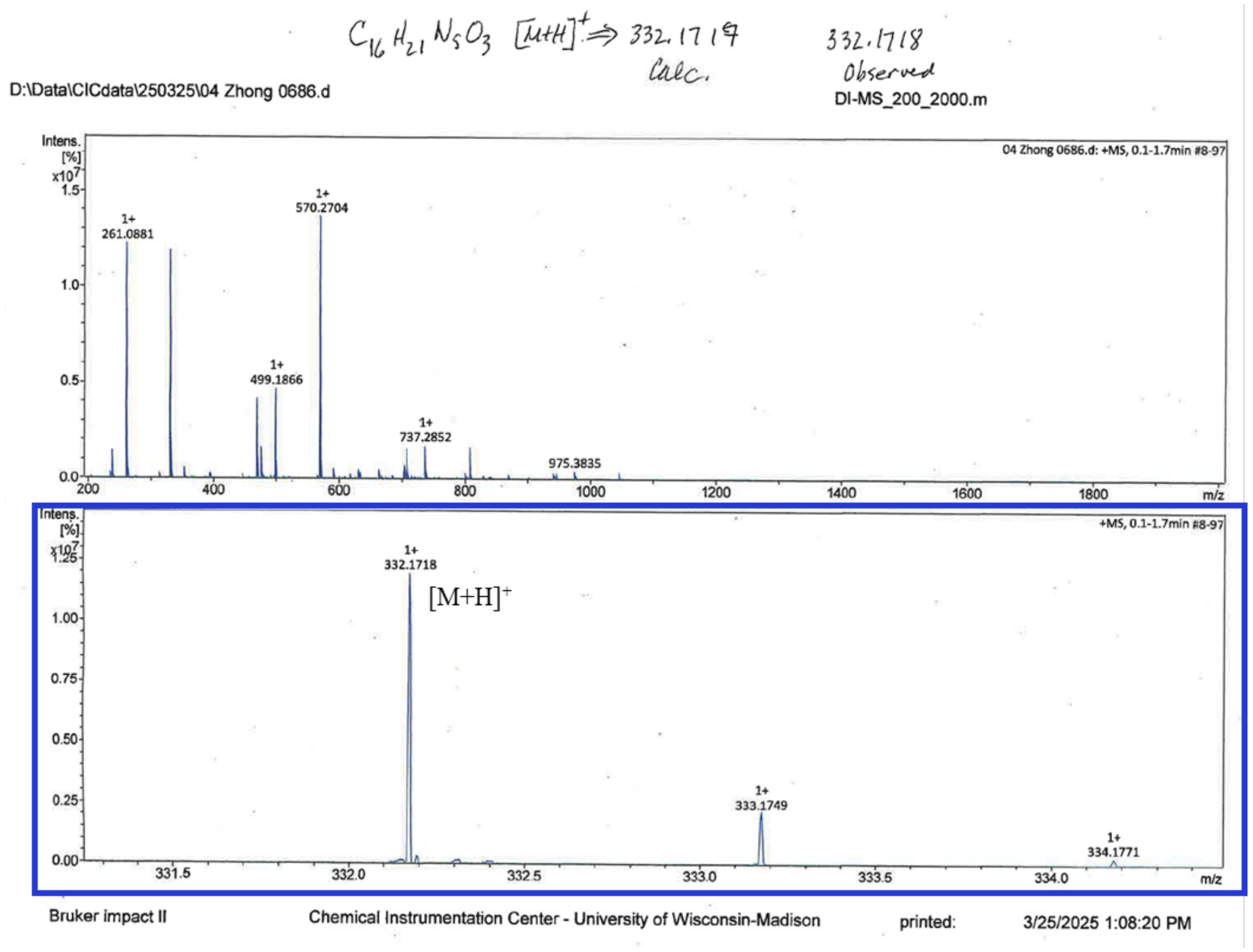

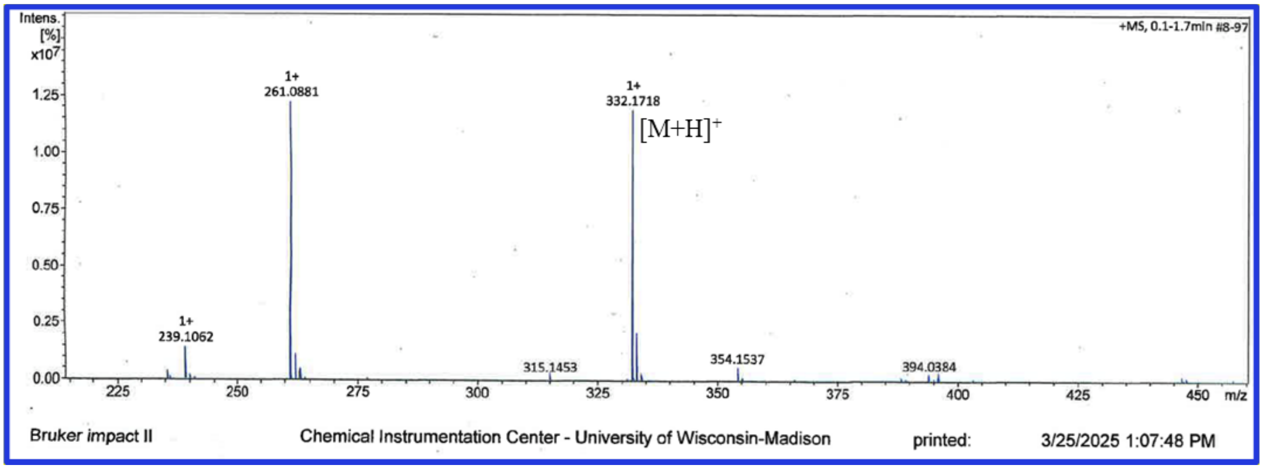

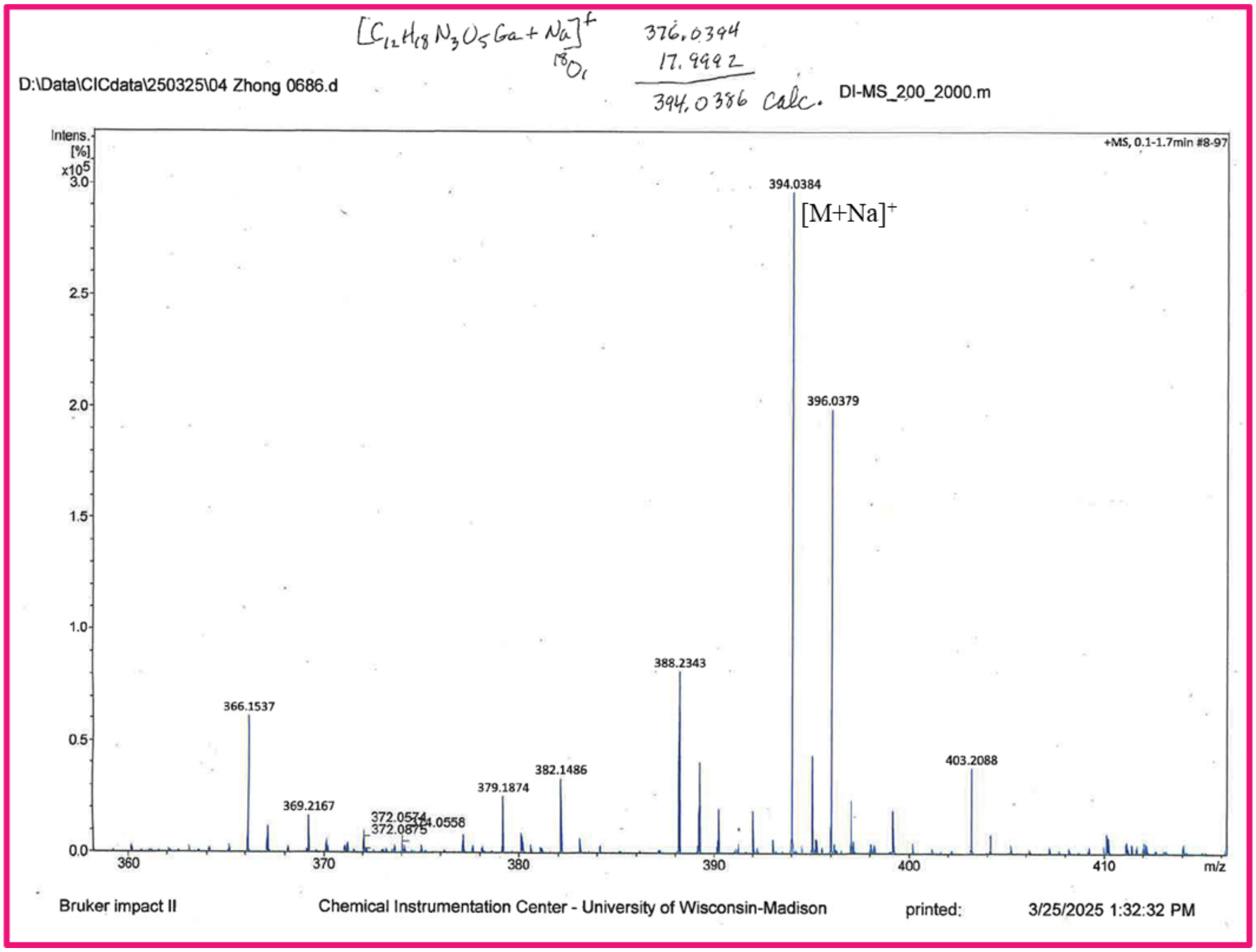

Figure S260. HR-ESI-MS of cleavage product of **[Ga(17)]**$^+$. HR-ESI-MS $[M+Na]^+$ calc. for $[C_{12}H_{18}N_3O_5{}^{18}O_1Ga+Na]^+$ 394.0386, found 394.0384 with ^{nat}Ga isotopic pattern.

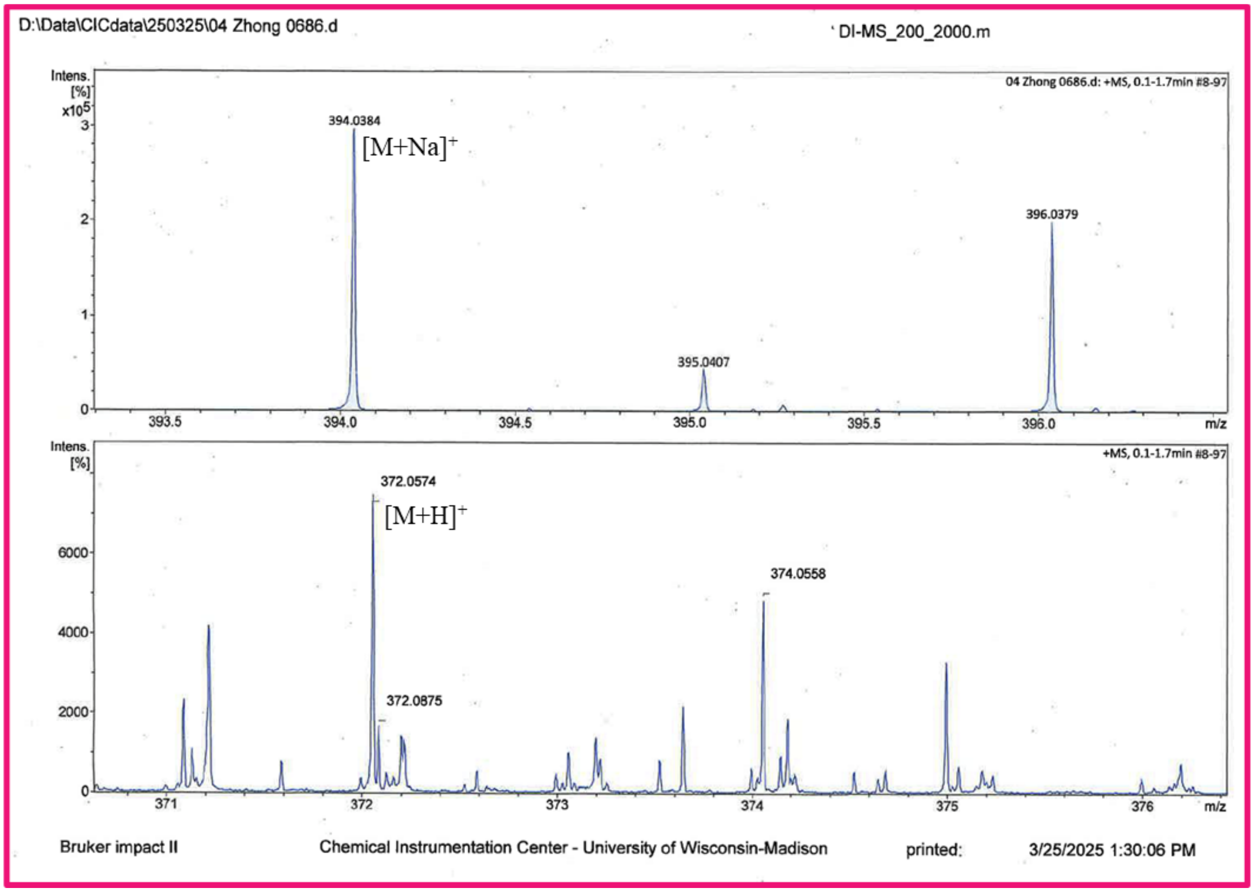

Figure S261. Zoomed-in view ESI-HRMS of cleavage product of **[Ga(17)]**$^+$. HR-ESI-MS $[M+Na]^+$ calc. for $[C_{12}H_{18}N_3O_5{}^{18}O_1Ga+Na]^+$ 394.0386, found 394.0384 with ^{nat}Ga isotopic pattern.

5.3 Cleavage assays in H_2O and D_2O for kinetic isotopic effect measurement

Cold cleavage assays of **[Ga(15)]**$^+$ were conducted in H_2O and D_2O to investigate the kinetic isotopic effects. In an Eppendorf tube, Ga complexes (0.75 mM) were initially prepared in H_2O according to general complexation protocol **Section 3.1**. The solvent was lyophilized, and the complexes were redissolved in D_2O. To minimize residual H_2O, the D_2O was lyophilized and redissolved three times. The Ga complexes were then redissolved in D_2O to a final concentration of 0.75 mM. The solution was buffered to pH 7.4 by adding pH 7.4 HEPES buffer. Cleavage reactions were performed at 80 °C and monitored by analytical HPLC.

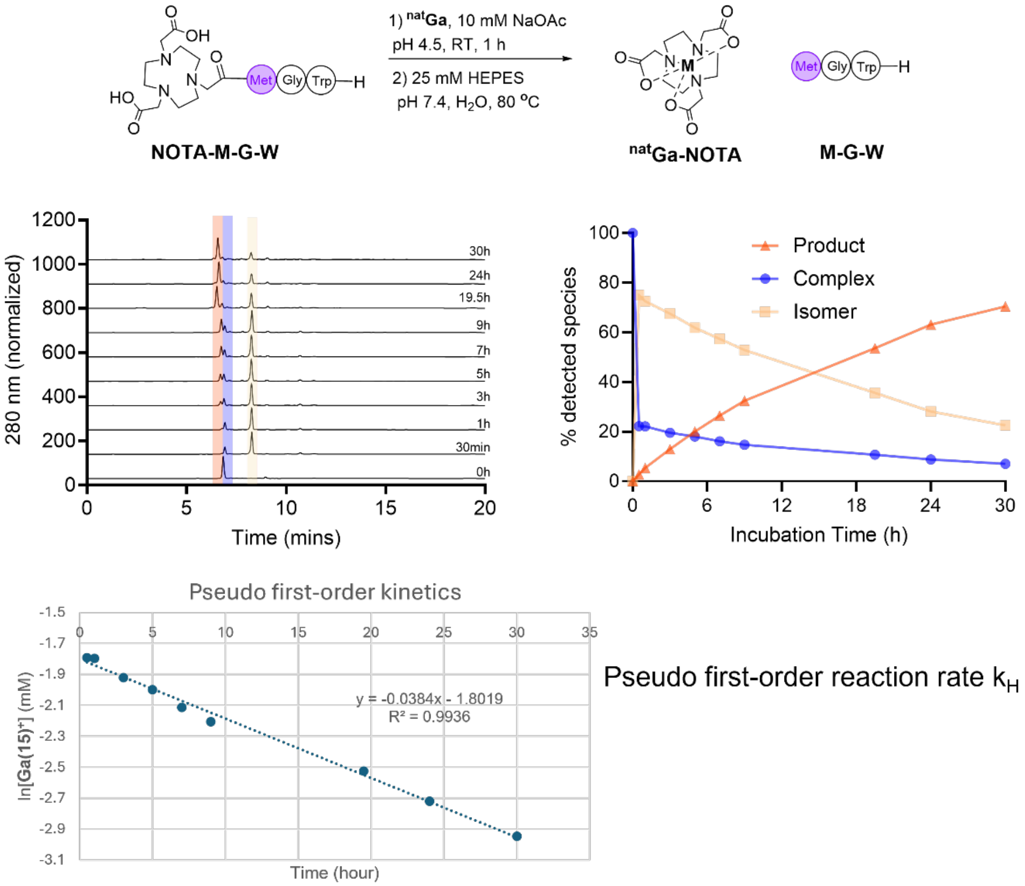

Pseudo first-order reaction rate k_H = 0.038 hour^{-1}

Figure S262. Schematic description of preparation and direct complexation procedure of **[^{nat}Ga(15)]**$^+$. Analytical HPLC chromatograms showing autolytic release of the product, ^{nat}Ga-NOTA, in H_2O. The kinetics data was fit using $Y_t = -kt+Y_0$ in Excel, where Y_t represents the ln([concentration of intact species]) in mM, k represents the pseudo first-order reaction rate in hour^{-1}, t represents the incubation time in hour, and Y_0 presents the ln([initial concentration of intact species]) in mM.

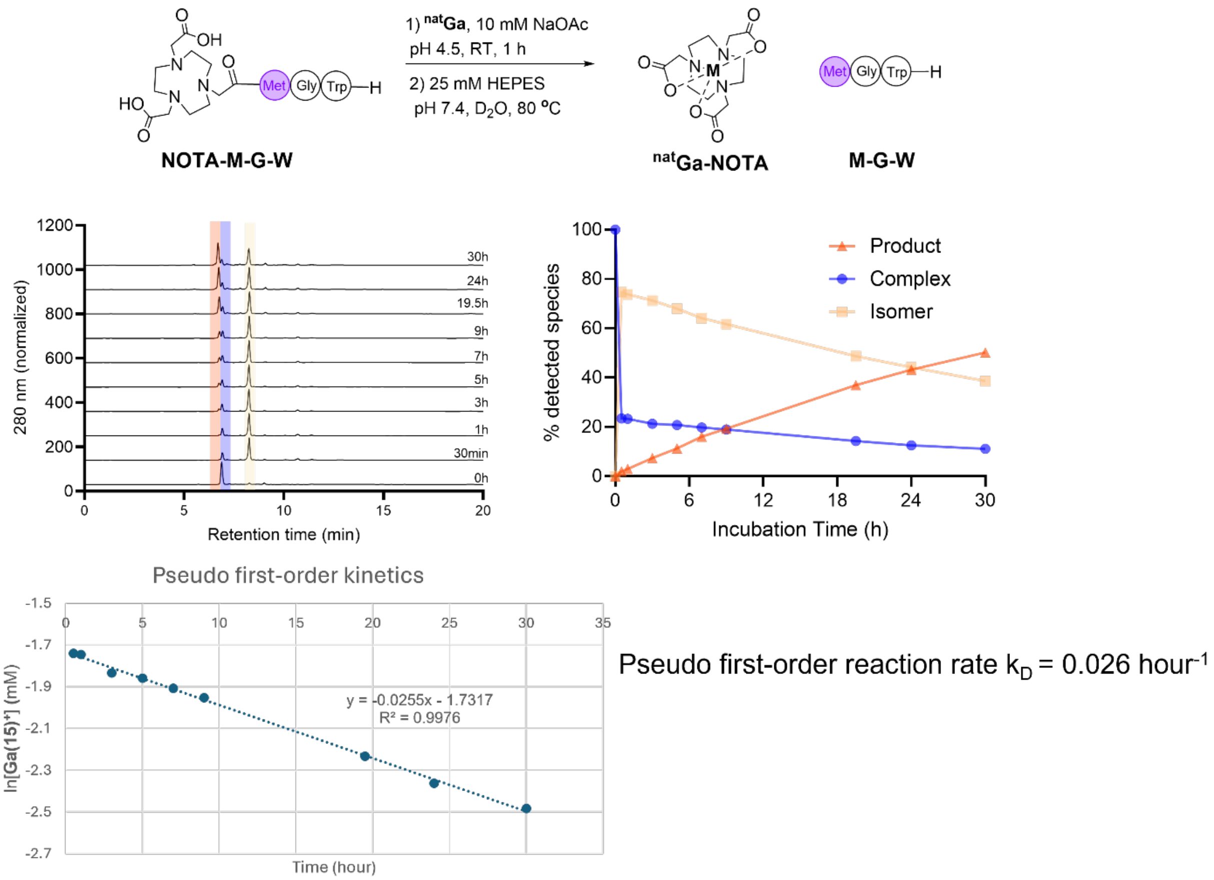

KIE = k_D/k_H = 0.038/0.026 = 1.46

Figure S263. Schematic description of preparation and direct complexation procedure of **[^{nat}Ga(15)]**$^+$. Analytical HPLC chromatograms showing autolytic release of the product, ^{nat}Ga-NOTA, in D_2O. The kinetics data was fit using $Y_t = -kt+Y_0$ in Excel, where Y_t represents the ln([concentration of intact species]) in mM, k represents the pseudo first-order reaction rate in hour^{-1}, t represents the incubation time in hour, and Y_0 presents the ln([initial concentration of intact species]) in mM. The kinetic isotopic effect can be calculated by the equation KIE = k_L/k_H, where k_L refers to the reaction rate involving the lighter isotope (H_2O), and k_H refers to the reaction rate involving the heavier isotope (D_2O).

Cold cleavage assays of **[Ga(19)]**$^{+}$ were conducted in H_2O and D_2O to investigate the kinetic isotopic effects. In an Eppendorf tube, Ga complexes (0.75 mM) were initially prepared in H_2O according to general complexation protocol **Section 3.1**. The solvent was lyophilized, and the complexes were redissolved in D_2O. To minimize residual H_2O, the D_2O was lyophilized and redissolved three times. The Ga complexes were then redissolved in D_2O to a final concentration of 0.75 mM. The solution was buffered to pH 7.4 by adding pH 7.4 HEPES buffer. Cleavage reactions were performed at 80 °C and monitored by analytical HPLC.

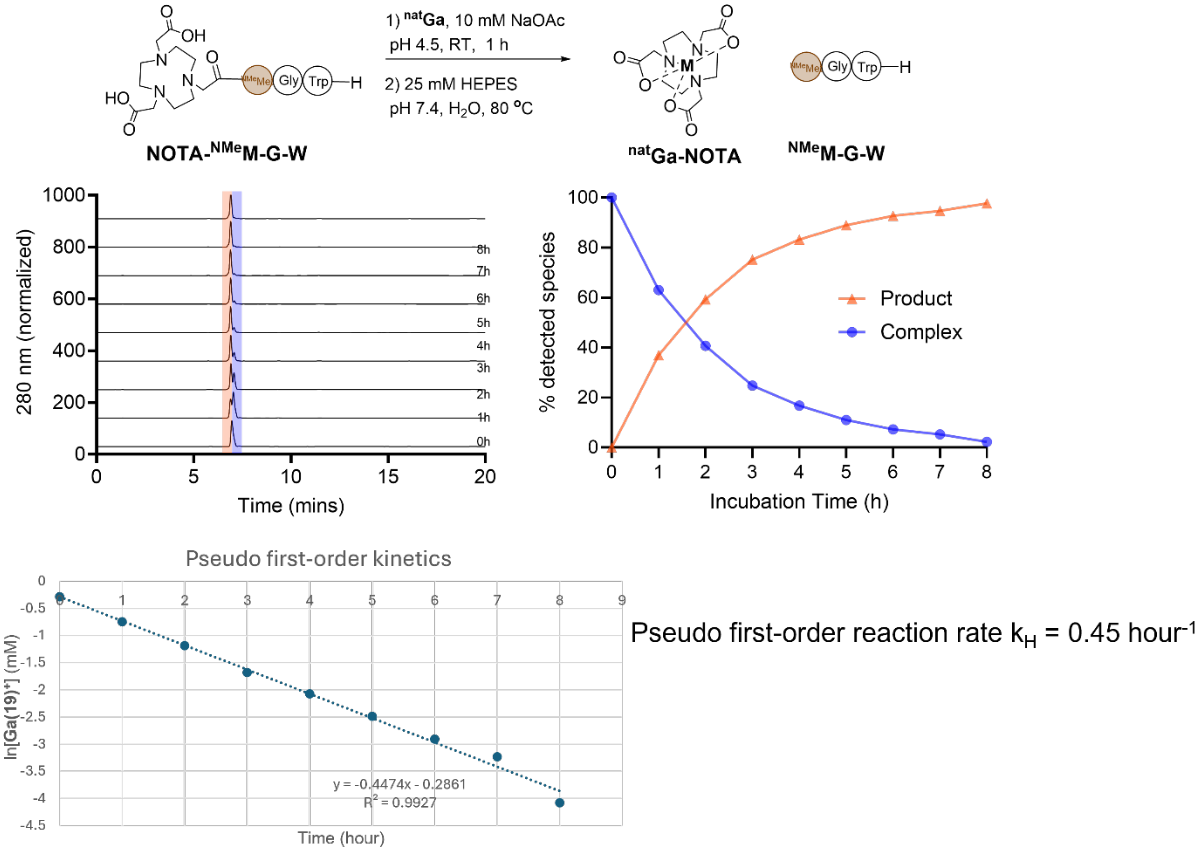

Figure S264. Schematic description of preparation and direct complexation procedure of **[natGa(19)]**$^{+}$. Analytical HPLC chromatograms showing autolytic release of the product, natGa-NOTA, in H_2O. The kinetics data was fit using $Y_t = -kt+Y_0$ in Excel, where Y_t represents the ln([concentration of intact species]) in mM, k represents the pseudo first-order reaction rate in hour^{-1}, t represents the incubation time in hour, and Y_0 presents the ln([initial concentration of intact species]) in mM.

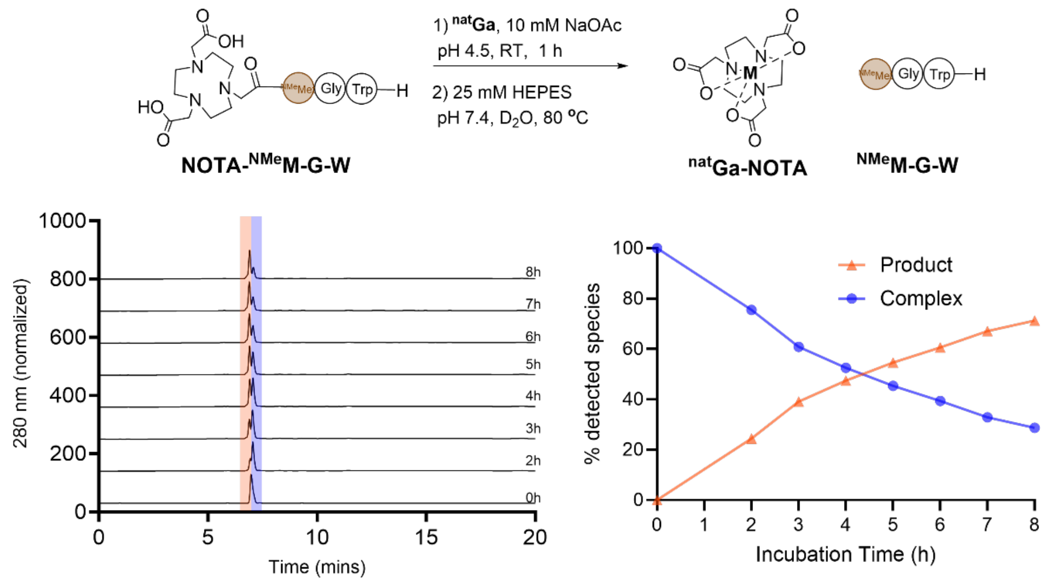

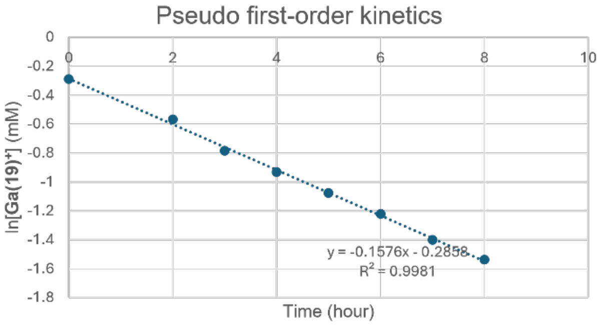

Pseudo first-order reaction rate k_D = 0.16 $hour^{-1}$

KIE = k_H/k_D = 0.45/0.16 = 2.81

Figure S265. Schematic description of preparation and direct complexation procedure of **[natGa(19)]**$^+$. Analytical HPLC chromatograms showing autolytic release of the product, natGa-NOTA, in D_2O. The kinetics data was fit using $Y_t = -kt+Y_0$ in Excel, where Y_t represents the ln([concentration of intact species]) in mM, k represents the pseudo first-order reaction rate in $hour^{-1}$, t represents the incubation time in hour, and Y_0 presents the ln([initial concentration of intact species]) in mM. The kinetic isotopic effect can be calculated by the equation KIE = k_L/k_H, where k_L refers to the reaction rate involving the lighter isotope (H_2O), and k_H refers to the reaction rate involving the heavier isotope (D_2O).

6 In vitro and In Vivo Experiments

All animal experiments were conducted with the approval of the University of Wisconsin-Madison Institutional Animal Care and Use Committee (IACUC). All studies were conducted in accordance with the relevant guidelines and regulations and approved under protocol number M006738 (PI: Boros) and conducted at UW-Madison School of Medicine and Public Health, at the Small Animal Imaging and Radiotherapy Facility (SAIRF).

6.1 HSA Binding Assay

To measure HSA binding of the complexes, a 0.05 mM solution (determined by ICP-OES) of the corresponding ^{nat}Ga complex in 4.5%w/v HSA was prepared and pipetted into a Amicon Ultra-0.5 Centrifugal Filter Unit (50 kDa cutoff, Millipore, UFC500396).[3] The mixture was incubated at 37 ºC for 15 min and subsequenctly centrifuged at 14000 rpm for 15 min. Binding is determined by measurement of Ga content in the filtrate by ICP-OES and compared to non-specific binding to the filter in absence of HSA. Experiments were conducted in accordance with published procedures.

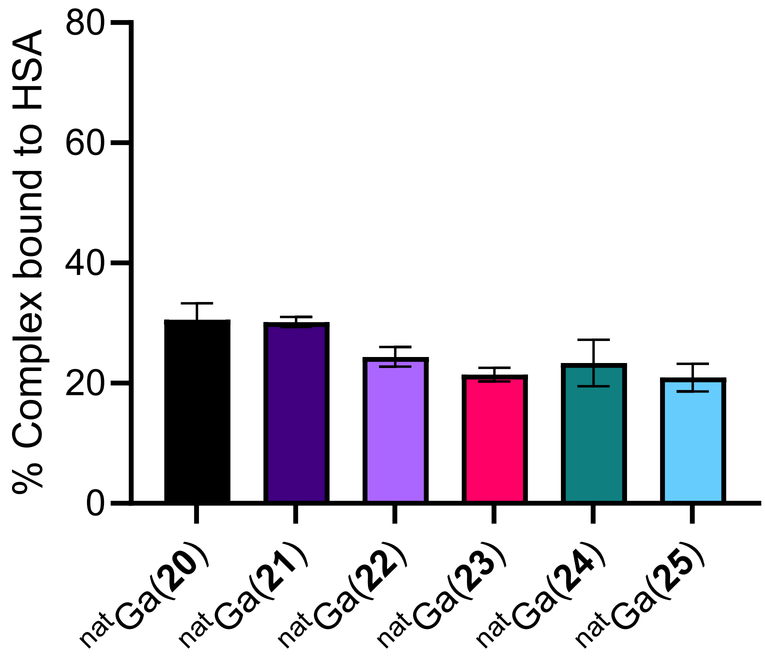

Figure S266. ICP-OES quantitation of Ga complex bound to 4.5% HSA in 50 mM pH 7.4 HEPES buffer.

Table S3. Tabulated data of %HSA bound Ga complexes

	**[^{nat}Ga(20)]$^+$**	**[^{nat}Ga(21)]$^+$**	**[^{nat}Ga(22)]$^+$**	**[^{nat}Ga(23)]$^+$**	**[^{nat}Ga(24)]$^+$**	**[^{nat}Ga(25)]$^+$**
HSA binding (%)	30.5±2.7	30.2±0.8	24.4±1.6	21.4±1.1	23.3±3.9	21.0±2.3

6.2 In Vivo Biodistribution and Pharmacokinetics in NU/J Mice

All animal experiments were conducted with the approval of the University of Wisconsin-Madison Institutional Animal Care and Use Committee (IACUC). All studies were conducted in accordance with the relevant guidelines and regulations and approved under protocol number M006738 (PI: Boros) and conducted at UW-Madison School of Medicine and Public Health, at the Small Animal Imaging and Radiotherapy Facility (SAIRF). Male NU/J mice (5 weeks) were purchased from Jackson Laboratory. [^{68}Ga][Ga(20)], [^{68}Ga][Ga(21)], [^{68}Ga][Ga(22)], [^{68}Ga][Ga(23)], [^{68}Ga][Ga(24)], and [^{68}Ga][Ga(2)] were intravenously injected via tail vein catheter in tumor-implanted mice. Mice were imaged by PET/CT scanner at 30-minute, 60-minute, and 90-minute post injection time points and sacrificed at 120-minute post-injection time point, and select organs (blood, heart, liver, kidneys, spleen, stomach, small intestines, large intestines, muscle, bone, tumor, brain and tail) were harvested. Radioactivity was counted by using a gamma counter, and the radioactivity associated with each organ was expressed as percent injected dose per gram tissue (%ID/g).

6.3 Tumor Xenograft Model

Tumor xenograft model PC3-Pip and PC3-flu cells were maintained in DMEM with 5% FBS at 37 ºC and 5% CO_2. Male NU/J mice (7 weeks, Jackson Laboratory) were implanted subcutaneously on the right shoulder with 1×10^6 PC3-Pip cells and on the left shoulder with 1×10^6 PC3-flu cells suspended in Matrigel (1:1). When tumors reached 500 mm^3, the mice were randomized based on tumor volumes into 4 groups (4 mice per group) for single dose compound administration.

Positron emission tomography/computed tomography (PET/CT) imaging: PET/CT imaging was performed using a Siemens Inveon Hybrid MicroPET/CT Scanner (Siemens Medical Solutions USA, Inc., Knoxville, TN). Mice were anesthetized with 4% isoflurane gas and anesthesia was maintained during scans at 2% isoflurane in oxygen. CT scans were acquired prior to PET scans for anatomical coregistration as well as attenuation correction. CT scan parameters were as follows: 220 rotation degrees, 120 rotation steps, binning factor of 4, exposure time of 250 ms, x-ray energy of 80 kVp, 1 mA current, and 105 μm resolution. PET scans were acquired with 40 million coincidence events per mouse, an energy window of 350-650 keV, and a timing window of 3.432 ns. Quantification of PET/CT images was performed in an Inveon Research Workstation and data is expressed as percent injected dose per gram of tissue (% ID/cc).

6.4 ^{68}Ga Tracer Preparation Procedure

To an aqueous solution of sodium acetate (20 μL, 1M, pH 4.5) was added 80 μL H_2O and ligand stock solution (2-10 μL, 10 nmol), of known concentration as determined UV-vis spectroscopy, followed by an aliquot of the [^{68}Ga]$GaCl_3$ stock (30 – 35 μL, ~600 μCi). Total reaction volume was 150 μL. The mixtures were incubated at room temperature for 15 min. The radiolabeled compounds were purified via radio-HPLC to achieve a radiochemical purity of > 98%.

6.5 In Vivo PET-CT Imaging and Ex Vivo Biodistribution Results

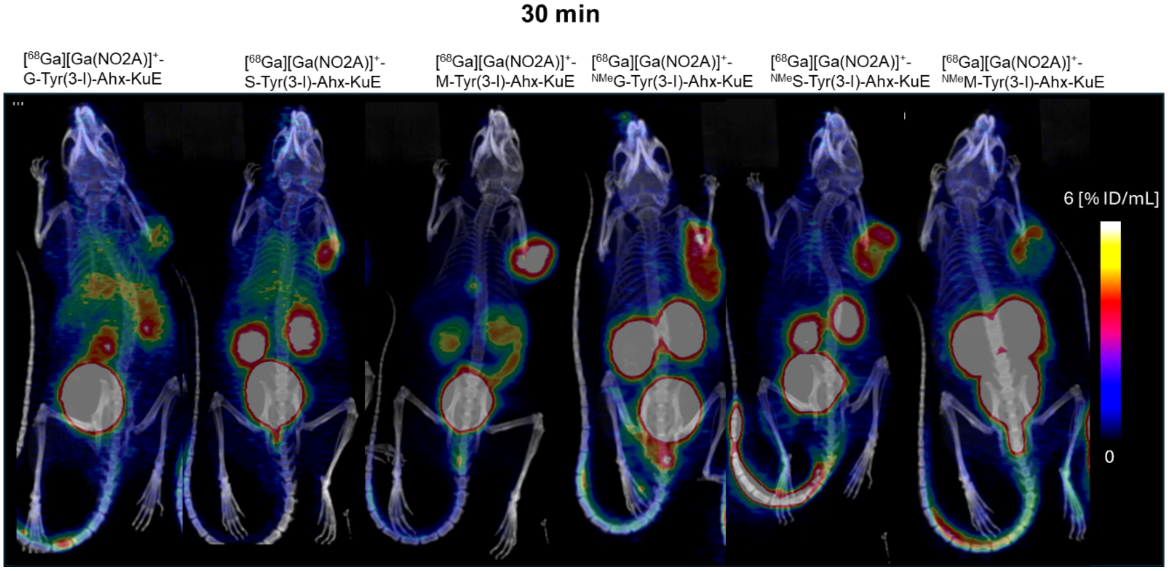

Figure S267. PET/CT images of Ahx-KuE peptide conjugates at 30-minute post injection time point.

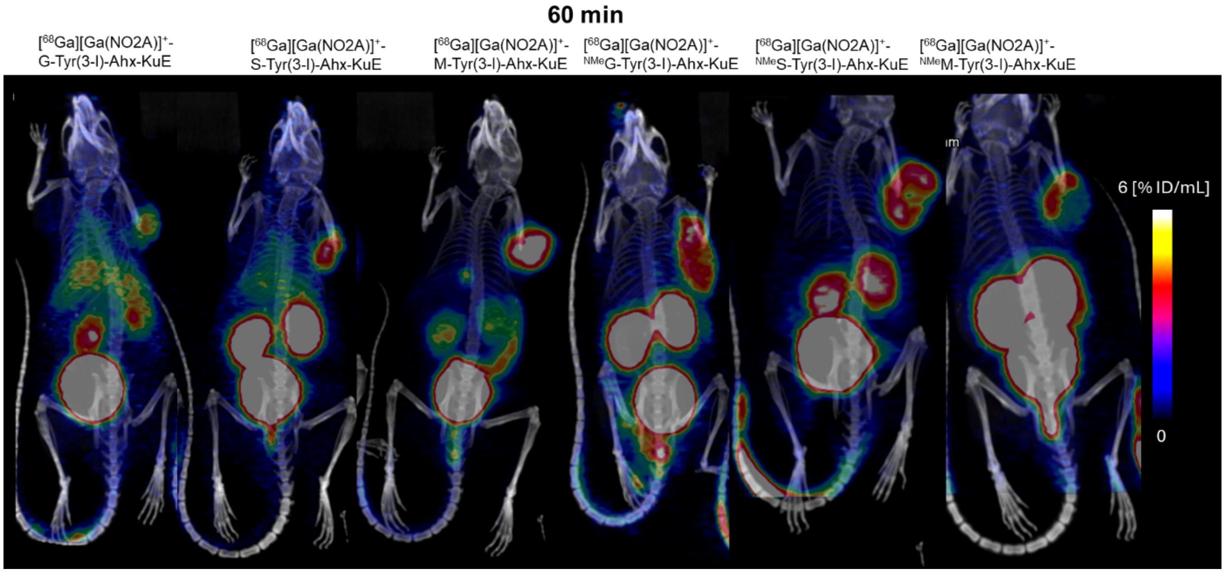

Figure S268. PET/CT images of Ahx-KuE conjugates at 60-minute post injection time point.

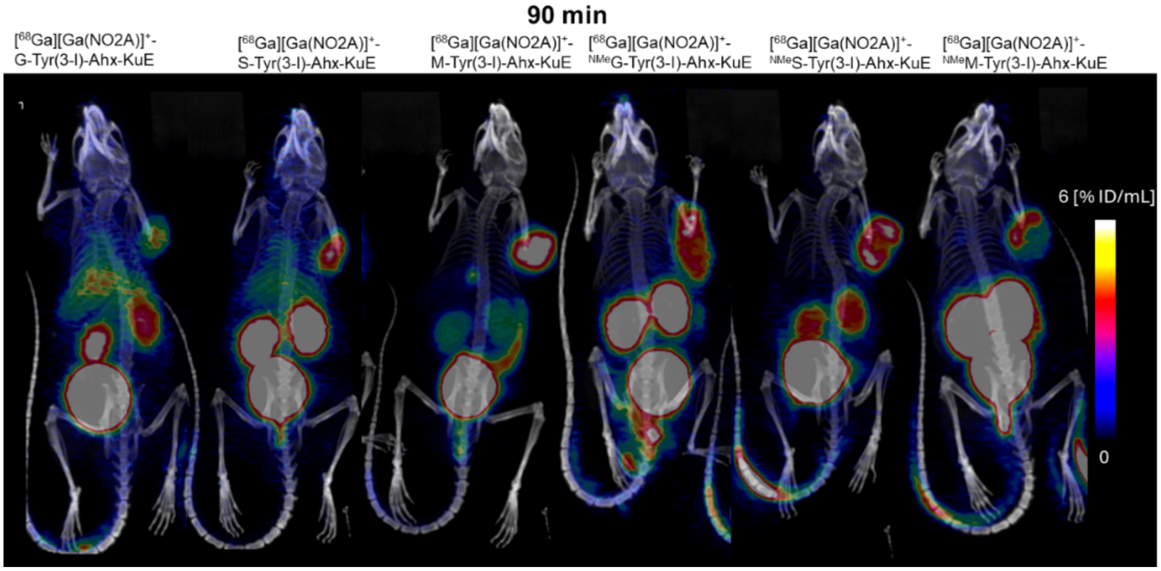

Figure S269. PET/CT images of Ahx-KuE conjugates at 90-minute post injection time point.

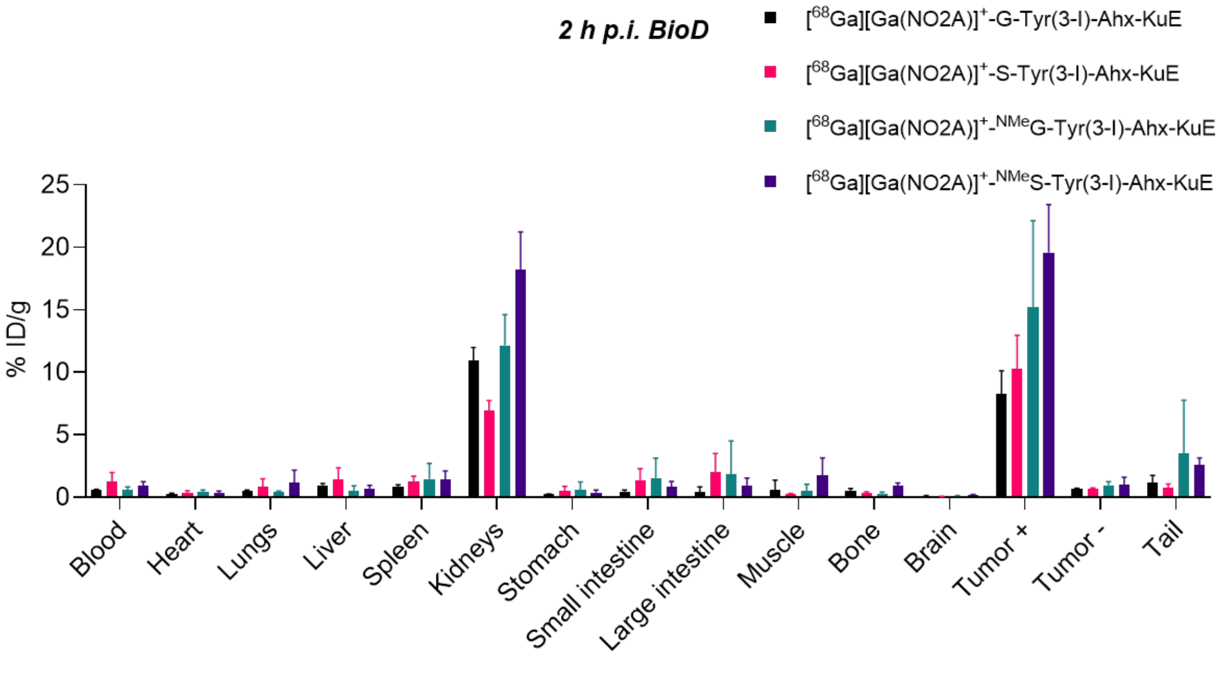

Figure S270. Ex vivo derived organ uptake values for Ahx-KuE conjugates **[^{68}Ga][Ga(20)]** (Gly-linked), **[^{68}Ga][Ga(21)]** (Ser-linked), **[^{68}Ga][Ga(23)]** (^{NMe}Gly-linked), and **[^{68}Ga][Ga(24)]** (^{NMe}Ser-linked) at 2-hour post injection time point.

6.6 Metabolite Analysis

The 100 µL mouse urine was collected and analyzed by radio-HPLC during biodistribution studies. If mouse urine was less than 100 µL, the urine was diluted by 1 x PBS to total volume of 100 µL. If the total activity was below the sensitivity of the radio detector, fractions were collected every 30 seconds. The metabolite traces were reconstructed by quantifying each fraction using a gamma counter.

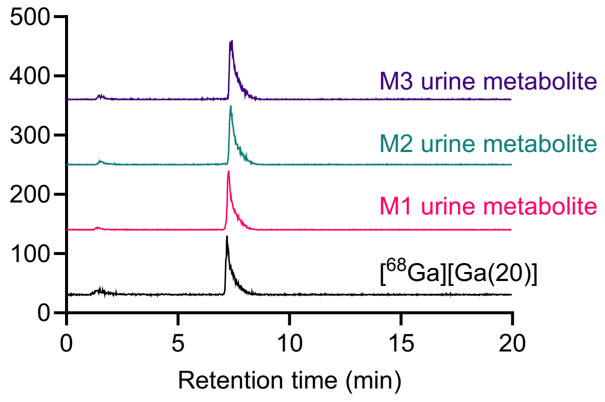

Figure S271. Urine metabolite identification for $[^{68}Ga][Ga(NO2A)]^{+}$-G-Tyr(3-I)-Ahx-KuE, **$[^{68}Ga][Ga(20)]$**, at 120-minute post injection time point.

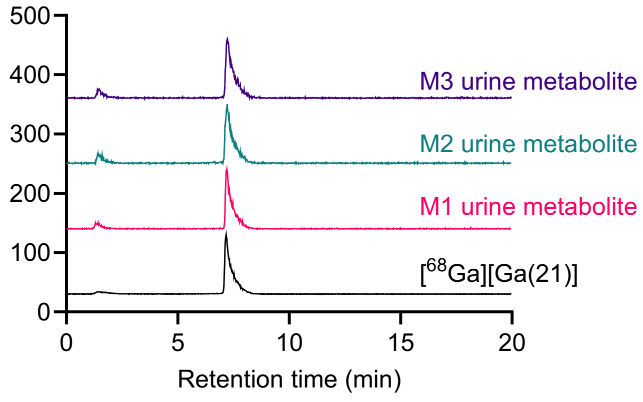

Figure S272. Urine metabolite identification for $[^{68}Ga][Ga(NO2A)]^{+}$-S-Tyr(3-I)-Ahx-KuE, **$[^{68}Ga][Ga(21)]$**, at 120-minute post injection time point.

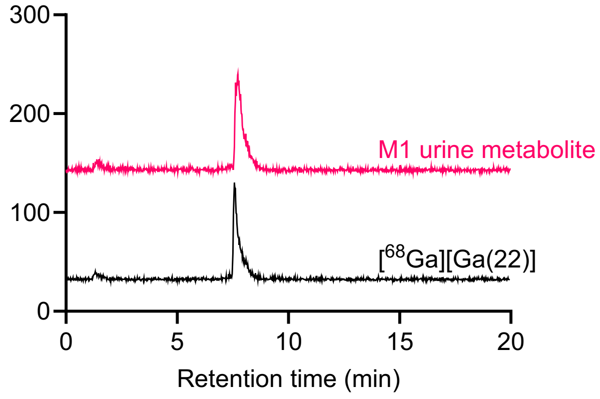

Figure S273. Urine metabolite identification for [^{68}Ga][Ga(NO2A)]$^{+}$-M-Tyr(3-I)-Ahx-KuE, **[^{68}Ga][Ga(22)]**, at 120-minute post injection time point.

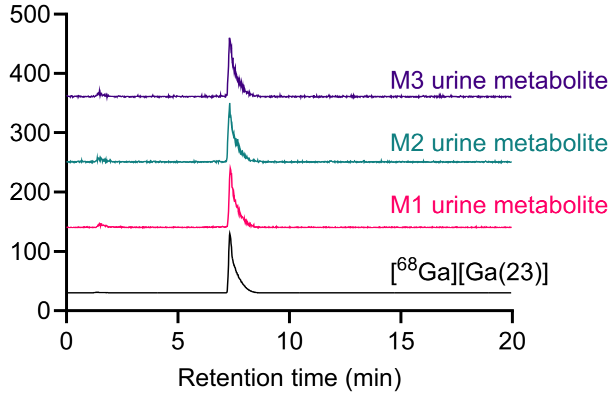

Figure S274. Urine metabolite identification for [^{68}Ga][Ga(NO2A)]$^{+}$-^{NMe}G-Tyr(3-I)-Ahx-KuE, **[^{68}Ga][Ga(23)]**, at 120-minute post injection time point.

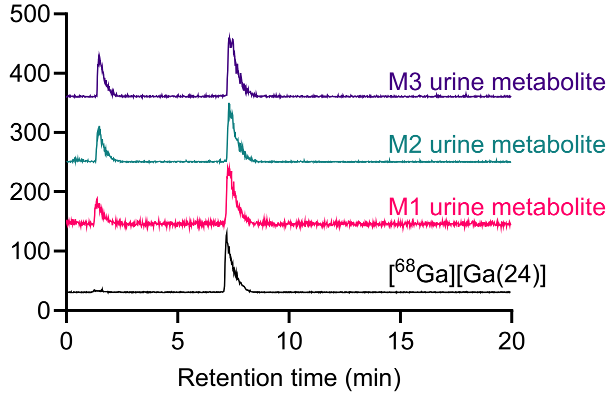

Figure S275. Urine metabolite identification for [^{68}Ga][Ga(NO2A)]$^{+}$-^{NMe}S-Tyr(3-I)-Ahx-KuE, **[^{68}Ga][Ga(24)]**, at 120-minute post injection time point.

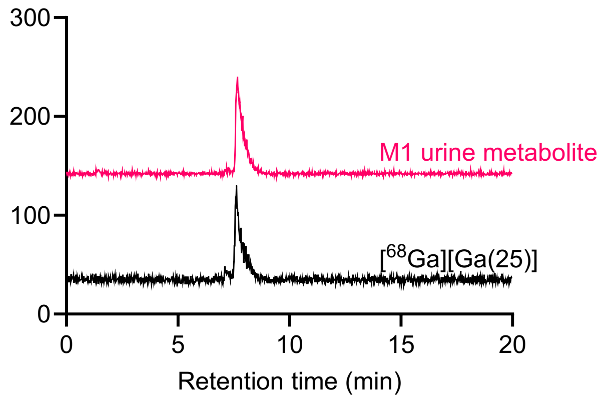

Figure S276. Urine metabolite identification for [^{68}Ga][Ga(NO2A)]$^{+}$-^{NMe}M-Tyr(3-I)-Ahx-KuE, **[^{68}Ga][Ga(25)]**, at 120-minute post injection time point.

6.7 Tabulated Biodistribution Data

Table S4. Decay-corrected biodistribution of **[^{68}Ga][Ga(20)]**, **[^{68}Ga][Ga(21)]**, **[^{68}Ga][Ga(22)]**, **[^{68}Ga][Ga(23)]**, **[^{68}Ga][Ga(24)]**, and **[^{68}Ga][Ga(25)]** at 2-hour post injection (n=3) in PC-3 tumor bearing mice at 2 hours post-injection.

Organ	[^{68}Ga][Ga(20)]	[^{68}Ga][Ga(21)]	[^{68}Ga][Ga(22)]	[^{68}Ga][Ga(23)]	[^{68}Ga][Ga(24)]	[^{68}Ga][Ga(25)]
**Blood**	0.56±0.0766	1.26±0.7123	0.40	0.57±0.2472	0.92±0.3318	0.44
**Heart**	0.24±0.0835	0.32±0.1858	0.09	0.39±0.1817	0.35±0.1313	0.16
**Lungs**	0.52±0.0556	0.87±0.6081	0.33	0.42±0.0792	1.18±0.9774	0.34
**Liver**	0.95±0.1417	1.38±0.9722	0.52	0.49±0.4132	0.69±0.2596	0.21
**Spleen**	0.85±0.1314	1.26±0.4127	0.34	1.45±1.2518	1.42±0.6688	0.75
**Kidneys**	10.92±1.0692	6.93±0.8059	3.53	12.16±2.4676	18.21±3.0209	36.60
**Stomach**	0.21±0.0589	0.52±0.3524	0.36	0.59±0.6341	0.37±0.2037	0.82
**Small intestine**	0.45±0.1296	1.38±0.9157	0.36	1.51±1.6064	0.82±0.4449	0.63
**Large intestine**	0.42±0.4167	2.02±1.4687	1.84	1.82±2.6773	0.91±0.6334	0.78
**Muscle**	0.60±0.7694	0.26±0.0414	0.09	0.50±0.5377	1.75±1.4060	0.09
**Bone**	0.51±0.1901	0.34±0.0854	0.18	0.27±0.1359	0.89±0.2564	0.17
**Brain**	0.09±0.0532	0.050±0.0143	0.03	0.09±0.0366	0.14±0.0692	0.04
**PC-3 Pip**	8.28±1.8289	10.23±2.7391	7.52	15.21±6.9231	19.59±3.8450	3.45
**PC-3 flu**	0.65±0.0632	0.64±0.1106	0.64	0.94±0.2957	1.02±0.5842	0.83

**Tail**	1.16±0.5718	0.78±0.2851	0.86	3.48±4.2869	2.56±0.5917	3.06

7 X-ray Diffraction Analysis

7.1 X-ray Diffraction Analysis of [Ga(27)]ClO_4

7.1.1 Data Collection

A colorless crystal with approximate dimensions 0.158 x 0.078 x 0.025 mm^3 was selected under oil under ambient conditions and attached to the tip of a MiTeGen MicroMount©. The crystal was mounted in a stream of cold nitrogen at 100(1) K and centered in the X-ray beam by using a video camera.

The crystal evaluation and data collection were performed on a Bruker D8 VENTURE Photon III four-circle diffractometer with Diamond II IµS Mo Kα (λ = 0.71073 Å) radiation and the detector to crystal distance of 5.0 cm.[4]

The initial cell constants were obtained from a 180° φ scan conducted at a 2θ = 30° angle with the exposure time of 1 second per frame. The reflections were successfully indexed by an automated indexing routine built in the APEX6 program. The final cell constants were calculated from a set of 9870 strong reflections from the actual data collection.

The data were collected by using the half sphere data collection routine to survey the reciprocal space to the extent of a half sphere to a resolution of 0.70 Å. A total of 48380 data were harvested by collecting 10 sets of frames with 0.5° scans in ω and φ with an exposure time 30 sec per frame. These highly redundant datasets were corrected for Lorentz and polarization effects. The absorption correction was based on fitting a function to the empirical transmission surface as sampled by multiple equivalent measurements.[5]

7.1.2 Structure Solution and Refinement

The systematic absences in the diffraction data were consistent for the space groups *P*2/*n* and *Pn*. The *E*-statistics strongly suggested the non-centrosymmetric space group *Pn* that yielded chemically reasonable and computationally stable results of refinement.[6-11]

A successful solution by intrinsic phasing provided most non-hydrogen atoms from the *E*-map. The remaining non-hydrogen atoms were located in an alternating series of least-squares cycles and difference Fourier maps. All non-hydrogen atoms were refined with anisotropic displacement coefficients. All hydrogen atoms were included in the structure factor calculation at idealized positions and were allowed to ride on the neighboring atoms with relative isotropic displacement coefficients.

The asymmetric unit contains one $[C_{20}H_{28}N_4O_5Ga]^+$ unit and one perchlorate anion for an overall chemical formula of $[C_{20}H_{28}N_4O_5Ga][ClO_4]$.

The absolute structure was established by resonant scattering effects.

The compound crystallizes as a racemate. The enantiomer shown in Figure 1 (N1 – *S*, N2 – *R*, N3 – *S*) was chosen arbitrarily.

The final least-squares refinement of 317 parameters against 6293 data resulted in residuals *R* (based on F^2 for $I \geq 2\sigma$) and *wR* (based on F^2 for all data) of 0.026 and 0.0612, respectively. The final difference Fourier map was featureless.

7.1.3 Summary

Crystal Data for $C_{20}H_{28}ClGaN_4O_9$ (M =573.63 g/mol): monoclinic, space group Pn (no. 7), a = 8.969(2) Å, b = 10.704(2) Å, c = 12.0242(15) Å, β = 96.234(11)°, V = 1147.5(4) Å^3, Z = 2, T = 100.00 K, μ(Mo Kα) = 1.376 mm^{-1}, $Dcalc$ = 1.660 g/cm^3, 47234 reflections measured (3.806° ≤ 2Θ ≤ 61.056°), 6293 unique (R_{int} = 0.0387, R_{sigma} = 0.0273) which were used in all calculations. The final R_1 was 0.0260 (I > 2σ(I)) and wR_2 was 0.0612 (all data).

7.1.4 Acknowledgement

The purchase of the Bruker D8 VENTURE Photon III Diamond II IμS X-ray diffractometer was partially funded by a 2024 University of Wisconsin Research Core Revitalization Program Round 3 Award to the Department of Chemistry.

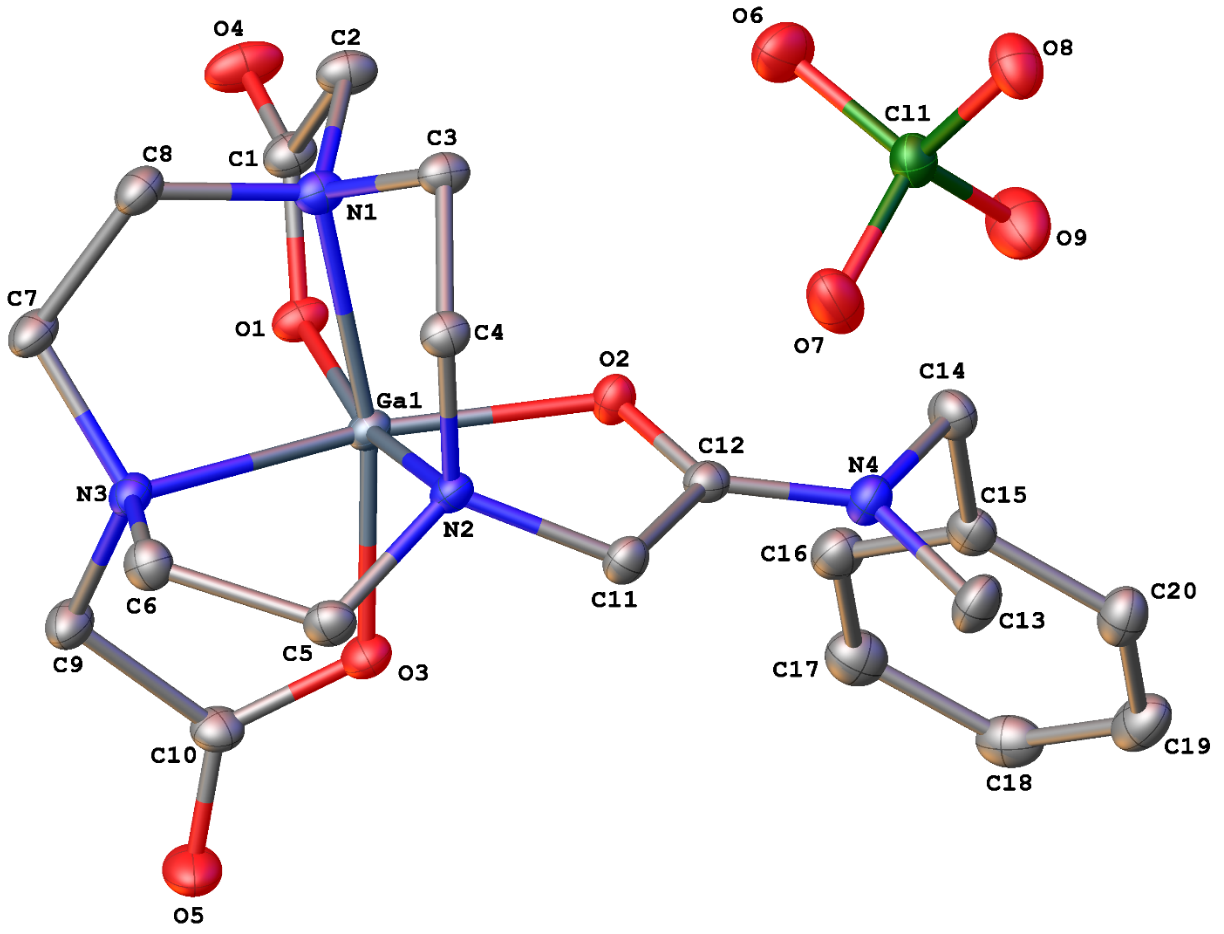

Figure S277. A molecular drawing of [Ga(27)]ClO_4 shown with 50% probability ellipsoids. All H atoms are omitted.

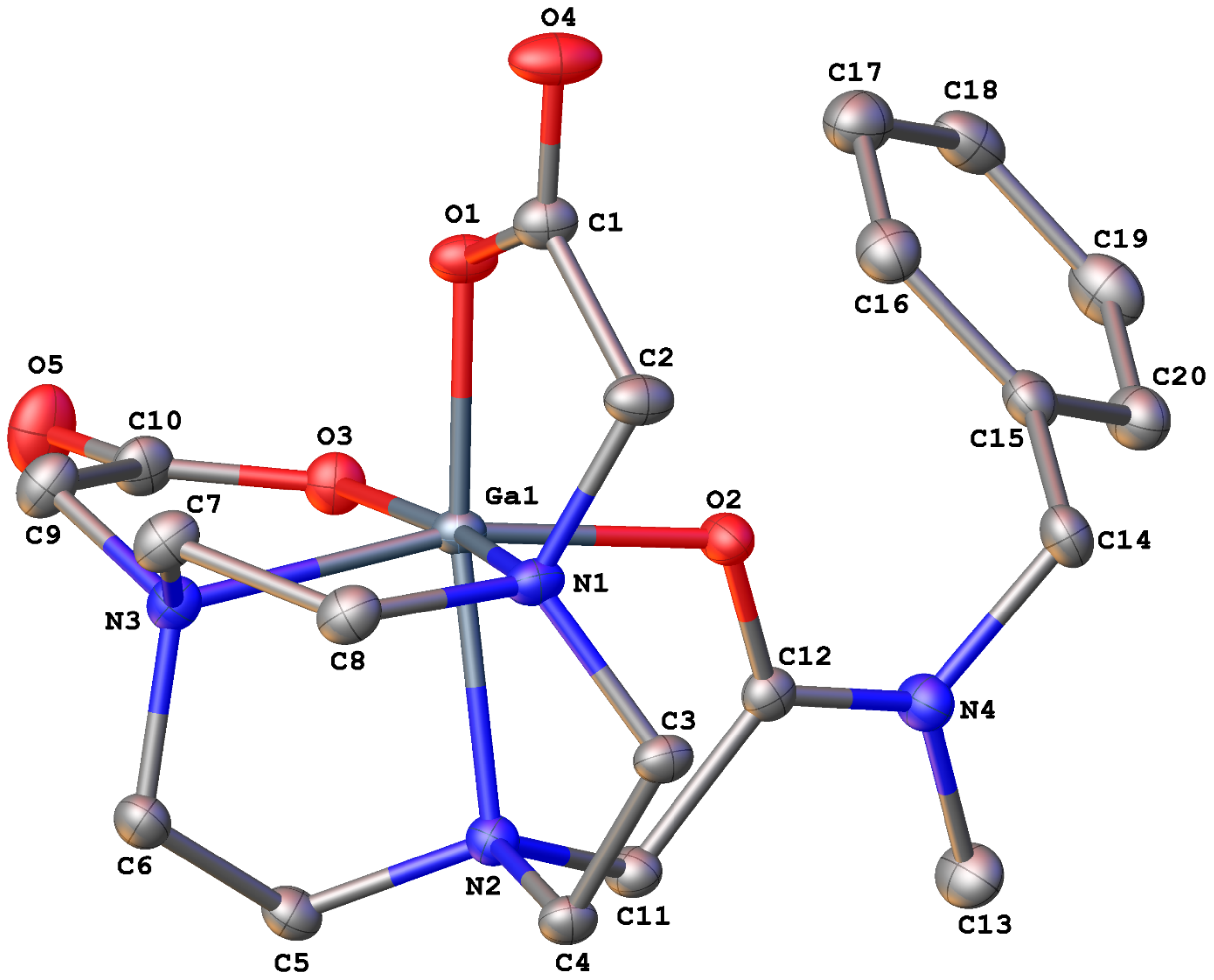

Figure S278. A chemical drawing of the cation in [Ga(27)]ClO_4 shown with 50% probability ellipsoids. All H atoms are omitted.

**Table S5 Crystal data and structure refinement for [Ga(27)]ClO_4.**	
**Identification code**	[Ga(27)]ClO_4
**Empirical formula**	[$C_{20}H_{28}N_4O_5Ga$][ClO_4]
**Formula weight**	573.63
**Temperature/K**	100.00
**Crystal system**	monoclinic
**Space group**	*Pn*
**a/Å**	8.969(2)
**b/Å**	10.704(2)
**c/Å**	12.0242(15)
**α/°**	90
**β/°**	96.234(11)
**γ/°**	90
**Volume/Å^3**	1147.5(4)
**Z**	2
**ρ_{calc}g/cm^3**	1.660
**μ/mm^{-1}**	1.376
**F(000)**	592.0
**Crystal size/mm^3**	0.158 × 0.078 × 0.025
**Radiation**	Mo Kα (λ = 0.71073)
**2Θ range for data collection/°**	3.806 to 61.056
**Index ranges**	$-12 \leq h \leq 12$, $-14 \leq k \leq 15$, $-17 \leq l \leq 15$
**Reflections collected**	47234
**Independent reflections**	6293 [R_{int} = 0.0387, R_{sigma} = 0.0273]
**Data/restraints/parameters**	6293/2/317
**Goodness-of-fit on F^2**	1.037
**Final R indexes [I>=2σ (I)]**	R_1 = 0.0260, wR_2 = 0.0585
**Final R indexes [all data]**	R_1 = 0.0306, wR_2 = 0.0612
**Largest diff. peak/hole / e Å^{-3}**	0.42/-0.40
**Flack parameter**	-0.009(4)

Table S6 Fractional Atomic Coordinates (×10^4) and Equivalent Isotropic Displacement Parameters (Å^2×10^3) for [Ga(27)]ClO$_4$. U$_{eq}$ is defined as 1/3 of the trace of the orthogonalised U$_{IJ}$ tensor.

Atom	*x*	*y*	*z*	U(eq)
**Ga1**	4685.3(2)	5535.3(2)	4031.2(2)	11.82(6)
**O1**	6664(2)	5352.4(18)	4823.4(16)	15.7(4)
**O2**	4324(2)	7234.3(17)	4542.2(15)	15.1(4)
**O3**	3649(2)	4744.6(18)	5160.7(16)	16.5(4)
**O4**	9081(2)	5750(2)	4713.8(18)	24.8(5)
**O5**	3049(3)	2877(2)	5798.9(19)	26.8(5)
**N1**	5887(2)	6019(2)	2706.2(18)	14.0(4)
**N2**	2789(2)	5982(2)	2907.7(18)	12.5(4)
**N3**	4492(3)	3791(2)	3266.0(18)	13.6(4)
**N4**	2510(3)	8627(2)	4788.9(19)	16.2(4)
**C1**	7765(3)	5820(3)	4337(2)	16.2(5)
**C2**	7311(3)	6530(3)	3254(2)	17.6(5)
**C3**	4943(3)	6962(2)	2057(2)	14.5(5)
**C4**	3325(3)	6497(2)	1855(2)	14.4(5)
**C5**	1983(3)	4779(2)	2711(2)	16.1(5)
**C6**	3090(3)	3758(2)	2462(2)	16.4(5)
**C7**	5869(3)	3673(3)	2687(2)	17.1(5)
**C8**	6118(3)	4865(3)	2038(2)	16.7(5)
**C9**	4426(3)	2919(3)	4216(2)	18.0(5)
**C10**	3625(3)	3525(3)	5132(2)	17.0(5)
**C11**	1915(3)	6904(2)	3485(2)	14.8(5)
**C12**	2982(3)	7627(2)	4317(2)	13.8(5)
**C13**	939(3)	9048(3)	4593(2)	21.2(5)
**C14**	3558(3)	9393(2)	5542(2)	17.5(5)
**C15**	3483(3)	9076(3)	6757(2)	17.1(5)
**C16**	4297(3)	8072(3)	7247(2)	20.1(5)
**C17**	4258(3)	7801(3)	8372(3)	23.4(6)
**C18**	3387(3)	8529(3)	9015(2)	22.5(6)
**C19**	2560(4)	9505(3)	8533(3)	24.3(6)
**C20**	2618(3)	9786(3)	7408(3)	21.0(6)
**Cl1**	2700.6(8)	10184.4(7)	1716.6(6)	18.20(13)
**O6**	4144(3)	9644(2)	1601(2)	28.5(5)
**O7**	1565(3)	9248(2)	1464(2)	27.9(5)
**O8**	2439(3)	11215.6(19)	960.0(18)	24.0(4)
**O9**	2656(3)	10612(2)	2848.3(19)	30.7(5)

Table S7 Anisotropic Displacement Parameters ($Å^2 \times 10^3$) for [Ga(27)]ClO_4. The Anisotropic displacement factor exponent takes the form: -$2\pi^2[h^2a^{*2}U_{11}+2hka^*b^*U_{12}+…]$.

Atom	U_{11}	U_{22}	U_{33}	U_{23}	U_{13}	U_{12}
**Ga1**	10.57(11)	15.09(12)	9.88(11)	0.27(11)	1.41(8)	0.76(12)
**O1**	12.5(9)	22.7(10)	11.9(9)	2.2(7)	1.5(7)	0.8(7)
**O2**	13.1(9)	15.5(8)	16.4(9)	-2.5(7)	0.4(7)	1.6(7)
**O3**	17.6(9)	18.6(9)	14.2(9)	-0.1(7)	5.6(7)	0.0(7)
**O4**	12.2(9)	42.9(13)	18.9(10)	6.7(9)	0.2(8)	-0.2(9)
**O5**	36.3(13)	22.1(10)	25.0(11)	4.3(8)	16.5(10)	-0.6(9)
**N1**	12.1(10)	17.6(10)	12.1(10)	2.5(8)	0.6(8)	0.9(8)
**N2**	11.1(10)	14.2(10)	12.5(10)	-0.7(8)	2.9(8)	0.3(8)
**N3**	14.8(10)	14.4(10)	12.1(9)	0.0(7)	3.9(8)	1.7(8)
**N4**	14.7(10)	17.2(10)	16.7(10)	-2.6(8)	2.4(9)	1.5(8)
**C1**	14.6(12)	20.8(12)	13.4(11)	1.6(9)	2.5(9)	0.7(10)
**C2**	12.5(12)	24.4(13)	15.7(12)	3.3(10)	0.4(9)	-1.6(10)
**C3**	12.2(11)	17.7(12)	13.4(11)	3.1(9)	0.9(9)	0.2(9)
**C4**	13.9(11)	17.2(12)	11.8(11)	1.6(9)	0.5(9)	0.6(9)
**C5**	13.3(12)	18.9(12)	15.9(12)	-1.3(9)	1.1(10)	-2.3(9)
**C6**	17.5(12)	16.7(12)	14.7(11)	-1.6(9)	0.2(10)	-0.7(10)
**C7**	17.4(13)	20.5(13)	14.6(12)	2.2(10)	6.0(10)	4.3(10)
**C8**	16.0(12)	19.8(12)	14.9(12)	0.5(9)	4.7(10)	3.6(10)
**C9**	20.6(14)	17.9(12)	16.3(13)	2.4(9)	6.2(10)	3.9(10)
**C10**	19.2(14)	18.7(13)	13.4(12)	3.1(10)	3.9(10)	-0.7(10)
**C11**	12.1(11)	16.7(11)	15.6(11)	-1.1(9)	1.3(9)	1.7(9)
**C12**	13.5(11)	14.5(11)	13.7(11)	1.8(9)	2.8(9)	-0.1(9)
**C13**	16.9(13)	23.1(13)	24.0(14)	-4.1(11)	4.0(11)	4.6(10)
**C14**	18.9(13)	15.4(12)	18.2(13)	-3.3(9)	1.9(10)	-3.1(10)
**C15**	14.9(12)	16.5(12)	19.6(13)	-3.2(9)	0.9(10)	-3.0(9)
**C16**	16.4(12)	21.2(13)	22.9(13)	-1.9(10)	3.8(10)	0.2(10)
**C17**	21.0(14)	24.4(14)	24.4(14)	2.3(11)	0.3(11)	-1.1(11)
**C18**	21.8(14)	28.3(15)	17.0(13)	-1.9(11)	0.9(11)	-7.6(11)
**C19**	21.7(14)	30.1(15)	21.8(14)	-8.4(11)	6.0(12)	-2.0(12)
**C20**	19.7(13)	21.3(13)	22.0(14)	-4.5(10)	1.6(11)	2.0(10)
**Cl1**	18.8(3)	15.6(3)	20.0(3)	0.0(2)	1.4(2)	1.9(3)
**O6**	19.9(11)	27.7(12)	38.6(13)	5.3(9)	6.0(10)	5.4(9)
**O7**	22.7(12)	18.9(10)	41.1(14)	0.1(10)	-0.5(10)	-0.2(9)
**O8**	32.7(12)	17.8(10)	21.2(10)	2.2(8)	1.6(9)	2.4(8)

**O9**	46.4(15)	27.9(12)	18.5(10)	-2.3(8)	6.7(10)	-1.4(10)

Table S8 Bond Lengths for [Ga(27)]ClO$_4$.

Atom	Atom	Length/Å	Atom	Atom	Length/Å
**Ga1**	O1	1.931(2)	N4	C12	1.303(3)
**Ga1**	O2	1.9577(19)	N4	C13	1.474(4)
**Ga1**	O3	1.923(2)	N4	C14	1.480(3)
**Ga1**	N1	2.084(2)	C1	C2	1.524(4)
**Ga1**	N2	2.109(2)	C3	C4	1.529(4)
**Ga1**	N3	2.080(2)	C5	C6	1.528(4)
**O1**	C1	1.302(3)	C7	C8	1.524(4)
**O2**	C12	1.276(3)	C9	C10	1.524(4)
**O3**	C10	1.306(3)	C11	C12	1.519(4)
**O4**	C1	1.220(3)	C14	C15	1.509(4)
**O5**	C10	1.217(3)	C15	C16	1.393(4)
**N1**	C2	1.477(3)	C15	C20	1.387(4)
**N1**	C3	1.483(3)	C16	C17	1.388(4)
**N1**	C8	1.500(3)	C17	C18	1.396(4)
**N2**	C4	1.507(3)	C18	C19	1.373(4)
**N2**	C5	1.483(3)	C19	C20	1.392(4)
**N2**	C11	1.479(3)	Cl1	O6	1.438(2)
**N3**	C6	1.501(3)	Cl1	O7	1.438(3)
**N3**	C7	1.487(4)	Cl1	O8	1.433(2)
**N3**	C9	1.481(3)	Cl1	O9	1.440(2)

Table S9 Bond Angles for [Ga(27)]ClO$_4$.

Atom	Atom	Atom	Angle/°	Atom	Atom	Atom	Angle/°
**O1**	Ga1	O2	96.54(8)	C12	N4	C13	122.1(2)
**O1**	Ga1	N1	83.01(8)	C12	N4	C14	120.4(2)
**O1**	Ga1	N2	166.70(8)	C13	N4	C14	117.4(2)
**O1**	Ga1	N3	98.92(9)	O1	C1	C2	115.5(2)
**O2**	Ga1	N1	97.24(9)	O4	C1	O1	124.0(2)
**O2**	Ga1	N2	80.85(8)	O4	C1	C2	120.4(2)
**O2**	Ga1	N3	164.53(8)	N1	C2	C1	109.5(2)
**O3**	Ga1	O1	95.50(8)	N1	C3	C4	109.9(2)
**O3**	Ga1	O2	94.47(8)	N2	C4	C3	111.7(2)
**O3**	Ga1	N1	168.29(9)	N2	C5	C6	109.7(2)
**O3**	Ga1	N2	97.70(9)	N3	C6	C5	112.0(2)
**O3**	Ga1	N3	83.91(8)	N3	C7	C8	110.1(2)

**N1**	Ga1	N2	84.39(9)	N1	C8	C7	112.3(2)
**N3**	Ga1	N1	84.84(9)	N3	C9	C10	110.5(2)
**N3**	Ga1	N2	84.12(9)	O3	C10	C9	115.9(2)
**C1**	O1	Ga1	116.16(17)	O5	C10	O3	124.0(3)
**C12**	O2	Ga1	115.09(16)	O5	C10	C9	120.1(3)
**C10**	O3	Ga1	115.39(18)	N2	C11	C12	108.7(2)
**C2**	N1	Ga1	104.20(16)	O2	C12	N4	121.5(2)
**C2**	N1	C3	113.5(2)	O2	C12	C11	118.9(2)
**C2**	N1	C8	112.4(2)	N4	C12	C11	119.6(2)
**C3**	N1	Ga1	104.97(16)	N4	C14	C15	112.1(2)
**C3**	N1	C8	112.5(2)	C16	C15	C14	120.4(2)
**C8**	N1	Ga1	108.51(16)	C20	C15	C14	120.7(3)
**C4**	N2	Ga1	108.18(15)	C20	C15	C16	119.0(3)
**C5**	N2	Ga1	104.51(16)	C17	C16	C15	120.3(3)
**C5**	N2	C4	112.2(2)	C16	C17	C18	119.9(3)
**C11**	N2	Ga1	106.38(15)	C19	C18	C17	120.1(3)
**C11**	N2	C4	112.6(2)	C18	C19	C20	119.9(3)
**C11**	N2	C5	112.4(2)	C15	C20	C19	120.8(3)
**C6**	N3	Ga1	109.26(15)	O6	Cl1	O9	109.67(16)
**C7**	N3	Ga1	104.46(17)	O7	Cl1	O6	108.86(15)
**C7**	N3	C6	112.1(2)	O7	Cl1	O9	109.23(15)
**C9**	N3	Ga1	103.48(16)	O8	Cl1	O6	109.61(14)
**C9**	N3	C6	112.4(2)	O8	Cl1	O7	109.83(14)
**C9**	N3	C7	114.3(2)	O8	Cl1	O9	109.62(13)

Table S10 Torsion Angles for [Ga(27)]ClO$_4$.

**A**	**B**	**C**	**D**	**Angle/°**	**A**	**B**	**C**	**D**	**Angle/°**
**Ga1**	O1	C1	O4	-177.8(2)	C3	N1	C8	C7	-133.3(2)
**Ga1**	O1	C1	C2	4.4(3)	C4	N2	C5	C6	69.6(3)
**Ga1**	O2	C12	N4	166.4(2)	C4	N2	C11	C12	-90.5(2)
**Ga1**	O2	C12	C11	-13.3(3)	C5	N2	C4	C3	-134.8(2)
**Ga1**	O3	C10	O5	178.3(2)	C5	N2	C11	C12	141.6(2)
**Ga1**	O3	C10	C9	0.3(3)	C6	N3	C7	C8	71.2(3)
**Ga1**	N1	C2	C1	35.2(2)	C6	N3	C9	C10	-84.3(3)
**Ga1**	N1	C3	C4	-48.0(2)	C7	N3	C6	C5	-134.4(2)
**Ga1**	N1	C8	C7	-17.7(3)	C7	N3	C9	C10	146.4(2)
**Ga1**	N2	C4	C3	-20.0(2)	C8	N1	C2	C1	-82.1(3)
**Ga1**	N2	C5	C6	-47.4(2)	C8	N1	C3	C4	69.8(3)
**Ga1**	N2	C11	C12	27.8(2)	C9	N3	C6	C5	95.1(3)

**Ga1**	N3	C6	C5	-19.1(2)	C9	N3	C7	C8	-159.4(2)
**Ga1**	N3	C7	C8	-47.0(2)	C11	N2	C4	C3	97.2(2)
**Ga1**	N3	C9	C10	33.4(2)	C11	N2	C5	C6	-162.3(2)
**O1**	C1	C2	N1	-28.5(3)	C12	N4	C14	C15	-97.0(3)
**O4**	C1	C2	N1	153.7(3)	C13	N4	C12	O2	-176.1(2)
**N1**	C3	C4	N2	46.6(3)	C13	N4	C12	C11	3.6(4)
**N2**	C5	C6	N3	45.8(3)	C13	N4	C14	C15	83.6(3)
**N2**	C11	C12	O2	-11.7(3)	C14	N4	C12	O2	4.5(4)
**N2**	C11	C12	N4	168.6(2)	C14	N4	C12	C11	-175.8(2)
**N3**	C7	C8	N1	44.5(3)	C14	C15	C16	C17	178.3(3)
**N3**	C9	C10	O3	-24.8(4)	C14	C15	C20	C19	-179.2(3)
**N3**	C9	C10	O5	157.2(3)	C15	C16	C17	C18	0.6(4)
**N4**	C14	C15	C16	83.5(3)	C16	C15	C20	C19	0.1(4)
**N4**	C14	C15	C20	-97.2(3)	C16	C17	C18	C19	0.6(4)
**C2**	N1	C3	C4	-161.1(2)	C17	C18	C19	C20	-1.5(5)
**C2**	N1	C8	C7	97.0(3)	C18	C19	C20	C15	1.1(5)
**C3**	N1	C2	C1	148.8(2)	C20	C15	C16	C17	-0.9(4)

Table S11 Hydrogen Atom Coordinates (Å×10^4) and Isotropic Displacement Parameters (Å^2×10^3) for [Ga(27)]ClO$_4$.

**Atom**	***x***	***y***	***z***	**U(eq)**
**H2A**	7187.01	7428.11	3418.85	21
**H2B**	8105.23	6448.74	2747.41	21
**H3A**	5337.86	7113.5	1331.68	17
**H3B**	4976.01	7760.54	2475.01	17
**H4A**	2665.93	7195.95	1573.61	17
**H4B**	3255.98	5837.8	1274.47	17
**H5A**	1196.17	4864.65	2071.21	19
**H5B**	1491.97	4549.76	3380.77	19
**H6A**	2604.57	2931.47	2506.9	20
**H6B**	3358.19	3865.15	1690.63	20
**H7A**	5765.71	2954.1	2166.69	21
**H7B**	6745.95	3519.51	3244.36	21
**H8A**	7151.48	4865.5	1821.52	20
**H8B**	5414.91	4877.92	1343.82	20
**H9A**	5456.57	2681.63	4521.82	22
**H9B**	3886.56	2149.93	3950.11	22
**H11A**	1152.83	6470.17	3880.85	18

**H11B**	1391.21	7484.88	2933.17	18
**H13A**	793.8	9527.11	3895.04	32
**H13B**	707.58	9577.41	5216.69	32
**H13C**	272.49	8320.43	4537.01	32
**H14A**	3313.34	10287.28	5419.61	21
**H14B**	4594.04	9256.3	5355.19	21
**H16**	4882.6	7570.2	6807.53	24
**H17**	4823.26	7120.08	8703.48	28
**H18**	3366.77	8349.08	9787.05	27
**H19**	1949.16	9989.17	8967.29	29
**H20**	2057.56	10471.52	7082.11	25

7.2 X-ray Diffraction Analysis of [Ga(26)]$ClO_4 \cdot H2O$

7.2.1 Data Collection

A colorless crystal with approximate dimensions 0.08 × 0.07 × 0.07 mm^3 was selected under oil under ambient conditions and attached to the tip of a MiTeGen MicroMount©. The crystal was mounted in a stream of cold nitrogen at 100(1) K and centered in the X-ray beam by using a video camera.

The crystal evaluation and data collection were performed on a Bruker D8 VENTURE Photon III four-circle diffractometer with Diamond II IµS Mo Kα (λ = 0.71073 Å) radiation and the detector to crystal distance of 5.0 cm.[4]

The initial cell constants were obtained from a 180° φ scan conducted at a 2θ = 30° angle with the exposure time of 1 second per frame. The reflections were successfully indexed by an automated indexing routine built in the APEX6 program. The final cell constants were calculated from a set of 9176 strong reflections from the actual data collection.

The data were collected by using the full sphere data collection routine to survey the reciprocal space to the extent of a full sphere to a resolution of 0.65 Å. A total of 124752 data were harvested by collecting 14 sets of frames with 0.5–1.0º scans in ω and φ with an exposure time 1–21.4 sec per frame. These highly redundant datasets were corrected for Lorentz and polarization effects. The absorption correction was based on fitting a function to the empirical transmission surface as sampled by multiple equivalent measurements.[5]

7.2.2 Structure Solution and Refinement

The systematic absences in the diffraction data were consistent for the space groups $P2_1$ and $P2_1$/m. The E-statistics strongly suggested the non-centrosymmetric space group $P2_1$ that yielded chemically reasonable and computationally stable results of refinement.[6-11]

A successful solution by intrinsic phasing provided most non-hydrogen atoms from the E-map. The remaining non-hydrogen atoms were located in an alternating series of least-squares cycles and difference Fourier maps. All non-hydrogen atoms were refined with anisotropic displacement coefficients. All hydrogen atoms were included in the structure factor calculation at idealized positions and were allowed to ride on the neighboring atoms with relative isotropic displacement coefficients.

The composition of the crystallized material is $[GaC_{19}H_{26}N_4O_5][ClO_4]\cdot H_2O$. There are two symmetry independent formula units in the asymmetric unit. The cations are enantiomers. The absolute configuration of the chiral atoms in the Ga1 fragment is N1 – *S*, N2 – *R*, N3 – *S*, and in the Ga1a fragment it is N1a – *R*, N2a – *S*, N3a – *R*.

The absolute structure was established by resonant scattering effects.

The compound crystallizes as a general twin with the two twin components related by a reflection in the *ab* plane. The minor component contribution is 0.4519(6). The model had to be treated as a general and inversion twin; the contributions of the inversion twin components refined to zero and were excluded from the final refinement.

The final least-squares refinement of 638 parameters against 17670 data resulted in residuals R (based on F^2 for $I \geq 2\sigma$) and wR (based on F^2 for all data) of 0.0239 and 0.0568, respectively. The final difference Fourier map was featureless.

7.2.3 Summary

Crystal Data for $C_{19}H_{28}ClGaN_4O_{10}$ (M =577.62 g/mol): monoclinic, space group P2$_1$ (no. 4), a = 10.4322(14) Å, b = 12.9515(16) Å, c = 17.266(2) Å, β = 90.034(8)°, V = 2332.9(5) Å^3, Z = 4, T = 100 K, μ(Mo Kα) = 1.358 mm^{-1}, $Dcalc$ = 1.645 g/cm^3, 124752 reflections measured (3.904° ≤ 2Θ ≤ 66.314°), 17670 unique (R_{int} = 0.0433, R_{sigma} = 0.0283) which were used in all calculations. The final R_1 was 0.0239 (I > 2σ(I)) and wR_2 was 0.0568 (all data).

7.2.4 Acknowledgement

The purchase of the Bruker D8 VENTURE Photon III Diamond II IμS X-ray diffractometer was partially funded by a 2024 University of Wisconsin Research Core Revitalization Program Round 3 Award to the Department of Chemistry.

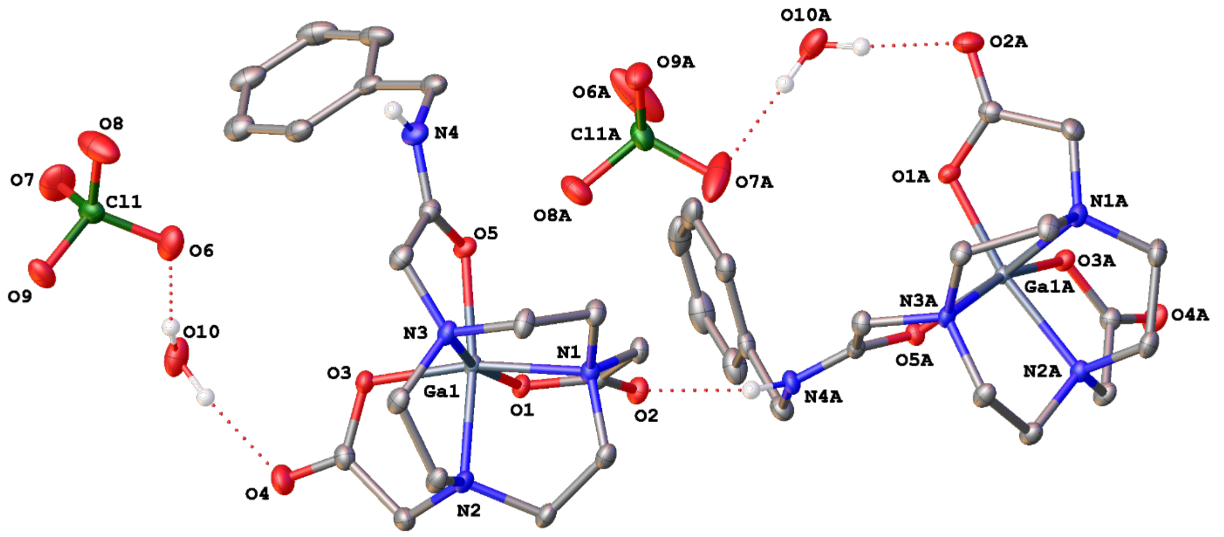

Figure S279. A molecular drawing of [Ga(26)]ClO_4 shown with 50% probability ellipsoids. All H atoms residing on C atoms are omitted.

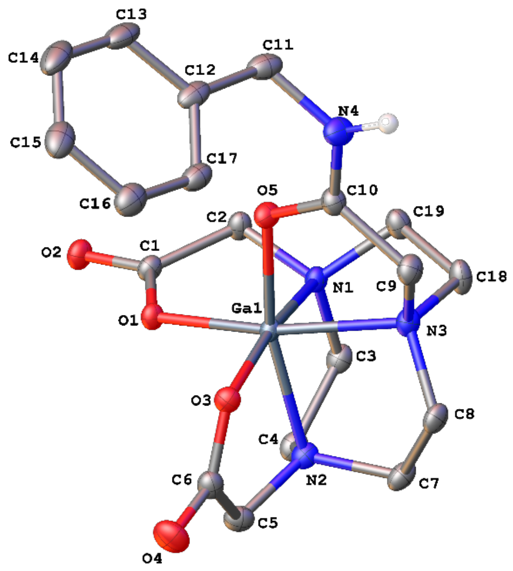

Figure S280. A molecular drawing of the Ga1 $[GaC_{19}H_{26}N_4O_5]^+$ cation shown with 50% probability ellipsoids. All H atoms residing on C atoms are omitted.

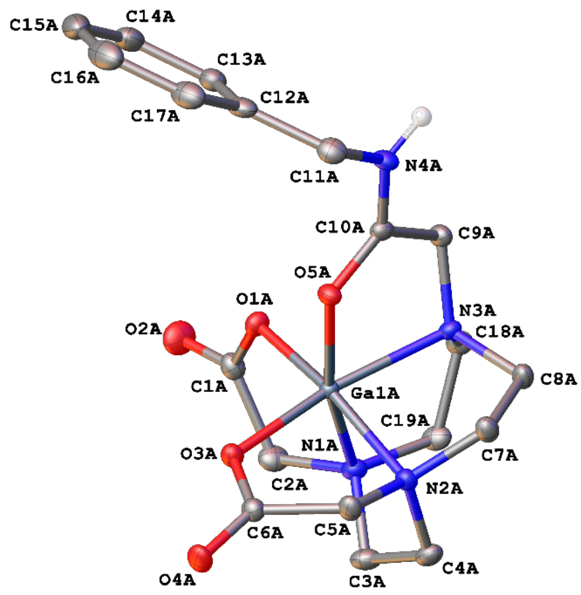

Figure S281. A molecular drawing of the Ga1a $[GaC_{19}H_{26}N_4O_5]^+$ cation shown with 50% probability ellipsoids. All H atoms residing on C atoms are omitted.

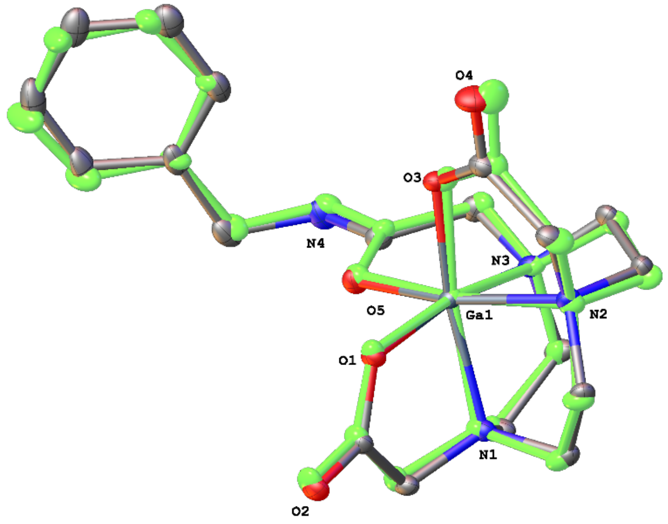

Figure S282. A molecular drawing of the two superimposed $[GaC_{19}H_{26}N_4O_5]^+$ cations shown with 50% probability ellipsoids. The Ga1a fragment is monochromatic. All H atoms are omitted. The symmetry-independent $[GaC_{19}H_{26}N_4O_5]^+$ cations are enantiomers, thus for this superposition one of them was inverted.

**Table S12 Crystal data and structure refinement for [Ga(26)]ClO_4.**	
**Identification code**	[Ga(26)]ClO_4
**Empirical formula**	$[GaC_{19}H_{26}N_4O_5][ClO_4]\cdot H_2O$
**Formula weight**	577.62
**Temperature/K**	100
**Crystal system**	monoclinic
**Space group**	$P2_1$
**a/Å**	10.4322(14)
**b/Å**	12.9515(16)
**c/Å**	17.266(2)
**α/°**	90
**β/°**	90.034(8)
**γ/°**	90
**Volume/Å^3**	2332.9(5)
**Z**	4
**ρ_{calc}g/cm^3**	1.645
**μ/mm^{-1}**	1.358
**F(000)**	1192.0
**Crystal size/mm^3**	0.08 × 0.07 × 0.07
**Radiation**	Mo Kα (λ = 0.71073)
**2Θ range for data collection/°**	3.904 to 66.314
**Index ranges**	$-15 \le h \le 16$, $-19 \le k \le 19$, $-26 \le l \le 26$
**Reflections collected**	124752
**Independent reflections**	17670 [R_{int} = 0.0433, R_{sigma} = 0.0283]
**Data/restraints/parameters**	17670/1/638
**Goodness-of-fit on F^2**	1.034
**Final R indexes [I>=2σ (I)]**	R_1 = 0.0239, wR_2 = 0.0565
**Final R indexes [all data]**	R_1 = 0.0246, wR_2 = 0.0568
**Largest diff. peak/hole / e Å^{-3}**	0.53/-0.32
**Flack parameter**	Refined to 0

Table S13 Fractional Atomic Coordinates (×10^4) and Equivalent Isotropic Displacement Parameters (Å^2×10^3) for [Ga(26)]ClO_4. U_{eq} is defined as 1/3 of the trace of the orthogonalised U_{IJ} tensor.

**Atom**	***x***	***y***	***z***	**U(eq)**
**Ga1**	9404.7(2)	3504.3(2)	4497.7(2)	11.06(5)
**O1**	9788.5(17)	4252.2(13)	5435.9(10)	13.4(3)
**O2**	9324(2)	4365.2(14)	6693.8(10)	17.2(3)
**O3**	10519.7(19)	4298.8(13)	3850.2(10)	14.4(3)
**O4**	12599(2)	4582.6(19)	3632.9(13)	25.5(4)
**O5**	7880.1(19)	4325.3(15)	4208.4(11)	15.6(3)
**N1**	8527(2)	2480.6(17)	5255.7(12)	13.5(4)
**N2**	10912(2)	2467.9(15)	4517.1(13)	15.1(3)

**N3**	8638(2)	2530.1(18)	3633.8(13)	14.9(4)
**N4**	6416(2)	4616(2)	3264.7(14)	20.6(4)
**C1**	9167(3)	3965.3(19)	6054.0(14)	13.8(4)
**C2**	8185(3)	3111(2)	5937.5(15)	15.5(4)
**C3**	9478(2)	1643.7(17)	5458.6(15)	16.5(4)
**C4**	10852(3)	1971(2)	5293.7(14)	16.7(5)
**C5**	12059(3)	3126(2)	4401.0(16)	18.2(5)
**C6**	11732(3)	4071(2)	3914.6(15)	17.4(5)
**C7**	10722(3)	1690(2)	3882.6(15)	17.8(5)
**C8**	9785(3)	2061.6(19)	3262.6(14)	16.9(4)
**C9**	7918(3)	3211(2)	3100.7(16)	18.1(5)
**C10**	7377(3)	4096(2)	3562.6(15)	16.0(4)
**C11**	5904(3)	5538(2)	3632.3(16)	22.2(5)
**C12**	6607(3)	6513(2)	3409.3(15)	20.6(5)
**C13**	6161(3)	7465(3)	3685.3(18)	27.1(6)
**C14**	6757(3)	8377(3)	3482.9(19)	31.0(7)
**C15**	7823(3)	8374(2)	3007.4(19)	27.2(6)
**C16**	8288(3)	7433(3)	2735.2(17)	25.0(6)
**C17**	7682(3)	6514(2)	2937.1(16)	20.7(5)
**C18**	7796(3)	1734(2)	4008.0(15)	18.4(5)
**C19**	7395(3)	2057(2)	4824.0(15)	17.7(5)
**Ga1A**	5372.0(2)	3640.4(2)	9498.9(2)	9.82(5)
**O1A**	4357.3(18)	4508.6(14)	8843.0(10)	14.4(3)
**O2A**	2346(2)	4974.3(19)	8597.7(14)	24.9(4)
**O3A**	5158.4(18)	4466.7(14)	10424.2(11)	14.1(3)
**O4A**	5672(2)	4558.7(15)	11676.7(11)	19.7(4)
**O5A**	7004.0(17)	4275.4(14)	9189.8(11)	13.2(3)
**N1A**	3707(2)	2762.7(16)	9559.6(13)	14.7(4)
**N2A**	6124(2)	2575.7(17)	10272.6(12)	13.3(4)
**N3A**	5908(2)	2577.7(16)	8649.7(12)	12.4(4)
**N4A**	8436(2)	4384.8(17)	8221.8(12)	14.6(4)
**C1A**	3123(3)	4404(2)	8908.2(15)	16.5(4)
**C2A**	2666(2)	3528(2)	9429.7(16)	18.5(4)
**C3A**	3746(3)	2315(2)	10351.6(14)	18.4(5)
**C4A**	5070(3)	1853.3(18)	10510.0(16)	17.5(4)
**C5A**	6606(3)	3203(2)	10927.1(14)	15.5(5)
**C6A**	5745(3)	4140.9(18)	11039.6(14)	14.4(4)
**C7A**	7150(3)	2028(2)	9838.7(15)	16.3(4)
**C8A**	6651(3)	1720.5(19)	9034.1(14)	15.9(4)
**C9A**	6671(3)	3162.7(19)	8087.3(15)	14.4(4)
**C10A**	7413(2)	3986.0(19)	8529.5(14)	13.0(4)
**C11A**	9199(3)	5168(2)	8614.3(16)	18.2(5)
**C12A**	8782(3)	6266(2)	8436.1(15)	18.0(5)
**C13A**	7757(3)	6514(2)	7953.0(16)	19.1(5)
**C14A**	7460(4)	7550(3)	7814.5(18)	26.5(6)

**C15A**	8178(4)	8334(2)	8161.7(19)	31.5(8)
**C16A**	9192(4)	8081(3)	8649(2)	32.0(7)
**C17A**	9494(3)	7055(2)	8787.5(17)	25.6(6)
**C18A**	4682(3)	2197.4(19)	8307.6(14)	15.8(4)
**C19A**	3737(3)	1944(2)	8949.9(16)	17.2(5)
**Cl1**	9737.9(7)	5818.6(5)	916.0(4)	20.36(12)
**O6**	9704(3)	5445.2(19)	1703.6(13)	30.8(5)
**O7**	9874(3)	6925.7(18)	928.8(17)	39.0(6)
**O8**	8559(2)	5525(3)	535.3(18)	45.3(7)
**O9**	10802(2)	5369.5(15)	514.2(15)	24.0(4)
**Cl1A**	5692.2(7)	5573.3(6)	6021.2(4)	23.00(13)
**O6A**	5483(3)	6638(3)	6251(3)	68.6(12)
**O7A**	5746(4)	4963(3)	6703.7(17)	61.5(11)
**O8A**	6866(2)	5508(2)	5596.8(16)	30.9(5)
**O9A**	4634(2)	5252.7(19)	5540.9(15)	28.8(4)
**O10**	11797(3)	6216.0(17)	2685.8(13)	28.4(5)
**O10A**	3681(3)	6370.7(19)	7664.0(13)	29.5(5)

Table S14 Anisotropic Displacement Parameters ($Å^2×10^3$) for [Ga(26)]ClO_4. The Anisotropic displacement factor exponent takes the form: $2\pi^2[h^2a^{*2}U_{11}+2hka^*b^*U_{12}+…]$.

Atom	U_{11}	U_{22}	U_{33}	U_{23}	U_{13}	U_{12}
**Ga1**	14.09(10)	10.56(12)	8.54(10)	0.03(10)	-0.1(1)	-0.28(9)
**O1**	16.9(8)	14.4(7)	8.9(7)	-0.2(6)	0.2(7)	-1.6(6)
**O2**	22.9(9)	18.0(8)	10.9(7)	-1.8(6)	0.6(7)	-1.1(7)
**O3**	16.4(8)	15.0(7)	11.8(7)	2.2(6)	1.5(7)	-0.5(7)
**O4**	20.9(10)	30.9(11)	24.6(10)	5.6(9)	3.6(9)	-6.8(9)
**O5**	17.5(8)	18.1(8)	11.2(8)	-0.8(6)	-2.7(7)	2.6(7)
**N1**	16.1(9)	13.4(9)	10.9(8)	-0.6(7)	0.5(7)	-1.3(7)
**N2**	18.3(9)	14.5(8)	12.4(8)	2.6(8)	0.1(9)	2.0(7)
**N3**	20.0(10)	14.4(9)	10.3(8)	0.5(7)	-0.3(8)	-1.0(8)
**N4**	20.7(11)	26.8(11)	14.1(9)	-1.6(8)	-4.2(9)	3.8(9)
**C1**	17.2(11)	14.5(9)	9.7(9)	-0.1(8)	0.6(8)	1.6(8)
**C2**	19.5(11)	15.7(10)	11.1(10)	-0.4(8)	2.4(9)	-3.0(9)
**C3**	22.8(11)	13.6(8)	13.1(9)	1.8(8)	0.5(11)	0.5(8)
**C4**	22.9(12)	16.1(10)	11.0(9)	2.3(8)	-0.6(9)	2.5(9)
**C5**	16.6(10)	22.7(11)	15.2(11)	1.4(9)	2.2(9)	1.2(9)
**C6**	19.1(11)	19.0(11)	14.1(10)	-0.7(9)	1.8(9)	-2.3(9)
**C7**	25.1(13)	15.9(10)	12.5(10)	-0.3(8)	2.2(9)	4.9(9)
**C8**	24.2(12)	16.3(10)	10.4(9)	-2.3(8)	0.4(9)	1.9(9)
**C9**	23.3(13)	18.3(11)	12.7(10)	-0.7(8)	-2.9(9)	0.2(9)
**C10**	17.7(11)	17.4(10)	12.8(10)	0.2(8)	-1.4(9)	-1.6(9)
**C11**	20.3(12)	31.1(14)	15.3(11)	0.3(10)	-1.4(9)	9.5(11)
**C12**	23.1(13)	25.8(13)	12.8(10)	-1.6(9)	-5.1(10)	11.8(11)
**C13**	23.5(13)	36.3(16)	21.7(13)	-7.8(12)	-4.9(11)	16.4(12)
**C14**	34.1(16)	28.8(15)	30.3(14)	-11.3(13)	-12.6(13)	16.1(13)

**C15**	33.5(16)	23.9(14)	24.3(13)	-3.2(11)	-13.5(12)	7.3(12)
**C16**	29.0(15)	28.8(14)	17.3(12)	-2.8(11)	-5.0(11)	5.8(12)
**C17**	23.0(13)	23.7(12)	15.3(11)	-2.5(10)	-2.6(10)	8.8(11)
**C18**	25.8(13)	16.4(11)	13.1(10)	0.4(8)	-1.4(10)	-7.5(10)
**C19**	19.6(12)	20.0(11)	13.5(10)	0.2(9)	-0.4(9)	-4.9(9)
**Ga1A**	11.37(9)	9.39(11)	8.69(9)	-0.1(1)	-0.12(9)	-0.44(8)
**O1A**	14.8(8)	13.9(7)	14.5(8)	1.7(6)	-2.6(7)	1.5(6)
**O2A**	17.0(9)	28.2(11)	29.6(11)	3.8(9)	-3.9(9)	4.7(8)
**O3A**	17.1(8)	14.4(7)	10.8(7)	-0.7(6)	-1.0(7)	2.3(6)
**O4A**	30.7(11)	18.4(8)	10.1(7)	-3.9(6)	-1.4(7)	3.7(8)
**O5A**	13.7(8)	13.6(7)	12.2(8)	-1.4(6)	1.8(6)	-2.3(6)
**N1A**	16.9(9)	15.4(8)	11.9(9)	1.6(8)	0.2(8)	-1.8(7)
**N2A**	15.7(10)	13.6(9)	10.5(8)	-0.7(7)	0.5(7)	0.2(7)
**N3A**	15.2(9)	10.7(8)	11.1(8)	1.8(7)	0.3(7)	-1.7(7)
**N4A**	14.0(9)	17.9(9)	12.0(9)	-0.1(7)	3.2(7)	-2.3(7)
**C1A**	15.8(11)	18.1(11)	15.5(11)	-2.9(9)	-0.8(9)	1.6(9)
**C2A**	12.5(8)	22.9(11)	20.0(10)	1.9(11)	0.8(9)	-1.9(10)
**C3A**	21.5(12)	20.4(11)	13.4(11)	2.7(8)	1.6(9)	-5.1(9)
**C4A**	24.8(11)	14.8(9)	12.9(9)	2.6(9)	0.2(11)	-2.3(8)
**C5A**	21.1(12)	15.2(10)	10.1(10)	-2.2(8)	-2.5(9)	1.8(9)
**C6A**	16.6(10)	13.8(9)	12.8(10)	-1.6(8)	0.0(8)	-1.0(8)
**C7A**	19.8(12)	16.6(10)	12.6(10)	-0.8(8)	-0.9(9)	3.8(9)
**C8A**	22.3(12)	13.4(10)	11.9(10)	-1.4(8)	-0.3(9)	3.5(9)
**C9A**	19.1(11)	13.1(10)	11.1(9)	-2.1(8)	1.4(9)	-1.9(9)
**C10A**	14.1(10)	13.4(9)	11.7(10)	2.5(8)	0.6(8)	0.3(8)
**C11A**	14.5(11)	21.9(11)	18.2(11)	-1.0(9)	0.8(9)	-3.9(9)
**C12A**	19.4(11)	21.3(11)	13.3(10)	-0.6(9)	5.0(9)	-6.8(9)
**C13A**	24.5(14)	18.5(11)	14.4(11)	-0.5(9)	5.4(10)	-1.2(10)
**C14A**	36.1(17)	26.3(14)	17.0(12)	3.1(11)	9.0(12)	5.1(12)
**C15A**	50(2)	17.6(12)	27.0(15)	1.4(11)	17.9(15)	0.0(12)
**C16A**	43.1(19)	26.1(14)	26.9(14)	-8.2(11)	11.9(14)	-15.0(13)
**C17A**	30.2(15)	26.2(12)	20.4(12)	-5.2(10)	5.5(11)	-11.8(12)
**C18A**	19.5(11)	16.0(9)	12.0(9)	-0.8(8)	-2.2(9)	-2.7(9)
**C19A**	19.3(11)	16.9(10)	15.3(11)	-0.7(9)	-1.8(9)	-7.0(9)
**Cl1**	19.3(3)	23.6(3)	18.2(3)	-2.7(2)	1.5(2)	5.0(2)
**O6**	41.8(14)	31.7(11)	18.8(9)	0.2(8)	6.7(10)	6.1(11)
**O7**	55.3(17)	18.3(10)	43.4(14)	1.3(10)	8.7(13)	14.6(11)
**O8**	22.5(11)	77(2)	36.0(13)	-17.7(16)	-2.7(12)	4.1(12)
**O9**	21.5(9)	20.3(8)	30.3(10)	-3.1(9)	10.0(10)	2.1(7)
**Cl1A**	18.7(3)	29.6(3)	20.7(3)	-1.2(2)	3.5(2)	-6.1(2)
**O6A**	43.7(18)	45.4(17)	117(3)	-43(2)	40(2)	-15.6(15)
**O7A**	53.8(19)	98(3)	32.5(14)	33.2(17)	-20.1(14)	-43(2)
**O8A**	18.1(9)	42.8(13)	31.9(12)	-4.7(11)	5.7(9)	0.1(9)
**O9A**	21.0(9)	39.1(11)	26.4(10)	9.7(10)	-3.5(10)	-3.8(9)
**O10**	46.1(14)	18.8(9)	20.2(10)	1.8(8)	4.4(10)	-7.5(9)

**O10A**	43.1(14)	27.8(11)	17.6(9)	4.4(8)	0.6(9)	10.6(10)

Table S15 Bond Lengths for [Ga(26)]ClO$_4$.

Atom	Atom	Length/Å	Atom	Atom	Length/Å
**Ga1**	O1	1.9292(18)	Ga1A	N2A	2.074(2)
**Ga1**	O3	1.9140(18)	Ga1A	N3A	2.088(2)
**Ga1**	O5	1.9771(19)	O1A	C1A	1.299(3)
**Ga1**	N1	2.076(2)	O2A	C1A	1.221(3)
**Ga1**	N2	2.068(2)	O3A	C6A	1.296(3)
**Ga1**	N3	2.111(2)	O4A	C6A	1.228(3)
**O1**	C1	1.303(3)	O5A	C10A	1.274(3)
**O2**	C1	1.231(3)	N1A	C2A	1.488(3)
**O3**	C6	1.303(3)	N1A	C3A	1.486(3)
**O4**	C6	1.222(3)	N1A	C19A	1.494(3)
**O5**	C10	1.268(3)	N2A	C4A	1.501(3)
**N1**	C2	1.476(3)	N2A	C5A	1.480(3)
**N1**	C3	1.511(3)	N2A	C7A	1.487(3)
**N1**	C19	1.500(4)	N3A	C8A	1.508(3)
**N2**	C4	1.489(3)	N3A	C9A	1.467(3)
**N2**	C5	1.482(3)	N3A	C18A	1.492(3)
**N2**	C7	1.501(3)	N4A	C10A	1.299(3)
**N3**	C8	1.487(4)	N4A	C11A	1.457(3)
**N3**	C9	1.479(3)	C1A	C2A	1.525(4)
**N3**	C18	1.501(3)	C3A	C4A	1.530(4)
**N4**	C10	1.312(4)	C5A	C6A	1.523(4)
**N4**	C11	1.455(4)	C7A	C8A	1.536(4)
**C1**	C2	1.522(4)	C9A	C10A	1.523(4)
**C3**	C4	1.522(4)	C11A	C12A	1.518(4)
**C5**	C6	1.524(4)	C12A	C13A	1.393(4)
**C7**	C8	1.527(4)	C12A	C17A	1.401(4)
**C9**	C10	1.507(4)	C13A	C14A	1.398(4)
**C11**	C12	1.510(5)	C14A	C15A	1.397(5)
**C12**	C13	1.401(4)	C15A	C16A	1.391(6)
**C12**	C17	1.387(4)	C16A	C17A	1.386(5)
**C13**	C14	1.380(5)	C18A	C19A	1.520(4)
**C14**	C15	1.383(5)	Cl1	O6	1.444(2)
**C15**	C16	1.394(4)	Cl1	O7	1.441(3)
**C16**	C17	1.392(5)	Cl1	O8	1.445(3)
**C18**	C19	1.528(4)	Cl1	O9	1.433(2)
**Ga1A**	O1A	1.9148(18)	Cl1A	O6A	1.452(3)
**Ga1A**	O3A	1.9359(18)	Cl1A	O7A	1.420(3)
**Ga1A**	O5A	1.9649(18)	Cl1A	O8A	1.430(2)
**Ga1A**	N1A	2.078(2)	Cl1A	O9A	1.442(2)

Table S16 Bond Angles for [Ga(26)]ClO$_4$.

Atom	Atom	Atom	Angle/°	Atom	Atom	Atom	Angle/°
**O1**	Ga1	O5	96.24(8)	O3A	Ga1A	N1A	99.45(8)
**O1**	Ga1	N1	83.26(8)	O3A	Ga1A	N2A	83.09(8)
**O1**	Ga1	N2	98.90(8)	O3A	Ga1A	N3A	167.20(8)
**O1**	Ga1	N3	166.38(8)	O5A	Ga1A	N1A	165.19(8)
**O3**	Ga1	O1	95.43(8)	O5A	Ga1A	N2A	97.23(9)
**O3**	Ga1	O5	93.00(8)	O5A	Ga1A	N3A	81.53(8)
**O3**	Ga1	N1	168.51(9)	N1A	Ga1A	N3A	84.19(9)
**O3**	Ga1	N2	84.03(8)	N2A	Ga1A	N1A	85.39(9)
**O3**	Ga1	N3	97.98(8)	N2A	Ga1A	N3A	84.99(8)
**O5**	Ga1	N1	98.49(9)	C1A	O1A	Ga1A	115.77(17)
**O5**	Ga1	N2	164.78(8)	C6A	O3A	Ga1A	116.23(16)
**O5**	Ga1	N3	80.70(9)	C10A	O5A	Ga1A	114.26(16)
**N1**	Ga1	N3	84.07(8)	C2A	N1A	Ga1A	103.79(15)
**N2**	Ga1	N1	84.90(9)	C2A	N1A	C19A	112.4(2)
**N2**	Ga1	N3	84.92(9)	C3A	N1A	Ga1A	103.75(16)
**C1**	O1	Ga1	116.20(16)	C3A	N1A	C2A	114.7(2)
**C6**	O3	Ga1	114.74(16)	C3A	N1A	C19A	111.8(2)
**C10**	O5	Ga1	115.42(18)	C19A	N1A	Ga1A	109.58(16)
**C2**	N1	Ga1	104.87(15)	C4A	N2A	Ga1A	108.28(16)
**C2**	N1	C3	111.8(2)	C5A	N2A	Ga1A	104.77(15)
**C2**	N1	C19	114.1(2)	C5A	N2A	C4A	112.5(2)
**C3**	N1	Ga1	108.32(16)	C5A	N2A	C7A	113.7(2)
**C19**	N1	Ga1	105.53(15)	C7A	N2A	Ga1A	105.36(15)
**C19**	N1	C3	111.7(2)	C7A	N2A	C4A	111.6(2)
**C4**	N2	Ga1	105.25(16)	C8A	N3A	Ga1A	108.30(15)
**C4**	N2	C7	111.19(19)	C9A	N3A	Ga1A	105.67(15)
**C5**	N2	Ga1	103.80(15)	C9A	N3A	C8A	113.1(2)
**C5**	N2	C4	113.9(2)	C9A	N3A	C18A	112.0(2)
**C5**	N2	C7	113.2(2)	C18A	N3A	Ga1A	105.42(15)
**C7**	N2	Ga1	108.85(16)	C18A	N3A	C8A	111.8(2)
**C8**	N3	Ga1	104.11(16)	C10A	N4A	C11A	122.4(2)
**C8**	N3	C18	112.1(2)	O1A	C1A	C2A	116.1(2)
**C9**	N3	Ga1	105.98(16)	O2A	C1A	O1A	123.8(3)
**C9**	N3	C8	112.5(2)	O2A	C1A	C2A	120.1(2)
**C9**	N3	C18	112.4(2)	N1A	C2A	C1A	110.83(19)
**C18**	N3	Ga1	109.15(15)	N1A	C3A	C4A	109.9(2)
**C10**	N4	C11	122.1(2)	N2A	C4A	C3A	111.6(2)
**O1**	C1	C2	115.7(2)	N2A	C5A	C6A	109.6(2)
**O2**	C1	O1	123.3(2)	O3A	C6A	C5A	115.7(2)
**O2**	C1	C2	120.9(2)	O4A	C6A	O3A	124.1(2)
**N1**	C2	C1	110.1(2)	O4A	C6A	C5A	120.2(2)
**N1**	C3	C4	112.07(19)	N2A	C7A	C8A	109.6(2)
**N2**	C4	C3	109.2(2)	N3A	C8A	C7A	112.4(2)
**N2**	C5	C6	110.8(2)	N3A	C9A	C10A	107.8(2)

**O3**	C6	C5	116.5(2)	O5A	C10A	N4A	121.6(2)
**O4**	C6	O3	124.2(3)	O5A	C10A	C9A	118.9(2)
**O4**	C6	C5	119.3(3)	N4A	C10A	C9A	119.4(2)
**N2**	C7	C8	112.6(2)	N4A	C11A	C12A	113.7(2)
**N3**	C8	C7	109.9(2)	C13A	C12A	C11A	123.8(2)
**N3**	C9	C10	108.3(2)	C13A	C12A	C17A	119.9(3)
**O5**	C10	N4	122.7(3)	C17A	C12A	C11A	116.3(3)
**O5**	C10	C9	119.2(2)	C12A	C13A	C14A	119.6(3)
**N4**	C10	C9	118.0(2)	C15A	C14A	C13A	120.3(3)
**N4**	C11	C12	113.4(2)	C16A	C15A	C14A	119.8(3)
**C13**	C12	C11	119.2(3)	C17A	C16A	C15A	120.2(3)
**C17**	C12	C11	122.9(3)	C16A	C17A	C12A	120.3(3)
**C17**	C12	C13	117.9(3)	N3A	C18A	C19A	109.8(2)
**C14**	C13	C12	121.2(3)	N1A	C19A	C18A	112.0(2)
**C13**	C14	C15	120.7(3)	O6	Cl1	O8	108.60(19)
**C14**	C15	C16	118.9(3)	O7	Cl1	O6	108.74(16)
**C17**	C16	C15	120.3(3)	O7	Cl1	O8	110.6(2)
**C12**	C17	C16	121.0(3)	O9	Cl1	O6	109.87(15)
**N3**	C18	C19	111.7(2)	O9	Cl1	O7	109.56(15)
**N1**	C19	C18	110.1(2)	O9	Cl1	O8	109.41(15)
**O1A**	Ga1A	O3A	95.72(8)	O7A	Cl1A	O6A	107.9(3)
**O1A**	Ga1A	O5A	94.15(8)	O7A	Cl1A	O8A	111.0(2)
**O1A**	Ga1A	N1A	83.65(8)	O7A	Cl1A	O9A	110.31(16)
**O1A**	Ga1A	N2A	168.62(9)	O8A	Cl1A	O6A	108.98(18)
**O1A**	Ga1A	N3A	96.88(8)	O8A	Cl1A	O9A	110.13(14)
**O3A**	Ga1A	O5A	95.34(8)	O9A	Cl1A	O6A	108.4(2)

Table S17 Hydrogen Bonds for [Ga(26)]ClO_4.

**D**	**H**	**A**	**d(D-H)/Å**	**d(H-A)/Å**	**d(D-A)/Å**	**D-H-A/°**
**N4**	H4	O4A[1]	0.88	2.04	2.850(3)	152.1
**N4A**	H4AA	O2	0.88	1.98	2.797(3)	154.6
**O10**	H10A	O4	0.87	1.94	2.801(3)	171.5
**O10**	H10B	O6	0.87	2.08	2.939(4)	170.9
**O10A**	H10C	O7A	0.87	2.44	3.274(5)	161.9
**O10A**	H10D	O2A	0.87	1.93	2.795(4)	174.9

[1]+X,+Y,-1+Z

Table S18 Torsion Angles for [Ga(26)]ClO_4.

**A**	**B**	**C**	**D**	**Angle/°**	**A**	**B**	**C**	**D**	**Angle/°**
**Ga1**	O1	C1	O2	179.2(2)	Ga1A	O1A	C1A	O2A	171.5(2)
**Ga1**	O1	C1	C2	-2.6(3)	Ga1A	O1A	C1A	C2A	-6.4(3)
**Ga1**	O3	C6	O4	-169.5(2)	Ga1A	O3A	C6A	O4A	-177.5(2)
**Ga1**	O3	C6	C5	8.1(3)	Ga1A	O3A	C6A	C5A	4.8(3)
**Ga1**	O5	C10	N4	-175.2(2)	Ga1A	O5A	C10A	N4A	175.52(19)
**Ga1**	O5	C10	C9	3.0(3)	Ga1A	O5A	C10A	C9A	-3.6(3)

**Ga1**	N1	C2	C1	-32.3(2)	Ga1A	N1A	C2A	C1A	30.4(2)
**Ga1**	N1	C3	C4	19.4(2)	Ga1A	N1A	C3A	C4A	-47.9(2)
**Ga1**	N1	C19	C18	46.8(2)	Ga1A	N1A	C19A	C18A	-19.7(3)
**Ga1**	N2	C4	C3	47.2(2)	Ga1A	N2A	C4A	C3A	-19.5(2)
**Ga1**	N2	C5	C6	-30.0(2)	Ga1A	N2A	C5A	C6A	33.9(2)
**Ga1**	N2	C7	C8	18.8(3)	Ga1A	N2A	C7A	C8A	-46.3(2)
**Ga1**	N3	C8	C7	46.1(2)	Ga1A	N3A	C8A	C7A	-17.9(3)
**Ga1**	N3	C9	C10	-32.1(3)	Ga1A	N3A	C9A	C10A	34.0(2)
**Ga1**	N3	C18	C19	18.5(3)	Ga1A	N3A	C18A	C19A	-45.6(2)
**O1**	C1	C2	N1	25.1(3)	O1A	C1A	C2A	N1A	-18.3(3)
**O2**	C1	C2	N1	-156.6(2)	O2A	C1A	C2A	N1A	163.7(2)
**N1**	C3	C4	N2	-45.4(3)	N1A	C3A	C4A	N2A	46.6(3)
**N2**	C5	C6	O3	16.9(3)	N2A	C5A	C6A	O3A	-27.6(3)
**N2**	C5	C6	O4	-165.4(3)	N2A	C5A	C6A	O4A	154.7(2)
**N2**	C7	C8	N3	-45.0(3)	N2A	C7A	C8A	N3A	43.9(3)
**N3**	C9	C10	O5	21.4(3)	N3A	C9A	C10A	O5A	-22.3(3)
**N3**	C9	C10	N4	-160.4(2)	N3A	C9A	C10A	N4A	158.6(2)
**N3**	C18	C19	N1	-44.3(3)	N3A	C18A	C19A	N1A	44.5(3)
**N4**	C11	C12	C13	176.1(3)	N4A	C11A	C12A	C13A	0.9(4)
**N4**	C11	C12	C17	-3.7(4)	N4A	C11A	C12A	C17A	-179.0(2)
**C2**	N1	C3	C4	-95.6(2)	C2A	N1A	C3A	C4A	-160.4(2)
**C2**	N1	C19	C18	161.4(2)	C2A	N1A	C19A	C18A	95.2(3)
**C3**	N1	C2	C1	84.8(2)	C3A	N1A	C2A	C1A	142.8(2)
**C3**	N1	C19	C18	-70.7(3)	C3A	N1A	C19A	C18A	-134.1(2)
**C4**	N2	C5	C6	-143.9(2)	C4A	N2A	C5A	C6A	-83.5(3)
**C4**	N2	C7	C8	134.3(2)	C4A	N2A	C7A	C8A	70.9(3)
**C5**	N2	C4	C3	160.2(2)	C5A	N2A	C4A	C3A	95.8(3)
**C5**	N2	C7	C8	-96.0(3)	C5A	N2A	C7A	C8A	-160.5(2)
**C7**	N2	C4	C3	-70.5(3)	C7A	N2A	C4A	C3A	-135.0(2)
**C7**	N2	C5	C6	87.8(3)	C7A	N2A	C5A	C6A	148.4(2)
**C8**	N3	C9	C10	-145.2(2)	C8A	N3A	C9A	C10A	-84.3(2)
**C8**	N3	C18	C19	133.3(2)	C8A	N3A	C18A	C19A	71.8(3)
**C9**	N3	C8	C7	160.4(2)	C9A	N3A	C8A	C7A	98.9(3)
**C9**	N3	C18	C19	-98.8(3)	C9A	N3A	C18A	C19A	-160.0(2)
**C10**	N4	C11	C12	86.1(3)	C10A	N4A	C11A	C12A	-90.4(3)
**C11**	N4	C10	O5	3.9(4)	C11A	N4A	C10A	O5A	1.5(4)
**C11**	N4	C10	C9	-174.3(3)	C11A	N4A	C10A	C9A	-179.4(2)
**C11**	C12	C13	C14	-178.6(3)	C11A	C12A	C13A	C14A	-179.0(3)
**C11**	C12	C17	C16	178.9(3)	C11A	C12A	C17A	C16A	179.2(3)
**C12**	C13	C14	C15	-0.8(5)	C12A	C13A	C14A	C15A	-0.4(5)
**C13**	C12	C17	C16	-0.9(4)	C13A	C12A	C17A	C16A	-0.7(4)
**C13**	C14	C15	C16	0.0(5)	C13A	C14A	C15A	C16A	-0.2(5)
**C14**	C15	C16	C17	0.3(4)	C14A	C15A	C16A	C17A	0.4(5)
**C15**	C16	C17	C12	0.2(4)	C15A	C16A	C17A	C12A	0.1(5)
**C17**	C12	C13	C14	1.2(4)	C17A	C12A	C13A	C14A	0.9(4)

**C18**	N3	C8	C7	-71.8(3)	C18A	N3A	C8A	C7A	-133.6(2)
**C18**	N3	C9	C10	87.1(3)	C18A	N3A	C9A	C10A	148.2(2)
**C19**	N1	C2	C1	-147.3(2)	C19A	N1A	C2A	C1A	-88.0(3)
**C19**	N1	C3	C4	135.2(2)	C19A	N1A	C3A	C4A	70.1(3)

Table S19 Hydrogen Atom Coordinates (Å×10^4) and Isotropic Displacement Parameters (Å^2×10^3) for [Ga(26)]ClO_4.

Atom	*x*	*y*	*z*	U(eq)
**H4**	6070.21	4399.63	2828.88	25
**H2A**	8154.8	2667.07	6404.12	19
**H2B**	7324.44	3417.4	5861.76	19
**H3A**	9277.22	1015.18	5155.57	20
**H3B**	9393.45	1470.42	6014.69	20
**H4A**	11146.3	2462.33	5695.31	20
**H4B**	11422.21	1359.61	5305.96	20
**H5A**	12736.18	2720.52	4138.5	22
**H5B**	12394.86	3349.63	4910.44	22
**H7A**	10395.99	1039.7	4110.21	21
**H7B**	11559.03	1540.64	3637.58	21
**H8A**	10209.36	2578.45	2927.13	20
**H8B**	9519.58	1471.99	2934.62	20
**H9A**	7216.07	2820.33	2849.95	22
**H9B**	8495.54	3476.18	2691.43	22
**H11A**	5949.54	5452.87	4201.42	27
**H11B**	4989.57	5611.91	3489.01	27
**H13**	5435.98	7482.2	4017.64	33
**H14**	6430.55	9013.58	3672.22	37
**H15**	8231.36	9003.2	2868.69	33
**H16**	9021.91	7418.6	2410.09	30
**H17**	8009.9	5877.82	2748.61	25
**H18A**	8261.71	1068.89	4034.25	22
**H18B**	7020.58	1630.09	3686.57	22
**H19A**	6715.34	2588.16	4792.71	21
**H19B**	7045.57	1452.7	5104.59	21
**H4AA**	8675.85	4174.18	7759.62	18
**H2AA**	2384.8	3813.96	9933.55	22
**H2AB**	1921.87	3180.65	9186.79	22
**H3AA**	3083.52	1771.73	10400	22
**H3AB**	3558.51	2860.02	10737.6	22
**H4AB**	5151.41	1698.48	11069.36	21
**H4AC**	5158.26	1196.28	10221.87	21
**H5AA**	7493.66	3431.18	10819.46	19
**H5AB**	6614.48	2782.22	11405.62	19
**H7AA**	7415.51	1403.05	10127.33	20
**H7AB**	7906.35	2483.69	9783.95	20

**H8AA**	7386.2	1528.89	8701.46	19
**H8AB**	6092.04	1106.5	9084.8	19
**H9AA**	6102.74	3489.66	7698.84	17
**H9AB**	7271.36	2696.51	7813.91	17
**H11C**	10107.72	5084.56	8461.2	22
**H11D**	9143.58	5054.28	9180.2	22
**H13A**	7263.81	5981.9	7719.19	23
**H14A**	6765.67	7722.28	7482.51	32
**H15A**	7975.5	9037.11	8064.95	38
**H16A**	9678.65	8612.43	8888.73	38
**H17A**	10187.19	6886.5	9121.62	31
**H18C**	4847.4	1572.39	7993.09	19
**H18D**	4314.32	2733.26	7964.12	19
**H19C**	2868.99	1866.68	8724.99	21
**H19D**	3977.59	1277.56	9189.87	21
**H10A**	12087.23	5683.98	2939.11	43
**H10B**	11243.4	5958.61	2361.8	43
**H10C**	4357.86	6098.98	7452.11	44
**H10D**	3309.49	5914.55	7957.92	44

7.3 X-ray Diffraction Analysis of Ga(26)·4H_2O

7.3.1 Data Collection

A colorless crystal with approximate dimensions 0.103 x 0.035 x 0.024 mm^3 was selected under oil under ambient conditions and attached to the tip of a MiTeGen MicroMount©. The crystal was mounted in a stream of cold nitrogen at 100(1) K and centered in the X-ray beam by using a video camera.

The crystal evaluation and data collection were performed on a Bruker D8 VENTURE PhotonIII four-circle diffractometer with Cu Kα (λ = 1.54178 Å) radiation and the detector to crystal distance of 5.0 cm.[4]

The initial cell constants were obtained from a 180° φ scan conducted at a 2θ = 50° angle with the exposure time of 1 second per frame. The reflections were successfully indexed by an automated indexing routine built in the APEX6 program. The final cell constants were calculated from a set of 9293 strong reflections from the actual data collection.

The data were collected by using the full sphere data collection routine to survey the reciprocal space to the extent of a full sphere to a resolution of 0.78 Å. A total of 88232 data were harvested by collecting 52 sets of frames with 0.8° scans in ω and φ with an exposure time 2–15 sec per frame. These highly redundant datasets were corrected for Lorentz and polarization effects. The absorption correction was based on fitting a function to the empirical transmission surface as sampled by multiple equivalent measurements.[5]

7.3.2 Structure Solution and Refinement

The systematic absences in the diffraction data were uniquely consistent for the space group $P2_12_12_1$ that yielded chemically reasonable and computationally stable results of refinement.[6-11]

A successful solution by intrinsic phasing provided most non-hydrogen atoms from the *E*-map. The remaining non-hydrogen atoms were located with an alternating series of least-squares cycles and difference Fourier maps. The atomic form factors were determined by DFT calculations, using the R2SCAN hybrid functional and the def2-TZVP basis set, in the NoSpherA2 extension of the olex2.refine program.[12-13] All non-hydrogen atoms bonded to oxygen atoms were refined with atomic distance restraints and relative isotropic displacement coefficients.

The asymmetric unit contains one $C_{19}H_{25}N_4O_5Ga$ molecule with four water molecules for an overall formula of $C_{19}H_{25}N_4O_5Ga\cdot 4H_2O$.

The absolute structure was unequivocally established by resonant scattering effects. The N1 atom is in the *S* configuration and N2 and N3 atoms are in the *R* configuration.

The final least-squares refinement of 322 parameters against 4820 data resulted in residuals *R* (based on F^2 for $I\geq 2\sigma$) and *wR* (based on F^2 for all data) of 0.0213 and 0.0507, respectively. The final difference Fourier map was featureless.

7.3.3 Summary

Crystal Data for $C_{19}H_{33}GaN_4O_9$ (M =531.219 g/mol): orthorhombic, space group $P2_12_12_1$ (no. 19), a = 8.5354(6) Å, b = 12.2468(9) Å, c = 21.4605(15) Å, V = 2243.3(3) Å^3, Z = 4, T = 100 K, μ(Cu Kα) = 2.204 mm^{-1}, $Dcalc$ = 1.573 g/cm^3, 87974 reflections measured (8.32° ≤ 2Θ ≤ 158.34°), 4820 unique (R_{int} = 0.0480, R_{sigma} = 0.0167) which were used in all calculations. The final R_1 was 0.0213 (I>=2u(I)) and wR_2 was 0.0507 (all data).

7.3.4 Acknowledgement

The purchase of the Bruker D8 VENTURE Photon III X-ray diffractometer was partially funded by NSF Award #CHE-1919350 to the UW–Madison Department of Chemistry.

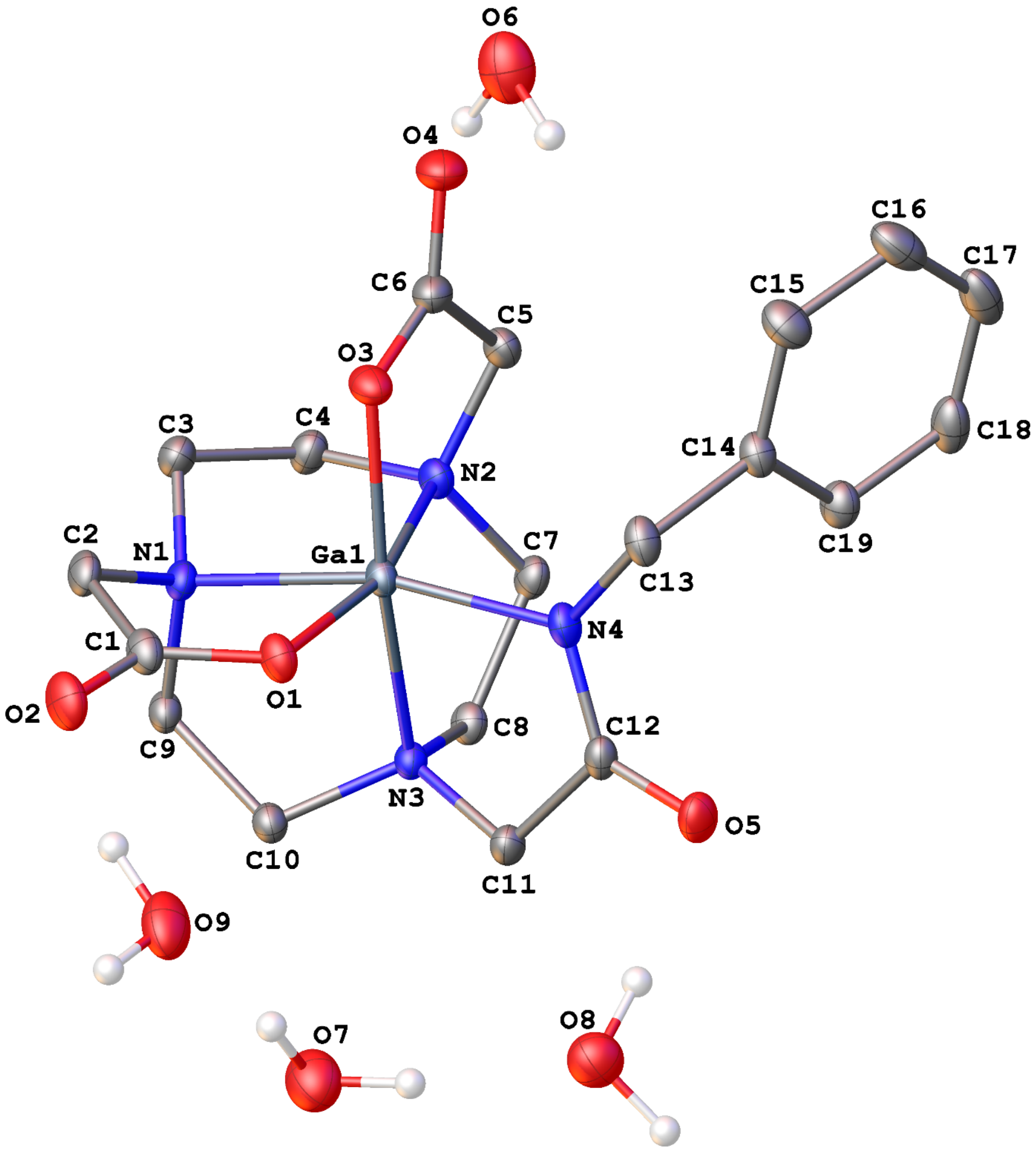

Figure S283. A molecular drawing of Ga(26) shown with 50% probability ellipsoids. All H atoms bonded to C atoms are omitted.

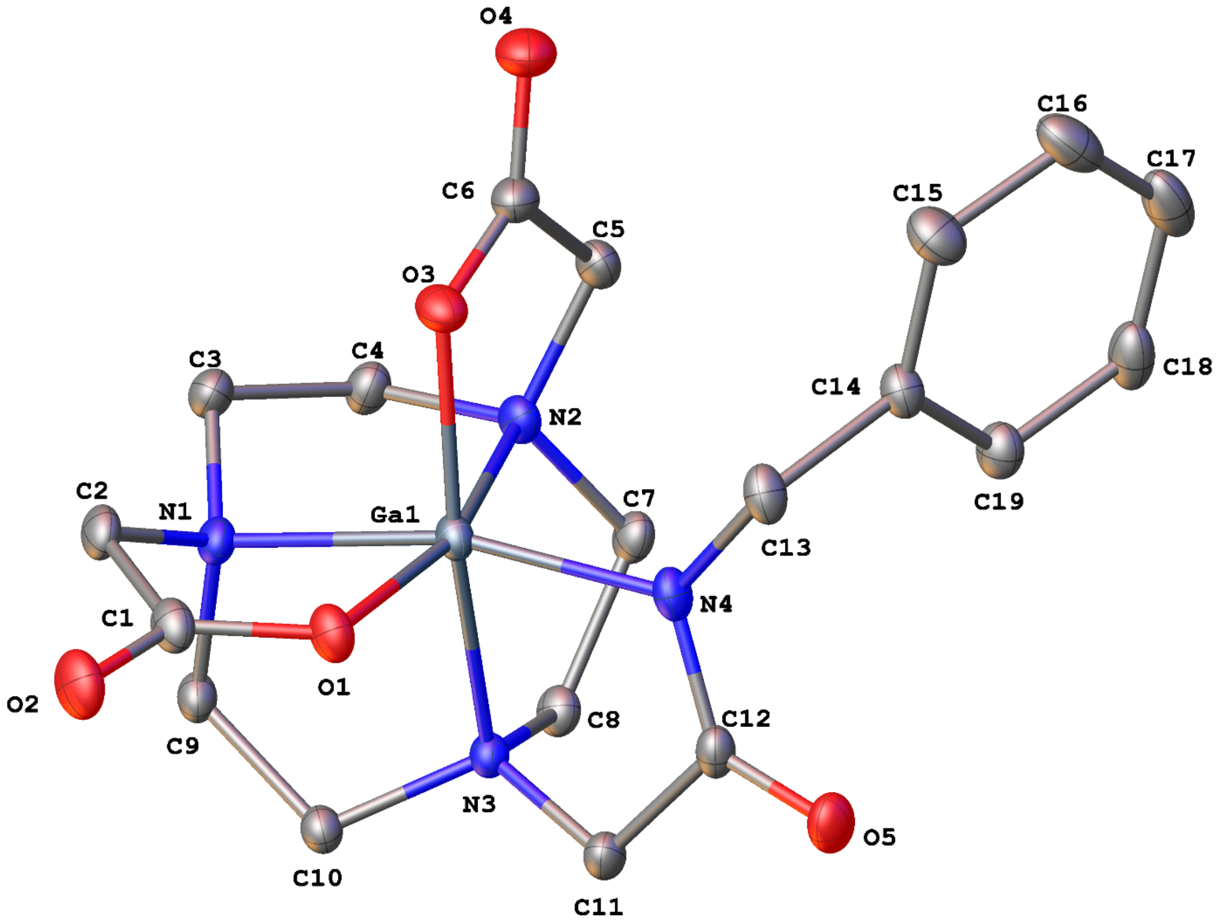

Figure S284. A drawing of the $C_{19}H_{25}N_4O_5Ga$ molecule in Ga(26) shown with 50% probability ellipsoids. All H atoms and water molecules are omitted.

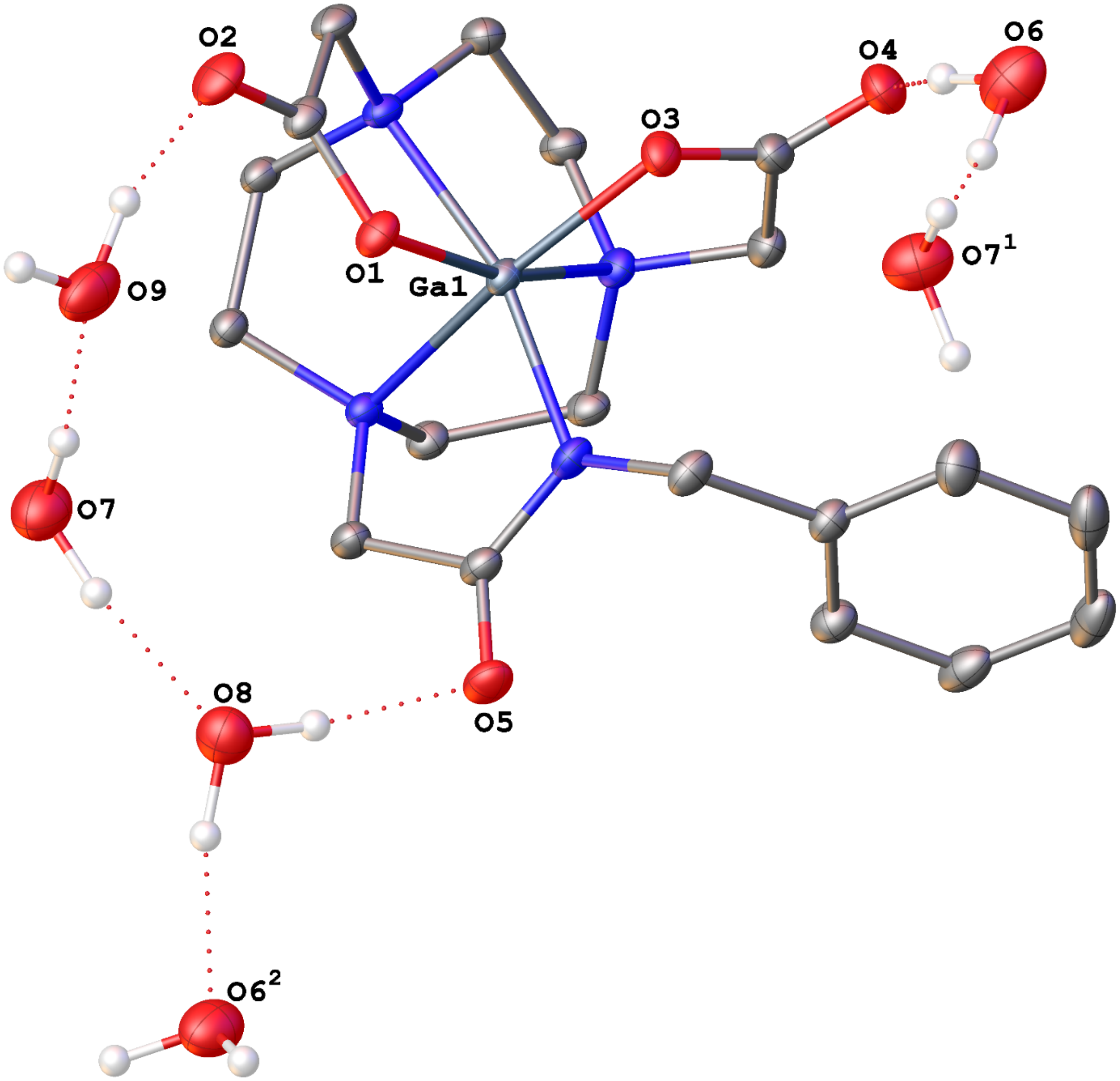

Figure S285. A molecular drawing of Ga(26) highlighting some of the hydrogen bonding. Drawn with 50% probability ellipsoids. All C and N labels and H atoms bonded to C atoms are omitted. Symmetry code: (1) 1-X,-0.5+Y,0.5-Z; (2) 1.5-X,1-Y,-0.5+Z.

**Table S20 Crystal data and structure refinement for Ga(26).**	
**Identification code**	Ga(26)
**Empirical formula**	$C_{19}H_{25}N_4O_5Ga\cdot 4H_2O$
**Formula weight**	531.219
**Temperature/K**	100
**Crystal system**	orthorhombic
**Space group**	$P2_12_12_1$
**a/Å**	8.5354(6)
**b/Å**	12.2468(9)
**c/Å**	21.4605(15)
**α/°**	90
**β/°**	90
**γ/°**	90
**Volume/Å^3**	2243.3(3)
**Z**	4
**ρ_{calc}g/cm^3**	1.573
**μ/mm^{-1}**	2.204
**F(000)**	1110.8
**Crystal size/mm^3**	0.103 × 0.035 × 0.024
**Radiation**	Cu Kα (λ = 1.54178)
**2Θ range for data collection/°**	8.32 to 158.34
**Index ranges**	-10 ≤ h ≤ 10, -15 ≤ k ≤ 15, -27 ≤ l ≤ 27
**Reflections collected**	87974
**Independent reflections**	4820 [R_{int} = 0.0480, R_{sigma} = 0.0167]
**Data/restraints/parameters**	4820/12/322
**Goodness-of-fit on F^2**	1.046
**Final R indexes [I>=2σ (I)]**	R_1 = 0.0213, wR_2 = 0.0506
**Final R indexes [all data]**	R_1 = 0.0214, wR_2 = 0.0507
**Largest diff. peak/hole / e Å^{-3}**	0.73/-0.17
**Flack parameter**	-0.023(5)

Table S21 Fractional Atomic Coordinates (×10^4) and Equivalent Isotropic Displacement Parameters (Å^2×10^3) for Ga(26). U_{eq} is defined as 1/3 of the trace of the orthogonalised U_{IJ} tensor.

**Atom**	***x***	***y***	***z***	**U(eq)**
**Ga1**	6194.4(2)	7563.45(15)	3361.43(9)	13.54(5)
**O1**	4368.7(13)	7201.8(9)	2849.1(6)	18.4(3)
**O2**	1895.1(14)	7656.7(11)	2639.1(6)	25.0(3)
**O3**	5519.4(14)	6746.1(10)	4098.7(6)	19.8(3)
**O4**	6274.6(18)	6248.3(11)	5052.7(6)	26.6(3)
**O5**	8736.9(16)	6067.2(10)	2047.7(6)	22.1(2)
**N1**	4823.5(16)	8974.7(12)	3553.5(6)	15.2(3)
**N2**	7729.9(16)	8290.8(12)	4016.2(6)	15.8(3)

**N3**	7335.0(16)	8609.5(11)	2725.9(6)	13.3(3)
**N4**	7557.5(17)	6457.2(11)	2984.6(7)	16.4(3)
**C1**	3115(2)	7770.1(14)	2937.7(8)	18.3(3)
**C2**	3192.7(19)	8612.6(15)	3463.4(8)	18.5(4)
**C3**	5197(2)	9273.0(15)	4206.5(8)	19.1(4)
**C4**	6981(2)	9280.6(15)	4293.7(8)	18.7(3)
**C5**	7971.7(18)	7428.3(16)	4486.7(7)	19.1(3)
**C6**	6475(2)	6755.1(14)	4567.8(8)	19.8(4)
**C7**	9148.2(19)	8565.8(15)	3649.3(8)	17.1(3)
**C8**	8678(2)	9159.5(13)	3049.4(7)	16.0(3)
**C9**	5268(2)	9856.9(14)	3107.3(8)	16.2(3)
**C10**	6112(2)	9406.9(13)	2531.5(8)	15.8(3)
**C11**	7855(2)	7887.5(14)	2214.4(8)	16.8(3)
**C12**	8112(2)	6709.0(14)	2424.0(8)	16.1(3)
**C13**	7636(2)	5314.1(13)	3178.5(8)	18.4(3)
**C14**	9031(2)	5036.8(13)	3587.4(8)	17.1(3)
**C15**	8854(3)	4261.4(14)	4057.6(9)	24.1(4)
**C16**	10139(3)	3920.7(16)	4411.4(9)	29.5(4)
**C17**	11606(2)	4380.1(17)	4308.8(9)	28.1(4)
**C18**	11786(2)	5180.3(16)	3854.1(9)	24.4(4)
**C19**	10503(2)	5499.0(14)	3488.2(8)	19.4(4)
**O6**	5221(2)	4049.2(14)	5101.6(8)	37.9(4)
**O7**	4893.1(18)	7822.3(13)	982.4(8)	37.4(4)
**O8**	7329.6(18)	6355.5(12)	902.2(7)	31.2(3)
**O9**	2128.1(18)	6778.1(12)	1427.1(8)	36.9(4)

Table S22 Anisotropic Displacement Parameters (Å^2×10^3) for Ga(26). The Anisotropic displacement factor exponent takes the form: $-2\pi^2[h^2a^{*2}U_{11}+2hka^*b^*U_{12}+\ldots]$.

Atom	U_{11}	U_{22}	U_{33}	U_{12}	U_{13}	U_{23}
**Ga1**	10.20(8)	13.34(9)	17.06(9)	0.77(8)	-0.69(7)	1.61(8)
**O1**	12.6(5)	18.0(6)	24.7(6)	0.2(4)	-3.8(5)	0.0(5)
**O2**	12.7(5)	27.0(7)	35.2(7)	-0.3(5)	-4.6(5)	0.3(6)
**O3**	17.1(6)	21.4(6)	20.8(6)	-0.6(5)	1.0(5)	7.1(5)
**O4**	27.8(7)	30.2(7)	21.7(6)	2.5(6)	3.9(6)	8.7(5)
**O5**	22.1(6)	21.6(6)	22.6(6)	6.4(6)	-0.3(6)	-5.6(5)
**N1**	10.8(6)	16.8(7)	18.0(7)	2.0(5)	2.1(5)	1.3(5)
**N2**	11.8(6)	19.4(7)	16.3(6)	1.3(5)	-2.3(5)	0.4(6)
**N3**	11.5(6)	13.0(6)	15.4(6)	0.9(5)	0.9(5)	0.7(5)
**N4**	14.4(7)	13.8(7)	20.9(7)	3.4(5)	-1.8(6)	-0.9(5)
**C1**	11.7(7)	18.7(8)	24.6(8)	-0.1(6)	-0.1(6)	3.7(6)
**C2**	10.9(7)	20.5(8)	24.0(9)	2.2(6)	2.9(6)	2.5(7)
**C3**	16.5(8)	23.2(9)	17.6(8)	3.8(7)	2.5(7)	0.2(7)
**C4**	15.1(8)	22.5(9)	18.4(8)	2.2(7)	1.4(7)	-2.5(7)
**C5**	16.1(7)	24.0(8)	17.1(7)	4.5(8)	-1.6(6)	1.8(8)
**C6**	19.6(9)	21.9(8)	17.9(8)	4.0(7)	3.2(7)	4.4(6)

**C7**	10.9(8)	21.8(8)	18.6(8)	0.9(6)	0.5(6)	-1.3(7)
**C8**	11.5(7)	17.4(7)	19.1(8)	-0.8(7)	0.1(7)	-0.1(6)
**C9**	12.9(7)	15.5(8)	20.2(8)	2.8(6)	1.7(6)	1.7(6)
**C10**	13.7(7)	15.9(7)	17.7(7)	2.2(7)	0.2(7)	1.7(6)
**C11**	15.9(8)	18.6(8)	15.9(8)	4.1(6)	0.6(6)	-0.6(6)
**C12**	13.3(7)	17.1(8)	18.0(8)	3.5(6)	-1.2(6)	-3.1(7)
**C13**	15.0(8)	14.3(8)	26.0(9)	1.6(6)	-2.5(7)	-1.3(6)
**C14**	14.8(8)	15.2(7)	21.3(8)	1.4(6)	-2.0(7)	-0.3(6)
**C15**	26.5(9)	18.9(8)	27.0(9)	0.2(8)	-3.9(8)	3.9(7)
**C16**	41.5(12)	22.5(9)	24.4(9)	6.7(8)	-9.7(9)	2.9(8)
**C17**	29.5(11)	30.2(10)	24.5(9)	12.1(8)	-12.3(8)	-7.2(8)
**C18**	16.6(8)	29.6(10)	27.0(9)	5.4(7)	-5.0(7)	-9.0(8)
**C19**	14.8(8)	21.6(8)	21.8(9)	0.5(7)	-1.9(7)	-2.1(7)
**O6**	31.5(8)	34.3(8)	47.9(9)	0.2(7)	-8.8(7)	-7.4(7)
**O7**	30.8(8)	36.9(9)	44.6(9)	0.9(6)	2.9(7)	6.8(7)
**O8**	33.2(8)	30.9(8)	29.6(7)	0.5(6)	-1.4(6)	-2.0(6)
**O9**	29.8(8)	25.6(7)	55.2(10)	5.7(6)	5.6(7)	8.8(7)

Table S23 Bond Lengths for Ga(26).

Atom	Atom	Length/Å	Atom	Atom	Length/Å
**Ga1**	O1	1.9579(12)	N3	C10	1.489(2)
**Ga1**	O3	1.9589(12)	N3	C11	1.478(2)
**Ga1**	N1	2.1276(14)	N4	C12	1.329(2)
**Ga1**	N2	2.1179(14)	N4	C13	1.462(2)
**Ga1**	N3	2.1092(14)	C1	C2	1.530(2)
**Ga1**	N4	1.9603(14)	C3	C4	1.534(2)
**O1**	C1	1.290(2)	C5	C6	1.531(2)
**O2**	C1	1.231(2)	C7	C8	1.532(2)
**O3**	C6	1.295(2)	C9	C10	1.533(2)
**O4**	C6	1.224(2)	C11	C12	1.528(2)
**O5**	C12	1.247(2)	C13	C14	1.517(2)
**N1**	C2	1.474(2)	C14	C15	1.394(2)
**N1**	C3	1.483(2)	C14	C19	1.395(2)
**N1**	C9	1.493(2)	C15	C16	1.398(3)
**N2**	C4	1.494(2)	C16	C17	1.390(3)
**N2**	C5	1.476(2)	C17	C18	1.392(3)
**N2**	C7	1.483(2)	C18	C19	1.403(2)
**N3**	C8	1.500(2)			

Table S24 Bond Angles for Ga(26).

Atom	Atom	Atom	Angle/˚	Atom	Atom	Atom	Angle/˚
**O3**	Ga1	O1	95.96(5)	C11	N3	C8	112.50(13)
**N1**	Ga1	O1	81.65(5)	C11	N3	C10	113.26(13)
**N1**	Ga1	O3	95.56(5)	C12	N4	Ga1	115.12(11)
**N2**	Ga1	O1	163.79(5)	C13	N4	Ga1	124.87(12)

**N2**	Ga1	O3	82.02(5)	C13	N4	C12	117.60(14)
**N2**	Ga1	N1	82.54(6)	O2	C1	O1	124.33(16)
**N3**	Ga1	O1	98.14(5)	C2	C1	O1	115.88(14)
**N3**	Ga1	O3	165.55(5)	C2	C1	O2	119.77(15)
**N3**	Ga1	N1	83.44(5)	C1	C2	N1	109.90(13)
**N3**	Ga1	N2	83.57(5)	C4	C3	N1	109.29(14)
**N4**	Ga1	O1	94.84(5)	C3	C4	N2	111.78(15)
**N4**	Ga1	O3	98.89(6)	C6	C5	N2	110.28(13)
**N4**	Ga1	N1	165.42(6)	O4	C6	O3	124.66(18)
**N4**	Ga1	N2	101.37(6)	C5	C6	O3	116.21(14)
**N4**	Ga1	N3	83.06(6)	C5	C6	O4	119.10(16)
**C1**	O1	Ga1	117.07(11)	C8	C7	N2	109.89(13)
**C6**	O3	Ga1	115.99(11)	C7	C8	N3	112.08(13)
**C2**	N1	Ga1	104.46(10)	C10	C9	N1	112.11(13)
**C3**	N1	Ga1	105.36(10)	C9	C10	N3	109.85(13)
**C3**	N1	C2	113.66(13)	C12	C11	N3	112.94(14)
**C9**	N1	Ga1	108.91(10)	N4	C12	O5	126.33(16)
**C9**	N1	C2	111.93(13)	C11	C12	O5	117.80(15)
**C9**	N1	C3	111.92(14)	C11	C12	N4	115.76(15)
**C4**	N2	Ga1	109.94(10)	C14	C13	N4	114.52(14)
**C5**	N2	Ga1	103.85(10)	C15	C14	C13	119.09(16)
**C5**	N2	C4	111.58(13)	C19	C14	C13	121.85(16)
**C7**	N2	Ga1	104.39(10)	C19	C14	C15	119.00(17)
**C7**	N2	C4	112.12(14)	C16	C15	C14	120.78(19)
**C7**	N2	C5	114.31(13)	C17	C16	C15	119.99(18)
**C8**	N3	Ga1	109.04(10)	C18	C17	C16	119.73(17)
**C10**	N3	Ga1	104.84(10)	C19	C18	C17	120.13(18)
**C10**	N3	C8	111.76(12)	C18	C19	C14	120.33(17)
**C11**	N3	Ga1	104.81(10)				

Table S25 Hydrogen Bonds for Ga(26).

D	H	A	d(D-H)/Å	d(H-A)/Å	d(D-A)/Å	D-H-A/°
**O6**	H6a	O7[1]	0.958(3)	1.831(9)	2.771(2)	166(3)
**O6**	H6b	O4	0.959(3)	1.953(16)	2.841(2)	153(3)
**O7**	H7c	O8	0.959(3)	1.809(8)	2.753(2)	168(3)
**O7**	H7d	O9	0.958(3)	1.896(5)	2.849(2)	173(2)
**O8**	H8c	O5	0.960(3)	1.807(5)	2.7588(19)	171(2)
**O8**	H8d	O6[2]	0.959(3)	1.808(7)	2.751(2)	167(2)
**O9**	H9c	O2	0.959(3)	1.929(12)	2.822(2)	154(2)

[1]1-X,-1/2+Y,1/2-Z; [2]3/2-X,1-Y,-1/2+Z

Table S26 Torsion Angles for Ga(26).

A	B	C	D	Angle/°	A	B	C	D	Angle/°
**Ga1**	O1	C1	O2	177.88(12)	C1	C2	N1	C3	-148.65(14)
**Ga1**	O1	C1	C2	-3.58(12)	C1	C2	N1	C9	83.35(15)

**Ga1**	O3	C6	O4	-176.43(12)	C2	N1	C3	C4	162.12(16)
**Ga1**	O3	C6	C5	1.67(12)	C2	N1	C9	C10	-95.99(14)
**Ga1**	N1	C2	C1	-34.34(10)	C3	N1	C9	C10	135.07(14)
**Ga1**	N1	C3	C4	48.35(11)	C3	C4	N2	C5	-95.42(16)
**Ga1**	N1	C9	C10	18.99(11)	C3	C4	N2	C7	134.89(15)
**Ga1**	N2	C4	C3	19.25(11)	C4	N2	C5	C6	83.89(14)
**Ga1**	N2	C5	C6	-34.48(10)	C4	N2	C7	C8	-70.55(14)
**Ga1**	N2	C7	C8	48.42(10)	C4	C3	N1	C9	-69.86(16)
**Ga1**	N3	C8	C7	17.92(11)	C5	N2	C7	C8	161.19(14)
**Ga1**	N3	C10	C9	48.12(10)	C6	C5	N2	C7	-147.58(13)
**Ga1**	N3	C11	C12	-26.39(11)	C7	C8	N3	C10	133.36(13)
**Ga1**	N4	C12	O5	-165.20(11)	C7	C8	N3	C11	-97.90(14)
**Ga1**	N4	C12	C11	10.73(13)	C8	N3	C10	C9	-69.86(14)
**Ga1**	N4	C13	C14	-101.15(13)	C8	N3	C11	C12	91.95(13)
**O1**	C1	C2	N1	27.54(16)	C9	C10	N3	C11	161.81(13)
**O2**	C1	C2	N1	-153.85(17)	C10	N3	C11	C12	-140.10(14)
**O3**	C6	C5	N2	24.36(16)	C11	C12	N4	C13	174.00(14)
**O4**	C6	C5	N2	-157.43(17)	C12	N4	C13	C14	97.38(16)
**O5**	C12	N4	C13	-1.9(2)	C13	C14	C15	C16	174.89(16)
**O5**	C12	C11	N3	-171.24(15)	C13	C14	C19	C18	-176.58(16)
**N1**	C3	C4	N2	-46.16(15)	C14	C15	C16	C17	1.9(2)
**N1**	C9	C10	N3	-46.09(15)	C14	C19	C18	C17	1.6(2)
**N2**	C7	C8	N3	-45.74(15)	C15	C14	C19	C18	0.45(19)
**N3**	C11	C12	N4	12.46(16)	C15	C16	C17	C18	0.1(2)
**N4**	C13	C14	C15	145.04(15)	C16	C15	C14	C19	-2.2(2)
**N4**	C13	C14	C19	-37.93(18)	C16	C17	C18	C19	-1.9(2)

Table S27 Hydrogen Atom Coordinates (Å×10^4) and Isotropic Displacement Parameters (Å^2×10^3) for Ga(26).

Atom	*x*	*y*	*z*	U(eq)
**H2a**	2743.0(19)	8245.9(15)	3901.8(8)	22.2(4)
**H2b**	2444.0(19)	9325.3(15)	3345.4(8)	22.2(4)
**H3a**	4712(2)	10093.5(15)	4314.1(8)	22.9(4)
**H3b**	4658(2)	8673.4(15)	4530.3(8)	22.9(4)
**H4a**	7257(2)	9315.7(15)	4799.2(8)	22.4(4)
**H4b**	7476(2)	10023.2(15)	4070.9(8)	22.4(4)
**H5a**	8945.6(18)	6886.1(16)	4337.6(7)	22.9(4)
**H5b**	8300.3(18)	7805.5(16)	4939.0(7)	22.9(4)
**H7a**	9933.2(19)	9095.9(15)	3930.4(8)	20.5(4)
**H7b**	9796.3(19)	7806.5(15)	3531.9(8)	20.5(4)
**H8a**	9701(2)	9188.4(13)	2729.5(7)	19.2(4)
**H8b**	8345(2)	10013.4(13)	3163.4(7)	19.2(4)
**H9a**	4195(2)	10298.0(14)	2957.2(8)	19.4(4)
**H9b**	6044(2)	10451.6(14)	3347.2(8)	19.4(4)
**H10a**	6665(2)	10088.4(13)	2269.8(8)	19.0(4)

**H10b**	5254(2)	9000.0(13)	2219.3(8)	19.0(4)
**H11a**	6962(2)	7899.7(14)	1838.1(8)	20.1(4)
**H11b**	8967(2)	8210.5(14)	2017.9(8)	20.1(4)
**H13a**	6546(2)	5108.8(13)	3435.5(8)	22.1(4)
**H13b**	7677(2)	4792.5(13)	2755.3(8)	22.1(4)
**H15**	7686(3)	3912.6(14)	4151.6(9)	29.0(4)
**H16**	9989(3)	3287.3(16)	4771.9(9)	35.4(5)
**H17**	12622(2)	4111.5(17)	4586.8(9)	33.7(5)
**H18**	12940(2)	5564.7(16)	3781.2(9)	29.3(5)
**H19**	10660(2)	6118.1(14)	3120.1(8)	23.3(4)
**H6a**	5350(40)	3662(18)	4717(7)	56.9(5)
**H6b**	5320(40)	4803(6)	4987(11)	56.9(5)
**H7c**	5680(20)	7284(15)	900(15)	56.1(5)
**H7d**	4010(20)	7414(18)	1133(14)	56.1(5)
**H8c**	7920(20)	6290(30)	1281(6)	46.8(5)
**H8d**	8100(20)	6280(30)	580(8)	46.8(5)
**H9c**	1730(30)	7000(30)	1825(6)	55.3(5)
**H9d**	1218(18)	6690(30)	1171(10)	55.3(5)

8 References

1. Śmiłowicz, D.; Schlyer, D.; Boros, E.; Meimetis, L. Evaluation of a Radio-IMmunoStimulant (RIMS) in a Syngeneic Model of Murine Prostate Cancer and ImmunoPET Analysis of T-cell Distribution. *Mol. Pharm.* **2022,** *19* (9), 3217-3227
2. Kelderman, C. A.; Glaser, O. M.; Whetter, J. N.; Aluicio-Sarduy, E.; Mixdorf, J. C.; Sanders, K. M.; Guzei, I. A.; Barnhart, T. E.; Engle, J. W.; Boros, E. Charting the coordinative landscape of the ^{18}F-Sc/^{44}Sc/^{177}Lu triad with the tri-aza-cyclononane (tacn) scaffold. *Chem. Sci*. **2024**, *15*(43), 17927–17936.
3. Boros, E.; Caravan, P. Structure-Relaxivity Relationships of Serum Albumin Targeted MRI Probes Based on a Single Amino Acid Gd. *J. Med. Chem.* **2013**, *56*(4), 1782-1786.
4. Bruker AXS LLC (2024). APEX6. Version 2024.9-0. Madison, Wisconsin, USA.
5. Krause, L., Herbst-Irmer, R., Sheldrick, G. M. & Stalke, D. (2015). *J. Appl. Cryst.* 48. 3-10.
6. Sheldrick, G. M. (2013b). *XPREP*. Version 2013/1. Georg-August-Universität Göttingen, Göttingen, Germany.
7. Sheldrick, G. M. (2013a). The *SHELX* homepage, http://shelx.uni-ac.gwdg.de/SHELX/.
8. Sheldrick, G. M. (2015a). *Acta Cryst. A*, 71, 3-8.
9. Sheldrick, G. M. (2015b). *Acta Cryst. C*, 71, 3-8.
10. Dolomanov, O. V., Bourhis, L. J., Gildea, R. J., Howard, J. A. K. & Puschmann, H. (2009). *J. Appl. Crystallogr.* 42, 339-341.
11. Guzei, I. A. (2007-2022). Programs *Gn*. University of Wisconsin-Madison, Madison, Wisconsin, USA.
12. Bourhis, L.J., Dolomanov, O.V., Gildea, R.J., Howard, J.A.K., Puschmann, H. (2015). *Acta Cryst. A*. 71, 59-75.
13. Neese, F. (2018). *Wiley Interdiscip. Rev.: Comput. Mol. Sci*. 8, e1327
